# Supplementary material for: Stretchable chiral pockets for palladium-catalyzed highly chemo- and enantioselective allenylation
Source: Nat Commun. 2021 Apr 23;12:2416. doi: 10.1038/s41467-021-22498-1 (PMC8065118; doi:10.1038/s41467-021-22498-1)
Supplement: Supplementary file 1 — Supplementary Information [file 41467_2021_22498_MOESM1_ESM.pdf]

Stretchable Chiral Pockets for Palladium-Catalyzed Highly Chemo- and Enantio-selective Allenylation

Yuchen Zhang,[a] Xue Zhang *[b] and Shengming Ma*[a,b]

[a] Laboratory of Molecular Recognition and Synthesis, Department of Chemistry, Zhejiang University, Hangzhou 310027, Zhejiang, P. R. China.

[b] State Key Laboratory of Organometallic Chemistry, Shanghai Institute of Organic Chemistry, Chinese Academy of Sciences, 345 Lingling Lu, Shanghai 200032, P. R. China.

E-mail: xzhang@sioc.ac.cn, masm@sioc.ac.cn

Supplementary Information

General Information	2
Syntheses of ligands **ZYC-Phos**	3-10
Syntheses of starting materials **1a~1d** and **2p**, **2u**, **2v**	10-17
Syntheses of products **3aa** and **4aa**	17-19
Syntheses of products	19-50
Gram-scale reaction and synthetic applications	51-56
Synthesis of the Pd(II)-**DACH-ZYC-Phos-C1** complex	56-58
DFT calculations	58-115
NMR and HPLC spectra	116-319
Supplementary References	320-324

General Information

^{1}H NMR and ^{13}C NMR spectra were recorded in $CDCl_3$ using a Bruker AM 300 MHz NMR spectrometer (^{1}H at 300 MHz, ^{13}C at 75 MHz). All ^{1}H NMR experiments were measured with tetramethylsilane (0 ppm) in $CDCl_3$ as the internal reference; ^{13}C NMR experiments were measured in relative to the signal of $CDCl_3$ (77.0 ppm); ^{19}F NMR experiments were measured in relative to the signal of residual $CFCl_3$ (0 ppm) in $CDCl_3$; ^{31}P NMR experiments were measured with 85% phosphoric acid (0 ppm) as the external reference. IR spectra were recorded with a Perkin–Elmer 983G instrument. Elemental analyses were measured with a Carlo-Erba EA1110 elementary analysis instrument. Mass spectrometry was performed with a HP 5989A system. High-resolution mass spectrometry was determined with a Finnigan MAT 8430 or a Bruker APEXIII instrument. $Pd_2(dba)_3$•$CHCl_3$ was purchased from *J&K Scientific Co., Ltd.* Toluene was dried over Na wire and distilled right before use. Petroleum ether (60-90 °C) was used for chromatography on silica gel. Unless otherwise indicated, chemicals and solvents were purchased from commercial suppliers. All the temperatures are referred to the oil baths used. Benzyl buta-2,3-dienyl carbonate[1] and pyrazol-5-ones **2f**,[2,3] **2l**,[2,3] **2o**,[2,3] **2a**,[4] **2g**,[4] **2n**,[4] **2q**,[4] **2r**,[4] **2s**,[4] **2b-2e**,[5] **2h**,[5] **2i**,[5] **2k**,[5] **2m**,[5] **2j**[6], and **2t**[7] were prepared according to previous literatures. We mixed the compounds (*S*)-**3**, (3*aS*,5*S*)-**5**, (*S*,*Z*)-**6** and (*S*)-**7** with their corresponding enantiomers for ee determination.

1. Synthesis of ZYC-Phos.

1.1 Synthesis of *N*,*N'*-((*R*,*R*)-cyclohexane-1,2-diyl)bis(3-(diphenylphosphanyl) propanamide) (*R*,*R*)-**DACH-ZYC-Phos-C2**. (zyc-3-186，zyc-3-118)

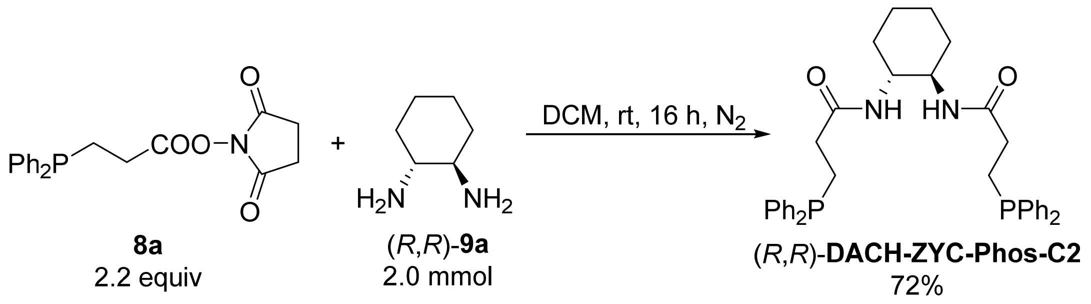

To a flame-dried Schlenk tube were added **8a**[8,9] (1.5641 g, 4.4 mmol)/DCM (4.0 mL) and (*R*,*R*)-**9a** (0.2286 g, 2.0 mmol, 99% ee)/DCM (2.0 mL). The reaction was complete after being stirred at room temperature for 16 hours as monitored by TLC. The reaction was quenched with water (10 mL) and the resulting mixture was transferred to a separatory funnel and extracted with DCM (10 mL × 3). The combined organic phase was dried over anhydrous Na_2SO_4. After filtration and evaporation of the solvent，the crude residual was purified by chromatography on silica gel (eluent: DCM (300 mL) to DCM/MeOH = 100/1 (500 mL)) to afford a solid. The resulting solid was recrystallized from ethyl acetate and collected by suction filtration washed with ethyl acetate. The second part of the product was collected by evaporation of some of the solvent and collected by suction filtration by washing with hexane to afford pure (*R*,*R*)-**DACH-ZYC-Phos-C2** (totally 0.8526 g, 72%) as a solid: m.p. 152.4-153.2 °C (ethyl acetate); $[\alpha]_D^{20}$ = +0.3 (c = 1.045, $CHCl_3$); ^1H NMR (300 MHz, $CDCl_3$) δ 7.46-7.32 (m, 8 H, ArH), 7.32-7.22 (m, 12 H, ArH), 6.11 (d, *J* = 6.9

Hz, 2 H, NH × 2), 3.68-3.51 (m, 2 H, NCH × 2), 2.37-2.10 (m, 8 H, CH_2 × 4), 2.03-1.90 (m, 2 H, CH_2), 1.78-1.62 (m, 2 H, CH_2), 1.37-1.08 (m, 4 H, CH_2 × 2); ^{13}C NMR (75 MHz, $CDCl_3$) δ 172.8, 172.7, 137.74 (d, J = 12.4 Hz), 137.69 (d, J = 12.4 Hz), 132.7, 132.6, 132.5, 132.4, 128.61, 128.58, 128.4, 128.3, 53.6, 32.7 (d, J = 18.6 Hz), 32.0, 24.5, 23.4, 23.2; ^{31}P NMR (121.5 MHz, $CDCl_3$) δ -15.9; IR (KBr) ν (cm^{-1}) 3273, 3069, 2933, 2855, 1639, 1546, 1476, 1433, 1257; MS (EI): *m/z* (%) 594 ($[M]^+$, 11.45), 256 (100); HRMS calcd. for $C_{36}H_{40}N_2O_2P_2$ [M^+]: 594.2565; Found: 594.2563.

1.2 Synthesis of *N*,*N'*-((*R*,*R*)-1,2-diphenylethane-1,2-diyl)bis(3-(diphenylphosphanyl) propanamide) (*R*,*R*)-**DADPE-ZYC-Phos-C2**. (zyc-3-181)

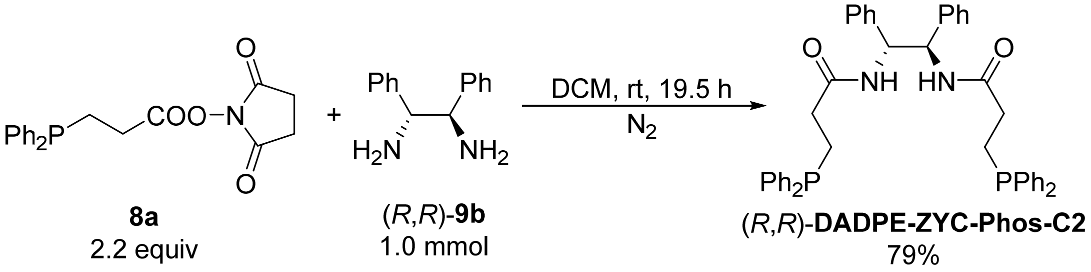

To a flame-dried Schlenk tube were added (*R*,*R*)-**9b** (212.6 mg, 1.0 mmol, 99% ee), **8a** (781.9 mg, 2.2 mmol), and DCM (8.0 mL). The reaction was complete after being stirred at room temperature for 19.5 hours as monitored by TLC. The resulting mixture was quenched with water (10 mL) and extracted with DCM (10 mL × 3). The combined organic phase was dried over anhydrous Na_2SO_4. After filtration and evaporation of the solvent，the crude residual was purified by chromatography on silica gel (eluent: DCM (500 mL) to DCM/MeOH = 100/1 (600 mL)) to afford a solid. The resulting solid was recrystallized from ethyl acetate and collected by suction filtration washed with ethyl acetate. The second part of the product was collected by

evaporation of some of the solvent and collected by suction filtration by washing with hexane to afford (*R*,*R*)-**DADPE-ZYC-Phos-C2** (totally 561.3 mg, 79%, purity = 98%) as a solid: m.p. 140.8-141.9 °C (ethyl acetate); $[\alpha]_D^{20}$ = -64.5 (c = 1.05, $CHCl_3$); ^1H NMR (300 MHz, $CDCl_3$) δ 7.44-7.22 (m, 20 H, ArH), 7.20-7.11 (m, 6 H, ArH), 7.11-7.01 (m, 4 H, ArH), 6.76-6.62 (m, 2 H, NH × 2), 5.28-5.16 (m, 2 H, NCH × 2), 2.36-2.13 (m, 8 H, CH_2 × 4); ^{13}C NMR (75 MHz, $CDCl_3$) δ 173.1, 172.9, 138.5, 137.73 (d, *J* = 12.4 Hz), 137.71 (d, *J* = 12.4 Hz), 132.8, 132.7, 132.6, 132.5, 128.8, 128.7, 128.6, 128.5, 127.8, 127.5, 59.4, 32.7 (d, *J* = 17.9 Hz), 23.3, 23.2; ^{31}P NMR (121.5 MHz, $CDCl_3$) δ -16.1; IR (KBr) ν (cm^{-1}) 3287, 3068, 3028, 2929, 1644, 1535, 1493, 1476, 1433, 1364, 1248; MS (EI): *m/z* (%) 692 ($[M]^+$, 5.29), 346 (100); HRMS calcd. for $C_{44}H_{42}N_2O_2P_2$ $[M^+]$: 692.2722; Found: 692.2722.

1.3 Synthesis of *N,N'*-((*S*,*S*)-1,2-diphenylethane-1,2-diyl)bis(3-(diphenylphosphanyl) propanamide) (*S*,*S*)-**DADPE-ZYC-Phos-C2**. (zyc-4-15)

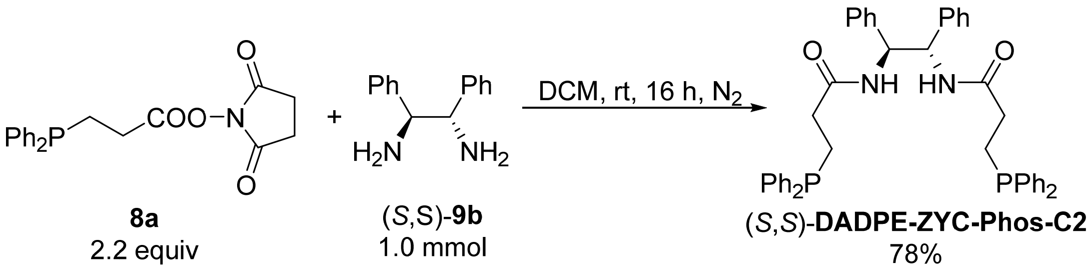

8a 2.2 equiv; (*S*,S)-**9b** 1.0 mmol; (*S*,*S*)-**DADPE-ZYC-Phos-C2** 78%

To a flame-dried Schlenk tube were added (*S*,*S*)-**9b** (212.5 mg, 1.0 mmol, 99% ee), **8a** (782.3 mg, 2.2 mmol), and DCM (8.0 mL). The reaction was complete after being stirred at room temperature for 16 hours as monitored by TLC. The reaction was quenched with water (10 mL) and the resulting mixture was transferred to a separatory funnel and extracted with DCM (10 mL × 3). The combined organic phase

was dried over anhydrous Na_2SO_4. After filtration and evaporation of the solvent，the crude residual was purified by chromatography on silica gel (eluent: DCM/MeOH = 100/1 (400 mL)) to afford a solid. The resulting solid was recrystallized from ethyl acetate and collected by suction filtration washed with ethyl acetate. The second part of the product was collected by evaporation of some of the solvent and collected by suction filtration by washing with hexane to afford pure (*S*,*S*)-**DADPE-ZYC-Phos-C2** (totally 538.6 mg, 78%) as a solid: m.p. 143.6-144.4 °C (ethyl acetate); $[\alpha]_D^{20}$ = +66.9 (c = 0.98, $CHCl_3$); ^{1}H NMR (300 MHz, $CDCl_3$) δ 7.41-7.19 (m, 20 H, ArH), 7.19-6.99 (m, 10 H, ArH), 6.97-6.82 (m, 2 H, NH × 2), 5.28-5.15 (m, 2 H, NCH × 2), 2.33-2.09 (m, 8 H, CH_2 × 4); ^{13}C NMR (75 MHz, $CDCl_3$) δ 173.1, 172.9, 138.6, 137.74 (d, *J* = 12.4 Hz), 137.70 (d, *J* = 13.1 Hz), 132.8, 132.7, 132.5, 132.4, 128.71, 128.66, 128.5, 128.43, 128.42, 127.7, 127.4, 59.3, 32.7 (d, *J* = 17.9 Hz), 23.3, 23.2; ^{31}P NMR (121.5 MHz, $CDCl_3$) δ -16.0; IR (KBr) ν (cm^{-1}) 3285, 3067, 3050, 3024, 2912, 1642, 1535, 1491, 1478, 1431, 1364, 1250, 1049; MS (EI): *m/z* (%) 692 ($[M]^+$, 7.23), 346 (100); Anal. Calcd. for $C_{44}H_{42}N_2O_2P_2$ (%): C 76.28, H 6.11, N 4.04; Found: C 76.09, H 6.14, N 3.93.

1.4 Synthesis of *N,N'*-((*R*,*R*)-cyclohexane-1,2-diyl)bis(4-(diphenylphosphanyl) butanamide) (*R*,*R*)-**DACH-ZYC-Phos-C3**. (zyc-3-198)

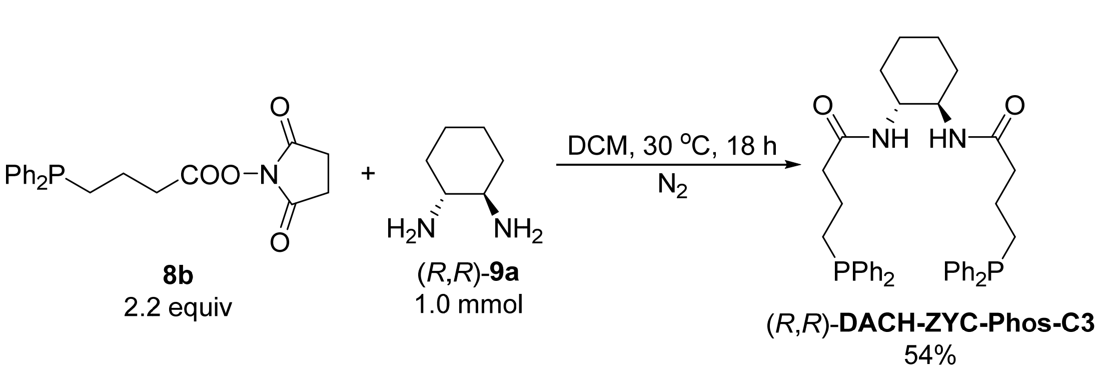

To a flame-dried Schlenk tube were added **8b** (890.5 mg, 2.2 mmol)/DCM (2.0 mL) and (*R*,*R*)-**9a** (117.1 mg, 1.0 mmol, 99% ee)/DCM (1.0 mL). The reaction was complete after being stirred at 30 °C for 18 hours as monitored by TLC. The reaction was quenched with water (5 mL) and the resulting mixture was transferred to a separatory funnel and extracted with DCM (10 mL × 3). The combined organic phase was dried over anhydrous Na_2SO_4. After filtration and evaporation of the solvent， the crude residual was purified by chromatography on silica gel (eluent: DCM (300 mL) to DCM/MeOH = 100/1 (600 mL)) to afford impure (*R*,*R*)-**DACH-ZYC-Phos-C3** (474.6 mg). Pure (*R*,*R*)-**DACH-ZYC-Phos-C3** (344.8 mg, 54%) was obtained via recrystallization (DCM/*n*-hexane) as a solid: m.p. 166.8-167.6 °C; $[\alpha]_D^{20}$ = +22.4 (c = 1.00, $CHCl_3$); ^{1}H NMR (300 MHz, $CDCl_3$) δ 7.55-7.09 (m, 20 H, ArH), 6.03 (d, *J* = 4.5 Hz, 2 H, NH × 2), 3.72-3.53 (m, 2 H, NCH × 2), 2.28-2.06 (m, 4 H, CH_2 × 2), 2.06-1.88 (m, 6 H, CH_2 × 3), 1.84-1.59 (m, 6 H, CH_2 × 3), 1.38-1.08 (m, 4 H, CH_2 × 2); ^{13}C NMR (75 MHz, $CDCl_3$) δ 172.9, 138.31 (d, *J* = 12.4 Hz), 138.27 (d, *J* = 13.1 Hz), 132.7, 132.5, 128.5, 128.4, 128.3, 53.6, 37.5 (d, *J* = 13.1 Hz), 32.2, 27.4, 27.3, 24.6, 22.3, 22.0; ^{31}P NMR (121.5 MHz, $CDCl_3$) δ -17.2; IR (KBr) ν (cm^{-1}) 3344, 3068, 3045, 2943, 2854, 1636, 1521, 1478, 1432, 1409, 1375; MS (EI): *m/z* (%) 622 ([M]$^+$, 47.62), 270 (100); Anal. Calcd. for $C_{38}H_{44}N_2O_2P_2$ (%): C 73.29, H 7.12, N 4.50;

Found: C 73.11, H 7.13, N 4.30.

1.5 Synthesis of *N,N'*-(*R*,*R*)--(cyclohexane-1,2-diyl)bis(2-(diphenylphosphanyl) acetamide) (*R*,*R*)-**DACH-ZYC-Phos-C1**. (zyc-4-7)

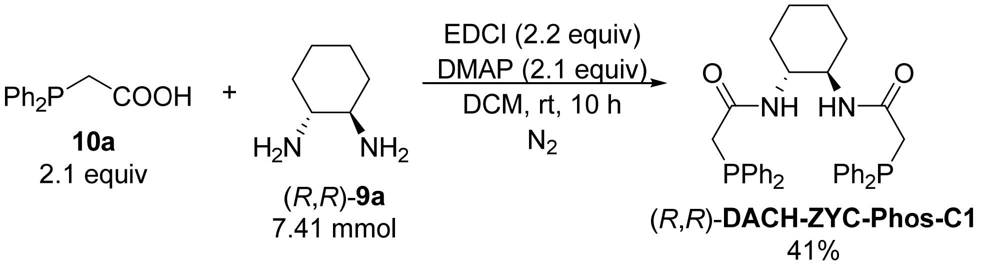

To a flame-dried Schlenk flask were added (*R*,*R*)-**9a** (0.8460 mg, 7.41 mmol, 99% ee)/DCM (10.0 mL), **10a**[8] (3.8000 mg, 15.56 mmol)/DCM (20.0 mL), and DMAP (1.8994 mg, 15.56 mmol) sequentially. The solution of EDCl (3.1255 mg, 16.30 mmol) in DCM (20.0 mL) was added dropwise to the resulting mixture within 5 minutes. The reaction was complete after being stirred at room temperature for 10 hours as monitored by TLC. The resulting mixture was transferred to a separatory funnel and washed with water (100 mL × 3). The organic phase was dried over anhydrous Na_2SO_4. After filtration and evaporation of the solvent，the crude residual was purified by chromatography on silica gel (eluent: DCM/MeOH = 1000/1 (150 mL) to 100/1 (1000 mL)) to afford (*R*,*R*)-**DACH-ZYC-Phos-C1** (1718.7 mg, 41%) as a solid: m.p. 217.4-218.0 °C (DCM/*n*-hexane); $[\alpha]_D^{20}$ = +32.9 (c = 0.995, $CHCl_3$); ^1H NMR (300 MHz, $CDCl_3$) δ 7.61-7.11 (m, 20 H, ArH), 5.99 (d, *J* = 3.9 Hz, 2 H, NH × 2), 3.62-3.36 (m, 2 H, NCH × 2), 2.87 (d, *J* = 15.3 Hz, 2 H, one proton of PCH_2 × 2), 2.82 (d, *J* = 14.1 Hz, 2 H, one proton of PCH_2 × 2), 1.86-1.50 (m, 4 H, CH_2 × 2), 1.33-1.06 (m, 2 H, CH_2), 1.06-0.80 (m, 2 H, CH_2); ^{13}C NMR (75 MHz, $CDCl_3$) δ

170.0, 169.9, 137.5 (d, J = 17.3 Hz), 137.3 (d, J = 17.3 Hz), 133.0, 132.7, 132.5, 132.3, 129.1, 128.8, 128.6, 128.54, 128.50, 128.46, 53.6, 37.4 (d, J = 20.7 Hz), 32.0, 24.5; ^{31}P NMR (121.5 MHz, $CDCl_3$) δ -16.8; IR (KBr) ν (cm^{-1}) 3292, 3067, 3052, 2933, 2912, 2854, 1627, 1530, 1480, 1433, 1401, 1328, 1147; MS (EI): *m/z* (%) 566 ($[M]^+$, 29.26), 381 (100); Anal. Calcd. for $C_{34}H_{36}N_2O_2P_2$ (%): C 72.07, H 6.40, N 4.94; Found: C 72.01, H 6.42, N 4.76.

1.6 Synthesis of *N,N'*-(*S,S*)--(cyclohexane-1,2-diyl)bis(2-(diphenylphosphanyl) acetamide) (*S,S*)-**DACH-ZYC-Phos-C1**. (zyc-4-52)

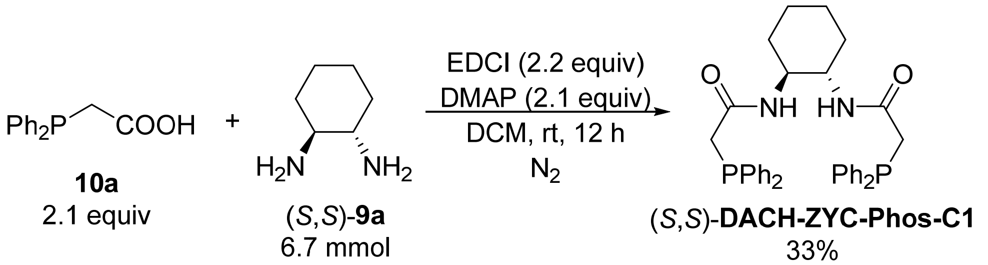

To a flame-dried Schlenk flask were added (*S,S*)-**9a** (764.1 mg, 6.7 mmol, 98% ee)/DCM (10.0 mL), **10a** (3451.7 mg, 14.1 mmol)/DCM (20.0 mL), DMAP (1723.0 mg, 14.1 mmol), and EDCl (2826.6 mg, 14.74 mmol)/DCM (20.0 mL) sequentially. The reaction was complete after being stirred at room temperature for 12 hours as monitored by TLC and the resulting mixture was transferred to a separatory funnel and washed with water (100 mL × 3). The organic phase was dried over anhydrous Na_2SO_4. After filtration and evaporation of the solvent, the crude residual was purified by chromatography on silica gel (eluent: DCM/MeOH = 1000/1 (300 mL) to 100/1 (800 mL)) to afford (*S,S*)-**DACH-ZYC-Phos-C1** (1252.3 mg, 33%) as a solid: m.p. 216.9-217.9 °C (DCM/*n*-hexane); $[\alpha]_D^{20}$ = -33.3 (c = 0.99, $CHCl_3$); ^{1}H NMR (300

MHz, $CDCl_3$) δ 7.53-7.20 (m, 20 H, ArH), 5.99 (d, *J* = 5.1 Hz, 2 H, NH × 2), 3.59-3.40 (m, 2 H, NCH × 2), 2.87 (d, *J* = 14.1 Hz, 2 H, one proton of PCH_2 × 2), 2.82 (d, *J* = 14.1 Hz, 2 H, one proton of PCH_2 × 2), 1.86-1.70 (m, 2 H, CH_2), 1.70-1.54 (m, 2 H, CH_2), 1.28-1.08 (m, 2 H, CH_2), 1.06-0.84 (m, 2 H, CH_2); ^{13}C NMR (75 MHz, $CDCl_3$) δ 170.0, 169.9, 137.5 (d, *J* = 15.8 Hz), 137.3 (d, *J* = 17.3 Hz), 133.0, 132.7, 132.5, 132.3, 129.1, 128.8, 128.6, 128.55, 128.51, 128.47, 53.6, 37.4 (d, *J* = 20.7 Hz), 32.0, 24.5; ^{31}P NMR (121.5 MHz, $CDCl_3$) δ -16.8; IR (KBr) ν (cm^{-1}) 3292, 3067, 3051, 2931, 2854, 1628, 1529, 1481, 1433, 1399, 1327, 1190, 1143; MS (EI): *m/z* (%) 566 ([M]$^+$, 32.57), 381 (100); Anal. Calcd. for $C_{34}H_{36}N_2O_2P_2$ (%): C 72.07, H 6.40, N 4.94; Found: C 72.03, H 6.44, N 4.81.

2. Synthesis of starting materials.

2.1 2,3-Allenylic carbonates.[1]

2.1.1 Synthesis of benzyl 2-butyl-2,3-butadienyl carbonate **1a**. (zyc-3-144)

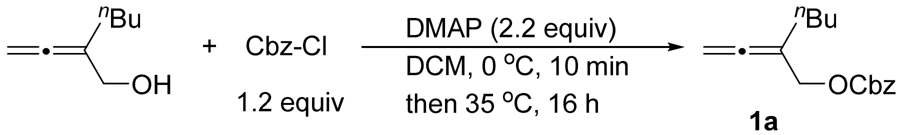

1a
69%

Typical Procedure I: To a three-necked flask were added DMAP (8.3870 g, 68.65 mmol), 2-butyl-2,3-butadienol (3.9339 g, 31.2 mmol), and DCM (40 mL) sequentially at room temperature. The resulting solution was cooled to 0 °C followed by the addition of Cbz-Cl (6.3860 g, 37.43 mmol) dropwise at this temperature within 10 min. The flask was put into a pre-heat oil bath and the reaction was complete after being stirred at 35 °C for 16 hours as monitored by TLC. The resulting mixture was

transferred to a separatory funnel followed by the addition of water (40 mL) and DCM (40 mL). After the separation of the organic phase, the aqueous phase was extracted with DCM (20 mL × 2). The combined organic phase was washed with brine and dried over anhydrous Na_2SO_4. After filtration and evaporation of the solvent，the crude residual was purified by chromatography on silica gel (eluent: petroleum ether (60-90 °C)/ethyl acetate = 50/1) to afford **1a** (5.6413 g, 69%) as a liquid: ^{1}H NMR (300 MHz, $CDCl_3$) δ 7.43-7.27 (m, 5 H, ArH), 5.16 (s, 2 H, OCH_2Ph), 4.83-4.75 (m, 2 H, $=CH_2$), 4.61 (t, J = 2.1 Hz, 2 H, OCH_2), 2.05-1.94 (m, 2 H, CH_2), 1.49-1.24 (m, 4 H, CH_2 × 2), 0.89 (t, J = 7.1 Hz, 3 H, CH_3); ^{13}C NMR (75 MHz, $CDCl_3$) δ 206.8, 155.0, 135.2, 128.5, 128.4, 128.3, 99.1, 76.9, 69.5, 68.5, 29.4, 28.5, 22.2, 13.8; IR (neat) ν (cm^{-1}) 3035, 2957, 2931, 2872, 2860, 1960, 1747, 1498, 1456, 1389, 1366, 1258; MS (EI): *m/z* (%) 173 ($[M-Pr-CO_2]^+$, 4.33), 169 ($[M-Bn]^+$, 1.33), 91 (100); HRMS calcd. for $C_{16}H_{20}O_3$ [M^+]: 260.1412; Found: 260.1411.

2.1.2 Synthesis of benzyl 2-methyl-2,3-butadienyl carbonate **1b**. (zyc-4-114)

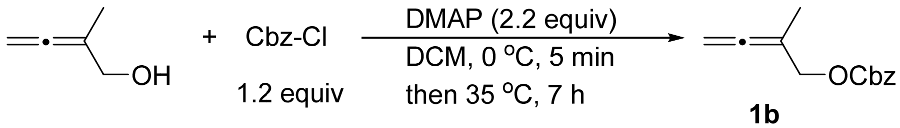

1b
82%

Following **Typical Procedure** Ⅰ, the reaction of DMAP (4.6523 g, 38.1 mmol), 2-methyl-2,3-butadienol (1.4593 g, 17.35 mmol)/DCM (18 mL), and Cbz-Cl (3.5471 g, 20.8 mmol) afforded **1b** (3.1143 g, 82%) (eluent: petroleum ether (60-90 °C)/ethyl acetate = 100/1) as a liquid: ^{1}H NMR (300 MHz, $CDCl_3$) δ 7.48-7.26 (m, 5 H, ArH), 5.17 (s, 2 H, OCH_2Ph), 4.78-4.68 (m, 2 H, $=CH_2$), 4.59 (t, J = 2.3 Hz, 2 H, OCH_2),

1.73 (t, J = 3.3 Hz, 3 H, CH_3); ^{13}C NMR (75 MHz, $CDCl_3$) δ 207.2, 155.0, 135.2, 128.53, 128.47, 128.3, 94.3, 75.7, 69.6, 69.2, 15.5; IR (neat) ν (cm^{-1}) 3066, 3035, 2986, 2951, 1962, 1755, 1747, 1498, 1456, 1390, 1375, 1362, 1258; MS (ESI): *m/z* 241 ($[M+Na]^+$), 236 ($[M+NH_4]^+$), 219 ($[M+H]^+$); HRMS calcd. for $C_{13}H_{14}O_3$ [M^+]: 218.0943; Found: 218.0945.

2.1.3 Synthesis of benzyl 2-octyl-2,3-butadienyl carbonate **1c**. (zyc-4-37)

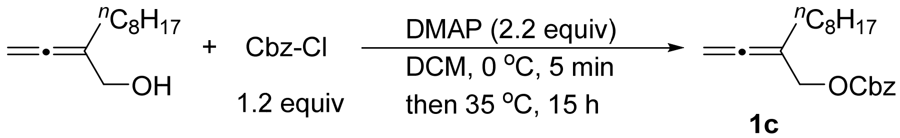

1c
80%

Following **Typical Procedure** Ⅰ, the reaction of DMAP (4.0080 g, 32.8 mmol), 2-octyl-2,3-butadienol (2.7195 g, 14.9 mmol)/DCM (15 mL), and Cbz-Cl (3.0544 g, 17.9 mmol) afforded **1c** (3.8442 g, 80%, purity = 98%) (eluent: petroleum ether (60-90 °C)/ethyl acetate = 100/1 (300 mL) to 50/1 (500 mL)) as a liquid: ^1H NMR (300 MHz, $CDCl_3$) δ 7.45-7.28 (m, 5 H, ArH), 5.16 (s, 2 H, OCH_2Ph), 4.83-4.75 (m, 2 H, $=CH_2$), 4.61 (t, J = 1.8 Hz, 2 H, OCH_2), 2.06-1.92 (m, 2 H, CH_2), 1.53-1.37 (m, 2 H, CH_2), 1.37-1.17 (m, 10 H, CH_2 × 5), 0.88 (t, J = 6.6 Hz, 3 H, CH_3); ^{13}C NMR (75 MHz, $CDCl_3$) δ 206.8, 155.0, 135.2, 128.5, 128.4, 128.3, 99.2, 76.9, 69.6, 68.5, 31.8, 29.3, 29.20, 29.16, 28.9, 27.2, 22.6, 14.1; IR (neat) ν (cm^{-1}) 3066, 3035, 2955, 2926, 2855, 1960, 1753, 1747, 1498, 1456, 1389, 1366, 1255; MS (ESI): *m/z* 340 ($[M+Na]^+$), 334 ($[M+NH_4]^+$), 317 ($[M+H]^+$); HRMS calcd. for $C_{20}H_{28}O_3$ [M^+]: 316.2038; Found: 316.2040.

2.1.4 Synthesis of benzyl 2-(trimethylsilyl)-2,3-butadienyl carbonate **1d**. (zyc-4-123)

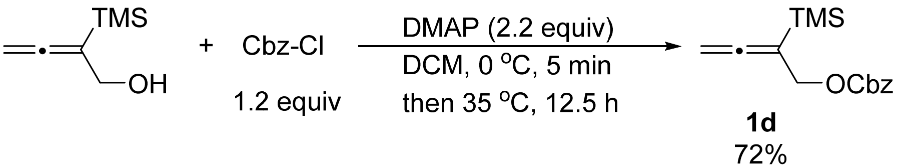

1d
72%

Following **Typical Procedure** Ⅰ, the reaction of DMAP (3.5607 g, 29.24 mmol), 2-(trimethylsilyl)-2,3-butadienol (1.8909 g, 13.29 mmol)/DCM (13 mL), and Cbz-Cl (2.7188 g, 15.95 mmol) afforded **1d** (2.6424 g, 72%) (eluent: petroleum ether (60-90 °C)/ethyl acetate = 100/1) as a liquid: ^{1}H NMR (300 MHz, $CDCl_3$) δ 7.44-7.27 (m, 5 H, ArH), 5.16 (s, 2 H, OCH_2Ph), 4.70 (t, J = 2.9 Hz, 2 H, $=CH_2$), 4.49 (t, J = 2.9 Hz, 2 H, OCH_2), 0.13 (s, 9 H, $CH_3 \times 3$); ^{13}C NMR (75 MHz, $CDCl_3$) δ 209.4, 154.9, 135.3, 128.5, 128.4, 128.2, 91.8, 70.6, 69.5, 66.8, -1.6; IR (neat) ν (cm^{-1}) 3067, 3035, 2957, 2897, 1934, 1748, 1498, 1456, 1387, 1362, 1251, 1114; MS (ESI): *m/z* 299 ([M+Na]$^+$), 294 ([M+NH_4]$^+$), 277 ([M+H]$^+$); HRMS calcd. for $C_{15}H_{20}O_3Si$ [M$^+$]: 276.1182; Found: 276.1183.

2.2 Pyrazol-5-one

2.2.1 Synthesis of 4-(2-((*tert*-butyldimethylsilyl)oxy)ethyl)-3-methyl-1-phenyl-1,4-dihydropyrazol-5-one **2p**. (zyc-4-143)

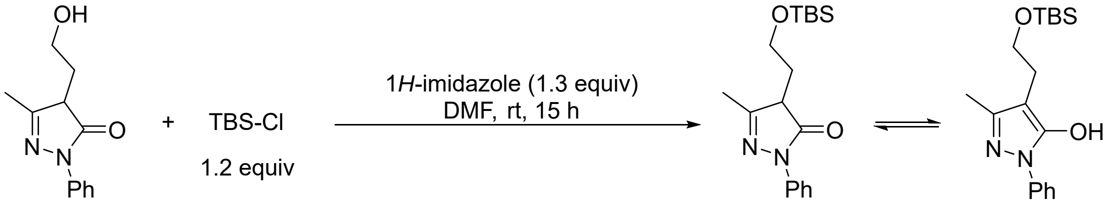

2o **2p-a** **2p-b**
2p
70%

To a flask were added **2o** (1.4320 g, 6.56 mmol)/DMF (13 mL), 1*H*-imidazole

(0.5812 g, 8.53 mmol), and TBS-Cl (1.1895 g, 7.87 mmol). The reaction was complete after being stirred at room temperature for 15 hours as monitored by TLC. Then water (50 mL) was added to quench the reaction. The resulting mixture was transferred to a separatory funnel and extracted with DCM (30 mL × 2). The combined organic phase was washed with sequentially water (50 mL × 3) and brine (20 mL) and dried over anhydrous Na_2SO_4. After filtration and evaporation of the solvent, the crude residual was purified by chromatography on silica gel (eluent: petroleum ether (60-90 °C)/ethyl acetate = 5/1) to afford **2p** (1536.3 g, 70%) as a solid: m.p. 118.6-120.3 °C (petroleum ether/ethyl acetate); ^{1}H NMR (300 MHz, $CDCl_3$) δ 7.46-7.26 (m, 2 H, ArH), 7.17 (t, *J* = 7.2 Hz, 1 H, ArH), 4.02-3.67 (m, 2 H, OCH_2), the following signals were discernible for isomer **2p-a**: 7.91 (d, *J* = 7.5 Hz, 0.53 H, ArH), 3.38 (t, *J* = 5.0 Hz, 0.25 H, CH), 2.19 (s, 1 H, CH_3), 0.86 (s, 2.84 H, CH_3 × 3), 0.01 (d, *J* = 3.6 Hz, 1.85 H, CH_3 × 2); the following signals were discernible for isomer **2p-b**: 10.01 (s, 0.64 H, OH), 7.69 (d, *J* = 6.3 Hz, 1.47 H, ArH), 2.55 (s, 1.45 H, CH_2), 2.12 (s, 2.67 H, CH_2 of isomer **2p-a** and CH_3), 0.94 (s, 6.54 H, CH_3 × 3), 0.13 (s, 4.44 H, CH_3 × 2); ^{13}C NMR (75 MHz, $CDCl_3$) δ 173.1, 160.5, 147.6, 138.4, 138.2, 128.7, 125.1, 124.7, 120.8, 118.6, 99.4, 64.4, 59.5, 49.6, 30.2, 26.2, 25.8, 18.3, 18.2, 15.8, 12.1, -5.5, -5.7; IR (neat) ν (cm^{-1}) 3061, 2949, 2928, 2857, 1710, 1617, 1589, 1567, 1500, 1471, 1459, 1401, 1364, 1309, 1253, 1088, 1040, 1001; MS (EI): *m/z* (%) 332 ([M]$^+$, 4.33), 275 (100); Anal. Calcd. for $C_{18}H_{28}N_2O_2Si$ (%): C 65.02, H 8.49, N 8.42; Found: C 65.21, H 8.26, N 8.30.

2.2.2 Synthesis of 4-benzyl-1-cyclohexyl-3-methyl-1,4-dihydropyrazol-5-one **2u**. (zyc-6-124, zyc-6-129)[7]

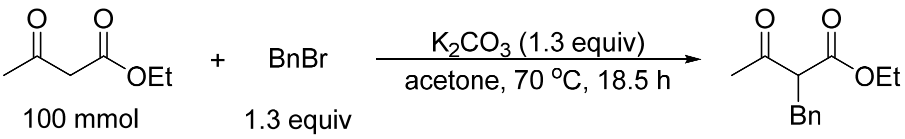

To a flask were added ethyl acetoacetate (12.7 mL, d = 1.0282 g/mL, 13.0581 g, 100 mmol), K_2CO_3 (17.9775 g, 130 mmol), and acetone (100 mL). The resulting mixture was stirred at room temperature for 5 minutes followed by the addition of BnBr (15.4 mL, d = 1.44 g/mL, 22.176 g, 130 mmol) within 5 minutes. The reaction was complete after being stirred at 70 °C for 18.5 hours as monitored by TLC (eluent: petroleum ether/ethyl acetate = 20/1). The resulting mixture was filtrated through a short column of silica gel and eluted with acetone (50 mL × 3). After evaporation, the residue (25.7764 g) was directly used for synthesis of **2u** and **2v**.

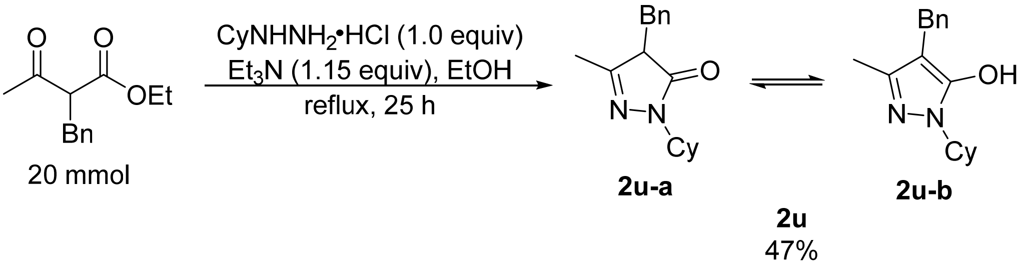

To a Schlenk tube were added ethyl 2-benzyl-3-oxobutanoate prepared above (5.1595 g, 20 mmol), $CyNHNH_2$•HCl (3.0126 g, 20 mmol), EtOH (20 mL), and Et_3N (3.2 mL, d = 0.728 g/mL, 2.3296 g, 23 mmol). The reaction was complete after being refluxed for 25 hours as monitored by TLC (eluent: petroleum ether/ethyl acetate = 2/1). After evaporation of EtOH, the residue was dissolved with DCM (100 mL) and transferred to a separatory funnel. The organic phase was washed with a saturated aqueous solution of $NaHCO_3$, water, and brine sequentially and dried over anhydrous

Na_2SO_4. After filtration and evaporation of DCM, the residue was purified by chromatography on silica gel (eluent: petroleum ether (60-90 °C)/ethyl acetate = 2/1 (900 mL) to ethyl acetate (1300 mL)) to afford **2u** (2.5631 g, 47%) as a solid: m.p. 126.3-126.8 °C (diethyl ether); ^{1}H NMR (300 MHz, $CDCl_3$) δ 7.30-6.98 (m, 5 H, ArH), 1.81-1.48 (m, 6.63 H, CH_2 of Cy), 1.43-0.82 (m, 3.48 H, CH_2 of Cy), the following signals were discernible for isomer **2u-a**: 3.99-3.83 (m, 0.17 H, CH of Cy), 3.29 (t, J = 5.6 Hz, 0.17 H, CH), 3.17 (d, J = 5.4 Hz, 0.33 H, CH_2), 1.98 (s, 0.52 H, CH_3); the following signals were discernible for isomer **2u-b**: 10.99 (brs, 0.28 H, OH), 4.27-4.04 (m, 0.81 H, CH of Cy), 3.54 (s, 1.58 H, CH_2), 1.86 (s, 2.42 H, CH_3); ^{13}C NMR (75 MHz, $CDCl_3$) δ 173.3, 161.9, 158.1, 144.8, 140.9, 136.1, 128.7, 128.4, 128.1, 128.0, 127.0, 125.6, 102.8, 53.4, 52.8, 52.2, 33.2, 31.3, 30.7, 30.5, 27.8, 25.6, 25.4, 25.3, 25.1, 16.1, 10.9; IR (neat) ν (cm^{-1}) 3062, 3027, 2934, 2856, 1593, 1550; MS (EI): *m/z* (%) 270 ([M]$^+$, 100); Anal. Calcd. for $C_{17}H_{22}N_2O$ (%): C 75.52, H 8.20, N 10.36; Found: C 75.27, H 8.23, N 10.22.

2.2.3 Synthesis of 1,4-dibenzyl-3-methyl-1,4-dihydropyrazol-5-one **2v**. (zyc-6-131)[7]

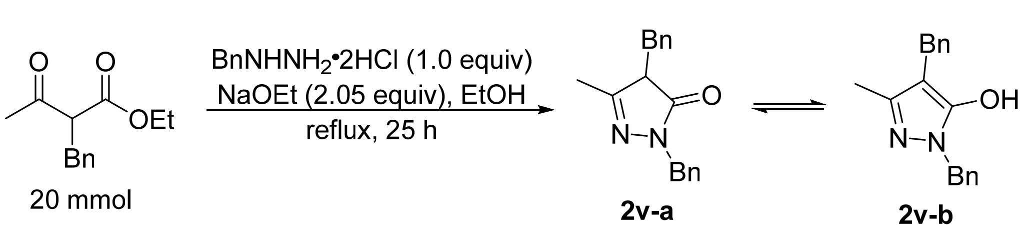

2v-a **2v-b**

2v
39%

To a Schlenk tube were added ethyl 2-benzyl-3-oxobutanoate prepared in 2.2.2 (5.1527 g, 20 mmol), $BnNHNH_2$•2HCl (3.9023 g, 20 mmol), EtOH (20 mL), and NaOEt (2.7901 g, 41 mmol). The reaction was complete after being refluxed for 25

hours as monitored by TLC (eluent: petroleum ether/ethyl acetate = 1/1). After evaporation of EtOH, the residue was dissolved with DCM (100 mL) and transferred to a separatory funnel. The organic phase was washed with saturated aqueous $NaHCO_3$, water, and brine sequentially and dried over anhydrous Na_2SO_4. After filtration and evaporation of DCM, the residue was purified by chromatography on silica gel (eluent: petroleum ether (60-90 °C)/ethyl acetate = 2/1 (600 mL) to petroleum ether (60-90 °C)/ethyl acetate/DCM = 1/1/1 (600 mL) to ethyl acetate (1500 mL)) to afford **2v**[10] (2.1739 g, 39%) as a solid: m.p. 151.1-151.6 °C (diethyl ether); ^{1}H NMR (300 MHz, $CDCl_3$) δ 7.47-6.68 (m, 10 H, ArH), 4.89-4.49 (m, 2 H, NCH_2), the following signals were discernible for isomer **2v-a**: 3.30 (t, J = 5.3 Hz, 0.11 H, CH), 3.18 (d, J = 5.4 Hz, 0.19 H, CH_2), 1.97 (s, 0.26 H, CH_3); the following signals were discernible for isomer **2v-b**: 10.86 (brs, 0.80 H, OH), 3.42 (s, 1.71 H, CH_2), 1.66 (s, 2.60 H, CH_3); ^{13}C NMR (75 MHz, $CDCl_3$) δ 173.9, 161.6, 158.9, 144.5, 140.8, 136.6, 136.14, 136.09, 128.8, 128.6, 128.4, 128.3, 128.1, 128.0, 127.7, 127.3, 127.0, 125.6, 102.0, 52.2, 47.4, 47.1, 33.0, 27.7, 16.1, 10.7; IR (neat) ν (cm^{-1}) 3062, 3028, 2920, 2691, 1582, 1495, 1453; MS (EI): *m/z* (%) 278 ($[M]^+$, 66.93), 91 (100).

3. Synthesis of 4-benzyl-3-methyl-1-phenyl-4-(2-butyl-2,3-butadienyl)pyrazol-5-one **3aa** and 4-benzyl-3-methyl-1-phenyl-2-(2-butyl-2,3-butadienyl)pyrazol-5-one **4aa**. (zyc-4-35)

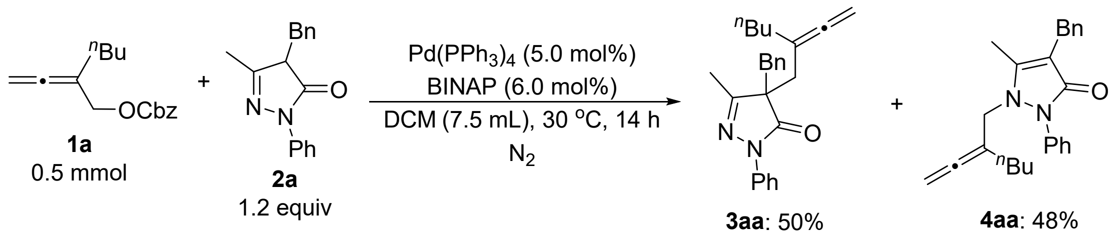

To a flame-dried Schlenk flask were added $Pd(PPh_3)_4$ (29.0 mg, 0.025 mmol), BINAP (18.7 mg, 0.030 mmol), **1a** (131.1 mg, 0.5 mmol)/DCM (5 mL), and **2a** (158.7 mg, 0.6 mmol)/DCM (2.5 mL) sequentially under nitrogen atmosphere at room temperature. The flask was put into a pre-heat oil bath and the reaction was complete after being stirred at 30 °C for 14 hours as monitored by TLC (eluent: petroleum ether/ethyl acetate = 20/1). The resulting mixture was filtrated through a short column of silica gel eluted with ethyl acetate (15 mL × 3). After evaporation, the residue was purified by chromatography on silica gel (eluent: petroleum ether (60-90 °C)/ethyl acetate = 40/1 (400 mL) to 4/1 (500 mL) to 2/1 (300 mL)) to afford **3aa** (92.3 mg, 50 %, purity = 94%) and impure **4aa** (105.4 mg), which was further purified by chromatography on silica gel (eluent: DCM/MeOH = 200/1 (500 mL) to 100/1 (200 mL)). Two-round chromatography afforded pure **4aa** (88.6 mg, 48%).

3aa: liquid; ^{1}H NMR (300 MHz, $CDCl_3$) δ 7.48 (d, J = 8.1 Hz, 2 H, ArH), 7.21 (t, J = 7.8 Hz, 2 H, ArH), 7.12-6.90 (m, 6 H, ArH), 4.56-4.38 (m, 2 H, =CH_2), 3.09 (d, J = 13.2 Hz, 1 H, one proton of CH_2), 2.79 (d, J = 13.2 Hz, 1 H, one proton of CH_2), 2.60 (d, J = 14.7 Hz, 1 H, one proton of CH_2), 2.30 (d, J = 15.3 Hz, 1 H, one proton of CH_2), 2.05 (s, 3 H, CH_3), 1.85-1.68 (m, 2 H, CH_2), 1.36-1.07 (m, 4 H, CH_2 × 2), 0.75 (t, J = 6.9 Hz, 3 H, CH_3); ^{13}C NMR (75 MHz, $CDCl_3$) δ 205.3, 174.5, 161.3, 137.6, 134.1, 129.1, 128.5, 128.1, 127.2, 124.8, 119.2, 98.3, 77.6, 60.1, 42.8, 36.1, 32.3, 29.4,

22.1, 14.7, 13.8; IR (neat) ν (cm^{-1}) 3063, 3031, 2956, 2927, 2871, 2856, 1955, 1709, 1597, 1500, 1456, 1440, 1402, 1366, 1123, 1082, 1031; MS (EI): *m/z* (%) 372 ($[M]^+$, 28.48), 186 (100); HRMS calcd. for $C_{25}H_{28}N_2O$ $[M]^+$: 372.2202; Found: 372.2204.

4aa: liquid; ^1H NMR (300 MHz, $CDCl_3$) δ 7.54-7.08 (m, 10 H, ArH), 4.59-4.40 (m, 2 H, $=CH_2$), 4.05 (s, 2 H, CH_2), 3.65 (s, 2 H, CH_2), 2.17 (s, 3 H, CH_3), 1.69-1.52 (m, 2 H, CH_2), 1.23-1.07 (m, 4 H, $CH_2 \times 2$), 0.78 (t, $J = 6.6$ Hz, 3 H, CH_3); ^{13}C NMR (75 MHz, $CDCl_3$) δ 206.2, 166.0, 151.8, 140.2, 135.1, 128.8, 128.3, 128.2, 126.1, 125.7, 123.9, 110.0, 98.5, 77.3, 48.8, 29.4, 29.1, 28.2, 22.0, 13.7, 11.4; IR (neat) ν (cm^{-1}) 3061, 3027, 2956, 2928, 2873, 2858, 1954, 1667, 1594, 1495, 1455, 1434, 1355, 1336, 1308, 1127, 1029; MS (EI): *m/z* (%) 372 ($[M]^+$, 12.89), 91 (100); HRMS calcd. for $C_{25}H_{28}N_2O$ $[M]^+$: 372.2202; Found: 372.2205.

4. Pd-catalyzed enantioselective allenylation of pyrazol-5-ones: synthesis of chiral products.

4.1 Synthesis of (*S*)-4-benzyl-3-methyl-1-phenyl-4-(2-butyl-2,3-butadienyl)pyrazol-5-one (*S*)-**3aa**. (zyc-4-30)

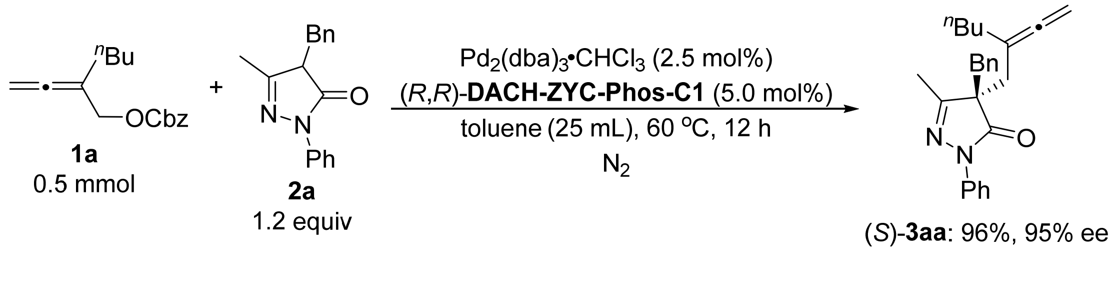

Typical Procedure II: To a flame-dried Schlenk flask were added $Pd_2(dba)_3$•$CHCl_3$ (12.9 mg, 0.0125 mmol), (*R*,*R*)-**DACH-ZYC-Phos-C1** (14.3 mg,

0.025 mmol), toluene (5 mL), **1a** (131.5 mg, 0.5 mmol)/toluene (11.7 mL), and **2a** (158.7 mg, 0.6 mmol)/toluene (8.3 mL) sequentially under nitrogen atmosphere at room temperature. The flask was put into a pre-heat oil bath and the reaction was complete after being stirred at 60 °C for 12 hours as monitored by TLC (eluent: petroleum ether/ethyl acetate = 20/1). The resulting mixture was filtrated through a short column of silica gel and eluted with ethyl acetate (15 mL × 3). After evaporation, the residue was purified by chromatography on silica gel (eluent: petroleum ether (60-90 °C)/ethyl acetate = 60/1) to afford a pure part of (*S*)-**3aa** and the impure part was further purified by chromatography on silica gel (eluent: petroleum ether (60-90 °C)/ethyl acetate = 40/1). Two-round chromatography afforded (*S*)-**3aa** (179.5 mg, 96%) as a liquid: 95% ee (HPLC condition: Chiralcel IA column, *n*-hexane/*i*-PrOH = 90/10, 1.0 mL/min, λ = 254 nm, t_R (major) = 5.7 min, t_R (minor) = 8.9 min); $[\alpha]_D^{20}$ = -8.2 (c = 1.25, $CHCl_3$); ^1H NMR (300 MHz, $CDCl_3$) δ 7.55 (d, *J* = 8.1 Hz, 2 H, ArH), 7.30 (t, *J* = 7.8 Hz, 2 H, ArH), 7.22-7.02 (m, 6 H, ArH), 4.64-4.49 (m, 2 H, $=CH_2$), 3.18 (d, *J* = 13.2 Hz, 1 H, one proton of CH_2), 2.89 (d, *J* = 13.5 Hz, 1 H, one proton of CH_2), 2.69 (dt, J_1 = 15.3 Hz, J_2 = 3.3 Hz, 1 H, one proton of CH_2), 2.39 (d, *J* = 15.0 Hz, 1 H, one proton of CH_2), 2.14 (s, 3 H, CH_3), 1.93-1.80 (m, 2 H, CH_2), 1.43-1.17 (m, 4 H, CH_2 × 2), 0.83 (t, *J* = 7.1 Hz, 3 H, CH_3); ^{13}C NMR (75 MHz, $CDCl_3$) δ 205.3, 174.6, 161.4, 137.6, 134.1, 129.1, 128.5, 128.1, 127.2, 124.8, 119.3, 98.3, 77.7, 60.1, 42.8, 36.1, 32.3, 29.4, 22.1, 14.7, 13.8; IR (neat) ν (cm^{-1}) 3063, 3031, 2956, 2927, 2859, 1954, 1712, 1597, 1500, 1455, 1440, 1402, 1366, 1123; MS (EI): *m/z* (%) 372 ($[M]^+$, 31.27), 186 (100); HRMS calcd. for $C_{25}H_{28}N_2O$ $[M]^+$: 372.2202; Found:

372.2202.

4.2 Synthesis of (*S*)-4-benzyl-3-methyl-1-phenyl-4-(2-methyl-2,3-butadienyl)pyrazol-5-one (*S*)-**3ba**. (zyc-4-34)

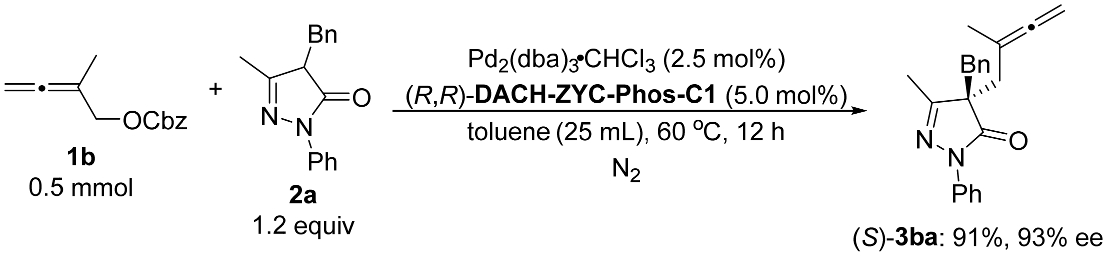

Following **Typical Procedure II**, the reaction of $Pd_2(dba)_3$•$CHCl_3$ (13.0 mg, 0.0125 mmol), (*R*,*R*)-**DACH-ZYC-Phos-C1** (14.2 mg, 0.025 mmol), toluene (5 mL), **1b** (109.2 mg, 0.5 mmol)/toluene (11.7 mL), and **2a** (158.6 mg, 0.6 mmol)/toluene (8.3 mL) afforded (*S*)-**3ba** (150.3 mg, 91%) (the residue was purified by chromatography on silica gel (eluent: petroleum ether (60-90 °C)/ethyl acetate = 40/1) to afford a pure part of (*S*)-**3ba** and the impure part was further purified by chromatography on silica gel (eluent: petroleum ether (60-90 °C)/ethyl acetate = 40/1)) as a liquid: 93% ee (HPLC condition: Chiralcel IA column, *n*-hexane/*i*-PrOH = 90/10, 1.0 mL/min, λ = 254 nm, t_R (major) = 5.8 min, t_R (minor) = 9.6 min); $[\alpha]_D^{20}$ = +0.4 (c = 0.905, $CHCl_3$); ^1H NMR (300 MHz, $CDCl_3$) δ 7.50 (d, *J* = 7.8 Hz, 2 H, ArH), 7.18 (t, *J* = 7.8 Hz, 2 H, ArH), 7.10-6.90 (m, 6 H, ArH), 4.40 (q, *J* = 2.1 Hz, 2 H, =CH_2), 3.06 (d, *J* = 13.2 Hz, 1 H, one proton of CH_2), 2.76 (d, *J* = 13.2 Hz, 1 H, one proton of CH_2), 2.56 (d, *J* = 14.7 Hz, 1 H, one proton of CH_2), 2.30 (d, *J* = 15.0 Hz, 1 H, one proton of CH_2), 2.02 (s, 3 H, CH_3), 1.51 (t, *J* = 2.4 Hz, 3 H, CH_3); ^{13}C NMR (75 MHz, $CDCl_3$) δ 206.0, 174.4, 161.3, 137.5, 134.0, 128.9, 128.4, 128.0, 127.1, 124.7, 119.1,

93.4, 75.7, 60.0, 42.5, 37.4, 19.4, 14.5; IR (neat) ν (cm^{-1}) 3063, 3031, 2981, 2918, 1957, 1713, 1596, 1501, 1455, 1441, 1402, 1367, 1324, 1123, 1030; MS (EI): *m/z* (%) 330 ($[M]^+$, 44.50), 77 (100); HRMS calcd. for $C_{22}H_{22}N_2O$ $[M]^+$: 330.1732; Found: 330.1732.

4.3 Synthesis of (*S*)-4-benzyl-3-methyl-1-phenyl-4-(2-octyl-2,3-butadienyl)pyrazol-5-one (*S*)-**3ca**. (zyc-4-45)

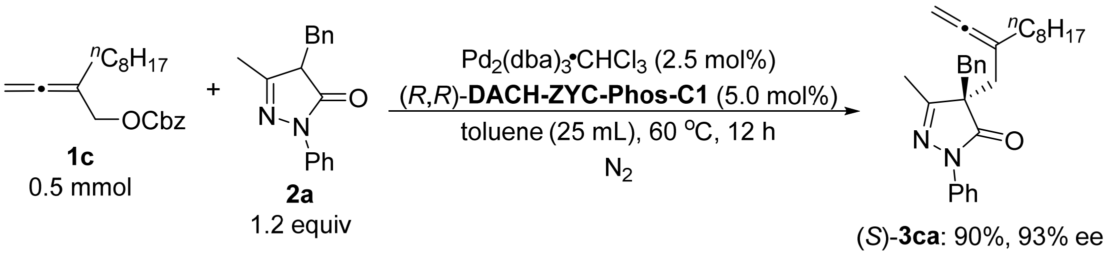

Following **Typical Procedure II**, the reaction of $Pd_2(dba)_3$•$CHCl_3$ (13.0 mg, 0.0125 mmol), (*R*,*R*)-**DACH-ZYC-Phos-C1** (14.4 mg, 0.025 mmol), toluene (5 mL), **1c** (158.1 mg, 0.5 mmol)/toluene (11.7 mL), and **2a** (158.8 mg, 0.6 mmol)/toluene (8.3 mL) afforded (*S*)-**3ca** (192.8 mg, 90%) (eluent: petroleum ether (60-90 °C)/ethyl acetate = 40/1) as a liquid: 93% ee (HPLC condition: Chiralcel IA column, *n*-hexane/*i*-PrOH = 90/10, 1.0 mL/min, λ = 254 nm, t_R (major) = 5.0 min, t_R (minor) = 7.8 min); $[\alpha]_D^{20}$ = -7.2 (c = 0.94, $CHCl_3$); ^1H NMR (300 MHz, $CDCl_3$) δ 7.57 (d, J = 8.0 Hz, 2 H, ArH), 7.29 (t, J = 7.8 Hz, 2 H, ArH), 7.20-7.03 (m, 6 H, ArH), 4.64-4.49 (m, 2 H, $=CH_2$), 3.17 (d, J = 13.2 Hz, 1 H, one proton of CH_2), 2.87 (d, J = 13.2 Hz, 1 H, one proton of CH_2), 2.68 (dt, J_1 = 15.0 Hz, J_2 = 3.3 Hz, 1 H, one proton of CH_2), 2.38 (d, J = 15.3 Hz, 1 H, one proton of CH_2), 2.13 (s, 3 H, CH_3), 1.94-1.80 (m, 2 H, CH_2), 1.48-1.15 (m, 12 H, $CH_2 \times 6$), 0.86 (t, J = 6.8 Hz, 3 H, CH_3); ^{13}C NMR (75

MHz, $CDCl_3$) δ 205.4, 174.5, 161.3, 137.6, 134.1, 129.0, 128.5, 128.1, 127.2, 124.7, 119.1, 98.3, 77.5, 60.1, 42.7, 36.1, 32.5, 31.7, 29.2, 29.1, 29.0, 27.2, 22.5, 14.6, 14.0; IR (neat) ν (cm^{-1}) 3058, 3032, 2925, 2854, 1955, 1709, 1597, 1500, 1456, 1441, 1402, 1365, 1123; MS (EI): *m/z* (%) 428 ($[M]^+$, 29.49), 186 (100); HRMS calcd. for $C_{29}H_{36}N_2O$ $[M]^+$: 428.2828; Found: 428.2829.

4.4 Synthesis of (*S*)-4-benzyl-3-ethyl-1-phenyl-4-(2-(trimethylsilyl)-2,3-butadienyl)-pyrazol-5-one (*S*)-**3da**. (zyc-4-139)

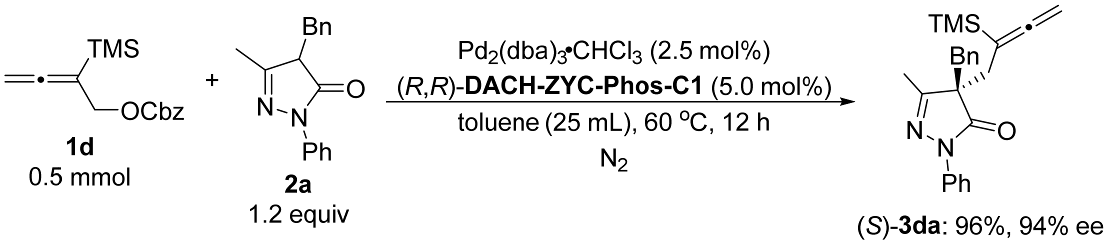

Following **Typical Procedure II**, the reaction of $Pd_2(dba)_3$•$CHCl_3$ (12.9 mg, 0.0125 mmol), (*R*,*R*)-**DACH-ZYC-Phos-C1** (14.4 mg, 0.025 mmol), toluene (5 mL), **1d** (137.9 mg, 0.5 mmol)/toluene (11.7 mL), and **2a** (158.7 mg, 0.6 mmol)/toluene (8.3 mL) afforded (*S*)-**3da** (185.9 mg, 96%) (eluent: petroleum ether (60-90 °C)/DCM = 2/1) as a liquid: 94% ee (HPLC condition: Chiralcel IA column, *n*-hexane/*i*-PrOH = 90/10, 1.0 mL/min, λ = 254 nm, t_R (major) = 4.9 min, t_R (minor) = 6.8 min); $[\alpha]_D^{20}$ = -46.3 (c = 0.99, $CHCl_3$); ^1H NMR (300 MHz, $CDCl_3$) δ 7.55 (d, *J* = 8.1 Hz, 2 H, ArH), 7.27 (t, *J* = 8.0 Hz, 2 H, ArH), 7.18-7.01 (m, 6 H, ArH), 4.28-4.07 (m, 2 H, =CH_2), 3.15 (d, *J* = 13.2 Hz, 1 H, one proton of CH_2), 2.88 (d, *J* = 13.2 Hz, 1 H, one proton of CH_2), 2.67 (dt, J_1 = 15.9 Hz, J_2 = 4.2 Hz, 1 H, one proton of CH_2), 2.29 (dt, J_1 = 15.9 Hz, J_2 = 3.0 Hz, 1 H, one proton of CH_2), 2.13 (s, 3 H, CH_3), 0.09 (s, 9 H, CH_3 × 3);

^{13}C NMR (75 MHz, $CDCl_3$) δ 207.3, 174.4, 160.8, 137.7, 133.9, 129.0, 128.4, 128.1, 127.2, 124.6, 119.2, 89.5, 71.8, 60.6, 43.1, 31.0, 14.5, -2.0; IR (neat) ν (cm^{-1}) 3063, 3032, 2955, 2917, 2890, 1925, 1709, 1597, 1500, 1456, 1442, 1404, 1366, 1312, 1250, 1128, 1082, 1030, 1002; MS (EI): *m/z* (%) 388 ($[M]^+$, 100); HRMS calcd. for $C_{24}H_{28}N_2OSi$ $[M]^+$: 388.1971; Found: 388.1968.

4.5 Synthesis of (*S*)-4-benzyl-3-methyl-1-phenyl-4-(2-phenyl-2,3-butadienyl)pyrazol-5-one (*S*)-**3ea**. (zyc-5-120)

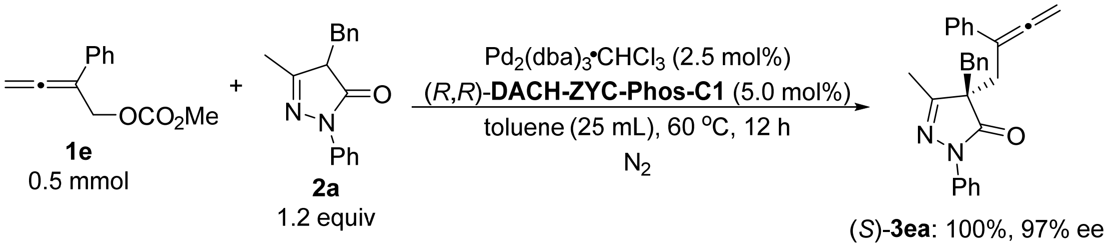

Following **Typical Procedure II**, the reaction of $Pd_2(dba)_3$•$CHCl_3$ (12.9 mg, 0.0125 mmol), (*R*,*R*)-**DACH-ZYC-Phos-C1** (14.3 mg, 0.025 mmol), toluene (5 mL), **1e** (109.8 mg, 0.5 mmol)/toluene (11.7 mL), and **2a** (158.8 mg, 0.6 mmol)/toluene (8.3 mL) afforded (*S*)-**3ea** (197.4 mg, 100%) (eluent: petroleum ether (60-90 °C)/ethyl acetate = 50/1)) as a liquid: 97% ee (HPLC condition: Chiralcel IA column, *n*-hexane/*i*-PrOH = 90/10, 1.0 mL/min, λ = 254 nm, t_R (minor) = 17.0 min, t_R (major) = 18.2 min); $[\alpha]_D^{20}$ = -30.6 (c = 1.025, $CHCl_3$); ^{1}H NMR (300 MHz, $CDCl_3$) δ 7.61-6.90 (m, 15 H, ArH), 5.05-4.77 (m, 2 H, =CH_2), 3.27 (d, *J* = 13.2 Hz, 1 H, one proton of CH_2), 3.24 (dt, J_1 = 15.3 Hz, J_2 = 3.9 Hz, 1 H, one proton of CH_2), 2.97 (d, *J* = 13.5 Hz, 1 H, one proton of CH_2), 2.80 (dt, J_1 = 15.3 Hz, J_2 = 2.4 Hz, 1 H, one proton of CH_2), 2.08 (s, 3 H, CH_3); ^{13}C NMR (75 MHz, $CDCl_3$) δ 207.6, 174.2, 161.0,

137.5, 135.7, 134.0, 129.2, 128.5, 128.3, 128.2, 127.3, 127.1, 126.1, 124.8, 119.2, 100.4, 80.1, 60.1, 42.9, 33.6, 14.7; IR (neat) ν (cm^{-1}) 3061, 3031, 2917, 1942, 1708, 1596, 1500, 1454, 1403, 1366, 1339, 1325, 1246, 1128, 1082, 1030; MS (EI): *m/z* (%) 392 ($[M]^+$, 100); HRMS calcd. for $C_{27}H_{24}N_2O$ $[M]^+$: 392.1889; Found: 392.1887.

4.6 Synthesis of (*R*)-4-benzyl-4-(2,3-butadienyl)-3-methyl-1-phenylpyrazol-5-one (*R*)-**3fa**. (zyc-6-154, zyc-4-171)

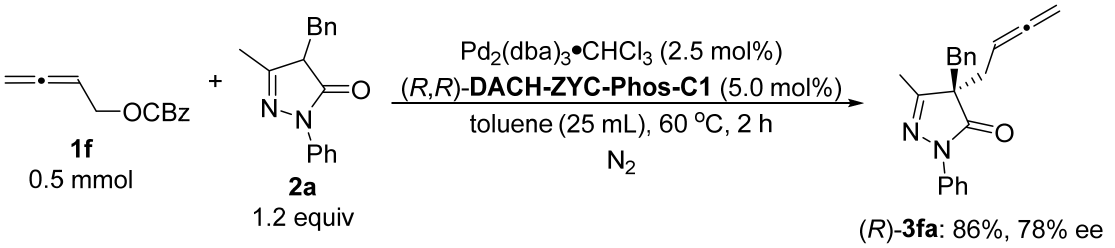

Following **Typical Procedure II**, the reaction of $Pd_2(dba)_3$•$CHCl_3$ (12.8 mg, 0.0125 mmol), (*R*,*R*)-**DACH-ZYC-Phos-C1** (14.2 mg, 0.025 mmol), toluene (5 mL), **1f** (102.4 mg, 0.5 mmol)/toluene (11.7 mL), and **2a** (158.6 mg, 0.6 mmol)/toluene (8.3 mL) afforded (*R*)-**3fa** (136.3 mg, 86%) (eluent: petroleum ether (60-90 °C)/ethyl acetate = 30/1) as a liquid: 78% ee (HPLC condition: Chiralcel IA column, *n*-hexane/*i*-PrOH = 90/10, 1.0 mL/min, λ = 254 nm, t_R (major) = 8.5 min, t_R (minor) = 9.8 min); $[\alpha]_D^{20}$ = -13.7 (c = 1.01, $CHCl_3$); ^1H NMR (300 MHz, $CDCl_3$) δ 7.65 (d, *J* = 8.1 Hz, 2 H, ArH), 7.31 (t, *J* = 7.8 Hz, 2 H, ArH), 7.22-7.01 (m, 6 H, ArH), 4.96-4.77 (m, 1 H, =CH), 4.75-4.57 (m, 2 H, =CH_2), 3.22 (d, *J* = 13.5 Hz, 1 H, one proton of CH_2), 2.89 (d, *J* = 13.5 Hz, 1 H, one proton of CH_2), 2.77-2.62 (m, 1 H, one proton of CH_2), 2.43 (dd, J_1 = 13.8 Hz, J_2 = 8.7 Hz, 1 H, one proton of CH_2), 2.12 (s, 3 H, CH_3); ^{13}C NMR (75 MHz, $CDCl_3$) δ 209.4, 174.3, 161.3, 137.5, 134.5, 128.8, 128.6, 128.3,

127.2, 125.0, 119.2, 83.3, 75.7, 60.7, 41.0, 33.7, 14.5; IR (neat) ν (cm^{-1}) 3063, 3031, 2981, 2917, 2843, 1955, 1706, 1596, 1500, 1456, 1439, 1402, 1366, 1341, 1327, 1305, 1236, 1184, 1123, 1083, 1030; MS (EI): *m/z* (%) 316 ($[M]^+$, 99), 91 (100); HRMS calcd. for $C_{21}H_{20}N_2O$ $[M]^+$: 316.1576; Found: 316.1574.

4.7 Synthesis of (*R*)-3-methyl-4-(2-methylbenzyl)-1-phenyl-4-(2-butyl-2,3-butadienyl)pyrazol-5-one (*R*)-**3ab**. (zyc-4-50)

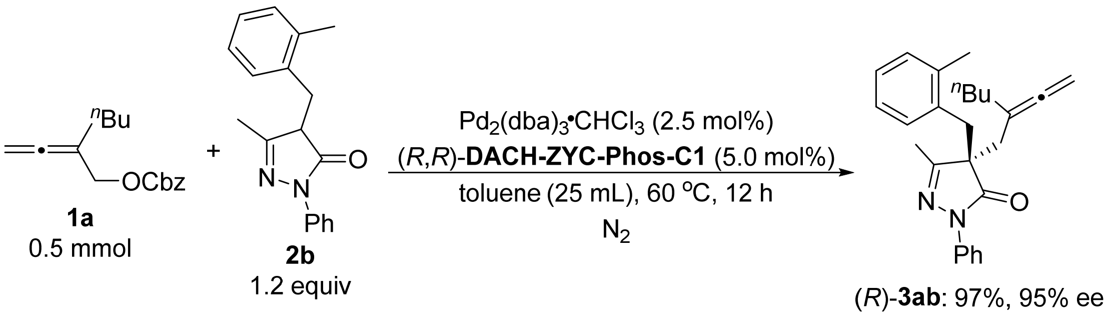

Following **Typical Procedure II**, the reaction of $Pd_2(dba)_3$•$CHCl_3$ (13.0 mg, 0.0125 mmol), (*R*,*R*)-**DACH-ZYC-Phos-C1** (14.2 mg, 0.025 mmol), toluene (5 mL), **1a** (131.2 mg, 0.5 mmol)/toluene (11.7 mL), and **2b** (167.2 mg, 0.6 mmol)/toluene (8.3 mL) afforded (*R*)-**3ab** (187.0 mg, 97%) (eluent: petroleum ether (60-90 °C)/ethyl acetate = 50/1) as a liquid: 95% ee (HPLC condition: Chiralcel IA column, *n*-hexane/*i*-PrOH = 90/10, 1.0 mL/min, λ = 254 nm, t_R (major) = 5.3 min, t_R (minor) = 6.2 min); $[\alpha]_D^{20}$ = +19.4 (c = 0.94, $CHCl_3$); ^1H NMR (300 MHz, $CDCl_3$) δ 7.65 (d, *J* = 8.4 Hz, 2 H, ArH), 7.31 (t, *J* = 7.8 Hz, 2 H, ArH), 7.16-6.96 (m, 5 H, ArH), 4.55 (pentet, *J* = 2.9 Hz, 2 H, =CH_2), 3.20 (d, *J* = 14.1 Hz, 1 H, one proton of CH_2), 2.94 (d, *J* = 13.8 Hz, 1 H, one proton of CH_2), 2.71 (dt, J_1 = 15.0 Hz, J_2 = 3.2 Hz, 1 H, one proton of CH_2), 2.42 (d, *J* = 15.0 Hz, 1 H, one proton of CH_2), 2.33 (s, 3 H, CH_3),

2.06 (s, 3 H, CH_3), 1.94-1.80 (m, 2 H, CH_2), 1.41-1.16 (m, 4 H, CH_2 × 2), 0.82 (t, J = 7.1 Hz, 3 H, CH_3); ^{13}C NMR (75 MHz, $CDCl_3$) δ 205.3, 174.9, 162.0, 137.9, 136.6, 132.9, 130.5, 129.5, 128.6, 127.2, 125.6, 124.7, 119.1, 98.3, 77.7, 59.2, 38.5, 36.1, 32.4, 29.4, 22.1, 20.0, 14.9, 13.8; IR (neat) ν (cm^{-1}) 3063, 3023, 2956, 2927, 2871, 2856, 1954, 1714, 1597, 1504, 1456, 1401, 1362, 1324, 1295, 1244, 1179, 1124, 1032; MS (EI): *m/z* (%) 386 ($[M]^+$, 58.07), 369 (100); HRMS calcd. for $C_{26}H_{30}N_2O$ $[M]^+$: 386.2358; Found: 386.2355.

4.8 Synthesis of (*S*)-3-methyl-4-(3-methylbenzyl)-1-phenyl-4-(2-butyl-2,3-butadienyl)pyrazol-5-one (*S*)-**3ac**. (zyc-4-94)

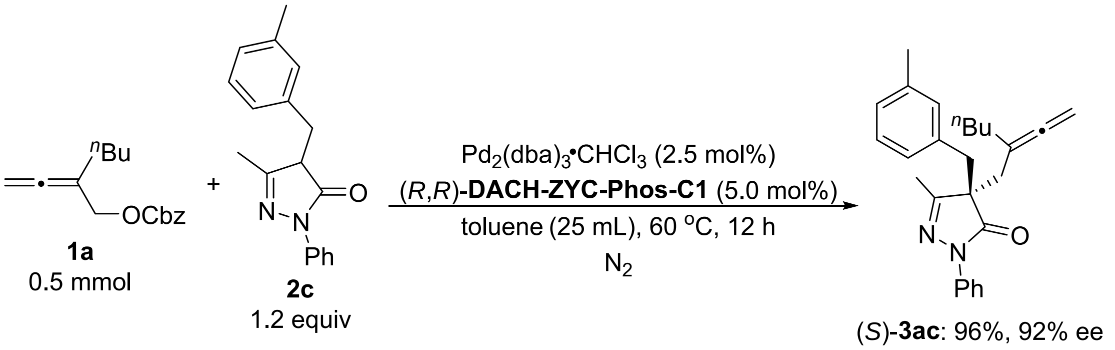

Following **Typical Procedure II**, the reaction of $Pd_2(dba)_3$•$CHCl_3$ (13.0 mg, 0.0125 mmol), (*R*,*R*)-**DACH-ZYC-Phos-C1** (14.3 mg, 0.025 mmol), toluene (5 mL), **1a** (131.8 mg, 0.5 mmol)/toluene (11.7 mL), and **2c** (167.2 mg, 0.6 mmol)/toluene (8.3 mL) afforded (*S*)-**3ac** (185.8 mg, 96%) (eluent: petroleum ether (60-90 °C)/diethyl ether = 50/1 (1000 mL) to 40/1 (800 mL)) as a liquid: 92% ee (HPLC condition: Chiralcel IA column, *n*-hexane/*i*-PrOH = 90/10, 1.0 mL/min, λ = 254 nm, t_R (major) = 5.4 min, t_R (minor) = 7.4 min); $[\alpha]_D^{20}$ = -10.4 (c = 1.08, $CHCl_3$); ^{1}H NMR (300 MHz, $CDCl_3$) δ 7.59 (d, J = 8.7 Hz, 2 H, ArH), 7.29 (t, J = 8.0 Hz, 2 H, ArH),

7.09 (t, J = 7.5 Hz, 1 H, ArH), 7.02 (t, J = 7.5 Hz, 1 H, ArH), 6.96-6.83 (m, 3 H, ArH), 4.56 (pentet, J = 3.1 Hz, 2 H, $=CH_2$), 3.14 (d, J = 13.2 Hz, 1 H, one proton of CH_2), 2.82 (d, J = 13.5 Hz, 1 H, one proton of CH_2), 2.67 (dt, J_1 = 15.3 Hz, J_2 = 3.3 Hz, 1 H, one proton of CH_2), 2.36 (dt, J_1 = 15.0 Hz, J_2 = 2.3 Hz, 1 H, one proton of CH_2), 2.14 (s, 3 H, CH_3), 2.10 (s, 3 H, CH_3), 1.92-1.80 (m, 2 H, CH_2), 1.42-1.17 (m, 4 H, $CH_2 \times 2$), 0.83 (t, J = 7.1 Hz, 3 H, CH_3); ^{13}C NMR (75 MHz, $CDCl_3$) δ 205.3, 174.6, 161.4, 137.61, 137.58, 134.0, 129.7, 128.4, 127.90, 127.88, 126.0, 124.7, 119.1, 98.3, 77.5, 60.0, 42.8, 35.9, 32.2, 29.3, 22.0, 21.0, 14.6, 13.7; IR (neat) ν (cm^{-1}) 3062, 3049, 3031, 2956, 2925, 2873, 2859, 1955, 1709, 1597, 1500, 1458, 1441, 1402, 1365, 1323, 1122; MS (EI): *m/z* (%) 386 ($[M]^+$, 41.96), 186 (100); HRMS calcd. for $C_{26}H_{30}N_2O$ $[M]^+$: 386.2358; Found: 386.2360.

4.9 Synthesis of (*S*)-3-methyl-4-(4-methylbenzyl)-1-phenyl-4-(2-butyl-2,3-butadienyl)pyrazol-5-one (*S*)-**3ad**. (zyc-4-75)

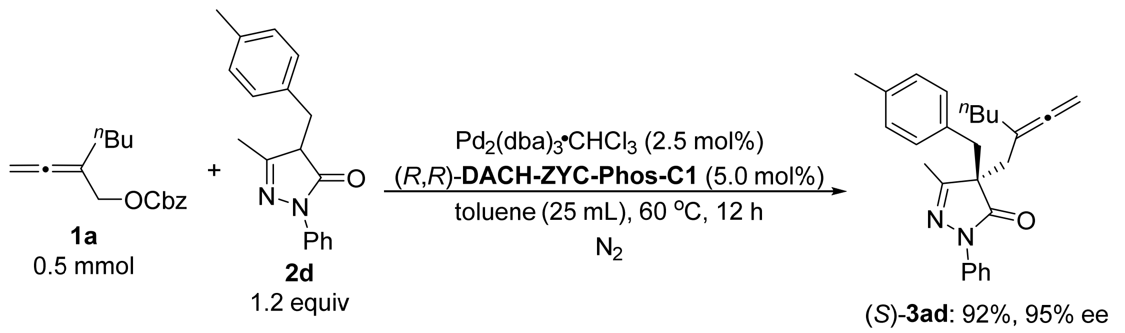

Following **Typical Procedure II**, the reaction of $Pd_2(dba)_3$•$CHCl_3$ (12.8 mg, 0.0125 mmol), (*R*,*R*)-**DACH-ZYC-Phos-C1** (14.3 mg, 0.025 mmol), toluene (5 mL), **1a** (131.1 mg, 0.5 mmol)/toluene (11.7 mL), and **2d** (167.0 mg, 0.6 mmol)/toluene (8.3 mL) afforded (*S*)-**3ad** (178.1 mg, 92%) (eluent: petroleum ether (60-90

°C)/diethyl ether = 60/1 (600 mL) to 40/1 (1000 mL)) as a liquid: 95% ee (HPLC condition: Chiralcel IA column, *n*-hexane/*i*-PrOH = 90/10, 1.0 mL/min, λ = 254 nm, t_R (major) = 5.7 min, t_R (minor) = 7.9 min); $[\alpha]_D^{20}$ = -24.9 (c = 1.125, $CHCl_3$); ^1H NMR (300 MHz, $CDCl_3$) δ 7.60 (d, *J* = 7.5 Hz, 2 H, ArH), 7.30 (t, *J* = 8.0 Hz, 2 H, ArH), 7.10 (t, *J* = 7.5 Hz, 1 H, ArH), 7.00-6.90 (m, 4 H, ArH), 4.55 (pentet, *J* = 3.0 Hz, 2 H, $=CH_2$), 3.14 (d, *J* = 13.2 Hz, 1 H, one proton of CH_2), 2.84 (d, *J* = 13.5 Hz, 1 H, one proton of CH_2), 2.67 (dt, J_1 = 15.3 Hz, J_2 = 3.3 Hz, 1 H, one proton of CH_2), 2.37 (dt, J_1 = 15.3 Hz, J_2 = 2.2 Hz, 1 H, one proton of CH_2), 2.19 (s, 3 H, CH_3), 2.12 (s, 3 H, CH_3), 1.92-1.80 (m, 2 H, CH_2), 1.40-1.17 (m, 4 H, $CH_2 \times 2$), 0.82 (t, *J* = 7.1 Hz, 3 H, CH_3); ^{13}C NMR (75 MHz, $CDCl_3$) δ 205.3, 174.7, 161.5, 137.7, 136.7, 131.0, 128.9, 128.8, 128.5, 124.7, 119.2, 98.3, 77.5, 60.1, 42.4, 36.1, 32.3, 29.4, 22.1, 20.9, 14.7, 13.7; IR (neat) ν (cm^{-1}) 3049, 3028, 2956, 2925, 2869, 2856, 1955, 1708, 1597, 1515, 1500, 1457, 1441, 1402, 1365, 1326, 1121; MS (EI): *m/z* (%) 386 ($[M]^+$, 22.51), 105 (100); HRMS calcd. for $C_{26}H_{30}N_2O$ $[M]^+$: 386.2358; Found: 386.2361.

4.10 Synthesis of (*S*)-4-(4-chlorobenzyl)-3-methyl-1-phenyl-4-(2-butyl-2,3-butadienyl)pyrazol-5-one (*S*)-**3ae**. (zyc-4-76)

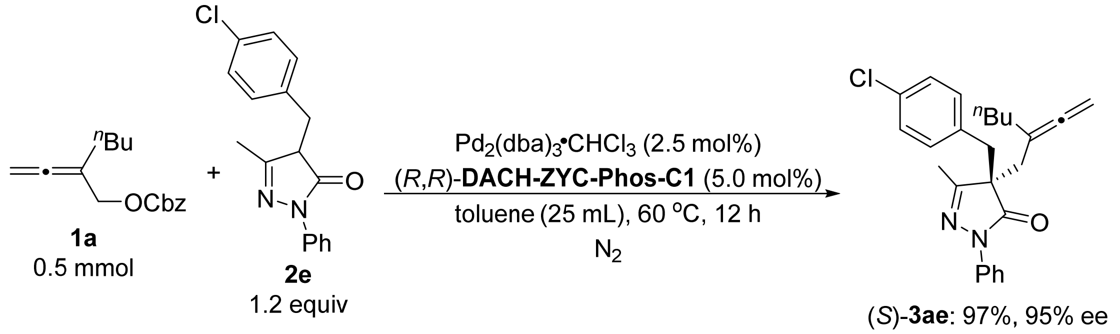

Following **Typical Procedure II**, the reaction of $Pd_2(dba)_3$•$CHCl_3$ (13.0 mg,

0.0125 mmol), (*R*,*R*)-**DACH-ZYC-Phos-C1** (14.4 mg, 0.025 mmol), toluene (5 mL), **1a** (131.0 mg, 0.5 mmol)/toluene (11.7 mL), and **2e** (179.6 mg, 0.6 mmol)/toluene (8.3 mL) afforded (*S*)-**3ae** (197.0 mg, 97%) (eluent: petroleum ether (60-90 ºC)/ diethyl ether = 40/1) as a liquid: 95% ee (HPLC condition: Chiralcel IA column, *n*-hexane/*i*-PrOH = 90/10, 1.0 mL/min, λ = 254 nm, t_R (major) = 6.7 min, t_R (minor) = 8.7 min); $[\alpha]_D^{20}$ = -27.9 (c = 0.99, $CHCl_3$); ^1H NMR (300 MHz, $CDCl_3$) δ 7.58 (d, *J* = 7.8 Hz, 2 H, ArH), 7.31 (t, *J* = 8.0 Hz, 2 H, ArH), 7.18-7.07 (m, 3 H, ArH), 7.00 (d, *J* = 8.4 Hz, 2 H, ArH), 4.56 (pentet, *J* = 2.9 Hz, 2 H, =CH_2), 3.13 (d, *J* = 13.5 Hz, 1 H, one proton of CH_2), 2.84 (d, *J* = 13.2 Hz, 1 H, one proton of CH_2), 2.66 (dt, J_1 = 15.0 Hz, J_2 = 3.2 Hz, 1 H, one proton of CH_2), 2.36 (d, *J* = 14.7 Hz, 1 H, one proton of CH_2), 2.13 (s, 3 H, CH_3), 1.92-1.79 (m, 2 H, CH_2), 1.42-1.17 (m, 4 H, CH_2 × 2), 0.82 (t, *J* = 7.2 Hz, 3 H, CH_3); ^{13}C NMR (75 MHz, $CDCl_3$) δ 205.3, 174.3, 161.1, 137.5, 133.1, 132.6, 130.4, 128.6, 128.3, 124.9, 119.1, 98.1, 77.7, 60.0, 41.9, 36.1, 32.3, 29.3, 22.0, 14.6, 13.7; IR (neat) ν (cm^{-1}) 3062, 3046, 2956, 2927, 2871, 2856, 1955, 1709, 1597, 1500, 1458, 1441, 1402, 1366, 1323, 1299, 1245, 1181, 1122, 1097, 1016; MS (EI): *m/z* (%) 408 ($[M(^{37}Cl)]^+$, 15.32), 406 ($[M(^{35}Cl)]^+$, 38.40), 125 (100); HRMS calcd. for $C_{25}H_{27}{}^{35}ClN_2O$ $[M]^+$: 406.1812; Found: 406.1814.

4.11 Synthesis of (*S*)-4-(4-bromobenzyl)-3-methyl-1-phenyl-4-(2-butyl-2,3-butadienyl)pyrazol-5-one (*S*)-**3af**. (zyc-4-97)

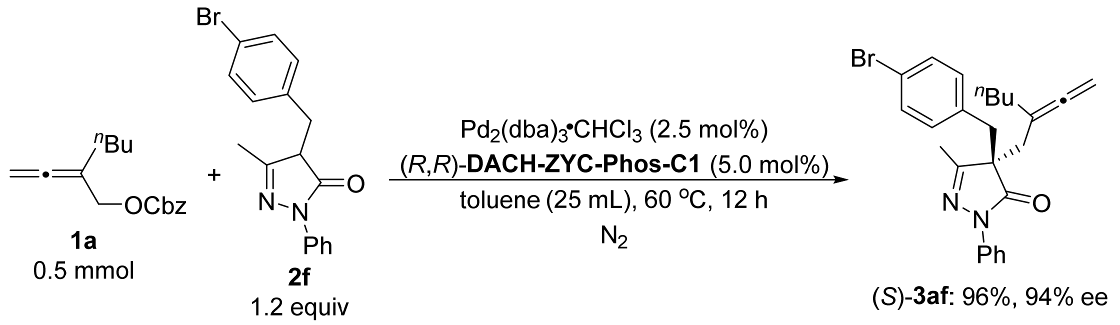

Following **Typical Procedure II**, the reaction of $Pd_2(dba)_3$•$CHCl_3$ (12.9 mg, 0.0125 mmol), (*R*,*R*)-**DACH-ZYC-Phos-C1** (14.3 mg, 0.025 mmol), toluene (5 mL), **1a** (130.4 mg, 0.5 mmol)/toluene (11.7 mL), and **2f** (205.5 mg, 0.6 mmol)/toluene (8.3 mL) afforded (*S*)-**3af** (216.7 mg, 96%) (eluent: petroleum ether (60-90 °C)/diethyl ether = 50/1) as a liquid: 94% ee (HPLC condition: Chiralcel IA column, *n*-hexane/*i*-PrOH = 90/10, 1.0 mL/min, λ = 254 nm, t_R (major) = 7.2 min, t_R (minor) = 9.7 min); $[\alpha]_D^{20}$ = -30.5 (c = 1.07, $CHCl_3$); ^{1}H NMR (300 MHz, $CDCl_3$) δ 7.60 (d, *J* = 8.4 Hz, 2 H, ArH), 7.39-7.19 (m, 4 H, ArH), 7.10 (t, *J* = 7.4 Hz, 1 H, ArH), 6.94 (d, *J* = 8.4 Hz, 2 H, ArH), 4.55 (pentet, *J* = 2.9 Hz, 2 H, =CH_2), 3.10 (d, *J* = 13.2 Hz, 1 H, one proton of CH_2), 2.81 (d, *J* = 13.5 Hz, 1 H, one proton of CH_2), 2.65 (dt, J_1 = 15.0 Hz, J_2 = 3.2 Hz, 1 H, one proton of CH_2), 2.35 (d, *J* = 15.3 Hz, 1 H, one proton of CH_2), 2.11 (s, 3 H, CH_3), 1.92-1.78 (m, 2 H, CH_2), 1.40-1.14 (m, 4 H, CH_2 × 2), 0.82 (t, *J* = 7.1 Hz, 3 H, CH_3); ^{13}C NMR (75 MHz, $CDCl_3$) δ 205.2, 174.2, 160.9, 137.4, 133.0, 131.1, 130.7, 128.5, 124.8, 121.2, 119.0, 98.0, 77.6, 59.8, 41.8, 36.0, 32.2, 29.2, 22.0, 14.5, 13.7; IR (neat) ν (cm^{-1}) 3063, 3046, 2956, 2926, 2871, 2856, 1955, 1705, 1596, 1497, 1488, 1458, 1441, 1403, 1366, 1323, 1298, 1278, 1245, 1182, 1122, 1103, 1074, 1029, 1012; MS (EI): *m/z* (%) 452 ([M(^{81}Br)]$^+$, 18.15), 450 ([M(^{79}Br)]$^+$, 16.05), 186 (100); HRMS calcd. for $C_{25}H_{27}{}^{79}BrN_2O$ [M]$^+$: 450.1307; Found: 450.1304.

4.12 Synthesis of (*S*)-4-(4-fluorobenzyl)-3-methyl-1-phenyl-4-(2-butyl-2,3-butadienyl)pyrazol-5-one (*S*)-**3ag**. (zyc-4-105)

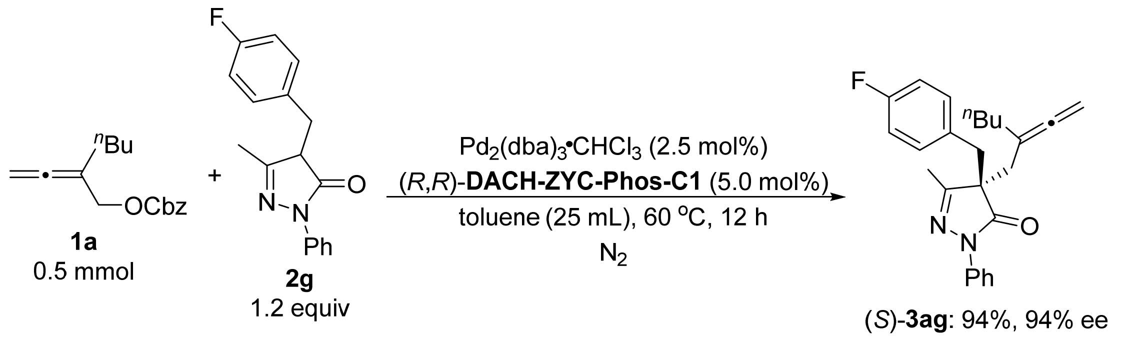

Following **Typical Procedure II**, the reaction of $Pd_2(dba)_3$•$CHCl_3$ (12.8 mg, 0.0125 mmol), (*R*,*R*)-**DACH-ZYC-Phos-C1** (14.2 mg, 0.025 mmol), toluene (5 mL), **1a** (130.1 mg, 0.5 mmol)/toluene (11.7 mL), and **2g** (169.5 mg, 0.6 mmol)/toluene (8.3 mL) afforded (*S*)-**3ag** (183.6 mg, 94%) (eluent: petroleum ether (60-90 °C)/DCM = 2/1) as a liquid: 94% ee (HPLC condition: Chiralcel IA column, *n*-hexane/*i*-PrOH = 90/10, 1.0 mL/min, λ = 254 nm, t_R (major) = 5.9 min, t_R (minor) = 7.2 min); $[\alpha]_D^{20}$ = -5.7 (c = 1.02, $CHCl_3$); ^{1}H NMR (300 MHz, $CDCl_3$) δ 7.59 (d, *J* = 8.7 Hz, 2 H, ArH), 7.30 (t, *J* = 8.0 Hz, 2 H, ArH), 7.15-6.98 (m, 3 H, ArH), 6.82 (t, *J* = 8.7 Hz, 2 H, ArH), 4.56 (pentet, *J* = 3.0 Hz, 2 H, =CH_2), 3.12 (d, *J* = 13.5 Hz, 1 H, one proton of CH_2), 2.85 (d, *J* = 13.8 Hz, 1 H, one proton of CH_2), 2.66 (dt, J_1 = 15.3 Hz, J_2 = 3.3 Hz, 1 H, one proton of CH_2), 2.36 (dt, J_1 = 15.3 Hz, J_2 = 2.1 Hz, 1 H, one proton of CH_2), 2.13 (s, 3 H, CH_3), 1.93-1.79 (m, 2 H, CH_2), 1.44-1.15 (m, 4 H, CH_2 × 2), 0.82 (t, *J* = 7.1 Hz, 3 H, CH_3); ^{13}C NMR (75 MHz, $CDCl_3$) δ 205.2, 174.3, 161.8 (d, *J* = 244.7 Hz), 161.1, 137.5, 130.6 (d, *J* = 8.3 Hz), 129.8 (d, *J* = 3.5 Hz), 128.5, 124.8, 119.0, 114.9 (d, *J* = 21.4 Hz), 98.2, 77.6, 60.0, 41.7, 35.9, 32.2, 29.3, 22.0, 14.5, 13.7; ^{19}F NMR

(282 MHz, $CDCl_3$) δ -115.4; IR (neat) ν (cm^{-1}) 3067, 3046, 2957, 2927, 2873, 2860, 1955, 1705, 1597, 1510, 1500, 1458, 1441, 1403, 1366, 1324, 1300, 1224, 1160, 1123, 1097; MS (EI): *m/z* (%) 390 ($[M]^+$, 20.90), 186 (100); HRMS calcd. for $C_{25}H_{27}FN_2O$ $[M]^+$: 390.2107; Found: 390.2105.

4.13 Synthesis of (*S*)-4-(4-cyanoylbenzyl)-3-methyl-1-phenyl-4-(2-butyl-2,3-butadienyl)pyrazol-5-one (*S*)-**3ah**. (zyc-4-88)

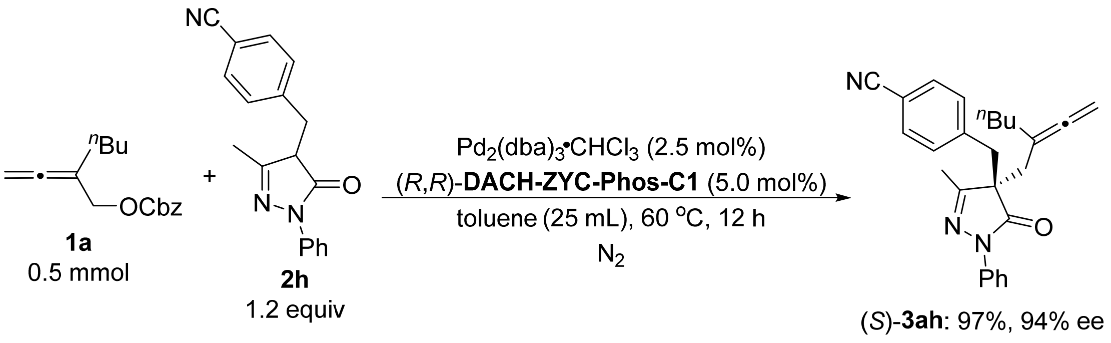

Following **Typical Procedure II**, the reaction of $Pd_2(dba)_3$•$CHCl_3$ (13.0 mg, 0.0125 mmol), (*R*,*R*)-**DACH-ZYC-Phos-C1** (14.2 mg, 0.025 mmol), toluene (5 mL), **1a** (131.5 mg, 0.5 mmol)/toluene (11.7 mL), and **2h** (173.8 mg, 0.6 mmol)/toluene (8.3 mL) afforded (*S*)-**3ah** (192.8 mg, 97%) (eluent: petroleum ether (60-90 °C)/diethyl ether = 10/1 (800 mL) to 5/1 (960 mL)) as a liquid: 94% ee (HPLC condition: Chiralcel IA column, *n*-hexane/*i*-PrOH = 90/10, 1.0 mL/min, λ = 254 nm, t_R (major) = 13.0 min, t_R (minor) = 14.6 min); $[\alpha]_D^{20}$ = -39.4 (c = 0.93, $CHCl_3$); ^1H NMR (300 MHz, $CDCl_3$) δ 7.54 (d, J = 7.5 Hz, 2 H, ArH), 7.45 (d, J = 8.4 Hz, 2 H, ArH), 7.32 (t, J = 8.0 Hz, 2 H, ArH), 7.24-7.09 (m, 3 H, ArH), 4.57 (pentet, J = 3.0 Hz, 2 H, =CH_2), 3.20 (d, J = 13.2 Hz, 1 H, one proton of CH_2), 2.94 (d, J = 13.2 Hz, 1 H, one proton of CH_2), 2.69 (dt, J_1 = 15.0 Hz, J_2 = 3.3 Hz, 1 H, one proton of CH_2), 2.40

(dt, J_1 = 15.3 Hz, J_2 = 2.1 Hz, 1 H, one proton of CH_2), 2.17 (s, 3 H, CH_3), 1.92-1.80 (m, 2 H, CH_2), 1.40-1.18 (m, 4 H, CH_2 × 2), 0.83 (t, J = 7.2 Hz, 3 H, CH_3); ^{13}C NMR (75 MHz, $CDCl_3$) δ 205.1, 173.8, 160.6, 139.5, 137.2, 131.7, 129.8, 128.5, 125.0, 118.8, 118.3, 111.0, 97.9, 77.8, 59.7, 42.1, 35.9, 32.2, 29.2, 21.9, 14.4, 13.6; IR (neat) ν (cm^{-1}) 3063, 3041, 2959, 2927, 2871, 2860, 2228, 1954, 1704, 1608, 1596, 1505, 1457, 1442, 1403, 1367, 1326, 1304, 1246, 1179, 1122, 1022; MS (EI): *m/z* (%) 397 ($[M]^+$, 32.05), 289 (100); HRMS calcd. for $C_{26}H_{27}N_3O$ $[M]^+$: 397.2154; Found: 397.2152.

4.14 Synthesis of (*S*)-4-(4-(trifluoromethyl)benzyl)-3-methyl-1-phenyl-4-(2-butyl-2,3-butadienyl)pyrazol-5-one (*S*)-**3ai**. (zyc-4-134)

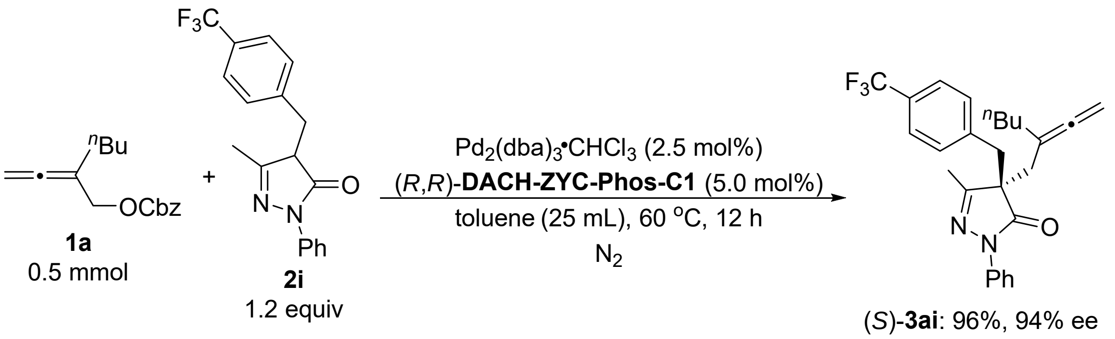

Following **Typical Procedure II**, the reaction of $Pd_2(dba)_3$•$CHCl_3$ (13.0 mg, 0.0125 mmol), (*R*,*R*)-**DACH-ZYC-Phos-C1** (14.4 mg, 0.025 mmol), toluene (5 mL), **1a** (130.0 mg, 0.5 mmol)/toluene (11.7 mL), and **2i** (199.4 mg, 0.6 mmol)/toluene (8.3 mL) afforded (*S*)-**3ai** (211.7 mg, 96%) (eluent: petroleum ether (60-90 °C)/DCM = 3/1 (1000 mL) to 2/1 (450 mL)) as a liquid: 94% ee (HPLC condition: Chiralcel IA column, *n*-hexane/*i*-PrOH = 90/10, 1.0 mL/min, λ = 254 nm, t_R (major) = 6.6 min, t_R (minor) = 7.6 min); $[\alpha]_D^{20}$ = -2.5 (c = 1.04, $CHCl_3$); ^{1}H NMR (300 MHz, $CDCl_3$) δ

7.54 (d, J = 7.8 Hz, 2 H, ArH), 7.41 (d, J = 7.8 Hz, 2 H, ArH), 7.30 (t, J = 8.0 Hz, 2 H, ArH), 7.19 (d, J = 7.8 Hz, 2 H, ArH), 7.11 (t, J = 7.4 Hz, 1 H, ArH), 4.57 (pentet, J = 2.4 Hz, 2 H, =CH_2), 3.20 (d, J = 13.2 Hz, 1 H, one proton of CH_2), 2.93 (d, J = 13.2 Hz, 1 H, one proton of CH_2), 2.70 (d, J = 15.0 Hz, 1 H, one proton of CH_2), 2.40 (d, J = 15.0 Hz, 1 H, one proton of CH_2), 2.15 (s, 3 H, CH_3), 1.97-1.78 (m, 2 H, CH_2), 1.47-1.15 (m, 4 H, CH_2 × 2), 0.83 (t, J = 7.2 Hz, 3 H, CH_3); ^{13}C NMR (75 MHz, $CDCl_3$) δ 205.3, 174.1, 160.9, 138.3, 137.4, 129.5, 129.4 (q, J = 32.2 Hz), 128.5, 125.03, 125.00 (q, J = 4.1 Hz), 123.9 (q, J = 270.5 Hz), 119.2, 98.1, 77.7, 59.9, 42.1, 36.1, 32.3, 29.3, 22.0, 14.5, 13.7; ^{19}F NMR (282 MHz, $CDCl_3$) δ -63.0; IR (neat) ν (cm^{-1}) 3067, 3047, 2958, 2928, 2877, 2860, 1955, 1709, 1618, 1597, 1500, 1458, 1442, 1419, 1402, 1367, 1325, 1246, 1165, 1126, 1069, 1019; MS (EI): *m/z* (%) 440 ($[M]^+$, 44.26), 332 (100); HRMS calcd. for $C_{26}H_{27}F_3N_2O$ $[M]^+$: 440.2075; Found: 440.2078.

4.15 Synthesis of (*S*)-3-methyl-4-(3-methoxylbenzyl)-1-phenyl-4-(2-butyl-2,3-butadienyl)pyrazol-5-one (*S*)-**3aj**. (zyc-4-101)

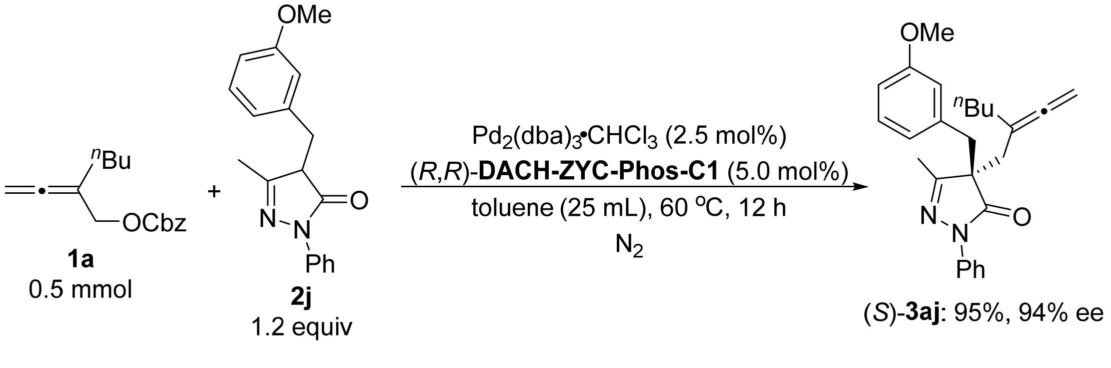

Following **Typical Procedure II**, the reaction of $Pd_2(dba)_3$•$CHCl_3$ (13.0 mg, 0.0125 mmol), (*R*,*R*)-**DACH-ZYC-Phos-C1** (14.2 mg, 0.025 mmol), toluene (5 mL),

1a (130.1 mg, 0.5 mmol)/toluene (11.7 mL), and **2j** (176.6 mg, 0.6 mmol)/toluene (8.3 mL) afforded (*S*)-**3aj** (191.3 mg, 95%) (eluent: petroleum ether (60-90 °C)/diethyl ether = 40/1 (1300 mL) to 20/1 (1000 mL)) as a liquid: 94.0% ee (HPLC condition: Chiralcel IA column, *n*-hexane/*i*-PrOH = 90/10, 1.0 mL/min, λ = 254 nm, t_R (major) = 6.3 min, t_R (minor) = 9.7 min); $[\alpha]_D^{20}$ = +1.9 (c = 0.96, $CHCl_3$); ^{1}H NMR (300 MHz, $CDCl_3$) δ 7.61 (d, *J* = 8.7 Hz, 2 H, ArH), 7.31 (t, *J* = 7.8 Hz, 2 H, ArH), 7.17-7.00 (m, 2 H, ArH), 6.73-6.57 (m, 3 H, ArH), 4.56 (pentet, *J* = 2.9 Hz, 2 H, =CH_2), 3.57 (s, 3 H, CH_3), 3.17 (d, *J* = 13.5 Hz, 1 H, one proton of CH_2), 2.85 (d, *J* = 13.2 Hz, 1 H, one proton of CH_2), 2.67 (dt, J_1 = 15.3 Hz, J_2 = 3.2 Hz, 1 H, one proton of CH_2), 2.38 (d, *J* = 14.7 Hz, 1 H, one proton of CH_2), 2.13 (s, 3 H, CH_3), 1.94-1.80 (m, 2 H, CH_2), 1.41-1.15 (m, 4 H, CH_2 × 2), 0.83 (t, *J* = 7.2 Hz, 3 H, CH_3); ^{13}C NMR (75 MHz, $CDCl_3$) δ 205.5, 174.7, 161.5, 159.3, 137.8, 135.7, 129.1, 128.6, 124.8, 121.4, 119.1, 114.2, 113.2, 98.3, 77.6, 60.1, 54.9, 42.8, 36.3, 32.4, 29.4, 22.1, 14.7, 13.8; IR (neat) ν (cm^{-1}) 3062, 3041, 3028, 2956, 2927, 2873, 2859, 2830, 1955, 1708, 1597, 1500, 1456, 1439, 1402, 1365, 1321, 1291, 1264, 1169, 1156, 1123, 1095, 1044; MS (EI): *m/z* (%) 402 ($[M]^+$, 36.14), 186 (100); HRMS calcd. for $C_{26}H_{30}N_2O_2$ $[M]^+$: 402.2307; Found: 402.2307.

4.16 Synthesis of (*S*)-4-(3-nitrobenzyl)-3-methyl-1-phenyl-4-(2-butyl-2,3-butadienyl)pyrazol-5-one (*S*)-**3ak**. (zyc-4-120)

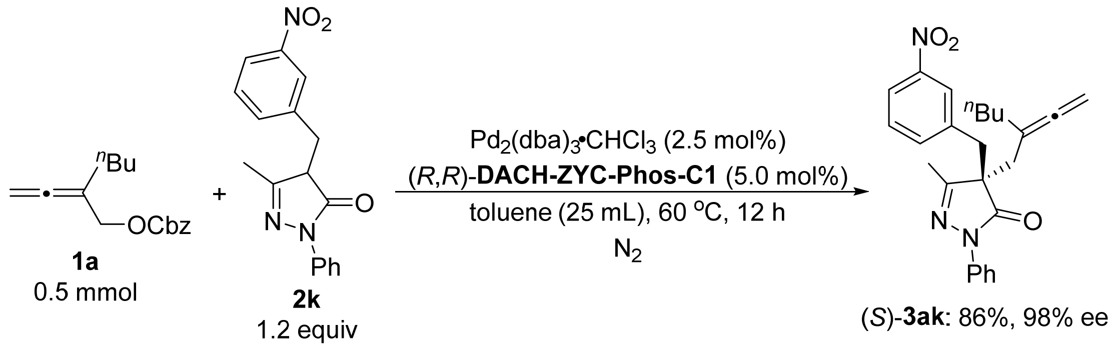

Following **Typical Procedure II**, the reaction of $Pd_2(dba)_3$•$CHCl_3$ (13.0 mg, 0.0125 mmol), (*R*,*R*)-**DACH-ZYC-Phos-C1** (14.3 mg, 0.025 mmol), toluene (5 mL), **1a** (129.7 mg, 0.5 mmol)/toluene (11.7 mL), and **2k** (185.6 mg, 0.6 mmol)/toluene (8.3 mL) afforded (*S*)-**3ak** (179.5 mg, 86%) (the residue was purified by chromatography on silica gel (eluent: petroleum ether (60-90 °C)/ethyl acetate = 20/1 (700 mL) to 10/1 (800 mL)) to afford impure (*S*)-**3ak**, which was further purified by recrystallization) as a solid: m.p. 107.0-107.4 °C (Et_2O/*n*-hexane); 98% ee (HPLC condition: Chiralcel IA column, *n*-hexane/*i*-PrOH = 90/10, 1.0 mL/min, λ = 254 nm, t_R (major) = 10.9 min, t_R (minor) = 14.0 min); $[\alpha]_D^{20}$ = -65.3 (c = 1.02, $CHCl_3$); ^1H NMR (300 MHz, $CDCl_3$) δ 8.07-7.93 (m, 2 H, ArH), 7.55 (d, J = 7.8 Hz, 2 H, ArH), 7.43 (d, J = 7.5 Hz, 1 H, ArH), 7.38-7.24 (m, 3 H, ArH), 7.11 (t, J = 7.4 Hz, 1 H, ArH), 4.57 (pentet, J = 2.9 Hz, 2 H, $=CH_2$), 3.27 (d, J = 13.5 Hz, 1 H, one proton of CH_2), 3.00 (d, J = 13.5 Hz, 1 H, one proton of CH_2), 2.71 (dt, J_1 = 15.3 Hz, J_2 = 3.3 Hz, 1 H, one proton of CH_2), 2.42 (dt, J_1 = 15.3 Hz, J_2 = 2.1 Hz, 1 H, one proton of CH_2), 2.21 (s, 3 H, CH_3), 1.94-1.82 (m, 2 H, CH_2), 1.43-1.15 (m, 4 H, CH_2 × 2), 0.83 (t, J = 7.1 Hz, 3 H, CH_3); ^{13}C NMR (75 MHz, $CDCl_3$) δ 205.2, 173.9, 160.7, 147.8, 137.3, 136.1, 135.4, 129.1, 128.6, 125.1, 123.9, 122.4, 119.0, 98.1, 78.0, 59.9, 41.9, 35.9, 32.4, 29.3, 22.1, 14.6, 13.7; IR (neat) ν (cm^{-1}) 3098, 3062, 3037, 2976, 2957, 2927, 2856, 1953,

1698, 1597, 1527, 1502, 1492, 1457, 1439, 1420, 1401, 1372, 1347, 1334, 1323, 1141, 1098, 1085; MS (EI): *m/z* (%) 417 ($[M]^+$, 100); Anal. Calcd. for $C_{25}H_{27}N_3O_3$ (%): C 71.92, H 6.52, N 10.06; Found: C 71.87, H 6.52, N 9.99.

4.17 Synthesis of (*S*)-4-ethyl-3-methyl-1-phenyl-4-(2-butyl-2,3-butadienyl)pyrazol-5-one (*S*)-**3al**. (zyc-4-64)

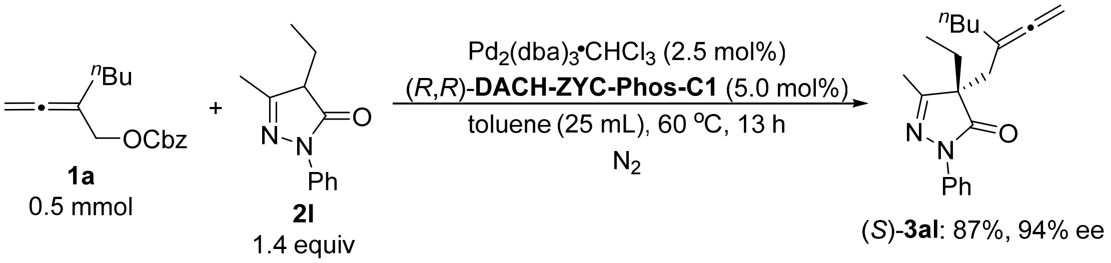

Following **Typical Procedure II**, the reaction of $Pd_2(dba)_3$•$CHCl_3$ (12.9 mg, 0.0125 mmol), (*R*,*R*)-**DACH-ZYC-Phos-C1** (14.3 mg, 0.025 mmol), toluene (5 mL), **1a** (131.0 mg, 0.5 mmol)/toluene (11.7 mL), and **2l** (141.6 mg, 0.7 mmol)/toluene (8.3 mL) afforded (*S*)-**3al** (134.6 mg, 87%) (the residue was purified by chromatography on silica gel (eluent: petroleum ether (60-90 °C)/ethyl acetate = 40/1) to afford a pure part of (*S*)-**3al** and the impure part was further purified by chromatography on silica gel (eluent: petroleum ether (60-90 °C)/ethyl acetate = 40/1)) as a liquid: 94% ee (HPLC condition: Chiralcel IA column, *n*-hexane/*i*-PrOH = 95/5, 1.0 mL/min, λ = 254 nm, t_R (minor) = 5.3 min, t_R (major) = 5.8 min); $[\alpha]_D^{20}$ = +152.9 (c = 0.99, $CHCl_3$); ^1H NMR (300 MHz, $CDCl_3$) δ 7.89 (d, *J* = 7.8 Hz, 2 H, ArH), 7.38 (t, *J* = 8.0 Hz, 2 H, ArH), 7.15 (t, *J* = 7.4 Hz, 1 H, ArH), 4.55 (pentet, *J* = 3.1 Hz, 2 H, =CH_2), 2.53 (dt, J_1 = 15.0 Hz, J_2 = 3.3 Hz, 1 H, one proton of CH_2), 2.25 (d, *J* = 15.0 Hz, 1 H, one proton of CH_2), 2.07 (s, 3 H, CH_3), 1.97-1.77 (m, 3 H, one proton of CH_2 and CH_2),

1.74-1.58 (m, 1 H, one proton of CH_2), 1.40-1.13 (m, 4 H, CH_2 × 2), 0.81 (t, J = 7.2 Hz, 3 H, CH_3), 0.72 (t, J = 7.5 Hz, 3 H, CH_3); ^{13}C NMR (75 MHz, $CDCl_3$) δ 205.3, 175.1, 162.3, 138.0, 128.6, 124.6, 118.6, 98.3, 77.3, 59.2, 36.3, 32.2, 29.6, 29.3, 22.0, 13.9, 13.7, 7.9; IR (neat) ν (cm^{-1}) 3063, 3041, 2960, 2931, 2873, 2856, 1955, 1713, 1619, 1597, 1500, 1458, 1403, 1386, 1365, 1311, 1261, 1136, 1094; MS (EI): *m/z* (%) 310 ($[M]^+$, 29.93), 187 (100); HRMS calcd. for $C_{20}H_{26}N_2O$ $[M]^+$: 310.2045; Found: 310.2042.

4.18 Synthesis of (*S*)-4-allyl-3-methyl-1-phenyl-4-(2-butyl-2,3-butadienyl)pyrazol-5-one (*S*)-**3am**. (zyc-4-87)

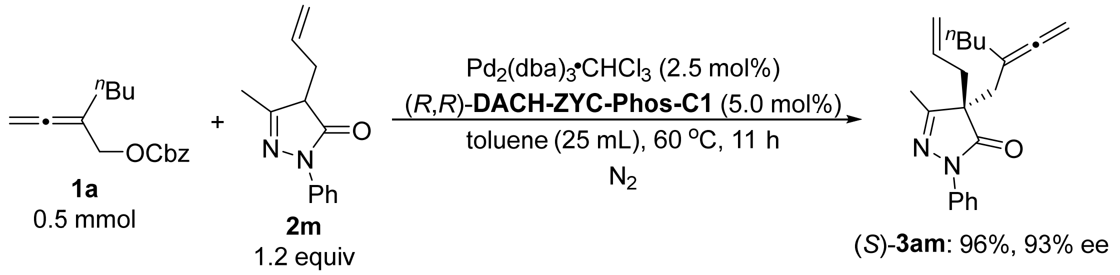

Following **Typical Procedure II**, the reaction of $Pd_2(dba)_3$•$CHCl_3$ (13.0 mg, 0.0125 mmol), (*R*,*R*)-**DACH-ZYC-Phos-C1** (14.2 mg, 0.025 mmol), toluene (5 mL), **1a** (131.5 mg, 0.5 mmol)/toluene (11.7 mL), and **2m** (128.4 mg, 0.6 mmol)/toluene (8.3 mL) afforded (*S*)-**3am** (155.0 mg, 96%) (eluent: petroleum ether (60-90 °C)/diethyl ether = 50/1) as a liquid: 93% ee (HPLC condition: Chiralcel OD column, *n*-hexane/*i*-PrOH = 98/2, 1.0 mL/min, λ = 254 nm, t_R (major) = 4.7 min, t_R (minor) = 5.2 min); $[\alpha]_D^{20}$ = +98.5 (c = 0.975, $CHCl_3$); ^{1}H NMR (300 MHz, $CDCl_3$) δ 7.87 (d, J = 8.1 Hz, 2 H, ArH), 7.37 (t, J = 8.0 Hz, 2 H, ArH), 7.14 (t, J = 7.5 Hz, 1 H, ArH), 5.57-5.39 (m, 1 H, =CH), 5.18-4.96 (m, 2 H, =CH_2), 4.55 (pentet, J = 2.9 Hz, 2 H,

$CH_2=C=C$), 2.65-2.45 (m, 2 H, CH_2), 2.43-2.20 (m, 2 H, CH_2), 2.08 (s, 3 H, CH_3), 1.91-1.73 (m, 2 H, CH_2), 1.40-1.14 (m, 4 H, $CH_2 \times 2$), 0.81 (t, J = 6.9 Hz, 3 H, CH_3); ^{13}C NMR (75 MHz, $CDCl_3$) δ 205.3, 174.6, 161.8, 137.9, 130.3, 128.6, 124.6, 119.5, 118.7, 98.2, 77.4, 58.4, 40.6, 35.7, 32.2, 29.3, 22.0, 14.2, 13.7; IR (neat) ν (cm^{-1}) 3079, 3062, 3054, 2956, 2928, 2877, 2859, 1955, 1714, 1642, 1616, 1597, 1500, 1458, 1436, 1402, 1365, 1321, 1243, 1120, 1098, 1031; MS (EI): *m/z* (%) 322 ($[M]^+$, 41.06), 239 (100); HRMS calcd. for $C_{21}H_{26}N_2O$ $[M]^+$: 322.2045; Found: 322.2044.

4.19 Synthesis of (*R*)-4-allyl-3-methyl-1-phenyl-4-(2-butyl-2,3-butadienyl)pyrazol-5-one (*R*)-**3am**. (zyc-4-86)

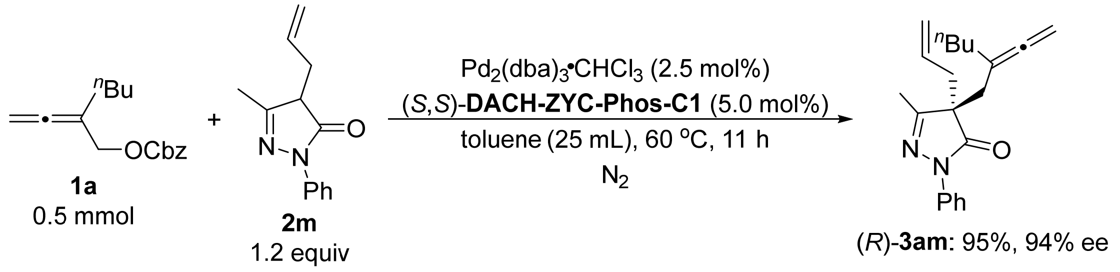

Following **Typical Procedure II**, the reaction of $Pd_2(dba)_3$•$CHCl_3$ (12.9 mg, 0.0125 mmol), (*S*,*S*)-**DACH-ZYC-Phos-C1** (14.2 mg, 0.025 mmol), toluene (5 mL), **1a** (131.0 mg, 0.5 mmol)/toluene (11.7 mL), and **2m** (128.6 mg, 0.6 mmol)/toluene (8.3 mL) afforded (*R*)-**3am** (152.1 mg, 95%) (eluent: petroleum ether (60-90 °C)/diethyl ether = 50/1) as a liquid: 94% ee (HPLC condition: Chiralcel OD column, *n*-hexane/*i*-PrOH = 98/2, 1.0 mL/min, λ = 254 nm, t_R (minor) = 4.7 min, t_R (major) = 5.1 min); $[\alpha]_D^{20}$ = -99.0 (c = 1.075, $CHCl_3$); ^1H NMR (300 MHz, $CDCl_3$) δ 7.86 (d, J = 7.8 Hz, 2 H, ArH), 7.37 (t, J = 8.0 Hz, 2 H, ArH), 7.15 (t, J = 7.5 Hz, 1 H, ArH), 5.57-5.40 (m, 1 H, =CH), 5.19-4.99 (m, 2 H, $=CH_2$), 4.55 (pentet, J = 3.0 Hz, 2 H,

CH_2=C=C), 2.64-2.49 (m, 2 H, CH_2), 2.43-2.23 (m, 2 H, CH_2), 2.09 (s, 3 H, CH_3), 1.89-1.77 (m, 2 H, CH_2), 1.40-1.14 (m, 4 H, $CH_2 \times 2$), 0.82 (t, J = 7.1 Hz, 3 H, CH_3); ^{13}C NMR (75 MHz, $CDCl_3$) δ 205.3, 174.6, 161.8, 137.9, 130.3, 128.6, 124.6, 119.5, 118.7, 98.2, 77.4, 58.4, 40.6, 35.7, 32.2, 29.3, 22.0, 14.2, 13.7; IR (neat) ν (cm^{-1}) 3079, 3058, 3041, 2956, 2929, 2869, 2860, 1955, 1714, 1642, 1597, 1500, 1458, 1435, 1402, 1365, 1321, 1244, 1121; MS (EI): *m/z* (%) 322 ($[M]^+$, 74.63), 77 (100); HRMS calcd. for $C_{21}H_{26}N_2O$ $[M]^+$: 322.2045; Found: 322.2043.

4.20 Synthesis of (*R*)-4-(α-naphthylmethyl)-3-methyl-1-phenyl-4-(2-butyl-2,3-butadienyl)pyrazol-5-one (*R*)-**3an**. (zyc-4-92)

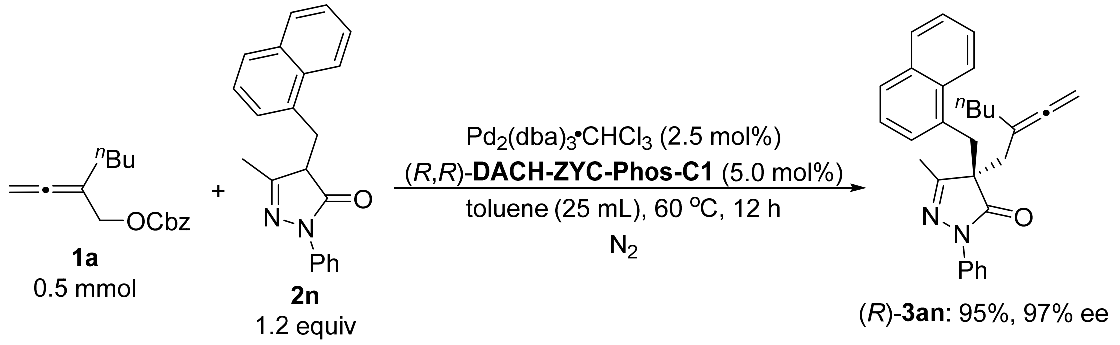

Following **Typical Procedure II**, the reaction of $Pd_2(dba)_3$•$CHCl_3$ (12.9 mg, 0.0125 mmol), (*R*,*R*)-**DACH-ZYC-Phos-C1** (14.3 mg, 0.025 mmol), toluene (5 mL), **1a** (131.1 mg, 0.5 mmol)/toluene (11.7 mL), and **2n** (188.4 mg, 0.6 mmol)/toluene (8.3 mL) afforded (*R*)-**3an** (200.9 mg, 95%) (eluent: petroleum ether (60-90 °C)/diethyl ether = 50/1) as a liquid: 97% ee (HPLC condition: Chiralcel IA column, *n*-hexane/*i*-PrOH = 90/10, 1.0 mL/min, λ = 254 nm, t_R (major) = 6.5 min, t_R (minor) = 11.1 min); $[\alpha]_D^{20}$ = -47.4 (c = 1.03, $CHCl_3$); ^{1}H NMR (300 MHz, $CDCl_3$) δ 8.09 (d, J = 8.7 Hz, 1 H, ArH), 7.76 (d, J = 8.1 Hz, 1 H, ArH), 7.70-7.62 (m, 1 H, ArH), 7.56 (d,

J = 8.7 Hz, 2 H, ArH), 7.52-7.37 (m, 2 H, ArH), 7.33-7.22 (m, 4 H, ArH), 7.08 (t, J = 7.4 Hz, 1 H, ArH), 4.53 (pentet, J = 3.0 Hz, 2 H, =CH_2), 3.64 (d, J = 14.1 Hz, 1 H, one proton of CH_2), 3.41 (d, J = 14.4 Hz, 1 H, one proton of CH_2), 2.78 (dt, J_1 = 15.3 Hz, J_2 = 3.5 Hz, 1 H, one proton of CH_2), 2.48 (dt, J_1 = 14.7 Hz, J_2 = 2.3 Hz, 1 H, one proton of CH_2), 1.98-1.80 (m, 5 H, CH_3 and CH_2), 1.42-1.15 (m, 4 H, $CH_2 \times 2$), 0.82 (t, J = 7.1 Hz, 3 H, CH_3); ^{13}C NMR (75 MHz, $CDCl_3$) δ 205.3, 175.1, 162.0, 137.8, 133.7, 132.0, 130.7, 128.7, 128.5, 128.1, 127.5, 125.8, 125.5, 124.9, 124.7, 123.6, 119.1, 98.3, 77.8, 59.5, 38.1, 36.1, 32.4, 29.4, 22.1, 15.1, 13.8; IR (neat) ν (cm^{-1}) 3062, 3046, 2956, 2927, 2871, 2852, 1954, 1709, 1597, 1500, 1457, 1399, 1365, 1322, 1121; MS (EI): *m/z* (%) 422 ([M]$^+$, 21.84), 186 (100); HRMS calcd. for $C_{29}H_{30}N_2O$ [M]$^+$: 422.2358; Found: 422.2361.

4.21 Synthesis of (*R*)-4-(2-hydroxyethyl)-3-methyl-1-phenyl-4-(2-butyl-2,3-butadienyl)pyrazol-5-one (*R*)-**3ao**. (zyc-4-126)

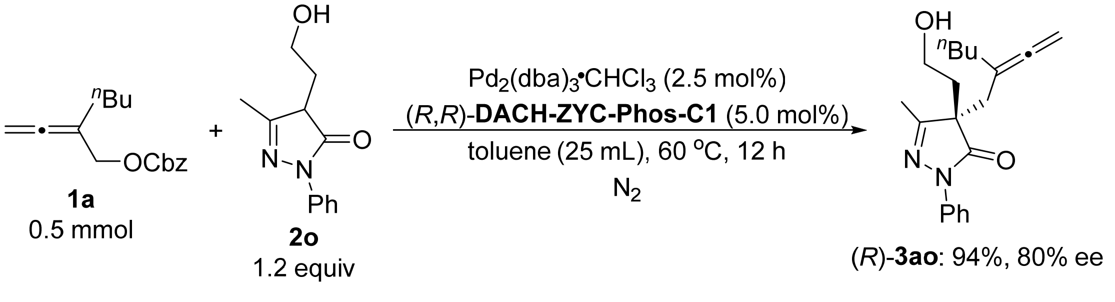

Following **Typical Procedure II**, the reaction of $Pd_2(dba)_3$•$CHCl_3$ (13.0 mg, 0.0125 mmol), (*R*,*R*)-**DACH-ZYC-Phos-C1** (14.4 mg, 0.025 mmol), toluene (5 mL), **1a** (130.4 mg, 0.5 mmol)/toluene (11.7 mL), and **2o** (131.4 mg, 0.6 mmol)/toluene (8.3 mL) afforded (*R*)-**3ao** (153.6 mg, 94%) (eluent: petroleum ether (60-90 °C)/ethyl acetate = 9/1 (500 mL) to 4/1 (700 mL)) as a liquid: 80% ee (HPLC condition:

Chiralcel OD column, *n*-hexane/*i*-PrOH = 90/10, 1.0 mL/min, λ = 254 nm, t_R (minor) = 5.9 min, t_R (major) = 7.3 min); $[\alpha]_D^{20}$ = +131.9 (c = 0.995, $CHCl_3$); ^{1}H NMR (300 MHz, $CDCl_3$) δ 7.82 (d, *J* = 7.8 Hz, 2 H, ArH), 7.36 (t, *J* = 8.0 Hz, 2 H, ArH), 7.15 (t, *J* = 7.4 Hz, 1 H, ArH), 4.54 (pentet, *J* = 2.6 Hz, 2 H, $=CH_2$), 3.46 (t, *J* = 6.5 Hz, 2 H, OCH_2), 2.97 (brs, 1 H, OH), 2.53 (dt, J_1 = 15.0 Hz, J_2 = 2.7 Hz, 1 H, one proton of CH_2), 2.25 (d, *J* = 14.7 Hz, 1 H, one proton of CH_2), 2.14-1.95 (m, 4 H, CH_3 and one proton of CH_2), 1.95-1.69 (m, 3 H, CH_2 and one proton of CH_2), 1.39-1.10 (m, 4 H, $CH_2 \times 2$), 0.80 (t, *J* = 7.1 Hz, 3 H, CH_3); ^{13}C NMR (75 MHz, $CDCl_3$) δ 205.4, 175.4, 163.1, 137.8, 128.6, 124.8, 118.9, 97.7, 77.3, 57.9, 57.0, 38.5, 36.6, 32.2, 29.3, 22.0, 14.1, 13.7; IR (neat) ν (cm^{-1}) 3428, 3063, 3045, 2956, 2928, 2872, 1955, 1709, 1596, 1500, 1458, 1429, 1404, 1367, 1323, 1128, 1048, 1001; MS (EI): *m/z* (%) 326 ([M]$^+$, 100); HRMS calcd. for $C_{20}H_{26}N_2O_2$ [M]$^+$: 326.1994; Found: 326.1992.

4.22 Synthesis of (*R*)-4-(2-((tert-butyldimethylsilyl)oxy)ethyl)-3-methyl-1-phenyl-4-(2-butyl-2,3-butadienyl)pyrazol-5-one (*R*)-**3ap**. (zyc-4-146)

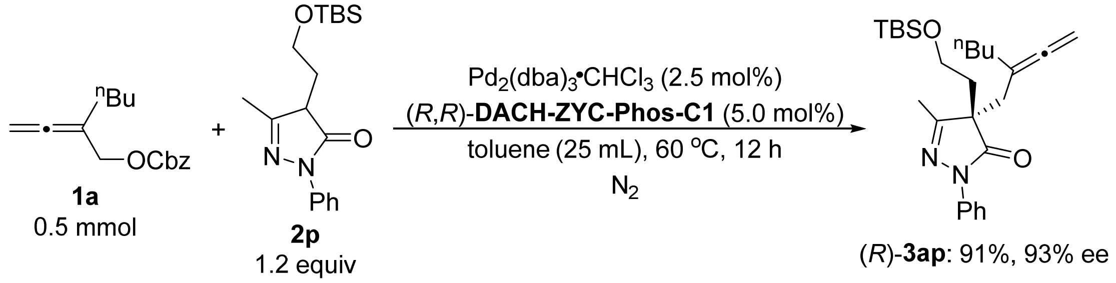

Following **Typical Procedure II**, the reaction of $Pd_2(dba)_3$•$CHCl_3$ (13.0 mg, 0.0125 mmol), (*R*,*R*)-**DACH-ZYC-Phos-C1** (14.2 mg, 0.025 mmol), toluene (5 mL), **1a** (130.1 mg, 0.5 mmol)/toluene (11.7 mL), and **2p** (200 mg, 0.6 mmol)/toluene (8.3 mL) afforded (*R*)-**3ap** (201.4 mg, 91%) (eluent: petroleum ether (60-90 °C)/DCM =

3/1) as a liquid: 93% ee (HPLC condition: Chiralcel IA column, *n*-hexane/*i*-PrOH = 95/5, 1.0 mL/min, λ = 254 nm, t_R (major) = 4.6 min, t_R (minor) = 6.9 min); $[\alpha]_D^{20}$ = +82.8 (c = 0.935, $CHCl_3$); ^{1}H NMR (300 MHz, $CDCl_3$) δ 7.89 (d, *J* = 7.8 Hz, 2 H, ArH), 7.37 (t, *J* = 8.0 Hz, 2 H, ArH), 7.14 (t, *J* = 7.2 Hz, 1 H, ArH), 4.64-4.44 (m, 2 H, =CH_2), 3.65-3.38 (m, 2 H, CH_2), 2.52 (d, *J* = 14.7 Hz, 1 H, one proton of CH_2), 2.31 (d, *J* = 14.4 Hz, 1 H, one proton of CH_2), 2.21-1.90 (m, 5 H, CH_3 and CH_2), 1.89-1.70 (m, 2 H, CH_2), 1.41-1.09 (m, 4 H, CH_2 × 2), 0.98-0.64 (m, 12 H, $SiCH_3$ × 3 and CH_3), -0.07 (s, 6 H, CH_3 × 2); ^{13}C NMR (75 MHz, $CDCl_3$) δ 206.0, 174.9, 163.0, 138.3, 128.6, 124.5, 118.7, 97.8, 77.0, 59.0, 57.2, 39.2, 37.7, 32.2, 29.5, 25.8, 22.1, 18.2, 14.7, 13.7, -5.8; IR (neat) ν (cm^{-1}) 3064, 3041, 2955, 2928, 2857, 1955, 1710, 1621, 1598, 1500, 1474, 1463, 1430, 1403, 1362, 1323, 1256, 1113, 1084, 1031, 1005; MS (EI): *m/z* (%) 440 ($[M]^+$, 8.04), 383 (100); HRMS calcd. for $C_{26}H_{40}N_2O_2Si$ $[M]^+$: 440.2859; Found: 440.2860.

4.23 Synthesis of (*R*)-3-methyl-1,4-diphenyl-4-(2-butyl-2,3-butadienyl)pyrazol-5-one (*R*)-**3at**. (zyc-6-145)

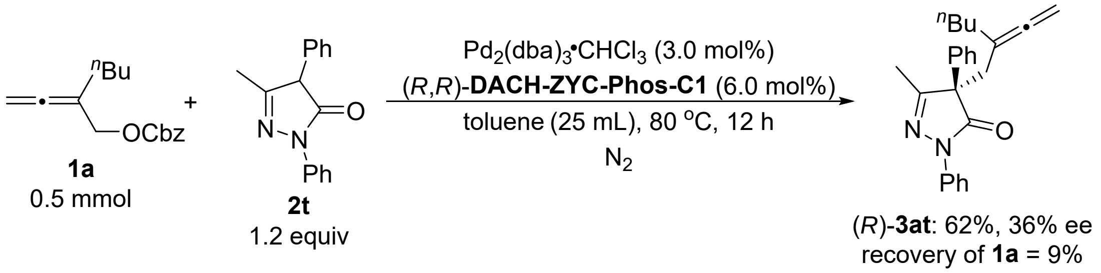

Following **Typical Procedure II**, the reaction of $Pd_2(dba)_3$•$CHCl_3$ (15.5 mg, 0.015 mmol), (*R*,*R*)-**DACH-ZYC-Phos-C1** (17.2 mg, 0.03 mmol), toluene (5 mL), **1a** (130.1 mg, 0.5 mmol)/toluene (11.7 mL), and **2t** (150.3 mg, 0.6 mmol)/toluene (8.3

mL) afforded (*R*)-**3at** (111.2 mg, 62%) (the residue was purified by chromatography on silica gel (eluent: petroleum ether (60-90 °C)/diethyl ether = 60/1) to afford a part of pure (*R*)-**3at** and the impure part was further purified by chromatography on silica gel (eluent: petroleum ether (60-90 °C)/diethyl ether = 60/1)) as a liquid: 36% ee (HPLC condition: Chiralcel IA column, *n*-hexane/*i*-PrOH = 90/10, 1.0 mL/min, λ = 254 nm, t_R (minor) = 12.4 min, t_R (major) = 26.1 min); $[\alpha]_D^{20}$ = +18.5 (c = 0.95, $CHCl_3$); ^{1}H NMR (300 MHz, $CDCl_3$) δ 7.91 (d, *J* = 8.1 Hz, 2 H, ArH), 7.48-7.21 (m, 7 H, ArH), 7.17 (t, *J* = 7.2 Hz, 1 H, ArH), 4.72-4.49 (m, 2 H, =CH_2), 3.09 (d, *J* = 14.7 Hz, 1 H, one proton of CH_2), 2.74 (d, *J* = 14.7 Hz, 1 H, one proton of CH_2), 2.08 (s, 3 H, CH_3), 2.01-1.87 (m, 2 H, CH_2), 1.49-1.15 (m, 4 H, CH_2 × 2), 0.83 (t, *J* = 7.2 Hz, 3 H, CH_3); ^{13}C NMR (75 MHz, $CDCl_3$) δ 205.5, 174.4, 162.8, 138.1, 136.2, 129.2, 128.7, 128.1, 126.0, 124.8, 118.7, 97.9, 77.8, 62.2, 34.2, 32.5, 29.5, 22.1, 14.8, 13.8; IR (neat) ν (cm^{-1}) 3062, 2956, 2928, 2871, 1955, 1715, 1597, 1499; MS (EI): *m/z* (%) 358 ($[M]^+$, 31.00), 250 (100); HRMS calcd. for $C_{24}H_{26}N_2O$ $[M]^+$: 358.2045; Found: 358.2046.

4.24 Synthesis of (*S*)-4-benzyl-3-ethyl-1-phenyl-4-(2-butyl-2,3-butadienyl)pyrazol-5-one (*S*)-**3aq**. (zyc-4-129)

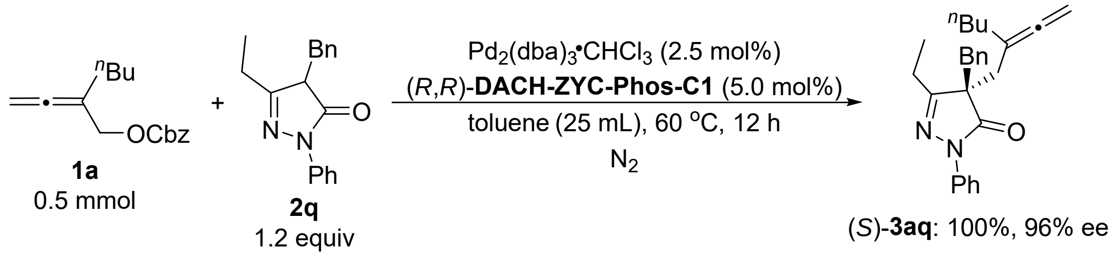

Following **Typical Procedure II**, the reaction of $Pd_2(dba)_3$•$CHCl_3$ (12.9 mg,

0.0125 mmol), (*R*,*R*)-**DACH-ZYC-Phos-C1** (14.3 mg, 0.025 mmol), toluene (5 mL), **1a** (130.2 mg, 0.5 mmol)/toluene (11.7 mL), and **2q** (166.6 mg, 0.6 mmol)/toluene (8.3 mL) afforded (*S*)-**3aq** (194.1 mg, 100%) (eluent: petroleum ether (60-90 °C)/diethyl ether = 50/1) as a liquid: 96% ee (HPLC condition: Chiralcel IA column, *n*-hexane/*i*-PrOH = 90/10, 1.0 mL/min, λ = 254 nm, t_R (major) = 5.4 min, t_R (minor) = 8.6 min); $[\alpha]_D^{20}$ = -23.6 (c = 0.905, $CHCl_3$); ^{1}H NMR (300 MHz, $CDCl_3$) δ 7.60 (d, *J* = 8.1 Hz, 2 H, ArH), 7.28 (t, *J* = 8.0 Hz, 2 H, ArH), 7.19-6.95 (m, 6 H, ArH), 4.53 (pentet, *J* = 2.9 Hz, 2 H, =CH_2), 3.15 (d, *J* = 13.2 Hz, 1 H, one proton of CH_2), 2.87 (d, *J* = 13.2 Hz, 1 H, one proton of CH_2), 2.66 (dt, J_1 = 15.3 Hz, J_2 = 3.5 Hz, 1 H, one proton of CH_2), 2.58-2.28 (m, 3 H, CH_2 and one proton of CH_2), 1.97-1.74 (m, 2 H, CH_2), 1.43-1.12 (m, 7 H, CH_2 × 2 and CH_3), 0.83 (t, *J* = 7.1 Hz, 3 H, CH_3); ^{13}C NMR (75 MHz, $CDCl_3$) δ 205.1, 174.8, 164.8, 137.8, 134.1, 129.0, 128.4, 128.0, 127.1, 124.6, 119.2, 98.4, 77.5, 60.1, 43.1, 36.0, 32.4, 29.3, 22.1, 21.7, 13.7, 8.5; IR (neat) ν (cm^{-1}) 3063, 3031, 2956, 2931, 2872, 1955, 1708, 1597, 1500, 1456, 1440, 1402, 1359, 1326, 1125, 1081, 1053; MS (EI): *m/z* (%) 386 ($[M]^+$, 41.60), 200 (100); HRMS calcd. for $C_{26}H_{30}N_2O$ $[M]^+$: 386.2358; Found: 386.2359.

4.25 Synthesis of (*S*)-4-benzyl-3-isopropyl-1-phenyl-4-(2-butyl-2,3-butadienyl)-pyrazol-5-one (*S*)-**3ar**. (zyc-4-141)

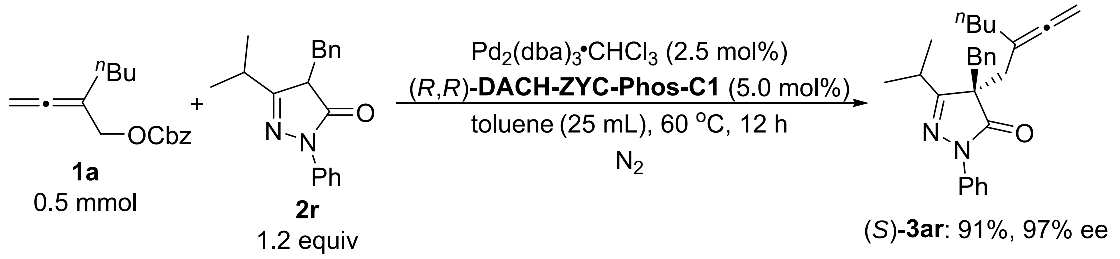

Following **Typical Procedure II**, the reaction of $Pd_2(dba)_3$•$CHCl_3$ (13.0 mg, 0.0125 mmol), (*R*,*R*)-**DACH-ZYC-Phos-C1** (14.3 mg, 0.025 mmol), toluene (5 mL), **1a** (130.4 mg, 0.5 mmol)/toluene (11.7 mL), and **2r** (175.6 mg, 0.6 mmol)/toluene (8.3 mL) afforded (*S*)-**3ar** (182.3 mg, 91%) (eluent: petroleum ether (60-90 ºC)/DCM = 2/1) as a liquid: 97% ee (HPLC condition: Chiralcel IA column, *n*-hexane/*i*-PrOH = 90/10, 1.0 mL/min, λ = 254 nm, t_R (major) = 6.1 min, t_R (minor) = 13.3 min); $[\alpha]_D^{20}$ = -88.1 (c = 0.91, $CHCl_3$); ^{1}H NMR (300 MHz, $CDCl_3$) δ 7.63 (d, *J* = 8.7 Hz, 2 H, ArH), 7.29 (t, *J* = 7.8 Hz, 2 H, ArH), 7.19-7.00 (m, 6 H, ArH), 4.47 (pentet, *J* = 3.2 Hz, 2 H, =CH_2), 3.17 (d, *J* = 13.2 Hz, 1 H, one proton of CH_2), 2.94 (d, *J* = 13.2 Hz, 1 H, one proton of CH_2), 2.82-2.61 (m, 2 H, CH and one proton of CH_2), 2.37 (dt, J_1 = 16.5 Hz, J_2 = 3.3 Hz, 1 H, one proton of CH_2), 1.98-1.78 (m, 2 H, CH_2), 1.44-1.12 (m, 10 H, CH_2 × 2 and CH_3 × 2), 0.86 (t, *J* = 7.1 Hz, 3 H, CH_3); ^{13}C NMR (75 MHz, $CDCl_3$) δ 204.2, 174.6, 167.5, 137.9, 134.0, 129.3, 128.4, 127.9, 127.2, 124.5, 119.1, 99.2, 78.7, 59.7, 44.1, 35.7, 33.0, 29.2, 28.5, 22.2, 21.5, 19.9, 13.8; IR (neat) ν (cm^{-1}) 3063, 3032, 2959, 2930, 2872, 1959, 1709, 1598, 1500, 1456, 1397, 1381, 1359, 1326, 1121, 1082, 1060, 1029, 1012; MS (EI): *m/z* (%) 400 ([M]$^+$, 100); HRMS calcd. for $C_{27}H_{32}N_2O$ [M]$^+$: 400.2515; Found: 400.2513.

4.26 Synthesis of (*S*)-4-benzyl-1,3-diphenyl-4-(2-butyl-2,3-butadienyl)pyrazol-5-one (*S*)-**3as**. (zyc-4-142)

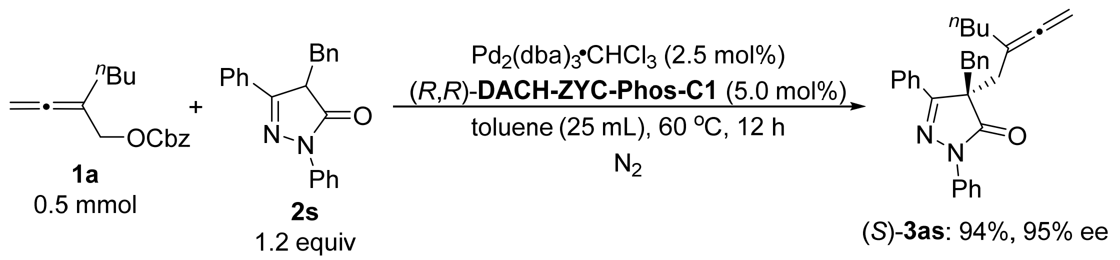

Following **Typical Procedure II**, the reaction of $Pd_2(dba)_3$•$CHCl_3$ (13.0 mg, 0.0125 mmol), (*R*,*R*)-**DACH-ZYC-Phos-C1** (14.2 mg, 0.025 mmol), toluene (5 mL), **1a** (130.0 mg, 0.5 mmol)/toluene (11.7 mL), and **2s** (195.8 mg, 0.6 mmol)/toluene (8.3 mL) afforded (*S*)-**3as** (204.5 mg, 94%) (eluent: petroleum ether (60-90 °C)/DCM = 2/1) as a liquid: 95% ee (HPLC condition: Chiralcel IA column, *n*-hexane/*i*-PrOH = 90/10, 1.0 mL/min, λ = 254 nm, t_R (major) = 6.1 min, t_R (minor) = 9.5 min); $[\alpha]_D^{20}$ = +57.6 (c = 1.00, $CHCl_3$); ^{1}H NMR (300 MHz, $CDCl_3$) δ 7.96-7.81 (m, 2 H, ArH), 7.65 (d, *J* = 8.6 Hz, 2 H, ArH), 7.51-7.35 (m, 3 H, ArH), 7.30 (t, *J* = 8.0 Hz, 2 H, ArH), 7.11 (t, *J* = 7.5 Hz, 1 H, ArH), 7.07-6.94 (m, 3 H, ArH), 6.94-6.83 (m, 2 H, ArH), 4.47-4.20 (m, 2 H, =CH_2), 3.30 (s, 2 H, CH_2), 2.97 (dt, J_1 = 15.3 Hz, J_2 = 3.6 Hz, 1 H, one proton of CH_2), 2.80 (d, *J* = 15.3 Hz, 1 H, one proton of CH_2), 1.86-1.65 (m, 2 H, CH_2), 1.35-1.02 (m, 4 H, CH_2 × 2), 0.75 (t, *J* = 7.2 Hz, 3 H, CH_3); ^{13}C NMR (75 MHz, $CDCl_3$) δ 205.4, 175.1, 158.5, 137.6, 133.9, 132.0, 129.9, 129.3, 128.6, 128.5, 127.8, 127.1, 126.5, 125.1, 119.5, 98.3, 77.6, 60.2, 44.2, 37.6, 32.2, 29.2, 21.9, 13.7; IR (neat) ν (cm^{-1}) 3063, 3031, 2955, 2927, 2873, 2858, 1955, 1713, 1597, 1496, 1456, 1446, 1394, 1354, 1311, 1134, 1028; MS (EI): *m/z* (%) 434 ([M]$^+$, 96.02), 343 (100); HRMS calcd. for $C_{30}H_{30}N_2O$ [M]$^+$: 434.2358; Found: 434.2358.

4.27 Synthesis of (*S*)-4-benzyl-1-cyclohexyl-3-methyl-4-(2-methyl-2,3-butadienyl)

pyrazol-5-one (*S*)-**3bu**. (zyc-6-140)

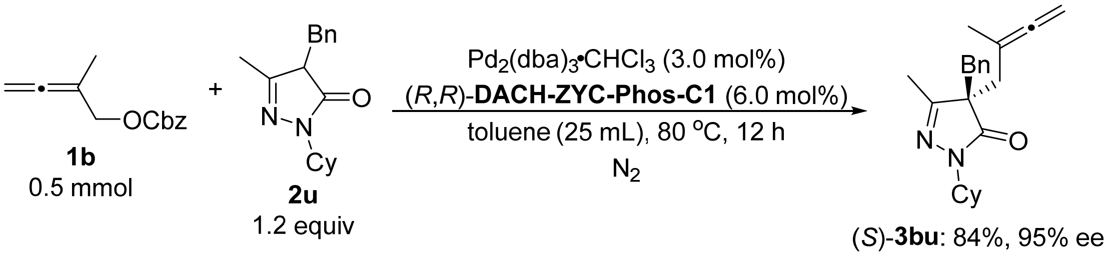

Following **Typical Procedure II**, the reaction of $Pd_2(dba)_3$•$CHCl_3$ (15.5 mg, 0.015 mmol), (*R*,*R*)-**DACH-ZYC-Phos-C1** (17.0 mg, 0.03 mmol), toluene (5 mL), **1b** (109.4 mg, 0.5 mmol)/toluene (11.7 mL), and **2u** (162.5 mg, 0.6 mmol)/toluene (8.3 mL) afforded (*S*)-**3bu** (146.2 mg, 84%, purity = 97%) (eluent: petroleum ether (60-90 °C)/ethyl acetate = 50/1 (1200 mL) to 40/1 (1200 mL) to 20/1 (500 mL)) as a liquid: 95% ee (HPLC condition: Chiralcel IA column, *n*-hexane/*i*-PrOH = 90/10, 1.0 mL/min, λ = 254 nm, t_R (major) = 5.2 min, t_R (minor) = 6.2 min); $[\alpha]_D^{20}$ = +64.3 (c = 1.02, $CHCl_3$); ^{1}H NMR (300 MHz, $CDCl_3$) δ 7.24-7.12 (m, 3 H, ArH), 7.11-6.99 (m, 2 H, ArH), 4.66-4.46 (m, 2 H, =CH_2), 3.88-3.71 (m, 1 H, CH of Cy), 3.03 (d, *J* = 13.2 Hz, 1 H, one proton of CH_2), 2.80 (d, *J* = 13.5 Hz, 1 H, one proton of CH_2), 2.55 (d, *J* = 14.4 Hz, 1 H, one proton of CH_2), 2.42 (d, *J* = 14.1 Hz, 1 H, one proton of CH_2), 2.10 (s, 3 H, CH_3), 1.82-1.44 (m, 7 H, CH_3 and CH_2 × 2), 1.35-0.80 (m, 6 H, CH_2 × 3); ^{13}C NMR (75 MHz, $CDCl_3$) δ 206.6, 174.5, 160.1, 134.4, 129.2, 128.0, 127.0, 93.7, 75.0, 59.4, 51.7, 42.0, 37.5, 30.4, 30.0, 25.3, 25.2, 25.0, 19.2, 14.7; IR (neat) ν (cm^{-1}) 3067, 3031, 2985, 2934, 2856, 1957, 1698; MS (EI): *m/z* (%) 336 ($[M]^+$, 100); HRMS calcd. for $C_{22}H_{28}N_2O$ $[M]^+$: 336.2202; Found: 336.2202.

4.28 Synthesis of (*S*)-1,4-dibenzyl-3-methyl-4-(2-methyl-2,3-butadienyl)pyrazol-5-

one (*S*)-**3bv**. (zyc-6-142)

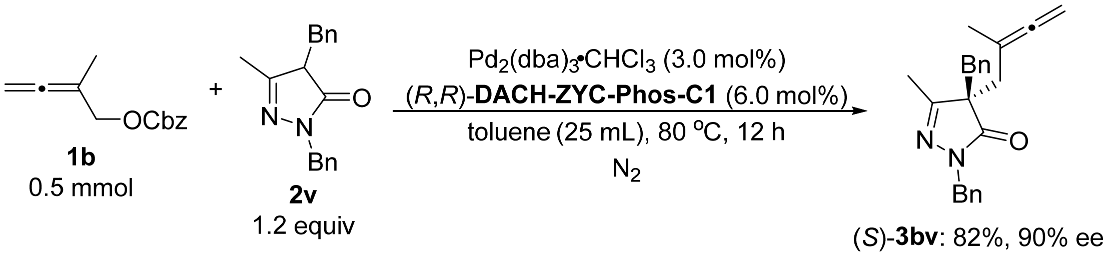

Following **Typical Procedure II**, the reaction of $Pd_2(dba)_3$•$CHCl_3$ (15.5 mg, 0.015 mmol), (*R*,*R*)-**DACH-ZYC-Phos-C1** (17.0 mg, 0.03 mmol), toluene (5 mL), **1b** (109.1 mg, 0.5 mmol)/toluene (11.7 mL), and **2v** (167.2 mg, 0.6 mmol)/toluene (8.3 mL) afforded (*S*)-**3bv** (144.3 mg, 82%, purity = 98%) (eluent: petroleum ether (60-90 °C)/ethyl acetate = 30/1 (600 mL) to 20/1 (1200 mL)) as a solid: m.p. 55.4-56.3 °C (DCM/*n*-hexane), 90% ee (HPLC condition: Chiralcel IA column, *n*-hexane/*i*-PrOH = 90/10, 1.0 mL/min, λ = 254 nm, t_R (major) = 8.6 min, t_R (minor) = 10.1 min); $[\alpha]_D^{20}$ = +74.3 (c = 0.94, $CHCl_3$); ^1H NMR (300 MHz, $CDCl_3$) δ 7.29-7.00 (m, 8 H, ArH), 6.87-6.71 (m, 2 H, ArH), 4.68 (d, *J* = 15.3 Hz, 1 H, one proton of NCH_2), 4.58-4.37 (m, 3 H, =CH_2 and one proton of NCH_2), 3.08 (d, *J* = 13.2 Hz, 1 H, one proton of CH_2), 2.86 (d, *J* = 13.5 Hz, 1 H, one proton of CH_2), 2.57 (d, *J* = 14.7 Hz, 1 H, one proton of CH_2), 2.41 (d, *J* = 14.4 Hz, 1 H, one proton of CH_2), 2.09 (s, 3 H, CH_3), 1.58 (t, *J* = 2.7 Hz, 3 H, CH_3); ^{13}C NMR (75 MHz, $CDCl_3$) δ 206.6, 175.5, 161.0, 135.9, 134.4, 129.3, 128.24, 128.20, 127.6, 127.1, 127.0, 93.3, 75.2, 58.9, 47.2, 41.8, 37.7, 19.5, 14.7; IR (neat) ν (cm^{-1}) 3088, 3.63, 3031, 2980, 2918, 1956, 1703, 1497, 1455; MS (EI): *m/z* (%) 344 ($[M]^+$, 100); HRMS calcd. for $C_{23}H_{24}N_2O$ $[M]^+$: 344.1889; Found: 344.1887.

5. The gram-scale reaction and the synthetic applications.

5.1 Gram-scale synthesis of (*S*)-4-allyl-3-methyl-1-phenyl-4-(2-butyl-2,3-butadienyl)pyrazol-5-one (*S*)-**3am**. (zyc-4-151)

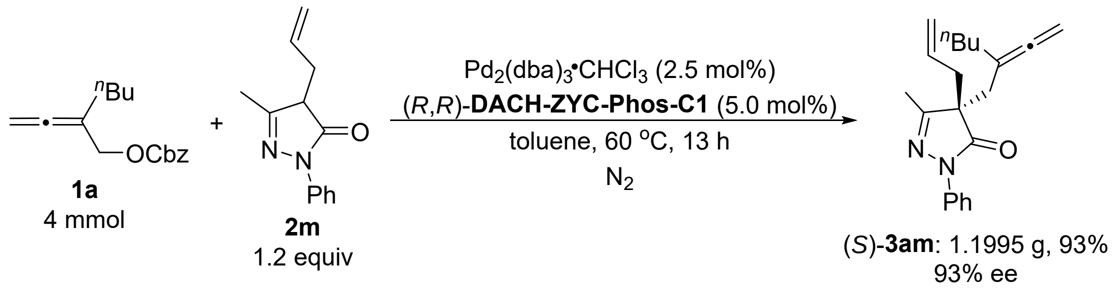

Following **Typical Procedure II**, the reaction of $Pd_2(dba)_3$•$CHCl_3$ (0.1036 g, 0.1 mmol), (*R*,*R*)-**DACH-ZYC-Phos-C1** (0.1135 g, 0.2 mmol), toluene (40 mL), **1a** (1.0394 g, 4.0 mmol)/toluene (93.6 mL), and **2m** (1.0291 g, 4.8 mmol)/toluene (66.4 mL) afforded (*S*)-**3am** (1.1995 g, 93%) (eluent: petroleum ether (60-90 °C)/diethyl ether = 50/1) as a liquid: 93% ee (HPLC condition: Chiralcel OD column, *n*-hexane/*i*-PrOH = 98/2, 1.0 mL/min, λ = 254 nm, t_R (major) = 4.5 min, t_R (minor) = 4.9 min); $[\alpha]_D^{20}$ = +101.5 (c = 1.025, $CHCl_3$); ^{1}H NMR (300 MHz, $CDCl_3$) δ 7.86 (d, *J* = 8.7 Hz, 2 H, ArH), 7.38 (t, *J* = 8.0 Hz, 2 H, ArH), 7.16 (t, *J* = 7.4 Hz, 1 H, ArH), 5.57-5.38 (m, 1 H, =CH), 5.19-4.97 (m, 2 H, =CH_2), 4.55 (pentet, *J* = 3.0 Hz, 2 H, CH_2=C=C), 2.65-2.48 (m, 2 H, CH_2), 2.43-2.22 (m, 2 H, CH_2), 2.09 (s, 3 H, CH_3), 1.90-1.73 (m, 2 H, CH_2), 1.40-1.12 (m, 4 H, CH_2 × 2), 0.82 (t, *J* = 7.2 Hz, 3 H, CH_3); ^{13}C NMR (75 MHz, $CDCl_3$) δ 205.4, 174.7, 161.9, 138.0, 130.4, 128.7, 124.7, 119.6, 118.8, 98.3, 77.5, 58.5, 40.7, 35.8, 32.3, 29.4, 22.1, 14.3, 13.8.

5.2 Synthesis of (3a*S*,5*S*)-**5am**. (zyc-4-193, zyc-5-102)

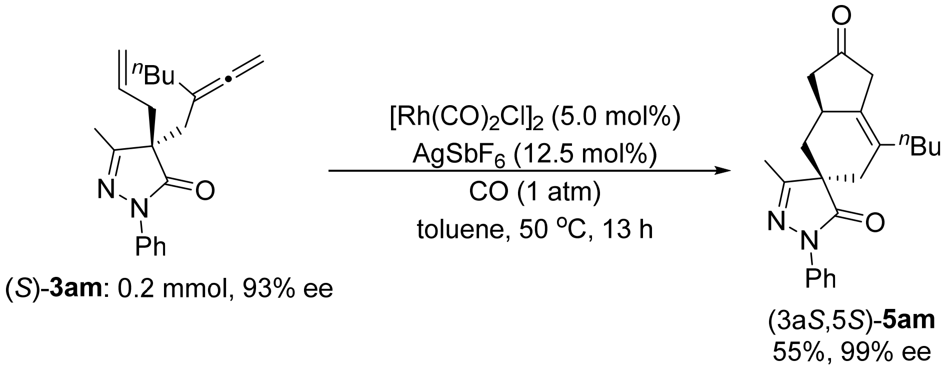

To a flame-dried Schlenk tube were added $[Rh(CO)_2Cl]_2$ (4.0 mg, 0.01 mmol) and $AgSbF_6$ (8.7 mg, 0.025 mmol) under N_2 atmosphere. The N_2 atmosphere was replaced with CO for three times with a balloon of CO (2 L) followed by the addition of toluene (1 mL). The resulting mixture was stirred at room temperature for 10 min. (*S*)-**3am** (64.0 mg, 0.2 mmol) and toluene (1 mL) were added sequentially. The reaction was complete after being stirred at 50 °C for 13 hours as monitored by TLC (eluent: petroleum ether/ethyl acetate = 5/1). The resulting mixture was filtrated through a short column of silica gel eluted with ethyl acetate (10 mL × 3). After evaporation, the residue was purified by chromatography on silica gel (eluent: petroleum ether (60-90 °C)/ethyl acetate = 10/1) to afford impure (3a*S*,5*S*)-**5am,** which was further purified by second round chromatography on silica gel (eluent: petroleum ether (60-90 °C)/ethyl acetate = 20/1 (1200 mL) to 15/1 (500 mL)) to afford pure (3a*S*,5*S*)-**5am** (38.5 mg, 55%, the absolute configuration of the new chiral center was established by NOE study) as a liquid: 99% ee (HPLC condition: Chiralcel IA column, *n*-hexane/*i*-PrOH = 90/10, 1.0 mL/min, λ = 254 nm, t_R (major) = 9.1 min, t_R (minor) = 12.4 min); $[\alpha]_D^{20}$ = +150.5 (c = 1.075, $CHCl_3$); ^1H NMR (300 MHz, $CDCl_3$) δ 7.92 (d, *J* = 7.8 Hz, 2 H, ArH), 7.41 (t, *J* = 7.8 Hz, 2 H, ArH), 7.19 (t, *J* =

7.4 Hz, 1 H, ArH), 3.15 (d, J = 21.9 Hz, 1 H, one proton of $COCH_2C$=), 2.97 (s, 1 H, CH), 2.84 (d, J = 22.2 Hz, 1 H, one proton of $COCH_2C$=), 2.74-2.51 (m, 2 H, one proton of $COCH_2$ and one proton of =CCH_2), 2.18 (s, 3 H, CH_3), 2.15-1.93 (m, 5 H, one proton of $COCH_2$, one proton of CH_2, one proton of =CCH_2 and CH_2), 1.81 (t, J = 12.5 Hz, 1 H, one proton of CH_2), 1.51-1.20 (m, 4 H, $CH_2 \times 2$), 0.91 (t, J = 6.8 Hz, 3 H, CH_3); ^{13}C NMR (75 MHz, $CDCl_3$) δ 214.7, 175.8, 163.9, 138.0, 129.2, 129.0, 128.8, 125.0, 118.7, 53.1, 47.3, 41.3, 34.6, 34.0, 33.3, 32.2, 29.9, 22.5, 16.1, 13.9; IR (neat) ν (cm^{-1}) 3058, 3037, 2956, 2928, 2858, 1749, 1710, 1597, 1500, 1459, 1445, 1397, 1368, 1323, 1227, 1187, 1152, 1124; MS (EI): *m/z* (%) 350 ($[M]^+$, 39.96), 187 (100); HRMS calcd. for $C_{22}H_{26}N_2O_2$ $[M]^+$: 350.1994; Found: 350.1993.

5.3 Synthesis of (*S*,*Z*)-4-benzyl-3-methyl-4-(2-(5-methylbenzo[*b*]oxepin-3(2H)-ylidene)-2-(trimethylsilyl)ethyl)-phenylpyrazol-5-one ((*S,Z*)-**6da**). (zyc-6-31)

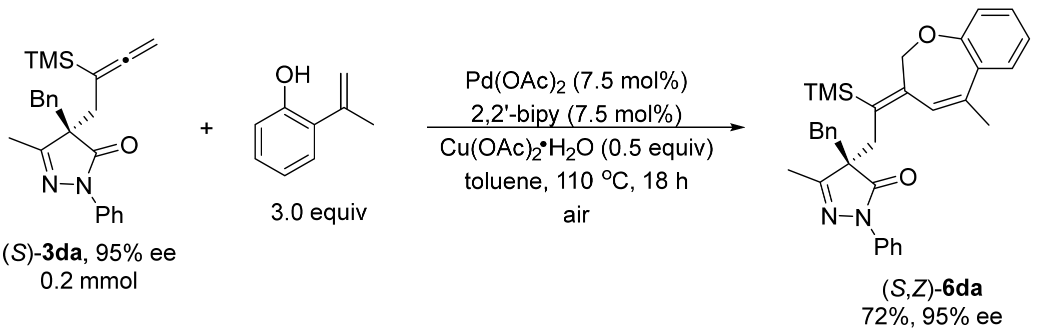

To a Schlenk tube were added $Pd(OAc)_2$ (3.5 mg, 0.015 mmol), $Cu(OAc)_2•H_2O$ (20.1 mg, 0.1 mmol), 2,2'-bipyridine (2.5 mg, 0.015 mmol), (*S*)-**3da** (77.6 mg, 0.2 mmol)/toluene (0.8 mL), and 2-(propen-2-yl)phenol (80.4 mg, 0.6 mmol)/toluene (0.8 mL) sequentially under air atmosphere at room temperature. The flask was put into a pre-heat oil bath of 110 °C and the reaction was complete after being stirred at 110 °C

for 18 hours as monitored by TLC (eluent: petroleum ether/ethyl acetate = 20/1). The resulting mixture was filtrated through a short column of silica gel eluted with ethyl acetate (10 mL × 3). After evaporation, the residue was purified by chromatography on silica gel (eluent: petroleum ether (60-90 °C)/ethyl acetate = 50/1) to afford (*S,Z*)-**6da** (74.7 mg, 72%) as a solid: m.p. 151.7-152.6 °C (*n*-hexane/DCM); 95% ee (HPLC condition: Chiralcel IA column, *n*-hexane/*i*-PrOH = 90/10, 1.0 mL/min, λ = 254 nm, t_R (major) = 5.7 min, t_R (minor) = 7.3 min; $[\alpha]_D^{20}$ = +85.1 (c = 0.98, $CHCl_3$); ^{1}H NMR (300 MHz, $CDCl_3$) δ 7.55 (d, *J* = 7.5 Hz, 2 H, ArH), 7.37 (dd, J_1 = 7.8 Hz, J_2 = 1.5 Hz, 1 H, ArH), 7.24 (t, *J* = 8.0 Hz, 2 H, ArH), 7.20-6.94 (m, 9 H, ArH), 6.70 (s, 1 H, =CH), 4.74 (d, *J* = 12.9 Hz, 1 H, one proton of OCH_2), 4.64 (d, *J* = 12.9 Hz, 1 H, one proton of OCH_2), 3.39 (d, *J* = 13.5 Hz, 1 H, one proton of CH_2), 3.05 (d, *J* = 15.3 Hz, 1 H, one proton of CH_2), 3.00 (d, *J* = 13.8 Hz, 1 H, one proton of CH_2), 2.92 (d, *J* = 14.1 Hz, 1 H, one proton of CH_2), 2.16 (s, 3 H, CH_3), 2.15 (s, 3 H, CH_3), 0.29 (s, 9 H, CH_3 × 3); ^{13}C NMR (75 MHz, $CDCl_3$) δ 174.5, 161.6, 160.1, 148.9, 137.6, 135.0, 134.5, 132.5, 129.14, 129.08, 128.9, 128.7, 128.5, 128.3, 128.0, 127.2, 124.9, 122.8, 119.9, 119.4, 74.3, 61.4, 41.3, 36.1, 26.6, 15.9, 1.8; IR (neat) ν (cm^{-1}) 3059, 3027, 2946, 2912, 2899, 1702, 1595, 1500, 1485, 1456, 1438, 1397, 1364, 1354, 1313, 1272, 1262, 1247, 1218, 1126, 1045; MS (EI): *m/z* (%) 520 ($[M]^+$, 0.004), 265 ($[M\text{-}Bn\text{-}Me\text{-}Ph\text{-}TMS]^+$, 14.71), 91 (100); Anal. Calcd. for $C_{33}H_{36}N_2O_2Si$ (%): C 76.11, H 6.97, N 5.38; Found: C 76.09, H 7.03, N 5.26.

5.4 Synthesis of (*S*)-4-benzyl-4-(but-2-ynyl)-3-methyl-phenylpyrazol-5-one ((*S*)-**7da**).

(zyc-6-40)

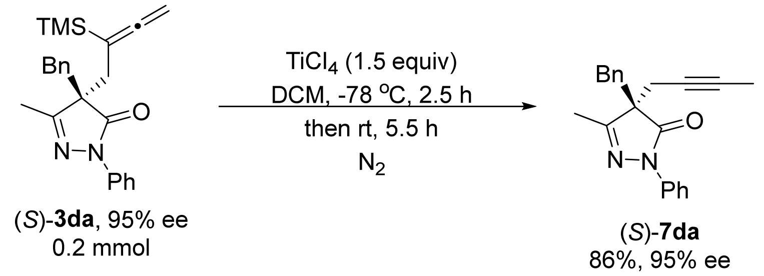

To a flame-dried Schlenk flask were added (*S*)-**3da** (77.6 mg, 0.2 mmol) and DCM (1.0 mL). The flask was put into a pre-cooled alcohol bath of -78 °C for 5 min followed by the addition of $TiCl_4$ (32.9 μL, 56.9 mg, d = 1.73 g/mL, 0.3 mmol) under nitrogen atmosphere at -78 °C. The reaction was complete after being stirred at -78 °C for 2.5 hours and at room temperature for 5.5 hours as monitored by TLC (eluent: petroleum ether/ethyl acetate = 20/1). A mixture of ether and water (50 mL, v/v = 1/1) was added to quench the reaction. The resulting mixture was transferred to a separatory funnel. After the separation of the aqueous phase, the organic phase was washed with brine and dried over anhydrous Na_2SO_4. After filtration and evaporation of the solvent，the residue was purified by chromatography on silica gel (eluent: petroleum ether (60-90 °C)/ethyl acetate = 60/1 (600 mL) to 50/1 (500 mL)) to afford (*S*)-**7da** (54.4mg, 86%) as a liquid: 95% ee (HPLC condition: Chiralcel IA column, *n*-hexane/*i*-PrOH = 90/10, 1.0 mL/min, λ = 254 nm, t_R (minor) = 8.8 min, t_R (major) = 12.5 min; $[\alpha]_D^{20}$ = -74.4 (c = 0.97, $CHCl_3$); ^{1}H NMR (300 MHz, $CDCl_3$) δ 7.63 (d, J = 8.1 Hz, 2 H, ArH), 7.32 (t, J = 7.8 Hz, 2 H, ArH), 7.22-7.03 (m, 6 H, ArH), 3.17 (d, J = 13.5 Hz, 1 H, one proton of $PhCH_2$), 2.97 (d, J = 13.8 Hz, 1 H, one proton of $PhCH_2$), 2.71 (d, J_1 = 16.5 Hz, 1 H, one proton of ≡CCH_2), 2.59 (d, J_1 = 16.5 Hz, 1 H, one proton of ≡CCH_2), 2.19 (s, 3 H, CH_3), 1.68 (s, 3 H, CH_3); ^{13}C NMR (75 MHz,

$CDCl_3$) δ 174.1, 161.2, 137.6, 134.6, 128.9, 128.6, 128.3, 127.3, 125.1, 119.4, 79.2, 72.1, 59.9, 40.0, 24.9, 14.7, 3.3; IR (neat) ν (cm^{-1}) 3063, 3032, 2919, 2854, 2237, 1713, 1596, 1500, 1456, 1439, 1404, 1367, 1305, 1240, 1183, 1123, 1082, 1029; MS (EI): *m/z* (%) 316 ($[M]^+$, 100); HRMS calcd. for $C_{21}H_{20}N_2O$ $[M]^+$: 316.1576; Found: 316.1578.

6.1 Synthesis of Pd(II)-DACH-ZYC-Phos-C1 complex. (zyc-4-198, zyc-4-161)

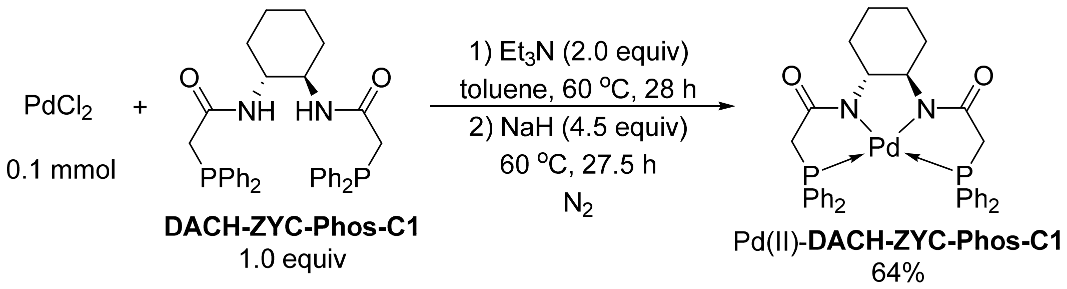

To a flame-dried Schlenk flask were added $PdCl_2$ (17.8 mg, 0.1 mmol), Et_3N (20.3 mg, 0.2 mmol), toluene (4 mL), **DACH-ZYC-Phos-C1** (56.9 mg, 0.1 mmol), and toluene (1 mL). After being stirred for 28 hours at 60 °C, NaH (18.0 mg, 0.45 mmol, 60% by weight in oil) was added to the mixture. The reaction was complete after being stirred at 60 °C for 27.5 hours as monitored by TLC (eluent: DCM/MeOH = 20/1). After evaporation of the solvent, the crude residual was purified by chromatography on silica gel (eluent: DCM/MeOH = 40/1) to afford impure Pd(II)-**DACH-ZYC-Phos-C1** (49.6 mg). The second chromatography on silica gel (eluent: $CHCl_3$/MeOH = 100/1) also afforded impure Pd(II)-**DACH-ZYC-Phos-C1** (44.0 mg). Pure Pd(II)-**DACH-ZYC-Phos-C1** complex (42.9 mg, 64%) was obtained by recrystallization from $CHCl_3$/*n*-hexane as a solid: m.p. 268.6-270.8 °C; ^1H NMR (300 MHz, $CDCl_3$) δ 7.59-7.07 (m, 20 H, ArH), 3.85 (dd, J_1 = 16.5 Hz, J_2 = 11.4 Hz,

2 H, one proton $COCH_2 \times 2$), 3.78-3.65 (m, 2 H, NCH × 2), 3.42-3.10 (m, 4 H, CH_2 and one proton $COCH_2 \times 2$), 1.73-1.49 (m, 2 H, CH_2), 1.47-1.16 (m, 4 H, $CH_2 \times 2$); ^{13}C NMR (75 MHz, $CDCl_3$) δ 175.2-174.9 (m), 132.8-132.1 (m), 131.24, 131.16, 130.3, 129.6, 129.3, 129.25-128.68 (m), 128.6, 73.7, 43.5-42.8 (m), 32.6, 25.9; ^{31}P NMR (121.5 MHz, $CDCl_3$) δ -4.1; IR (KBr) ν (cm^{-1}) 2923, 2854, 1594, 1577, 1561; MS (ESI): *m/z* (%) 670 ($[M]^+$), 671 ($[M+H]^+$), 693 ($[M+Na]^+$); HRMS calcd. for $C_{34}H_{35}N_2O_2P_2{}^{106}Pd$ $[M+H]^+$: 671.1203; Found: 671.1203.

6.2 The reaction of allene 1a and pyrazolone 2a with Pd(II)-DACH-ZYC-Phos-C1 complex. (zyc-4-195)

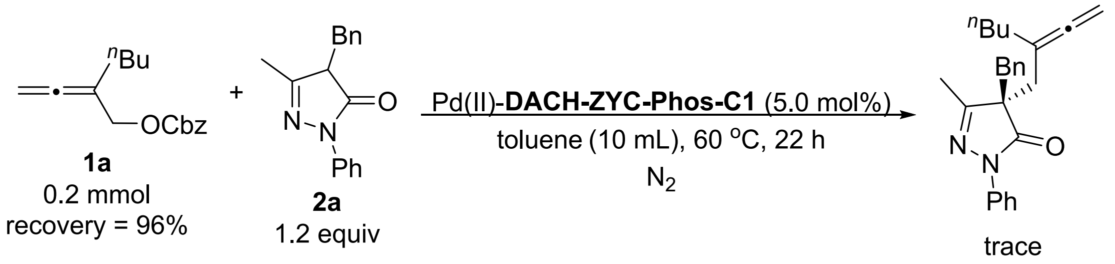

To a flame-dried Schlenk flask were added Pd(II)-**DACH-ZYC-Phos-C1** complex (6.8 mg, 0.01 mmol), toluene (2 mL), **1a** (52.7 mg, 0.2 mmol)/toluene (4.7 mL), and **2a** (63.4 mg, 0.24 mmol)/toluene (3.3 mL) sequentially under nitrogen atmosphere at room temperature. The flask was put into a pre-heat oil bath and the reaction was stirred at 60 °C for 22 hours, affording trace (*S*)-**3aa** with 96% recovery of **1a**.

6.3 The reaction of allene 1a and pyrazolone 2a with Pd(II)-DACH-ZYC-Phos-C1 complex and AcOH. (zyc-6-144)

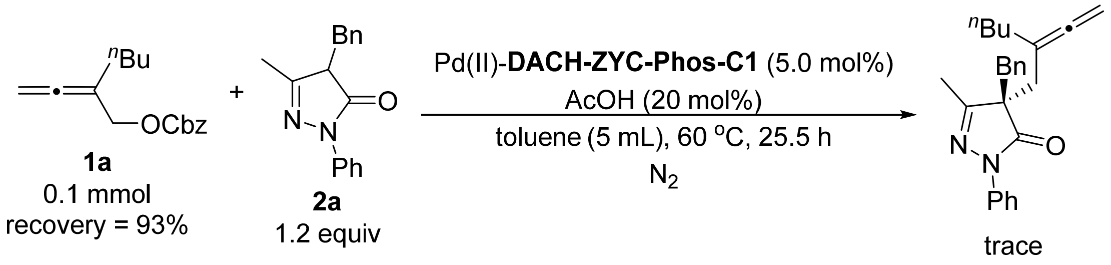

To a flame-dried Schlenk flask were added Pd(II)-**DACH-ZYC-Phos-C1** complex (3.5 mg, 0.005 mmol), toluene (1 mL), AcOH (1.1 μL, d = 1.05 g/mL, 1.2 mg, 0.02 mmol), **1a** (26.0 mg, 0.1 mmol)/toluene (2.4 mL), and **2a** (31.5 mg, 0.12 mmol)/toluene (1.6 mL) sequentially under nitrogen atmosphere at room temperature. The flask was put into a pre-heat oil bath and the reaction was stirred at 60 °C for 25.5 hours, affording trace (*S*)-**3aa** with 93% recovery of **1a**.

7. DFT calculations

7.1 Computational method.

All calculations were performed with the Gaussian 09 program.[11] Geometries have been fully optimized with the density functional theory of B3LYP method[12-16], including Grimme's D3 dispersion corrections (B3LYP-D3 with Becke–Johnson damping).[15,16] The LANL2DZ basis set in conjunction with the LANL2DZ pseudopotential[17-19] was used for palladium, while the 6-31G(d)[20,21] basis set was used for carbon, hydrogen, nitrogen, oxygen and phosphorus atoms. Harmonic vibration frequency calculations were conducted at the same level of theory to verify the stationary points to be minima (no imaginary frequency) or saddle points (one imaginary frequency). Intrinsic reaction coordinate (IRC)[22-24] calculations were performed to confirm the connection of the transition structures with their

corresponding reactants and products. The solvent effects were determined by single-point calculations of the gas-phase stationary points at M06[25,26]/SDD[27]-6-311+G(d,p) level by using IEFPCM[28] solvation model. The CYLview software was employed to show the 3D structures.[29] The reported energies are the solution-phase Gibbs free energies (ΔG_{sol}) in toluene.

7.2 The optimized structures and relative free energies of the less favourable transition states basing on the *exo*-methylene-*π*-allyl palladium complexes

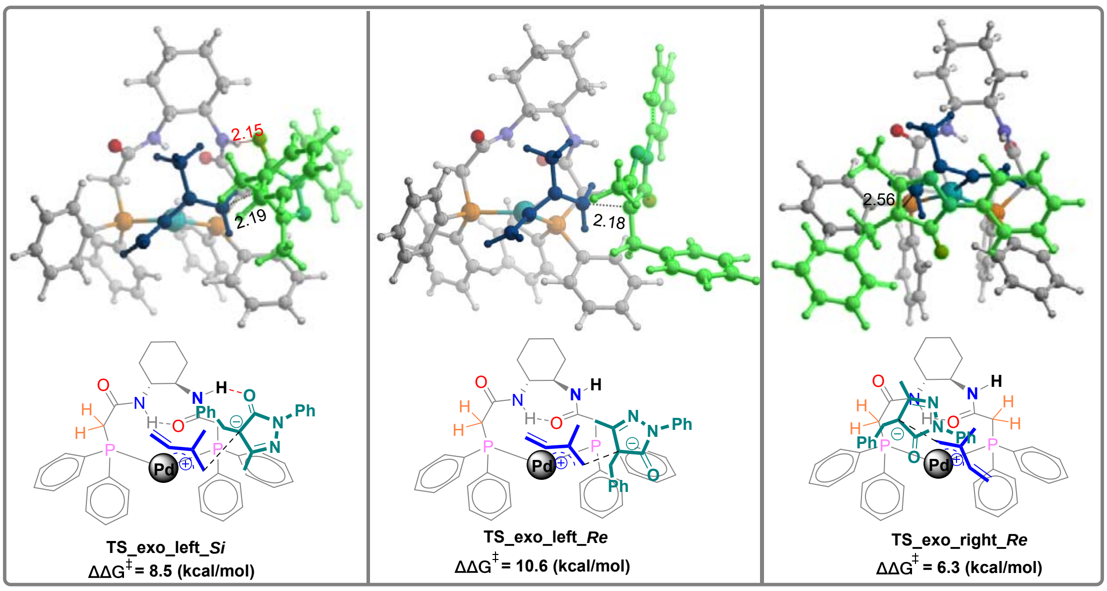

Supplementary Figure 1 Less favorable transition structures for the DACH-ZYC-Phos-C1-ligated palladium catalyst: Less favorable transition structures with *exo*-methylene-*π*-allyl palladium complexes of the reaction of **1b** with **2a** catalyzed by the **DACH-ZYC-Phos-C1**-ligated palladium catalyst. **TS_*exo*_right_*Si*** was not obtained. All energies are given with respect of **TS_left_*Si*_a**. Selected bond lengths (Å) are listed.

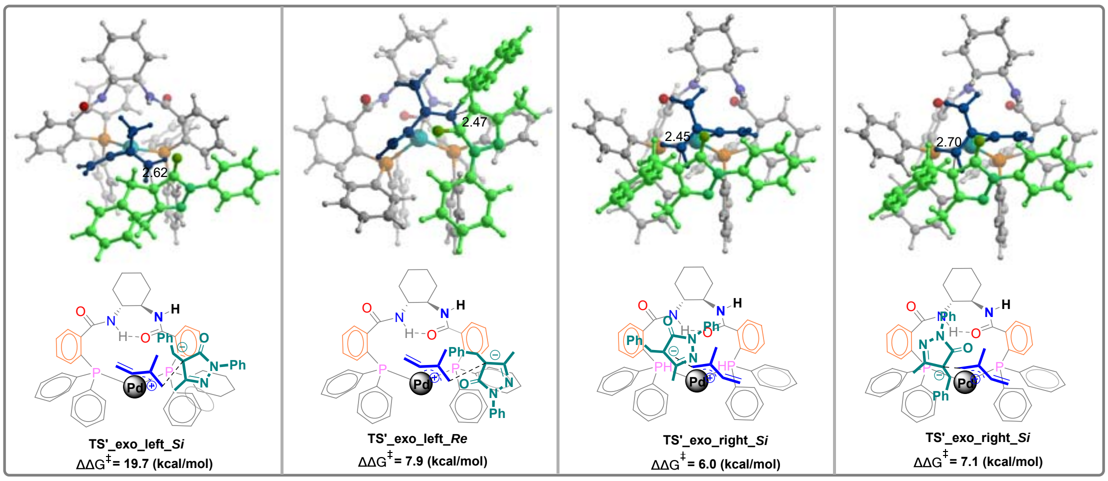

Supplementary Figure 2 Less favorable transition structures for the DACH-Phenyl-Trost-ligated palladium catalyst: Less favorable transition structures with *exo*-methylene-π-allyl palladium complexes of the reaction of **1b** with **2a** catalyzed by the DACH-Phenyl-Trost-ligated palladium catalyst. All energies are given with respect of **TS'_right_*Si***. Selected bond lengths (Å) are listed.

7.3 The plot of non-covalent interactions (NCI) of TS_left_*Si*_a

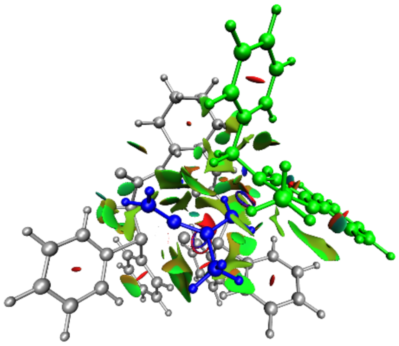

Supplementary Figure 3. The NCI plot of TS_left_*Si*_a obtained by Multiwfn[30,31] and VMD[32] softwares.

7.4 Energies of the transition states

Supplementary Table 1. Electronic energies (E_{elec}), Gibbs free energies (G_{298}), thermal correction to Gibbs free energy (*cor* G_{gas}), solvation energies (E_{sol}), solvation free energies (G_{sol}) in toluene (ε = 2.37) for all stationary points of the process. Calculations were carried out at M06/6-311+G(d,p)/SDD/ IEFPCM (toluene)//B3LYP-D3(BJ)/6-31G(d)/LANL2DZ level.

species	E_{elec} (a.u.)	G_{298} (a.u.)	*cor* G_{gas} (a.u.)	E_{sol} (a.u.)	G_{sol} (a.u.)
**TS_left_*Si*_a**	-3423.391065	-3422.46509	0.925978	-3422.99298	-3422.066998
**TS_left_*Si*_b**	-3423.377381	-3422.455278	0.922103	-3422.985947	-3422.063844
**TS_left_*Re*_*a***	-3423.378829	-3422.45543	0.923404	-3422.98319	-3422.059786

**TS_left_*Re*_b**	-3423.381858	-3422.45724	0.924614	-3422.98794	-3422.063324
**TS_right_*Si***	-3423.382019	-3422.458094	0.923925	-3422.985732	-3422.061807
**TS_right_*Re***	-3423.371978	-3422.445711	0.926267	-3422.977318	-3422.051051
**TS_*exo*_left_*Si***	-3423.372017	-3422.44956	0.922457	-3422.975983	-3422.053526
**TS_*exo*_left_*Re***	-3423.369438	-3422.44638	0.923061	-3422.97311	-3422.050048
**TS_*exo*_right_*Re***	-3423.370934	-3422.44783	0.923106	-3422.98014	-3422.057036
**TS'_left_*Si*_a**	-3806.906855	-3805.885132	1.021722	-3806.247375	-3805.225653
**TS'_left_*Si*_b**	-3806.898329	-3805.878912	1.019417	-3806.245561	-3805.226100
**TS'_left_*Re*_a**	-3806.906375	-3805.883600	1.022750	-3806.245411	-3805.222700
**TS'_left_*Re*_b**	-3806.897676	-3805.877701	1.019975	-3806.245652	-3805.225677
**TS'_right_*Si***	-3806.907104	-3805.884056	1.023049	-3806.250601	-3805.2275515
**TS'_*exo*_left_*Si***	-3806.881300	-3805.862371	1.018929	-3806.233962	-3805.215033
**TS'_*exo*_left_*Re***	-3806.878027	-3805.852878	1.025149	-3806.221268	-3805.196119
**TS'_*exo*_right_*Si***	-3806.887968	-3805.868371	1.019597	-3806.237531	-3805.217934
**TS'_*exo*_right_*Re***	-3806.882216	-3805.862603	1.019613	-3806.235927	-3805.216314

7.5 Cartesian coordinates for the optimized structures

TS_left_*Si*_a

C	2.91365600	-3.67530500	3.65156100
C	2.22480900	-4.70503900	2.74975400
C	0.72317100	-4.41809500	2.65597900
C	0.45775400	-3.00576400	2.11438300
C	1.16991100	-1.93840500	2.97267900
C	2.66275200	-2.25282800	3.14352200
N	-0.96806900	-2.68722900	2.08987200
N	1.05128000	-0.58879400	2.40667400
C	-1.83768600	-3.22780400	1.21379900
O	-1.58286900	-4.18940100	0.48412600
C	-0.01500000	0.21506700	2.57917700
O	-1.05775000	-0.13690700	3.16013900
H	-1.24469500	-1.85038500	2.60454400
H	1.89171400	-0.19684300	1.96280100
C	-3.18815600	-2.51594000	1.16434800
P	-3.25212700	-1.31298200	-0.25896700
C	0.11474800	1.60543600	1.98564900
P	-1.09566900	1.86138700	0.57214900
H	2.53213100	-3.77666100	4.67780200
H	3.99378700	-3.85723000	3.69268600
H	2.67395000	-4.66634800	1.74686000
H	2.39049700	-5.72000400	3.12955200
H	0.21683400	-5.13262700	2.00236200
H	0.26416700	-4.50233700	3.65140500
H	0.83742200	-2.94778700	1.08502000

H	0.68175600	-1.92696600	3.95650100
H	3.10342300	-1.50941300	3.81764500
H	3.16558300	-2.12872700	2.17853300
C	-4.76160200	3.96180300	2.48708500
C	-4.10360600	4.56043400	1.41304300
C	-2.98065900	3.95197400	0.84976400
C	-2.50434500	2.73781800	1.36209900
C	-3.16935800	2.13835100	2.44396400
C	-4.28895500	2.75213700	3.00084400
H	-5.63789100	4.43428100	2.92211000
H	-4.45960200	5.50572400	1.01213000
H	-2.47257300	4.42672000	0.01701600
H	-2.79392000	1.20647700	2.85414500
H	-4.79740500	2.28019000	3.83674400
C	1.00580000	5.05156100	-2.05325800
C	1.33321400	4.97235600	-0.69961500
C	0.68738700	4.04928700	0.12309700
C	-0.27387200	3.17709100	-0.40513100
C	-0.59618800	3.26512100	-1.76838800
C	0.03087100	4.20475000	-2.58571700
H	1.51936400	5.76234700	-2.69391300
H	2.10690300	5.60848500	-0.28439100
H	0.96058000	4.00253600	1.17155900
H	-1.31841200	2.57015100	-2.18890700
H	-0.22408500	4.25874200	-3.64043700
C	-4.87205700	-3.69704600	-3.89550600
C	-5.05266900	-2.31417400	-3.80816300
C	-4.58466100	-1.61627800	-2.69758800
C	-3.93073800	-2.29318400	-1.65644600
C	-3.74919500	-3.68004000	-1.74750500
C	-4.22155100	-4.37323400	-2.86446500
H	-5.23692800	-4.24093700	-4.76227500
H	-5.56325400	-1.77869000	-4.60391200
H	-4.74156800	-0.54359500	-2.63477500
H	-3.21599600	-4.21291900	-0.96633600
H	-4.07393400	-5.44802900	-2.92501300
C	-6.81658900	1.49349400	0.69318600
C	-6.78297200	0.22047300	1.26466000
C	-5.71904700	-0.64053300	0.99614700
C	-4.66687400	-0.22741200	0.16781800
C	-4.70579900	1.05678600	-0.39323700
C	-5.77997400	1.90775900	-0.14316900
H	-7.64468300	2.16350700	0.90569300
H	-7.58884500	-0.10631000	1.91592600

H	-5.71711100	-1.63505700	1.43128900
H	-3.87790700	1.39049800	-1.01188300
H	-5.78839400	2.90425200	-0.57195400
Pd	-1.27186000	-0.17598800	-0.73216000
C	1.34163400	0.22534200	-1.10567900
C	0.52154400	-0.35654800	-2.12368500
C	4.18678800	-0.48709400	-3.54835900
H	4.94158700	-1.27741300	-3.44608300
H	3.26393300	-0.96841700	-3.89091300
H	4.52307700	0.22322800	-4.30777300
C	4.53661300	-2.56034000	-0.46442400
C	5.37932300	-2.11972100	0.56736500
C	4.89831600	-3.70421000	-1.18329100
C	6.54838100	-2.81463500	0.87295800
H	5.11348400	-1.23020000	1.13038200
C	6.07151200	-4.39971900	-0.88167600
H	4.25158400	-4.05483800	-1.98433800
C	6.89963300	-3.95722800	0.14927000
H	7.18930500	-2.46099600	1.67611600
H	6.33644900	-5.28526600	-1.45309500
H	7.81281300	-4.49571200	0.38682400
C	3.24385100	-1.83104400	-0.77437400
H	2.74965100	-2.30784000	-1.62771300
H	2.55265700	-1.95709100	0.06700300
C	-0.22739700	-2.85021200	-2.09233400
H	-1.03806100	-3.47846700	-1.74555900
H	0.59926500	-3.35112300	-2.59882500
C	3.61596400	0.64753900	-0.03237300
O	3.29770500	0.63228000	1.18316600
N	4.12587600	1.74374100	-0.72264700
N	4.36653000	1.45026000	-2.06940100
C	3.98524700	0.20881200	-2.24229900
C	4.33063700	3.06127700	-0.26725500
C	4.71491300	4.04658100	-1.18993900
C	4.14976500	3.40484500	1.08256900
C	4.91385900	5.35674900	-0.76419100
H	4.85134600	3.76500600	-2.22548800
C	4.34458600	4.72525600	1.48693500
H	3.86933900	2.63857700	1.79065900
C	-0.19364500	-1.54585200	-1.88004900
H	1.53478000	1.28949300	-1.18568100
H	1.26293800	-0.17007600	-0.10386100
C	3.39589600	-0.36020100	-1.05366900
C	4.72678900	5.71033600	0.57460100

H	5.21585100	6.10816600	-1.48898900
H	4.20394400	4.97982700	2.53421400
H	4.88402000	6.73395500	0.90211000
H	-3.35809000	-1.92474800	2.06870300
H	-3.98567100	-3.25707200	1.06659000
H	1.12300100	1.77026000	1.59878700
H	-0.09334000	2.33985500	2.76896100
C	0.45364500	0.30052400	-3.48829400
H	0.65527500	1.37367900	-3.41978900
H	1.20216100	-0.13717200	-4.16075900
H	-0.52797100	0.15062300	-3.94636400

TS_left_*Si*_b

C	2.35009000	-4.14427700	3.85618900
C	1.91817600	-5.02836000	2.68109200
C	0.50472400	-4.66986800	2.21457700
C	0.39392400	-3.18546200	1.83363300
C	0.84793000	-2.28924400	3.00161000
C	2.24886900	-2.65740600	3.49799800
N	-0.98540000	-2.82740200	1.50250300
N	0.80584000	-0.86390900	2.64944800
C	-1.59177600	-3.17726800	0.35207800
O	-1.07920900	-3.89106000	-0.51286000
C	-0.31201700	-0.13319400	2.82314400
O	-1.38206500	-0.62566100	3.22085600
H	-1.44859200	-2.18629200	2.14297900
H	1.67028600	-0.45585400	2.28188800
C	-2.99652400	-2.59680600	0.18520600
P	-2.98821300	-1.11797600	-0.94510500
C	-0.23726800	1.34894400	2.51273200
P	-1.42858600	1.80976100	1.13246400
H	1.71530600	-4.35699000	4.72904500
H	3.38087700	-4.37588500	4.14928800
H	2.62005100	-4.89703000	1.85140100
H	1.95868000	-6.08670700	2.96654400
H	0.20912100	-5.26741400	1.34758400
H	-0.21999100	-4.87348800	3.01622000
H	1.02287600	-2.98812100	0.95715900
H	0.12935200	-2.42291000	3.81919900
H	2.49018500	-2.02969400	4.36423900
H	2.97805200	-2.41945800	2.71975400
C	-5.60620600	2.60644400	2.98275600
C	-5.06718100	1.31796700	2.97038200
C	-3.79709800	1.09308200	2.44824800

C	-3.03863500	2.16187800	1.94371800
C	-3.58465600	3.45283600	1.95852400
C	-4.86268600	3.67150900	2.47671400
H	-6.60286100	2.77682900	3.38008700
H	-5.64407400	0.48183200	3.35388200
H	-3.37668600	0.09612100	2.44718900
H	-3.01933500	4.28942600	1.56338000
H	-5.27375200	4.67733900	2.48319200
C	0.15733000	5.97232500	-0.17292300
C	-0.49486100	5.13078600	-1.07672400
C	-0.97285700	3.89100800	-0.65392600
C	-0.81841700	3.48084900	0.67835500
C	-0.17783600	4.34043700	1.58229300
C	0.31452900	5.57523900	1.15626200
H	0.54426700	6.93170100	-0.50358900
H	-0.61741900	5.43209300	-2.11304300
H	-1.44260600	3.21566200	-1.36351300
H	-0.05928500	4.05165300	2.62175900
H	0.81998700	6.22657900	1.86343800
C	-3.67196600	-2.73115800	-5.23568000
C	-4.04272600	-1.42814000	-4.89258900
C	-3.85279500	-0.96679100	-3.59259600
C	-3.29133300	-1.80526100	-2.61745200
C	-2.91612400	-3.11057700	-2.96422600
C	-3.11031100	-3.56576200	-4.27051300
H	-3.81960800	-3.09028200	-6.25021700
H	-4.48417800	-0.77190800	-5.63754300
H	-4.15366200	0.04394000	-3.33222100
H	-2.44445700	-3.75652600	-2.22960900
H	-2.81381700	-4.57809300	-4.53063900
C	-6.97446000	1.06809400	-0.04176000
C	-6.90391200	-0.31707400	0.12046200
C	-5.71596700	-0.99449000	-0.15196300
C	-4.58065300	-0.28899900	-0.57293100
C	-4.66167400	1.10106100	-0.73082100
C	-5.85382100	1.77595800	-0.47609700
H	-7.89908600	1.59442000	0.17690300
H	-7.77607300	-0.87149100	0.45551700
H	-5.67817600	-2.07367400	-0.03854300
H	-3.77473500	1.65092500	-1.03172100
H	-5.89636100	2.85511700	-0.58390700
Pd	-1.13971000	0.27895200	-0.70368600
C	1.27347700	1.10308400	-0.62644000
C	0.74808700	0.74440700	-1.89429000

C	3.67502100	2.21619900	1.79854500
H	4.57673900	2.82234000	1.65017800
H	2.81637900	2.89195400	1.67163200
H	3.67370700	1.83277800	2.82322500
C	5.37050800	2.72831500	-1.40585900
C	6.30435200	1.81270000	-1.91253800
C	5.83264000	3.92034300	-0.84099200
C	7.66820200	2.08951200	-1.85394500
H	5.94647800	0.88313100	-2.34757000
C	7.20083700	4.19877600	-0.77798300
H	5.11424300	4.63781500	-0.44956900
C	8.12230600	3.28341300	-1.28457800
H	8.38091500	1.37234000	-2.25249200
H	7.54366500	5.12999900	-0.33430800
H	9.18679200	3.49640300	-1.23790100
C	3.89165900	2.39220400	-1.44568100
H	3.30896100	3.27008500	-1.13442600
H	3.59806500	2.16007300	-2.47572900
C	0.40135500	-1.72249200	-2.64760500
H	-0.33862100	-2.51257800	-2.60706000
H	1.35015000	-1.92907300	-3.14099200
C	3.57638500	-0.15825500	-1.10169700
O	3.59240900	-0.58899700	-2.25916600
N	3.48333300	-0.94815200	0.07793200
N	3.55664500	-0.16817100	1.23750900
C	3.63481800	1.08715300	0.81961000
C	3.85068800	-2.30852700	0.13473200
C	4.82624600	-2.74067400	1.04272100
C	3.26234500	-3.22006900	-0.74945500
C	5.20819300	-4.08083900	1.06327700
H	5.27357800	-2.01540200	1.71309100
C	3.66515700	-4.55468200	-0.73148200
H	2.49738200	-2.87450200	-1.43111400
C	0.21943400	-0.54343000	-2.08250100
H	1.29935000	2.14720700	-0.34316000
H	1.32609400	0.35811200	0.15466300
C	3.55700400	1.19824200	-0.59903000
C	4.63820900	-4.99206200	0.17023300
H	5.96405200	-4.41078100	1.77089400
H	3.20354300	-5.25774100	-1.41893000
H	4.94203600	-6.03479900	0.18462100
C	0.71678200	1.74745500	-3.02663900
H	-0.18079800	1.62741800	-3.63962900
H	0.76357400	2.77522500	-2.65319500

H	1.58845100	1.57602000	-3.66904500
H	-0.50084500	1.89696300	3.42242700
H	0.76243800	1.65272200	2.19341600
H	-3.65558000	-3.37291000	-0.21317300
H	-3.40147900	-2.24828800	1.13979400

TS_left_*Re*_a

C	-2.37759800	3.91140700	3.79255700
C	-1.53035700	4.88290800	2.96227400
C	-0.09049000	4.37488500	2.84482600
C	-0.04625200	2.96887600	2.23076000
C	-0.90540700	1.97949300	3.04274200
C	-2.33725100	2.49830600	3.20065900
N	1.31645500	2.44546200	2.17037000
N	-0.94432500	0.64268200	2.42751200
C	2.22171300	2.82438200	1.24684300
O	2.08793100	3.77958300	0.47985500
C	-0.00006700	-0.27926300	2.67538800
O	1.00546400	-0.04654200	3.37496400
H	1.49334400	1.60765700	2.72174000
H	-1.77349800	0.42313900	1.85866400
C	3.42864100	1.89397500	1.14802100
P	3.22290300	0.94943100	-0.44028900
C	-0.18324700	-1.65408500	2.05450600
P	1.06009800	-1.99306700	0.68397400
H	-2.00235700	3.89241500	4.82617000
H	-3.41850800	4.25339300	3.84251400
H	-1.96829900	4.97970800	1.96021000
H	-1.54084100	5.88277500	3.41266700
H	0.51919000	5.03632600	2.22260700
H	0.37751000	4.33648500	3.83897100
H	-0.44080700	3.01159500	1.20824200
H	-0.44021800	1.86059100	4.02936400
H	-2.90346800	1.79528400	3.82255100
H	-2.80918900	2.49533400	2.21574600
C	4.75389100	-3.97577600	2.67141000
C	4.04652100	-2.99235300	3.36237100
C	2.92594100	-2.38903900	2.78871400
C	2.49832800	-2.77995100	1.51139700
C	3.22231400	-3.75838600	0.81304600
C	4.34126900	-4.35161700	1.39187400
H	5.62362800	-4.44455100	3.12337500
H	4.36212200	-2.69155200	4.35781300
H	2.38204500	-1.61850900	3.32530700

H	2.90775600	-4.06521100	-0.17912800
H	4.89113600	-5.10897100	0.84074100
C	-0.97930100	-5.48291600	-1.59895800
C	-0.15695300	-4.57178500	-2.26541500
C	0.46074000	-3.54232700	-1.55534400
C	0.27803200	-3.42167100	-0.16986900
C	-0.52761000	-4.35630200	0.49380700
C	-1.16238000	-5.37392400	-0.21918000
H	-1.47727600	-6.27346300	-2.15268200
H	-0.01015700	-4.65220400	-3.33874200
H	1.06581500	-2.80372400	-2.07567600
H	-0.67324000	-4.28963800	1.56726800
H	-1.80281300	-6.07861800	0.30294500
C	4.90606100	3.79331700	-3.68248900
C	3.58014500	3.36687700	-3.74581600
C	3.07209600	2.50938500	-2.76810300
C	3.88972500	2.07910100	-1.71943600
C	5.22610000	2.50255900	-1.66484300
C	5.73041600	3.35829000	-2.64044900
H	5.30044200	4.46117800	-4.44322100
H	2.93610300	3.70293300	-4.55350200
H	2.03909100	2.18262800	-2.80540000
H	5.87532800	2.15288000	-0.86655500
H	6.76573200	3.68384200	-2.59185600
C	6.44147600	-2.38571300	-0.49414300
C	6.29963400	-1.61117600	0.65527100
C	5.34062800	-0.59767600	0.70846300
C	4.51275600	-0.35108600	-0.39191200
C	4.64900400	-1.14927100	-1.54008800
C	5.61025200	-2.15445900	-1.59323600
H	7.18866300	-3.17313700	-0.53162300
H	6.92841500	-1.79826500	1.52019900
H	5.24709000	-0.00719800	1.61297300
H	4.00510900	-0.96878500	-2.39717900
H	5.71114100	-2.75823800	-2.49083100
Pd	1.12912900	-0.01899400	-0.71987900
C	-1.34683900	-0.69500500	-1.22883500
C	-0.80435500	0.26806600	-2.10738900
C	-4.16118400	-1.28018800	-3.67014400
H	-3.26175200	-1.88007300	-3.86735800
H	-4.99650400	-1.98222100	-3.55501600
H	-4.35406700	-0.64409400	-4.53796500
C	-5.08917500	-2.76645900	-0.19969500
C	-5.72885200	-3.80290800	-0.88507300

C	-5.75920700	-2.14112600	0.86203100
C	-7.01465000	-4.21283600	-0.52286300
H	-5.21479300	-4.29380000	-1.70880900
C	-7.04023400	-2.55017000	1.22585000
H	-5.26698700	-1.32775100	1.38871700
C	-7.67371400	-3.58760700	0.53483600
H	-7.49877900	-5.01924600	-1.06763700
H	-7.54870100	-2.05775700	2.05069400
H	-8.67364000	-3.90349200	0.81941900
C	-3.70179100	-2.30168000	-0.59866700
H	-3.04412400	-2.35193000	0.28017500
H	-3.28309600	-2.99873600	-1.33556900
C	-0.25078900	2.74663500	-1.47475000
H	0.56976400	3.31493500	-1.04770700
H	-1.17155000	3.28093700	-1.70997900
C	-3.67044700	0.27274400	-0.30407300
O	-3.40194500	0.38859600	0.92491700
N	-3.91301400	1.31834300	-1.19765200
N	-4.08832300	0.87294200	-2.50677300
C	-3.99511200	-0.44028300	-2.44516100
C	-3.98144500	2.69431100	-0.93002900
C	-3.68796700	3.61341600	-1.94820400
C	-4.36937200	3.15615200	0.33463700
C	-3.74496500	4.98046100	-1.68471100
H	-3.43365600	3.23602700	-2.93129300
C	-4.41359400	4.52698800	0.58514900
H	-4.63583400	2.43789200	1.09801700
C	-0.19045400	1.44049300	-1.65239500
H	-1.39021000	-1.73008200	-1.53143900
H	-1.45353300	-0.46680800	-0.18061100
H	-0.68729200	-0.00081100	-3.15667600
C	-3.67701300	-0.89931100	-1.13233900
C	-4.09422500	5.44744100	-0.41508100
H	-3.51188700	5.68498500	-2.47853200
H	-4.71400400	4.87546400	1.56966900
H	-4.13076200	6.51373600	-0.21258400
H	3.45516700	1.17465600	1.97135600
H	4.35605900	2.47257100	1.12917400
H	-0.06193500	-2.39927800	2.84625700
H	-1.17929200	-1.76678100	1.61863100

TS_left_*Re*_b

C	-2.34270400	1.74211400	5.43925400
C	-1.81022400	3.09388200	4.95000800

C	-0.37354200	2.95296500	4.43817600
C	-0.28018700	1.91693000	3.30811800
C	-0.85870000	0.55780200	3.74745200
C	-2.27196500	0.68631500	4.33029100
N	1.10337500	1.69682600	2.89315800
N	-0.87126100	-0.40855300	2.64457800
C	1.80550600	2.58380600	2.16192500
O	1.40118500	3.71421900	1.87769600
C	0.18659900	-1.19536500	2.36227300
O	1.26848200	-1.09845300	2.96796800
H	1.46764600	0.75418200	3.02705000
H	-1.74501900	-0.48489500	2.12143500
P	3.08563800	1.52495500	-0.09635100
P	1.15149700	-1.87339700	-0.19668100
H	-1.74548600	1.41101200	6.30095900
H	-3.37720900	1.83491700	5.78992300
H	-2.45369700	3.47079100	4.14209700
H	-1.85116800	3.83632300	5.75552700
H	0.01591900	3.90343400	4.06381400
H	0.28203400	2.63071800	5.25943000
H	-0.84302700	2.28377300	2.44004800
H	-0.19070100	0.14809700	4.51490700
H	-2.58082300	-0.29501100	4.70918900
H	-2.97180200	0.93928800	3.52650400
C	5.13572000	-4.09336400	0.66392800
C	4.34505000	-4.48497400	-0.41544900
C	3.12626100	-3.84961500	-0.66104400
C	2.68672600	-2.81456600	0.17553000
C	3.48997600	-2.42233900	1.25828400
C	4.70159000	-3.06317500	1.50117400
H	6.08626700	-4.58498700	0.85129500
H	4.67216300	-5.28850600	-1.06983800
H	2.51977600	-4.16366100	-1.50333800
H	3.14598200	-1.63639100	1.91893300
H	5.31390000	-2.74915900	2.34140300
C	-0.81797400	-4.28115000	-3.63352000
C	-1.00981100	-4.70088600	-2.31699400
C	-0.41105000	-4.00900100	-1.26371900
C	0.37067700	-2.87477500	-1.51847000
C	0.55846400	-2.45902100	-2.84539600
C	-0.02499300	-3.16212600	-3.89807600
H	-1.29216400	-4.81741900	-4.45010800
H	-1.63378200	-5.56413800	-2.10491500
H	-0.57708100	-4.34767900	-0.24747300

H	1.14535400	-1.56592300	-3.04289100
H	0.12314700	-2.82685000	-4.92060800
C	4.19137300	5.25465900	-2.62442200
C	4.46881000	3.96707000	-3.09055000
C	4.15649900	2.85737800	-2.30884400
C	3.56206200	3.02143800	-1.04797200
C	3.28414200	4.31445200	-0.58284200
C	3.60163700	5.42204300	-1.37215300
H	4.43527600	6.11975900	-3.23457100
H	4.93471700	3.82638100	-4.06199600
H	4.39073700	1.86212100	-2.67492100
H	2.80085300	4.45608800	0.37903400
H	3.38175500	6.41967500	-1.00202800
C	6.80930100	-1.16773300	-0.69036500
C	6.84191900	-0.16705800	0.28278300
C	5.73265500	0.65478700	0.47970200
C	4.57241900	0.46977700	-0.28396600
C	4.54805800	-0.54093600	-1.25460500
C	5.66345100	-1.34911700	-1.46476700
H	7.67274700	-1.80979300	-0.83857400
H	7.73380500	-0.02269000	0.88610500
H	5.77683200	1.44192300	1.22622700
H	3.63862500	-0.70283500	-1.82597100
H	5.62365300	-2.14065200	-2.20593600
Pd	1.10579400	0.47801100	-0.75190100
C	-1.50492200	0.04796900	-1.15637400
C	-0.77251000	1.00623000	-1.92366400
C	-3.47911500	2.28894700	0.80552400
H	-2.53387300	2.71595400	0.44734900
H	-4.29248200	2.90684500	0.40830300
H	-3.49957600	2.34579600	1.89635500
C	-5.44876700	1.74927700	-2.20544400
C	-5.75452500	3.10884300	-2.09086500
C	-6.49959000	0.82258500	-2.26713600
C	-7.08208000	3.54109600	-2.03373100
H	-4.94586800	3.83550900	-2.04990400
C	-7.82398000	1.25167000	-2.21291300
H	-6.26589400	-0.23497200	-2.35652500
C	-8.12063400	2.61275200	-2.09391800
H	-7.30175500	4.60166700	-1.94353700
H	-8.62855500	0.52286300	-2.26349100
H	-9.15417000	2.94505500	-2.05000200
C	-4.01145700	1.26575300	-2.22931500
H	-3.84457200	0.64321300	-3.11663400

H	-3.33360200	2.12512000	-2.30377600
C	-0.18096000	3.36939500	-0.99699800
H	0.60779300	3.88380400	-0.46180300
H	-1.06178200	3.95691500	-1.26114500
C	-3.90875900	-0.99480700	-0.95629900
O	-4.10361900	-1.80960100	-1.86460600
N	-3.75696700	-1.29174400	0.41424000
N	-3.60053200	-0.13181500	1.18917600
C	-3.62344400	0.87743600	0.34155700
C	-3.61735400	-2.54772500	1.02557400
C	-3.54225300	-2.63413200	2.42611700
C	-3.49068100	-3.71433200	0.25079700
C	-3.30331500	-3.86312400	3.03591100
H	-3.67014500	-1.73709100	3.01721400
C	-3.25918500	-4.93465700	0.88280000
H	-3.57067500	-3.64103000	-0.82459600
C	-0.11880000	2.08273200	-1.29473600
H	-1.64127800	-0.92996400	-1.60477800
H	-1.43315900	0.08419300	-0.07900600
C	-3.65899500	0.43946800	-1.02562400
C	-3.15161000	-5.02224400	2.27225500
H	-3.23925400	-3.91191200	4.11967200
H	-3.16133800	-5.83017400	0.27387400
H	-2.96554800	-5.97822700	2.75264200
C	-0.71165600	0.86498800	-3.43379900
H	-0.83729700	-0.17925600	-3.73584400
H	-1.51309300	1.44807000	-3.90310200
H	0.24039900	1.23635100	-3.82328600
C	0.01041900	-2.21543500	1.25668700
C	3.16070100	2.06698400	1.68589900
H	-1.01071100	-2.23245000	0.87208300
H	0.23514100	-3.20278500	1.67021400
H	3.47468400	1.18915500	2.25777200
H	3.90752500	2.85589400	1.80936500

TS_right_*Si*

C	-3.76298300	0.14505100	4.67938600
C	-3.87786900	1.54200300	4.05435800
C	-2.49305000	2.12289400	3.74482100
C	-1.68863000	1.17451100	2.84753000
C	-1.57592800	-0.22455900	3.47621500
C	-2.95935800	-0.80790400	3.78290900
N	-0.33787700	1.67473100	2.58374200
N	-0.78547300	-1.11944000	2.62070700

C	-0.05209700	2.45798000	1.52465700
O	-0.90422200	2.93719700	0.77408100
C	0.54315700	-1.21463700	2.80952700
O	1.12860000	-0.56317500	3.69456400
H	0.41358900	1.24464300	3.11526500
H	-1.25552500	-1.45597400	1.77610200
C	1.43797400	2.70781900	1.28001900
P	1.89203600	2.06249200	-0.39597500
C	1.32731300	-2.19455400	1.94879000
P	2.47533200	-1.43356000	0.68163600
H	-3.27405500	0.22544700	5.66180700
H	-4.76032300	-0.27412000	4.85855600
H	-4.46041100	1.48124400	3.12678500
H	-4.42405200	2.21590200	4.72536800
H	-2.57631000	3.09173300	3.24089800
H	-1.93504600	2.28664000	4.67835000
H	-2.19685800	1.06861300	1.88642600
H	-1.01279000	-0.13635300	4.41095600
H	-2.83188800	-1.78200100	4.27062000
H	-3.48566800	-0.97608900	2.83949200
C	6.22994200	0.59084500	2.50048500
C	5.09762700	0.44980300	3.30002500
C	3.95165700	-0.18182500	2.80807200
C	3.93637300	-0.67840200	1.49824200
C	5.08226300	-0.53245500	0.69589700
C	6.21930600	0.09314100	1.19547800
H	7.11690300	1.08427900	2.88795500
H	5.09982200	0.82482100	4.31997300
H	3.07688600	-0.29806200	3.43654500
H	5.08596600	-0.91096700	-0.32115200
H	7.09222600	0.20364100	0.56058200
C	4.49008100	-5.15269100	-1.21302200
C	3.84604900	-4.21661400	-2.02174000
C	3.22465000	-3.10625300	-1.44700500
C	3.24473900	-2.92587400	-0.05928800
C	3.90847500	-3.86304500	0.74693500
C	4.52171500	-4.97415900	0.17307200
H	4.96984300	-6.01907400	-1.65925500
H	3.81999600	-4.35104500	-3.09921300
H	2.70283100	-2.38334000	-2.06553700
H	3.96212000	-3.71528900	1.82187800
H	5.02869500	-5.69810500	0.80449700
C	0.24703800	4.93482000	-3.64269700
C	0.72508600	3.66345000	-3.96704600

C	1.20180300	2.82390200	-2.96229300
C	1.22088900	3.24828700	-1.62497100
C	0.73964200	4.52242000	-1.30778300
C	0.25338200	5.35866800	-2.31334000
H	-0.13719700	5.58774400	-4.42108500
H	0.71565400	3.32104700	-4.99807600
H	3.80810200	1.08247800	-2.26791300
H	6.21826600	1.49886000	-2.66328300
Pd	1.18381900	-0.17176000	-0.76607000
C	-1.13745800	0.45871400	-1.45043300
C	-0.55685700	-0.58199800	-2.19894700
C	-0.00929800	-1.70204800	-1.51982000
H	-1.28187900	1.43503800	-1.89708200
H	-1.24938600	0.38612700	-0.37717400
C	-0.09927500	-3.02059100	-1.43234200
H	-0.92867600	-3.56141400	-1.87997400
H	0.63151800	-3.61667100	-0.89546500
C	-3.53419700	0.23162600	-1.26351700
C	-3.88418300	-0.25709700	-2.55678900
N	-3.79679600	-1.57046900	-2.64512700
N	-3.43969300	-2.00322100	-1.36824600
C	-3.26821900	-0.93803900	-0.48014200
O	-2.87008500	-1.03517000	0.71566100
C	-3.77091700	1.61262200	-0.72231600
H	-3.59425700	2.35100300	-1.51448700
H	-3.03411100	1.84409900	0.05625600
H	-3.63272200	-3.87293500	-3.19127600
C	-2.88285700	-5.25076500	0.34368100
H	-2.99004500	-3.19009900	0.98698900
C	-2.96978700	-6.14073800	-0.72795000
H	-3.33044200	-6.31621200	-2.84748300
H	-2.68231000	-5.62433200	1.34483100
H	-2.83243700	-7.20699000	-0.57286600
C	-4.28589200	0.55995600	-3.74312200
H	-4.47548500	-0.09197300	-4.60009000
H	-5.19517000	1.13617600	-3.53148000
H	-3.50386700	1.28072800	-4.01611300
C	-5.16912600	1.82161700	-0.16533500
C	-5.94648500	2.91142700	-0.56896200
C	-5.70429900	0.93027600	0.77667800
C	-7.22503100	3.11680700	-0.04451200
H	-5.54382100	3.60864500	-1.30049500
C	-6.97828000	1.13427500	1.30467900
H	-5.11470300	0.07455900	1.08986600

C	-7.74518000	2.22899100	0.89648900
H	-7.81364400	3.96902600	-0.37458200
H	-7.37543000	0.43412800	2.03523300
H	-8.73961400	2.38457400	1.30591600
C	-3.30991200	-3.37932600	-1.12936900
C	-3.41955000	-4.27252200	-2.20857100
C	-3.05287000	-3.87997500	0.15865000
C	-3.24695200	-5.63879400	-2.00150500
H	1.54444600	1.82354800	-3.21232200
H	0.70084700	4.85422700	-0.27789300
H	-0.13113900	6.34064200	-2.05319700
C	6.43621900	2.78761800	-0.94570100
C	5.79031700	3.37277300	0.14303700
C	4.42546400	3.16364400	0.34733300
C	3.69784100	2.35221400	-0.52947500
C	4.36181400	1.74244800	-1.60527200
C	5.71845200	1.96935600	-1.82119100
H	7.49651300	2.96034800	-1.10691900
H	6.34698200	3.99795700	0.83519100
H	3.93834500	3.63450200	1.19465600
C	-0.46545000	-0.47740200	-3.70633200
H	0.42687100	-0.98047500	-4.08975500
H	-1.34561000	-0.96479400	-4.14045700
H	-0.45569900	0.56801400	-4.03185300
H	1.64207400	3.78018800	1.36154500
H	2.07453600	2.18006700	1.99796800
H	0.66072400	-2.82439000	1.35204800
H	1.90149300	-2.83119900	2.62918400

TS_right_*Re*

C	-3.43137500	-2.86395500	3.40543300
C	-3.70320400	-1.37548700	3.66423800
C	-2.40011100	-0.60842700	3.93706100
C	-1.43560600	-0.80056400	2.75741700
C	-1.10969200	-2.29905000	2.62422000
C	-2.39370300	-3.06154200	2.29111800
N	-0.20383100	-0.00795000	2.84447100
N	-0.05004300	-2.58667600	1.64389700
C	-0.22633000	1.31857300	2.59508300
O	-1.26642700	1.97637500	2.58357600
C	1.24350300	-2.59426000	2.02242000
O	1.59100100	-2.30004800	3.17678200
H	0.66671500	-0.49775200	3.01158700
H	-0.30044100	-2.75368800	0.67560000

C	1.10431700	1.97139800	2.21707500
P	1.37402200	1.90680500	0.37368300
C	2.29385000	-2.94041200	0.98024500
P	2.94556600	-1.43181600	0.09017900
H	-3.06754500	-3.33974400	4.32879600
H	-4.35657200	-3.37754900	3.12275500
H	-4.18382600	-0.94178000	2.78025400
H	-4.39113400	-1.25772800	4.51047500
H	-2.59651400	0.45949500	4.05548400
H	-1.92162600	-0.96665800	4.86080200
H	-1.95982700	-0.47531800	1.85272700
H	-0.70819500	-2.64954300	3.58073700
H	-2.16711800	-4.12475900	2.16018300
H	-2.80461400	-2.68559900	1.34848600
C	5.98161000	0.87548000	2.71605000
C	4.90518000	0.22871700	3.32358300
C	4.00588400	-0.51967600	2.56210500
C	4.18406300	-0.62929100	1.17422000
C	5.27869700	0.01083500	0.57139000
C	6.16957400	0.75726500	1.33844600
H	6.67386600	1.46305400	3.31217300
H	4.76292900	0.29719200	4.39847800
H	3.19314500	-1.04563000	3.05003800
H	5.43346700	-0.06876900	-0.49840600
H	7.00280000	1.25650300	0.85464700
C	5.55717500	-3.28317900	-3.25193700
C	4.52544700	-2.40169500	-3.57452300
C	3.73744700	-1.84920600	-2.56325200
C	3.97982200	-2.17350700	-1.22339900
C	5.02727200	-3.05209300	-0.90439500
C	5.80783000	-3.60795500	-1.91532900
H	6.16829300	-3.71616500	-4.03857100
H	4.32911100	-2.14647600	-4.61181400
H	2.92088600	-1.17635200	-2.80325300
H	5.24111000	-3.28994400	0.13377900
H	6.61362100	-4.29078000	-1.66229500
C	-1.48196100	4.75032100	-1.90157800
C	-0.36125100	4.19222700	-2.52551300
C	0.51524900	3.39173400	-1.79908800
C	0.28770200	3.14813000	-0.43248300
C	-0.82251300	3.72652100	0.19236900
C	-1.70483100	4.52039700	-0.54676300
H	-2.18436900	5.34767900	-2.47495300
H	-0.17639600	4.37168600	-3.58124900

H	1.37158500	2.94858800	-2.29892700
H	-1.04464500	3.51478300	1.23034100
H	-2.58188400	4.92845300	-0.05648600
C	5.56122200	3.72060300	-0.44097000
C	4.91637500	4.01901100	0.76043400
C	3.66419400	3.47193600	1.04168800
C	3.05275700	2.60250500	0.13099400
C	3.72215000	2.28419100	-1.06055500
C	4.96059600	2.85183000	-1.35338500
H	6.53035800	4.15873400	-0.66188400
H	5.38396900	4.68710100	1.47794600
H	3.16849500	3.73002100	1.97163800
H	3.27053400	1.57596700	-1.75067900
H	5.46241100	2.60349100	-2.28448300
Pd	1.12638400	-0.22158600	-0.67824900
C	-1.27193100	0.13300600	-1.35783600
C	-0.44366500	-0.48375700	-2.30295600
C	0.29379700	-1.64733600	-1.94388700
H	-1.59257400	1.15882200	-1.49640200
H	-1.53604600	-0.35550700	-0.42734900
C	0.43421600	-2.93107400	-2.24772500
H	-0.26771800	-3.43977400	-2.90749300
H	1.25966400	-3.53006300	-1.87403500
C	-3.68048300	-0.62488100	-1.97953300
C	-4.05191000	0.36469800	-2.93379100
N	-4.36175300	1.52099500	-2.37628700
N	-4.22727300	1.32155200	-1.01332600
C	-3.84524200	-0.00288100	-0.69397200
O	-3.64617800	-0.43897900	0.46564700
C	-3.38478100	-2.06168500	-2.29217600
H	-2.31160700	-2.20538200	-2.48964100
H	-3.87643200	-2.30672300	-3.24633900
C	-3.81107600	-3.10512900	-1.27623400
C	-5.00387500	-2.98824500	-0.55206600
C	-3.03342500	-4.25523400	-1.09505000
C	-5.40597500	-3.99520600	0.32330600
H	-5.60719600	-2.09472100	-0.66730200
C	-3.43659300	-5.27145600	-0.22649200
H	-2.10128400	-4.35693800	-1.64594100
C	-4.62663700	-5.14337800	0.48899400
H	-6.33098400	-3.88171300	0.88232400
H	-2.81801800	-6.15719600	-0.10469300
H	-4.94288600	-5.92610400	1.17310900
C	-4.46223400	2.40524500	-0.14897700

C	-5.13581400	3.53679500	-0.63727000
C	-3.99596400	2.39239300	1.17290100
C	-5.33367800	4.63870100	0.19077000
H	-5.47835100	3.53475600	-1.66411400
C	-4.19632800	3.50646300	1.98559300
H	-3.45885400	1.53422200	1.54250800
C	-4.86551800	4.63537300	1.50844900
H	-5.85792500	5.50786800	-0.19921400
H	-3.80187100	3.48618600	2.99750000
H	-5.01903500	5.49917200	2.14959500
C	-4.12567800	0.21773300	-4.42231300
H	-4.44125900	1.16526900	-4.86738500
H	-3.15911700	-0.06558600	-4.85840100
H	-4.84613900	-0.55784800	-4.71452500
C	-0.22658000	0.15577200	-3.65698100
H	-0.94781800	-0.26384200	-4.36900400
H	-0.39166100	1.23544900	-3.60643100
H	0.77828500	-0.04050400	-4.04204900
H	3.10790500	-3.46753000	1.48353500
H	1.88608600	-3.57878600	0.19225800
H	1.96636000	1.45140800	2.64977900
H	1.09370100	3.00669800	2.56698400

TS_*exo*_left_*Si*

C	-0.13746800	-2.28357600	-1.48688300
C	-0.61180200	-3.10285800	-2.40545600
C	0.86433600	-1.81246300	-0.65122900
H	-1.66785100	-3.14113600	-2.65472800
H	0.05113200	-3.77844800	-2.94758500
C	1.51191300	-0.54664100	-0.89832000
H	1.37525300	-0.07193900	-1.86194000
H	1.61447900	0.12012400	-0.06150600
C	-3.27016000	2.70787800	0.82180000
C	-4.41921900	3.48884600	0.89891800
C	-4.65182900	4.49239600	-0.04443300
C	-3.72226000	4.71690100	-1.05910600
C	-2.56337700	3.94131600	-1.13494800
C	-2.32627100	2.93143700	-0.19336200
C	-4.60503600	-2.87579600	-2.09434000
C	-4.93612800	-4.08008200	-2.71543000
C	-4.55981900	-5.29391600	-2.13747900
C	-3.84739800	-5.29599800	-0.93660200
C	-3.50323900	-4.09648300	-0.31343600
C	-3.88969400	-2.87518900	-0.88672300

H	-5.14270500	3.30286500	1.68715600
H	-5.55594200	5.09231200	0.00980900
H	-3.89478400	5.49733000	-1.79537300
H	-1.84733200	4.12306700	-1.92896700
H	-5.49438700	-4.06825900	-3.64761600
H	-4.81994400	-6.23158800	-2.62065000
H	-3.55031400	-6.23708200	-0.48184100
H	-2.93018300	-4.10019100	0.60942400
P	-3.31474800	-1.28653800	-0.17651200
P	-0.87857200	1.80801400	-0.31837900
C	-4.67930500	-0.13976000	-0.60314600
C	-5.93081700	-0.18583000	0.02707700
C	-4.45577100	0.81120400	-1.60745300
C	-6.93233000	0.71553400	-0.33142900
H	-6.12762300	-0.92818100	0.79508100
C	-5.46256500	1.70108700	-1.97839100
C	-6.69951000	1.65862800	-1.33527100
H	-7.89633400	0.67871100	0.16836000
H	-5.26927000	2.44431300	-2.74510900
H	-7.47993000	2.36240400	-1.61043400
C	-3.50678700	-1.47614100	1.68077000
C	0.25033100	2.32118300	1.09071700
C	0.02213800	2.42234600	-1.79219100
C	-0.20413200	1.76588200	-3.01380500
C	0.95728200	3.46264200	-1.74227000
C	0.49232700	2.14243700	-4.16016600
H	-0.90753500	0.93702900	-3.04910700
C	1.66239500	3.83071200	-2.88933400
H	1.15963900	3.97718200	-0.80971800
C	1.43264100	3.17450200	-4.09769300
H	0.31299700	1.62186100	-5.09661200
H	2.40041700	4.62520400	-2.83283200
H	1.98952600	3.45850600	-4.98589400
Pd	-1.15339800	-0.55651700	-0.59892700
C	3.77038000	0.02761400	0.35024200
C	3.69273000	-0.71255600	-0.90416200
C	3.98234300	0.27827200	-1.91887900
N	3.96148100	1.35626100	-0.04093600
O	3.53244100	-0.35124200	1.51856300
H	-4.91148700	-1.93650700	-2.54469900
H	-3.08668600	1.93570600	1.55817700
C	3.99409900	-2.18498000	-1.00195400
H	3.79620100	-2.62520100	-0.01917300
H	3.31167100	-2.67346500	-1.70968100

C	4.07546800	0.04189600	-3.39016900
H	4.91775000	-0.61802000	-3.62748700
H	3.16517100	-0.44579400	-3.76299100
H	4.20266000	0.99319300	-3.91270600
N	4.08415600	1.48843000	-1.43057400
C	3.86403900	2.51923300	0.74382800
C	3.80813700	3.76998400	0.10586000
C	3.79437800	2.45478300	2.14638500
C	3.63833500	4.92833500	0.85881300
H	3.89335500	3.80330400	-0.97151000
C	3.61605300	3.62675600	2.88122300
H	3.87860800	1.49694500	2.63824300
C	3.52620400	4.86754400	2.25026700
H	3.59338300	5.88810300	0.35048100
H	3.55257200	3.56131300	3.96411500
H	3.38495200	5.77350000	2.83202200
C	-0.10155400	1.51766200	2.32329000
O	-1.18201800	1.70139000	2.90842900
N	0.78035800	0.57424800	2.71534400
H	1.67147500	0.44791200	2.23500800
C	0.56923300	-0.18658100	3.94804200
C	-0.28717500	-1.44950000	3.73722400
C	1.92413000	-0.55533000	4.56422000
H	0.02668200	0.47805600	4.63161000
C	-0.49009000	-2.18186300	5.07173000
H	0.24195000	-2.12164900	3.05922900
C	1.74878300	-1.32395800	5.87990300
H	2.49504900	-1.15623700	3.84500800
H	2.49920700	0.36378100	4.72752600
C	0.85943000	-2.56054700	5.69349200
H	-1.10129100	-3.07066400	4.88758000
H	-1.05303000	-1.53062400	5.75533500
H	2.73093700	-1.61204700	6.27253400
H	1.29209200	-0.66032500	6.62818600
H	0.70471800	-3.06760000	6.65319000
H	1.36871100	-3.27889700	5.03565800
N	-1.55860000	-1.10401200	3.10257800
C	-2.24050200	-1.99941300	2.35507300
O	-1.88053900	-3.17425200	2.22578300
H	-3.47866700	0.86307200	-2.07763100
H	-1.81612500	-0.11923200	3.10519500
C	5.42938800	-2.47121000	-1.40132700
C	6.48662500	-2.02429800	-0.59703900
C	5.72921200	-3.16598800	-2.57697200

C	7.80919700	-2.26852100	-0.96046100
H	6.26374300	-1.48165900	0.31821800
C	7.05422100	-3.41072400	-2.94681400
H	4.91652500	-3.51883600	-3.20807300
C	8.09847400	-2.96207200	-2.13913800
H	8.61694900	-1.91667700	-0.32436000
H	7.26757100	-3.95096200	-3.86536900
H	9.13000900	-3.14988200	-2.42373200
C	1.13468800	-2.57954000	0.62934300
H	0.20479300	-2.90699800	1.09853300
H	1.71856100	-3.47766700	0.39482500
H	1.72332300	-1.97526900	1.32266000
H	-4.32082000	-2.17454000	1.89760100
H	-3.78430400	-0.48809200	2.05933600
H	0.12015900	3.38256600	1.31724300
H	1.28157200	2.16012300	0.77245300

TS_*exo*_left_*Re*

C	-0.31371800	0.63453400	-2.50039900
C	-0.82848700	1.32442500	-3.50195500
C	0.74515600	-0.05439600	-1.92140700
H	-1.88527100	1.56621900	-3.55558500
H	-0.19599100	1.68048300	-4.31613700
C	1.44148100	0.46995600	-0.77962000
H	1.22231200	1.46987900	-0.43691900
H	1.70612900	-0.24750900	-0.01886300
C	-3.17199100	0.24850300	3.07047300
C	-4.25555700	0.50465600	3.90509000
C	-4.34391900	1.71891700	4.58941600
C	-3.33557800	2.67100900	4.44256500
C	-2.24094400	2.41282000	3.61483400
C	-2.14886100	1.19815400	2.92211200
C	-4.85265400	1.21328600	-3.02803800
C	-5.25888800	1.43811900	-4.34379400
C	-4.92625400	0.52590500	-5.34639200
C	-4.18182000	-0.61170800	-5.02659800
C	-3.76292500	-0.83963900	-3.71635800
C	-4.10469400	0.07124300	-2.70416000
H	-5.04263300	-0.23661600	4.00538900
H	-5.19805200	1.92312500	5.22900800
H	-3.39585300	3.61743200	4.97309100
H	-1.46170000	3.15943300	3.50612000
H	-5.84128600	2.32398200	-4.58206100
H	-5.24481700	0.70079400	-6.37022300

H	-3.91892000	-1.32585700	-5.80219200
H	-3.16879800	-1.71598900	-3.47315700
P	-3.43394300	-0.14035100	-1.01190600
P	-0.77820000	0.85479600	1.74637800
C	-4.69778200	0.67905500	0.02983300
C	-5.94318700	0.10004100	0.30983300
C	-4.39951100	1.94368400	0.55503200
C	-6.86458900	0.76961000	1.11461600
H	-6.19794800	-0.87175300	-0.10296200
C	-5.32706700	2.62133200	1.34395800
C	-6.55802500	2.03082400	1.63064200
H	-7.82412400	0.30985200	1.33441300
H	-5.07516500	3.59159300	1.75983400
H	-7.27565900	2.54851600	2.26068000
C	-3.66203200	-1.95994900	-0.62223800
C	0.36122900	-0.31821600	2.66863600
C	0.16123300	2.42967400	1.71289800
C	-0.28555400	3.42062800	0.81967800
C	1.32555600	2.66271200	2.45446700
C	0.40779400	4.62065000	0.68533000
H	-1.16439900	3.23130700	0.20885000
C	2.02744600	3.86154400	2.30778500
H	1.71435400	1.90850800	3.12666700
C	1.57039400	4.84284700	1.42970300
H	0.05318300	5.37351900	-0.01277000
H	2.94415700	4.01448100	2.86879800
H	2.12659400	5.76774100	1.30902200
Pd	-1.20853900	0.30634400	-0.55665200
C	3.83859400	0.35848700	0.25076900
C	3.53563800	1.04876200	-0.99308500
C	4.02229700	0.16780300	-2.02471000
N	4.30135700	-0.90139600	-0.16442800
O	3.60323500	0.70010600	1.42596000
H	-5.12634600	1.92259400	-2.25308700
H	-3.10603100	-0.69393600	2.54298400
C	3.40500200	2.54716200	-1.08066300
H	2.82429300	2.81733000	-1.97141400
H	2.83543700	2.89228600	-0.21121000
C	4.02672200	0.44991800	-3.49031700
H	3.01914100	0.71701300	-3.83615900
H	4.68479200	1.29621800	-3.72193500
H	4.37125800	-0.42760500	-4.04358100
N	4.42502100	-0.98857800	-1.55614200
C	4.56454800	-2.03907600	0.61330400

C	4.98593800	-3.22218600	-0.02011900
C	4.40860300	-2.02078100	2.01219900
C	5.25204000	-4.35920100	0.73692400
H	5.10249800	-3.22092200	-1.09589700
C	4.67992800	-3.17403000	2.75011600
H	4.11668500	-1.09885200	2.49549700
C	5.10234700	-4.34819300	2.12655300
H	5.58268900	-5.26351000	0.23277600
H	4.56444900	-3.14342100	3.83048800
H	5.31470000	-5.23851200	2.71105400
C	-0.09809700	-1.72380800	2.34451600
O	-1.21316100	-2.13489200	2.69693000
N	0.73529400	-2.46648500	1.58136800
H	1.68133200	-2.13915700	1.42158300
C	0.42231800	-3.84786400	1.20080800
C	-0.43416200	-3.92840100	-0.07650700
C	1.73124600	-4.62212100	1.02211600
H	-0.15342200	-4.28104800	2.02817500
C	-0.69456600	-5.39523100	-0.45596500
H	0.11528200	-3.45349400	-0.89378800
C	1.48760400	-6.07712300	0.61204400
H	2.33866200	-4.12555300	0.25408900
H	2.30920900	-4.57058800	1.94888900
C	0.62028800	-6.15855300	-0.64920800
H	-1.29766900	-5.41008000	-1.36806700
H	-1.28852600	-5.86535400	0.34059000
H	2.45203700	-6.57394100	0.45534500
H	0.98574800	-6.60924600	1.43288200
H	0.41472000	-7.20381700	-0.90833200
H	1.16919600	-5.72491000	-1.49708100
N	-1.68627900	-3.19326000	0.09911000
C	-2.41157800	-2.77027600	-0.96016000
O	-2.10118900	-3.02198700	-2.12871100
H	-3.42561000	2.38103700	0.35895900
H	-1.90421600	-2.87485700	1.04037400
C	4.74425000	3.25546600	-1.10452600
C	5.21415100	3.88750400	-2.25963900
C	5.54809400	3.26399600	0.04500400
C	6.46318400	4.51334200	-2.27515300
H	4.59415800	3.89324900	-3.15346400
C	6.79312300	3.88934400	0.03254200
H	5.18831600	2.76593700	0.94141000
C	7.25643000	4.51581600	-1.12833300
H	6.81348600	4.99868300	-3.18236800

H	7.40612800	3.88789800	0.93009500
H	8.22839100	5.00142000	-1.13677000
C	1.06065600	-1.45468600	-2.40916800
H	0.15015900	-2.01429700	-2.63827700
H	1.66971400	-1.39674400	-3.31898400
H	1.65295400	-1.99855500	-1.66743800
H	-3.90318800	-2.02157900	0.44302400
H	-4.49997400	-2.36484000	-1.19732400
H	0.28891400	-0.14858100	3.74642000
H	1.38551300	-0.12341400	2.33395900

TS_*exo*_right_*Re*

C	-3.57929700	4.45168200	4.12036500
C	-3.22936500	3.26859800	5.03260100
C	-3.69127600	1.93909400	4.42260400
C	-3.09324000	1.75172700	3.02143100
C	-3.51401700	2.91870000	2.10913200
C	-3.03295600	4.24903200	2.70026300
N	-3.44498200	0.46529300	2.42102100
N	-3.04934700	2.76800200	0.73174800
C	-2.74524900	-0.64075200	2.76557800
O	-1.94629500	-0.64424800	3.70405500
C	-3.78951800	2.09247600	-0.17468500
O	-4.88149900	1.58250500	0.11698800
H	-4.10914800	0.46587100	1.65384500
H	-2.08692500	3.00336600	0.50400000
C	-2.90473400	-1.87221800	1.87504600
P	-1.31833700	-2.03427200	0.91175700
C	-3.19990700	1.95622800	-1.56439600
P	-2.14865000	0.41121000	-1.70830000
H	-4.67195800	4.55912600	4.06911700
H	-3.19009900	5.38804400	4.53642400
H	-2.14122000	3.23465200	5.18416400
H	-3.67908800	3.40666700	6.02256200
H	-3.38312000	1.09503200	5.04507800
H	-4.78757100	1.91599700	4.34847800
H	-2.00221700	1.75291100	3.11623600
H	-4.60708900	2.92804200	2.04856100
H	-3.34633000	5.06692800	2.04181200
H	-1.93355300	4.25899900	2.72240800
C	-5.04145400	-3.04465000	-2.80024300
C	-5.40588500	-2.11433900	-1.82639500
C	-4.56165800	-1.04777900	-1.51716500
C	-3.33702800	-0.90001800	-2.18881600

C	-2.98037700	-1.83493900	-3.17105800
C	-3.82929200	-2.89779000	-3.47392400
H	-5.69841900	-3.87729000	-3.03473200
H	-6.35607100	-2.20985900	-1.30809300
H	-4.87711400	-0.30979800	-0.78839900
H	-2.03880300	-1.73568800	-3.69824200
H	-3.53382700	-3.61788100	-4.23030600
C	0.33517900	1.00145300	-5.56111800
C	0.96233100	0.63576800	-4.37104100
C	0.21586000	0.49159800	-3.19958000
C	-1.17162400	0.68827100	-3.22514700
C	-1.79997600	1.05157400	-4.42847400
C	-1.04711000	1.21682200	-5.58803800
H	0.91873300	1.11984600	-6.46980100
H	2.03248600	0.46302600	-4.33124300
H	0.73674300	0.24962700	-2.27871800
H	-2.87748700	1.18753600	-4.46213400
H	-1.53708000	1.50408300	-6.51401700
C	1.88723100	-4.16743400	3.50523500
C	2.09806200	-3.91465400	2.14801800
C	1.11058700	-3.28337700	1.39582600
C	-0.10278500	-2.89569800	1.98849100
C	-0.30464600	-3.14267600	3.35198200
C	0.68802500	-3.77821200	4.10097500
H	2.65784200	-4.65604400	4.09446300
H	3.03478800	-4.19485000	1.67570500
H	1.29374900	-3.08879900	0.34414800
H	-1.20319700	-2.79369100	3.84274500
H	0.52054100	-3.96014900	5.15883900
C	-1.74734700	-5.35648200	-2.27149700
C	-2.64509800	-5.32852800	-1.20299800
C	-2.56827100	-4.31478600	-0.24785700
C	-1.60673800	-3.30619400	-0.36976800
C	-0.73481100	-3.31608600	-1.46952000
C	-0.79214900	-4.34766000	-2.40465100
H	-1.79896000	-6.15740200	-3.00346500
H	-3.39934100	-6.10400000	-1.10495800
H	-3.25350600	-4.31912100	0.59423300
H	-0.01804500	-2.50849900	-1.59630200
H	-0.10261200	-4.35245900	-3.24386400
Pd	-0.75044900	0.13009400	0.12450800
C	1.41006800	0.14735800	1.06522000
C	0.82669900	1.41975200	1.19472600
C	0.22250400	1.98198700	0.04179400

H	1.60645000	-0.45123800	1.94597300
H	1.63633500	-0.27975000	0.09595300
C	0.20148200	3.07054300	-0.71568200
H	0.94779500	3.85351000	-0.60763200
H	-0.49013500	3.18223000	-1.54514300
C	3.93860900	0.23173700	0.69420000
C	4.25469100	1.33487500	1.52786900
N	3.66774700	2.17590600	-0.37237200
C	4.14216900	-1.23051600	0.94164300
H	3.89870100	-1.48613100	1.98041300
H	3.43522300	-1.78321900	0.30829000
C	5.54771100	-1.70656800	0.62590300
C	6.38749000	-2.20617700	1.62513800
C	6.03021300	-1.63155800	-0.68919600
C	7.68656100	-2.62533700	1.32454500
H	6.02107600	-2.26525600	2.64804100
C	7.32319300	-2.05204000	-0.99138400
H	5.37907800	-1.23271600	-1.46280000
C	8.15757100	-2.55053100	0.01457600
H	8.32806300	-3.00810300	2.11438000
H	7.68514000	-1.98915900	-2.01449000
H	9.16713400	-2.87504100	-0.22305300
C	4.72153300	1.28363000	2.94852700
H	3.99919600	0.75979900	3.58930700
H	5.67320100	0.74370700	3.03001700
H	4.85990500	2.29743500	3.33470100
N	4.07097800	2.49706300	0.92370800
C	3.36267100	3.21355100	-1.25847200
C	3.42786500	4.54652200	-0.81554600
C	2.94897000	2.94473500	-2.57375300
C	3.04798600	5.58088100	-1.66832000
H	3.76176500	4.74051000	0.19540200
C	2.56677200	3.99164700	-3.40846900
H	2.91671800	1.91727500	-2.89935300
C	2.60363000	5.31588700	-2.96658400
H	3.09814500	6.60622300	-1.30988400
H	2.22793100	3.76088200	-4.41565200
H	2.30116500	6.12722000	-3.62271800
C	3.57255000	0.78394300	-0.57343100
O	3.15006400	0.20380000	-1.60987600
C	0.69905800	2.05823300	2.55788700
H	1.66439400	2.51445400	2.80532000
H	-0.05589600	2.84790700	2.55908500
H	0.44756300	1.32076100	3.32679700

H	-3.09859200	-2.75524100	2.49104700
H	-3.70514200	-1.76513000	1.13610700
H	-2.54235900	2.79541400	-1.80235900
H	-4.01723100	1.92292100	-2.28781500

TS'_left_*Si*_*a*

C	2.97538700	-2.75701900	4.44324200
C	2.01855800	-3.82752800	3.91086100
C	0.60971900	-3.25003900	3.76011700
C	0.58301700	-2.02326500	2.83185200
C	1.57670600	-0.94476700	3.30884800
C	2.98005500	-1.53530300	3.52228300
N	-0.76327100	-1.45069800	2.80289700
N	1.71076600	0.17566300	2.37080200
C	-1.79790000	-2.07754400	2.21243800
O	-1.72307500	-3.17943500	1.65724200
C	0.83621100	1.19147000	2.26059600
O	-0.27115400	1.23102200	2.83038400
H	-0.84418400	-0.45131400	3.01396400
H	2.58298900	0.20036500	1.81911800
C	-5.73363800	-0.41975200	2.68146000
C	-5.24399500	-0.67609200	1.39933900
C	-3.94376100	-1.15760000	1.21068900
C	-3.13386700	-1.39082900	2.34162900
C	-3.62023400	-1.09943300	3.61780400
C	-4.91688400	-0.61378700	3.79218600
H	-6.74830000	-0.05177200	2.80471700
H	-5.87498100	-0.47586500	0.54114400
P	-3.18704100	-1.28994800	-0.46349100
H	-2.97867800	-1.27452200	4.47582100
H	-5.28412100	-0.39375000	4.79014600
C	2.02629200	4.64735000	0.01962600
C	0.76142000	4.09356800	-0.18628700
C	0.35572700	2.94125700	0.49948000
C	1.26105400	2.34781100	1.40808700
C	2.53129900	2.89962900	1.59169600
C	2.91781700	4.04971100	0.90455500
H	2.31538300	5.54200400	-0.52415200
H	0.08964300	4.56427200	-0.89365600
P	-1.26070700	2.14289800	0.09207400
H	3.22373100	2.42399800	2.27611400
H	3.90756900	4.46329600	1.05649000
H	2.66232900	-2.46105600	5.45512100
H	3.99439500	-3.15207200	4.53008600

H	2.37760600	-4.18512700	2.93533600
H	1.99783800	-4.69735200	4.57814700
H	-0.08580200	-3.99249500	3.36083000
H	0.23003200	-2.94188400	4.74487900
H	0.85804500	-2.33452700	1.81542600
H	1.19637500	-0.54174200	4.25817500
H	3.63269200	-0.74783000	3.91591800
H	3.39280700	-1.80765500	2.54411600
C	-4.36586600	3.73808000	3.13040100
C	-3.03311800	4.13915700	3.24074700
C	-2.07950800	3.66537400	2.34067700
C	-2.45370100	2.78103200	1.32269900
C	-3.79051400	2.37595400	1.21948200
C	-4.74283400	2.85650900	2.11622500
H	-5.10629400	4.10773300	3.83436400
H	-2.73242900	4.81988600	4.03220600
H	-1.04310600	3.96587000	2.43905900
H	-4.09090200	1.69119400	0.43469500
H	-5.77470900	2.53141100	2.02764800
C	-2.44665800	4.30010200	-3.85529100
C	-1.48507800	3.28513100	-3.84344000
C	-1.13721600	2.67026700	-2.64396500
C	-1.73636000	3.06284100	-1.43558500
C	-2.68976600	4.08759500	-1.45413500
C	-3.04446700	4.69930900	-2.65971900
H	-2.72634400	4.77536300	-4.79116100
H	-1.01369800	2.96764300	-4.76922700
H	-0.40534100	1.86938100	-2.63683400
H	-3.16338300	4.40266100	-0.53115200
H	-3.78905800	5.49056400	-2.66044100
C	-3.71973300	-5.64623700	-1.92983800
C	-3.15076100	-4.67196000	-2.75435600
C	-2.97861300	-3.37419500	-2.28030200
C	-3.37875800	-3.03651000	-0.97976400
C	-3.94543700	-4.01374900	-0.15646000
C	-4.11468700	-5.31457900	-0.63352600
H	-3.84987800	-6.66063900	-2.29625000
H	-2.83467300	-4.92513200	-3.76236900
H	-2.52233600	-2.62050300	-2.91549400
H	-4.23108500	-3.76519200	0.85816800
H	-4.55048200	-6.07026300	0.01382600
C	-6.32644100	0.71173800	-3.24527900
C	-6.57889700	-0.55500100	-2.70916500
C	-5.63797700	-1.16840800	-1.88448700

C	-4.43377800	-0.51376100	-1.57472800
C	-4.18464800	0.74516200	-2.12982800
C	-5.12556700	1.35962800	-2.95852000
H	-7.06133900	1.18390600	-3.89130400
H	-7.50811400	-1.06849800	-2.93983500
H	-5.83232500	-2.15845600	-1.48427700
H	-3.24688700	1.24056000	-1.92542200
H	-4.90694000	2.33756600	-3.37629300
Pd	-1.09322900	-0.20886700	-0.57462800
C	1.60642300	0.04314900	-1.19509100
C	0.76440000	-0.79493700	-1.94521200
C	4.27492700	-0.72033900	-3.94897300
H	4.88498800	-1.63046000	-4.00990500
H	3.25881800	-0.99243600	-4.25459800
H	4.67072300	0.01346900	-4.65615300
C	4.71612500	-3.13133800	-1.12872300
C	5.66329800	-2.98650700	-0.10413600
C	4.93208500	-4.10159500	-2.11152100
C	6.79487600	-3.79816000	-0.06750200
H	5.50673800	-2.22567700	0.65581600
C	6.06815500	-4.91458100	-2.07906700
H	4.20208000	-4.22283000	-2.90909200
C	7.00272000	-4.76564400	-1.05538900
H	7.51989800	-3.67550800	0.73295300
H	6.22026600	-5.66228400	-2.85316000
H	7.88733200	-5.39590700	-1.02602800
C	3.49400100	-2.23414200	-1.16760100
H	2.82176600	-2.57024100	-1.96591200
H	2.93297700	-2.33595800	-0.23072200
C	0.03555700	-3.09957900	-0.96955900
H	-0.75134900	-3.54841100	-0.37575800
H	0.86558700	-3.74031400	-1.26667800
C	4.24596800	0.08631700	-0.30307100
O	4.10059500	-0.02739200	0.94628200
N	4.81639700	1.18350400	-0.95194300
N	4.84711300	1.02021900	-2.33643200
C	4.30984100	-0.16130400	-2.56240300
C	5.41281000	2.32222400	-0.38695700
C	5.60835600	3.45875400	-1.18541900
C	5.82927500	2.33240300	0.95367800
C	6.19805800	4.59649000	-0.64097700
H	5.29781900	3.42395400	-2.22195100
C	6.41225700	3.48224900	1.48394300
H	5.68048000	1.44799700	1.55758000

C	0.03561500	-1.81643100	-1.28785900
H	1.92894100	1.00218700	-1.58539600
H	1.70813800	-0.11791800	-0.13278800
C	3.83429800	-0.78679800	-1.36479500
C	6.59845600	4.62204300	0.69784200
H	6.34341800	5.47151200	-1.26907900
H	6.73099600	3.48114400	2.52310900
H	7.05637300	5.51247500	1.11886600
C	0.57609500	-0.59128200	-3.43530800
H	-0.48406500	-0.58631100	-3.70657300
H	1.03015600	0.34740400	-3.76797100
H	1.05147700	-1.41049100	-3.98805400

TS'_left_*Si*_*b*

C	-2.80458600	-3.88393800	-3.84674700
C	-2.30078900	-4.70027900	-2.65386400
C	-0.88415700	-4.27240300	-2.27005300
C	-0.79172100	-2.77288400	-1.93526700
C	-1.38062800	-1.91236100	-3.07220800
C	-2.76515700	-2.38724600	-3.53005000
N	0.61730200	-2.39892000	-1.77083800
N	-1.48551000	-0.49321500	-2.70959200
C	1.40674500	-2.85859400	-0.78122000
O	1.02284200	-3.54355600	0.17109800
C	-0.45232400	0.36042600	-2.83758600
O	0.70576900	0.00450400	-3.12301900
H	0.97636800	-1.66826600	-2.38742900
H	-2.38870600	-0.17980800	-2.32886300
C	5.65937600	-2.42017600	-1.28664300
C	5.05003800	-1.88386400	-0.15066200
C	3.66129600	-1.95262300	0.01992300
C	2.88144900	-2.58369100	-0.96996600
C	3.49851600	-3.09214100	-2.11632900
C	4.88165900	-3.01183100	-2.27968700
H	6.73870800	-2.36169400	-1.39575900
H	5.65947200	-1.38732200	0.59546000
P	2.82154800	-1.04210400	1.37975400
H	2.88320100	-3.56458500	-2.87571300
H	5.34586900	-3.41224700	-3.17611200
C	-1.18827700	4.57464400	-2.58137200
C	-0.07574300	4.02486800	-1.93864400
C	0.17705200	2.64866000	-1.98045800
C	-0.73636000	1.81933600	-2.66931600
C	-1.85105300	2.37505400	-3.29960400

C	-2.07719700	3.75125500	-3.26594100
H	-1.35227700	5.64771600	-2.54405400
H	0.60198200	4.67966200	-1.40410300
P	1.61643000	1.92904000	-1.06376600
H	-2.53508700	1.72178100	-3.83093000
H	-2.94468700	4.17018600	-3.76622800
H	-2.18313100	-4.09683600	-4.72932700
H	-3.83161700	-4.16998700	-4.10183800
H	-2.97375600	-4.55643100	-1.80373200
H	-2.31201500	-5.77142900	-2.89004800
H	-0.51830600	-4.83573500	-1.40742100
H	-0.19719500	-4.47305000	-3.10560900
H	-1.33553100	-2.56579000	-1.00637300
H	-0.68263200	-1.96952000	-3.91777300
H	-3.05853000	-1.79251900	-4.40379800
H	-3.49013300	-2.16964200	-2.74296300
C	5.02706400	1.51420500	-4.16300900
C	3.94789500	2.35643000	-4.44224700
C	2.90357300	2.48491400	-3.52777100
C	2.93325000	1.76998300	-2.32369400
C	4.01369500	0.92543400	-2.05035500
C	5.05809000	0.79907100	-2.96542600
H	5.83627200	1.41191100	-4.88069400
H	3.91565100	2.90887700	-5.37721500
H	2.05993300	3.12863700	-3.75438900
H	4.03411000	0.35383500	-1.13173900
H	5.88325300	0.12979300	-2.74310900
C	3.03331600	5.48971700	1.57196700
C	1.95749000	4.68662200	1.96112100
C	1.54031000	3.63819100	1.14552000
C	2.18251300	3.38266200	-0.07692800
C	3.25680700	4.19443500	-0.46243800
C	3.68024700	5.24121200	0.36091800
H	3.36720400	6.30108100	2.21232200
H	1.45261000	4.86903500	2.90548400
H	0.72516400	2.99571200	1.46087400
H	3.76669500	4.00365900	-1.40035900
H	4.51698500	5.86198000	0.05253200
C	2.19284700	-3.99456500	4.88323800
C	1.91400500	-2.63260800	5.03082000
C	2.10106700	-1.76551900	3.95876200
C	2.57919500	-2.24949500	2.73187800
C	2.85919500	-3.61119400	2.58766300
C	2.66335100	-4.47977900	3.66349400

H	2.03744600	-4.67422600	5.71627000
H	1.53910200	-2.25037600	5.97581400
H	1.86544600	-0.71079200	4.06736200
H	3.19923600	-3.99596400	1.63456600
H	2.87382300	-5.53839300	3.54193500
C	6.25092500	1.59147600	3.08193700
C	6.12417400	0.26329400	3.49801100
C	5.08823700	-0.53141900	3.00883400
C	4.16687200	-0.00490100	2.08837500
C	4.30191300	1.32689000	1.68169000
C	5.33635700	2.12316800	2.17283800
H	7.05617900	2.20812900	3.47131800
H	6.83126900	-0.15443800	4.20921800
H	4.99203000	-1.55907700	3.34267800
H	3.58861300	1.75093500	0.99174100
H	5.41117300	3.15640100	1.84764400
Pd	0.99889200	0.22110400	0.59061700
C	-1.42845200	1.15401100	0.59490000
C	-1.00727200	0.65731000	1.83053700
C	-4.68356300	2.33214600	-1.48527200
H	-5.65475400	2.72091300	-1.15440500
H	-3.96266100	3.15360600	-1.40829000
H	-4.76732500	2.03471300	-2.53435100
C	-5.76073100	2.52578500	2.01436400
C	-6.48983000	1.51201500	2.65359300
C	-6.42326200	3.69683500	1.63696200
C	-7.84878400	1.67468400	2.91145700
H	-5.97653300	0.59632800	2.93587200
C	-7.78819900	3.86061300	1.89060100
H	-5.86524100	4.48786900	1.13998400
C	-8.50446500	2.84994000	2.52994300
H	-8.40115400	0.88210900	3.40962600
H	-8.28851100	4.77726100	1.58840300
H	-9.56549200	2.97361500	2.72962400
C	-4.28749400	2.31193800	1.71567200
H	-3.85415300	3.24927000	1.33943200
H	-3.76727700	2.06956300	2.64946000
C	-0.68382500	-1.87870100	2.27053500
H	0.02312200	-2.68524500	2.12693100
H	-1.65073800	-2.08961600	2.72259300
C	-3.84365400	-0.17106600	1.14862200
O	-3.64249400	-0.67240700	2.26907100
N	-3.85557800	-0.89361500	-0.09005800
N	-4.15667900	-0.04959200	-1.17076300

C	-4.29251200	1.16424800	-0.63762100
C	-4.26495100	-2.24098200	-0.17445900
C	-5.27600600	-2.62080500	-1.07065400
C	-3.68848500	-3.20531200	0.66332700
C	-5.69496800	-3.94874200	-1.12857500
H	-5.72119700	-1.86050100	-1.70101600
C	-4.13357900	-4.52525000	0.61312500
H	-2.90413500	-2.90938300	1.34198000
C	-0.45837500	-0.64483500	1.86349900
H	-1.59562900	2.21232700	0.43699200
H	-1.58496300	0.48723300	-0.24066300
C	-4.05907700	1.18575700	0.75266800
C	-5.13535100	-4.90827700	-0.28140900
H	-6.47707800	-4.22978900	-1.82917500
H	-3.67957700	-5.26182500	1.27055000
H	-5.46914500	-5.94104400	-0.32408600
C	-0.97892500	1.51277100	3.07459900
H	-0.03461300	1.40620700	3.61686000
H	-1.14442000	2.57012700	2.84387000
H	-1.78973900	1.17218800	3.72799200

TS'_left_*Re_a*

C	-2.64438800	3.65061100	4.06406700
C	-1.71920300	4.56581600	3.25662000
C	-0.33656900	3.92715900	3.11355300
C	-0.40953800	2.54622400	2.43928000
C	-1.36601600	1.60936700	3.20098300
C	-2.73659100	2.26774400	3.41495600
N	0.92681600	1.94388500	2.39356700
N	-1.59516900	0.33348400	2.51451000
C	1.91207100	2.45421200	1.62900200
O	1.77993900	3.42712100	0.87763800
C	-0.76623400	-0.72091900	2.62438800
O	0.34645200	-0.67281600	3.17724100
H	1.03817300	1.02139500	2.82008800
H	-2.44076200	0.26080000	1.93431400
C	5.93391500	1.00037900	2.04808100
C	5.30195300	0.93754200	0.80305900
C	3.97090300	1.34312000	0.65821700
C	3.27584300	1.83075700	1.78489800
C	3.90393900	1.86209700	3.03037700
C	5.23126900	1.44820400	3.16531100
H	6.97006500	0.68753900	2.14175900
H	5.84053800	0.54983000	-0.05475300

P	3.02176700	1.05399400	-0.89003000
H	3.35148200	2.22708300	3.89085000
H	5.71189300	1.47980800	4.13878600
C	-2.19963100	-4.51228700	1.20635400
C	-0.94472200	-4.05244200	0.79747400
C	-0.45301800	-2.81559100	1.23140000
C	-1.26921400	-2.02246500	2.06806400
C	-2.53327600	-2.47884300	2.45208100
C	-2.99659300	-3.72784300	2.03674000
H	-2.55451200	-5.47998800	0.86366500
H	-0.34211800	-4.66537000	0.13636900
P	1.17616900	-2.16401600	0.63865100
H	-3.15269600	-1.84815800	3.08116000
H	-3.98214900	-4.06881600	2.33806600
H	-2.26076100	3.55755700	5.09059200
H	-3.64964600	4.08285700	4.14138100
H	-2.15167000	4.73685300	2.26201500
H	-1.63034900	5.54695200	3.73861500
H	0.33233300	4.55871300	2.52394300
H	0.11934300	3.80201000	4.10654700
H	-0.78178700	2.66395900	1.41469200
H	-0.90624800	1.37460800	4.17019700
H	-3.35736500	1.59167600	4.01481800
H	-3.22005900	2.35554700	2.43816500
C	4.08209600	-3.22958200	4.09299000
C	2.91750700	-3.98007500	3.91411900
C	2.02712500	-3.65848300	2.89119100
C	2.29452400	-2.58250100	2.03275000
C	3.45433300	-1.82610000	2.22731500
C	4.34697200	-2.15138100	3.24904800
H	4.77328000	-3.47984300	4.89304900
H	2.69855400	-4.81332200	4.57625000
H	1.11672000	-4.23623600	2.76765300
H	3.65334300	-0.96979400	1.59699900
H	5.23903600	-1.54878100	3.38618000
C	2.45029900	-5.11282500	-2.72625600
C	1.70451700	-3.96648700	-3.00952400
C	1.32306900	-3.11195300	-1.97807400
C	1.66680300	-3.39473400	-0.64685000
C	2.41647400	-4.54623300	-0.37167700
C	2.80603700	-5.39880500	-1.40725100
H	2.76061100	-5.77434800	-3.53014500
H	1.43730300	-3.72735900	-4.03497700
H	0.78435200	-2.19574600	-2.19816600

H	2.70667100	-4.77139300	0.64881000
H	3.39084700	-6.28627500	-1.18101200
C	3.25948200	4.96251700	-3.35562000
C	2.44720900	3.90445600	-3.76879500
C	2.37423000	2.74265600	-3.00266000
C	3.12580600	2.62060700	-1.82601800
C	3.93692700	3.68438900	-1.41593000
C	4.00053300	4.85045000	-2.17803500
H	3.30881900	5.87347800	-3.94552900
H	1.85917200	3.98859600	-4.67834500
H	1.72268000	1.93125700	-3.30908600
H	4.50335200	3.60714300	-0.49477700
H	4.62621800	5.67470200	-1.84744700
C	5.66695500	-1.88655800	-3.34145800
C	5.53163300	-0.56612000	-3.77004900
C	4.76479600	0.34101700	-3.03484200
C	4.11701700	-0.06704100	-1.86058400
C	4.26938600	-1.39630800	-1.43447200
C	5.03748800	-2.29845000	-2.16440000
H	6.25847200	-2.59060800	-3.92005300
H	6.02424900	-0.23488700	-4.68013900
H	4.67001100	1.36437900	-3.37912100
H	3.78409800	-1.73221900	-0.52840400
H	5.12298700	-3.32492700	-1.82145900
Pd	0.96733300	-0.03203400	-0.52252900
C	-1.64643500	-0.73727100	-1.10980800
C	-0.94236700	0.16506700	-1.95146800
C	-4.15233100	-0.89702600	-3.93081100
H	-3.31496000	-1.59145500	-4.07133600
H	-5.07332500	-1.49346400	-3.94377200
H	-4.17776700	-0.19980800	-4.77248500
C	-5.59876000	-2.43563100	-0.72050100
C	-6.29745400	-3.26357100	-1.60456500
C	-6.28550200	-1.87621200	0.36753200
C	-7.65533900	-3.53041300	-1.41398800
H	-5.77145600	-3.70650300	-2.44756400
C	-7.63990300	-2.14232200	0.55964300
H	-5.74880800	-1.21863200	1.04537000
C	-8.33044500	-2.97073300	-0.32974200
H	-8.18288400	-4.17566500	-2.11160300
H	-8.16056900	-1.70122700	1.40569100
H	-9.38649100	-3.17644100	-0.17783400
C	-4.12847700	-2.13206600	-0.92681000
H	-3.58970000	-2.33279000	0.00207600

H	-3.71901400	-2.81586500	-1.68106900
C	-0.44983900	2.64234900	-1.26272900
H	0.34213000	3.20103800	-0.77531400
H	-1.34238600	3.18700700	-1.56771100
C	-3.95854600	0.41246800	-0.42945900
O	-3.90155000	0.43818500	0.82520200
N	-4.04185800	1.52175400	-1.26900200
N	-4.07598400	1.16641000	-2.62046900
C	-4.03403400	-0.14580200	-2.64424500
C	-4.10108300	2.88093200	-0.91880700
C	-3.74570200	3.85334700	-1.86559600
C	-4.52846300	3.27503400	0.35684800
C	-3.77803300	5.20215400	-1.51866300
H	-3.45936800	3.53264900	-2.85965900
C	-4.54861200	4.62884200	0.68952900
H	-4.84362000	2.52012800	1.06288100
C	-0.38013400	1.33635900	-1.43568600
H	-1.65885600	-1.78879400	-1.37541000
H	-1.74693900	-0.51765900	-0.05881200
C	-3.85457800	-0.71121000	-1.33609900
C	-4.16603900	5.60027100	-0.23734400
H	-3.49385100	5.94658400	-2.25740000
H	-4.87881700	4.92298500	1.68230200
H	-4.18181700	6.65257100	0.03034700
C	-0.73405100	-0.17327700	-3.41518900
H	-1.47641000	0.35562700	-4.02268000
H	0.26148800	0.12420200	-3.75683900
H	-0.85272700	-1.24744500	-3.59576300

TS'_left_*Re_b*

C	2.89522500	0.67674900	-5.43098300
C	2.11887100	1.98136600	-5.22150600
C	0.69448800	1.67971700	-4.74922900
C	0.67311500	0.85598400	-3.45014500
C	1.53623500	-0.41726000	-3.56823800
C	2.93545500	-0.14591600	-4.14067800
N	-0.69816400	0.44497400	-3.14849600
N	1.69470800	-1.10458400	-2.28344000
C	-1.64410700	1.29722700	-2.70459800
O	-1.45040400	2.49237500	-2.46128100
C	0.77784000	-1.96421800	-1.79135200
O	-0.34527300	-2.13362400	-2.29883100
H	-0.86338600	-0.55982700	-3.05072800

H	2.55802300	-0.90768300	-1.76464100
C	-5.69602300	-0.14891500	-2.73390900
C	-5.18890300	0.43419500	-1.57201900
C	-3.85891700	0.86707500	-1.50314700
C	-3.03387600	0.71105400	-2.63600000
C	-3.54475000	0.10132400	-3.78599600
C	-4.86988500	-0.32982000	-3.83994000
H	-6.73279300	-0.47196600	-2.76406200
H	-5.83138700	0.52791500	-0.70463300
P	-3.11735300	1.44448100	0.07948700
H	-2.89482500	-0.01654400	-4.64712000
H	-5.25138400	-0.79985700	-4.74143700
C	1.92174500	-4.54042000	1.42944900
C	0.69537700	-3.87326700	1.48820300
C	0.29974400	-2.99117300	0.47502200
C	1.18339000	-2.78273600	-0.60877300
C	2.40802800	-3.45058700	-0.65557800
C	2.78301000	-4.33263000	0.35741500
H	2.19745300	-5.22082500	2.22985800
H	0.04437600	-4.04648400	2.33601400
P	-1.28648600	-2.05252400	0.62512200
H	3.07227800	-3.28842700	-1.49561400
H	3.74204700	-4.83363100	0.30273100
H	2.41248800	0.09455600	-6.22922800
H	3.91908500	0.88147400	-5.76495300
H	2.63493200	2.60105400	-4.47505900
H	2.08907400	2.56544700	-6.14889100
H	0.12318300	2.59689000	-4.57933400
H	0.16642400	1.10676300	-5.52470100
H	1.05058800	1.47167200	-2.62601400
H	1.00508100	-1.10752800	-4.23702200
H	3.42857500	-1.11094300	-4.30830000
H	3.53467200	0.37411100	-3.38672200
C	-4.45627100	-4.41128300	-1.78487400
C	-3.16378900	-4.92702300	-1.66771900
C	-2.18946300	-4.22666900	-0.95815200
C	-2.50181500	-3.00003400	-0.36074700
C	-3.79766400	-2.48514000	-0.48376100
C	-4.77157500	-3.18910300	-1.18983500
H	-5.21250700	-4.95815400	-2.34113000
H	-2.91081000	-5.87497000	-2.13419200
H	-1.18340600	-4.62277000	-0.88182900
H	-4.04983100	-1.53659500	-0.02546200
H	-5.77043000	-2.77455700	-1.28267800

C	-2.53385600	-2.91120100	5.02296500
C	-1.52363700	-1.99248900	4.72291600
C	-1.15868400	-1.76854500	3.39840000
C	-1.78866800	-2.46274000	2.35287700
C	-2.79251400	-3.38837700	2.66145800
C	-3.16357600	-3.60768000	3.99096000
H	-2.82581600	-3.08269900	6.05507500
H	-1.02515100	-1.44721100	5.51922200
H	-0.38383100	-1.04752000	3.16471800
H	-3.28972600	-3.93278800	1.86665800
H	-3.94579100	-4.32704000	4.21711500
C	-3.49621500	6.05638300	0.19295000
C	-3.09406700	5.33726300	1.32213600
C	-2.97137300	3.95246100	1.25677200
C	-3.25517300	3.27128800	0.06364700
C	-3.65635900	3.99308900	-1.06403100
C	-3.77544500	5.38278600	-0.99588600
H	-3.58750300	7.13781600	0.24093400
H	-2.86970100	5.85600100	2.24976500
H	-2.64781600	3.39561000	2.13137100
H	-3.85105500	3.47700000	-1.99549700
H	-4.08319900	5.93746300	-1.87767900
C	-6.32344400	0.42673300	3.28432000
C	-6.54457900	1.48032600	2.39150700
C	-5.58518700	1.80101700	1.43340700
C	-4.39440900	1.05937000	1.34742600
C	-4.17585600	0.02039200	2.25756100
C	-5.13521800	-0.29963300	3.22020200
H	-7.07261500	0.18352700	4.03257600
H	-7.46320000	2.05759300	2.44744600
H	-5.75359800	2.63161500	0.75532000
H	-3.24693400	-0.53051300	2.22424000
H	-4.94066000	-1.11138400	3.91421600
Pd	-1.06739700	0.38517700	0.57328400
C	1.52508900	0.24261400	1.17575700
C	0.74249400	1.26516000	1.72900500
C	3.41439100	2.36156100	-1.18159500
H	2.34478500	2.57819100	-1.07535900
H	3.95930000	3.26097500	-0.87218000
H	3.63292200	2.17396000	-2.23501000
C	4.91143100	3.21563800	2.01389100
C	4.81611100	4.54409700	1.58964200
C	6.16617700	2.71494600	2.39067500
C	5.94830500	5.36194700	1.53654100

H	3.84568800	4.94246300	1.30005200
C	7.29523000	3.52925100	2.34357600
H	6.24221300	1.68082200	2.71659000
C	7.19135100	4.85619700	1.91404600
H	5.85640500	6.39184100	1.20119200
H	8.26100500	3.12889100	2.64066800
H	8.07400300	5.48878000	1.87475400
C	3.69828400	2.30529600	2.04108400
H	3.58200000	1.88159600	3.04631200
H	2.79666400	2.89572000	1.83143900
C	0.07990700	3.29583000	0.24650100
H	-0.68122600	3.61491900	-0.45420200
H	0.91946200	3.97035200	0.41780800
C	4.36932100	-0.11845100	1.44883600
O	4.61548300	-0.59199600	2.57036000
N	4.54552700	-0.77685200	0.20355600
N	4.19765900	0.04782300	-0.87539200
C	3.82416000	1.20038400	-0.33310800
C	5.25041700	-1.96058100	-0.04819100
C	5.53791200	-2.33155400	-1.37381900
C	5.66701300	-2.79287500	1.00614000
C	6.19877100	-3.52915700	-1.63633800
H	5.24203800	-1.67042700	-2.17835100
C	6.34508300	-3.97669200	0.72209400
H	5.45203900	-2.49124700	2.02176400
C	0.03802300	2.12133500	0.84957700
H	1.88285100	-0.57494900	1.79082400
H	1.61415400	0.13513800	0.10566400
C	3.81560300	1.16052100	1.08318400
C	6.60619300	-4.36540400	-0.59390800
H	6.40710800	-3.80331200	-2.66755000
H	6.66683100	-4.60696400	1.54757300
H	7.12859500	-5.29433700	-0.80365300
C	0.59222900	1.42407300	3.22985400
H	1.10661200	0.61830500	3.76230900
H	1.03394800	2.37324800	3.55095500
H	-0.46134400	1.42989300	3.52666800

TS'_right_*Si*

C	4.27620400	-0.13428200	4.39501500
C	4.19486400	-1.52945500	3.76509900
C	2.73410800	-1.93261900	3.54877500
C	1.97610600	-0.90515700	2.69369900
C	2.09238400	0.51529800	3.26130700

C	3.55601500	0.90129000	3.52374500
N	0.56119200	-1.27285200	2.61857300
N	1.48320800	1.50333800	2.36607500
C	0.10418600	-2.19914700	1.75402100
O	0.79791500	-2.72382000	0.87483500
C	0.17300300	1.80830500	2.43762200
O	-0.63986200	1.19193100	3.15124200
H	-0.10430400	-0.65923800	3.08951700
H	2.04022600	1.73747900	1.53561300
C	-3.76079700	-3.92684900	2.49547300
C	-3.43240900	-3.51656300	1.20202700
C	-2.21010000	-2.88920300	0.93415400
C	-1.30406200	-2.68065500	1.99524800
C	-1.65570700	-3.06856200	3.29166900
C	-2.87954500	-3.68730300	3.54659000
H	-4.71175900	-4.41971900	2.67748000
H	-4.14747300	-3.66566200	0.40138400
P	-1.86590100	-2.12010700	-0.70208700
H	-0.94755400	-2.90139700	4.09677800
H	-3.13570700	-3.98614100	4.55869900
C	-1.26758700	5.50373400	0.81440400
C	-2.03876200	4.34542100	0.67617600
C	-1.56130200	3.10290300	1.10778900
C	-0.29131200	3.04471500	1.72739100
C	0.48441300	4.20113300	1.82925000
C	0.00803300	5.42798300	1.36443800
H	-1.66119900	6.45433600	0.46671300
H	-3.00777200	4.41124600	0.19544000
P	-2.42430000	1.55979000	0.58180500
H	1.46112200	4.13375600	2.29613300
H	0.62935400	6.31470500	1.44105000
H	3.82439500	-0.15902100	5.39812600
H	5.32260900	0.16559700	4.52782400
H	4.72420000	-1.52917000	2.80494200
H	4.69534600	-2.26898600	4.40188100
H	2.66196400	-2.90901100	3.05729400
H	2.22315400	-2.01862400	4.51905400
H	2.39914700	-0.88834100	1.68722100
H	1.52985200	0.55958200	4.20230000
H	3.57426700	1.89065100	3.99733300
H	4.06203800	0.98771100	2.55801000
C	-5.06602700	0.11942100	4.09239800
C	-4.36387200	1.32103000	4.20831700
C	-3.54564000	1.75932400	3.16870700

C	-3.42556400	0.99503500	2.00179800
C	-4.13341700	-0.20740300	1.88825200
C	-4.95063500	-0.64329400	2.92959300
H	-5.69874900	-0.22174800	4.90704600
H	-4.44673700	1.91589200	5.11345100
H	-2.98294800	2.67962900	3.26908400
H	-4.05587100	-0.80214200	0.98691900
H	-5.48518800	-1.58245100	2.83257200
C	-5.63206900	3.02412200	-2.44835200
C	-4.29797800	2.86805900	-2.83515300
C	-3.34574900	2.44705300	-1.91013800
C	-3.71308900	2.18544400	-0.57939300
C	-5.04953400	2.35548300	-0.19518400
C	-6.00370500	2.76931800	-1.12788100
H	-6.37632300	3.34215000	-3.17296200
H	-4.00015400	3.06481000	-3.86110500
H	-2.31297400	2.31047300	-2.21474500
H	-5.34895700	2.15564900	0.82749500
H	-7.03796200	2.89160800	-0.81863700
C	0.62439600	-5.05596100	-3.27442200
C	0.09493200	-3.88254700	-3.81948200
C	-0.66300700	-3.02850100	-3.02186200
C	-0.89431100	-3.33414100	-1.67267200
C	-0.37321500	-4.51314600	-1.13444300
C	0.38343300	-5.36971700	-1.93667200
H	1.22124900	-5.72149000	-3.89147700
H	0.27316500	-3.63344800	-4.86185500
H	-3.88618600	-0.12449600	-1.36012800
H	-6.06750900	-0.20536500	-2.49587700
Pd	-1.00514800	0.14484300	-0.67150000
C	1.21394100	-0.53511800	-1.40802100
C	0.67741600	0.49236500	-2.19099100
C	0.18723100	1.64744800	-1.52802500
H	1.34787700	-1.53093900	-1.80805400
H	1.43690900	-0.38688500	-0.36068300
C	0.28171200	2.96574400	-1.58935900
H	1.07709000	3.43477700	-2.16306500
H	-0.38108400	3.62928100	-1.05085200
C	3.76782500	-0.43980000	-1.42871900
C	3.90584100	-0.14989700	-2.81377000
N	3.81877300	1.14335900	-3.07749000
N	3.66622300	1.75550300	-1.83533000
C	3.62077600	0.82427900	-0.78885500
O	3.42781600	1.10615600	0.43200600

C	3.93366400	-1.76606700	-0.74741700
H	3.69398200	-2.56806700	-1.45701300
H	3.19293900	-1.87305900	0.05601100
H	3.52771400	3.35025600	-3.90889100
C	3.30041900	5.20970900	-0.52620700
H	3.58139500	3.26232700	0.37010600
C	3.18277100	5.93918400	-1.71014100
H	3.20178300	5.81409800	-3.86374400
H	3.25350500	5.72023000	0.43276400
H	3.03543000	7.01511700	-1.68512700
C	4.07819000	-1.13745500	-3.92436500
H	4.13107900	-0.61559300	-4.88376100
H	4.99473100	-1.72579700	-3.79361800
H	3.24209600	-1.84932200	-3.95844300
C	5.31463900	-2.03136100	-0.16776300
C	5.89681700	-3.29870000	-0.27981300
C	6.01702900	-1.03192900	0.52137700
C	7.14535600	-3.57047900	0.28314000
H	5.36327900	-4.08179700	-0.81442200
C	7.26406000	-1.30098500	1.08580400
H	5.57565400	-0.04615000	0.61982200
C	7.83437300	-2.57077700	0.96991200
H	7.58052600	-4.56141800	0.18042600
H	7.79289200	-0.51416000	1.61783700
H	8.80745000	-2.77723300	1.40747300
C	3.54515000	3.14953100	-1.77350000
C	3.45767300	3.88326000	-2.96972500
C	3.48495500	3.82880700	-0.54382400
C	3.27436600	5.26320800	-2.92939400
H	-1.07493200	-2.11560900	-3.44230000
H	-0.52911100	-4.74384400	-0.08836200
H	0.79331900	-6.28017200	-1.50888400
C	-5.95334500	-2.32596700	-2.90269000
C	-5.17960400	-3.48347500	-2.77097300
C	-3.94748200	-3.43357500	-2.12149500
C	-3.47828200	-2.22321900	-1.58411700
C	-4.25368000	-1.06843100	-1.73606500
C	-5.48695600	-1.11674600	-2.38818000
H	-6.91070600	-2.36882100	-3.41447500
H	-5.53448200	-4.42490900	-3.18090300
H	-3.34273900	-4.33107100	-2.03573100
C	0.51290500	0.34578900	-3.68712000
H	-0.45694300	0.72198500	-4.02587300
H	1.30298700	0.93063000	-4.16966000

H	0.62504700	-0.69573900	-3.99943700

TS'_*exo*_left_*Si*

C	0.32075400	2.25070800	-0.14431300
C	0.23222600	3.45322100	0.39426700
C	1.12357500	1.40006400	-0.92816500
H	-0.60628700	3.76493700	1.00560200
H	1.01614400	4.19016300	0.22349700
C	1.84841700	0.42050800	-0.23651300
H	2.03049600	0.51892600	0.82351000
H	2.25504900	-0.43857700	-0.75142700
C	-1.89030800	-4.31297300	0.82645700
C	-2.99391700	-5.08803200	1.18188700
C	-3.93081800	-4.60522200	2.09773300
C	-3.76155000	-3.33911400	2.66021900
C	-2.66463000	-2.55943500	2.29978500
C	-1.71652300	-3.04185100	1.38570700
C	-3.88164700	0.67391900	3.55122600
C	-3.82476100	0.41180200	4.92036300
C	-2.59309400	0.37293100	5.57882300
C	-1.41423300	0.60043400	4.86633900
C	-1.46780100	0.85939100	3.49764100
C	-2.70098800	0.89928900	2.82815900
H	-3.12420600	-6.07003100	0.73625000
H	-4.79084700	-5.21164600	2.36764800
H	-4.48720200	-2.94725200	3.36716800
H	-2.55446400	-1.56933200	2.72334100
H	-4.74275400	0.22833500	5.47145600
H	-2.55282200	0.15498100	6.64212500
H	-0.45090600	0.54776100	5.36344100
H	-0.55051500	0.99275800	2.93112800
P	-2.65544000	1.09569300	1.00363400
P	-0.31579800	-1.96479100	0.90402300
C	-4.20540100	0.29442600	0.42802400
C	-5.44231100	0.96197400	0.47829700
C	-4.14400500	-0.99588700	-0.11033800
C	-6.59640100	0.33183500	0.01298000
H	-5.49722000	1.97503500	0.86468300
C	-5.29917300	-1.62205600	-0.57776800
C	-6.52681000	-0.96192700	-0.51316400
H	-7.54808100	0.85399500	0.05349100
H	-5.21810800	-2.61837300	-1.00039300
H	-7.42719400	-1.44534500	-0.88170000
C	-3.02485800	2.87516400	0.69850200

C	-3.36898400	3.68997600	1.78827900
C	-2.86912700	3.47462600	-0.57461800
C	-3.57098200	5.06144600	1.63656800
H	-3.46655300	3.24782500	2.77315100
C	-3.05572800	4.85653900	-0.70420900
C	-3.41310300	5.64859400	0.38321800
H	-3.83846600	5.66451900	2.49938200
H	-2.88898200	5.29818900	-1.68007600
H	-3.55307300	6.71746300	0.25301800
C	0.54395900	-2.81761000	-0.47760100
C	1.71419300	-3.55744200	-0.28735000
C	0.12067900	-2.54407900	-1.79896300
C	2.48847300	-3.97118200	-1.37416000
H	2.05408100	-3.78337300	0.71613400
C	0.91286500	-2.93829200	-2.87897500
C	2.10453900	-3.63531400	-2.66814400
H	3.40586200	-4.52111500	-1.20204900
H	0.57925600	-2.71857300	-3.88959800
H	2.73114000	-3.91433300	-3.50852800
C	0.81941500	-2.03961600	2.35462300
C	0.31740500	-2.28010300	3.64305600
C	2.18308100	-1.72823800	2.21536500
C	1.15438800	-2.19906800	4.75691500
H	-0.72426400	-2.53742400	3.78691300
C	3.01839800	-1.65694800	3.32808300
H	2.62884700	-1.55254700	1.24721300
C	2.50565200	-1.88510800	4.60597500
H	0.74382800	-2.39204700	5.74448700
H	4.06281900	-1.41384400	3.16272600
H	3.15424300	-1.82585500	5.47551900
Pd	-0.55628600	0.36578500	0.26136800
C	4.52485700	0.23498200	-1.33180000
C	4.30074600	1.33348300	-0.43614600
C	4.59850000	0.85289600	0.86560100
N	4.89003900	-0.83112400	-0.45953700
O	4.38551700	0.15077800	-2.56769900
H	-4.84003400	0.67686100	3.04265400
H	-1.17943500	-4.68315700	0.09720600
C	4.04802800	2.73306800	-0.89707300
H	4.09529700	2.71520100	-1.99533200
H	3.03171000	3.07248600	-0.64143800
C	4.52247300	1.62090900	2.14815700
H	5.21623100	2.46874600	2.14284800
H	3.51649000	2.03644600	2.30089100

H	4.76309500	0.97176200	2.99442300
N	4.92451500	-0.43106200	0.87694600
C	5.26094100	-2.13674600	-0.79080200
C	5.48038200	-3.07886600	0.23077600
C	5.42450000	-2.52745000	-2.13335300
C	5.85257900	-4.38243400	-0.08819500
H	5.36569000	-2.76657300	1.26043700
C	5.80069900	-3.83552400	-2.43035800
H	5.24776900	-1.79488000	-2.90907300
C	6.01512700	-4.77685000	-1.41946200
H	6.02013100	-5.09590600	0.71508400
H	5.93181400	-4.12009600	-3.47184200
H	6.31082700	-5.79347600	-1.66277800
C	-1.26468000	-2.03882700	-2.09345800
O	-2.24340000	-2.70743800	-1.75075500
N	-1.37983700	-0.89153200	-2.81220100
H	-0.53964700	-0.38396700	-3.05500100
C	-2.63901400	-0.52459400	-3.45171000
C	-2.86661600	0.99391000	-3.46715300
C	-2.68508400	-1.10437500	-4.87828400
H	-3.42406000	-0.99758100	-2.85558300
C	-4.15876500	1.34469500	-4.22979700
H	-2.02294200	1.48858000	-3.95619500
C	-3.98416000	-0.75355700	-5.60896800
H	-1.82700700	-0.71213300	-5.44338800
H	-2.55461000	-2.18988800	-4.80869000
C	-4.18630900	0.76447800	-5.64654800
H	-4.25693600	2.43549900	-4.24959900
H	-5.01328100	0.95395300	-3.65705800
H	-3.96758800	-1.16905200	-6.62318100
H	-4.83147300	-1.22195600	-5.08825900
H	-5.13380600	1.01864800	-6.13596600
H	-3.38785700	1.22527400	-6.24488600
N	-2.93642600	1.55264100	-2.12018600
C	-2.44799100	2.80036200	-1.86020200
O	-1.72932800	3.39779600	-2.66047200
H	-3.19874500	-1.50705100	-0.20673100
H	-3.67677800	1.19401500	-1.53076900
C	5.03579000	3.75296600	-0.36311200
C	6.41524400	3.53221300	-0.47813800
C	4.59900900	4.92282600	0.26566100
C	7.32954900	4.45865100	0.01856100
H	6.76209700	2.61618200	-0.94836400
C	5.51094900	5.85474000	0.76707300

H	3.53064000	5.10196100	0.36956300
C	6.88097000	5.62532800	0.64502400
H	8.39548300	4.26974600	-0.07912800
H	5.14970900	6.75549600	1.25691900
H	7.59438800	6.34533400	1.03678500
C	1.11498400	1.44069900	-2.43586100
H	0.17354600	1.83608900	-2.82308800
H	1.92064800	2.10485000	-2.76607300
H	1.35474700	0.45612100	-2.85188600

TS'_*exo*_left_*Re*

C	0.80658700	0.20749200	-1.98416300
C	0.84200000	1.11748500	-2.94108600
C	1.54287000	-0.84895400	-1.42214700
H	-0.02635300	1.67485000	-3.26303800
H	1.79921800	1.34723400	-3.40258600
C	2.01611200	-0.79967800	-0.09869300
H	1.94681800	0.09506400	0.49936600
H	2.29492400	-1.71098200	0.41772700
C	-2.03490300	2.37532900	2.64029800
C	-2.02567100	3.77010200	2.69350900
C	-0.90846100	4.48759900	2.27063400
C	0.19689200	3.80542400	1.75823400
C	0.18294300	2.41412300	1.68688600
C	-0.91773900	1.67657500	2.16581200
C	-1.27860500	3.27021200	-1.43477400
C	-0.84374800	4.45791100	-2.01384500
C	-1.18665900	4.75612900	-3.33541200
C	-1.97428200	3.86506200	-4.06480200
C	-2.43034700	2.68131500	-3.47869300
C	-2.08749100	2.37779400	-2.15590800
H	-2.90835100	4.28983300	3.05422200
H	-0.90281800	5.57283800	2.31785900
H	1.06010200	4.34730600	1.39248700
H	1.02567000	1.91872300	1.21876500
H	-0.21524200	5.12967300	-1.43830300
H	-0.83286300	5.67352100	-3.79728000
H	-2.23373500	4.08532000	-5.09642100
H	-3.03324700	1.99262700	-4.05925400
P	-2.54203300	0.81770400	-1.31292700
P	-0.86473000	-0.15292600	1.96856400
C	-3.78575100	1.38159600	-0.07019400
C	-4.08386600	2.72907700	0.16417100
C	-4.47699000	0.39632900	0.65044600

C	-5.04175500	3.08427600	1.11577000
H	-3.56661900	3.50529800	-0.38642500
C	-5.42068500	0.75372400	1.61070100
C	-5.70319800	2.10099000	1.85086200
H	-5.26187300	4.13467200	1.28528600
H	-5.93269100	-0.02331500	2.16970400
H	-6.44099100	2.37977000	2.59809200
C	-3.68814000	-0.00899700	-2.50284000
C	-4.89527800	0.64240200	-2.81549400
C	-3.41142100	-1.24106800	-3.11487800
C	-5.79291600	0.09704100	-3.72725500
H	-5.12943200	1.58663900	-2.33486300
C	-4.32023700	-1.78624600	-4.03155800
C	-5.50316800	-1.12329800	-4.34179800
H	-6.71688500	0.62072700	-3.95455500
H	-4.08759800	-2.73972300	-4.49648900
H	-6.19943800	-1.55849600	-5.05274300
C	-2.28423900	-0.80147500	2.95933500
C	-2.54506700	-0.23542300	4.21915100
C	-3.10910700	-1.85107500	2.51535900
C	-3.63331400	-0.63953400	4.98514900
H	-1.88617100	0.53915100	4.59575400
C	-4.22039800	-2.23729300	3.28168300
C	-4.48753100	-1.63491700	4.50520100
H	-3.81386300	-0.17936100	5.95214600
H	-4.86303900	-3.02155600	2.89655000
H	-5.34693100	-1.95063900	5.08927300
C	0.49325400	-0.70736700	3.10162800
C	1.64516400	0.05316400	3.34422500
C	0.39517300	-1.97713300	3.70148500
C	2.65562100	-0.43483900	4.17253000
H	1.77757300	1.03308700	2.90967900
C	1.41762200	-2.47170400	4.50868300
H	-0.50813800	-2.56986400	3.59117400
C	2.55387100	-1.69807700	4.75197000
H	3.52915400	0.18435200	4.34234400
H	1.31333700	-3.45231300	4.96493400
H	3.34835800	-2.07531300	5.38911300
Pd	-0.60189800	-0.26374800	-0.45590500
C	4.02572300	0.87476000	-0.84160100
C	4.44274400	-0.36123700	-0.23445300
C	4.63706400	-0.06924700	1.14404800
N	3.82825900	1.73933800	0.27189500
O	3.82579600	1.15288200	-2.03431000

H	-0.97472400	3.02708300	-0.42465300
H	-2.93078700	1.84557900	2.93692200
C	5.01079800	-1.50557700	-1.02139500
H	4.73927500	-2.47048300	-0.57293000
C	5.20745300	-0.99764300	2.16801900
H	4.52431600	-1.82723900	2.39187200
H	6.14664600	-1.42978900	1.80419900
H	5.40921800	-0.46082100	3.09791800
N	4.24865700	1.15327700	1.46674800
C	3.51461100	3.10488500	0.25312400
C	3.79646300	3.90063100	1.37689600
C	2.91224600	3.68894800	-0.87546500
C	3.49564000	5.26083200	1.36108400
H	4.25514700	3.43470000	2.23951800
C	2.62915500	5.05357200	-0.87471600
H	2.70118900	3.07160400	-1.73648100
C	2.91378900	5.85204800	0.23625800
H	3.72354600	5.86333400	2.23670400
H	2.18204500	5.49474400	-1.76173100
H	2.69016200	6.91503500	0.22485600
C	-2.91354300	-2.66748600	1.26754300
O	-3.88118800	-2.93784600	0.54065100
N	-1.68317000	-3.18459900	1.04775000
H	-0.92042500	-2.92063000	1.65497500
C	-1.41725600	-4.23549500	0.06420300
C	-1.13841800	-3.69431200	-1.34565400
C	-0.23298300	-5.07530600	0.55887800
H	-2.32052900	-4.85702100	0.02305700
C	-0.84096200	-4.85411000	-2.31037300
H	-0.26464100	-3.03900600	-1.28798300
C	0.08974100	-6.22497300	-0.40291300
H	0.64564700	-4.41956900	0.65360100
H	-0.45853900	-5.45780400	1.56145000
C	0.33739100	-5.70301000	-1.82281400
H	-0.64253800	-4.43486700	-3.29994500
H	-1.74375300	-5.47581500	-2.39308600
H	0.96050000	-6.77970100	-0.03488500
H	-0.75316600	-6.93008400	-0.41421000
H	0.50892800	-6.53858600	-2.51094200
H	1.25174200	-5.09501900	-1.83367900
N	-2.26417800	-2.88777400	-1.80076100
C	-2.15794600	-2.03616800	-2.84763400
O	-1.16901500	-1.94696300	-3.57333500
H	-4.30112700	-0.65352600	0.44712100

H	-3.10040500	-2.87479100	-1.22057700
H	4.56188200	-1.47646700	-2.01921600
C	6.52043600	-1.42191100	-1.16084800
C	7.36208400	-2.39170000	-0.60878300
C	7.09441200	-0.33748900	-1.84066500
C	8.75087300	-2.28603500	-0.72644500
H	6.92577400	-3.23950900	-0.08449900
C	8.47789500	-0.23220100	-1.96316700
H	6.44171800	0.41883100	-2.26871500
C	9.31267500	-1.20532700	-1.40453200
H	9.39023400	-3.04860000	-0.28893800
H	8.90852500	0.61198900	-2.49537400
H	10.39184300	-1.11966700	-1.49878100
C	1.81676300	-2.05316200	-2.29669600
H	0.94736400	-2.30537500	-2.90540700
H	2.63457200	-1.78882900	-2.97668900
H	2.13457200	-2.91851700	-1.70488700

TS'_*exo*_right_*Si*

C	2.46936000	2.79173200	-5.90339500
C	1.96679600	1.36667100	-6.16164300
C	2.63208600	0.37735400	-5.20019100
C	2.40500400	0.77335200	-3.73309400
C	2.88581600	2.20500400	-3.45930400
C	2.24474300	3.19503600	-4.44242800
N	3.07863700	-0.16277400	-2.83790600
N	2.58841900	2.65490100	-2.10123000
C	2.42158300	-1.27208700	-2.42428200
O	1.28057100	-1.55665100	-2.81127000
C	3.33754600	2.36572700	-1.01325400
O	4.30432400	1.59302400	-1.02325500
H	3.82421000	0.21173600	-2.24995400
H	1.68441400	3.08475800	-1.93429700
C	4.56728200	-4.06709800	0.02248900
C	3.20301700	-3.86206600	0.23585600
C	2.48350500	-2.93481400	-0.52314500
C	3.17201000	-2.19789900	-1.51459100
C	4.54164400	-2.39668300	-1.70883300
C	5.24275200	-3.32820700	-0.94385300
H	5.10025200	-4.79508300	0.62719900
H	2.70703800	-4.41348600	1.02489900
P	0.73907200	-2.47997100	-0.12536800
H	5.05038600	-1.83328200	-2.48426600
H	6.30607800	-3.47494200	-1.10710900

C	2.44707900	4.78953400	2.41797700
C	2.23031000	3.41377600	2.48941000
C	2.49086200	2.57816100	1.39399900
C	2.97844700	3.16270300	0.21003400
C	3.18212200	4.54546600	0.14011500
C	2.92143300	5.36123000	1.23946400
H	2.22975800	5.41176300	3.28092500
H	1.83275800	2.98858600	3.40271400
P	2.01725600	0.79487400	1.46824800
H	3.54468000	4.97687900	-0.78800700
H	3.07875600	6.43331100	1.17053800
H	3.54232900	2.84846400	-6.13585500
H	1.96554800	3.50720300	-6.56331400
H	0.87707900	1.33321200	-6.02316400
H	2.16154500	1.07377700	-7.19973000
H	2.24254300	-0.63537200	-5.34446600
H	3.71404600	0.33950300	-5.38994100
H	1.33420300	0.72077400	-3.51654800
H	3.97756900	2.23506100	-3.56841600
H	2.64114900	4.19773300	-4.24524200
H	1.16363100	3.23446700	-4.24209800
C	5.87888100	-1.45437200	2.70175800
C	5.96835400	-0.14295700	2.23501000
C	4.81760800	0.55519400	1.86777900
C	3.56339400	-0.06211200	1.95281000
C	3.48104000	-1.38735800	2.40722700
C	4.63066100	-2.07278200	2.79060100
H	6.77633100	-1.99261200	2.99362200
H	6.93599600	0.34473900	2.15886900
H	4.90025200	1.56800600	1.49629900
H	2.51784400	-1.87958100	2.47998000
H	4.54992700	-3.09473900	3.14909400
C	-0.70252700	0.51450900	5.22738700
C	-1.22680700	0.89876900	3.99035200
C	-0.38344400	1.02483800	2.88795000
C	0.98878100	0.74231600	2.99911900
C	1.50899900	0.37610800	4.24787700
C	0.66557100	0.26601300	5.35569300
H	-1.35790100	0.40885400	6.08758700
H	-2.28813200	1.08056800	3.84962600
H	-0.80411500	1.32953500	1.93667900
H	2.56588600	0.15925900	4.35380200
H	1.08016000	-0.02533900	6.31687500
C	-2.08532400	-5.07163800	-2.74543200

C	-2.55900900	-4.41100200	-1.60998200
C	-1.68779200	-3.64912800	-0.83379000
C	-0.33628600	-3.52332400	-1.19306800
C	0.13360900	-4.19465000	-2.32567600
C	-0.73807400	-4.96749700	-3.09395600
H	-2.76204000	-5.66686800	-3.35167900
H	-3.60422800	-4.49009100	-1.32498300
H	-2.05950600	-3.16349900	0.06377200
H	1.17236200	-4.09946500	-2.61529200
H	-0.36144400	-5.48546300	-3.97160600
C	-0.36822200	-4.47507100	3.92166800
C	-0.00602100	-5.27821000	2.83467500
C	0.34361900	-4.69482000	1.61974800
C	0.36265100	-3.29531200	1.48187900
C	-0.02769700	-2.50409000	2.56710000
C	-0.38924000	-3.08872900	3.78381600
H	-0.64934200	-4.93432100	4.86520900
H	-0.01153100	-6.36026600	2.93160600
H	0.58000700	-5.32429700	0.76714200
H	-0.06369100	-1.42907200	2.45879000
H	-0.68914200	-2.45172400	4.60944100
Pd	0.53782600	-0.04347500	-0.16216500
C	-1.81946000	-0.23306300	-0.61396600
C	-1.22730900	0.78608100	-1.38076200
C	-0.45232600	1.74692200	-0.69396000
H	-2.16519100	-1.13512000	-1.09902400
H	-1.87426000	-0.17161100	0.46263300
C	-0.38149300	3.04415900	-0.43276400
H	-1.19653100	3.70759600	-0.71139000
H	0.42985600	3.48059600	0.13702900
C	-4.29309500	0.11863200	-0.18099400
C	-4.12647300	0.05511700	1.22864200
N	-3.73913100	2.10990400	0.68567900
C	-4.86069100	-0.91141400	-1.10980800
H	-4.60175600	-0.61351300	-2.13398600
H	-4.40205400	-1.89541400	-0.93940800
C	-6.36788000	-1.04037100	-0.99218200
C	-7.18507800	0.05149800	-1.31951000
C	-6.96752800	-2.21595200	-0.53237300
C	-8.56904500	-0.03840300	-1.19244000
H	-6.71768400	0.96852300	-1.66903300
C	-8.35592400	-2.30771900	-0.39932800
H	-6.34084000	-3.06815600	-0.27588700
C	-9.16078600	-1.21863100	-0.73022000

H	-9.19073700	0.81452700	-1.45231400
H	-8.80539200	-3.22922400	-0.03778800
H	-10.24069600	-1.28555300	-0.62887000
C	-4.24307500	-1.16288600	2.09001800
H	-5.21911400	-1.64523500	1.96031000
H	-3.47648900	-1.90990200	1.83512200
H	-4.11781900	-0.89641600	3.14327400
N	-3.75415600	1.21330600	1.75344800
C	-3.34296000	3.43465800	0.89270500
C	-2.69853400	3.79862300	2.08680700
C	-3.55615100	4.41219600	-0.09759600
C	-2.24104200	5.10138600	2.26675700
H	-2.55870200	3.05416300	2.85710800
C	-3.09014800	5.71052600	0.10179500
H	-4.05433900	4.12440500	-1.01281000
C	-2.42177700	6.06782300	1.27520100
H	-1.73512800	5.35939700	3.19412800
H	-3.25736900	6.45276100	-0.67503100
H	-2.05938000	7.08193000	1.41864500
C	-4.08666600	1.49016400	-0.54206400
O	-4.12288300	2.05314800	-1.65560800
C	-1.32834000	0.74964000	-2.88781900
H	-0.69815100	1.51953500	-3.33964400
H	-1.03412900	-0.22771000	-3.28217300
H	-2.36923700	0.96508900	-3.15015000

TS'_*exo*_right_*Re*

C	2.00732000	5.47524800	3.65692300
C	1.58298600	5.87264700	2.23844100
C	2.36617900	5.06767500	1.19715000
C	2.18781500	3.55649500	1.40749400
C	2.59193000	3.13937800	2.82815600
C	1.83872000	3.96779300	3.87891100
N	2.95181800	2.80083100	0.41861400
N	2.35066900	1.72059500	3.09521500
C	2.35148000	2.45651200	-0.74565600
O	1.19818200	2.80479900	-1.02749000
C	3.20095600	0.73100600	2.74486600
O	4.21504800	0.90273700	2.05576600
H	3.72339200	2.22903800	0.76133700
H	1.44426600	1.44546700	3.45911500
C	4.64485600	0.37437100	-3.70940400
C	3.28448600	0.09839700	-3.55957700
C	2.52425000	0.73696200	-2.57671600

C	3.16381100	1.66884800	-1.72708000
C	4.53011500	1.92253400	-1.86950500
C	5.27367800	1.27742500	-2.85775200
H	5.21264000	-0.13416400	-4.48303700
H	2.82348700	-0.64422200	-4.19945100
P	0.78272600	0.25789400	-2.21229400
H	5.00234800	2.65225000	-1.21962200
H	6.33402400	1.48626500	-2.96219400
C	2.65387900	-3.04517200	4.70689300
C	2.45072400	-2.96852300	3.32924400
C	2.57268200	-1.75274200	2.64024000
C	2.91663500	-0.60343900	3.37575300
C	3.09778700	-0.68181900	4.76128500
C	2.96859600	-1.89710400	5.43054600
H	2.55062600	-4.00123200	5.21133600
H	2.17504200	-3.86479900	2.78786100
P	2.10749900	-1.66390200	0.85105100
H	3.34861100	0.22103700	5.30993800
H	3.11049400	-1.94374500	6.50600200
H	3.06022000	5.75000800	3.81298700
H	1.42366500	6.02295600	4.40553600
H	0.50774600	5.68521600	2.11147700
H	1.73658800	6.94621600	2.08053400
H	2.03824100	5.31188200	0.18184300
H	3.43646800	5.31030400	1.26120600
H	1.13382300	3.30883600	1.25678300
H	3.67188900	3.29670000	2.94511200
H	2.18506000	3.67580900	4.87715000
H	0.76970400	3.71459400	3.82469300
C	6.03453500	-2.39611300	-1.49607200
C	6.09559600	-1.98522000	-0.16451100
C	4.92410600	-1.76458000	0.56045200
C	3.67649000	-1.94201000	-0.05120900
C	3.62048000	-2.33047100	-1.39878400
C	4.79323300	-2.56987400	-2.11007100
H	6.94888600	-2.57609400	-2.05440600
H	7.05749900	-1.83999300	0.31870300
H	4.98330100	-1.43293400	1.58879800
H	2.66218500	-2.46144500	-1.88913600
H	4.73457400	-2.88032400	-3.14915100
C	-0.30147000	-5.61869600	0.36691100
C	-0.90208700	-4.46912100	0.88744300
C	-0.15983600	-3.29817900	1.03010700
C	1.19436100	-3.25878400	0.65458800

C	1.79365300	-4.42149500	0.15317900
C	1.04621200	-5.59235100	0.00611200
H	-0.88094600	-6.52972600	0.24796800
H	-1.94881900	-4.47827400	1.17717000
H	-0.63513000	-2.40937100	1.42871500
H	2.83918800	-4.41612200	-0.13209400
H	1.52213700	-6.48381400	-0.39265900
C	-2.05888300	3.05620900	-4.57068800
C	-2.53390700	1.88665100	-3.97524800
C	-1.65326700	1.05047300	-3.29164200
C	-0.29834300	1.39073600	-3.16891500
C	0.17341500	2.56250200	-3.76987600
C	-0.70470700	3.38521500	-4.47477600
H	-2.74190900	3.71166900	-5.10355700
H	-3.58644900	1.62599400	-4.01127000
H	-2.03267500	0.13529700	-2.85185400
H	1.21596000	2.83932100	-3.67007800
H	-0.33066700	4.29350000	-4.93920800
C	-0.18360000	-3.64718700	-4.53136000
C	0.15586600	-2.49157700	-5.24329000
C	0.46434800	-1.31529700	-4.56417500
C	0.46462000	-1.28647200	-3.15897800
C	0.09673600	-2.44047700	-2.45914300
C	-0.22310300	-3.61851700	-3.13834100
H	-0.43333100	-4.55932300	-5.06589000
H	0.16312900	-2.50464300	-6.32950400
H	0.68092100	-0.40920200	-5.12191200
H	0.04879700	-2.41366600	-1.37912800
H	-0.50443800	-4.50067300	-2.57171600
Pd	0.53826300	-0.03010500	0.20191900
C	-1.71358400	0.42729100	-0.02759400
C	-1.27965500	0.99048800	1.18194000
C	-0.53765500	0.14387100	2.03122800
H	-2.10353500	1.05984900	-0.81283900
H	-1.80415100	-0.64065100	-0.15330300
C	-0.56441300	-0.37251200	3.25298400
H	-1.45148400	-0.23959200	3.87131800
H	0.24043100	-0.98040700	3.65224000
C	-4.16203300	-0.68582700	0.17524800
C	-4.45342700	-0.41604600	-1.17894000
N	-4.63287500	1.47243000	-0.15422400
C	-3.74109000	-1.96876100	0.82029600
H	-3.09881800	-2.54558300	0.13889000
H	-3.12904300	-1.71059900	1.69659200

C	-4.88701200	-2.85124100	1.27272100
C	-5.08831900	-4.12355700	0.73050400
C	-5.77571600	-2.38216600	2.25134000
C	-6.15475100	-4.92196200	1.15506900
H	-4.40893700	-4.48789700	-0.03828100
C	-6.83688500	-3.17696200	2.67743400
H	-5.62005800	-1.38713700	2.66103000
C	-7.03090700	-4.45066300	2.13136800
H	-6.30073500	-5.90765200	0.72004900
H	-7.51918500	-2.80348300	3.43680000
H	-7.86187100	-5.06707100	2.46393400
C	-4.44857200	-1.39559200	-2.31180800
H	-3.45894800	-1.85850600	-2.44313700
H	-5.15859500	-2.21319900	-2.13540800
H	-4.71991400	-0.89444600	-3.24570400
N	-4.71202700	0.86987000	-1.40089700
C	-4.68140500	2.86837500	-0.05388100
C	-4.44806900	3.65829900	-1.19157800
C	-4.93909500	3.49075500	1.18037800
C	-4.46436700	5.04683300	-1.09020800
H	-4.25188500	3.16680100	-2.13507800
C	-4.94125400	4.88221700	1.26304800
H	-5.11131200	2.87220700	2.05073400
C	-4.70552200	5.67182300	0.13562600
H	-4.27743200	5.64452200	-1.97915500
H	-5.13964200	5.35227000	2.22333100
H	-4.71219400	6.75581900	0.20998700
C	-4.30526000	0.54656200	0.87889500
O	-4.13813100	0.82359900	2.09046800
C	-1.42244600	2.46911800	1.43271100
H	-0.75860300	2.79966700	2.23497600
H	-1.21719700	3.04783800	0.52788100
H	-2.45617200	2.64524600	1.74395100

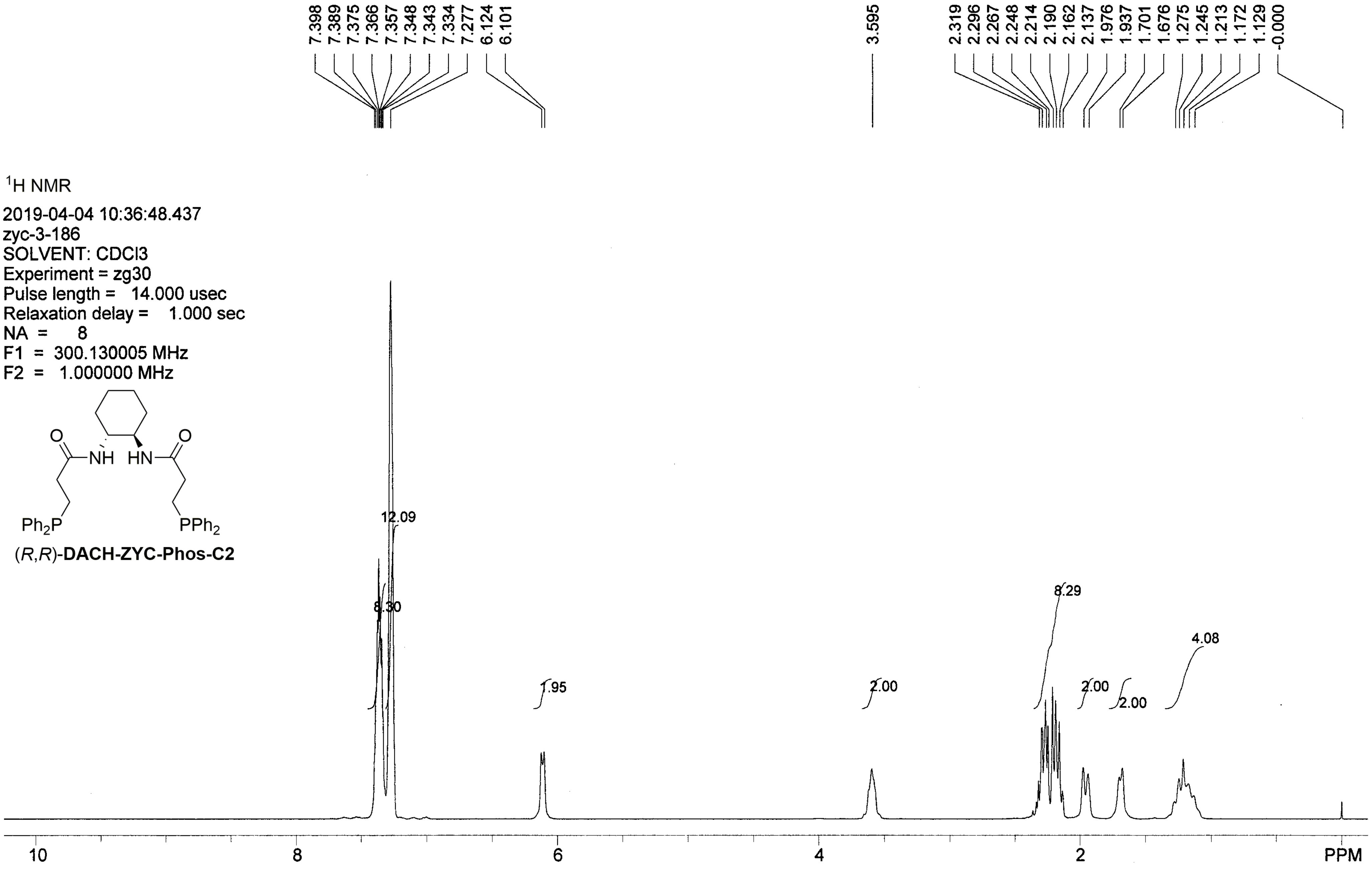

(*R*,*R*)-**DACH-ZYC-Phos-C2**

^{13}C NMR

2019-04-04 10:41:18.046
zyc-3-186
SOLVENT: CDCl3
Experiment = zgpg30
Pulse length = 9.500 usec
Relaxation delay = 2.000 sec
NA = 53
F1 = 75.467751 MHz
F2 = 1.000000 MHz

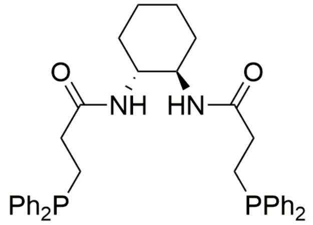

(*R*,*R*)-**DACH-ZYC-Phos-C2**

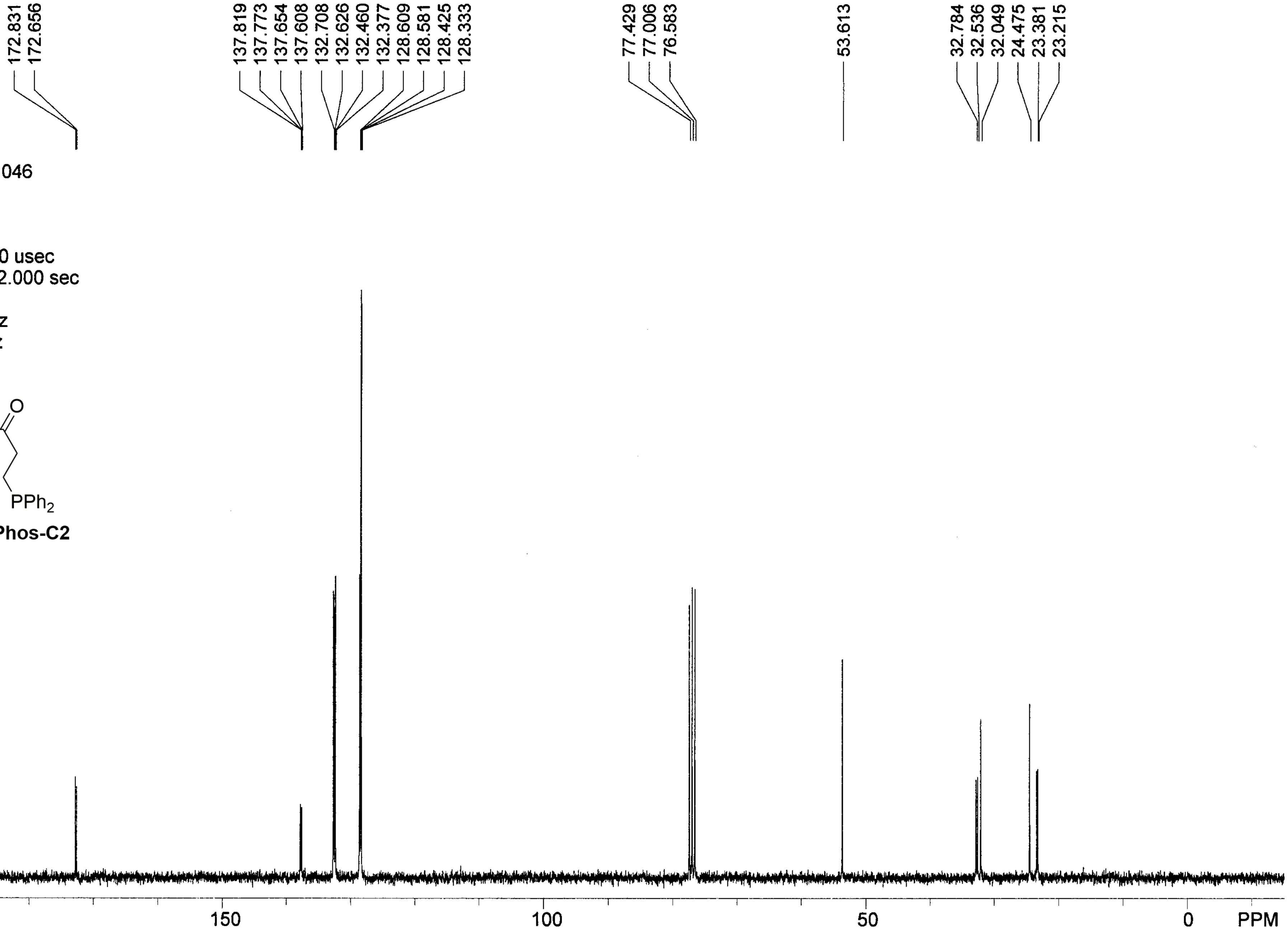

^{13}C NMR

2019-04-04 10:41:18.046
zyc-3-186
SOLVENT: CDCl3
Experiment = zgpg30
Pulse length = 9.500 usec
Relaxation delay = 2.000 sec
NA = 53
F1 = 75.467751 MHz
F2 = 1.000000 MHz

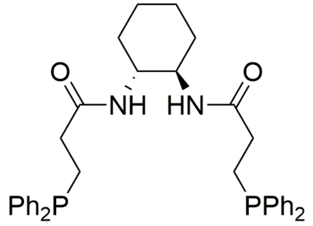

(*R*,*R*)-**DACH-ZYC-Phos-C2**

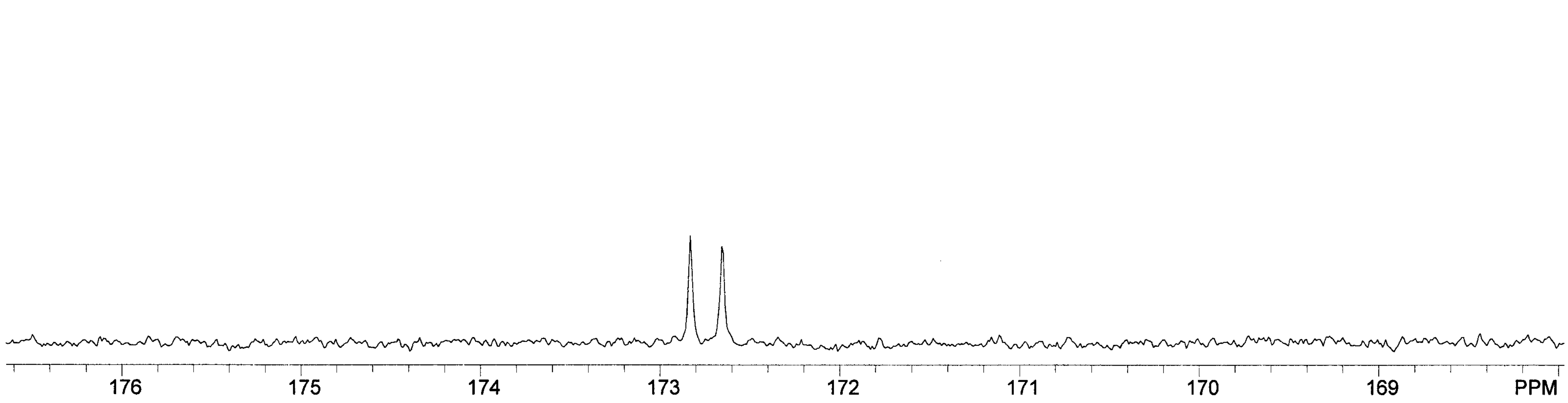

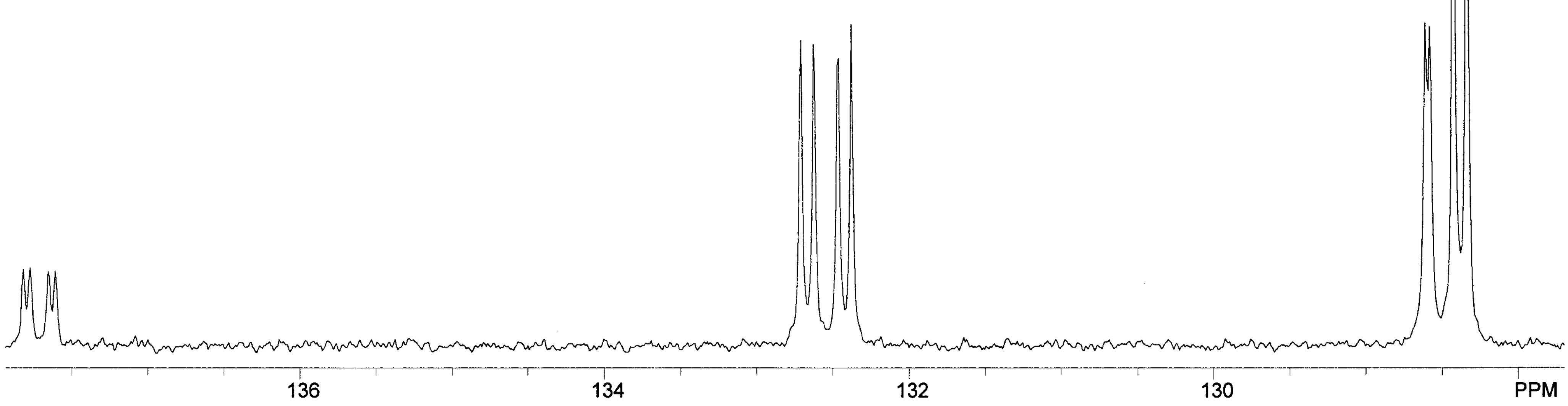

^{13}C NMR

2019-04-04 10:41:18.046
zyc-3-186
SOLVENT: CDCl3
Experiment = zgpg30
Pulse length = 9.500 usec
Relaxation delay = 2.000 sec
NA = 53
F1 = 75.467751 MHz
F2 = 1.000000 MHz

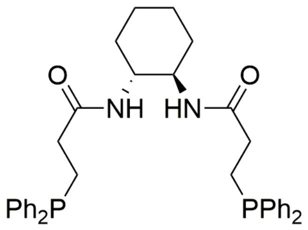

(*R*,*R*)-**DACH-ZYC-Phos-C2**

77.429
77.006
76.583
53.613
32.784
32.536
32.049
24.475
23.381
23.215

^{13}C NMR

2019-04-04 10:41:18.046
zyc-3-186
SOLVENT: CDCl3
Experiment = zgpg30
Pulse length = 9.500 usec
Relaxation delay = 2.000 sec
NA = 53
F1 = 75.467751 MHz
F2 = 1.000000 MHz

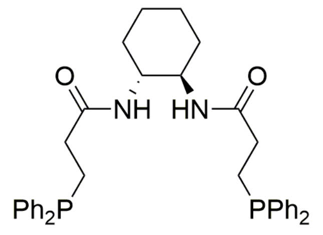

(*R*,*R*)-**DACH-ZYC-Phos-C2**

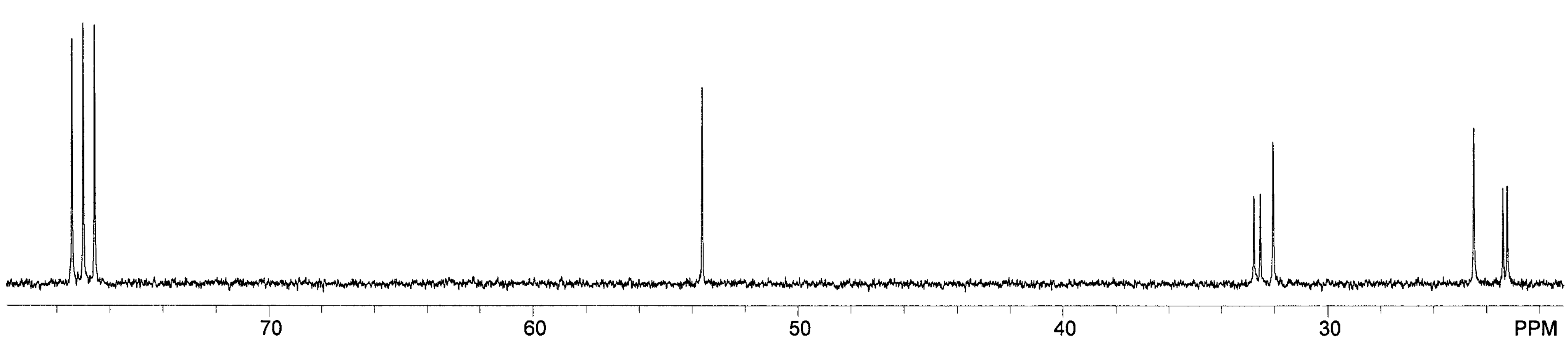

^{31}P NMR
2019-04-04 13:45:05.234
zyc-3-186
SOLVENT: CDCl3
Experiment = zgpg30
Pulse length = 9.200 usec
Relaxation delay = 2.000 sec
NA = 16
F1 = 121.494850 MHz
F2 = 1.000000 MHz

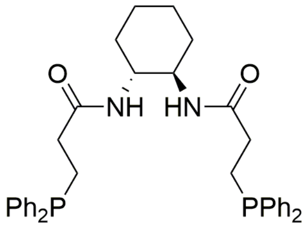

(*R*,*R*)-**DACH-ZYC-Phos-C2**

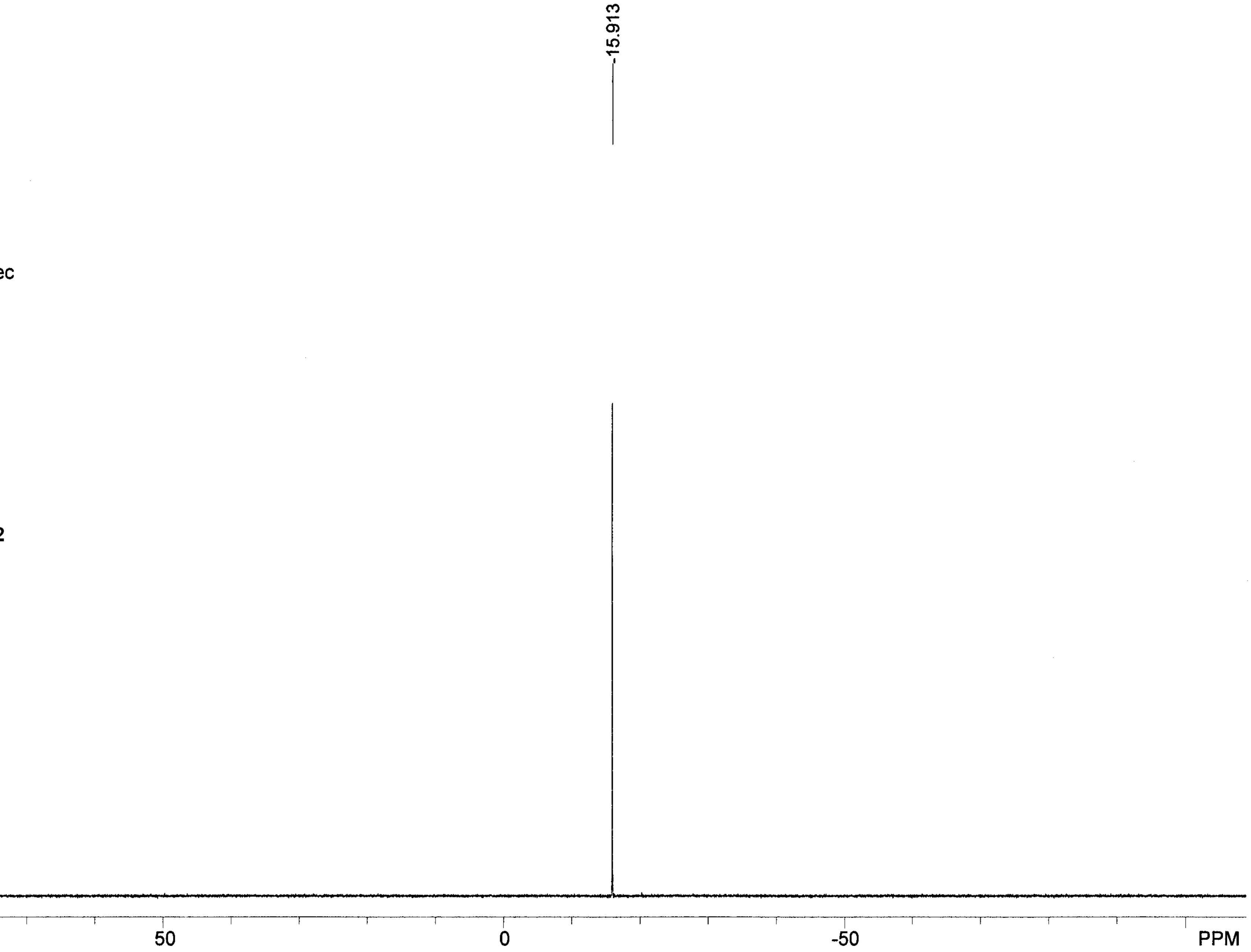

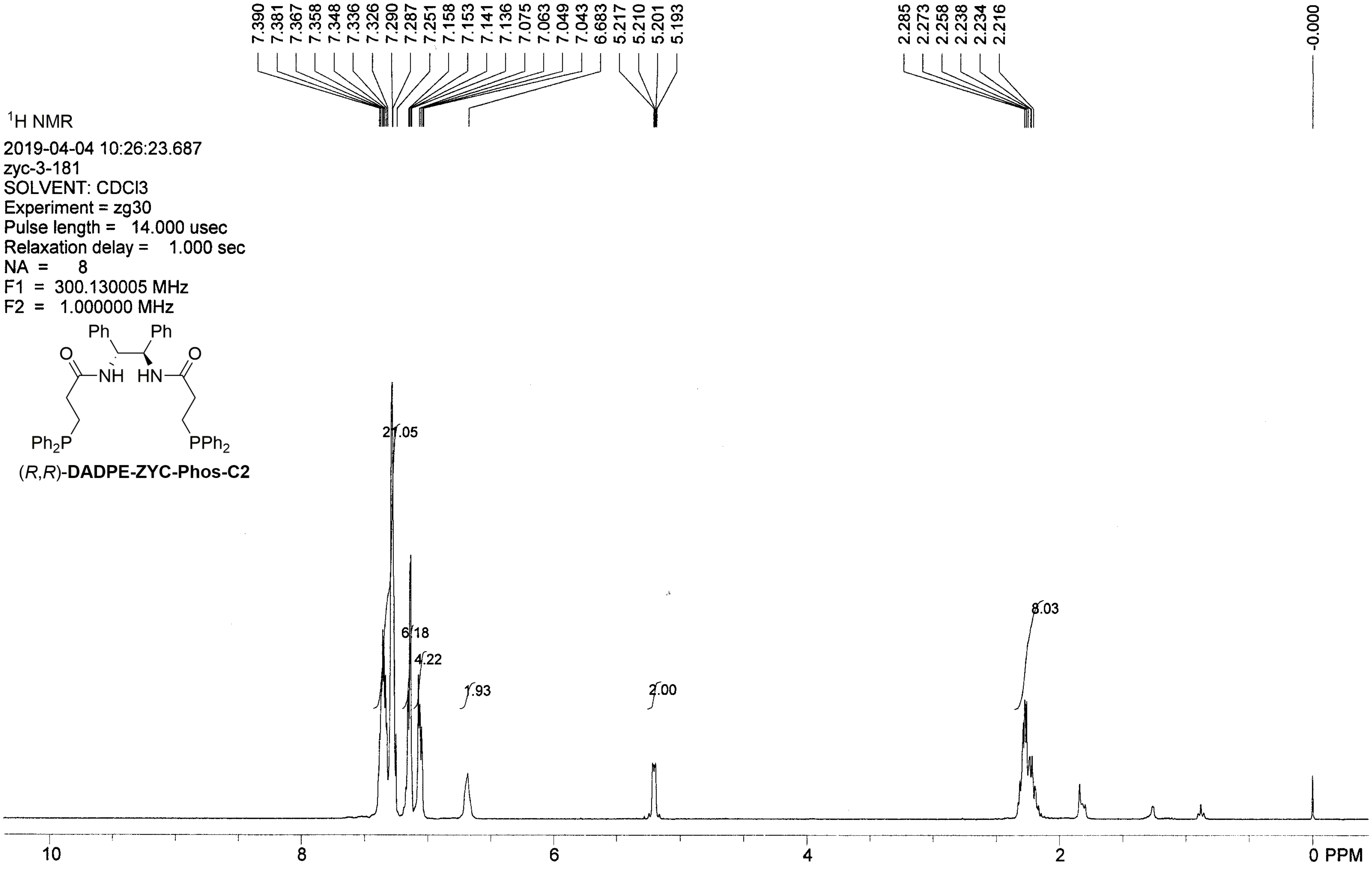

7.390 7.381 7.367 7.358 7.348 7.336 7.326 7.290 7.287 7.251 7.158 7.153 7.141 7.136 7.075 7.063 7.049 7.043 6.683 5.217 5.210 5.201 5.193
2.285 2.273 2.258 2.238 2.234 2.216
-0.000
1H NMR
2019-04-04 10:26:23.687
zyc-3-181
SOLVENT: CDCl3
Experiment = zg30
Pulse length = 14.000 usec
Relaxation delay = 1.000 sec
NA = 8
F1 = 300.130005 MHz
F2 = 1.000000 MHz
Ph
Ph
O
O
NH
HN
Ph2P
PPh2
(R,R)-DADPE-ZYC-Phos-C2
21.05
6.18
4.22
1.93
2.00
8.03
10
8
6
4
2
0 PPM

2019-04-10 09:34:33.578
zyc-3-181purity
SOLVENT: CDCl3
Experiment = zg30
Pulse length = 14.000 usec
Relaxation delay = 1.000 sec
NA = 8
F1 = 300.130005 MHz
F2 = 1.000000 MHz

purity = 98%

11.2 mg sample was added with 5 μL

$$\text{purity} = \frac{\frac{29.22}{200} \times \frac{5}{46} \times 692.8}{11.2} \times 100\%$$

= 98%

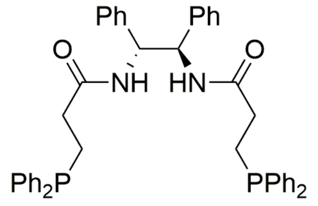

(*R*,*R*)-**DADPE-ZYC-Phos-C2**

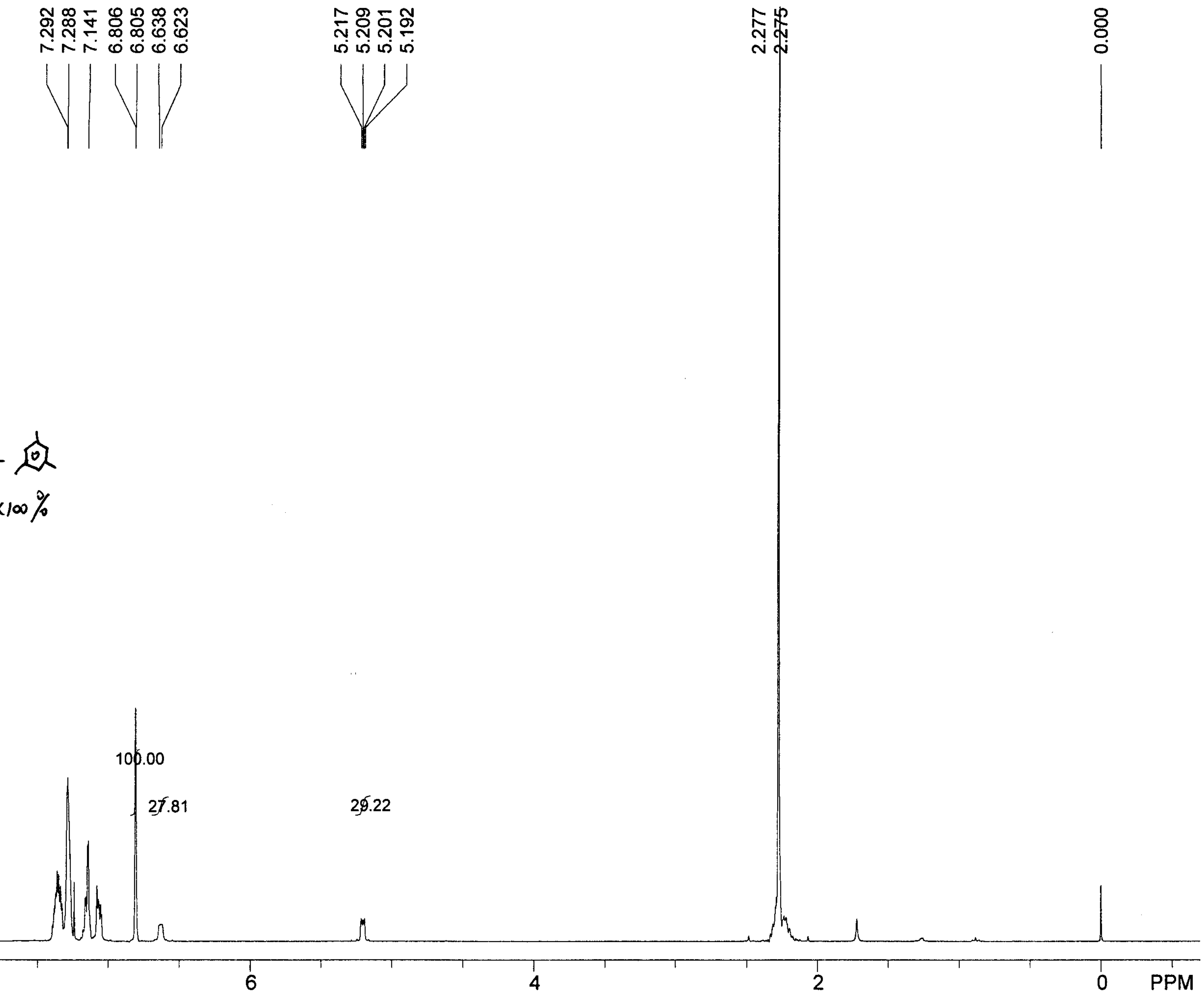

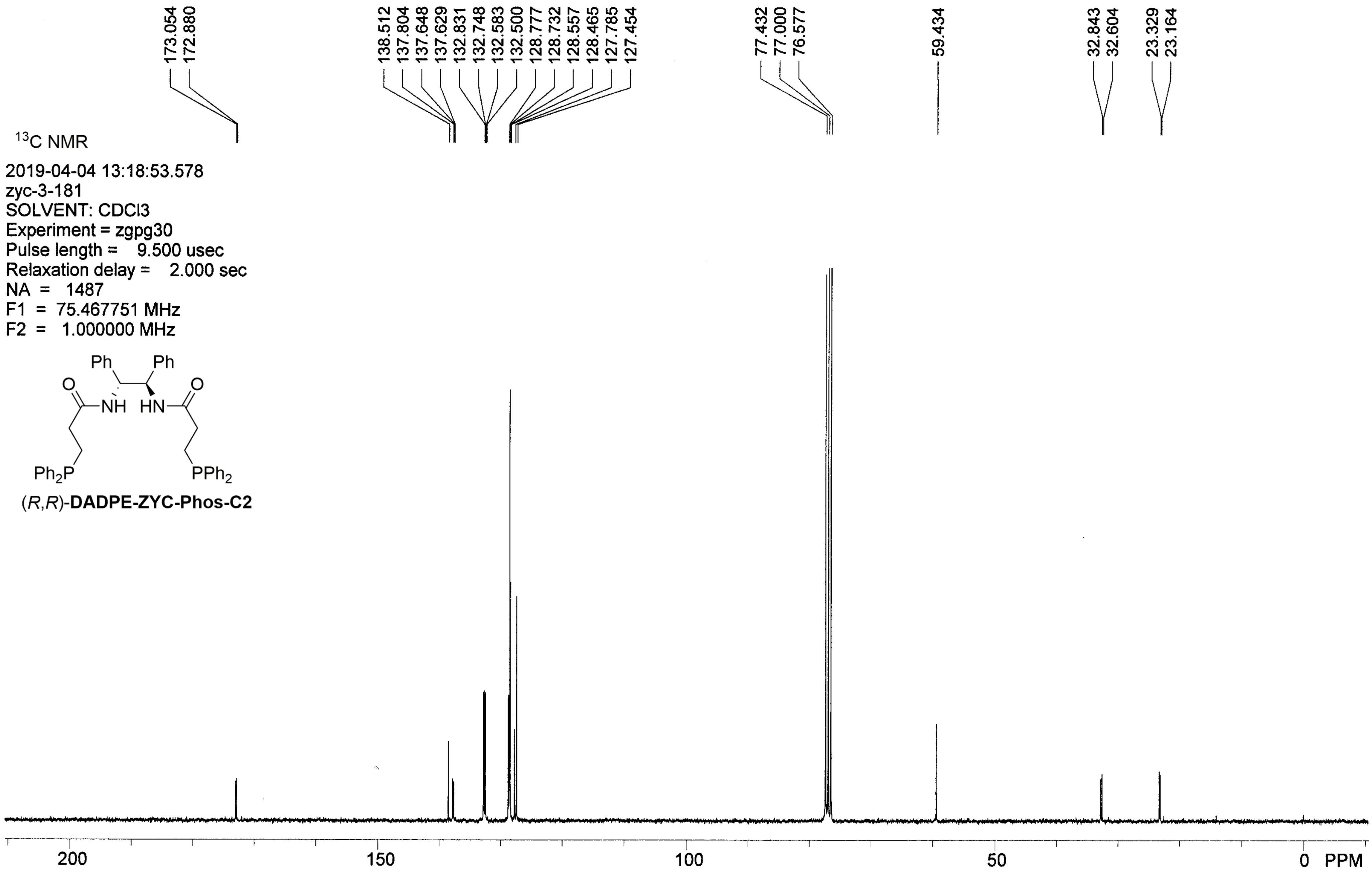

^{13}C NMR

2019-04-04 13:18:53.578
zyc-3-181
SOLVENT: CDCl3
Experiment = zgpg30
Pulse length = 9.500 usec
Relaxation delay = 2.000 sec
NA = 1487
F1 = 75.467751 MHz
F2 = 1.000000 MHz

(*R*,*R*)-**DADPE-ZYC-Phos-C2**

^{13}C NMR
2019-04-04 13:18:53.578
zyc-3-181
SOLVENT: CDCl3
Experiment = zgpg30
Pulse length = 9.500 usec
Relaxation delay = 2.000 sec
NA = 1487
F1 = 75.467751 MHz
F2 = 1.000000 MHz

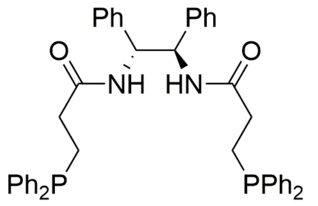

(*R*,*R*)-**DADPE-ZYC-Phos-C2**

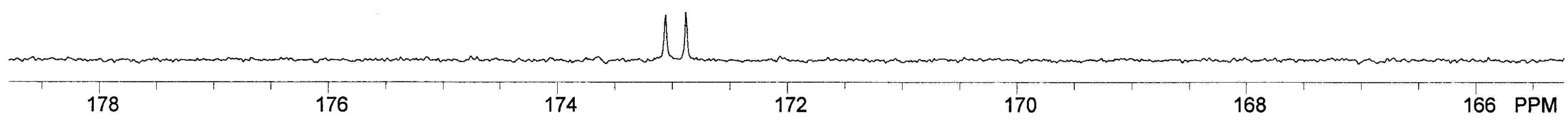

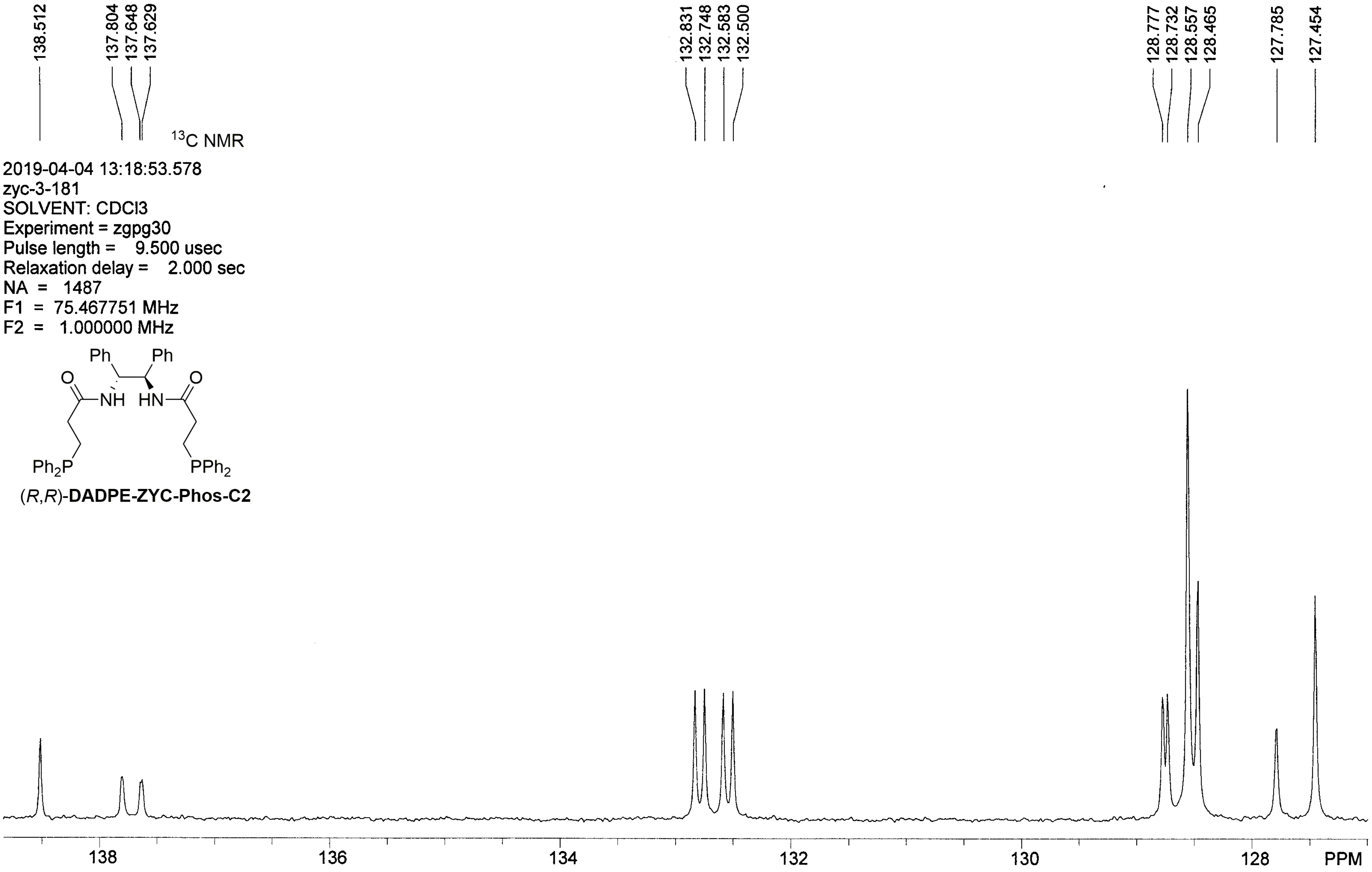

^{13}C NMR

2019-04-04 13:18:53.578
zyc-3-181
SOLVENT: CDCl3
Experiment = zgpg30
Pulse length = 9.500 usec
Relaxation delay = 2.000 sec
NA = 1487
F1 = 75.467751 MHz
F2 = 1.000000 MHz

(*R*,*R*)-**DADPE-ZYC-Phos-C2**

^{13}C NMR

2019-04-04 13:18:53.578
zyc-3-181
SOLVENT: CDCl3
Experiment = zgpg30
Pulse length = 9.500 usec
Relaxation delay = 2.000 sec
NA = 1487
F1 = 75.467751 MHz
F2 = 1.000000 MHz

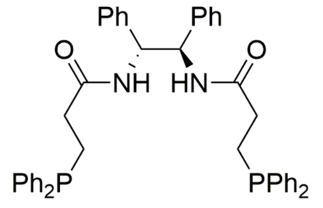

(*R*,*R*)-**DADPE-ZYC-Phos-C2**

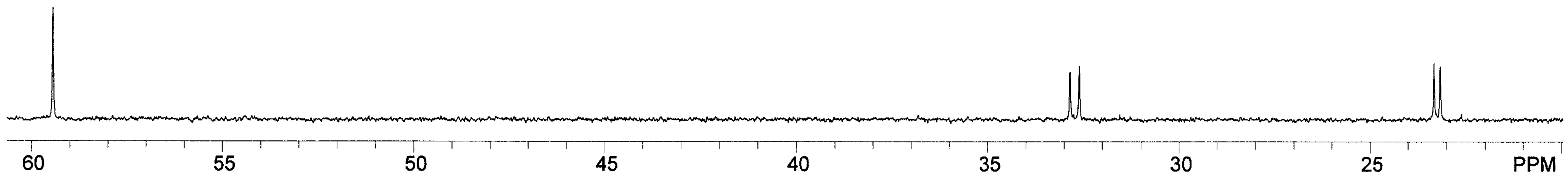

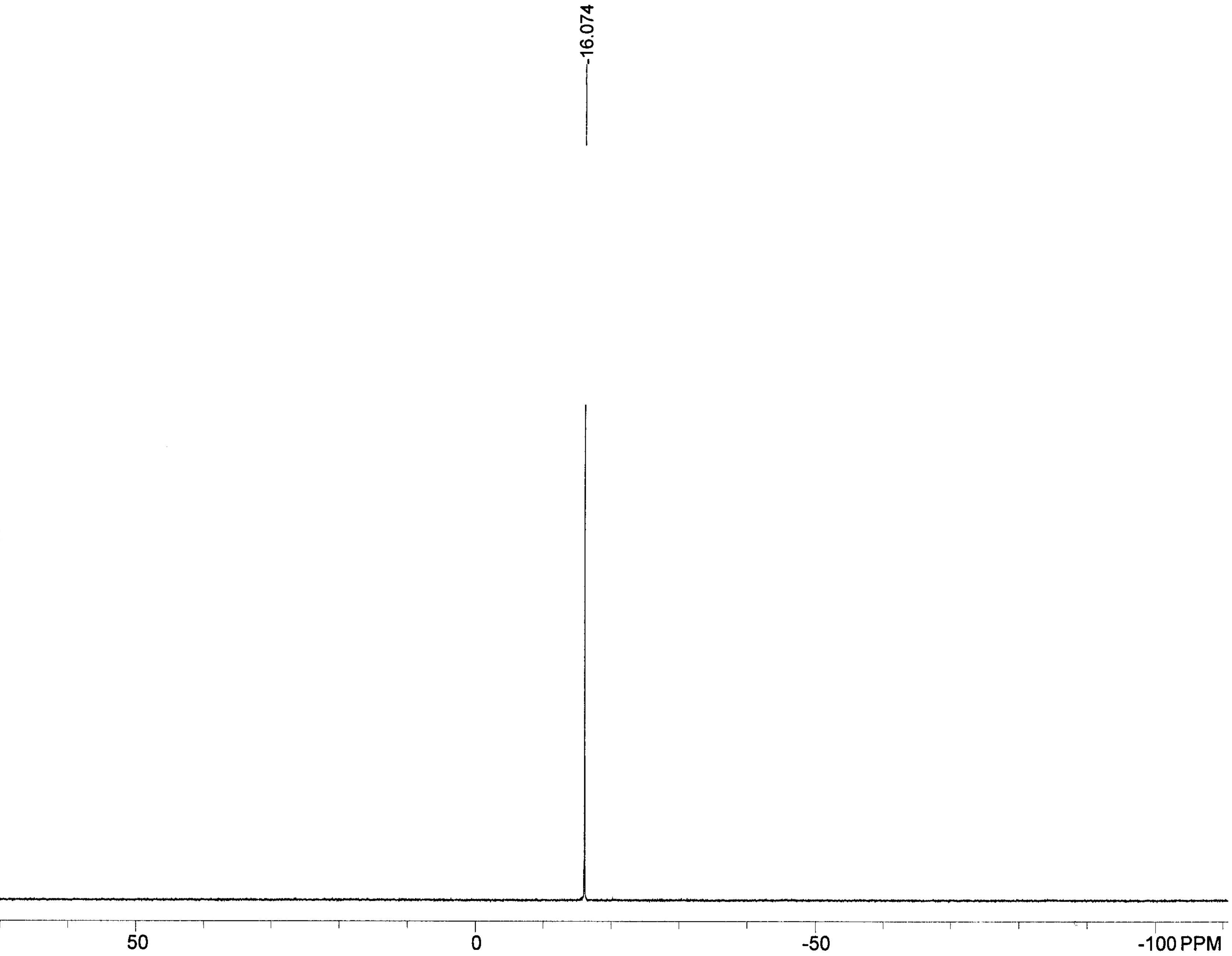

^{31}P NMR

2019-04-04 13:39:24.406
zyc-3-181
SOLVENT: CDCl3
Experiment = zgpg30
Pulse length = 9.200 usec
Relaxation delay = 2.000 sec
NA = 16
F1 = 121.494850 MHz
F2 = 1.000000 MHz

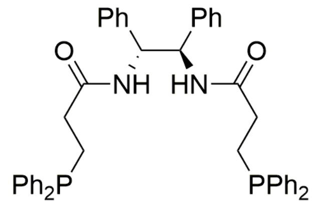

(*R*,*R*)-**DADPE-ZYC-Phos-C2**

^{1}H NMR

2019-04-19 20:38:12.562
zyc-4-15
SOLVENT: CDCl3
Experiment = zg30
Pulse length = 14.000 usec
Relaxation delay = 1.000 sec
NA = 8
F1 = 300.130005 MHz
F2 = 1.000000 MHz

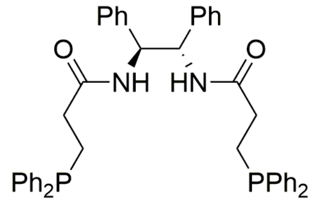

(*S*,*S*)-**DADPE-ZYC-Phos-C2**

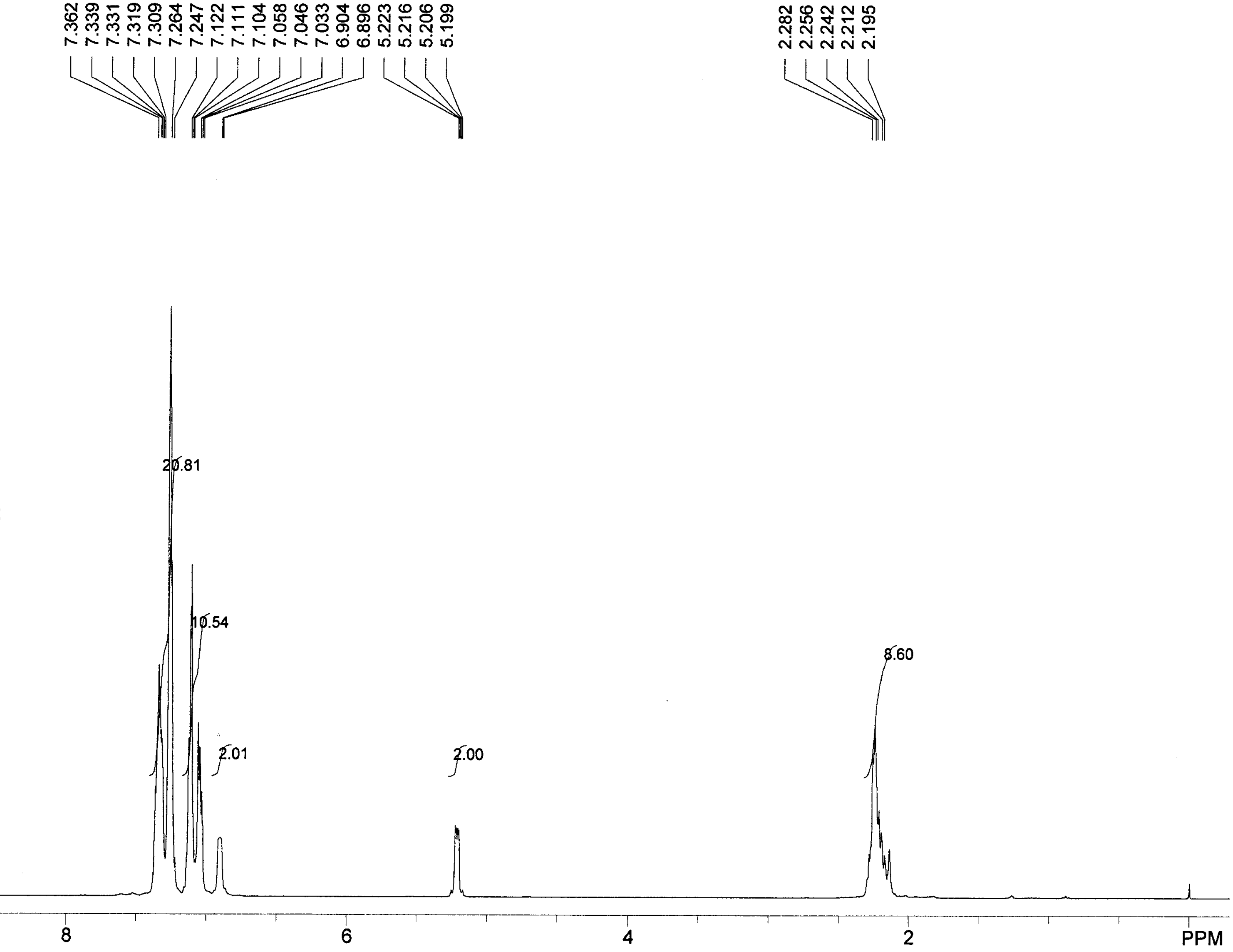

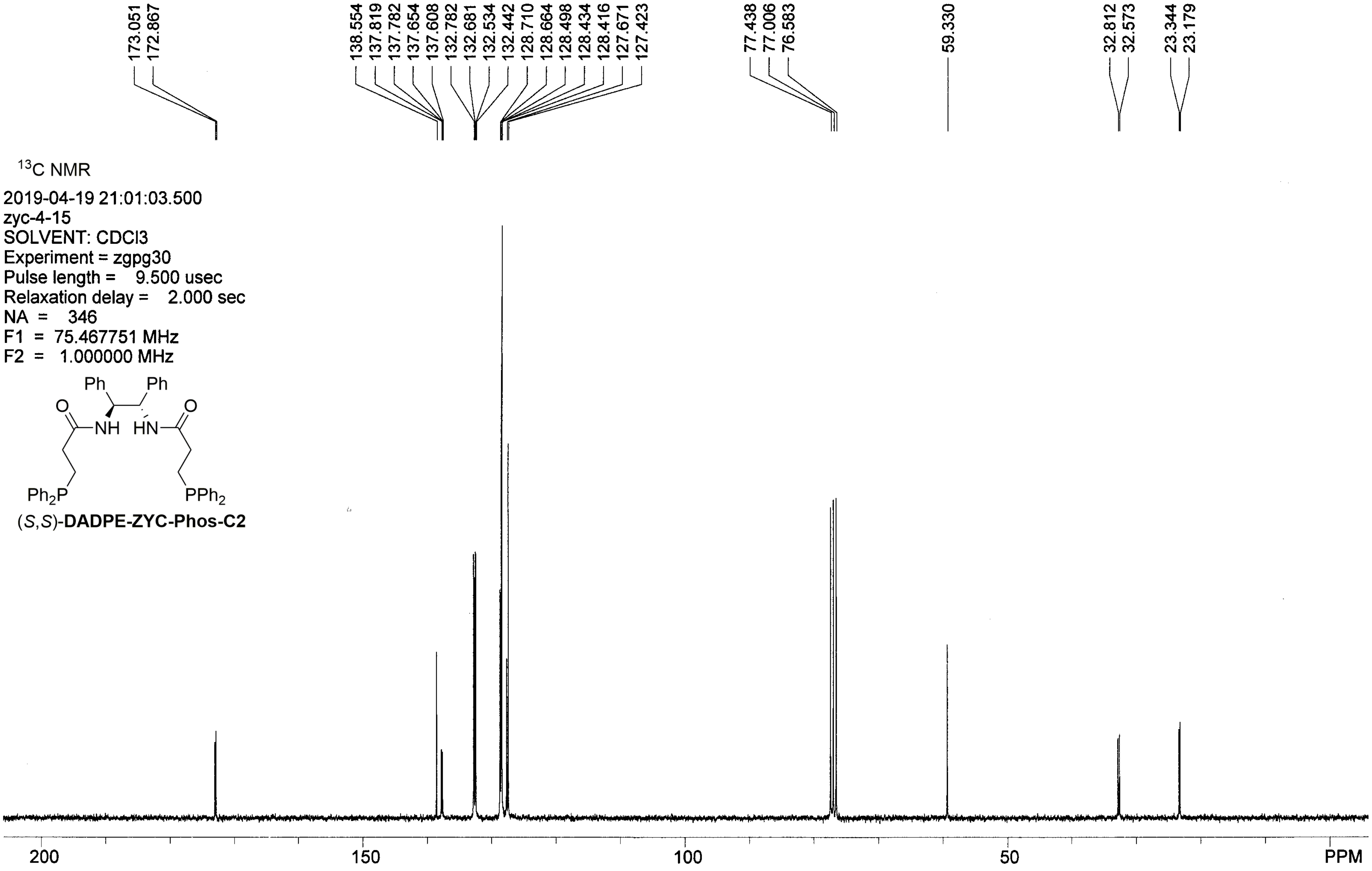

^{13}C NMR

2019-04-19 21:01:03.500
zyc-4-15
SOLVENT: CDCl3
Experiment = zgpg30
Pulse length = 9.500 usec
Relaxation delay = 2.000 sec
NA = 346
F1 = 75.467751 MHz
F2 = 1.000000 MHz

(*S*,*S*)-**DADPE-ZYC-Phos-C2**

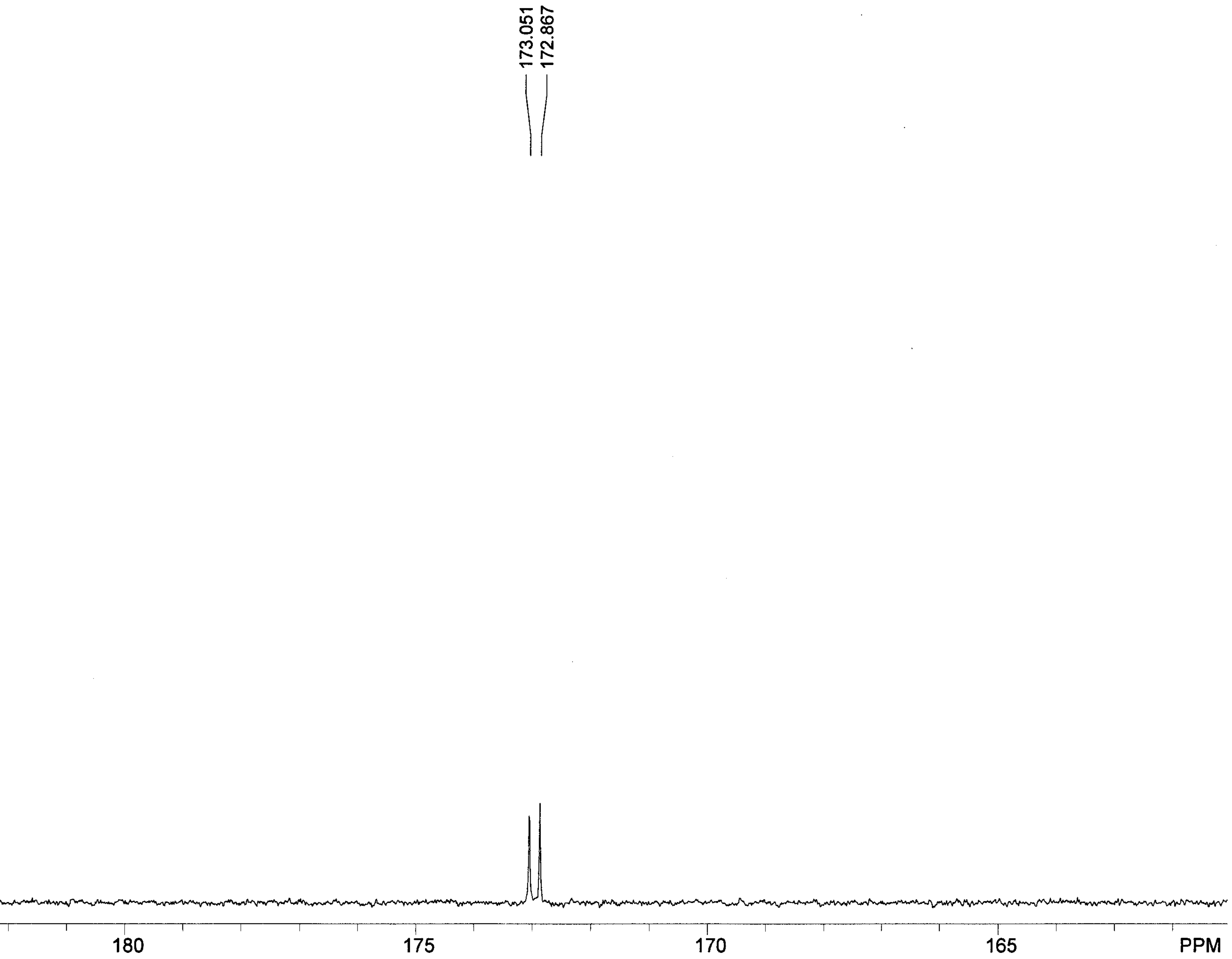

^{13}C NMR

2019-04-19 21:01:03.500
zyc-4-15
SOLVENT: CDCl3
Experiment = zgpg30
Pulse length = 9.500 usec
Relaxation delay = 2.000 sec
NA = 346
F1 = 75.467751 MHz
F2 = 1.000000 MHz

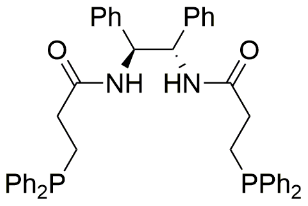

(*S*,*S*)-**DADPE-ZYC-Phos-C2**

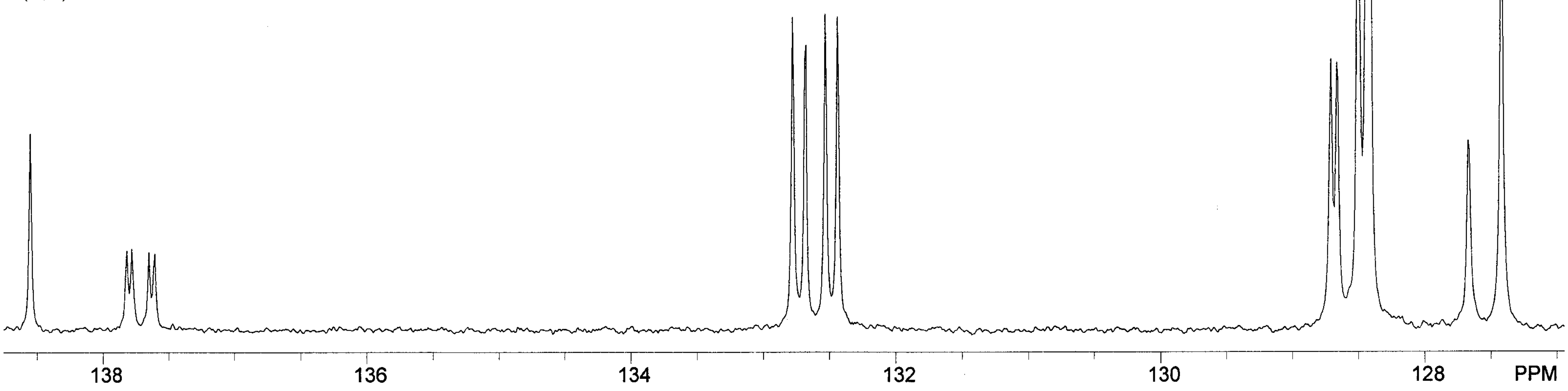

^{13}C NMR
2019-04-19 21:01:03.500
zyc-4-15
SOLVENT: CDCl3
Experiment = zgpg30
Pulse length = 9.500 usec
Relaxation delay = 2.000 sec
NA = 346
F1 = 75.467751 MHz
F2 = 1.000000 MHz

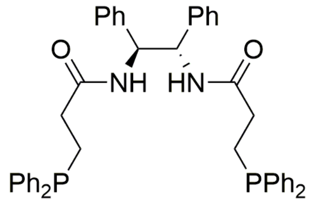

(*S*,*S*)-**DADPE-ZYC-Phos-C2**

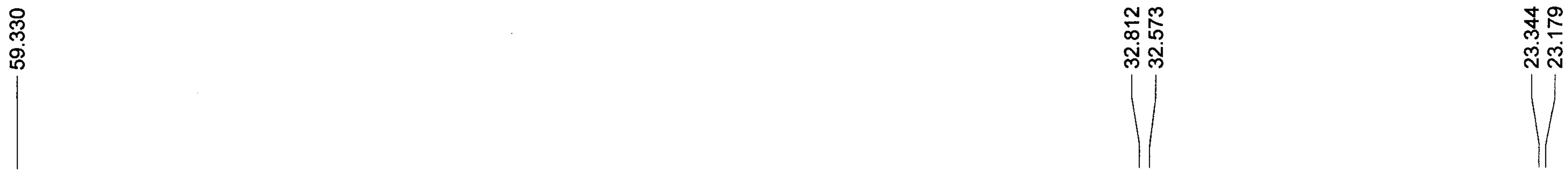

^{13}C NMR
2019-04-19 21:01:03.500
zyc-4-15
SOLVENT: CDCl3
Experiment = zgpg30
Pulse length = 9.500 usec
Relaxation delay = 2.000 sec
NA = 346
F1 = 75.467751 MHz
F2 = 1.000000 MHz

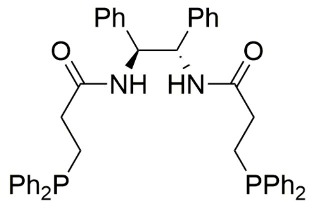

(*S*,*S*)-**DADPE-ZYC-Phos-C2**

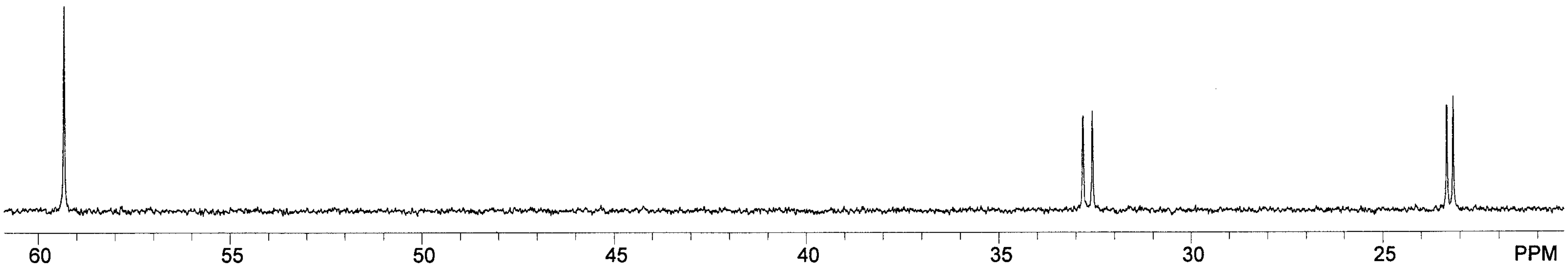

^{31}P NMR
2019-04-19 20:32:02.031
zyc-4-15
SOLVENT: CDCl3
Experiment = zgpg30
Pulse length = 9.200 usec
Relaxation delay = 2.000 sec
NA = 16
F1 = 121.494850 MHz
F2 = 1.000000 MHz

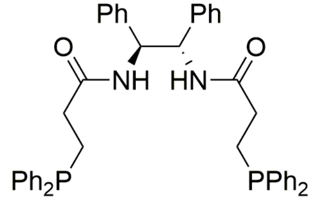

(*S*,*S*)-**DADPE-ZYC-Phos-C2**

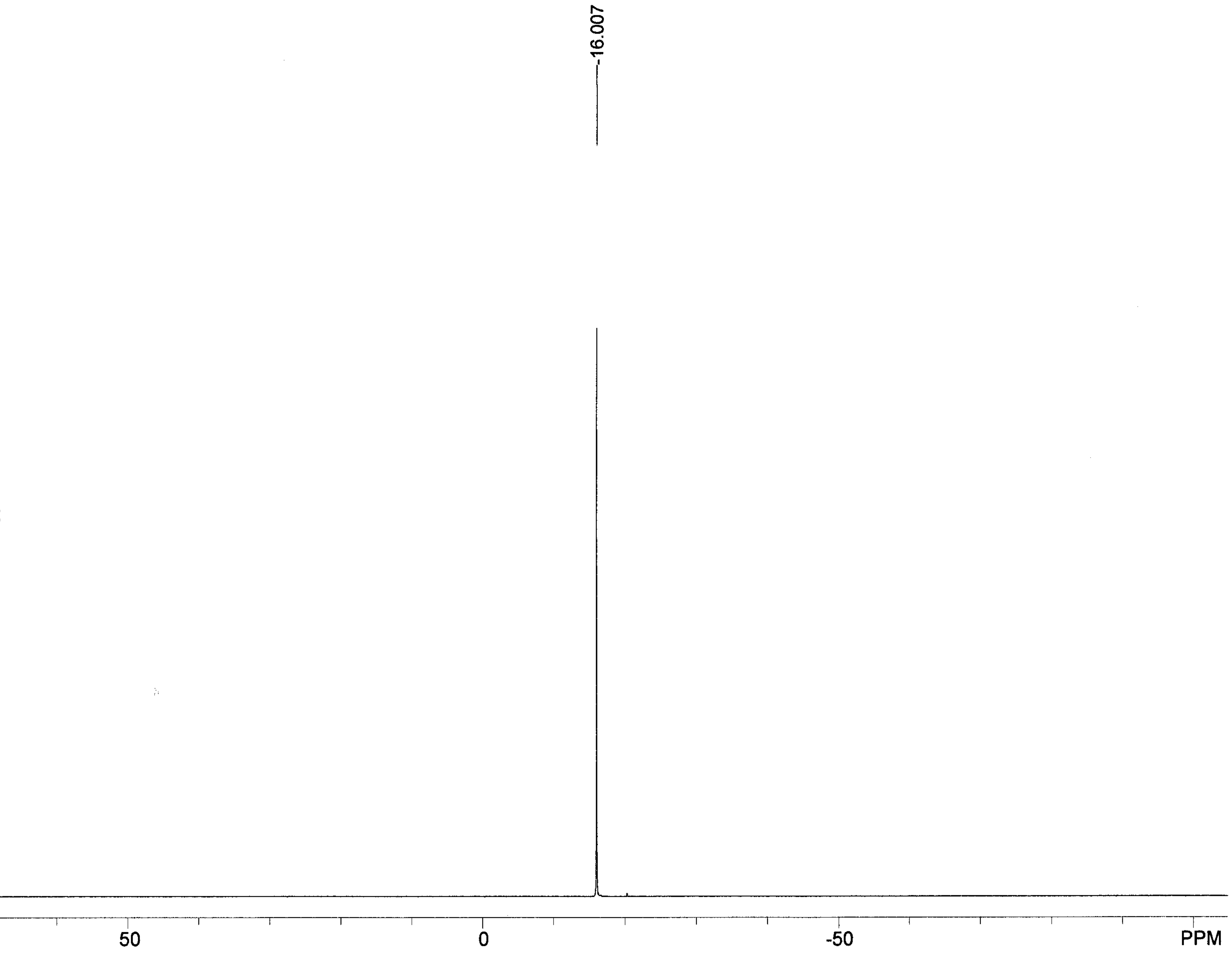

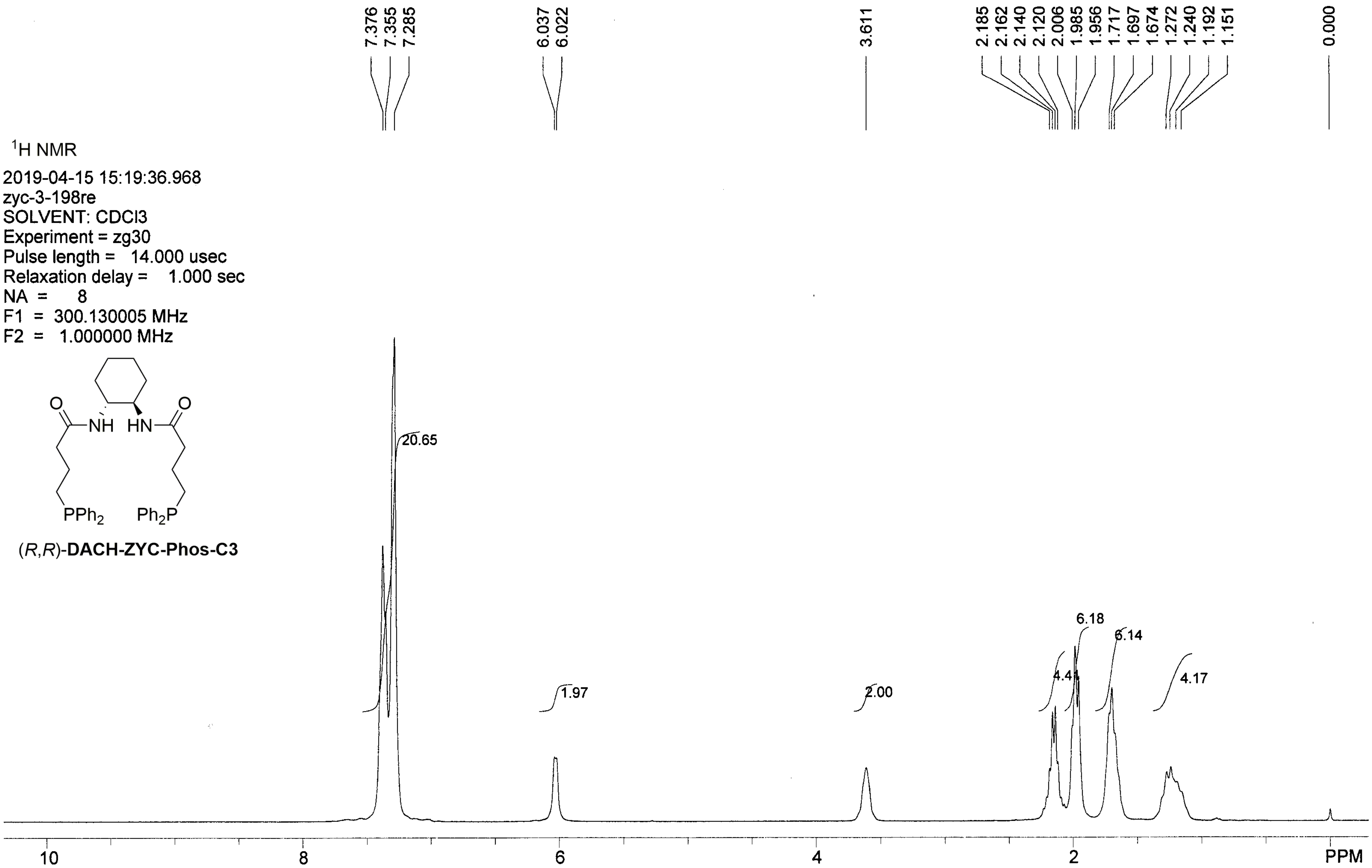

(*R*,*R*)-**DACH-ZYC-Phos-C3**

^{13}C NMR
2019-04-15 15:33:38.734
zyc-3-198re
SOLVENT: CDCl3
Experiment = zgpg30
Pulse length = 9.500 usec
Relaxation delay = 2.000 sec
NA = 210
F1 = 75.467751 MHz
F2 = 1.000000 MHz

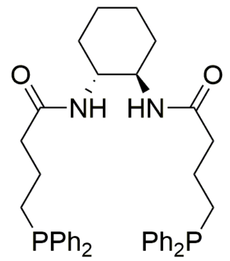

(*R*,*R*)-**DACH-ZYC-Phos-C3**

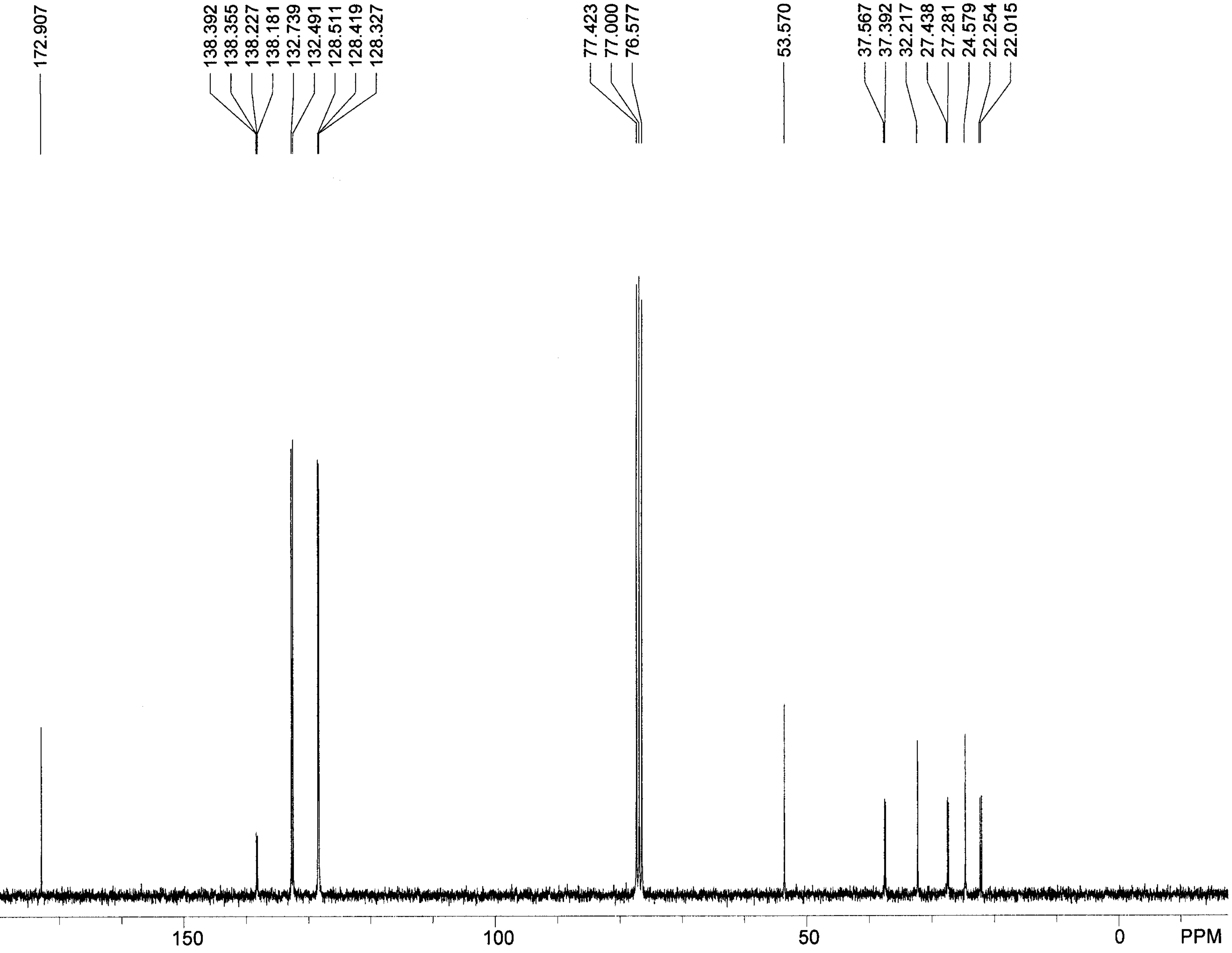

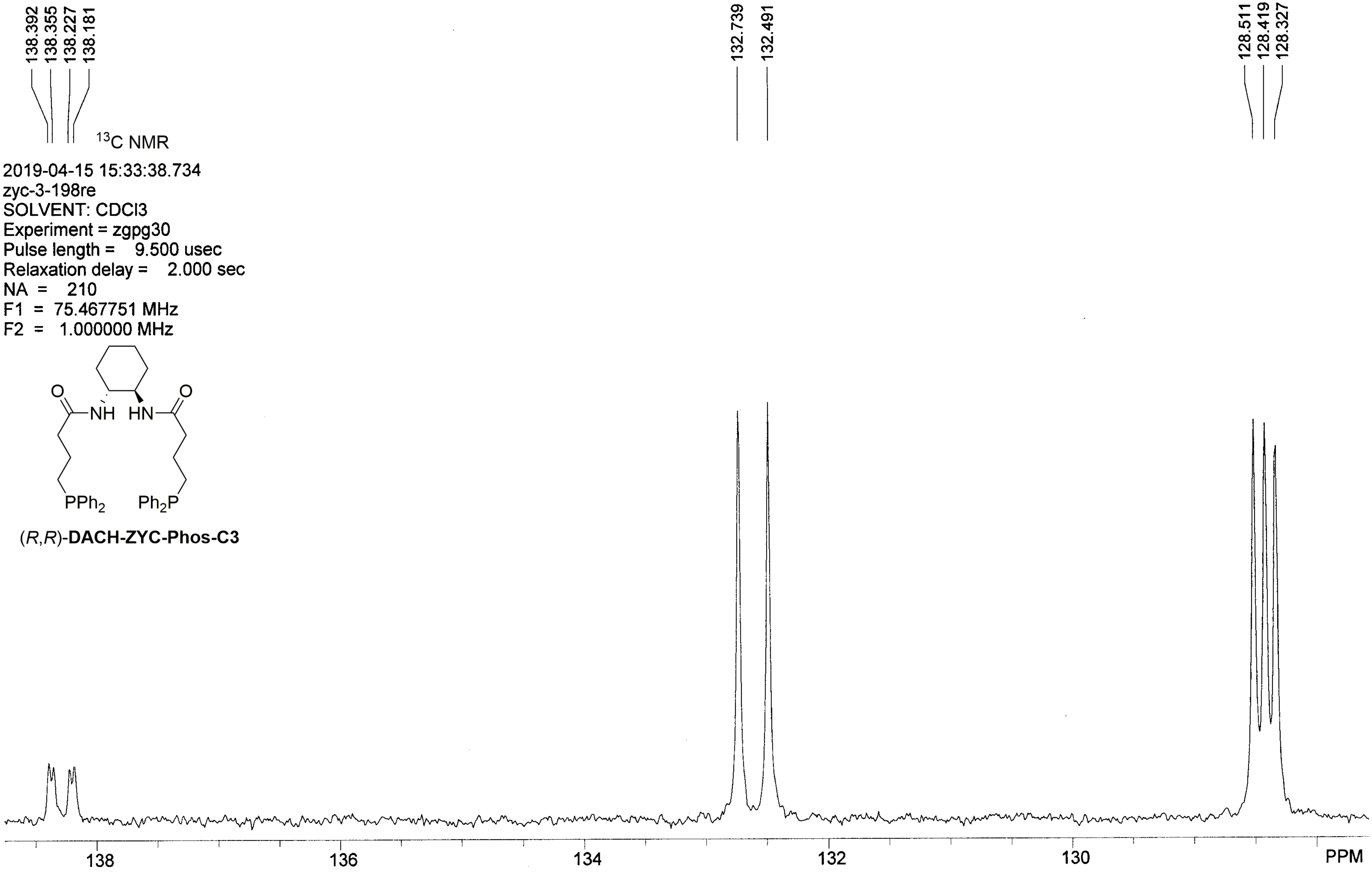

^{13}C NMR

2019-04-15 15:33:38.734
zyc-3-198re
SOLVENT: CDCl3
Experiment = zgpg30
Pulse length = 9.500 usec
Relaxation delay = 2.000 sec
NA = 210
F1 = 75.467751 MHz
F2 = 1.000000 MHz

(*R*,*R*)-**DACH-ZYC-Phos-C3**

^{13}C NMR

2019-04-15 15:33:38.734
zyc-3-198re
SOLVENT: CDCl3
Experiment = zgpg30
Pulse length = 9.500 usec
Relaxation delay = 2.000 sec
NA = 210
F1 = 75.467751 MHz
F2 = 1.000000 MHz

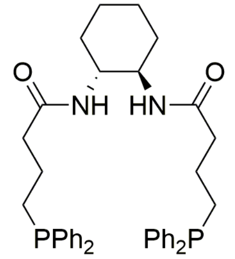

(*R*,*R*)-**DACH-ZYC-Phos-C3**

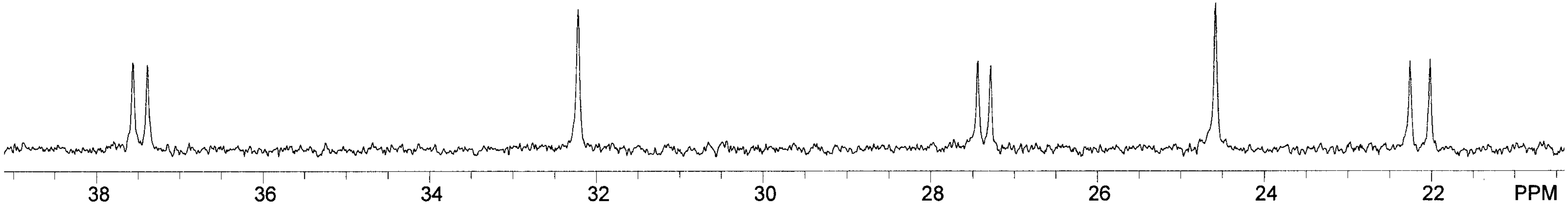

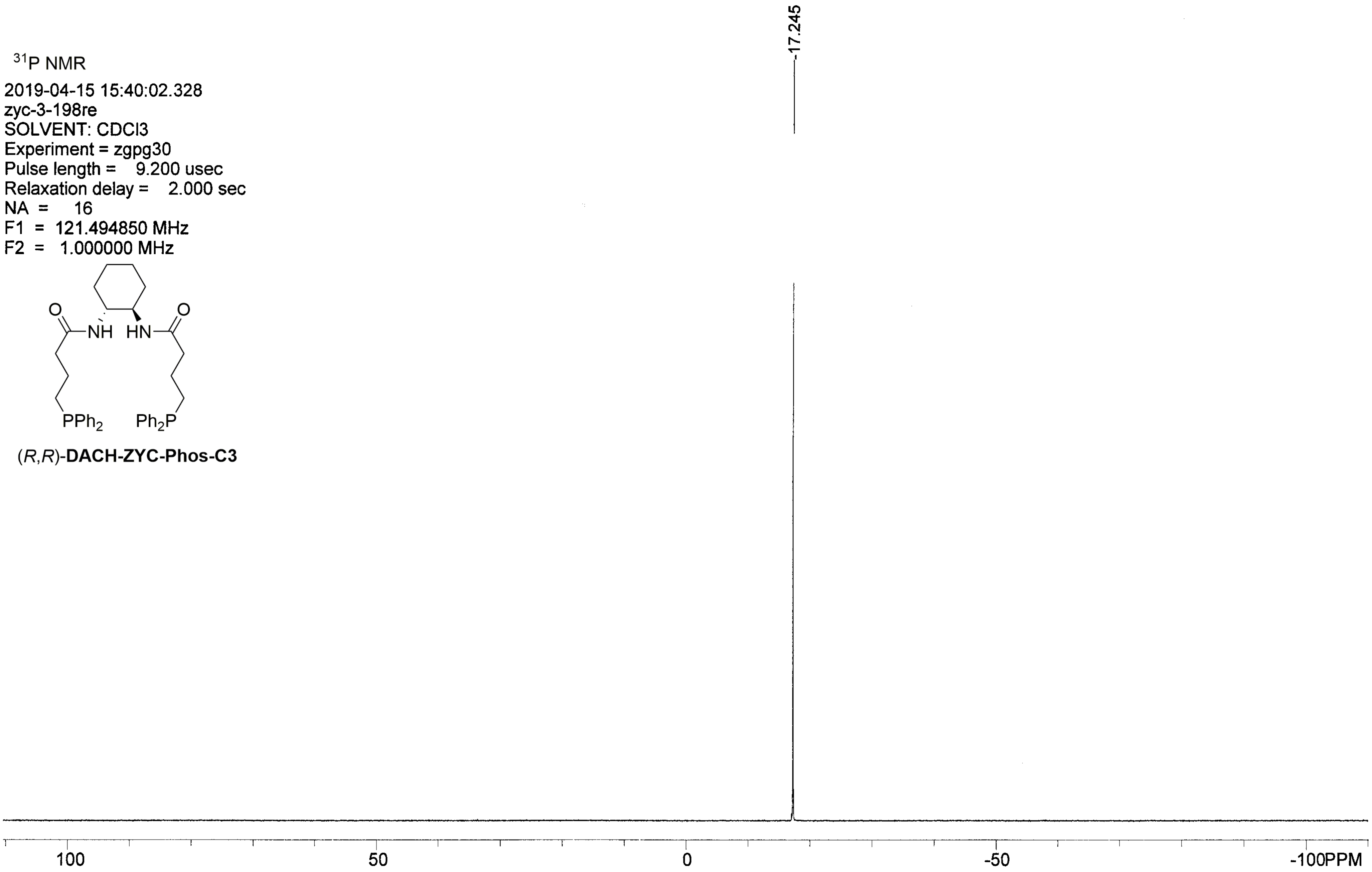

(*R*,*R*)-**DACH-ZYC-Phos-C3**

^{1}H NMR

2019-04-16 17:22:21.578
zyc-4-7
SOLVENT: CDCl3
Experiment = zg30
Pulse length = 14.000 usec
Relaxation delay = 1.000 sec
NA = 8
F1 = 300.130005 MHz
F2 = 1.000000 MHz

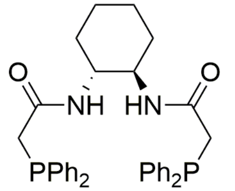

(*R*,*R*)-**DACH-ZYC-Phos-C1**

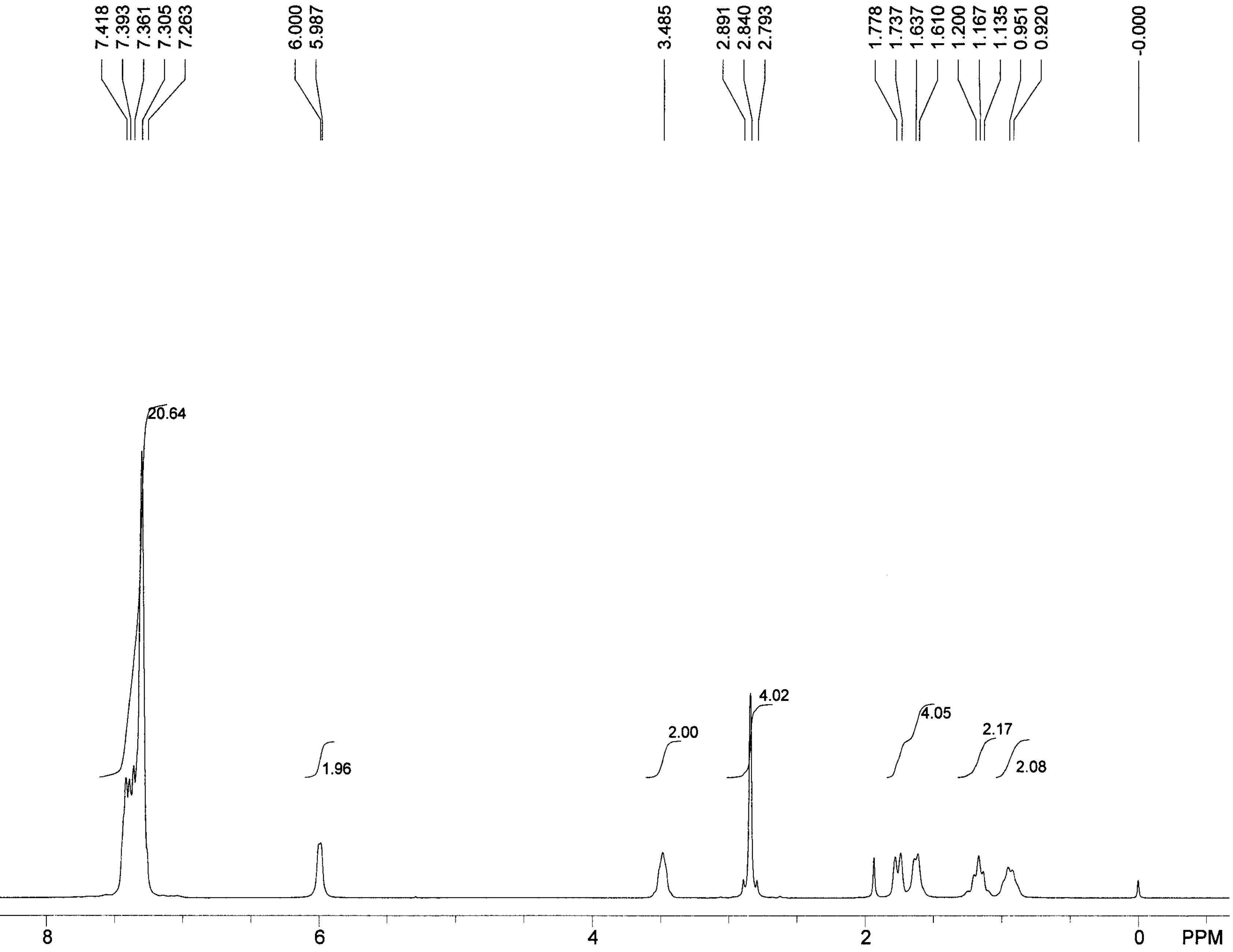

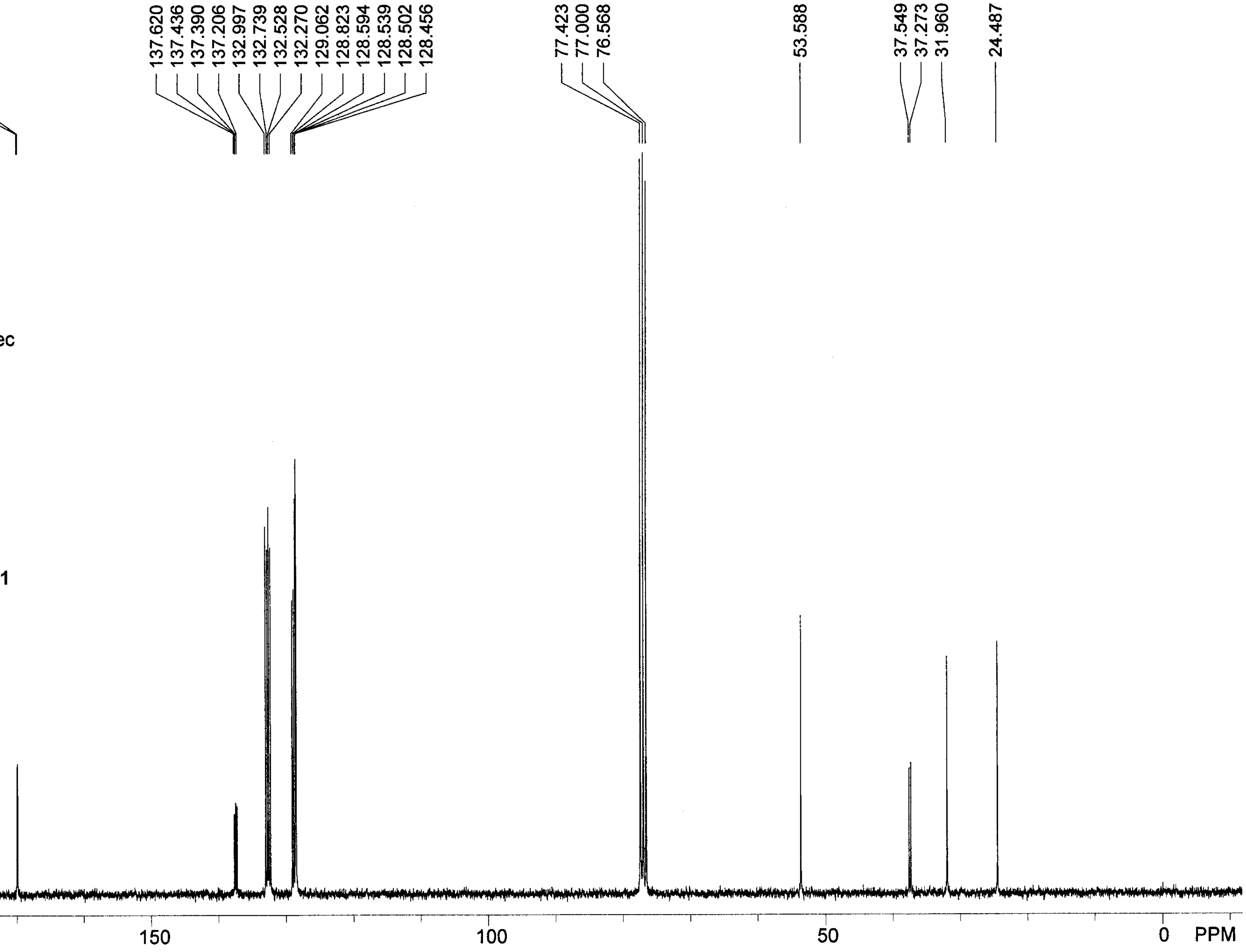

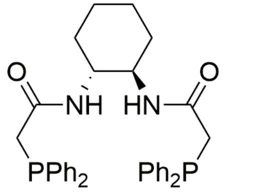

(*R*,*R*)-**DACH-ZYC-Phos-C1**

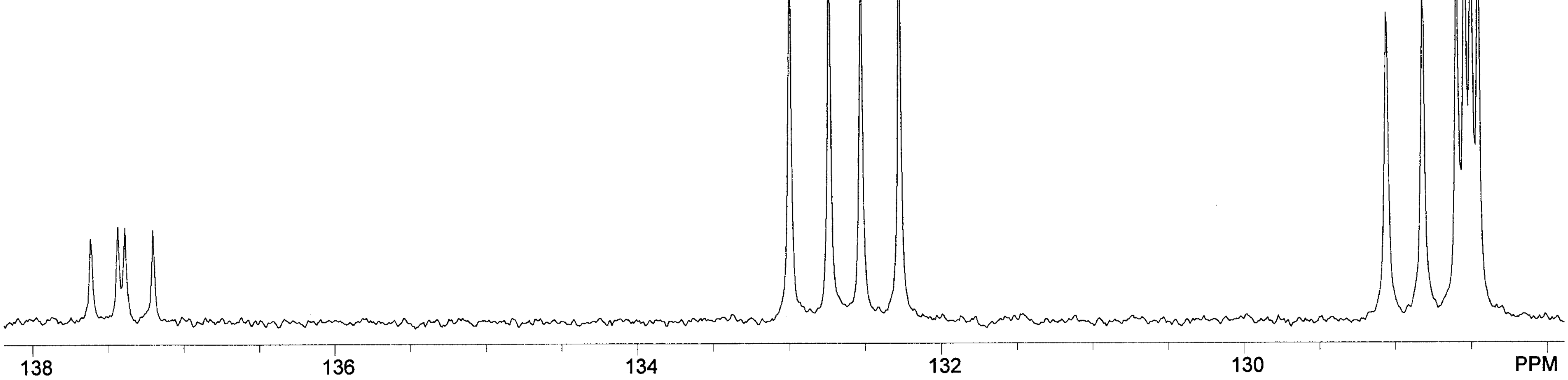

^{13}C NMR

2019-04-16 18:46:27.593
zyc-4-7
SOLVENT: CDCl3
Experiment = zgpg30
Pulse length = 9.500 usec
Relaxation delay = 2.000 sec
NA = 1419
F1 = 75.467751 MHz
F2 = 1.000000 MHz

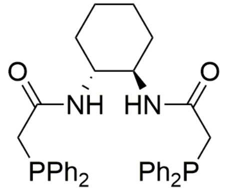

(*R*,*R*)-**DACH-ZYC-Phos-C1**

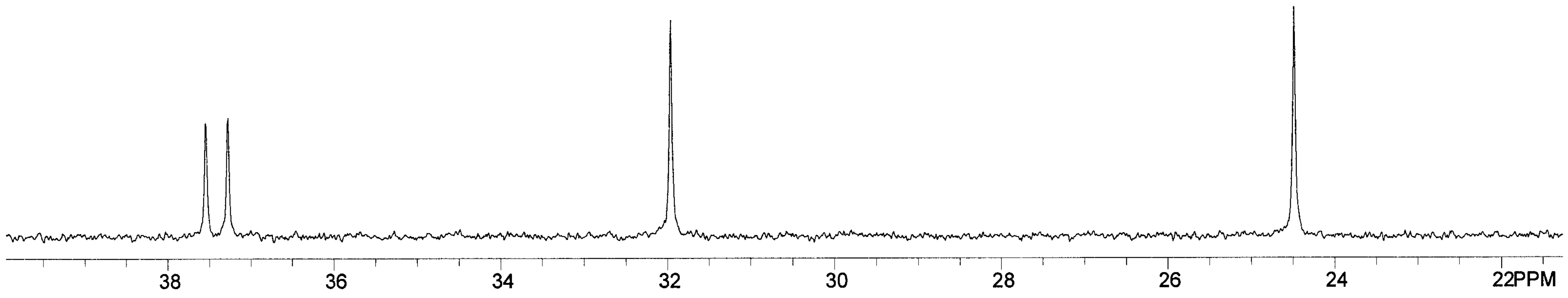

^{13}C NMR

2019-04-16 18:46:27.593
zyc-4-7
SOLVENT: CDCl3
Experiment = zgpg30
Pulse length = 9.500 usec
Relaxation delay = 2.000 sec
NA = 1419
F1 = 75.467751 MHz
F2 = 1.000000 MHz

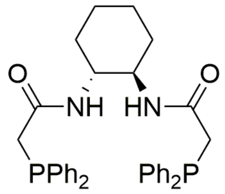

(*R*,*R*)-**DACH-ZYC-Phos-C1**

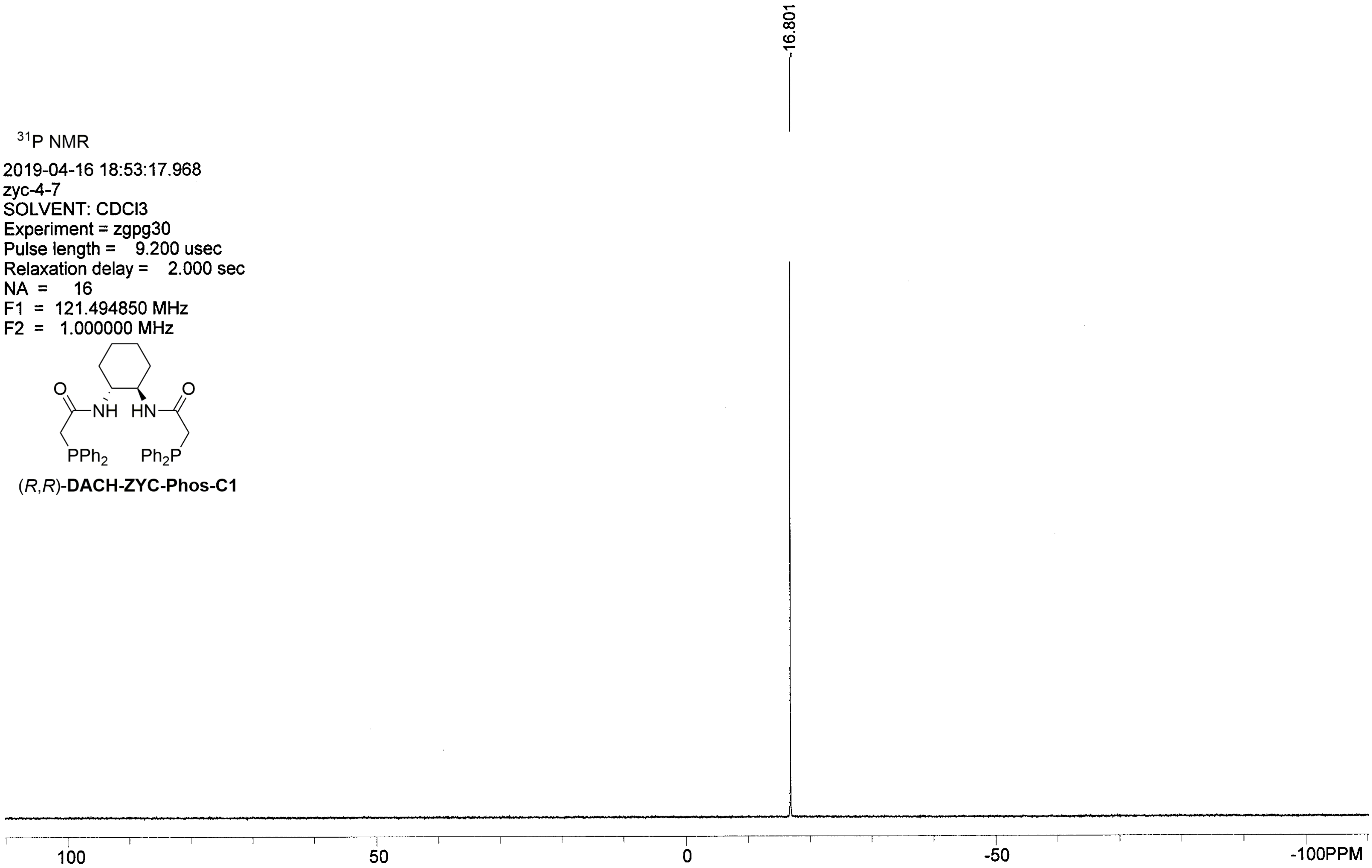

(*R*,*R*)-**DACH-ZYC-Phos-C1**

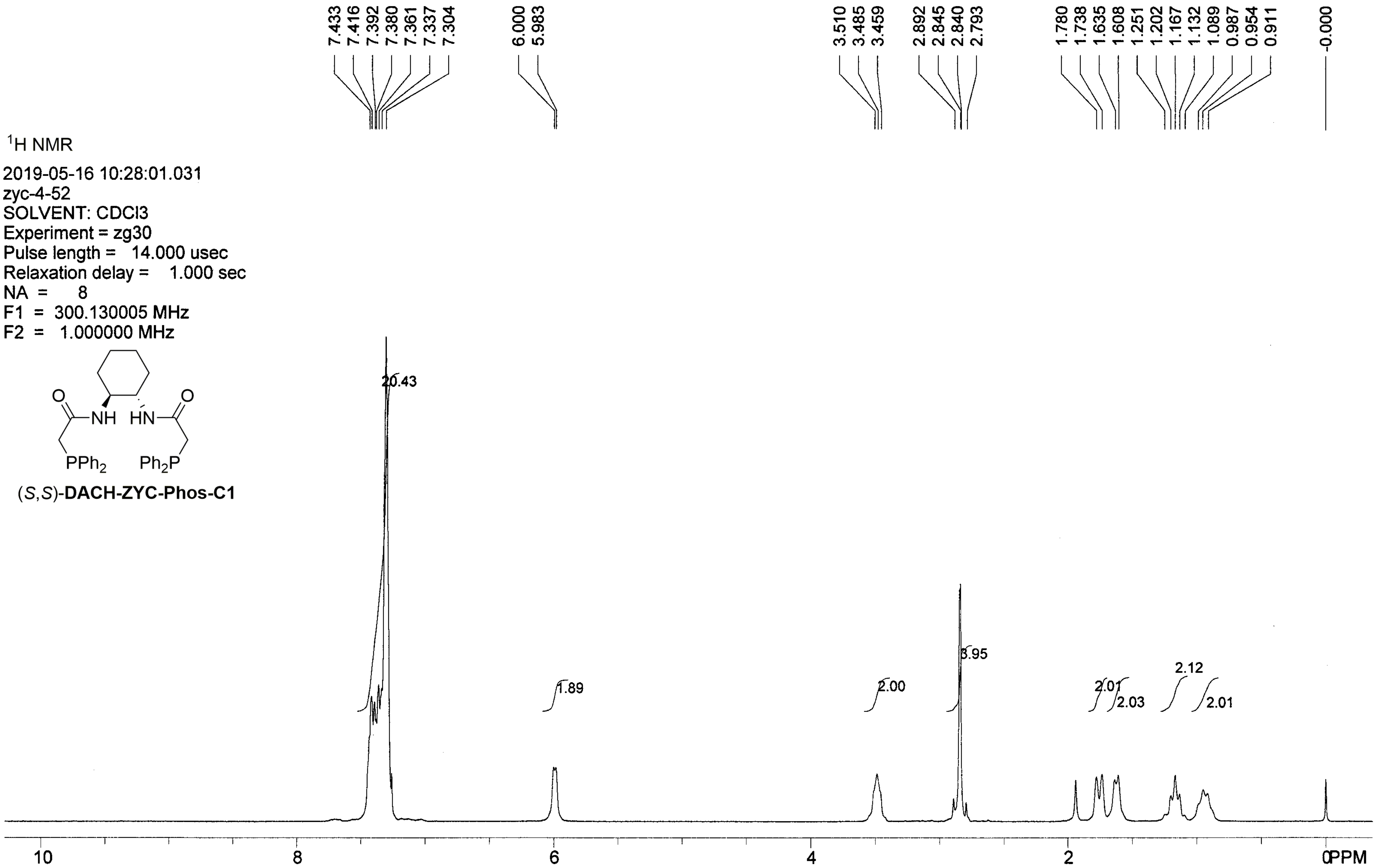
7.433
7.416
7.392
7.380
7.361
7.337
7.304
6.000
5.983
3.510
3.485
3.459
2.892
2.845
2.840
2.793
1.780
1.738
1.635
1.608
1.251
1.202
1.167
1.132
1.089
0.987
0.954
0.911
-0.000
1H NMR
2019-05-16 10:28:01.031
zyc-4-52
SOLVENT: CDCl3
Experiment = zg30
Pulse length = 14.000 usec
Relaxation delay = 1.000 sec
NA = 8
F1 = 300.130005 MHz
F2 = 1.000000 MHz
O
NH
HN
O
PPh2
Ph2P
(S,S)-DACH-ZYC-Phos-C1
20.43
1.89
2.00
3.95
2.01
2.03
2.12
2.01
10
8
6
4
2
0PPM

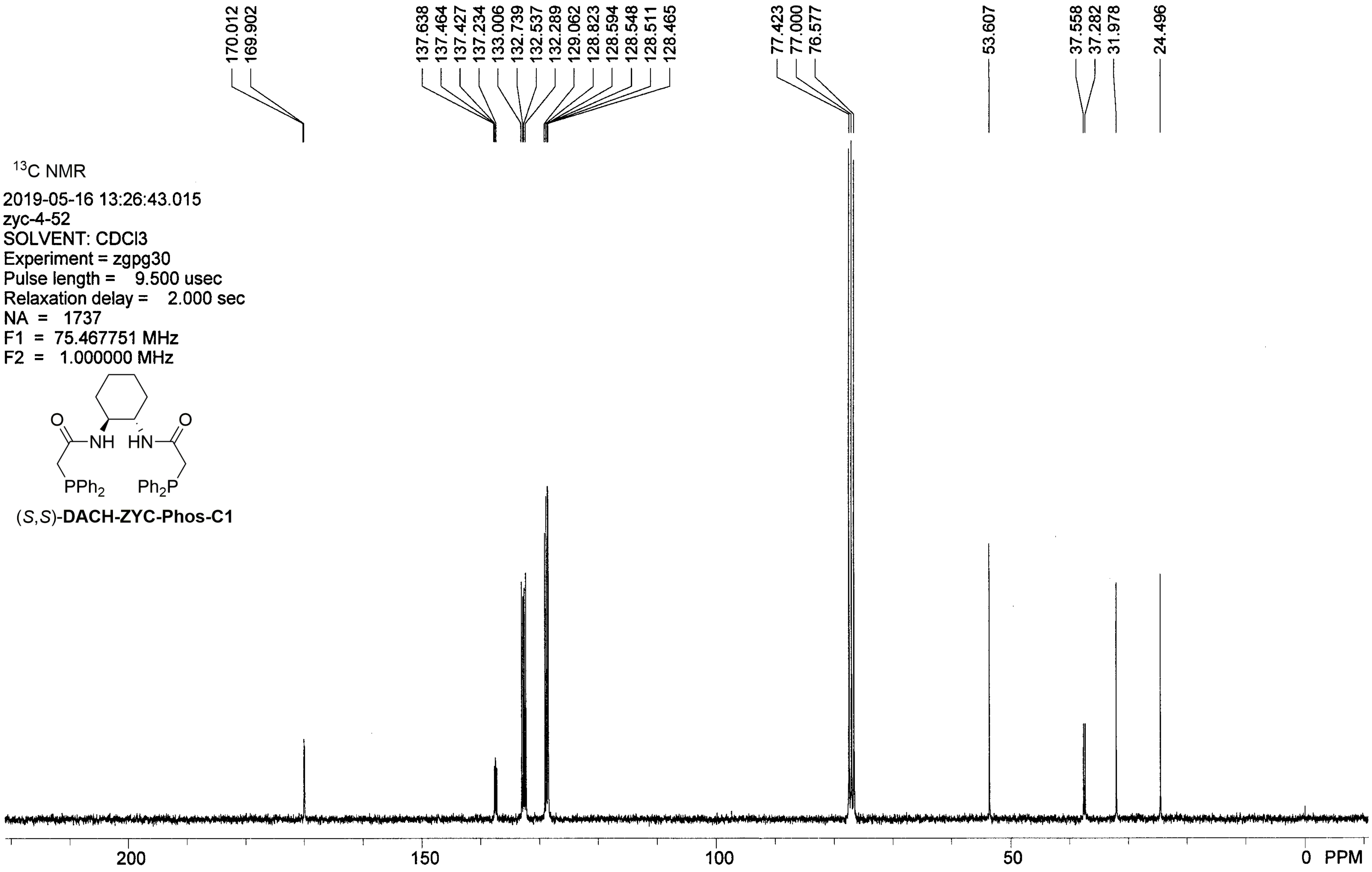

(*S*,*S*)-**DACH-ZYC-Phos-C1**

^{13}C NMR

2019-05-16 13:26:43.015
zyc-4-52
SOLVENT: CDCl3
Experiment = zgpg30
Pulse length = 9.500 usec
Relaxation delay = 2.000 sec
NA = 1737
F1 = 75.467751 MHz
F2 = 1.000000 MHz

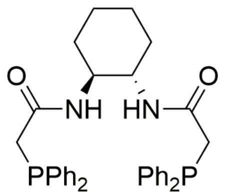

(*S*,*S*)-**DACH-ZYC-Phos-C1**

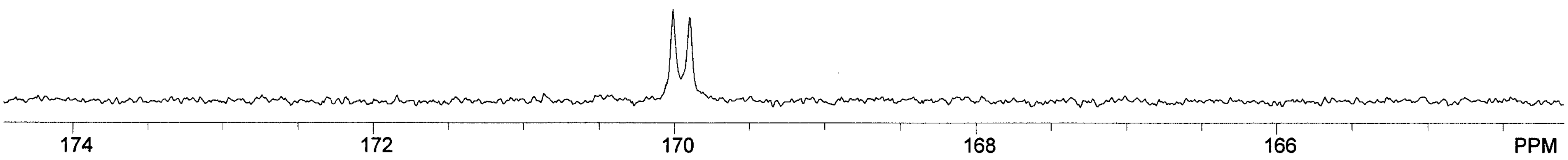

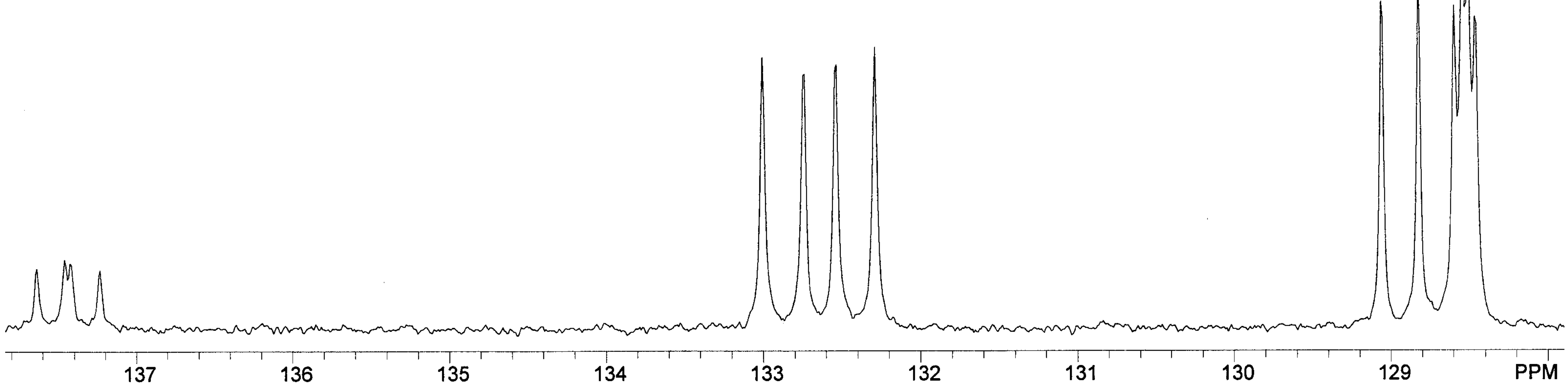

^{13}C NMR
2019-05-16 13:26:43.015
zyc-4-52
SOLVENT: CDCl3
Experiment = zgpg30
Pulse length = 9.500 usec
Relaxation delay = 2.000 sec
NA = 1737
F1 = 75.467751 MHz
F2 = 1.000000 MHz

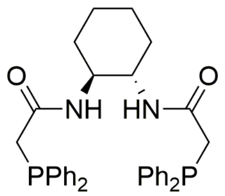

(*S*,*S*)-**DACH-ZYC-Phos-C1**

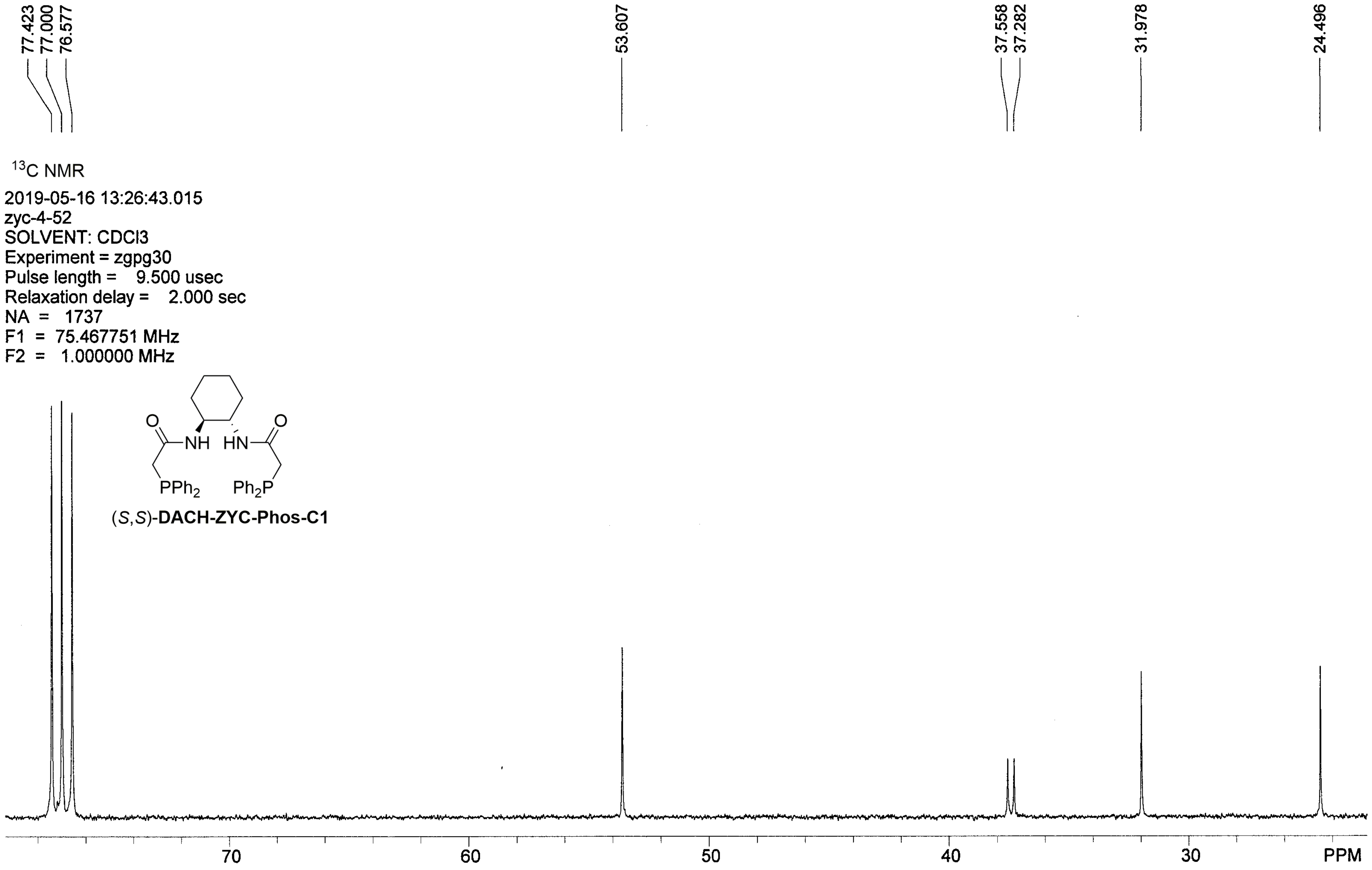
77.423
77.000
76.577
53.607
37.558
37.282
31.978
24.496
^{13}C NMR
2019-05-16 13:26:43.015
zyc-4-52
SOLVENT: CDCl3
Experiment = zgpg30
Pulse length = 9.500 usec
Relaxation delay = 2.000 sec
NA = 1737
F1 = 75.467751 MHz
F2 = 1.000000 MHz
NH
HN
O
O
PPh2
Ph2P
(S,S)-DACH-ZYC-Phos-C1
70
60
50
40
30
PPM

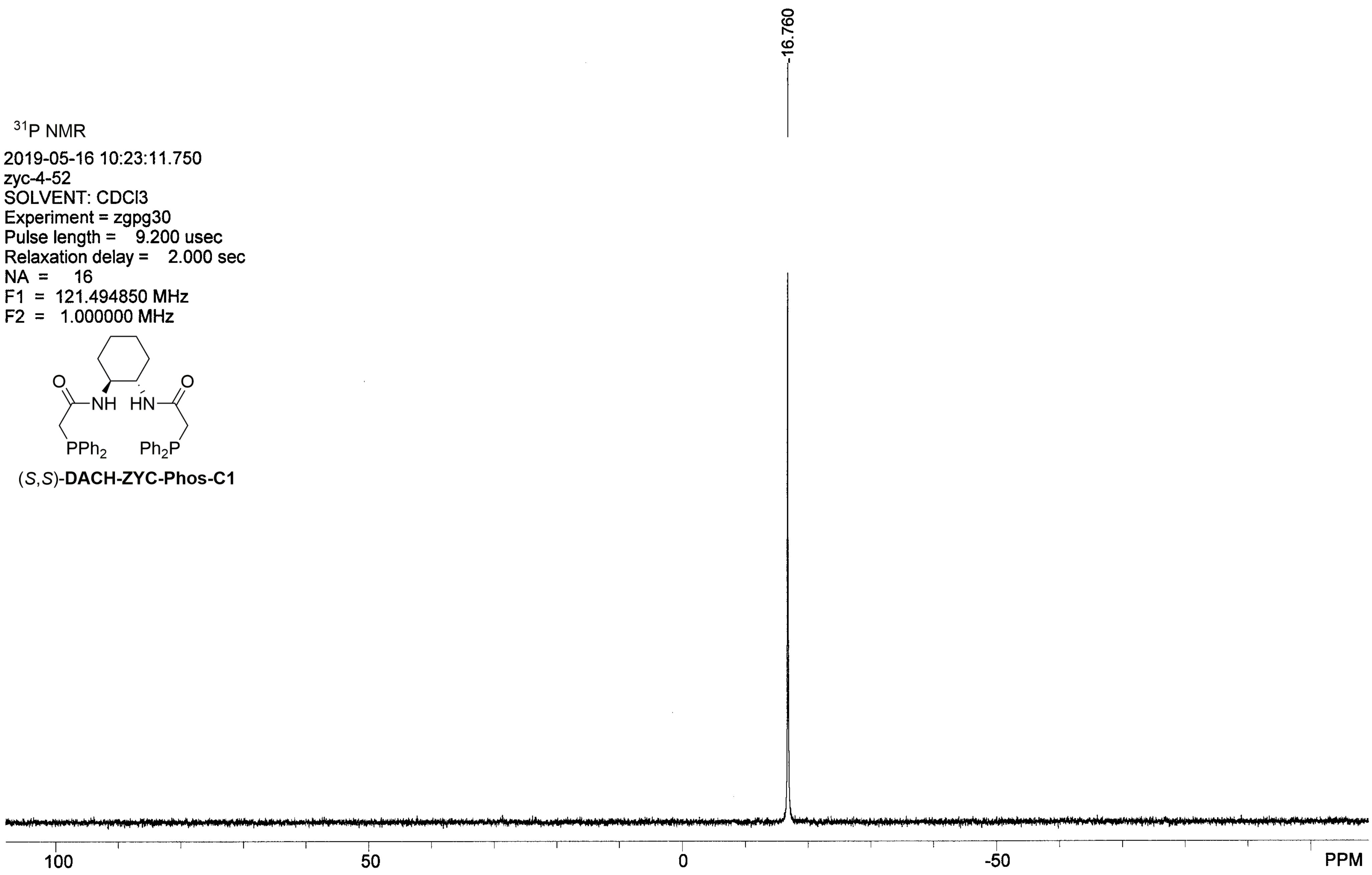

(*S*,*S*)-**DACH-ZYC-Phos-C1**

^1H NMR

2019-03-13 16:24:12.000
zyc-3-144
NA = 8
Solvent = CDCl3
PTS1d = 32768
F1 = 300.130005 MHz
F2 = 1.000000 MHz

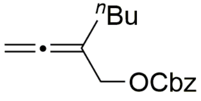

1a

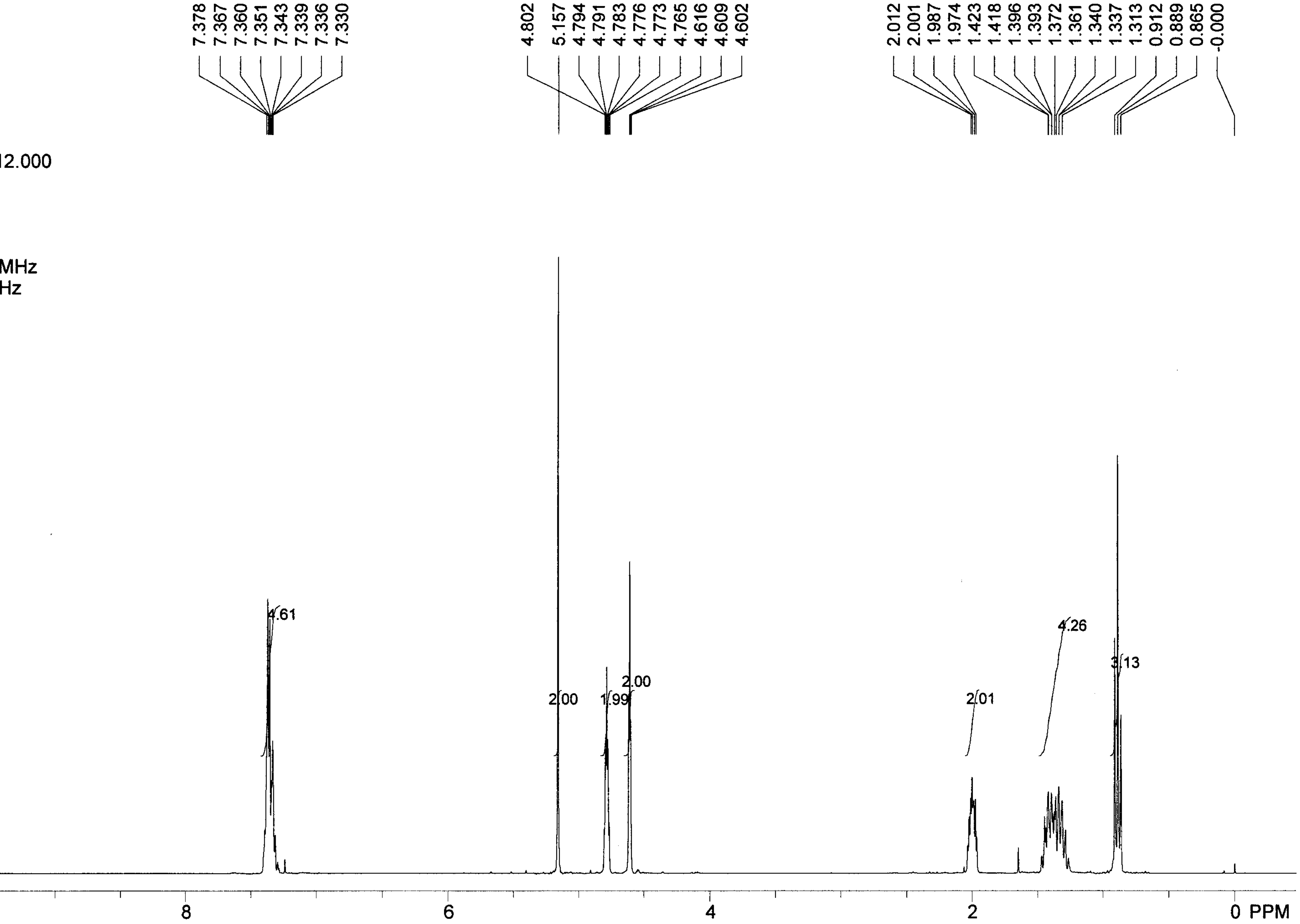

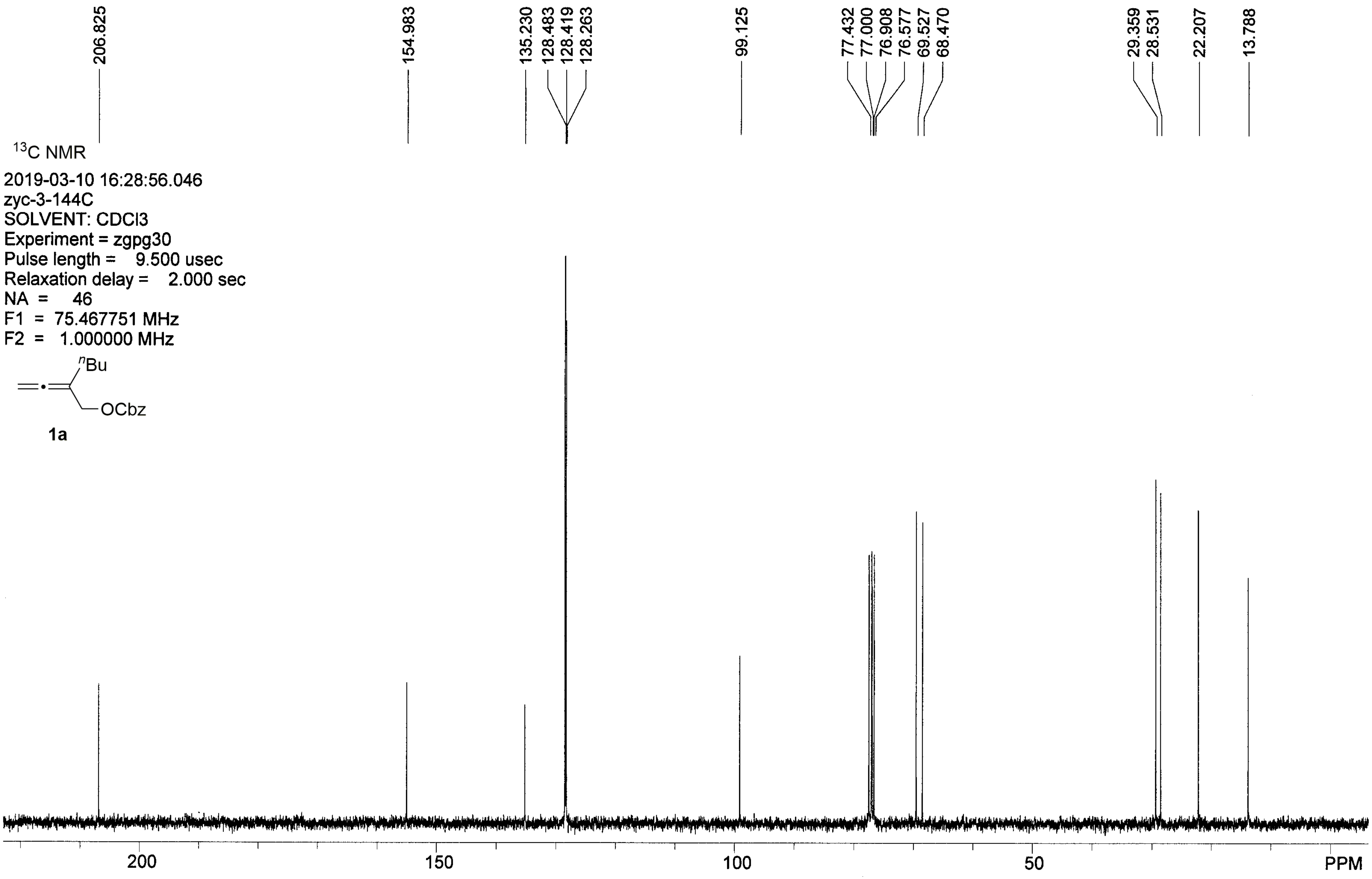
206.825
154.983
135.230
128.483
128.419
128.263
99.125
77.432
77.000
76.908
76.577
69.527
68.470
29.359
28.531
22.207
13.788
^{13}C NMR
2019-03-10 16:28:56.046
zyc-3-144C
SOLVENT: CDCl3
Experiment = zgpg30
Pulse length = 9.500 usec
Relaxation delay = 2.000 sec
NA = 46
F1 = 75.467751 MHz
F2 = 1.000000 MHz
^{n}Bu
OCbz
1a
200
150
100
50
PPM

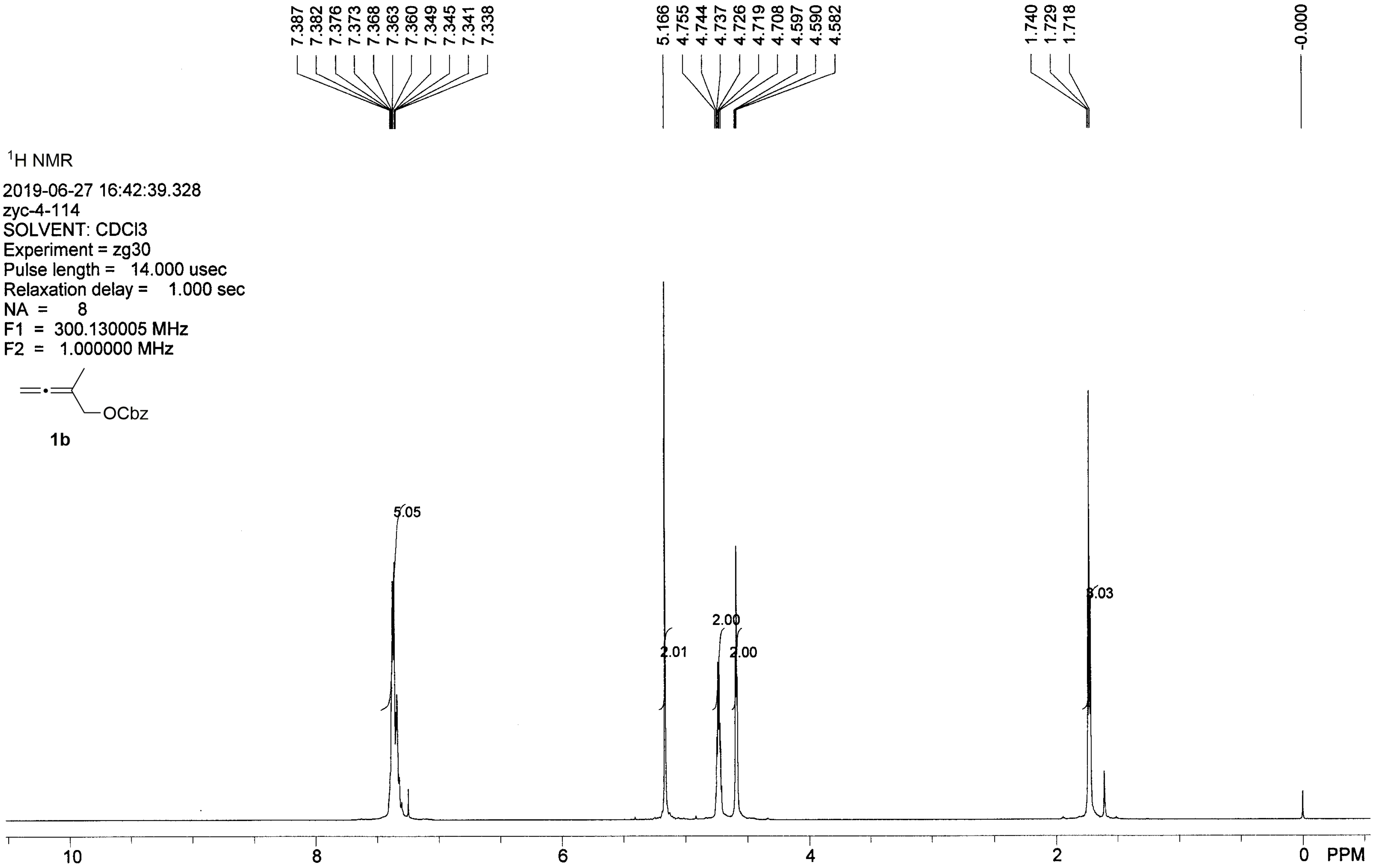
7.387
7.382
7.376
7.373
7.368
7.363
7.360
7.349
7.345
7.341
7.338
5.166
4.755
4.744
4.737
4.726
4.719
4.708
4.597
4.590
4.582
1.740
1.729
1.718
-0.000
^{1}H NMR
2019-06-27 16:42:39.328
zyc-4-114
SOLVENT: CDCl3
Experiment = zg30
Pulse length = 14.000 usec
Relaxation delay = 1.000 sec
NA = 8
F1 = 300.130005 MHz
F2 = 1.000000 MHz
OCbz
1b
5.05
2.01
2.00
2.00
3.03
10
8
6
4
2
0
PPM

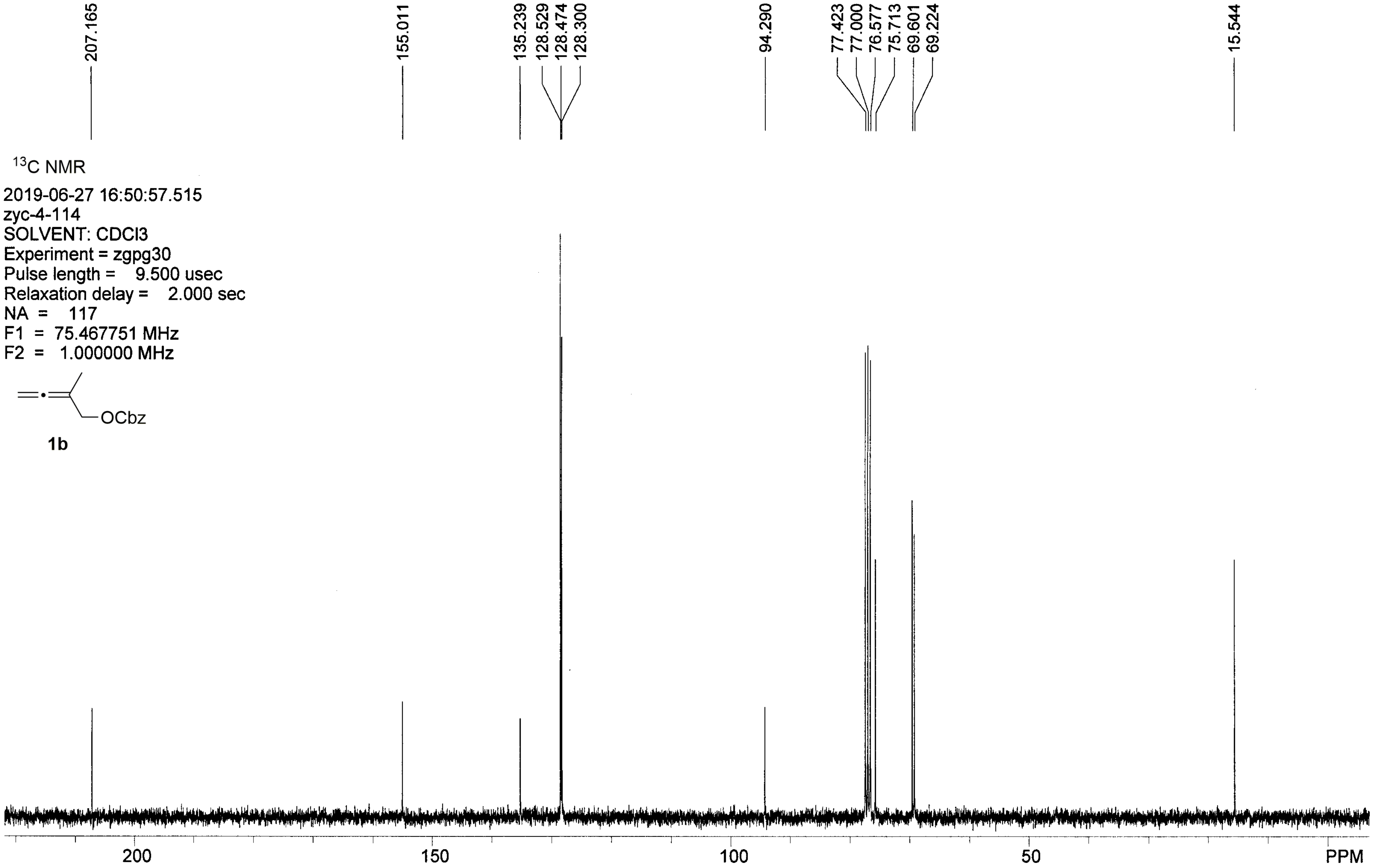

^{13}C NMR

2019-06-27 16:50:57.515
zyc-4-114
SOLVENT: CDCl3
Experiment = zgpg30
Pulse length = 9.500 usec
Relaxation delay = 2.000 sec
NA = 117
F1 = 75.467751 MHz
F2 = 1.000000 MHz

1b

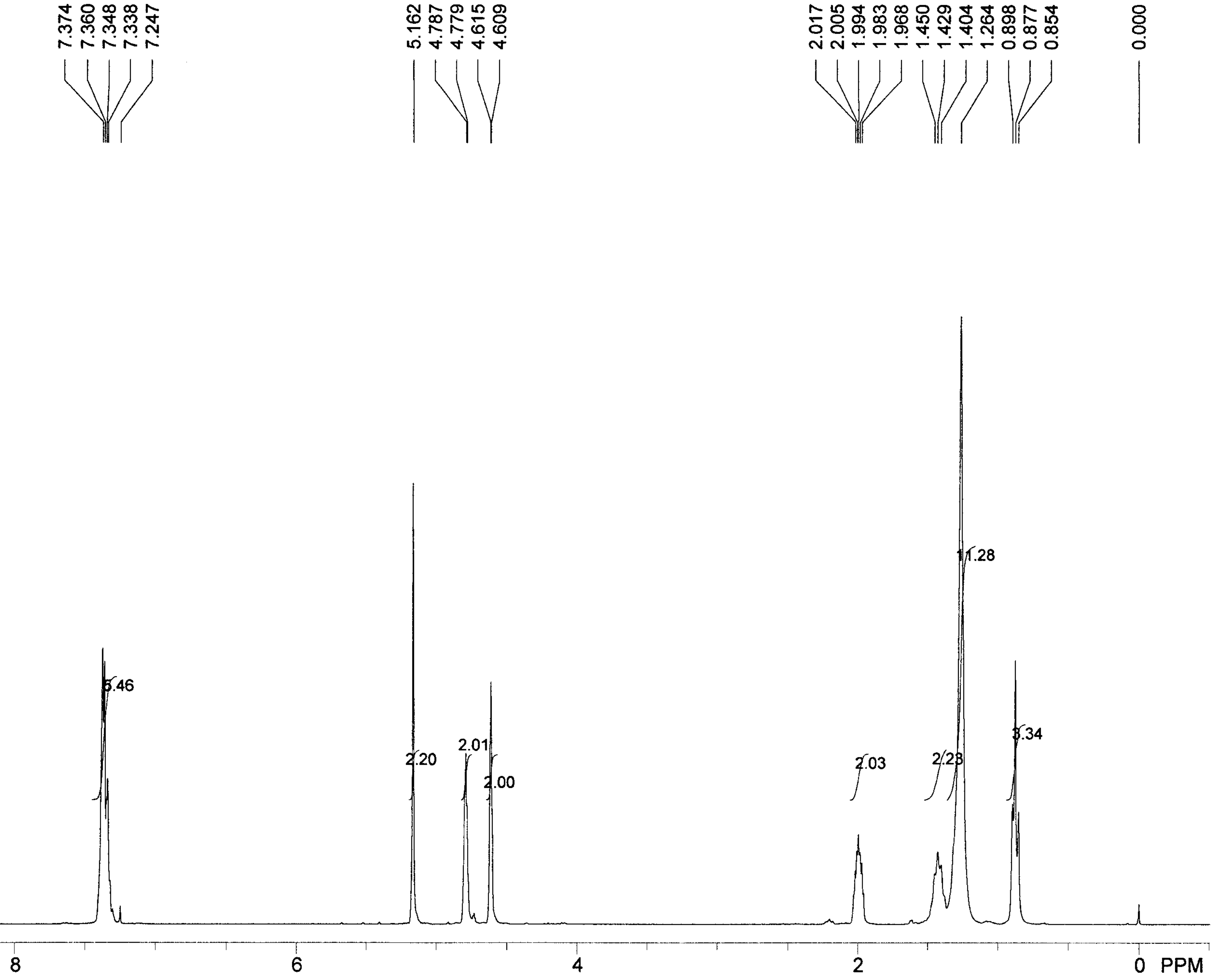

^{1}H NMR

2019-05-06 21:30:18.625
zyc-4-37
SOLVENT: CDCl3
Experiment = zg30
Pulse length = 14.000 usec
Relaxation delay = 1.000 sec
NA = 8
F1 = 300.130005 MHz
F2 = 1.000000 MHz

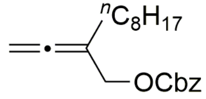

1c

2019-05-08 20:24:39.921
zyc-4-37purity
SOLVENT: CDCl3
Experiment = zg30
Pulse length = 14.000 usec
Relaxation delay = 1.000 sec
NA = 8
F1 = 300.130005 MHz
F2 = 1.000000 MHz

43.5mg sample was add 10 uL [illegible]

purity $= \left(\frac{124.03}{2\times 100} \times \frac{10}{46} \times 316.44 / 43.5 \right) \times 100\%$

$= 98\%$

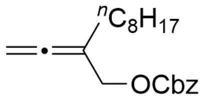

1c

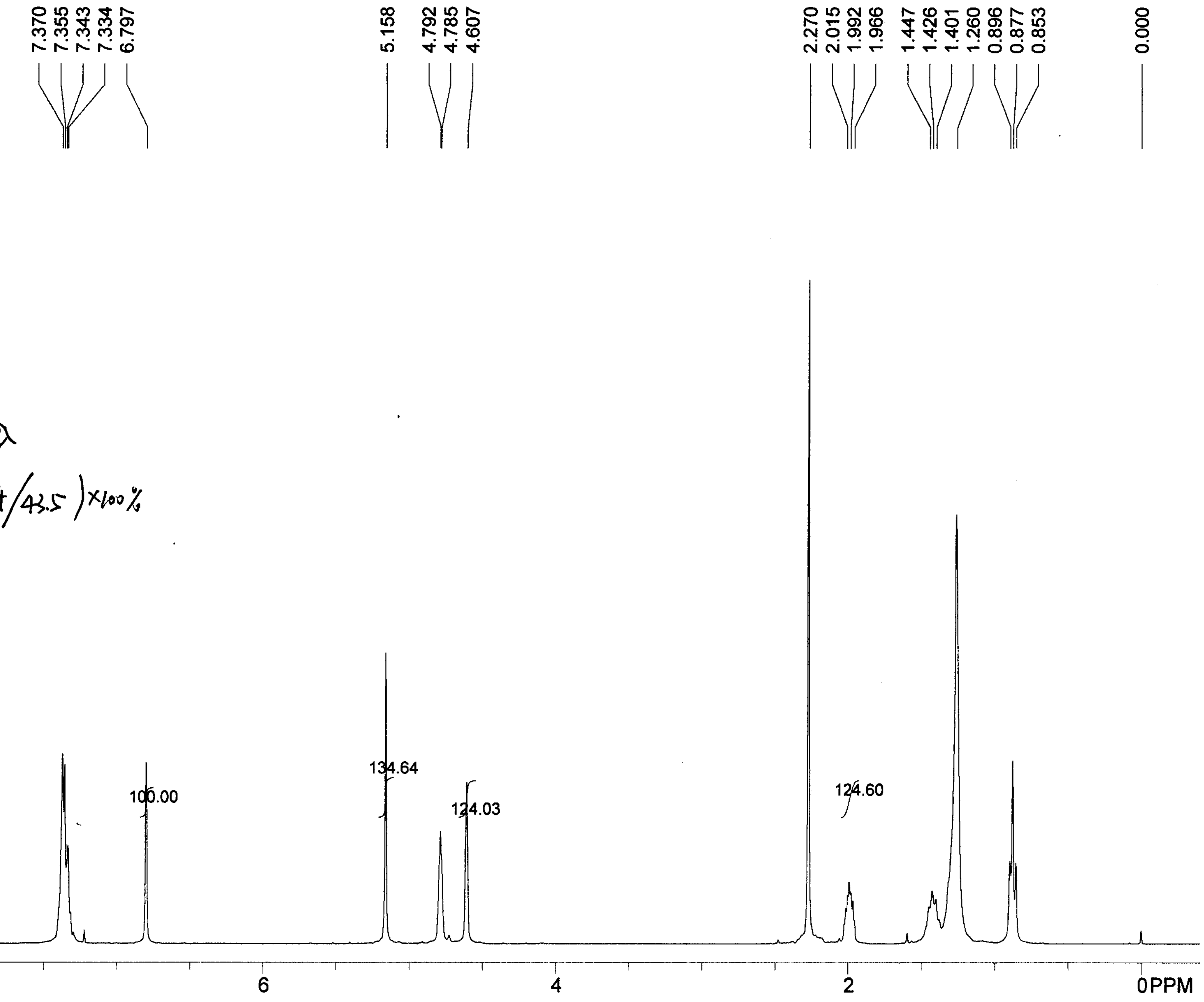

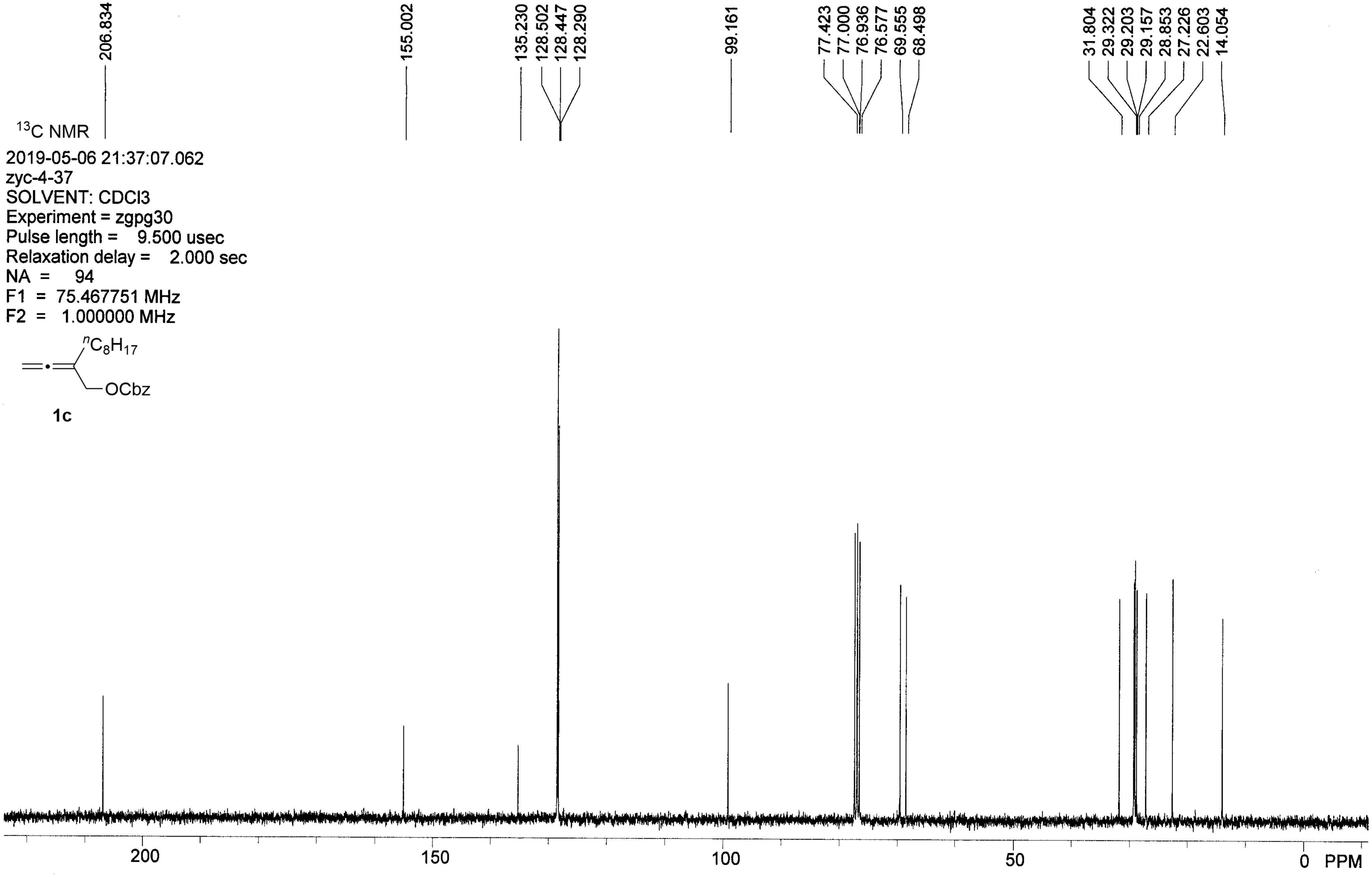

^{13}C NMR
2019-05-06 21:37:07.062
zyc-4-37
SOLVENT: CDCl3
Experiment = zgpg30
Pulse length = 9.500 usec
Relaxation delay = 2.000 sec
NA = 94
F1 = 75.467751 MHz
F2 = 1.000000 MHz
$^{n}C_8H_{17}$
OCbz
1c
206.834
155.002
135.230
128.502
128.447
128.290
99.161
77.423
77.000
76.936
76.577
69.555
68.498
31.804
29.322
29.203
29.157
28.853
27.226
22.603
14.054
200
150
100
50
0 PPM

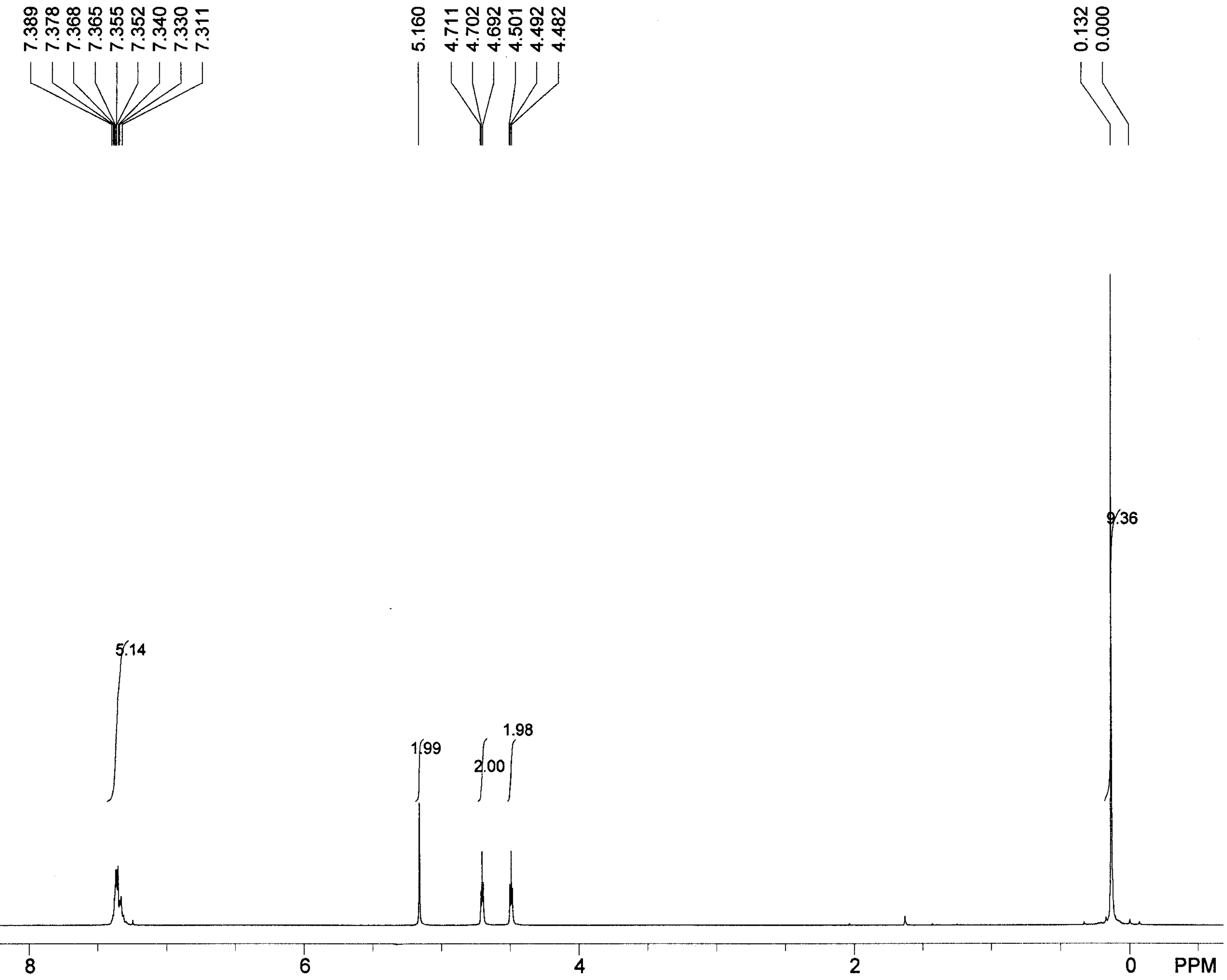

^1H NMR

2019-07-05 14:32:48.953
zyc-4-123
SOLVENT: CDCl3
Experiment = zg30
Pulse length = 14.000 usec
Relaxation delay = 1.000 sec
NA = 8
F1 = 300.130005 MHz
F2 = 1.000000 MHz

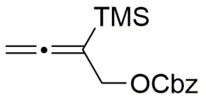

1d

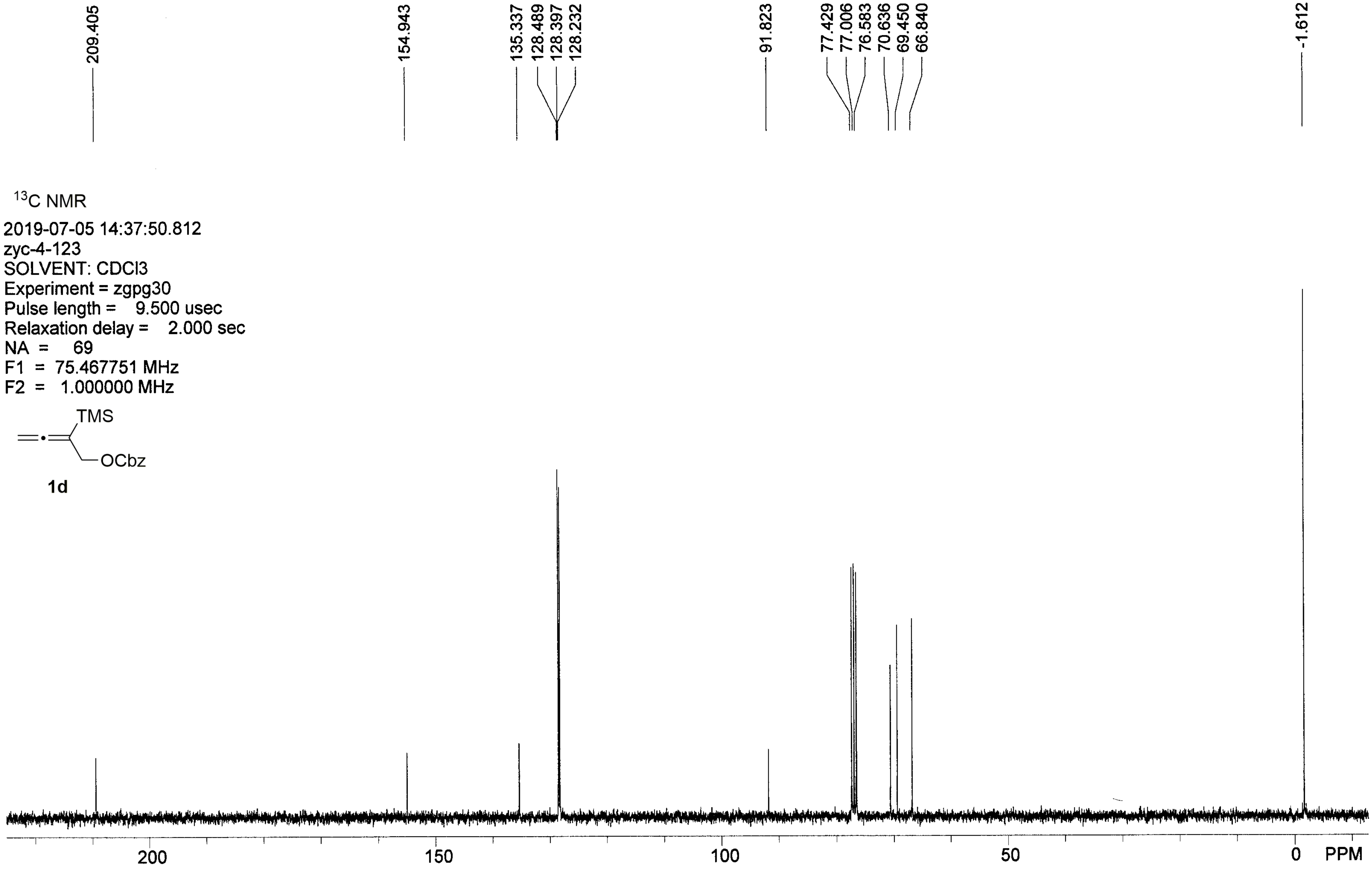
209.405
154.943
135.337
128.489
128.397
128.232
91.823
77.429
77.006
76.583
70.636
69.450
66.840
-1.612
^{13}C NMR
2019-07-05 14:37:50.812
zyc-4-123
SOLVENT: CDCl3
Experiment = zgpg30
Pulse length = 9.500 usec
Relaxation delay = 2.000 sec
NA = 69
F1 = 75.467751 MHz
F2 = 1.000000 MHz
TMS
OCbz
1d
200
150
100
50
0
PPM

^{1}H NMR

2019-07-19 20:59:47.421
zyc-4-143
SOLVENT: CDCl3
Experiment = zg30
Pulse length = 14.000 usec
Relaxation delay = 1.000 sec
NA = 8
F1 = 300.130005 MHz
F2 = 1.000000 MHz

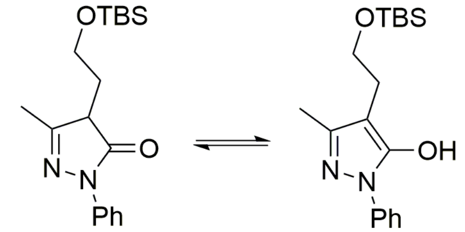

2p

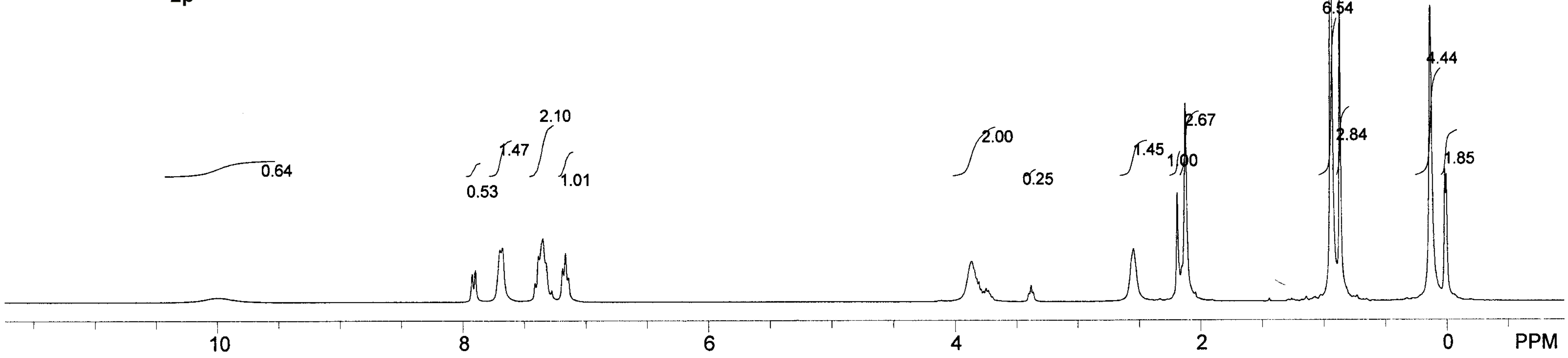

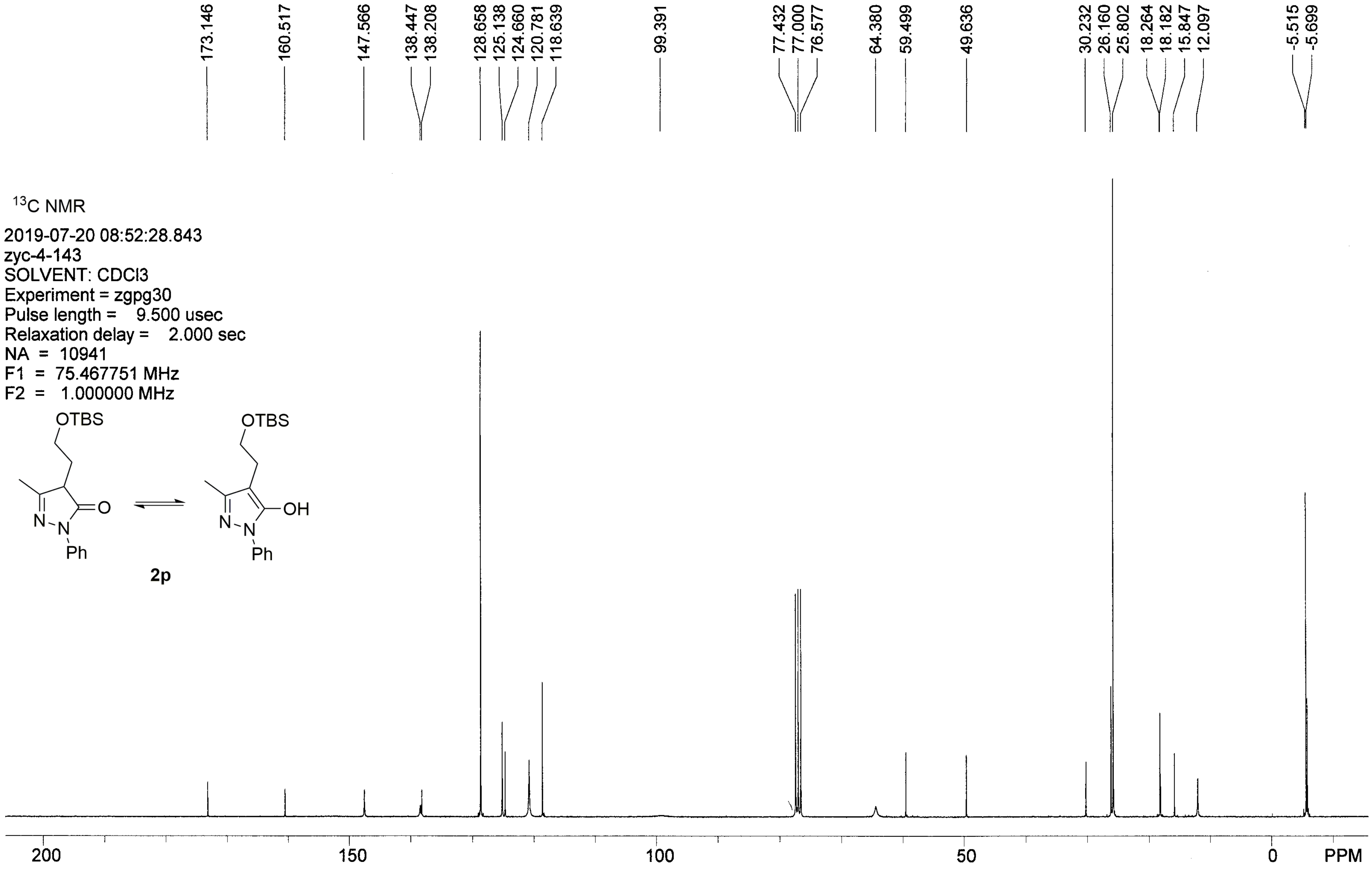

173.146
160.517
147.566
138.447
138.208
128.658
125.138
124.660
120.781
118.639
99.391
77.432
77.000
76.577
64.380
59.499
49.636
30.232
26.160
25.802
18.264
18.182
15.847
12.097
-5.515
-5.699
13C NMR
2019-07-20 08:52:28.843
zyc-4-143
SOLVENT: CDCl3
Experiment = zgpg30
Pulse length = 9.500 usec
Relaxation delay = 2.000 sec
NA = 10941
F1 = 75.467751 MHz
F2 = 1.000000 MHz
OTBS
N
N
Ph
O
OH
2p
200
150
100
50
0
PPM

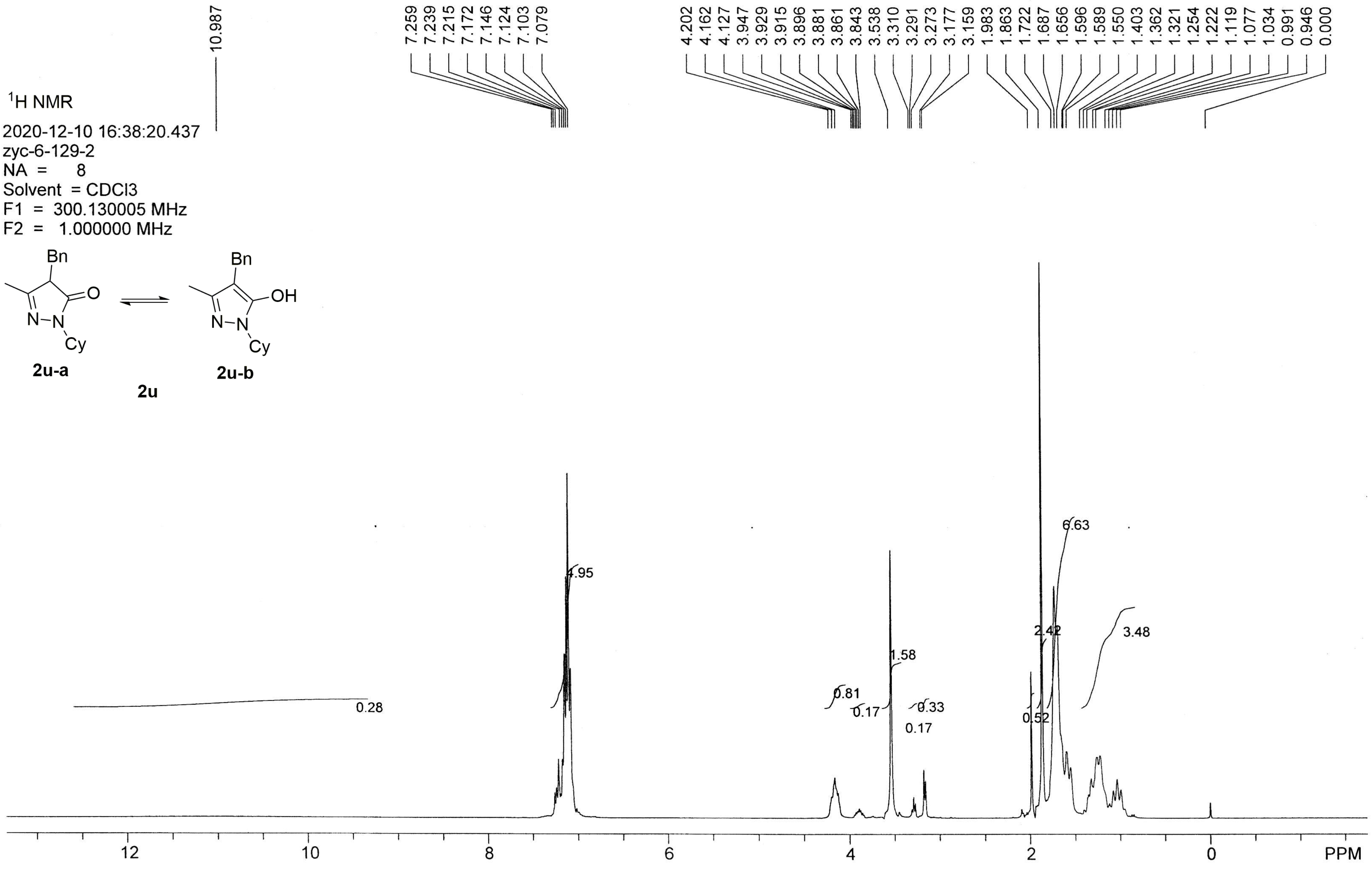

^{1}H NMR
2020-12-10 16:38:20.437
zyc-6-129-2
NA = 8
Solvent = CDCl3
F1 = 300.130005 MHz
F2 = 1.000000 MHz
Bn
O
N–N
Cy
2u-a
Bn
OH
N–N
Cy
2u-b
2u
10.987
7.259
7.239
7.215
7.172
7.146
7.124
7.103
7.079
4.202
4.162
4.127
3.947
3.929
3.915
3.896
3.881
3.861
3.843
3.538
3.310
3.291
3.273
3.177
3.159
1.983
1.863
1.722
1.687
1.656
1.596
1.589
1.550
1.403
1.362
1.321
1.254
1.222
1.119
1.077
1.034
0.991
0.946
0.000
0.28
4.95
0.81
0.17
1.58
0.33
0.17
0.52
2.42
6.63
3.48
12
10
8
6
4
2
0
PPM

^{13}C NMR

2020-12-10 18:47:30.281
zyc-6-129-2
NA = 2165
Solvent = CDCl3
F1 = 75.467751 MHz
F2 = 1.000000 MHz

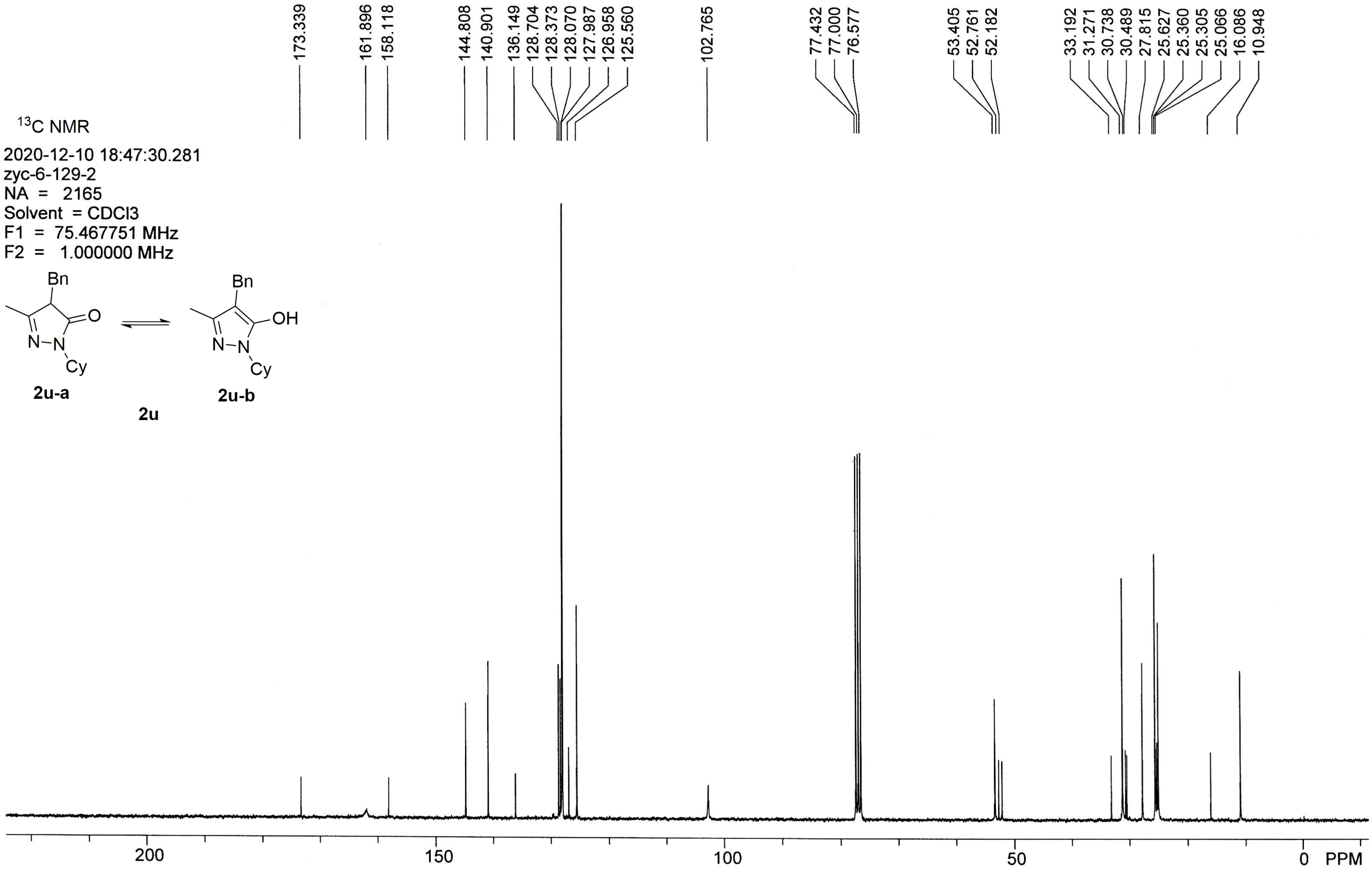

2u-a **2u** **2u-b**

^1H NMR

2020-12-11 14:23:28.359
zyc-6-131
NA = 8
Solvent = CDCl3
F1 = 300.130005 MHz
F2 = 1.000000 MHz

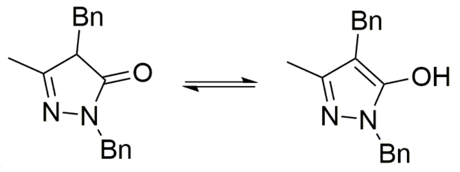

2v-a **2v** **2v-b**

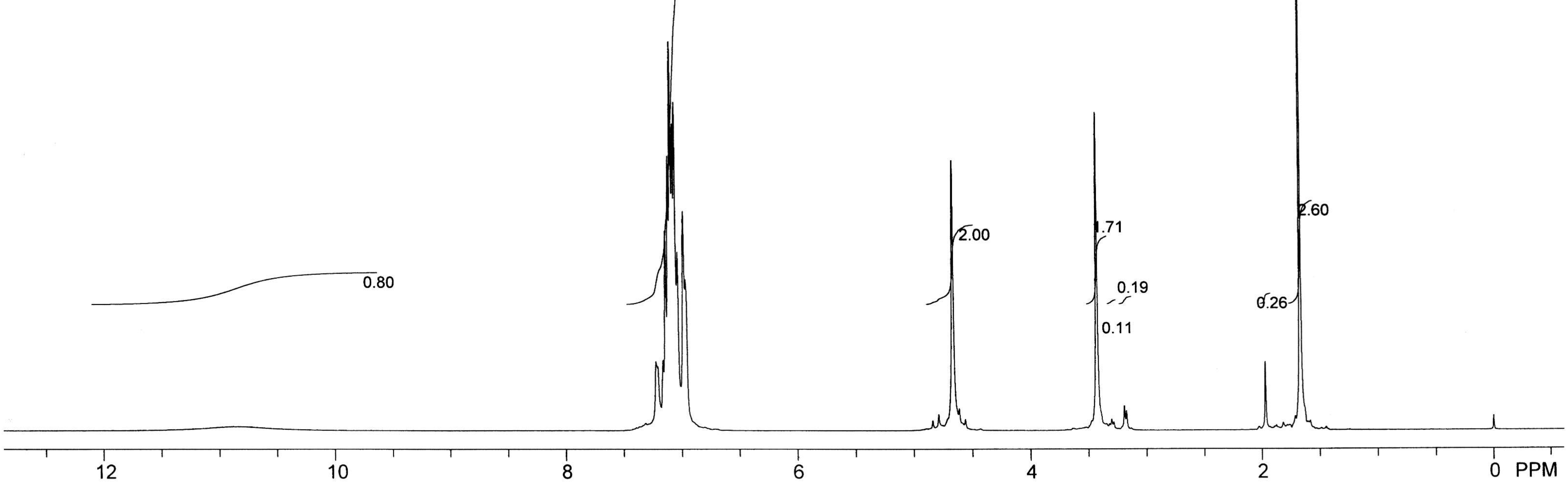

^{13}C NMR

2020-12-11 17:37:23.703
zyc-6-131
NA = 500
Solvent = CDCl3
F1 = 75.467751 MHz
F2 = 1.000000 MHz

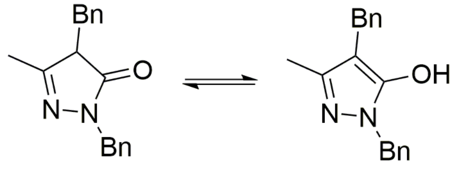

2v-a **2v-b**

2v

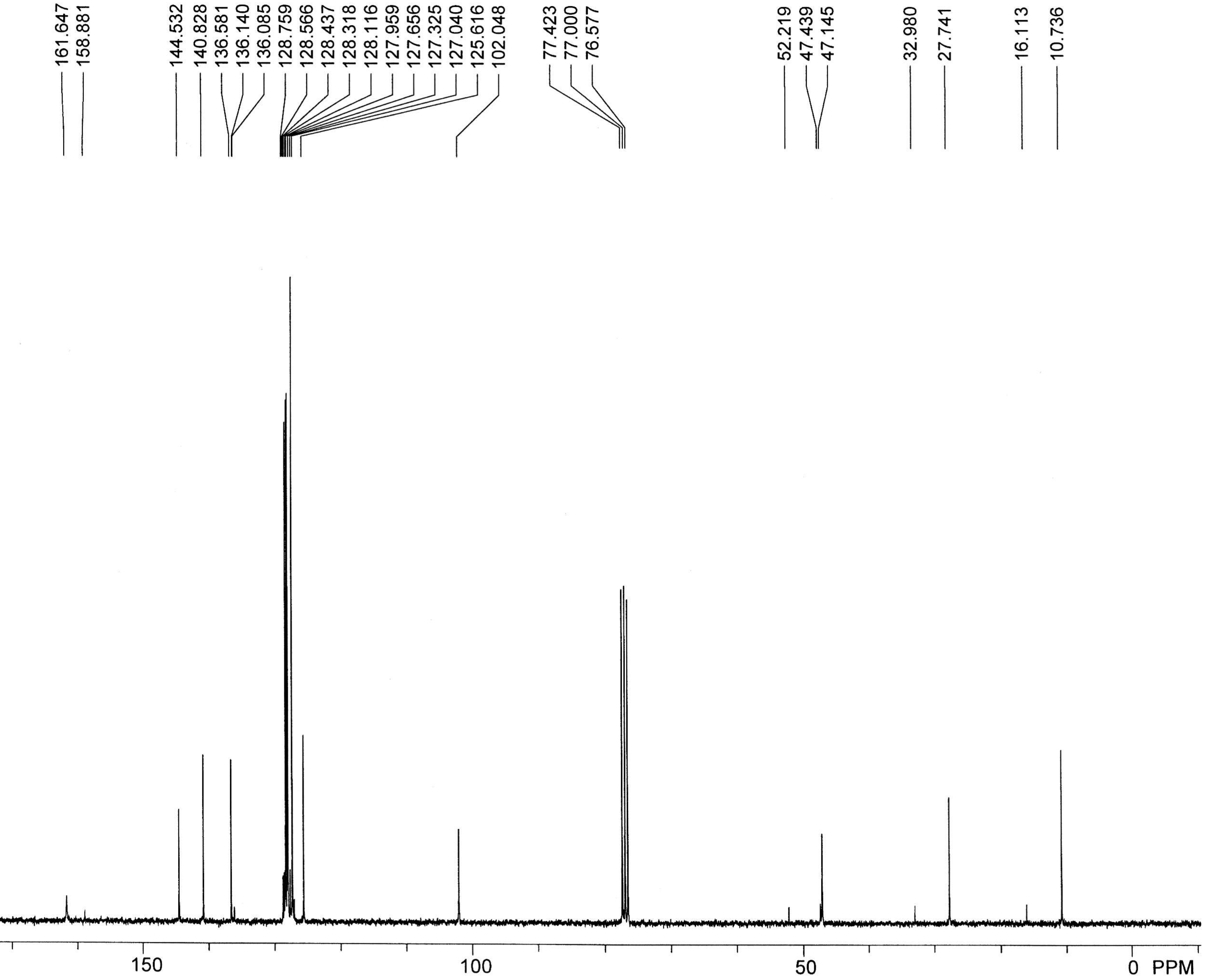

^{1}H NMR

2019-05-06 16:26:10.343
zyc-4-35-1
SOLVENT: CDCl3
Experiment = zg30
Pulse length = 14.000 usec
Relaxation delay = 1.000 sec
NA = 8
F1 = 300.130005 MHz
F2 = 1.000000 MHz

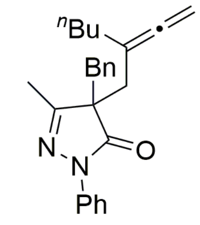

3aa

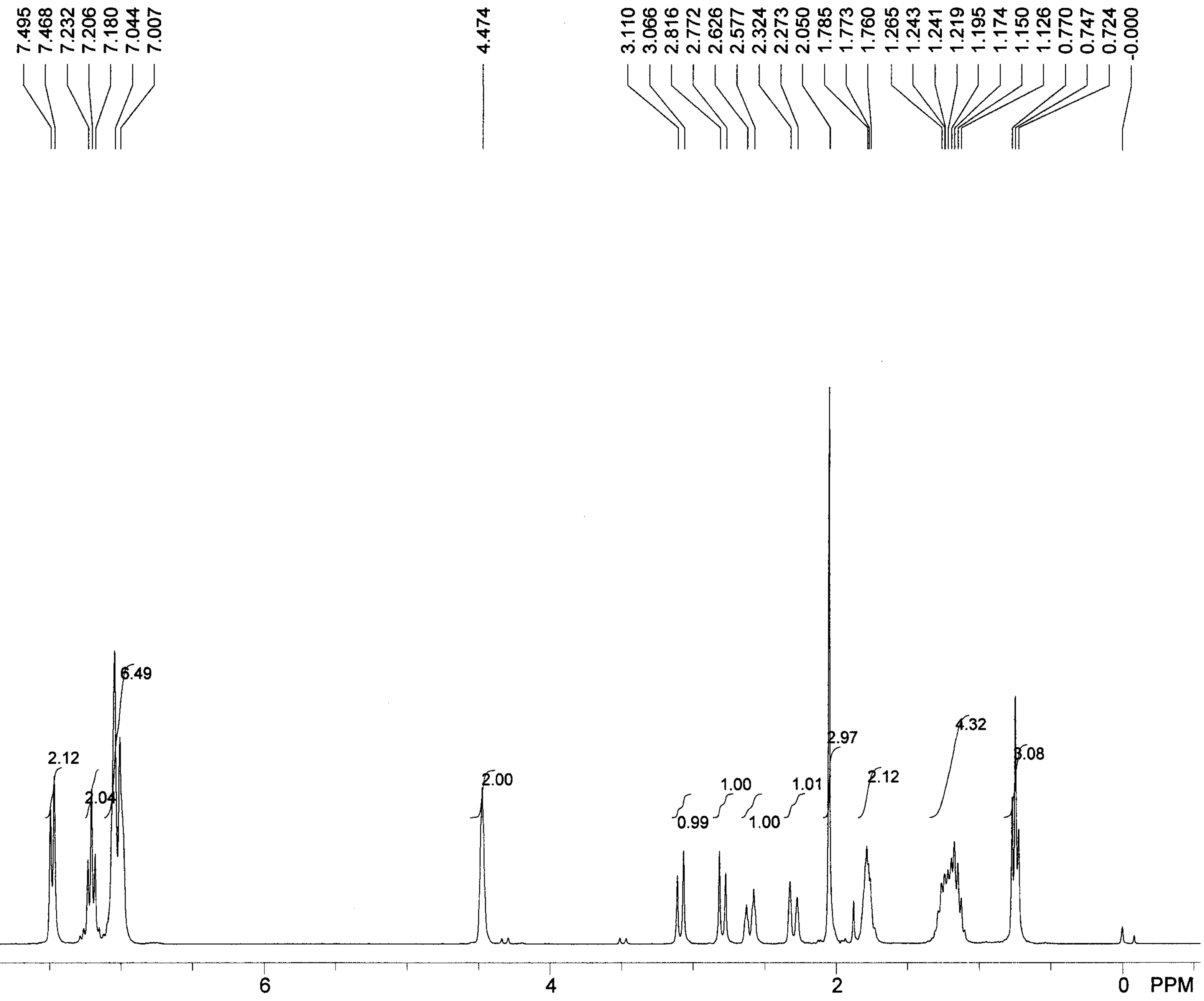

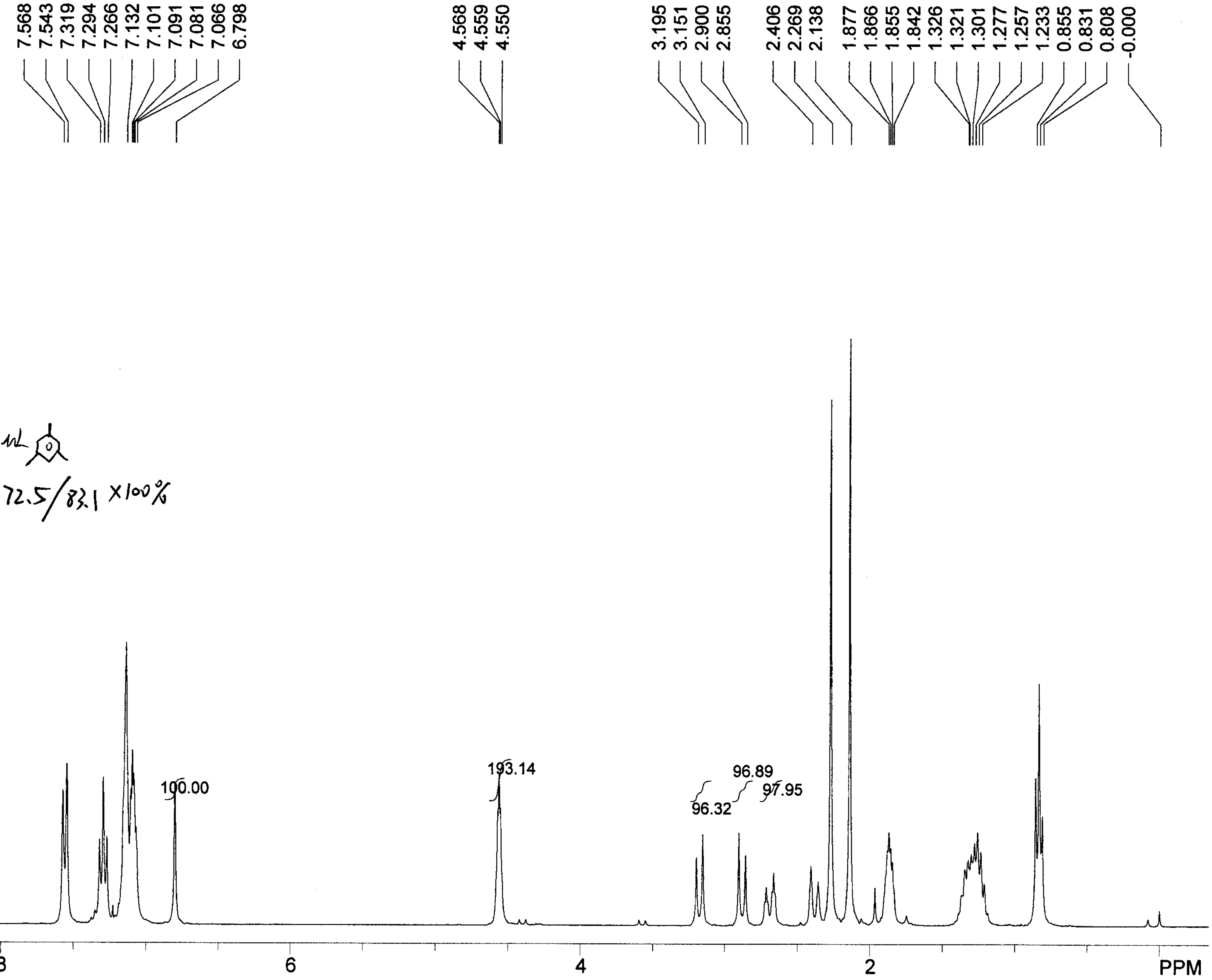

2019-05-07 16:58:54.953
zyc-4-35-1purity
SOLVENT: CDCl3
Experiment = zg30
Pulse length = 14.000 usec
Relaxation delay = 1.000 sec
NA = 8
F1 = 300.130005 MHz
F2 = 1.000000 MHz

83.1mg sample was added 10 uL

purity = $\frac{96.32}{100} \times \frac{10}{46} \times 372.5 / 83.1 \times 100\%$

= 94%

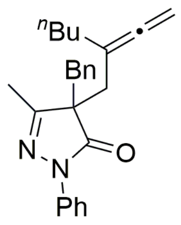

3aa

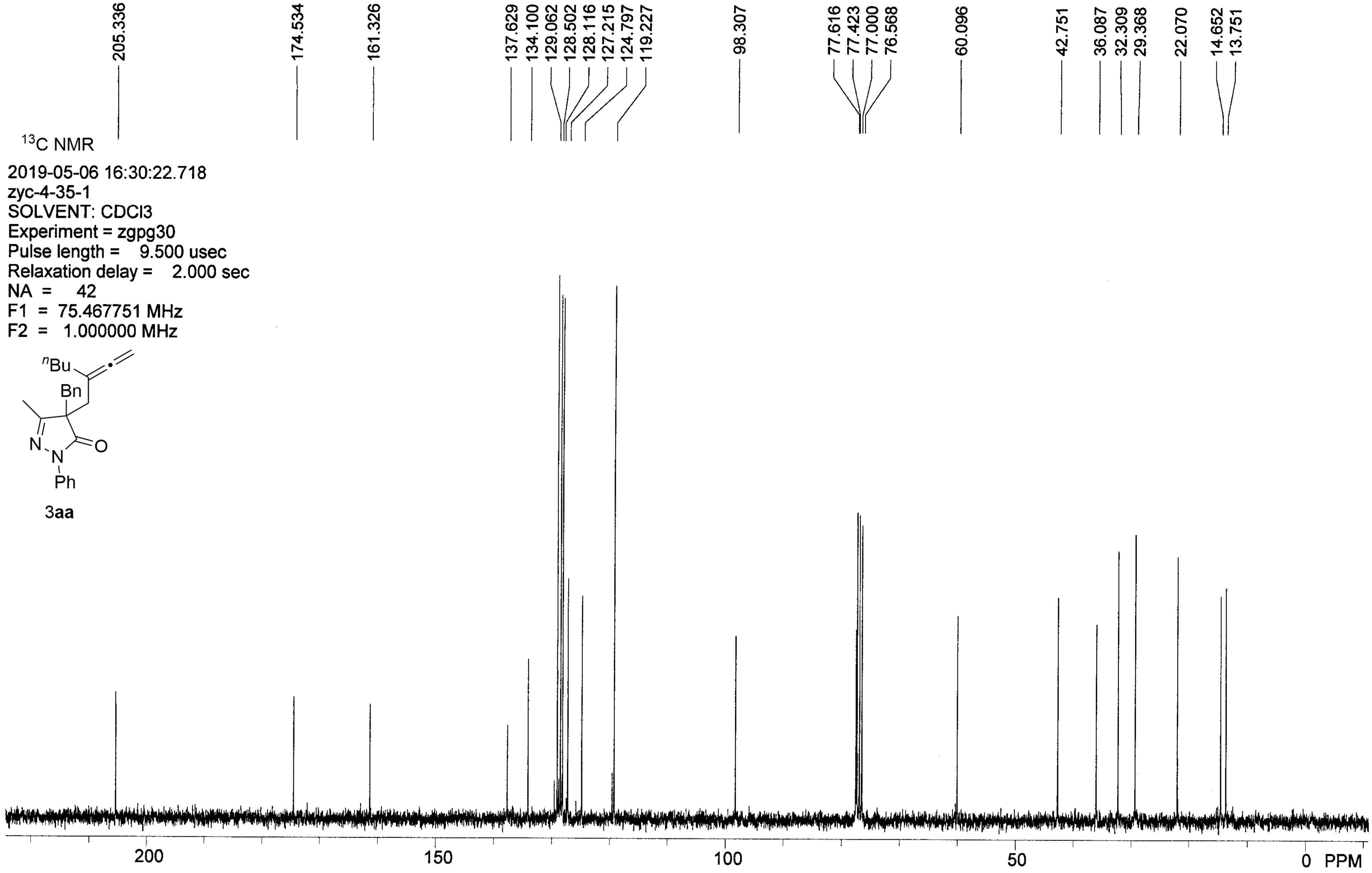
205.336
174.534
161.326
137.629
134.100
129.062
128.502
128.116
127.215
124.797
119.227
98.307
77.616
77.423
77.000
76.568
60.096
42.751
36.087
32.309
29.368
22.070
14.652
13.751
13C NMR
2019-05-06 16:30:22.718
zyc-4-35-1
SOLVENT: CDCl3
Experiment = zgpg30
Pulse length = 9.500 usec
Relaxation delay = 2.000 sec
NA = 42
F1 = 75.467751 MHz
F2 = 1.000000 MHz
nBu
Bn
N
N
O
Ph
3aa
200
150
100
50
0 PPM

^{1}H NMR

2019-05-09 14:45:42.796
zyc-4-35-2-2
SOLVENT: CDCl3
Experiment = zg30
Pulse length = 14.000 usec
Relaxation delay = 1.000 sec
NA = 8
F1 = 300.130005 MHz
F2 = 1.000000 MHz

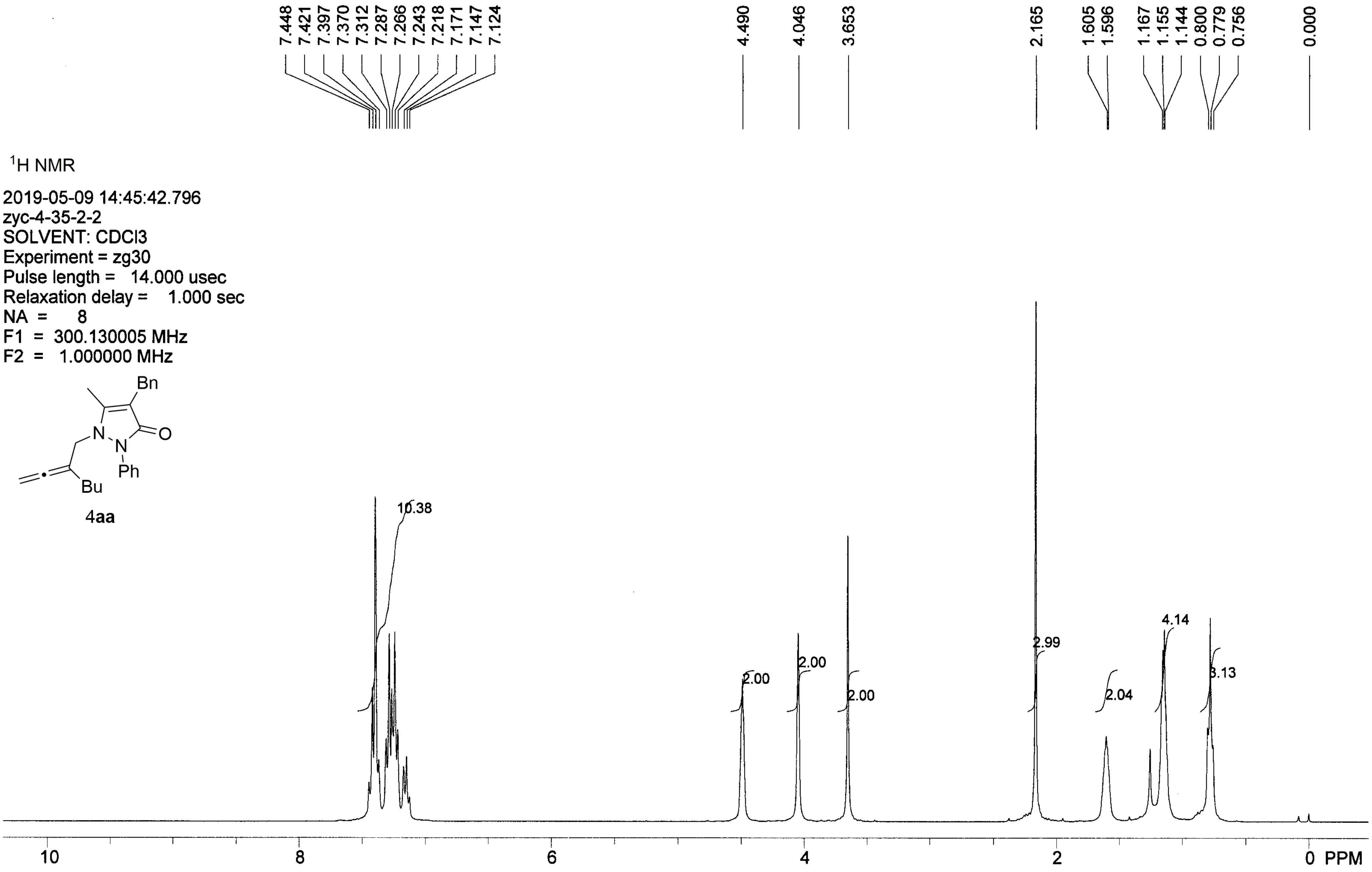

4aa

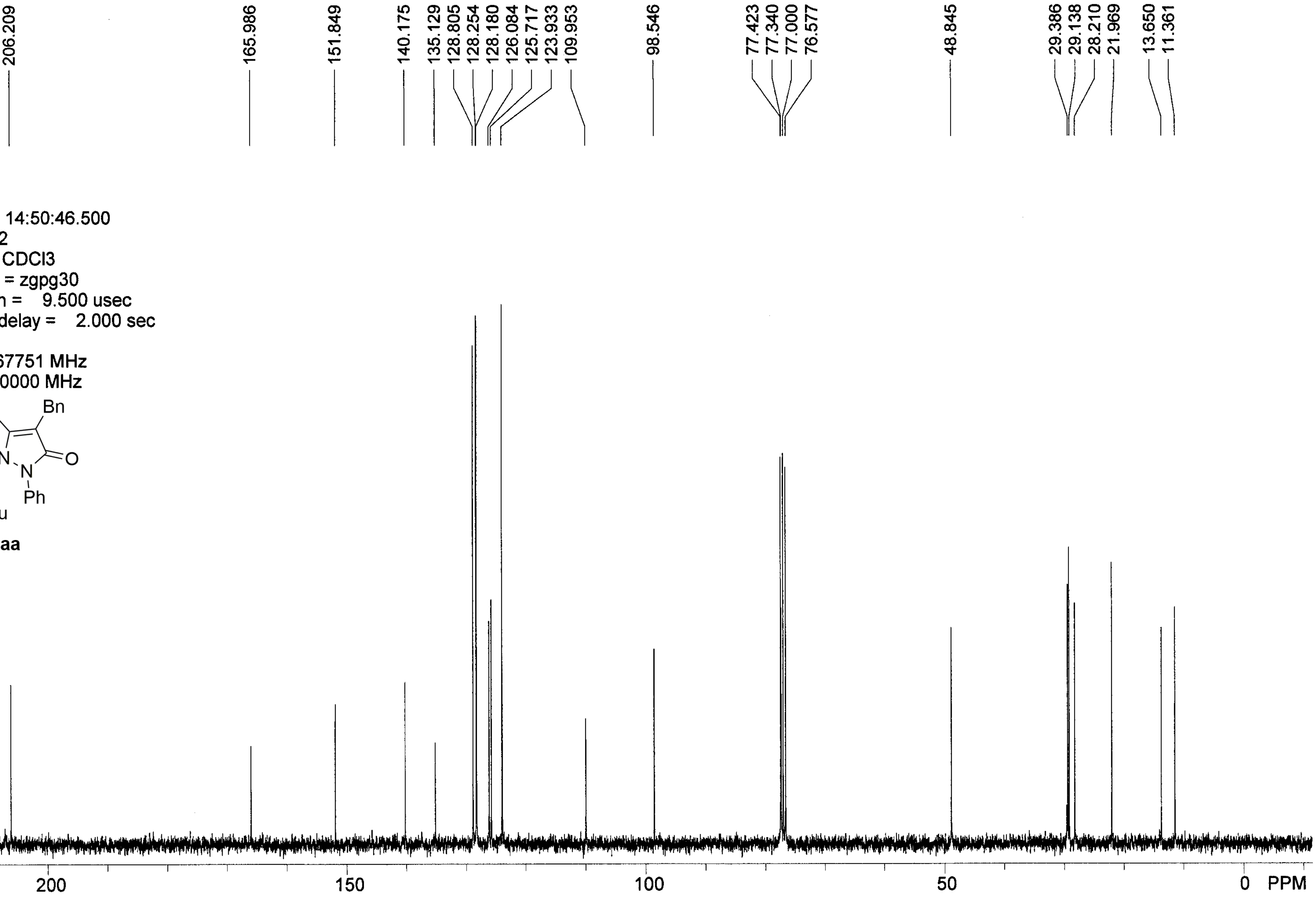

^{13}C NMR

2019-05-09 14:50:46.500
zyc-4-35-2-2
SOLVENT: CDCl3
Experiment = zgpg30
Pulse length = 9.500 usec
Relaxation delay = 2.000 sec
NA = 68
F1 = 75.467751 MHz
F2 = 1.000000 MHz

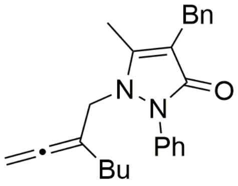

4aa

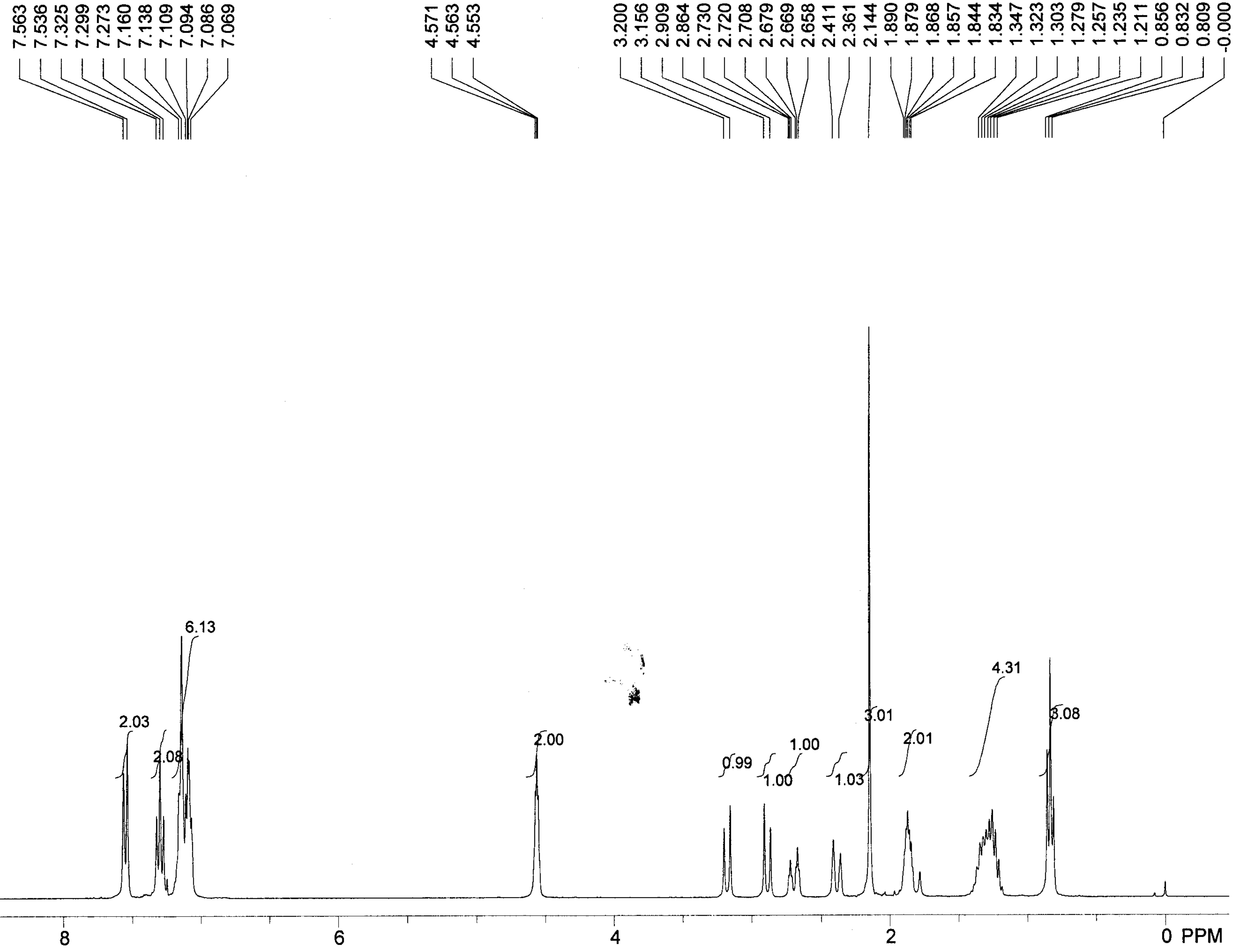

^1H NMR

2019-05-03 16:28:05.796
zyc-4-30
SOLVENT: CDCl3
Experiment = zg30
Pulse length = 14.000 usec
Relaxation delay = 1.000 sec
NA = 8
F1 = 300.130005 MHz
F2 = 1.000000 MHz

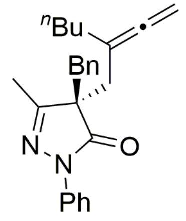

(*S*)-**3aa**

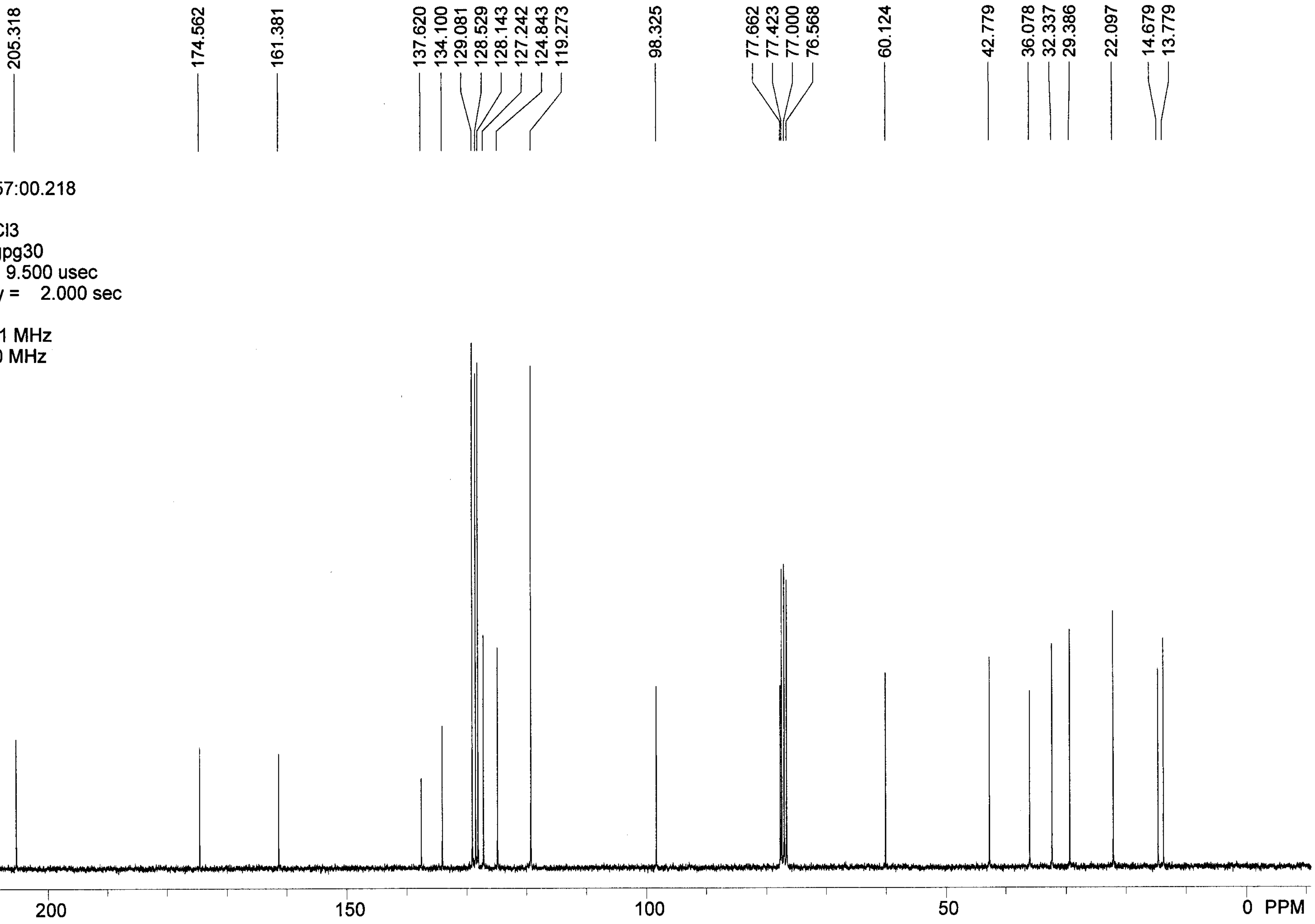

^{13}C NMR

2019-05-03 17:57:00.218
zyc-4-30
SOLVENT: CDCl3
Experiment = zgpg30
Pulse length = 9.500 usec
Relaxation delay = 2.000 sec
NA = 300
F1 = 75.467751 MHz
F2 = 1.000000 MHz

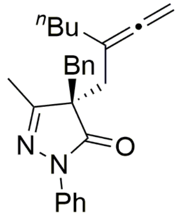

(*S*)-**3aa**

zyc-4-30

实验时间：2019-05-19,11:35:08
谱图文件:D:\浙大智达\N2000\样品\S20190519113508.org
方法文件:D:\浙大智达\N2000\djx.mtd

实验者：zyc
报告时间：2019-05-19,12:37:26
积分方法:面积归一法

实验内容简介：
ia,n-hexane/i-PrOH = 90/10,1.0,254

色谱图(S20190519113508.org)

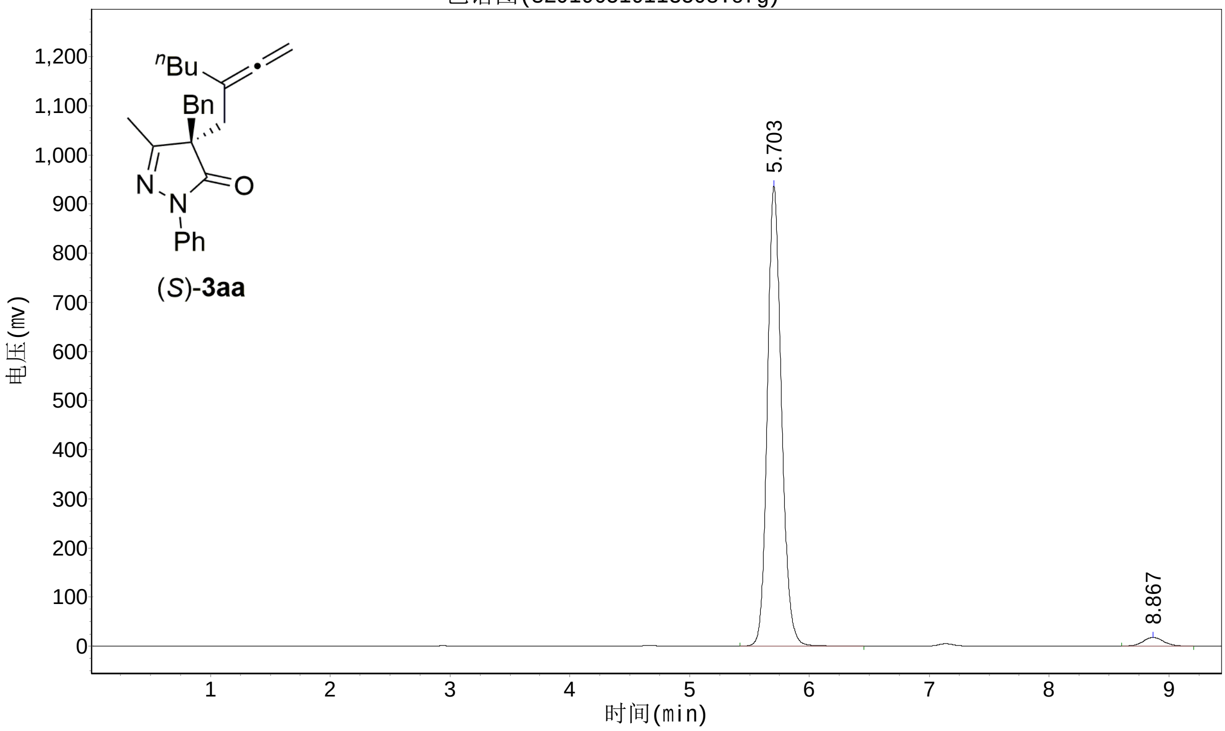

分析结果表

峰号	峰名	保留时间	峰高	峰面积	含量
1		5.703	936682.688	7651342.500	97.3107
2		8.867	17591.822	211450.703	2.6893
总计			954274.510	7862793.203	100.0000

zyc-4-30mix

实验时间：2019-05-19,11:18:06
谱图文件:D:\浙大智达\N2000\样品\S20190519111807.org
方法文件:D:\浙大智达\N2000\djx.mtd

实验者：zyc
报告时间：2019-05-19,12:14:53
积分方法:面积归一法

实验内容简介：
ia,n-hexane/i-PrOH = 90/10,1.0,254

色谱图(S20190519111807.org)

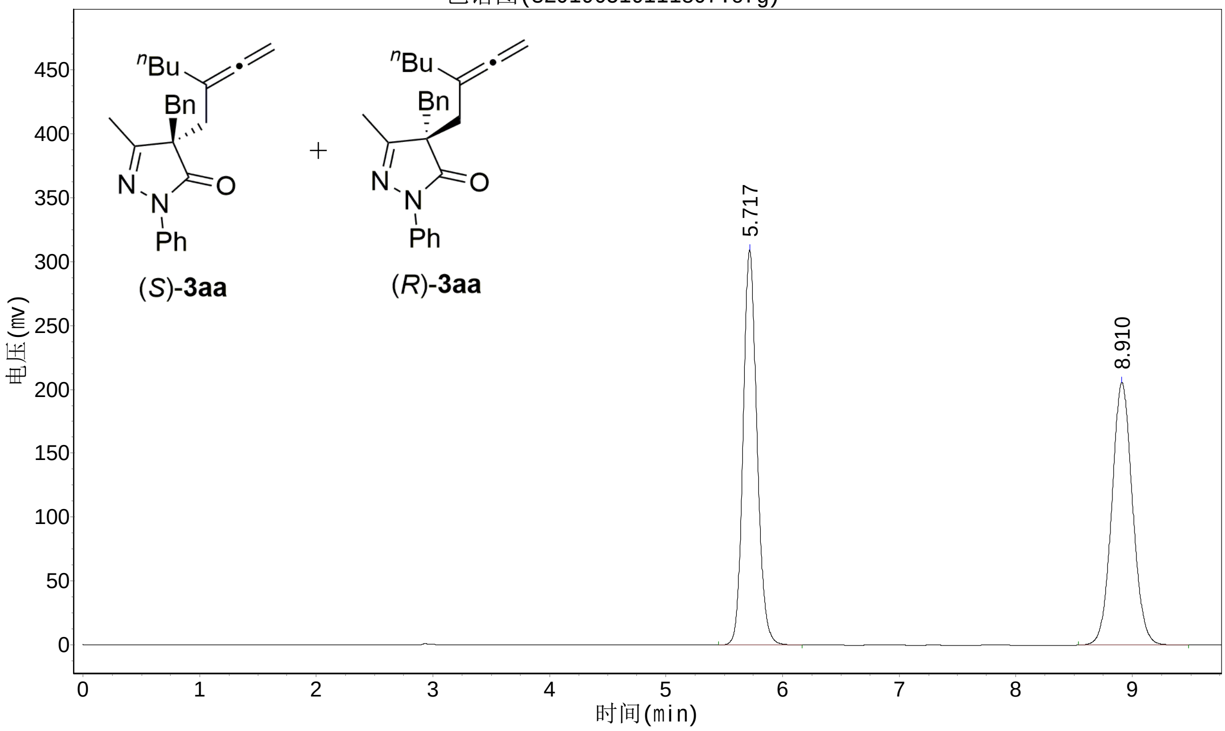

分析结果表

峰号	峰名	保留时间	峰高	峰面积	含量
1		5.717	309445.844	2550613.750	50.0845
2		8.910	205634.297	2542007.750	49.9155
总计			515080.141	5092621.500	100.0000

^{1}H NMR

2019-05-05 20:50:25.968
zyc-4-34
SOLVENT: CDCl3
Experiment = zg30
Pulse length = 14.000 usec
Relaxation delay = 1.000 sec
NA = 8
F1 = 300.130005 MHz
F2 = 1.000000 MHz

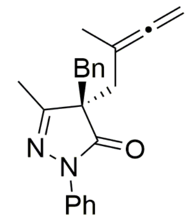

(*S*)-**3ba**

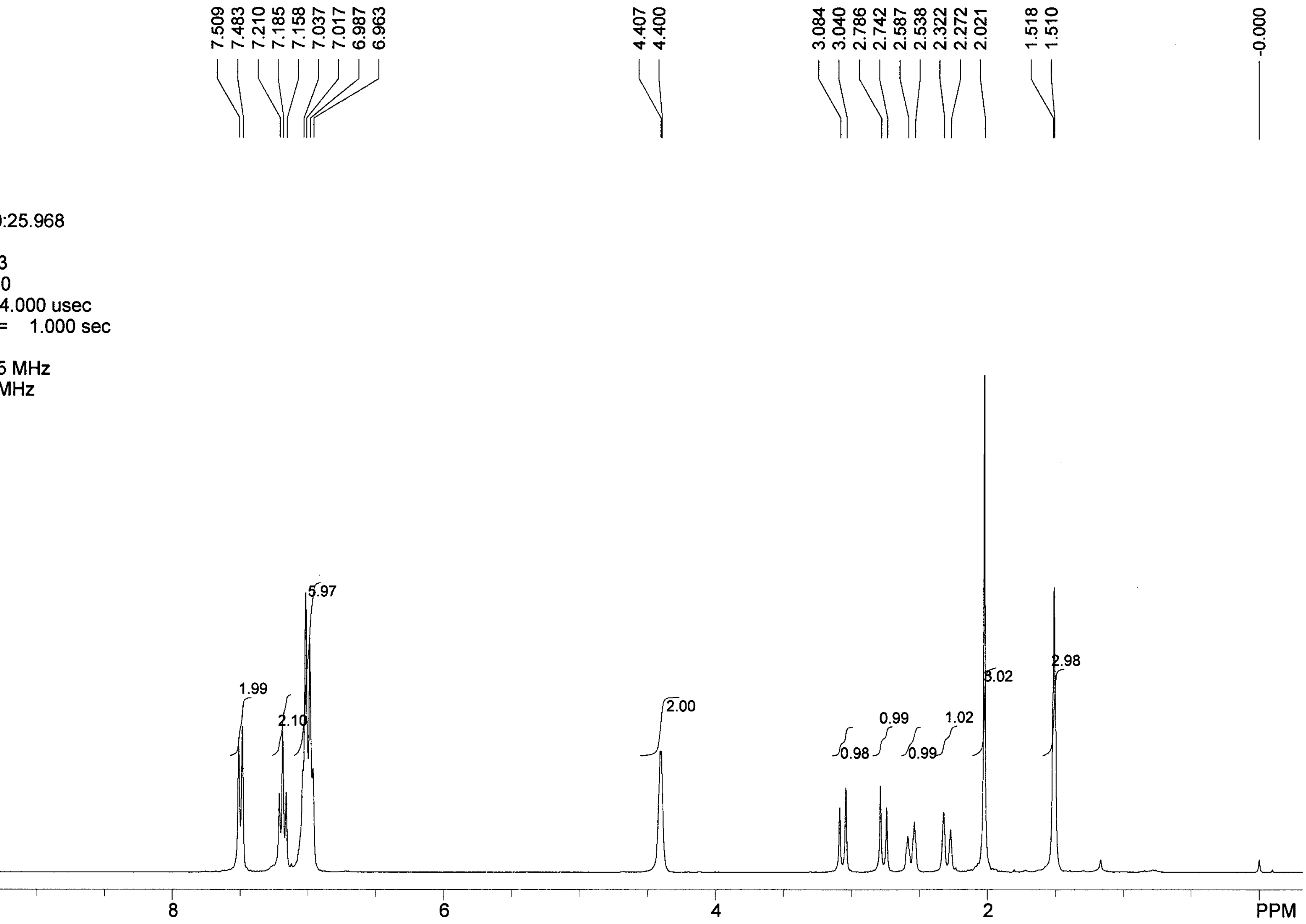

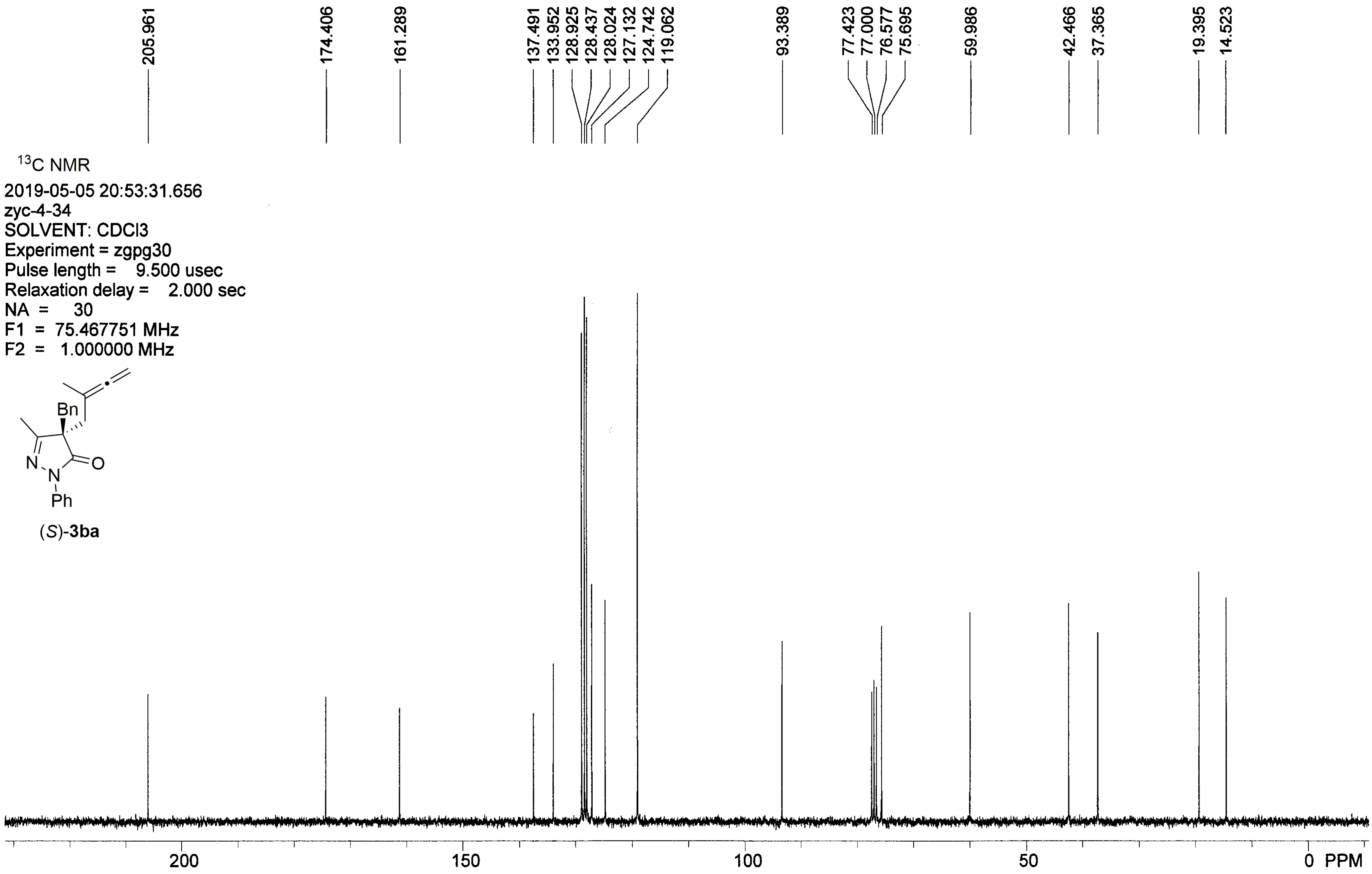
205.961
174.406
161.289
137.491
133.952
128.925
128.437
128.024
127.132
124.742
119.062
93.389
77.423
77.000
76.577
75.695
59.986
42.466
37.365
19.395
14.523
^{13}C NMR
2019-05-05 20:53:31.656
zyc-4-34
SOLVENT: CDCl3
Experiment = zgpg30
Pulse length = 9.500 usec
Relaxation delay = 2.000 sec
NA = 30
F1 = 75.467751 MHz
F2 = 1.000000 MHz
Bn
N
N
O
Ph
200
150
100
50
0 PPM

(*S*)-**3ba**

zyc-4-34

实验时间：2019-05-20,20:10:58
谱图文件:D:\浙大智达\N2000\样品\S20190520201058.org
方法文件:D:\浙大智达\N2000\djx.mtd

实验者：zyc
报告时间：2019-05-20,20:23:45
积分方法:面积归一法

实验内容简介：
ia,n-hexane/i-PrOH = 90/10,1.0,254

色谱图(S20190520201058.org)

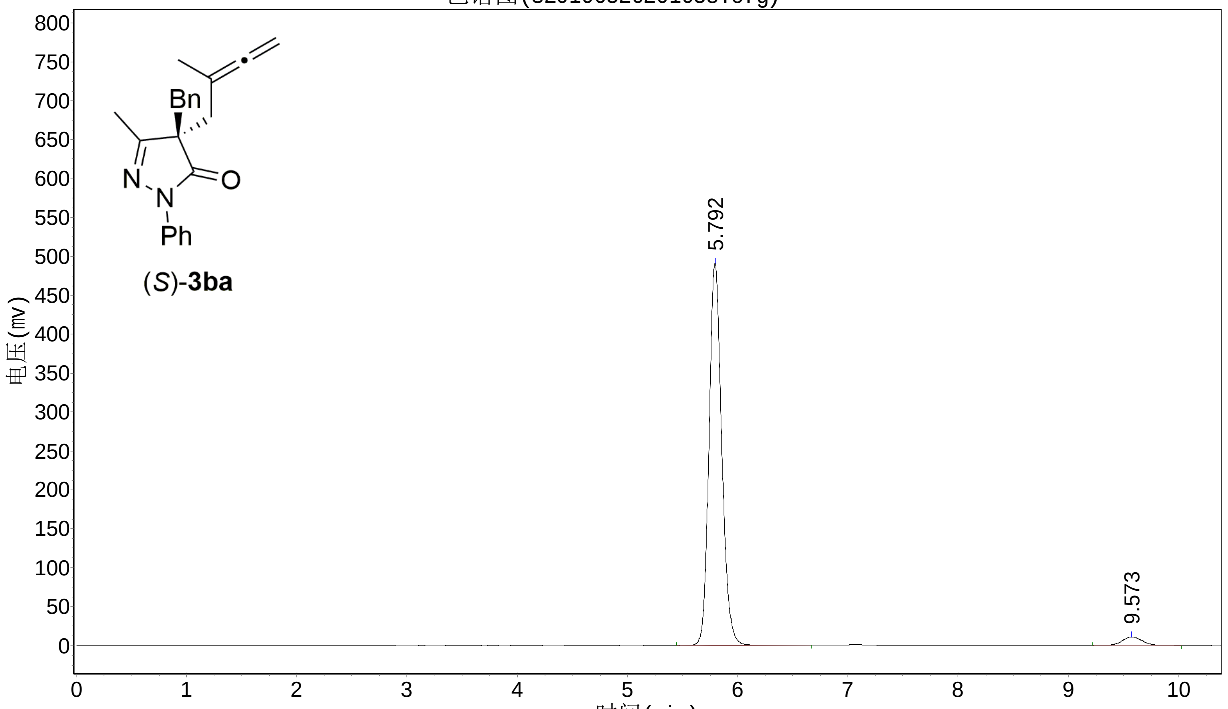

分析结果表

峰号	峰名	保留时间	峰高	峰面积	含量
1		5.792	490628.750	3938868.750	96.5803
2		9.573	10930.484	139468.156	3.4197
总计			501559.234	4078336.906	100.0000

zyc-4-34mix

实验时间：2019-05-20,19:54:36
谱图文件:D:\浙大智达\N2000\样品\S20190520195436.org
方法文件:D:\浙大智达\N2000\djx.mtd

实验者：zyc
报告时间：2019-05-20,20:09:26
积分方法:面积归一法

实验内容简介：
ia,n-hexane/i-PrOH = 90/10,1.0,254

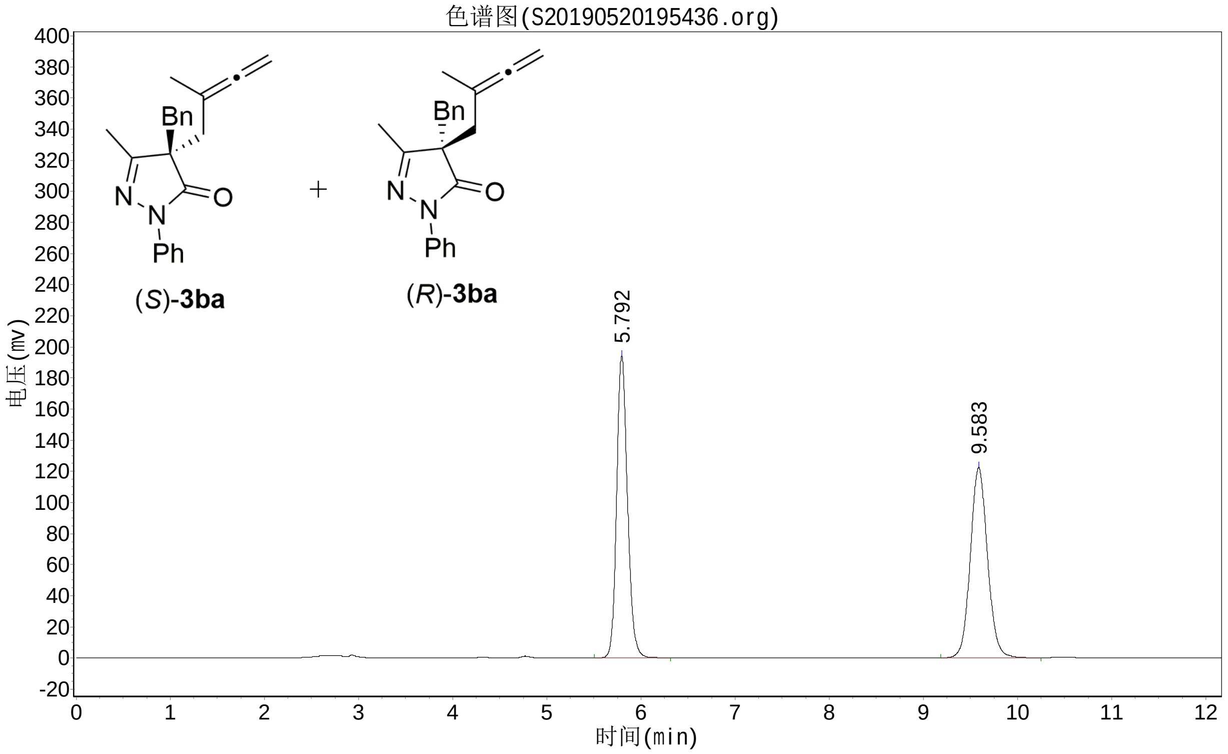

分析结果表

峰号	峰名	保留时间	峰高	峰面积	含量
1		5.792	194203.797	1547263.750	49.9556
2		9.583	122342.867	1550013.250	50.0444
总计			316546.664	3097277.000	100.0000

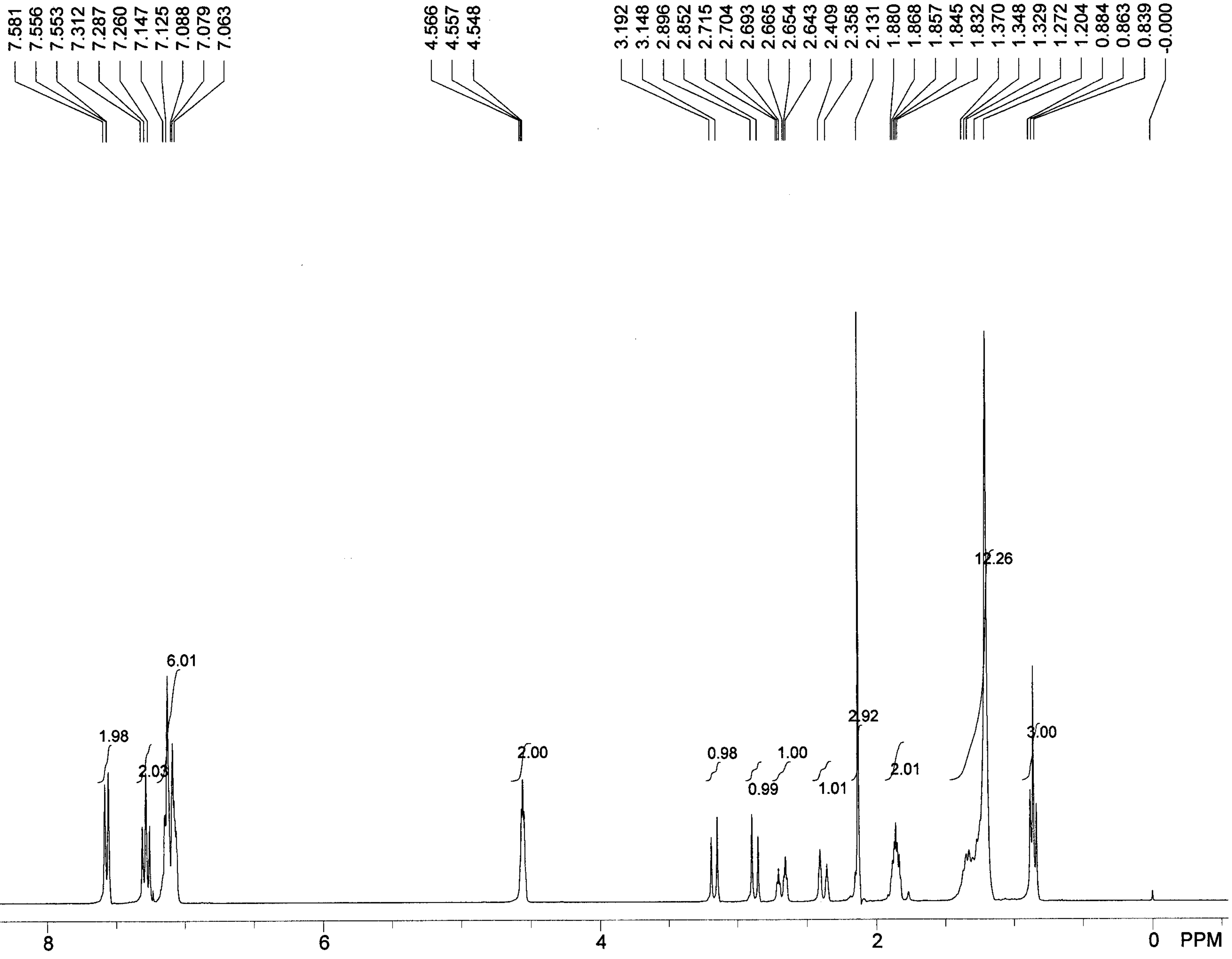

^1H NMR

2019-05-12 16:24:52.328
zyc-4-45
SOLVENT: CDCl3
Experiment = zg30
Pulse length = 14.000 usec
Relaxation delay = 1.000 sec
NA = 8
F1 = 300.130005 MHz
F2 = 1.000000 MHz

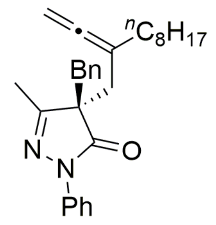

(*S*)-**3ca**

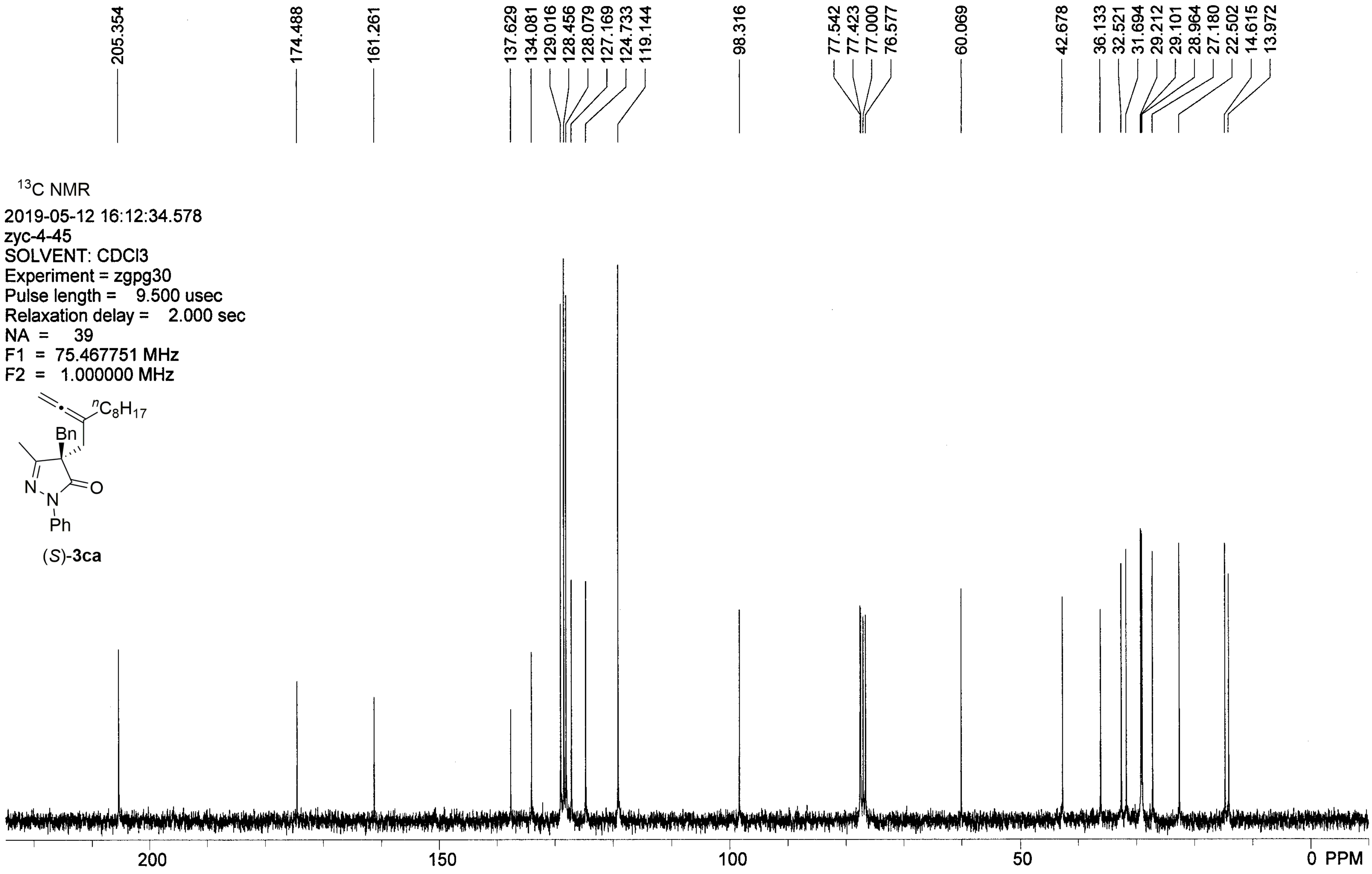
205.354
174.488
161.261
137.629
134.081
129.016
128.456
128.079
127.169
124.733
119.144
98.316
77.542
77.423
77.000
76.577
60.069
42.678
36.133
32.521
31.694
29.212
29.101
28.964
27.180
22.502
14.615
13.972
^{13}C NMR
2019-05-12 16:12:34.578
zyc-4-45
SOLVENT: CDCl3
Experiment = zgpg30
Pulse length = 9.500 usec
Relaxation delay = 2.000 sec
NA = 39
F1 = 75.467751 MHz
F2 = 1.000000 MHz
200
150
100
50
0 PPM
$^{n}C_8H_{17}$
Bn
N
N
O
Ph

(*S*)-**3ca**

zyc-4-45

实验时间：2019-05-21,18:45:41
谱图文件:D:\浙大智达\N2000\样品\S20190521184541.org
方法文件:D:\浙大智达\N2000\djx.mtd

实验者：zyc
报告时间：2019-05-21,18:58:39
积分方法:面积归一法

实验内容简介：
ia,n-hexane/i-PrOH = 90/10,1.0,254

色谱图(S20190521184541.org)

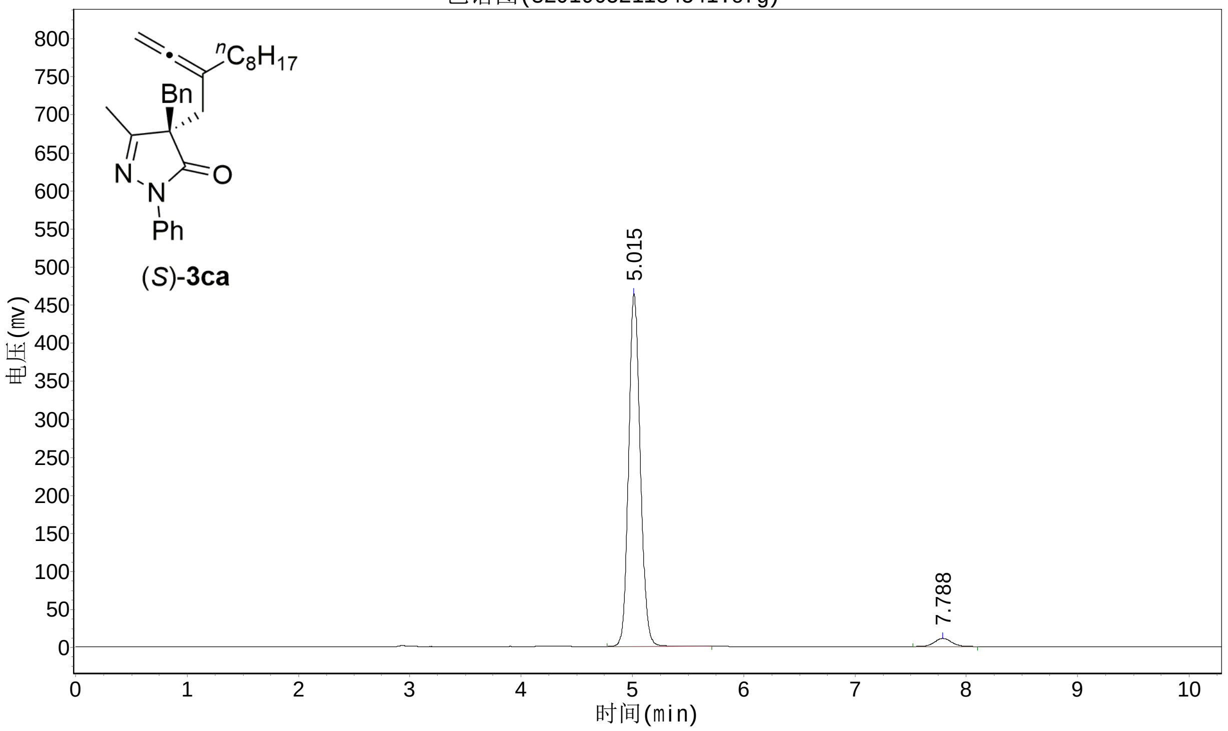

分析结果表

峰号	峰名	保留时间	峰高	峰面积	含量
1		5.015	463735.188	3374730.250	96.7350
2		7.788	10614.591	113905.102	3.2650
总计			474349.778	3488635.352	100.0000

zyc-4-45mix

实验时间：2019-05-21,18:32:09
谱图文件:D:\浙大智达\N2000\样品\S20190521183209.org
方法文件:D:\浙大智达\N2000\djx.mtd

实验者：zyc
报告时间：2019-05-21,18:44:17
积分方法:面积归一法

实验内容简介：
ia,n-hexane/i-PrOH = 90/10,1.0,254

色谱图(S20190521183209.org)

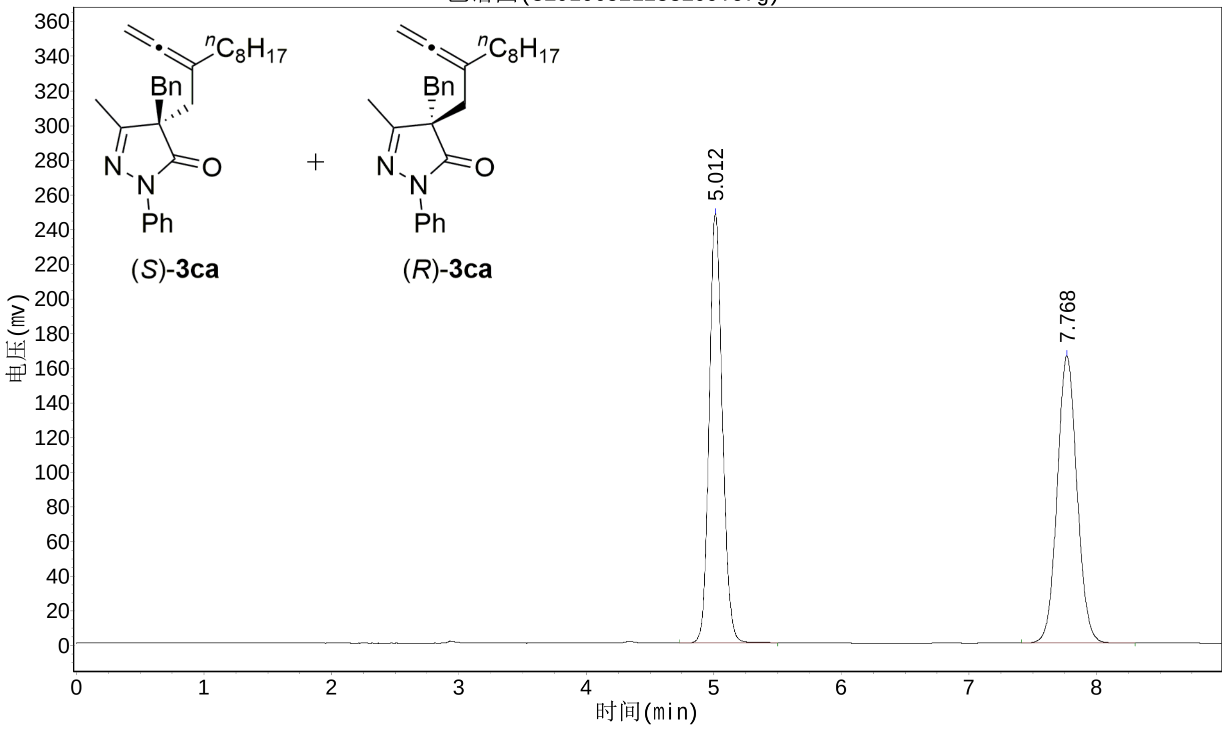

分析结果表

峰号	峰名	保留时间	峰高	峰面积	含量
1		5.012	247577.016	1780434.875	49.8839
2		7.768	165684.313	1788719.250	50.1161
总计			413261.328	3569154.125	100.0000

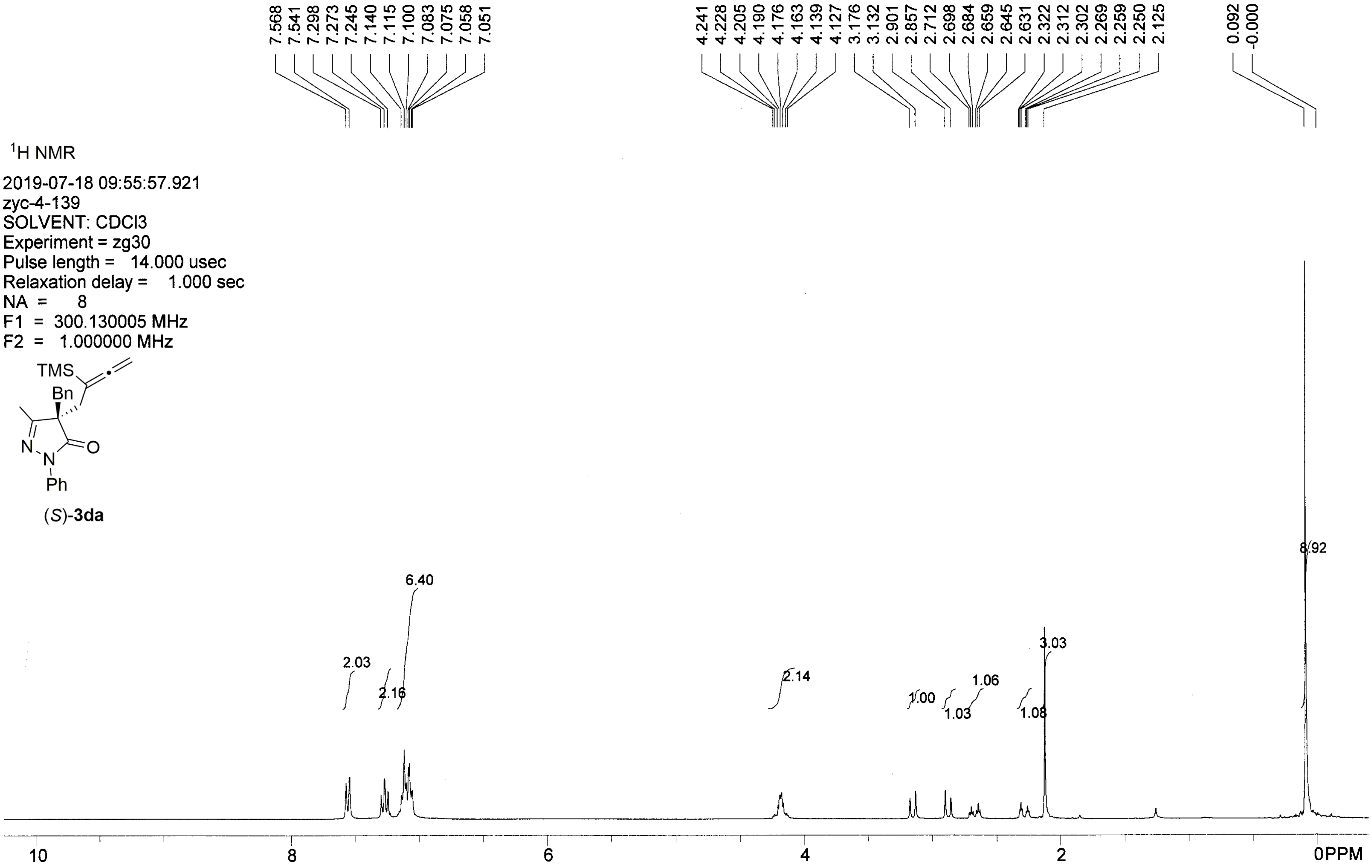

^{1}H NMR

2019-07-18 09:55:57.921
zyc-4-139
SOLVENT: CDCl3
Experiment = zg30
Pulse length = 14.000 usec
Relaxation delay = 1.000 sec
NA = 8
F1 = 300.130005 MHz
F2 = 1.000000 MHz

(*S*)-**3da**

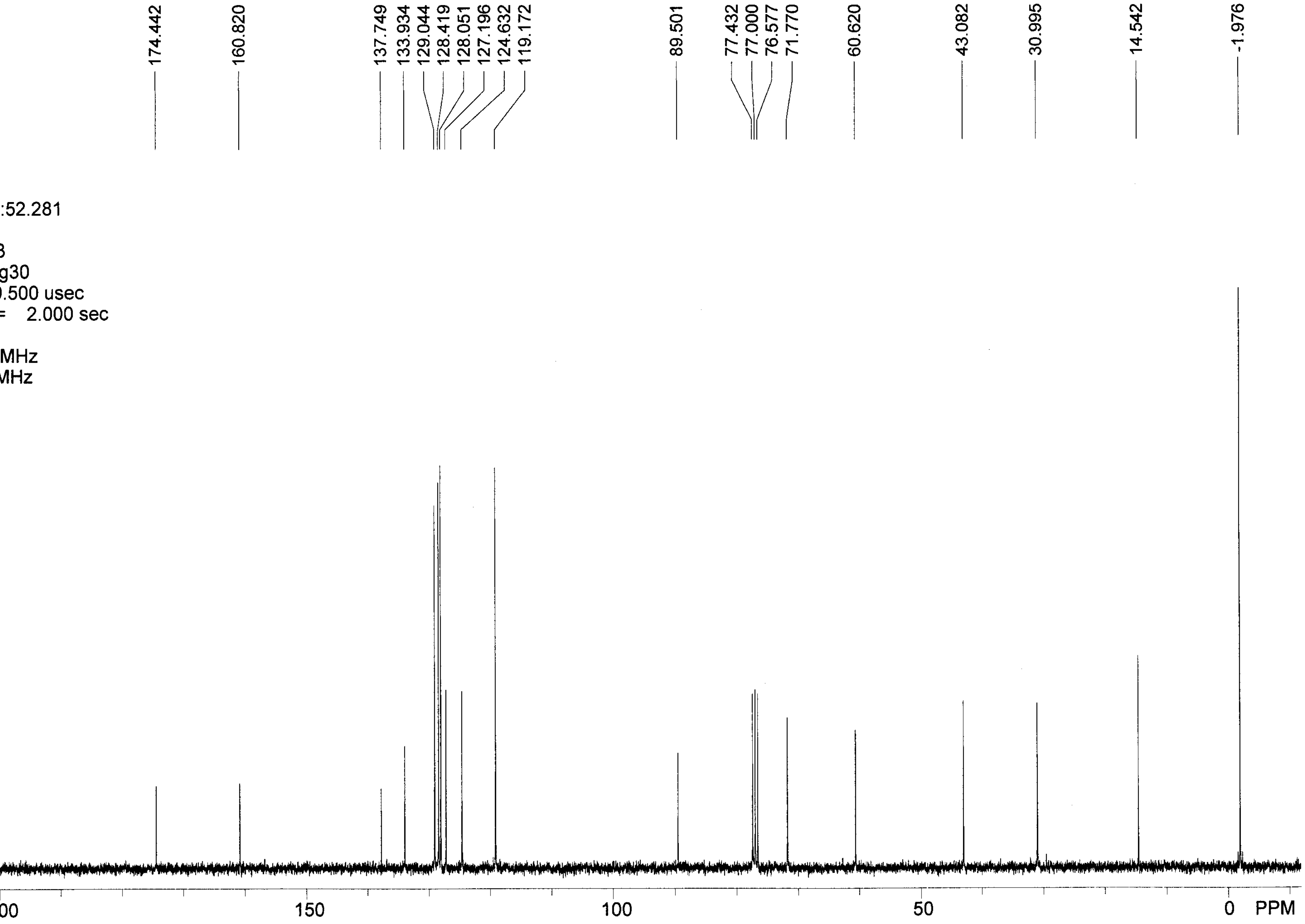

^{13}C NMR

2019-07-18 09:58:52.281
zyc-4-139
SOLVENT: CDCl3
Experiment = zgpg30
Pulse length = 9.500 usec
Relaxation delay = 2.000 sec
NA = 33
F1 = 75.467751 MHz
F2 = 1.000000 MHz

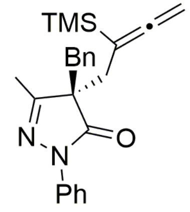

(*S*)-**3da**

zyc-4-139

实验时间：2019-07-17,21:08:04
谱图文件:D:\浙大智达\N2000\样品\S20190717210804.org
方法文件:D:\浙大智达\N2000\djx.mtd

实验者：zyc
报告时间：2019-07-17,21:18:31
积分方法:面积归一法

实验内容简介：
ia,n-hexane/i-PrOH = 90/10,1.0,254

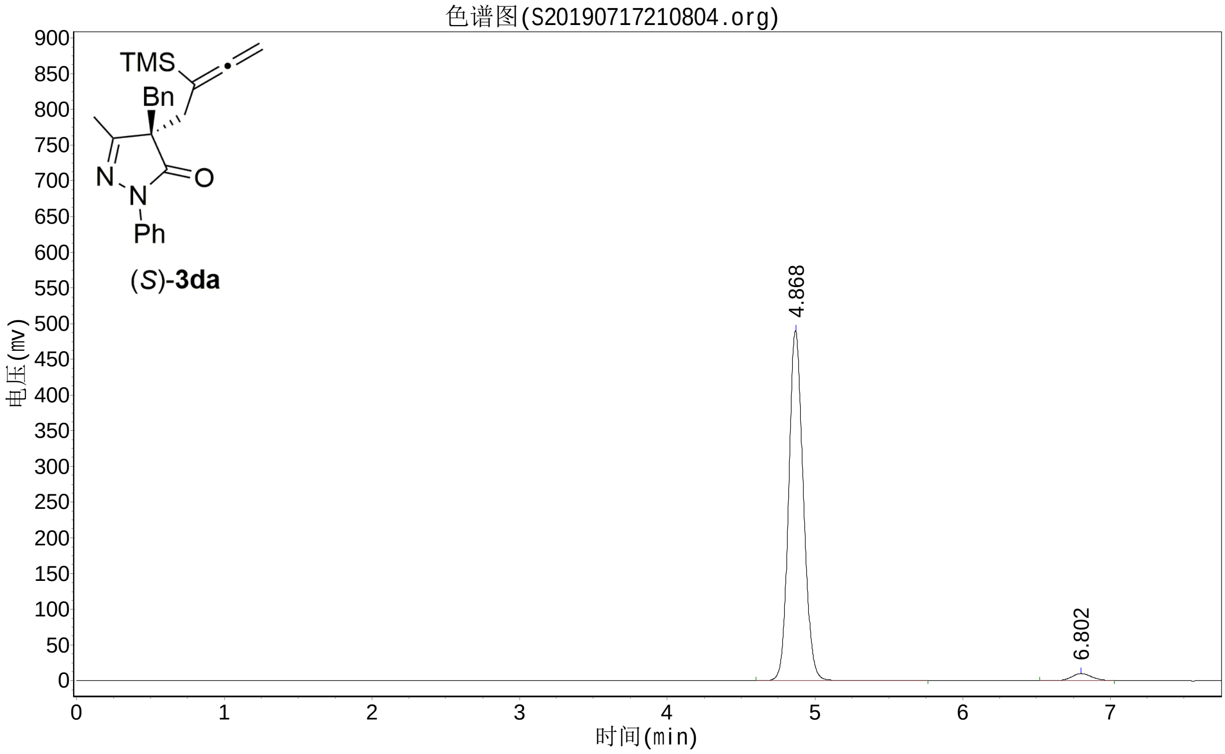

分析结果表

峰号	峰名	保留时间	峰高	峰面积	含量
1		4.868	490760.313	3380853.000	97.2132
2		6.802	10283.647	96917.813	2.7868
总计			501043.960	3477770.813	100.0000

zyc-4-139mix

实验时间：2019-07-17,21:31:46
谱图文件:D:\浙大智达\N2000\样品\S20190717213146.org
方法文件:D:\浙大智达\N2000\djx.mtd

实验者：zyc
报告时间：2019-07-17,21:42:20
积分方法:面积归一法

实验内容简介：
ia,n-hexane/i-PrOH = 90/10,1.0,254

色谱图(S20190717213146.org)

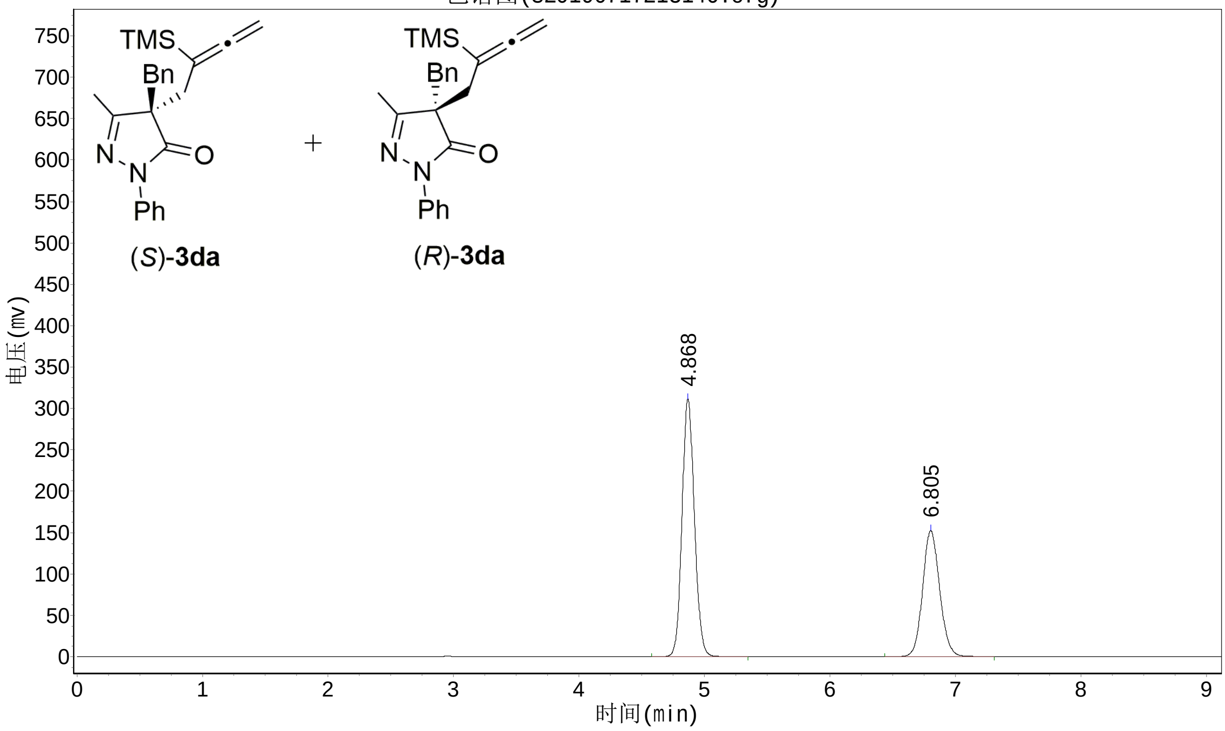

分析结果表

峰号	峰名	保留时间	峰高	峰面积	含量
1		4.868	311667.719	2141256.000	59.6531
2		6.805	152632.969	1448256.500	40.3469
总计			464300.688	3589512.500	100.0000

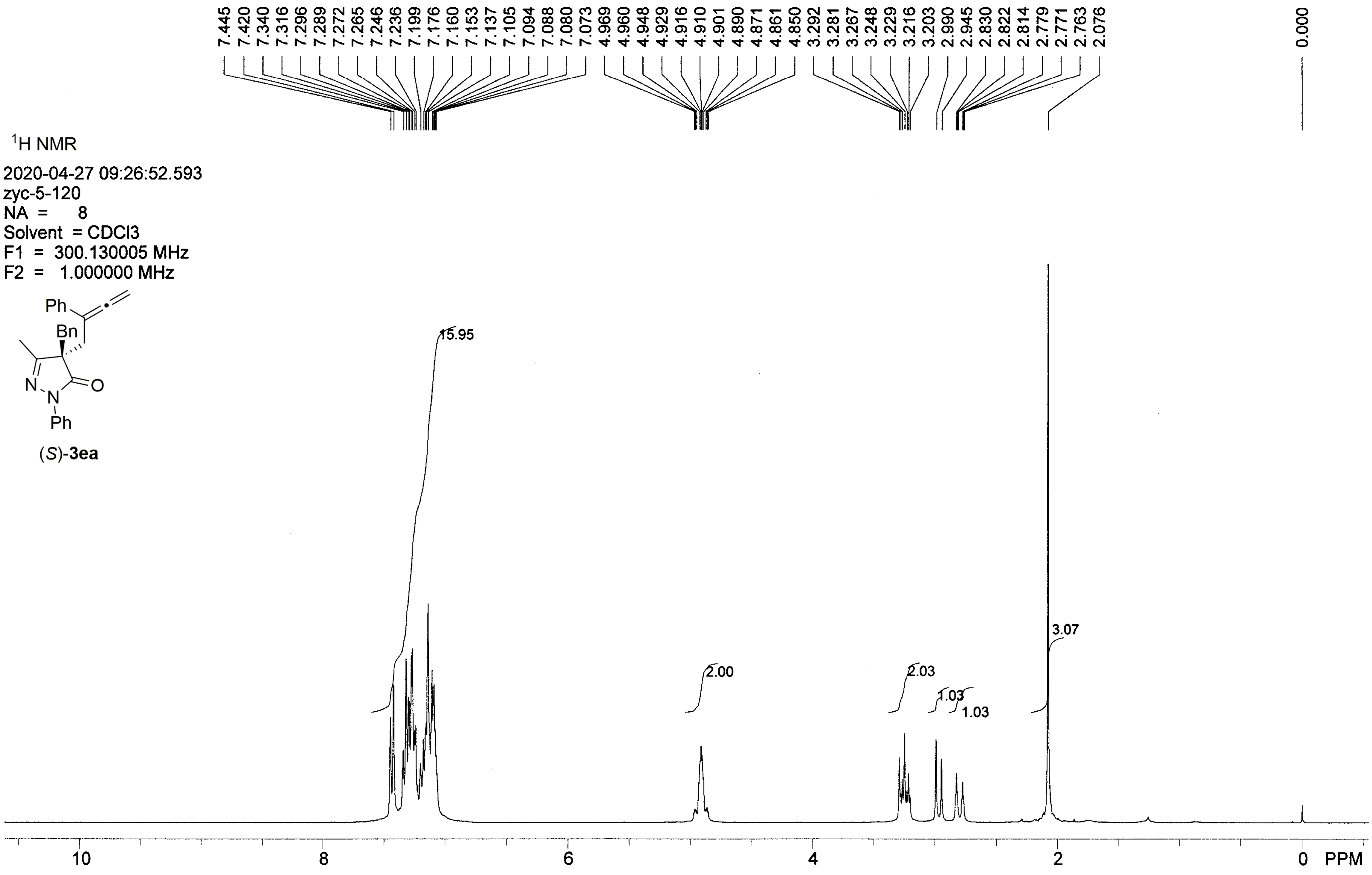
7.445
7.420
7.340
7.316
7.296
7.289
7.272
7.265
7.246
7.236
7.199
7.176
7.160
7.153
7.137
7.105
7.094
7.088
7.080
7.073
4.969
4.960
4.948
4.929
4.916
4.910
4.901
4.890
4.871
4.861
4.850
3.292
3.281
3.267
3.248
3.229
3.216
3.203
2.990
2.945
2.830
2.822
2.814
2.779
2.771
2.763
2.076
0.000
^{1}H NMR
2020-04-27 09:26:52.593
zyc-5-120
NA = 8
Solvent = CDCl3
F1 = 300.130005 MHz
F2 = 1.000000 MHz
Ph
Bn
N
N
O
Ph
(S)-3ea
15.95
2.00
2.03
1.03
1.03
3.07
10
8
6
4
2
0
PPM

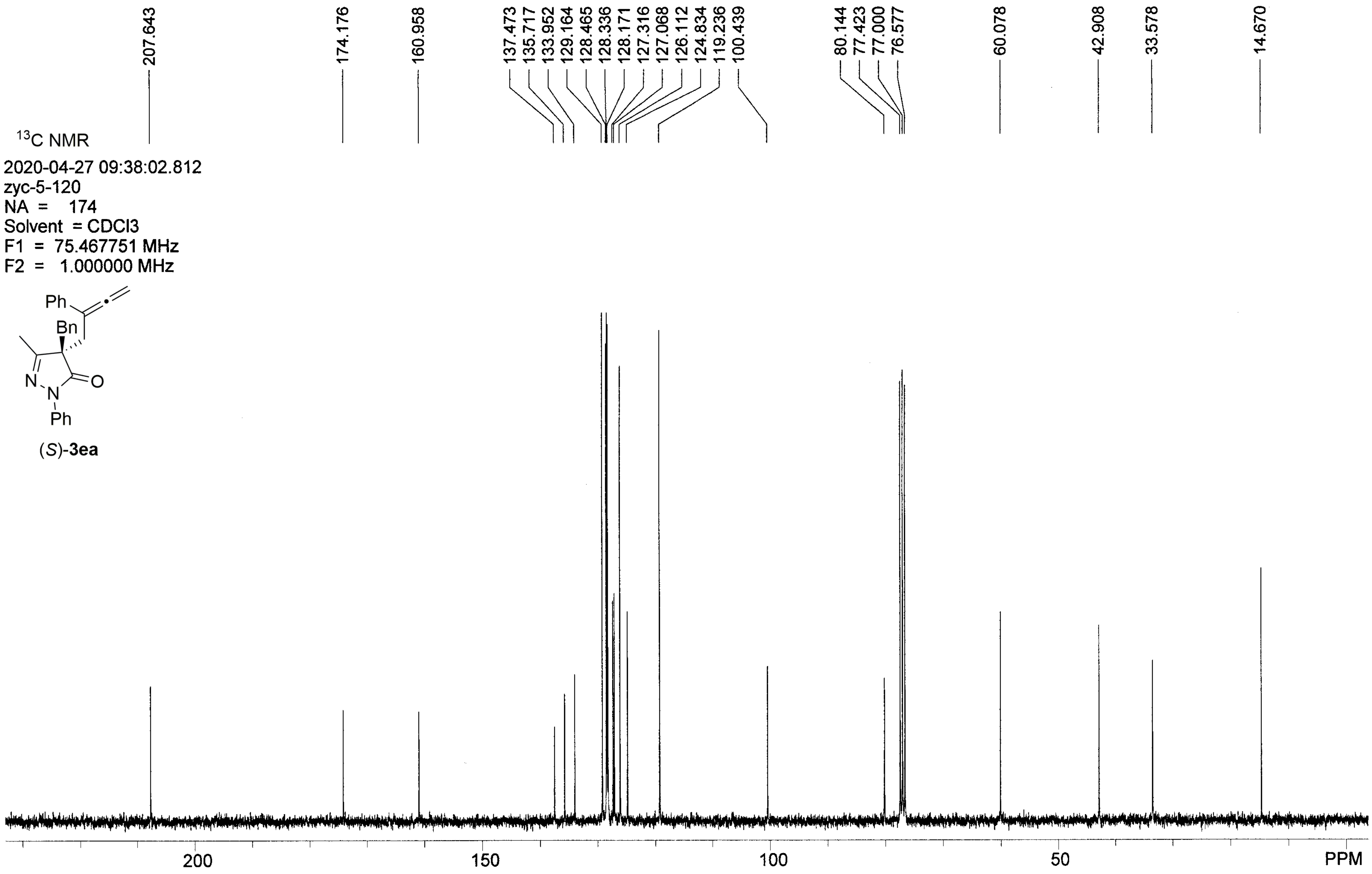
207.643
174.176
160.958
137.473
135.717
133.952
129.164
128.465
128.336
128.171
127.316
127.068
126.112
124.834
119.236
100.439
80.144
77.423
77.000
76.577
60.078
42.908
33.578
14.670
^{13}C NMR
2020-04-27 09:38:02.812
zyc-5-120
NA = 174
Solvent = CDCl3
F1 = 75.467751 MHz
F2 = 1.000000 MHz
Ph
Bn
N
N
O
Ph
(S)-3ea
200
150
100
50
PPM

zyc-5-120

实验时间：2020-04-27,12:27:55
谱图文件:D:\浙大智达\N2000\样品\S20200427122755.org
方法文件:D:\浙大智达\N2000\djx.mtd

实验者：zyc
报告时间：2020-04-27,13:15:26
积分方法:面积归一法

实验内容简介：
ia,n-hexane/i-PrOH = 90/10,1.0,254

色谱图(S20200427122755.org)

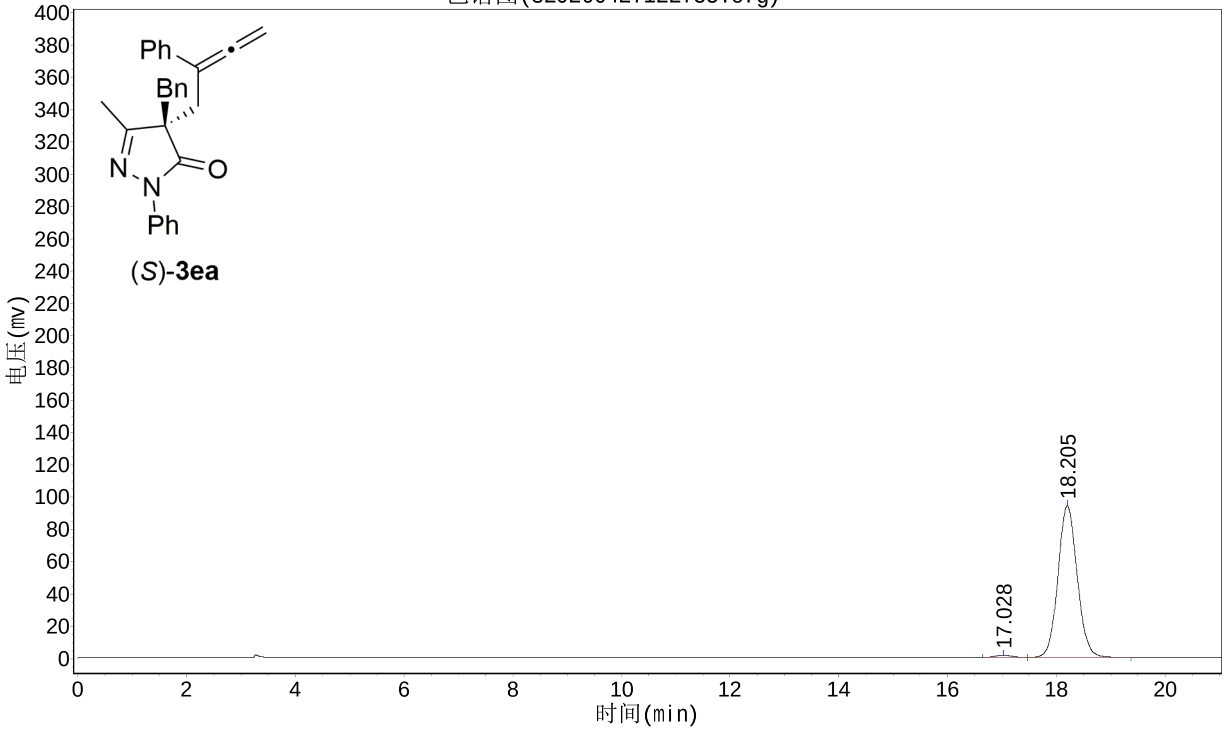

分析结果表

峰号	峰名	保留时间	峰高	峰面积	含量
1		17.028	1473.712	31425.002	1.3357
2		18.205	94198.297	2321241.500	98.6643
总计			95672.009	2352666.502	100.0000

zyc-5-120mix

实验时间：2020-04-27,13:25:19
谱图文件:D:\浙大智达\N2000\样品\S20200427132519.org
方法文件:D:\浙大智达\N2000\djx.mtd

实验者：zyc
报告时间：2020-04-27,13:49:38
积分方法:面积归一法

实验内容简介：
ia,n-hexane/i-PrOH = 90/10,1.0,254

色谱图(S20200427132519.org)

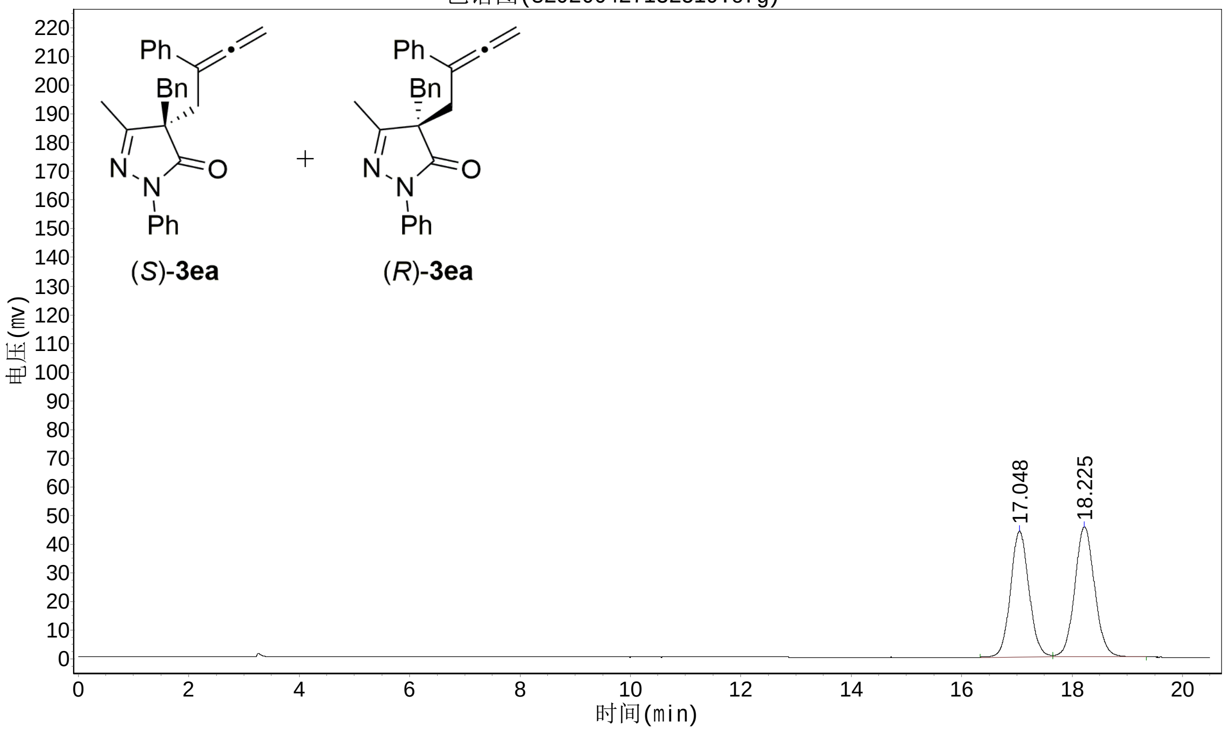

分析结果表

峰号	峰名	保留时间	峰高	峰面积	含量
1		17.048	43929.574	1022159.563	47.2614
2		18.225	45472.398	1140620.000	52.7386
总计			89401.973	2162779.563	100.0000

2021-01-10 19:11:13.437
zyc-6-154
NA = 8
Solvent = CDCl3
F1 = 300.130005 MHz
F2 = 1.000000 MHz

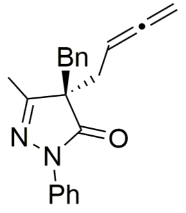

(*R*)-**3fa**

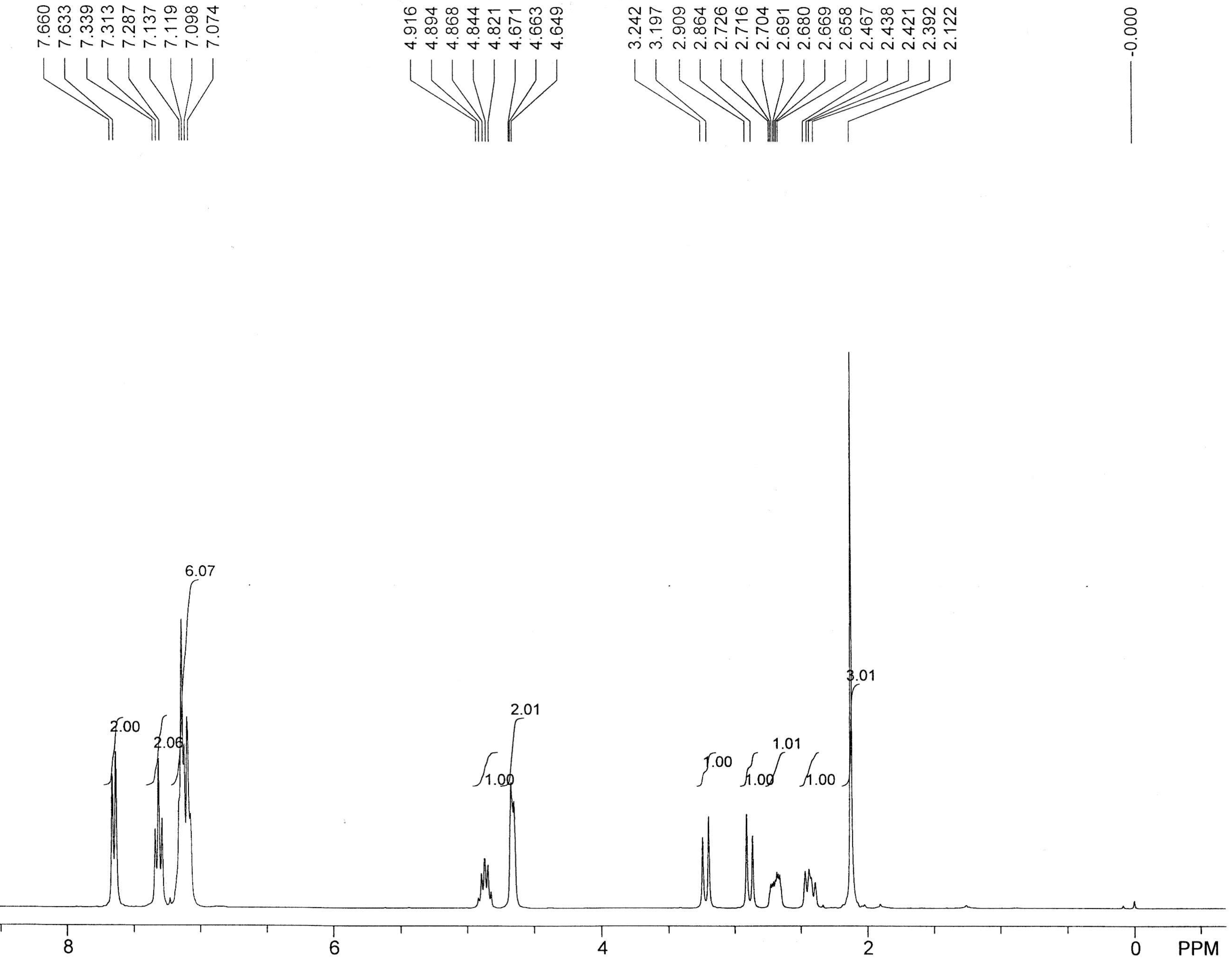

2021-01-10 19:19:56.453
zyc-6-154
NA = 125
Solvent = CDCl3
F1 = 75.467751 MHz
F2 = 1.000000 MHz

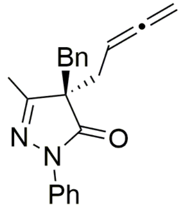

(*R*)-**3fa**

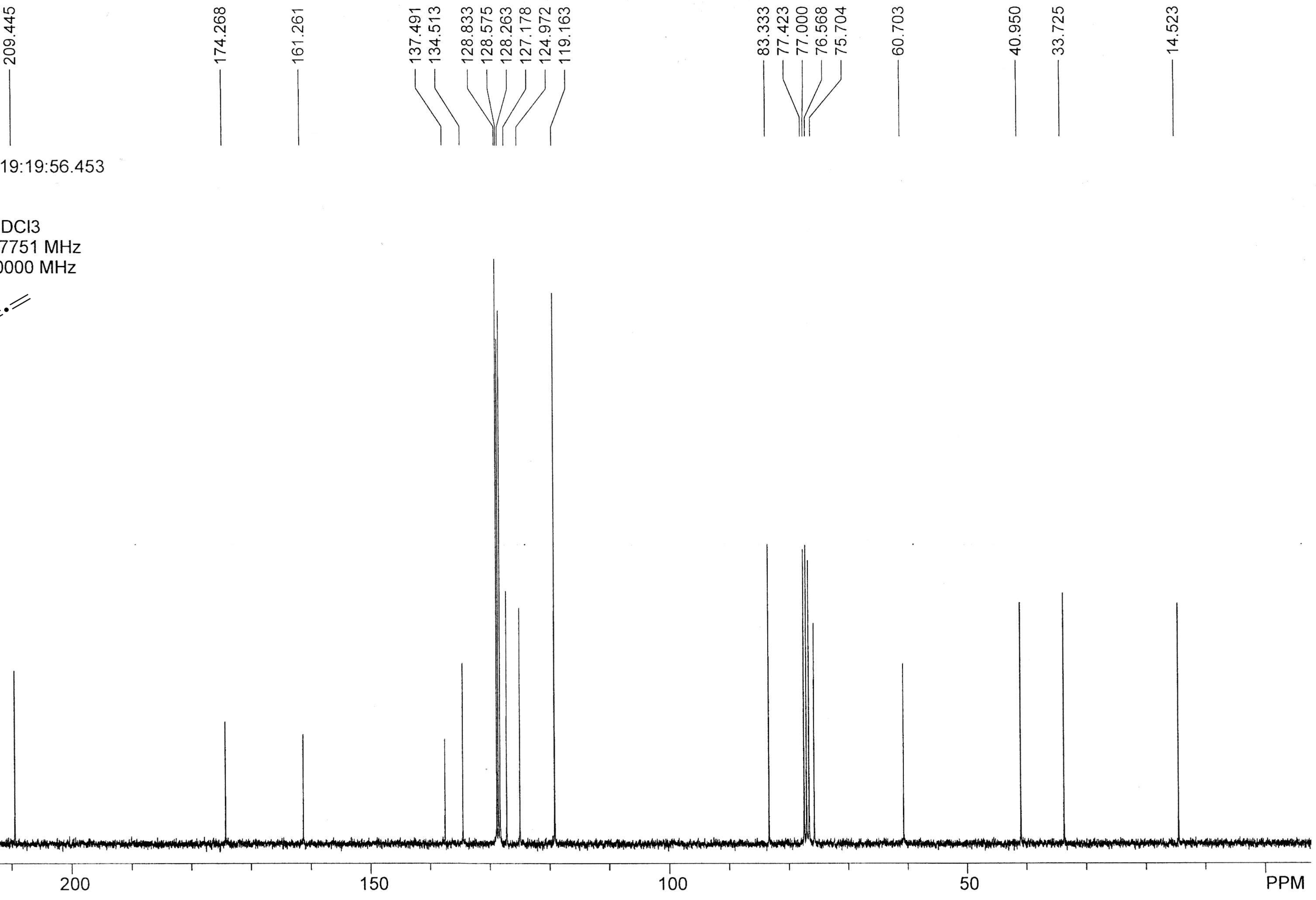

zyc-6-154

实验时间：2021-01-10,18:28:35
谱图文件:D:\浙大智达\N2000\样品\S20210110182835.org
方法文件:D:\浙大智达\N2000\djx.mtd

实验者：zyc
报告时间：2021-01-10,19:01:17
积分方法:面积归一法

实验内容简介：
ia,n-hexane/i-PrOH = 90/10,1.0,254

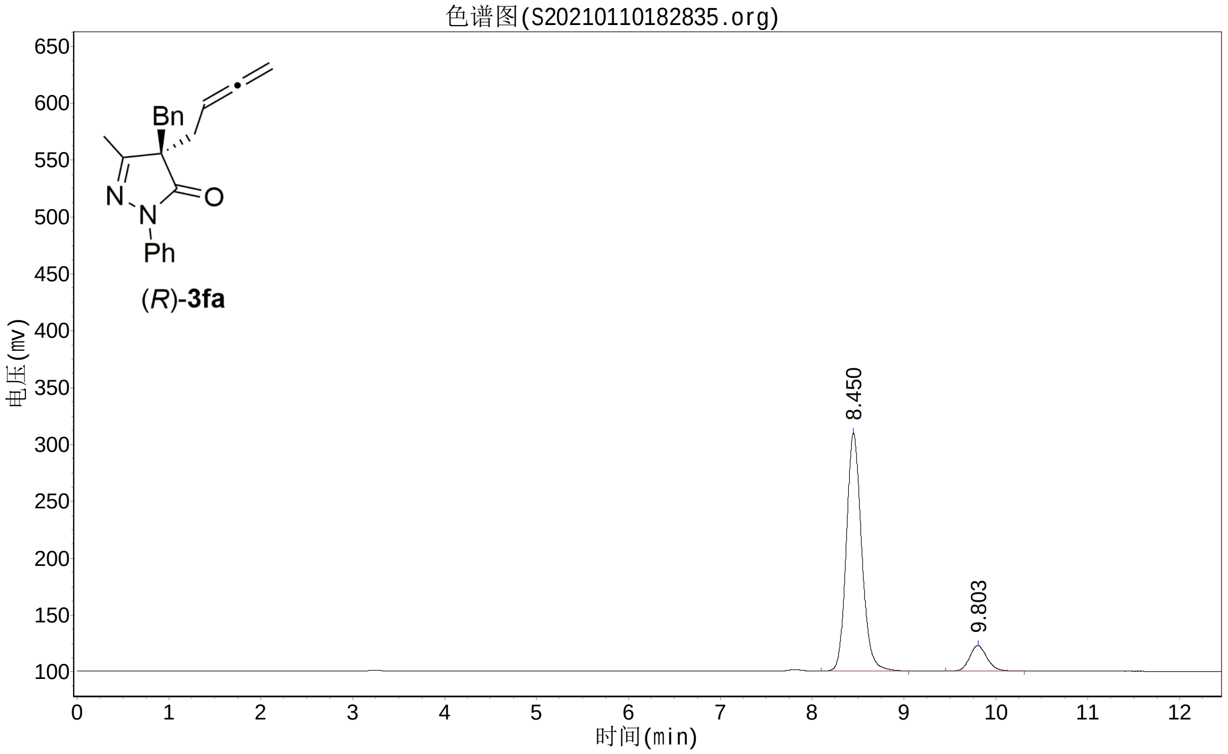

分析结果表

峰号	峰名	保留时间	峰高	峰面积	含量
1		8.450	209444.281	2424035.500	89.0216
2		9.803	22530.186	298938.938	10.9784
总计			231974.467	2722974.438	100.0000

zyc-6-154mix

实验时间：2021-01-10,18:49:11
谱图文件:D:\浙大智达\N2000\样品\S20210110184911.org
方法文件:D:\浙大智达\N2000\djx.mtd

实验者：zyc
报告时间：2021-01-10,19:04:02
积分方法:面积归一法

实验内容简介：
ia,n-hexane/i-PrOH = 90/10,1.0,254

色谱图(S20210110184911.org)

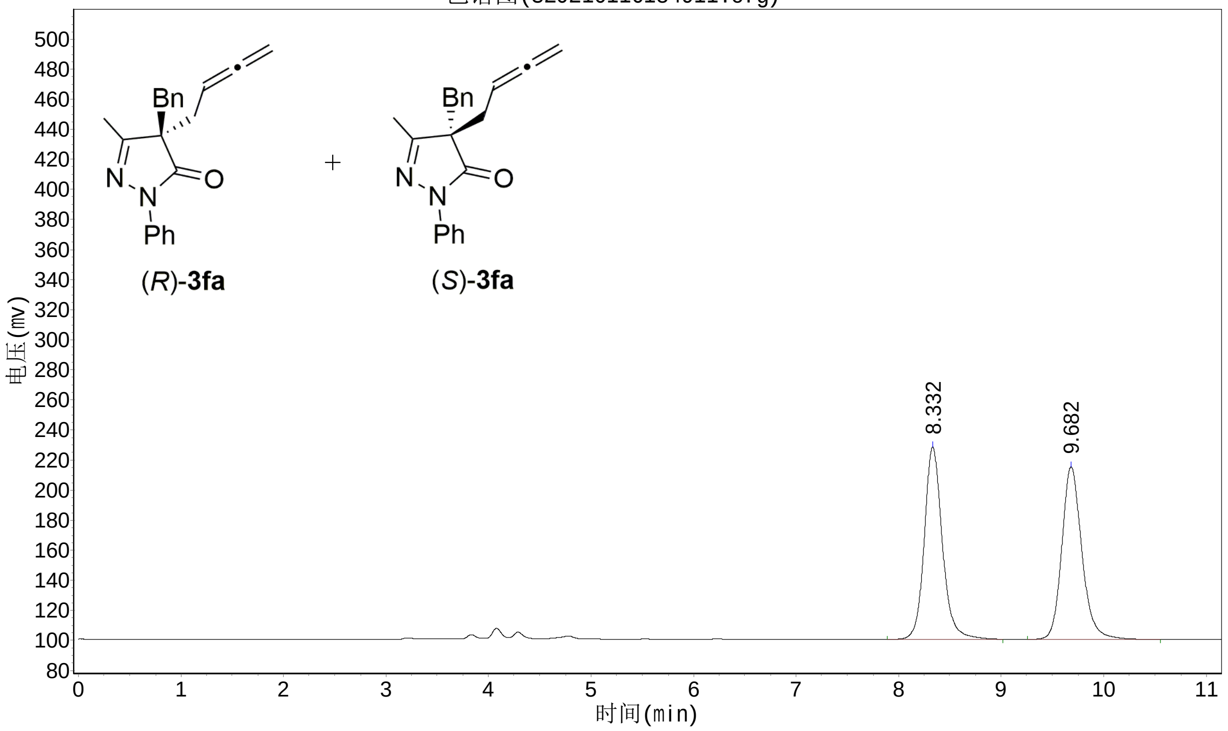

分析结果表

峰号	峰名	保留时间	峰高	峰面积	含量
1		8.332	128078.477	1566558.125	50.0254
2		9.682	114799.172	1564965.375	49.9746
总计			242877.648	3131523.500	100.0000

^{1}H NMR

2019-05-15 20:00:14.796
zyc-4-50
SOLVENT: CDCl3
Experiment = zg30
Pulse length = 14.000 usec
Relaxation delay = 1.000 sec
NA = 8
F1 = 300.130005 MHz
F2 = 1.000000 MHz

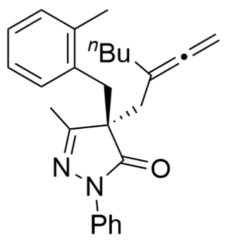

(*R*)-**3ab**

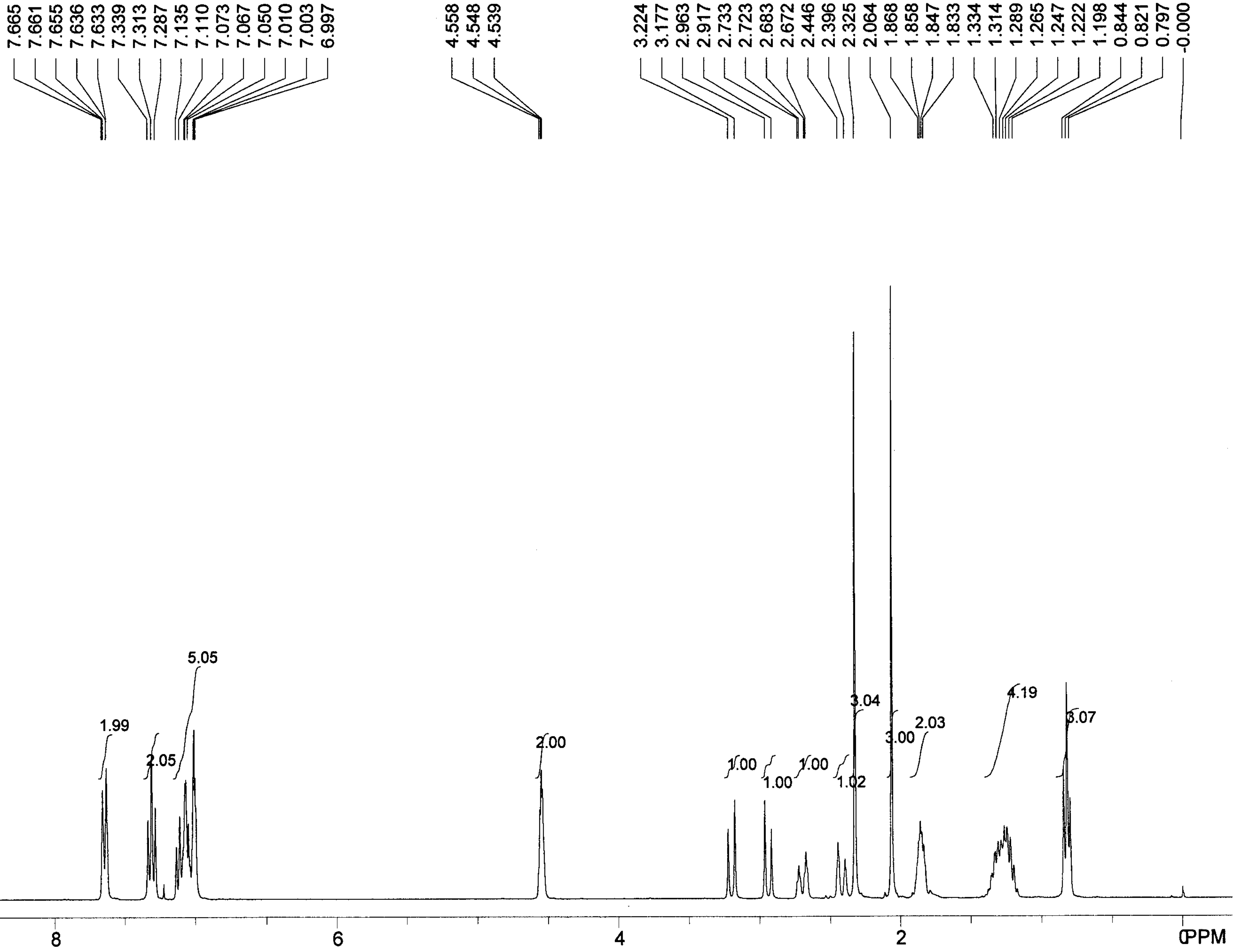

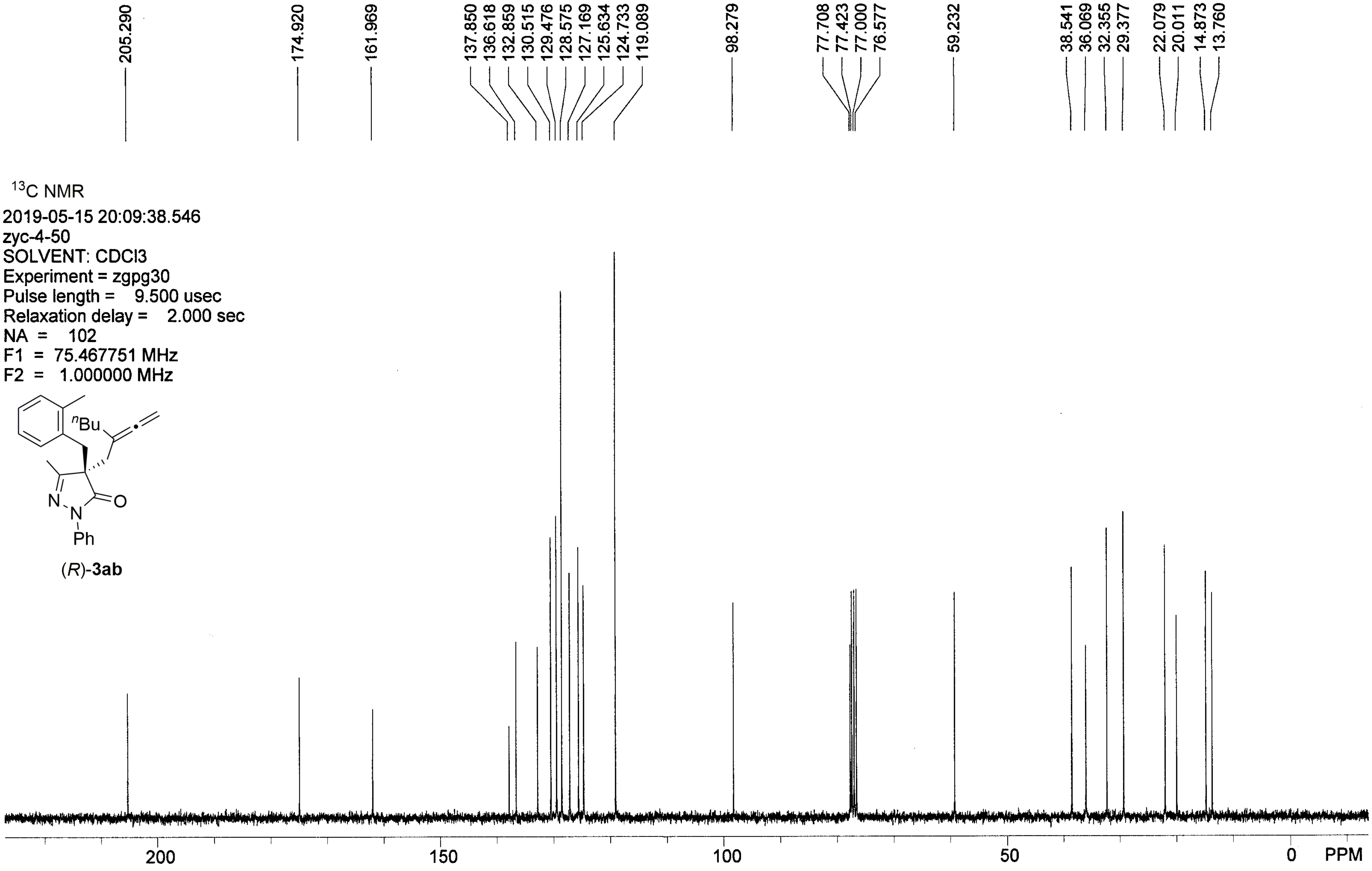

^{13}C NMR
2019-05-15 20:09:38.546
zyc-4-50
SOLVENT: CDCl3
Experiment = zgpg30
Pulse length = 9.500 usec
Relaxation delay = 2.000 sec
NA = 102
F1 = 75.467751 MHz
F2 = 1.000000 MHz

(*R*)-**3ab**

zyc-4-50

实验时间：2019-05-22,18:32:36
谱图文件:D:\浙大智达\N2000\样品\S20190522183236.org
方法文件:D:\浙大智达\N2000\djx.mtd

实验者：zyc
报告时间：2019-05-22,18:43:30
积分方法:面积归一法

实验内容简介：
ia,n-hexane/i-PrOH = 90/10,1.0,254

色谱图(S20190522183236.org)

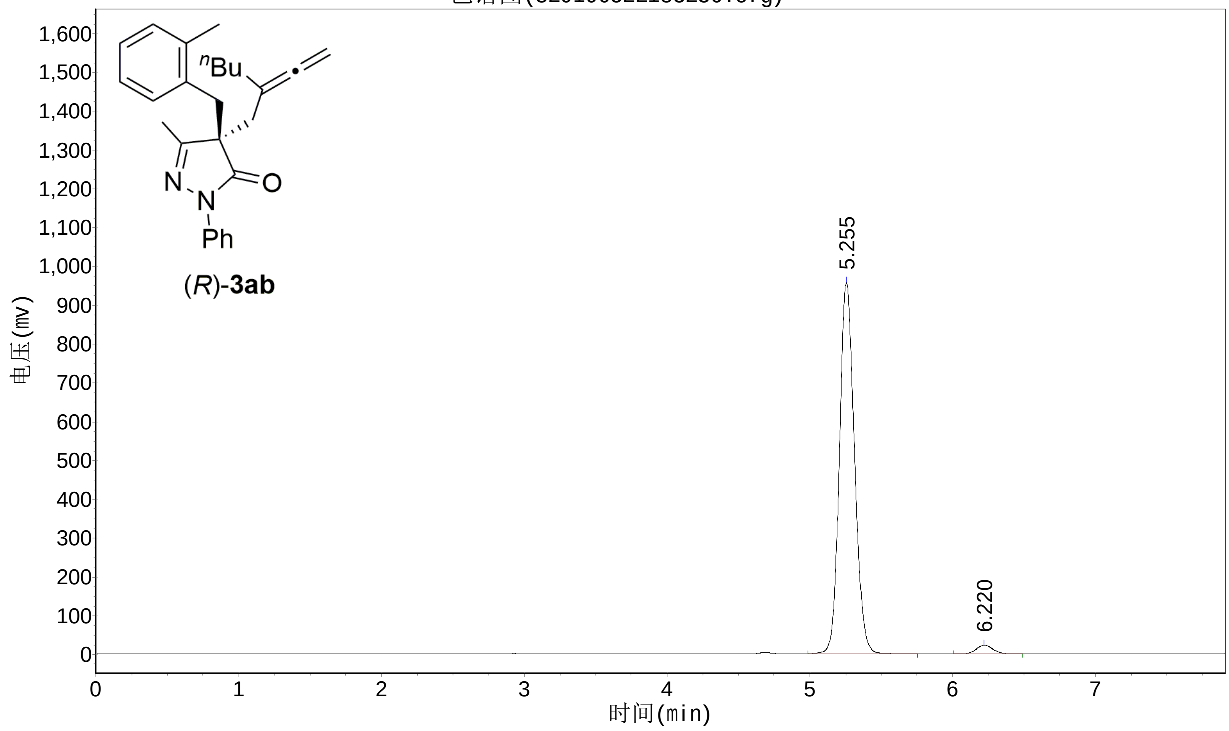

分析结果表

峰号	峰名	保留时间	峰高	峰面积	含量
1		5.255	957352.875	7035984.000	97.3783
2		6.220	22661.320	189426.438	2.6217
总计			980014.195	7225410.438	100.0000

zyc-4-50mix

实验时间：2019-05-22,18:58:09
谱图文件:D:\浙大智达\N2000\样品\S20190522185809.org
方法文件:D:\浙大智达\N2000\djx.mtd

实验者：zyc
报告时间：2019-05-22,19:07:39
积分方法:面积归一法

实验内容简介：
ia,n-hexane/i-PrOH = 90/10,1.0,254

色谱图(S20190522185809.org)

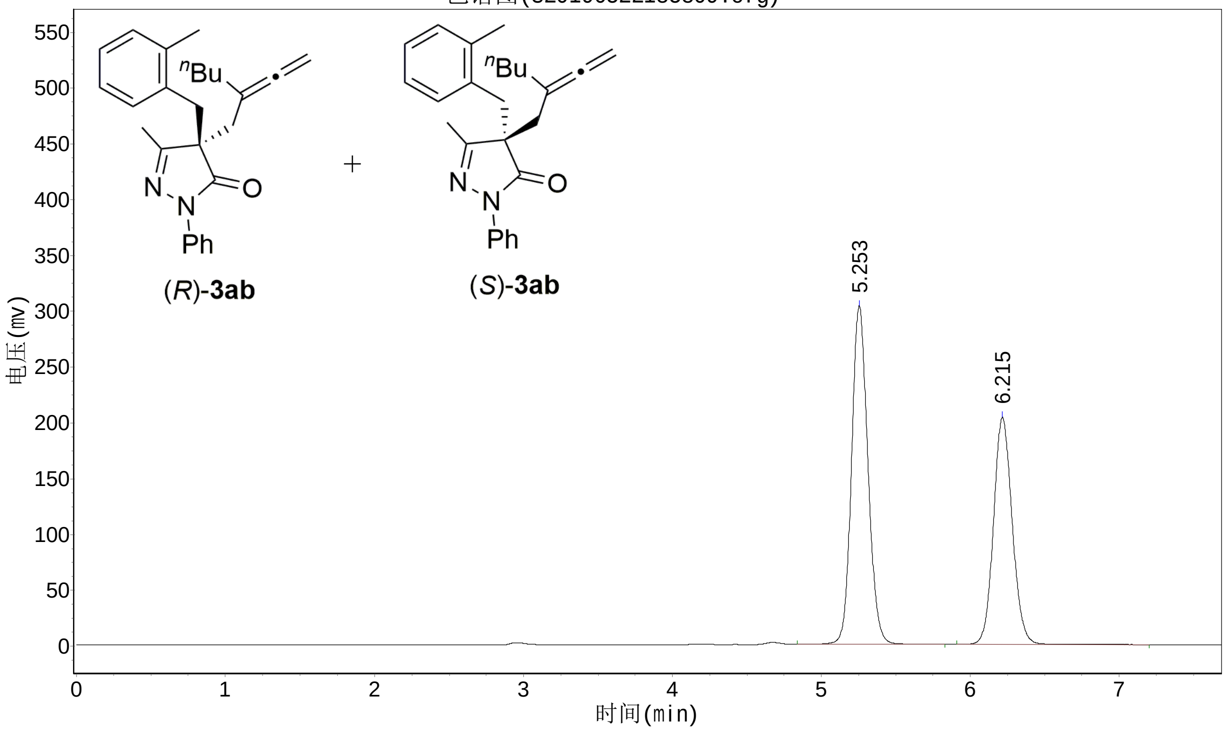

分析结果表

峰号	峰名	保留时间	峰高	峰面积	含量
1		5.253	303446.969	2321911.750	56.6181
2		6.215	203588.438	1779095.500	43.3819
总计			507035.406	4101007.250	100.0000

^{1}H NMR

2019-06-10 20:24:40.625
zyc-4-94
SOLVENT: CDCl3
Experiment = zg30
Pulse length = 14.000 usec
Relaxation delay = 1.000 sec
NA = 8
F1 = 300.130005 MHz
F2 = 1.000000 MHz

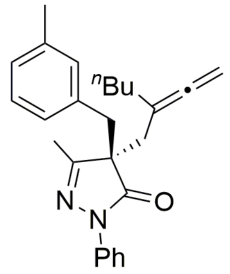

(*S*)-**3ac**

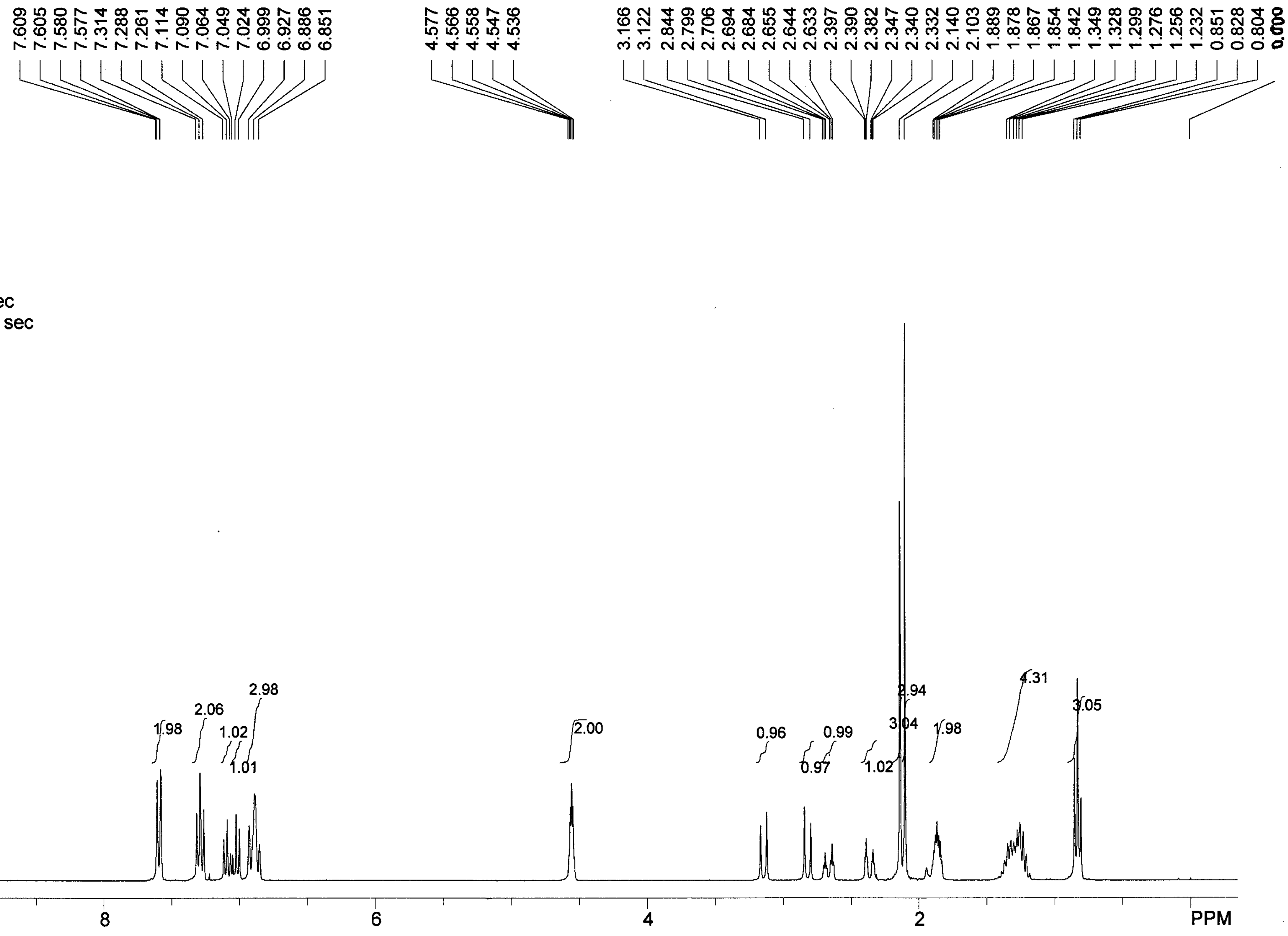

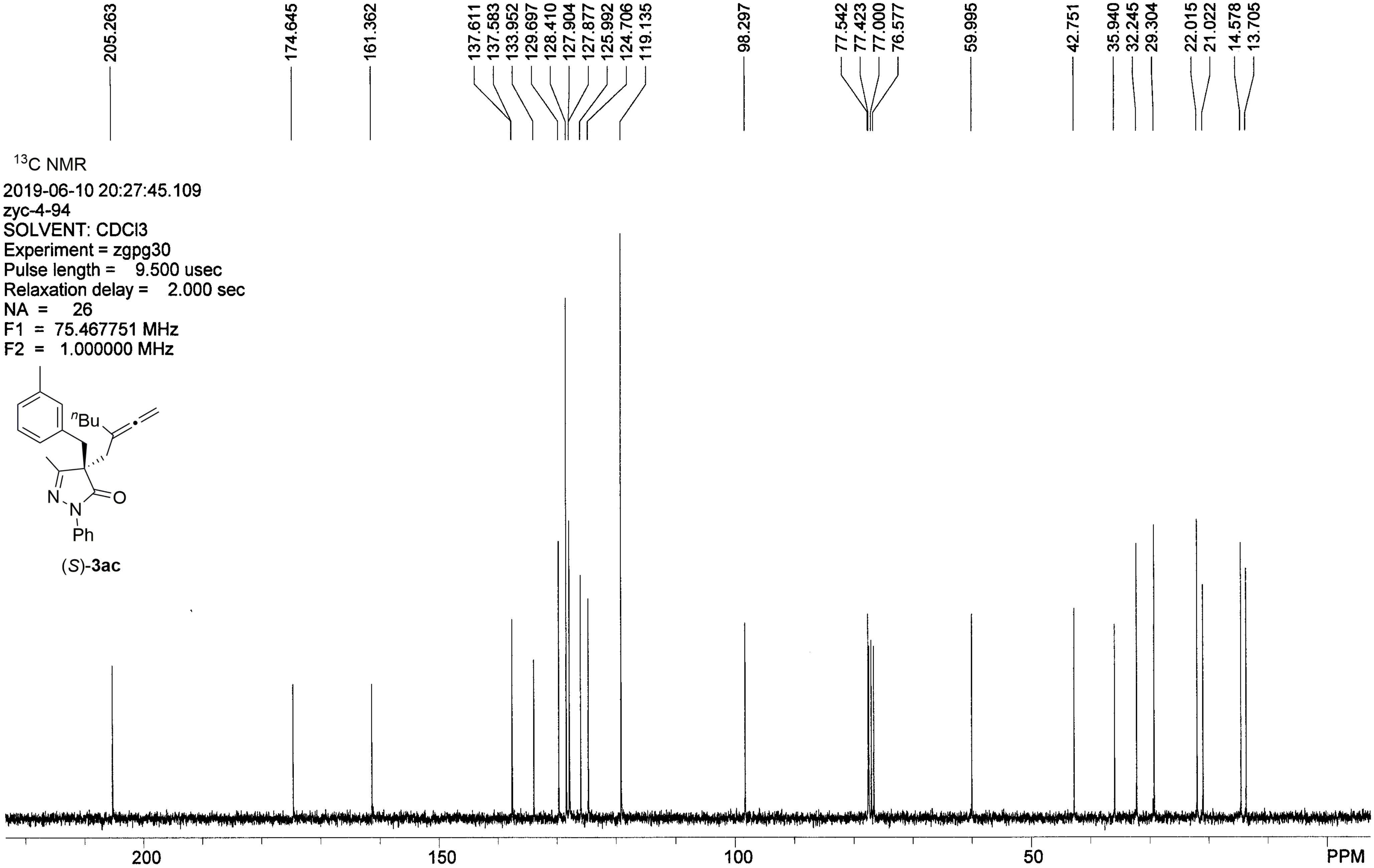
205.263
174.645
161.362
137.611
137.583
133.952
129.697
128.410
127.904
127.877
125.992
124.706
119.135
98.297
77.542
77.423
77.000
76.577
59.995
42.751
35.940
32.245
29.304
22.015
21.022
14.578
13.705
^{13}C NMR
2019-06-10 20:27:45.109
zyc-4-94
SOLVENT: CDCl3
Experiment = zgpg30
Pulse length = 9.500 usec
Relaxation delay = 2.000 sec
NA = 26
F1 = 75.467751 MHz
F2 = 1.000000 MHz
200
150
100
50
PPM
nBu
N
N
O
Ph

(*S*)-**3ac**

zyc-5-94

实验时间：2020-01-09,20:13:31
谱图文件:D:\浙大智达\N2000\样品\S20200109201331.org
方法文件:D:\浙大智达\N2000\djx.mtd

实验者：zyc
报告时间：2020-01-09,20:40:26
积分方法:面积归一法

实验内容简介：
ia,n-hexane/i-PrOH = 90/10,1.0,254

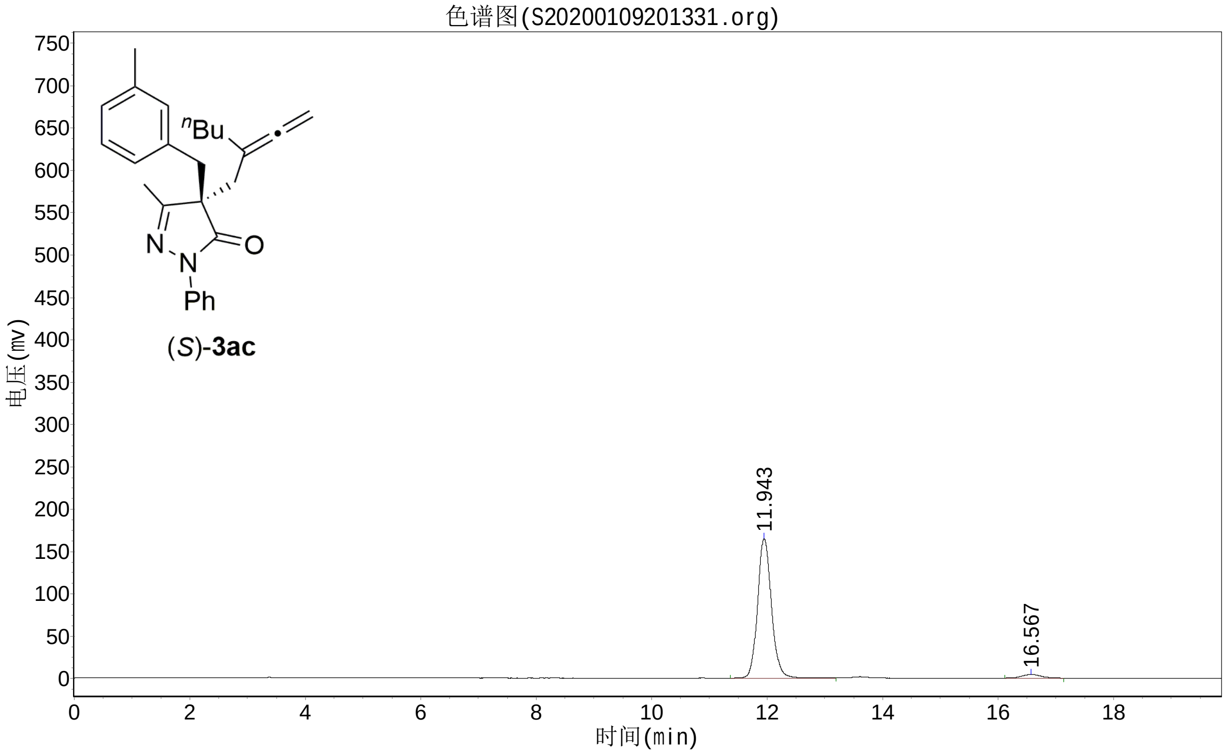

分析结果表

峰号	峰名	保留时间	峰高	峰面积	含量
1		11.943	164684.313	2747922.500	96.4767
2		16.567	4455.997	100352.211	3.5233
总计			169140.309	2848274.711	100.0000

zyc-5-94mix

实验时间：2020-01-09,19:46:03
谱图文件:D:\浙大智达\N2000\样品\S20200109194603.org
方法文件:D:\浙大智达\N2000\djx.mtd

实验者：zyc
报告时间：2020-01-09,20:15:16
积分方法:面积归一法

实验内容简介：
ia,n-hexane/i-PrOH = 90/10,1.0,254

色谱图(S20200109194603.org)

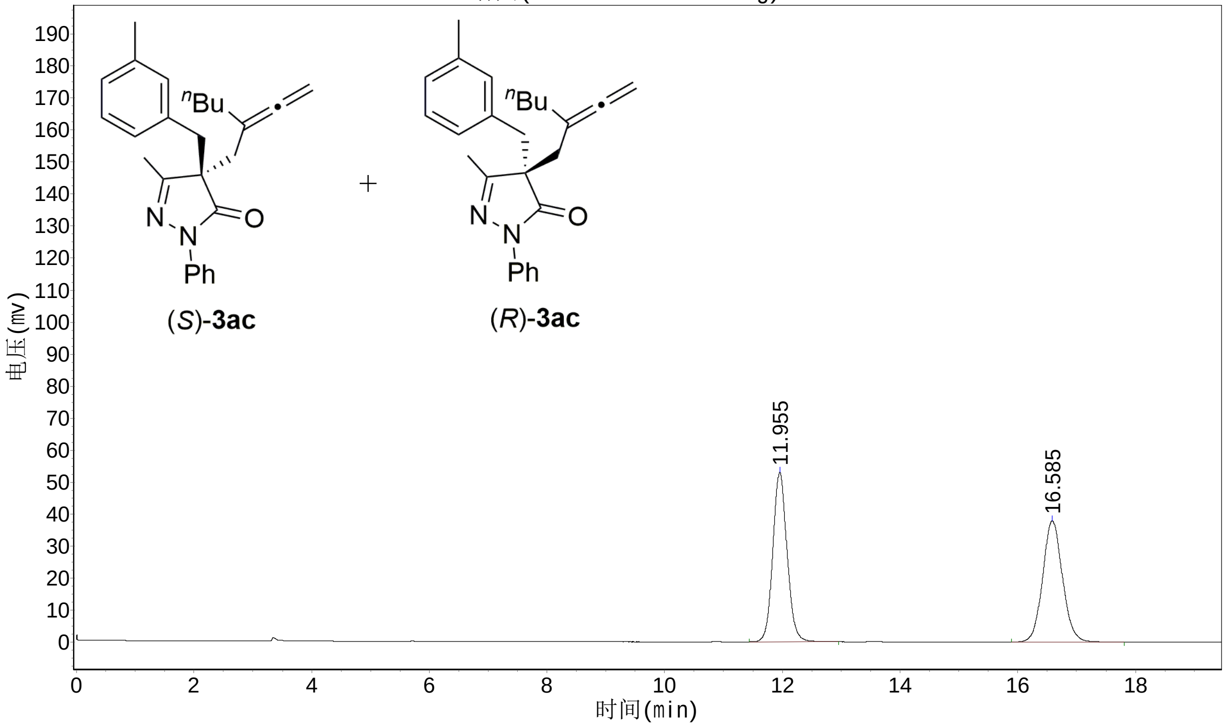

分析结果表

峰号	峰名	保留时间	峰高	峰面积	含量
1		11.955	53040.086	892794.813	50.0713
2		16.585	37888.281	890252.188	49.9287
总计			90928.367	1783047.000	100.0000

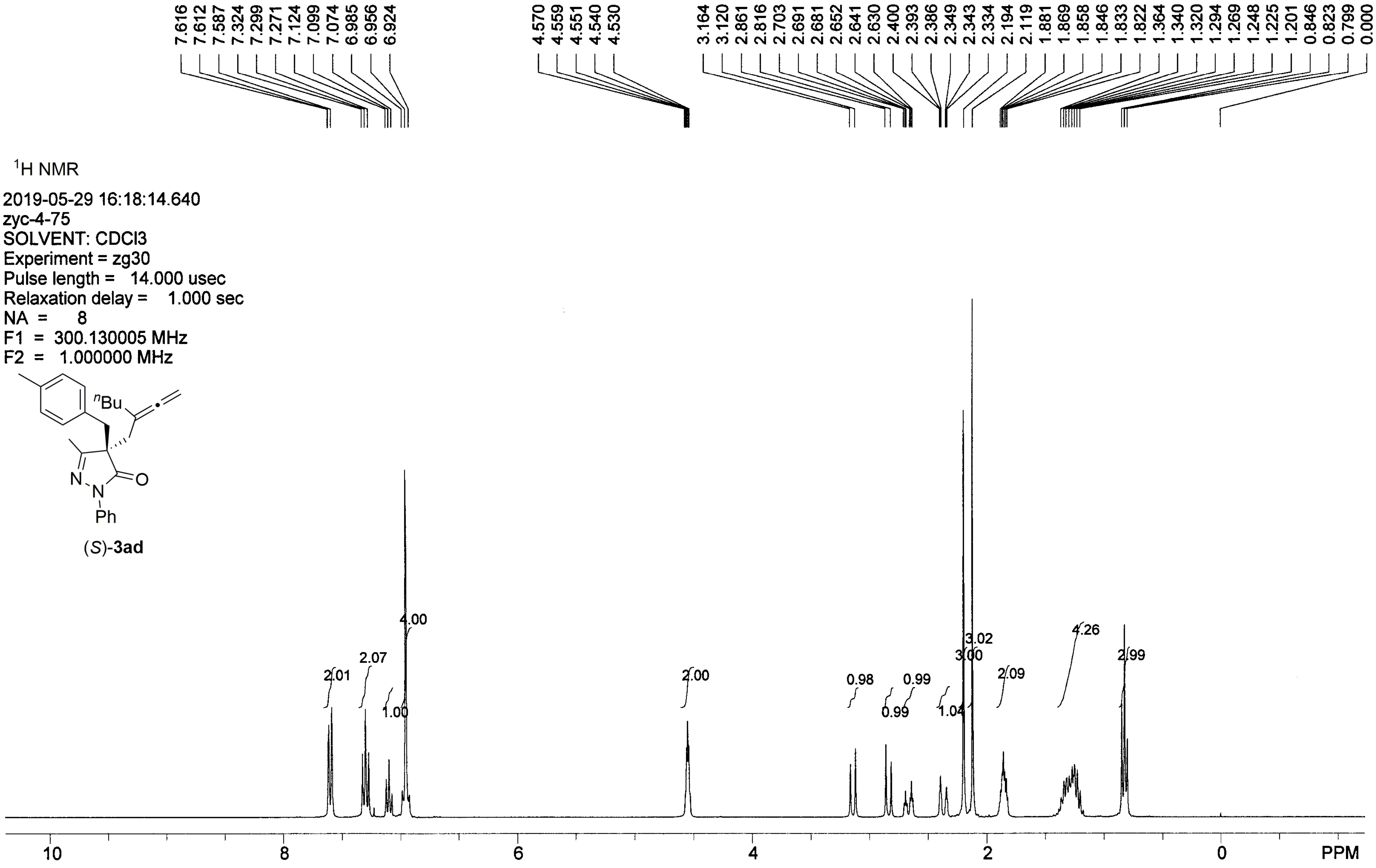

7.616 7.612 7.587 7.324 7.299 7.271 7.124 7.099 7.074 6.985 6.956 6.924
4.570 4.559 4.551 4.540 4.530
3.164 3.120 2.861 2.816 2.703 2.691 2.681 2.652 2.641 2.630 2.400 2.393 2.386 2.349 2.343 2.334 2.194 2.119 1.881 1.869 1.858 1.846 1.833 1.822 1.364 1.340 1.320 1.294 1.269 1.248 1.225 1.201 0.846 0.823 0.799 0.000
1H NMR
2019-05-29 16:18:14.640
zyc-4-75
SOLVENT: CDCl3
Experiment = zg30
Pulse length = 14.000 usec
Relaxation delay = 1.000 sec
NA = 8
F1 = 300.130005 MHz
F2 = 1.000000 MHz
nBu
N
N
O
Ph
(S)-3ad
2.01
2.07
1.00
4.00
2.00
0.98
0.99
0.99
1.04
3.00
3.02
2.09
4.26
2.99
10
8
6
4
2
0
PPM

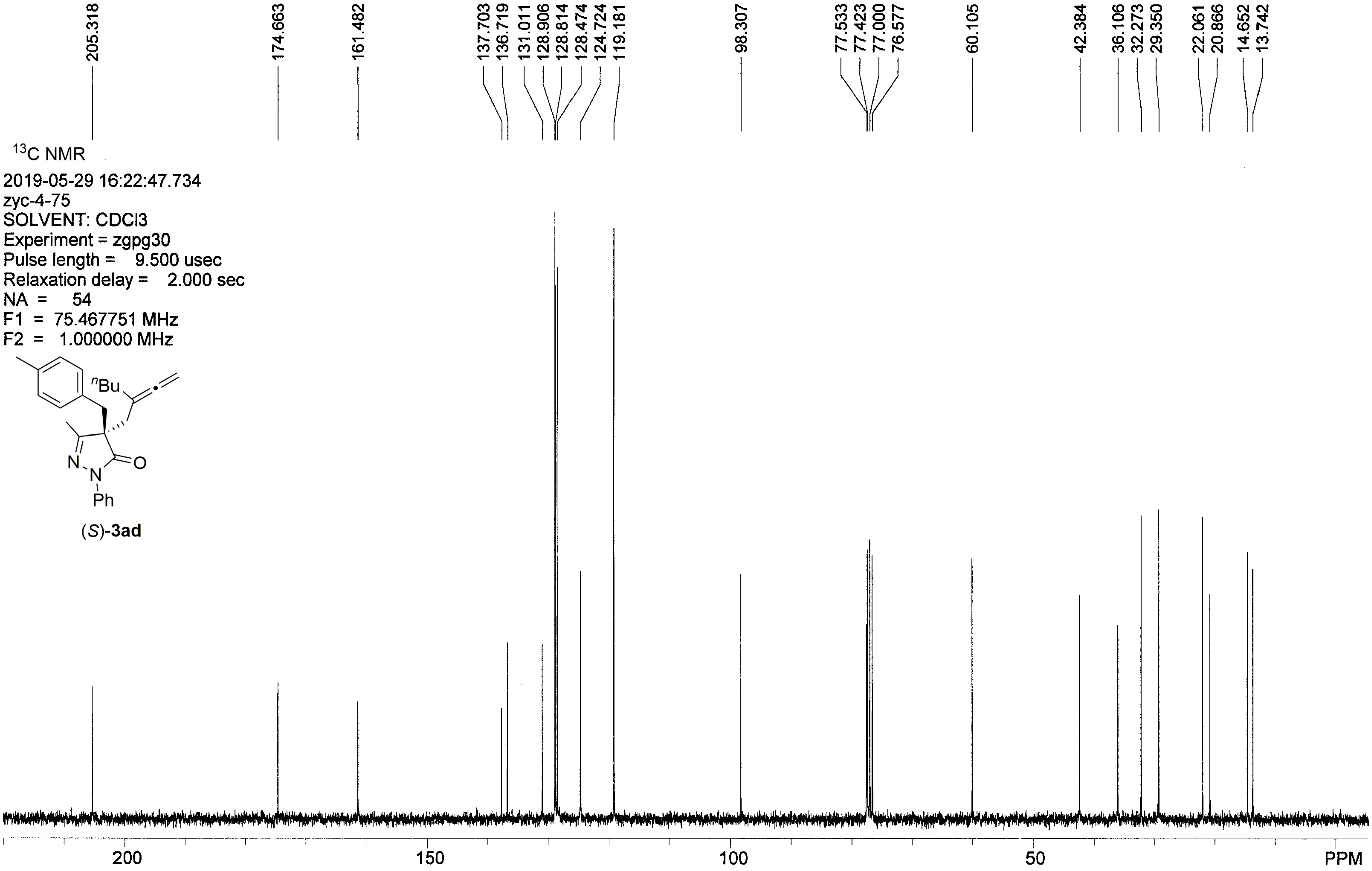

^{13}C NMR

2019-05-29 16:22:47.734
zyc-4-75
SOLVENT: CDCl3
Experiment = zgpg30
Pulse length = 9.500 usec
Relaxation delay = 2.000 sec
NA = 54
F1 = 75.467751 MHz
F2 = 1.000000 MHz

(*S*)-**3ad**

zyc-4-75

实验时间：2019-05-30,14:45:31
谱图文件:D:\浙大智达\N2000\样品\S20190530144531.org
方法文件:D:\浙大智达\N2000\djx.mtd

实验者：zyc
报告时间：2019-05-30,15:00:02
积分方法:面积归一法

实验内容简介：
ia,n-hexane/i-PrOH = 90/10,1.0,254

色谱图(S20190530144531.org)

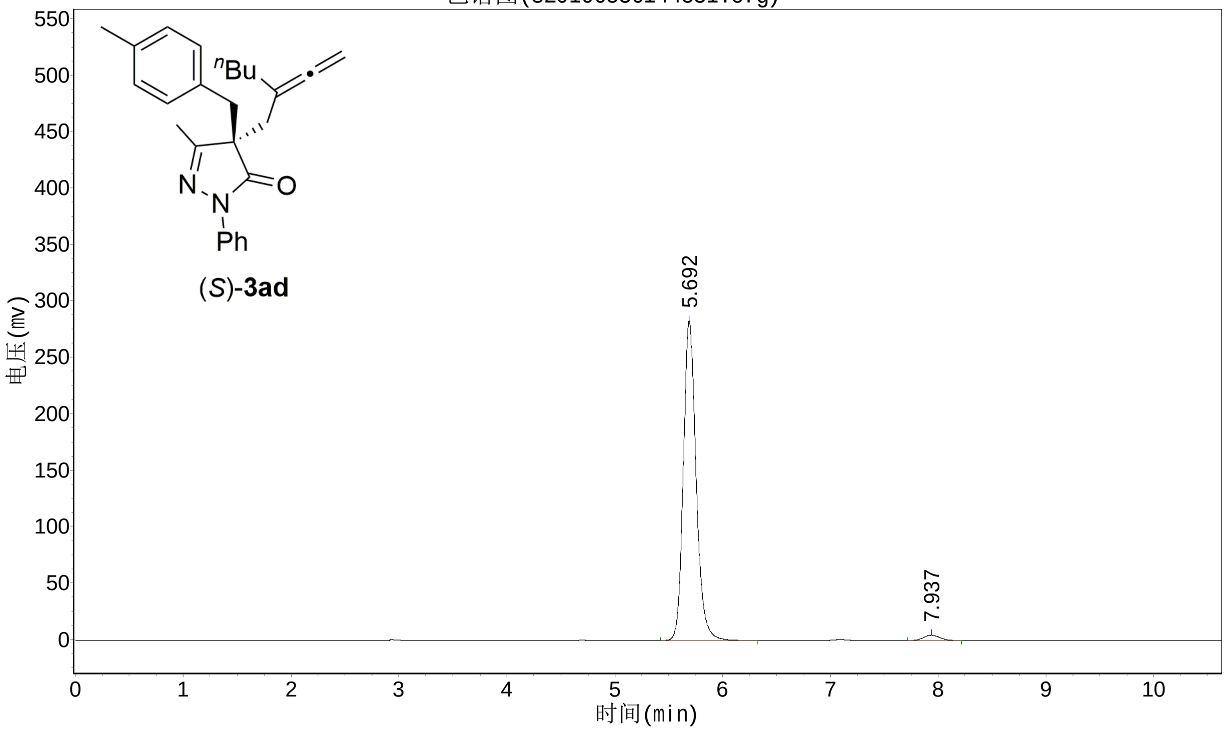

分析结果表

峰号	峰名	保留时间	峰高	峰面积	含量
1		5.692	283639.781	2351272.000	97.7116
2		7.937	5044.901	55067.602	2.2884
总计			288684.682	2406339.602	100.0000

zyc-4-75mix

实验时间：2019-05-30,14:31:43
谱图文件:D:\浙大智达\N2000\样品\S20190530143143.org
方法文件:D:\浙大智达\N2000\djx.mtd

实验者：zyc
报告时间：2019-05-30,14:47:18
积分方法:面积归一法

实验内容简介：
ia,n-hexane/i-PrOH = 90/10,1.0,254

色谱图(S20190530143143.org)

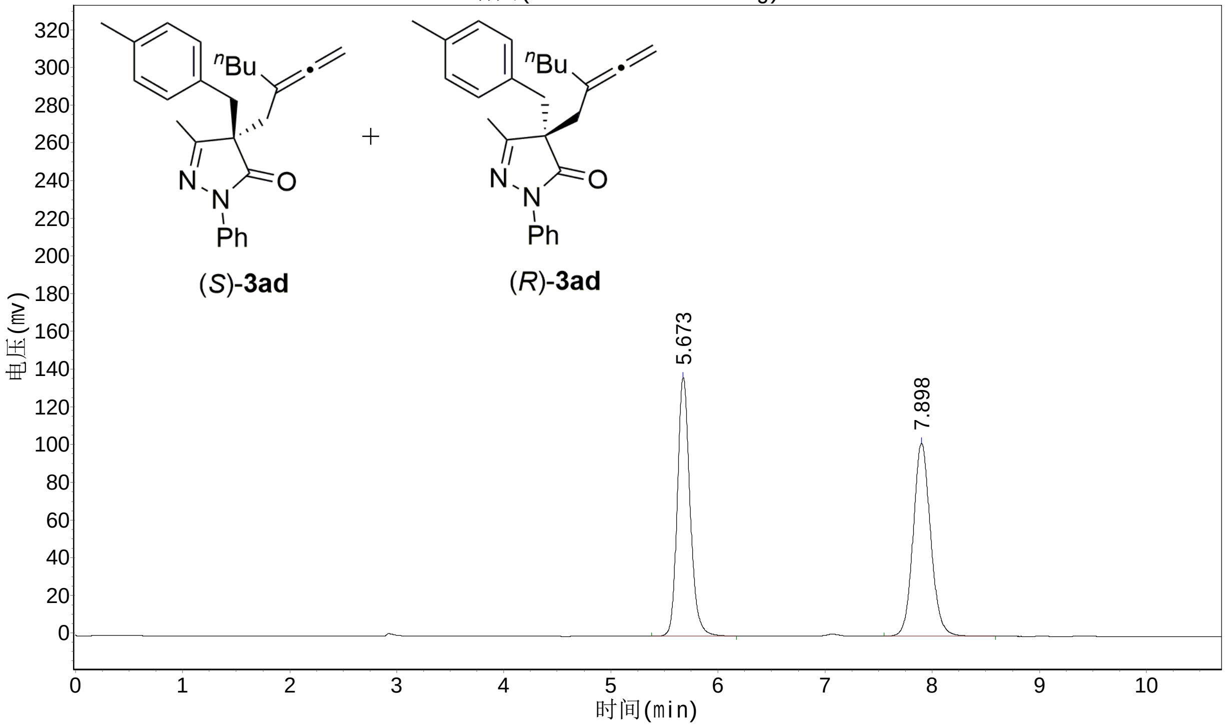

分析结果表

峰号	峰名	保留时间	峰高	峰面积	含量
1		5.673	137267.922	1134848.000	49.4143
2		7.898	102486.336	1161749.000	50.5857
总计			239754.258	2296597.000	100.0000

^{1}H NMR

2019-05-29 21:01:26.609
zyc-4-76
SOLVENT: CDCl3
Experiment = zg30
Pulse length = 14.000 usec
Relaxation delay = 1.000 sec
NA = 8
F1 = 300.130005 MHz
F2 = 1.000000 MHz

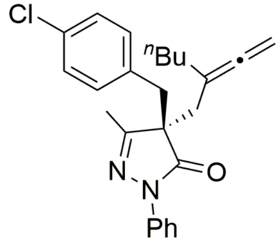

(*S*)-**3ae**

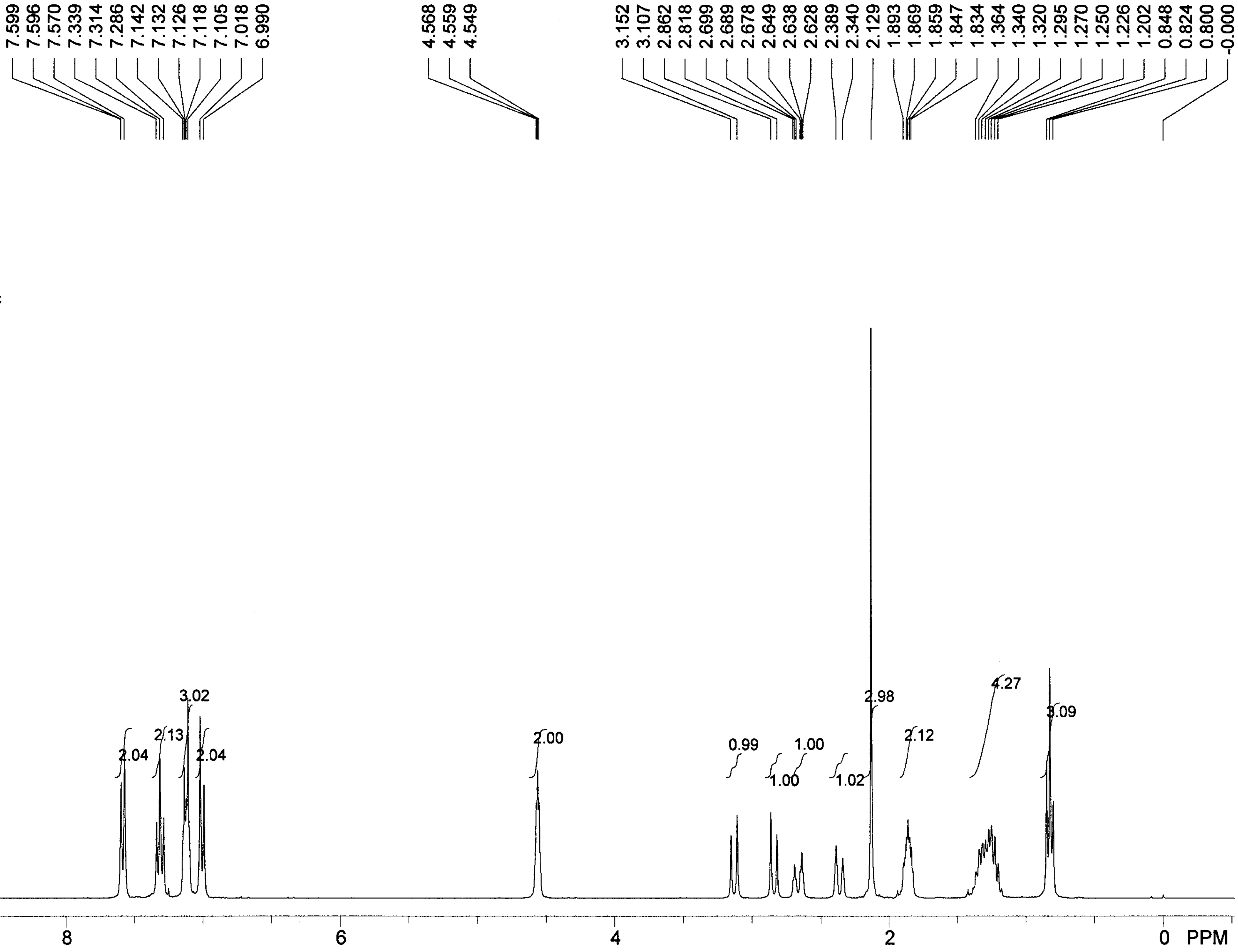

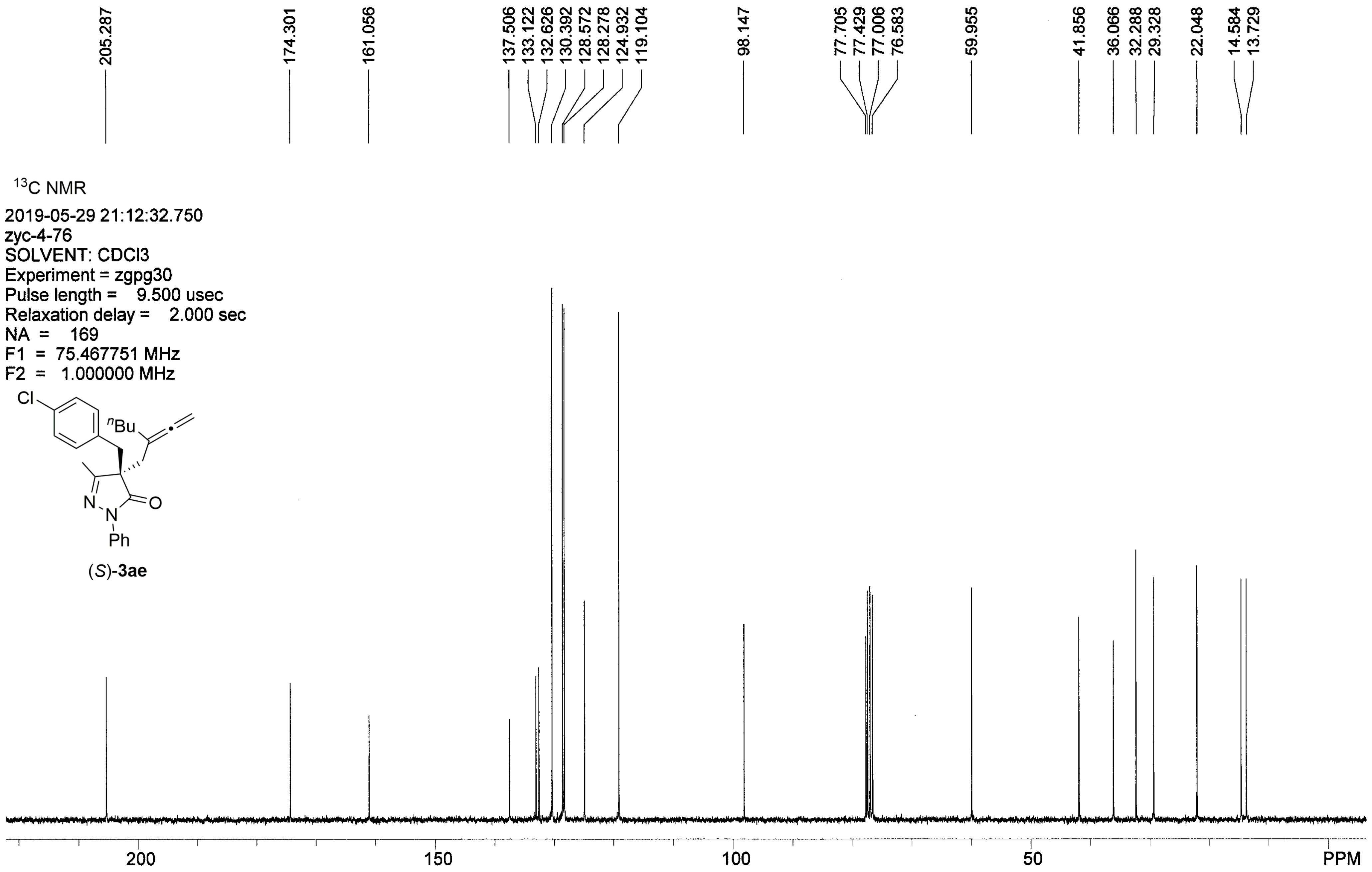
205.287
174.301
161.056
137.506
133.122
132.626
130.392
128.572
128.278
124.932
119.104
98.147
77.705
77.429
77.006
76.583
59.955
41.856
36.066
32.288
29.328
22.048
14.584
13.729
13C NMR
2019-05-29 21:12:32.750
zyc-4-76
SOLVENT: CDCl3
Experiment = zgpg30
Pulse length = 9.500 usec
Relaxation delay = 2.000 sec
NA = 169
F1 = 75.467751 MHz
F2 = 1.000000 MHz
Cl
nBu
N
N
O
Ph
(S)-3ae
200
150
100
50
PPM

zyc-4-76

实验时间：2019-05-30,15:24:05
谱图文件:D:\浙大智达\N2000\样品\S20190530152405.org
方法文件:D:\浙大智达\N2000\djx.mtd

实验者：zyc
报告时间：2019-05-30,15:38:21
积分方法:面积归一法

实验内容简介：
ia,n-hexane/i-PrOH = 90/10,1.0,254

色谱图(S20190530152405.org)

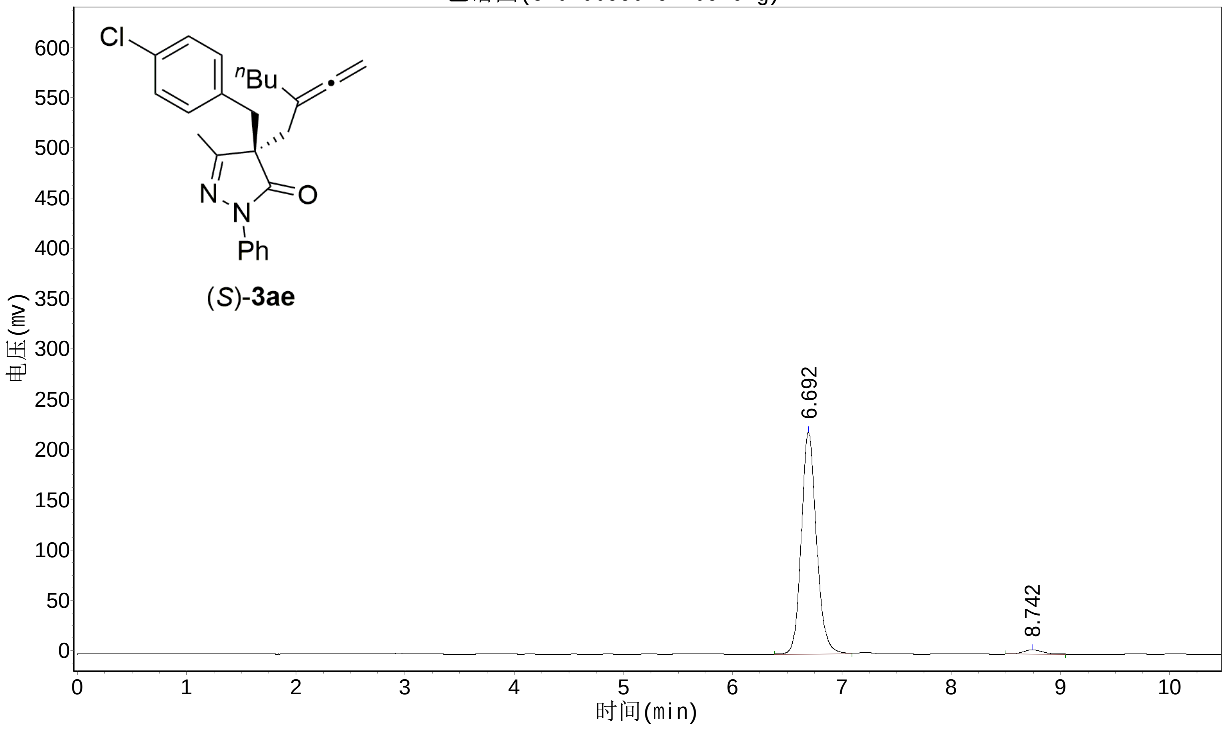

分析结果表

峰号	峰名	保留时间	峰高	峰面积	含量
1		6.692	220636.453	2139591.500	97.5744
2		8.742	4395.492	53188.254	2.4256
总计			225031.945	2192779.754	100.0000

zyc-4-76mix

实验时间：2019-05-30,15:40:50
谱图文件:D:\浙大智达\N2000\样品\S20190530154050.org
方法文件:D:\浙大智达\N2000\djx.mtd

实验者：zyc
报告时间：2019-05-30,15:59:34
积分方法:面积归一法

实验内容简介：
ia,n-hexane/i-PrOH = 90/10,1.0,254

色谱图(S20190530154050.org)

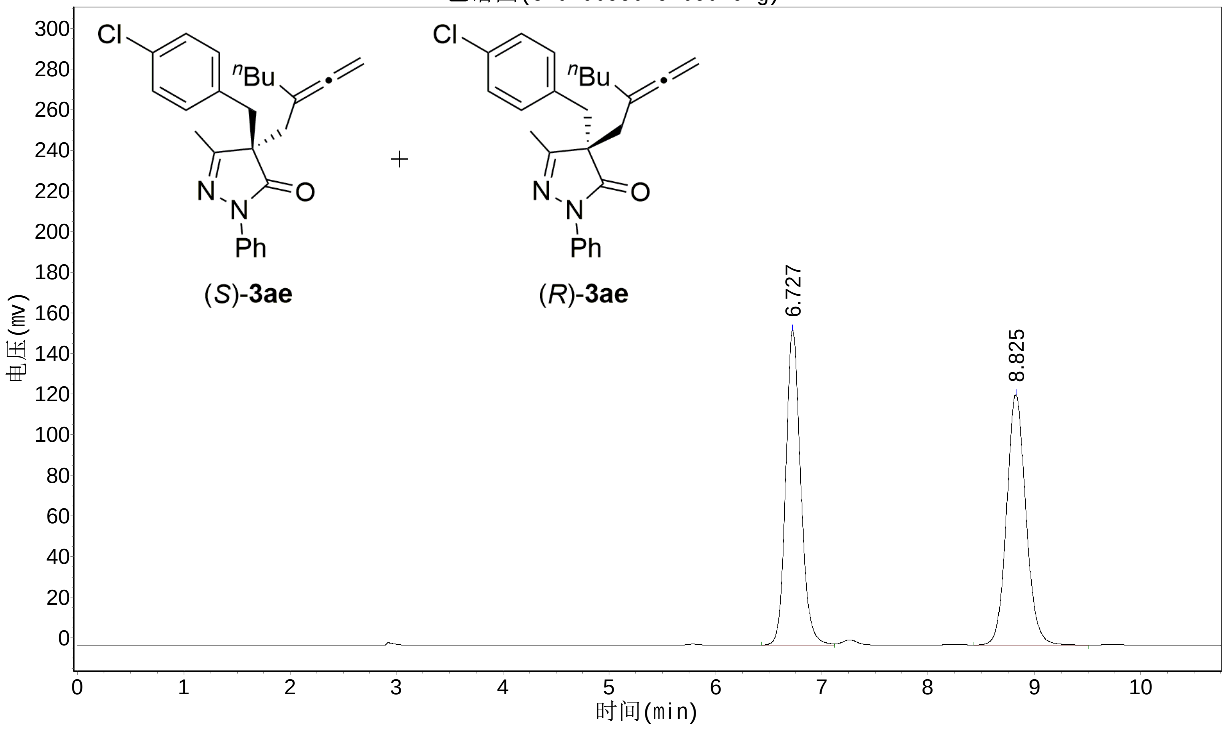

分析结果表

峰号	峰名	保留时间	峰高	峰面积	含量
1		6.727	155037.344	1515561.250	49.3564
2		8.825	123378.414	1555083.625	50.6436
总计			278415.758	3070644.875	100.0000

^{1}H NMR
2019-06-12 21:47:59.812
zyc-4-97
SOLVENT: CDCl3
Experiment = zg30
Pulse length = 14.000 usec
Relaxation delay = 1.000 sec
NA = 8
F1 = 300.130005 MHz
F2 = 1.000000 MHz

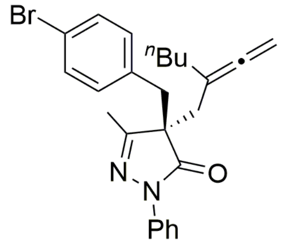

(S)-**3af**

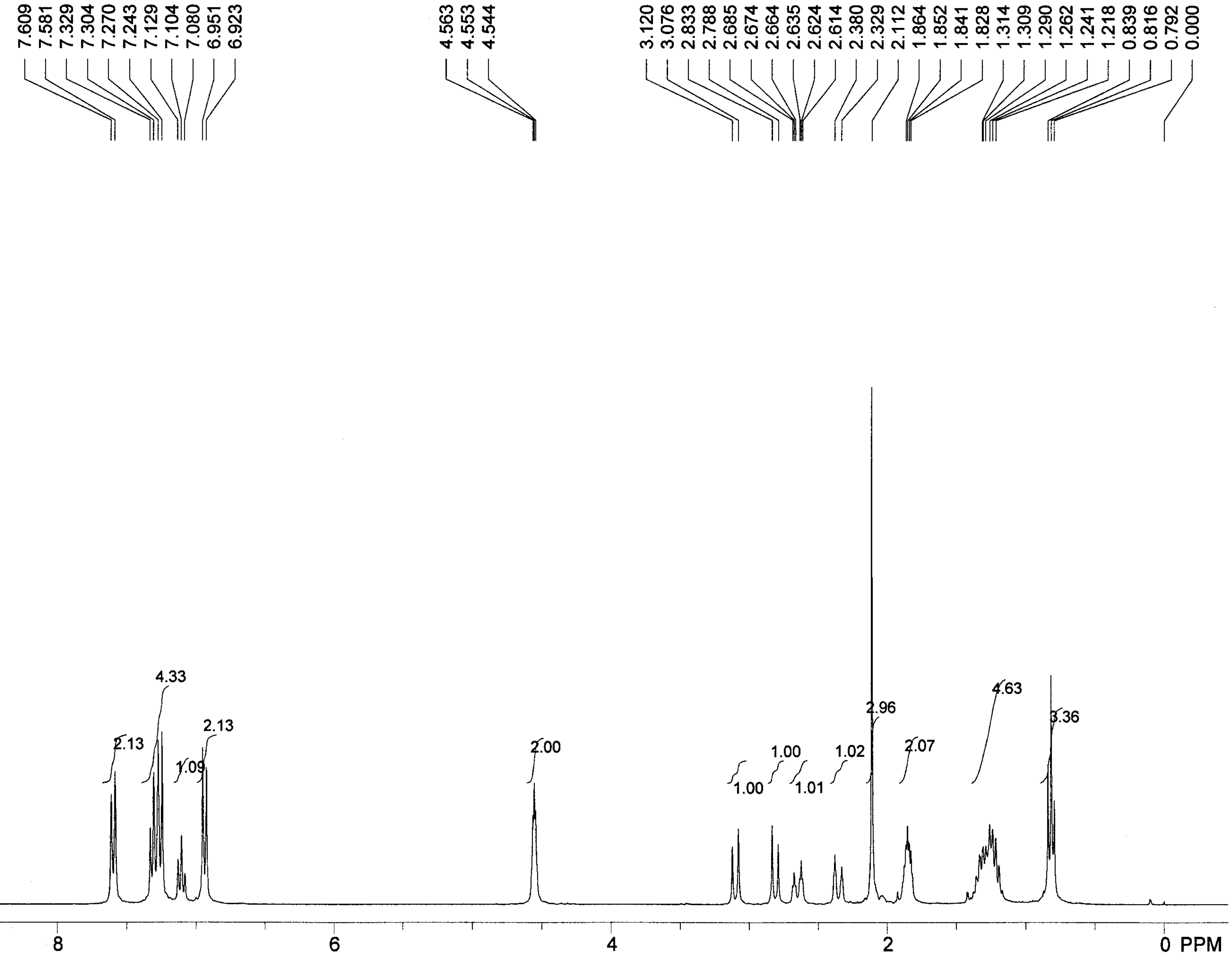

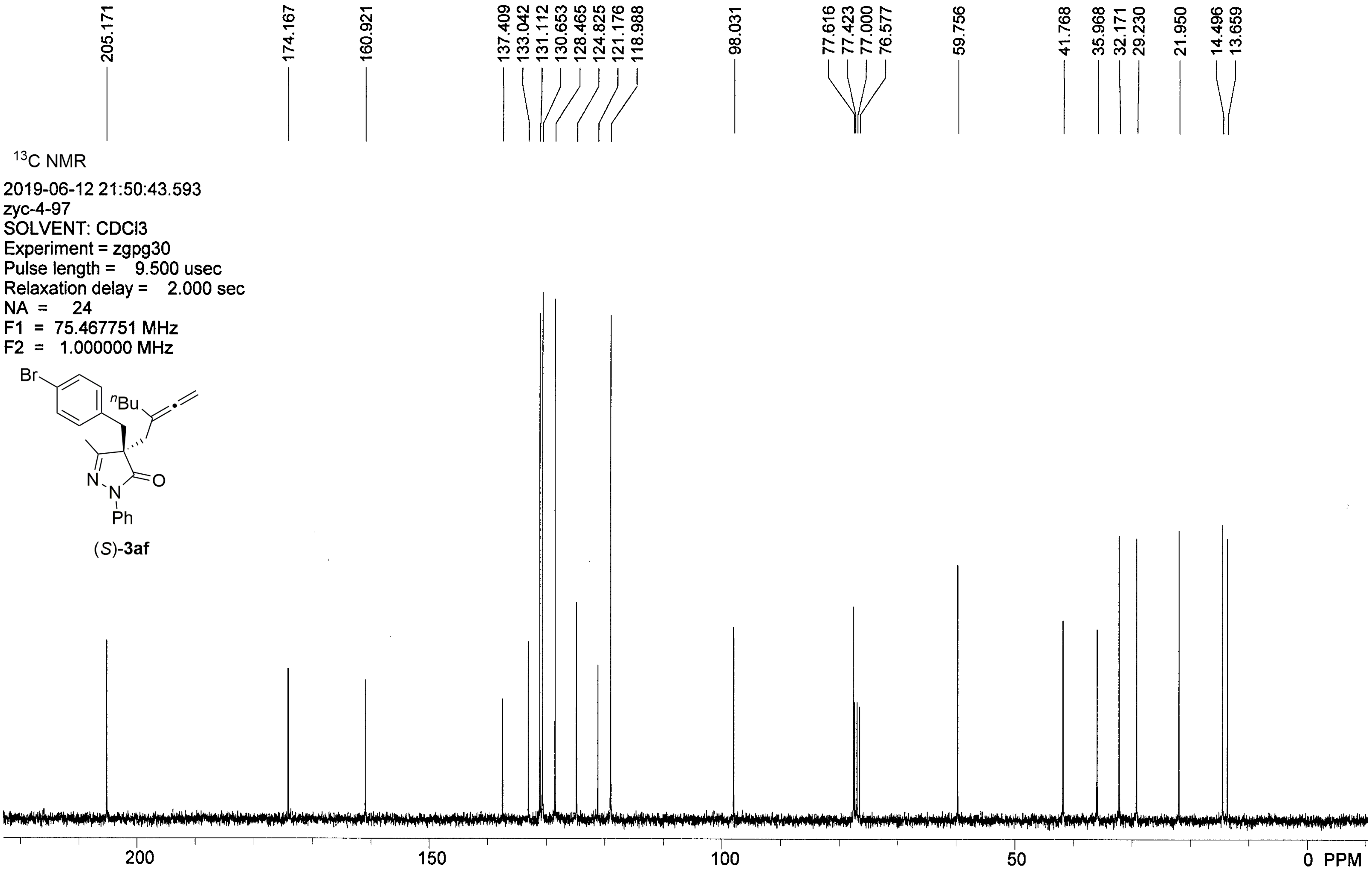

^{13}C NMR

2019-06-12 21:50:43.593
zyc-4-97
SOLVENT: CDCl3
Experiment = zgpg30
Pulse length = 9.500 usec
Relaxation delay = 2.000 sec
NA = 24
F1 = 75.467751 MHz
F2 = 1.000000 MHz

(*S*)-**3af**

zyc-4-97

实验时间：2019-06-13,12:04:25　　　　实验者：zyc
谱图文件:D:\浙大智达\N2000\样品\S20190613120425.org　　　　报告时间：2019-06-13,12:20:51
方法文件:D:\浙大智达\N2000\djx.mtd　　　　积分方法:面积归一法

实验内容简介：
ia,n-hexane/i-PrOH = 90/10,1.0,254

色谱图(S20190613120425.org)

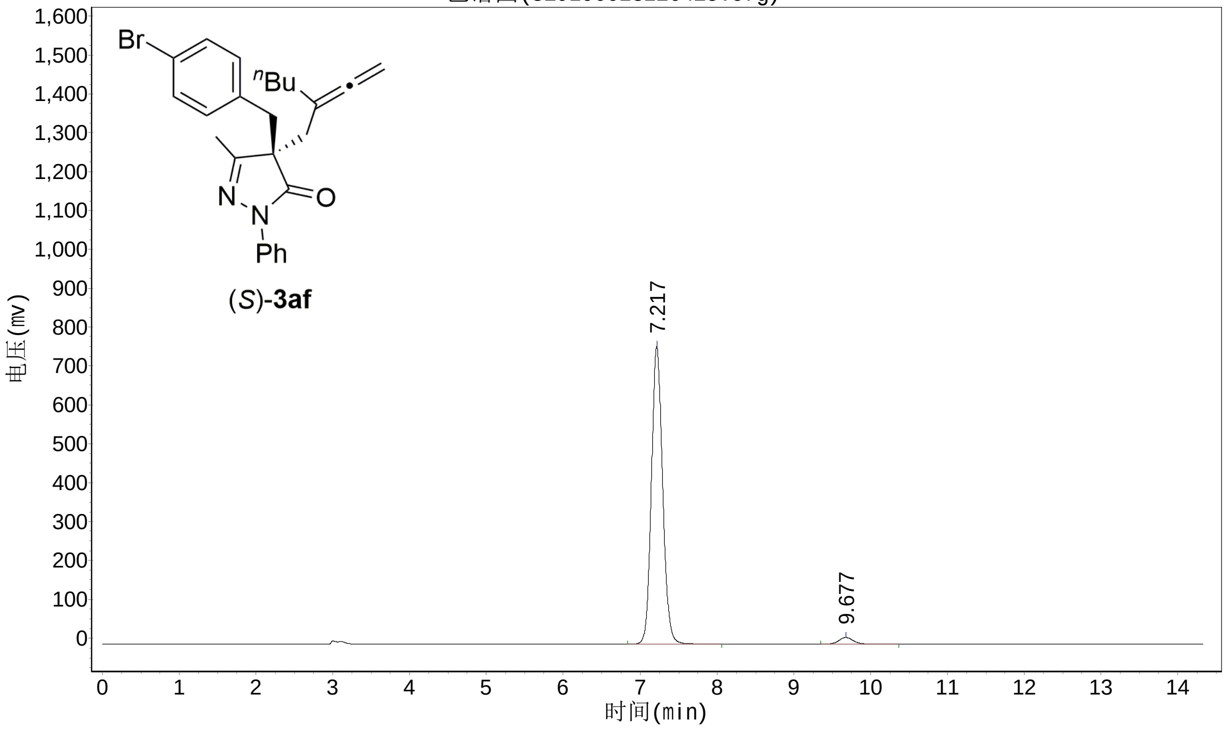

分析结果表

峰号	峰名	保留时间	峰高	峰面积	含量
1		7.217	766741.625	7795622.500	97.0520
2		9.677	17205.238	236797.734	2.9480
总计			783946.863	8032420.234	100.0000

zyc-4-97mix

实验时间：2019-06-13,11:43:19
谱图文件:D:\浙大智达\N2000\样品\S20190613114319.org
方法文件:D:\浙大智达\N2000\djx.mtd

实验者：zyc
报告时间：2019-06-13,11:59:00
积分方法:面积归一法

实验内容简介：
ia,n-hexane/i-PrOH = 90/10,1.0,254

色谱图(S20190613114319.org)

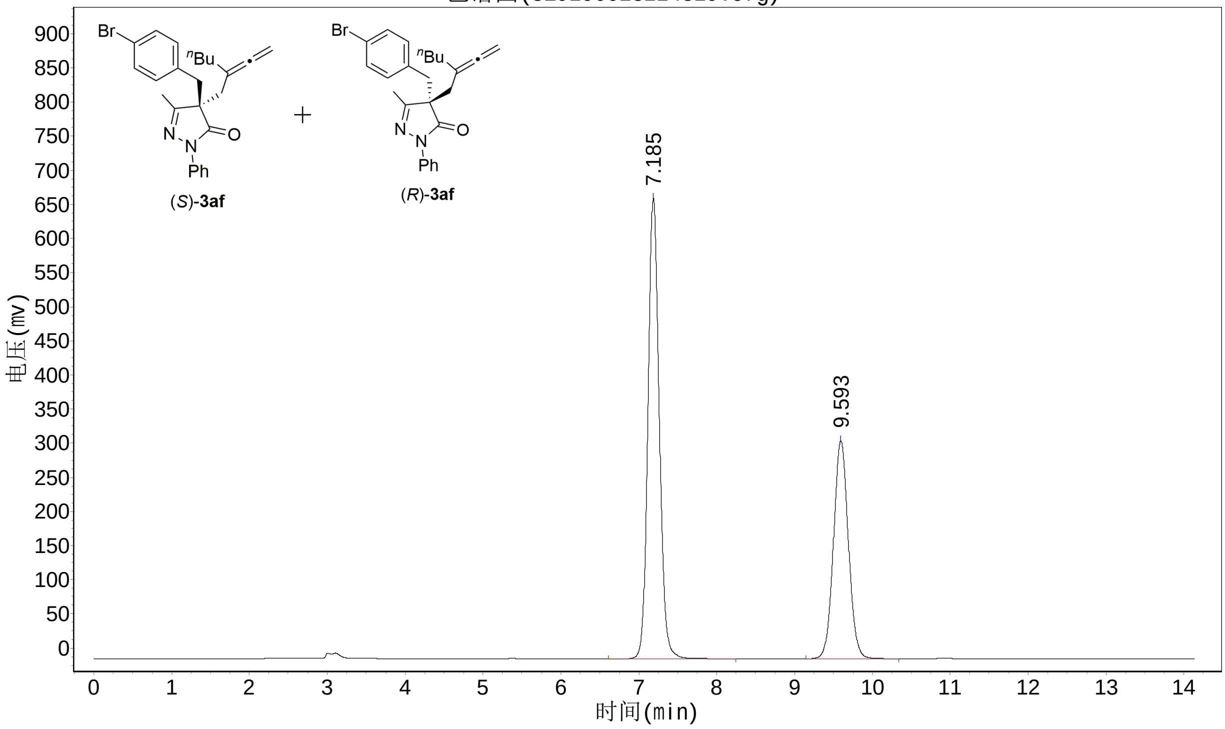

分析结果表

峰号	峰名	保留时间	峰高	峰面积	含量
1		7.185	674470.500	6795611.000	61.5631
2		9.593	318987.875	4242829.000	38.4369
总计			993458.375	11038440.000	100.0000

^{1}H NMR
2019-07-11 12:19:18.703
zyc-4-105
SOLVENT: CDCl3
Experiment = zg30
Pulse length = 14.000 usec
Relaxation delay = 1.000 sec
NA = 8
F1 = 300.130005 MHz
F2 = 1.000000 MHz

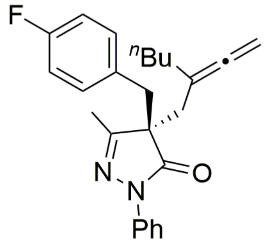

(*S*)-**3ag**

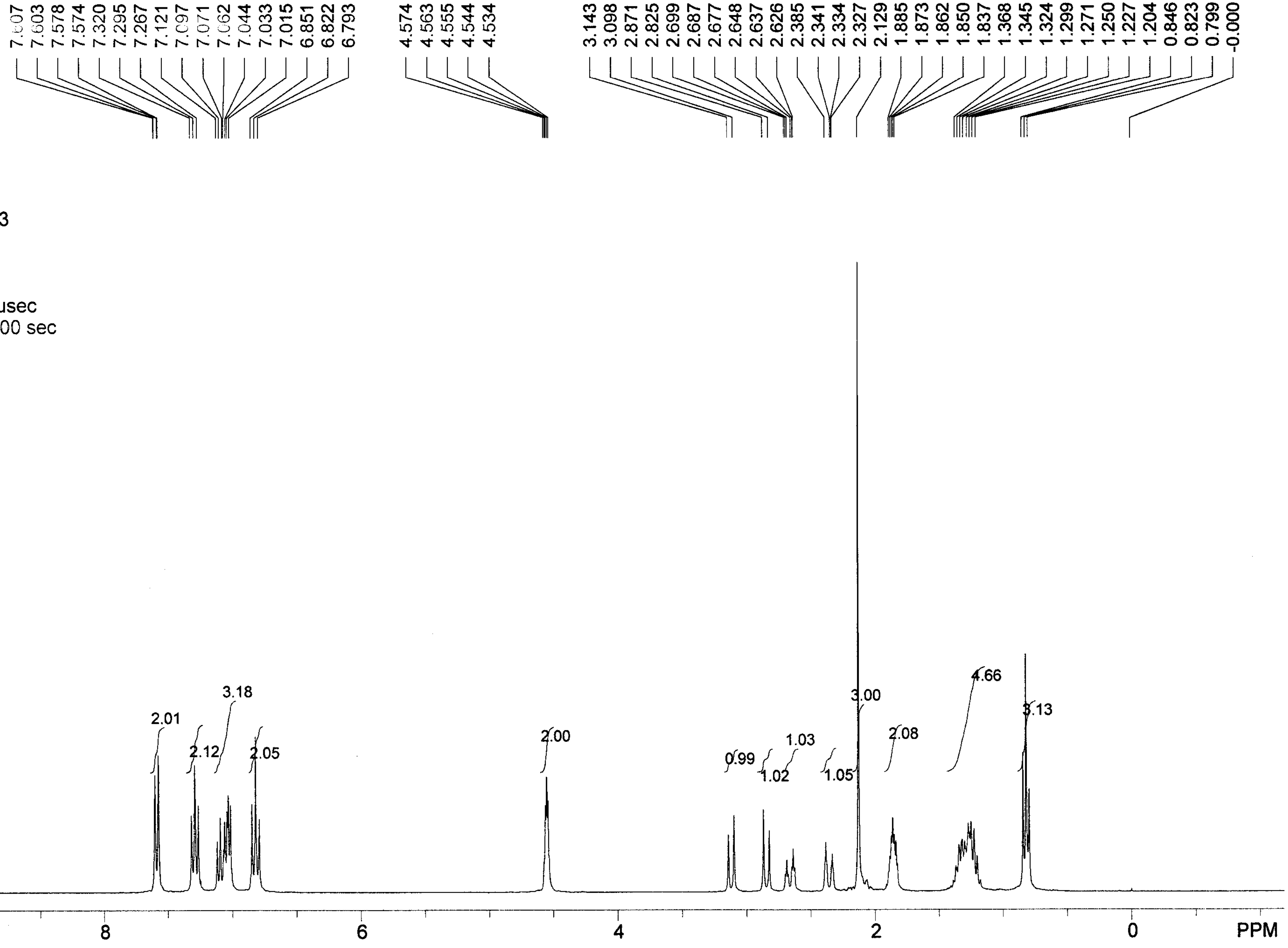

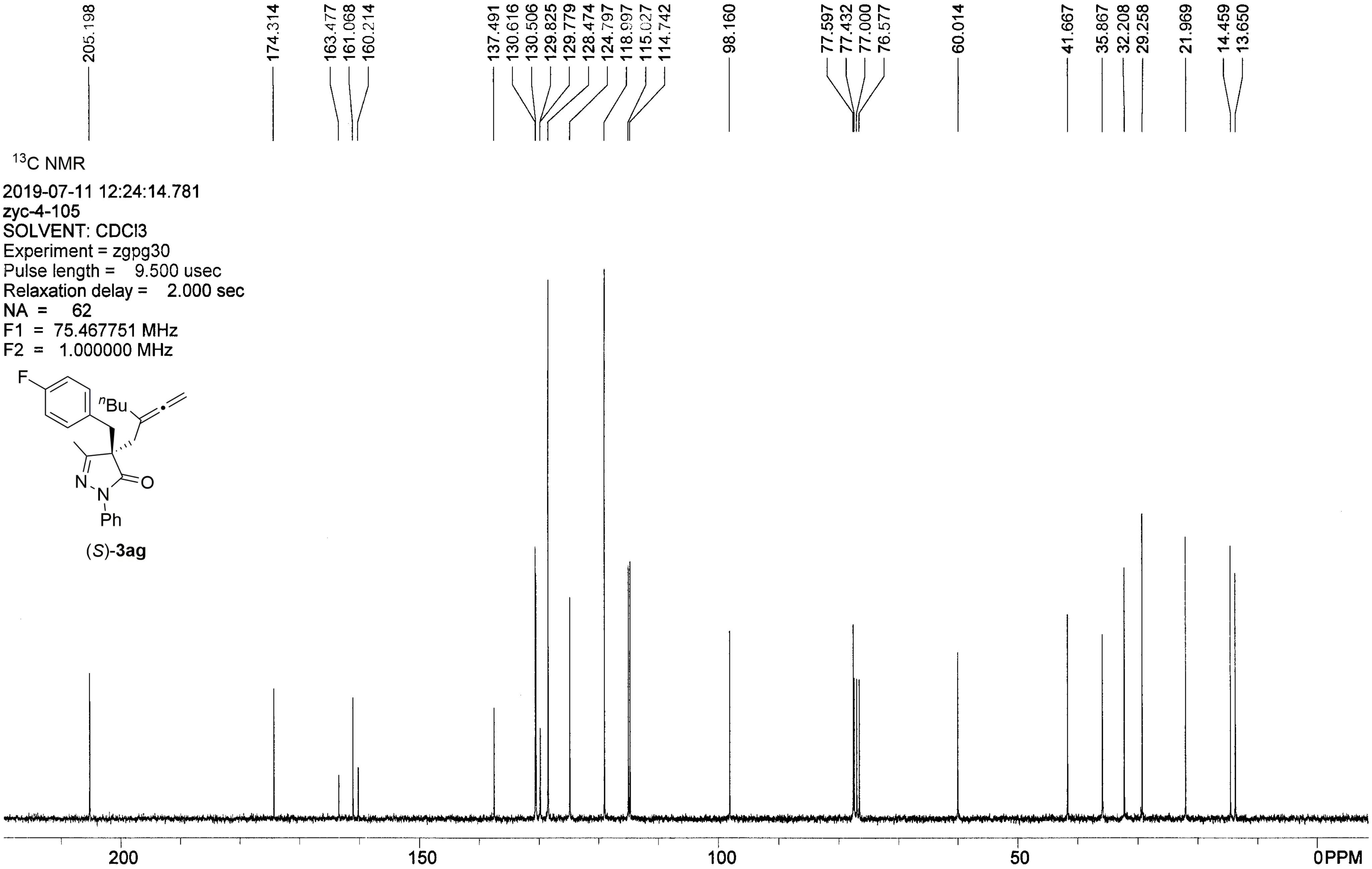

^{13}C NMR

2019-07-11 12:24:14.781
zyc-4-105
SOLVENT: CDCl3
Experiment = zgpg30
Pulse length = 9.500 usec
Relaxation delay = 2.000 sec
NA = 62
F1 = 75.467751 MHz
F2 = 1.000000 MHz

(*S*)-**3ag**

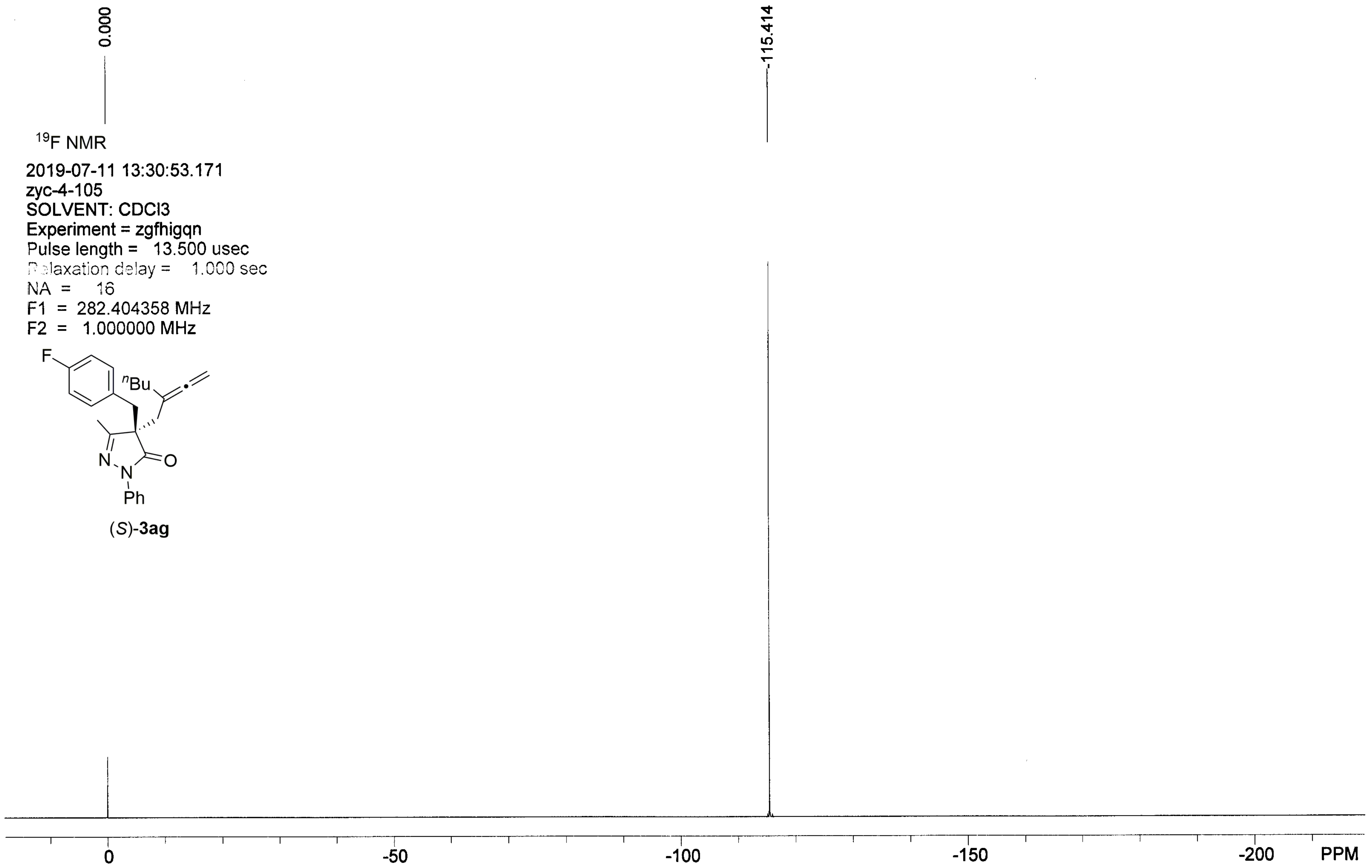
0.000
-115.414
^{19}F NMR
2019-07-11 13:30:53.171
zyc-4-105
SOLVENT: CDCl3
Experiment = zgfhigqn
Pulse length = 13.500 usec
Relaxation delay = 1.000 sec
NA = 16
F1 = 282.404358 MHz
F2 = 1.000000 MHz
F
nBu
O
N
N
Ph
0
-50
-100
-150
-200
PPM
(S)-**3ag**

zyc-4-105

实验时间：2019-07-11,11:01:47
谱图文件:D:\浙大智达\N2000\样品\S20190711110147.org
方法文件:D:\浙大智达\N2000\djx.mtd

实验者：zyc
报告时间：2019-07-11,11:16:14
积分方法:面积归一法

实验内容简介：
ia,n-hexane/i-PrOH = 90/10,1.0,254

色谱图(S20190711110147.org)

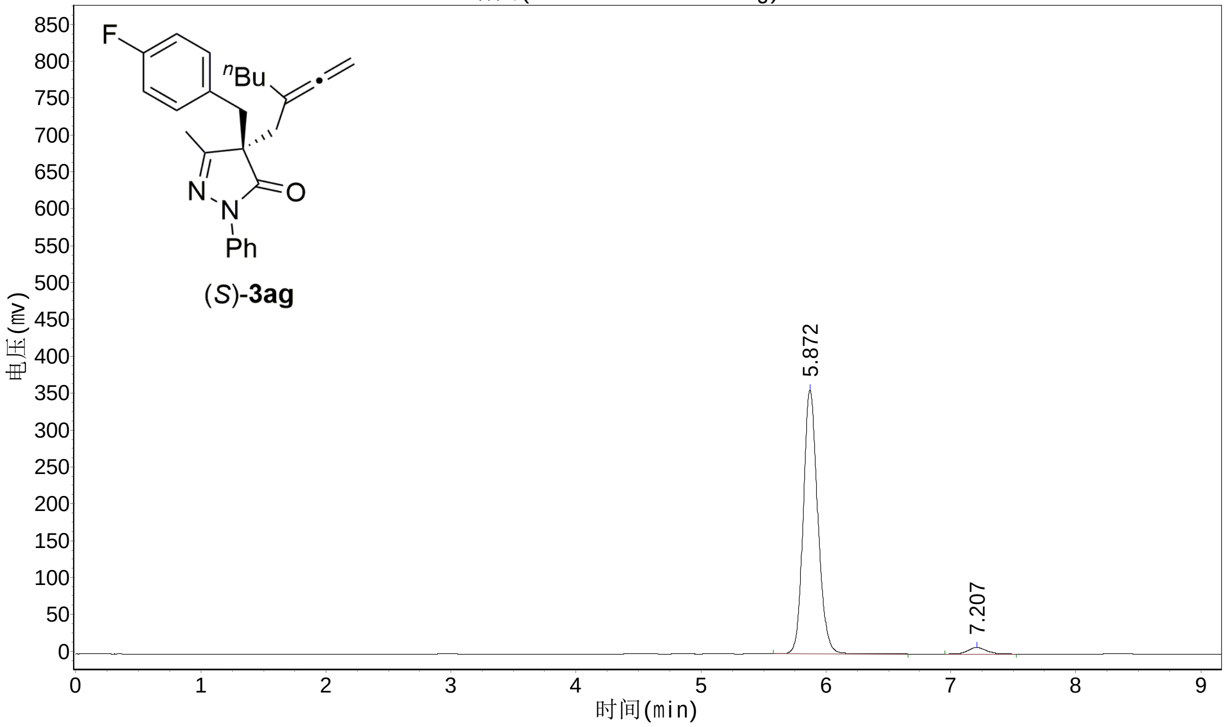

分析结果表

峰号	峰名	保留时间	峰高	峰面积	含量
1		5.872	357821.219	2883964.750	97.1755
2		7.207	8725.784	83824.648	2.8245
总计			366547.003	2967789.398	100.0000

zyc-4-105mix

实验时间：2019-07-11,11:14:27
谱图文件:D:\浙大智达\N2000\样品\S20190711111427.org
方法文件:D:\浙大智达\N2000\djx.mtd

实验者：zyc
报告时间：2019-07-11,11:37:38
积分方法:面积归一法

实验内容简介：
ia,n-hexane/i-PrOH = 90/10,1.0,254

色谱图(S20190711111427.org)

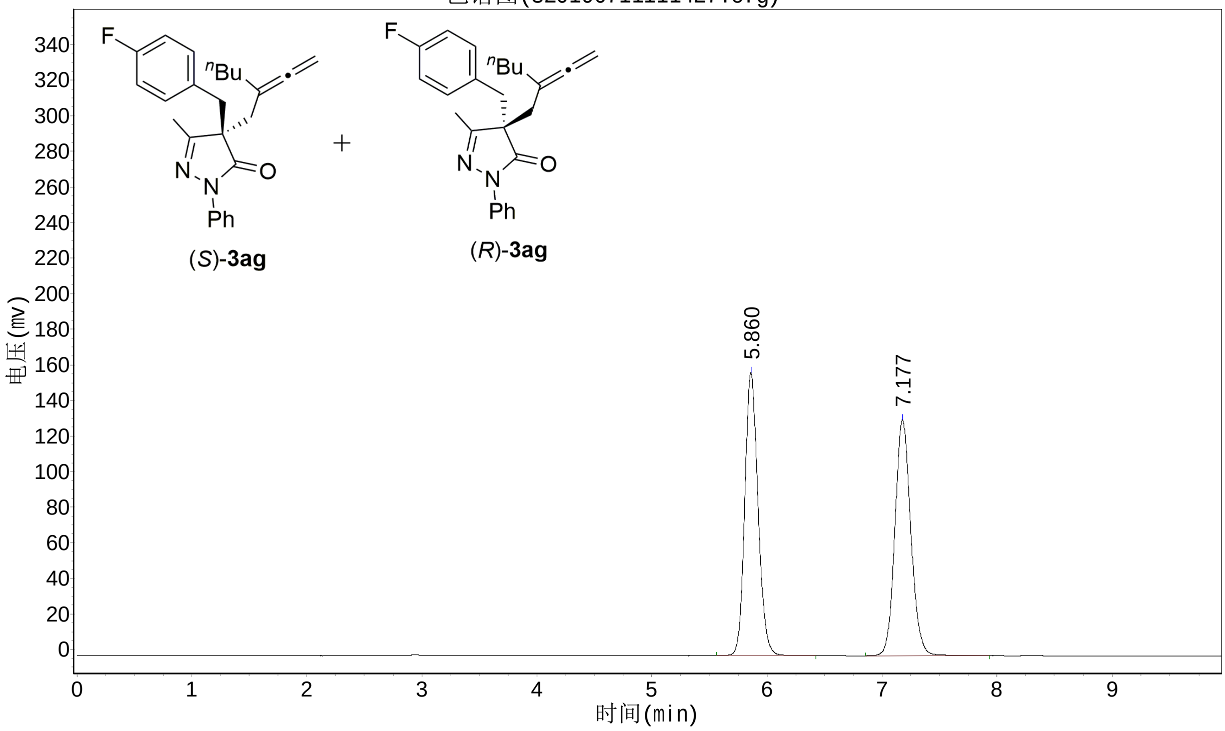

分析结果表

峰号	峰名	保留时间	峰高	峰面积	含量
1		5.860	159218.313	1276585.250	49.9698
2		7.177	132661.219	1278126.500	50.0302
总计			291879.531	2554711.750	100.0000

^{1}H NMR

2019-06-06 12:32:16.031
zyc-4-88
SOLVENT: CDCl3
Experiment = zg30
Pulse length = 14.000 usec
Relaxation delay = 1.000 sec
NA = 8
F1 = 300.130005 MHz
F2 = 1.000000 MHz

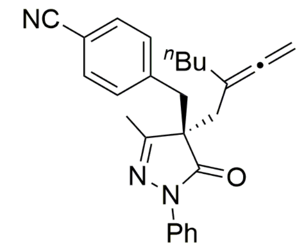

(S)-**3ah**

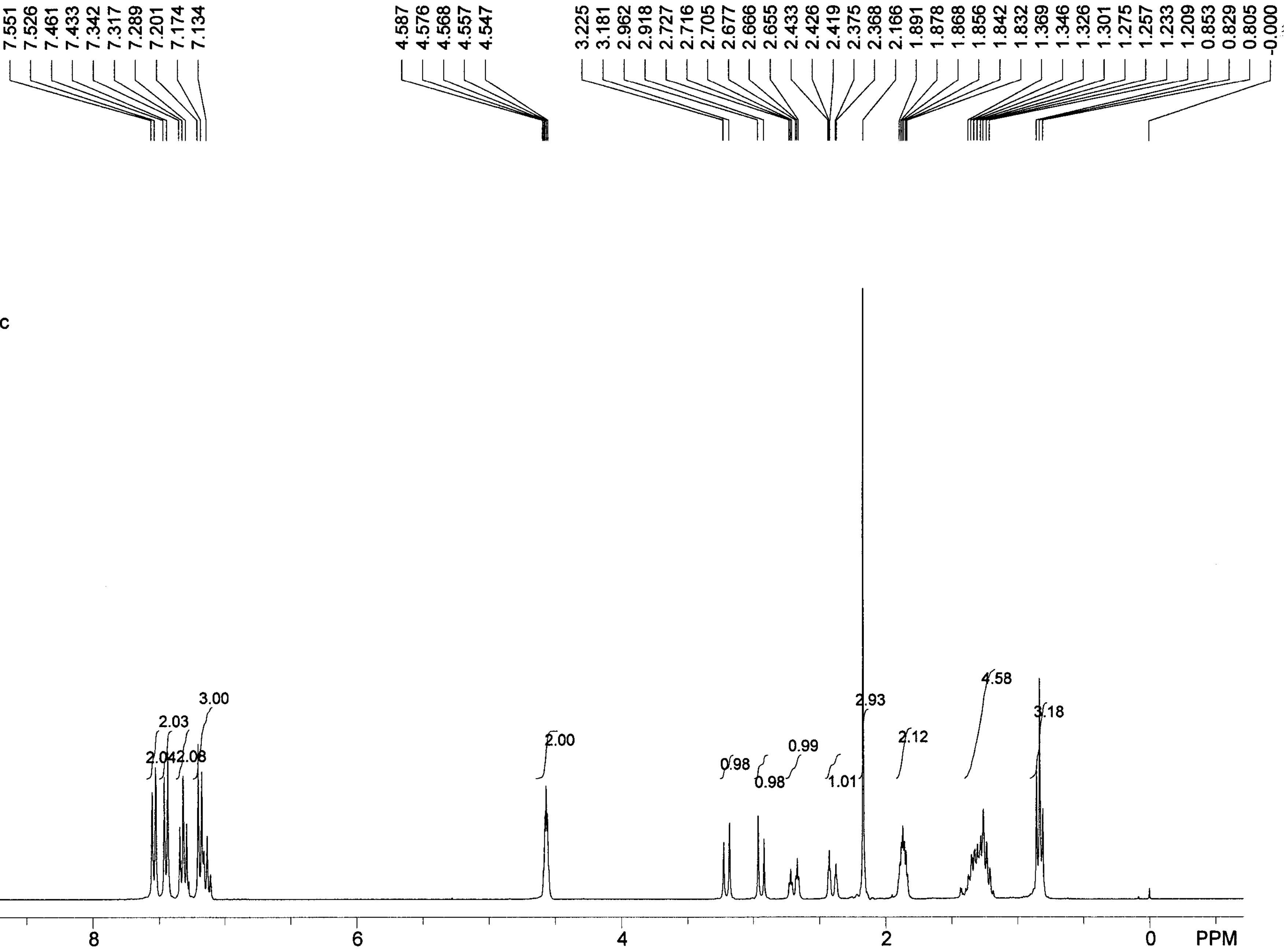

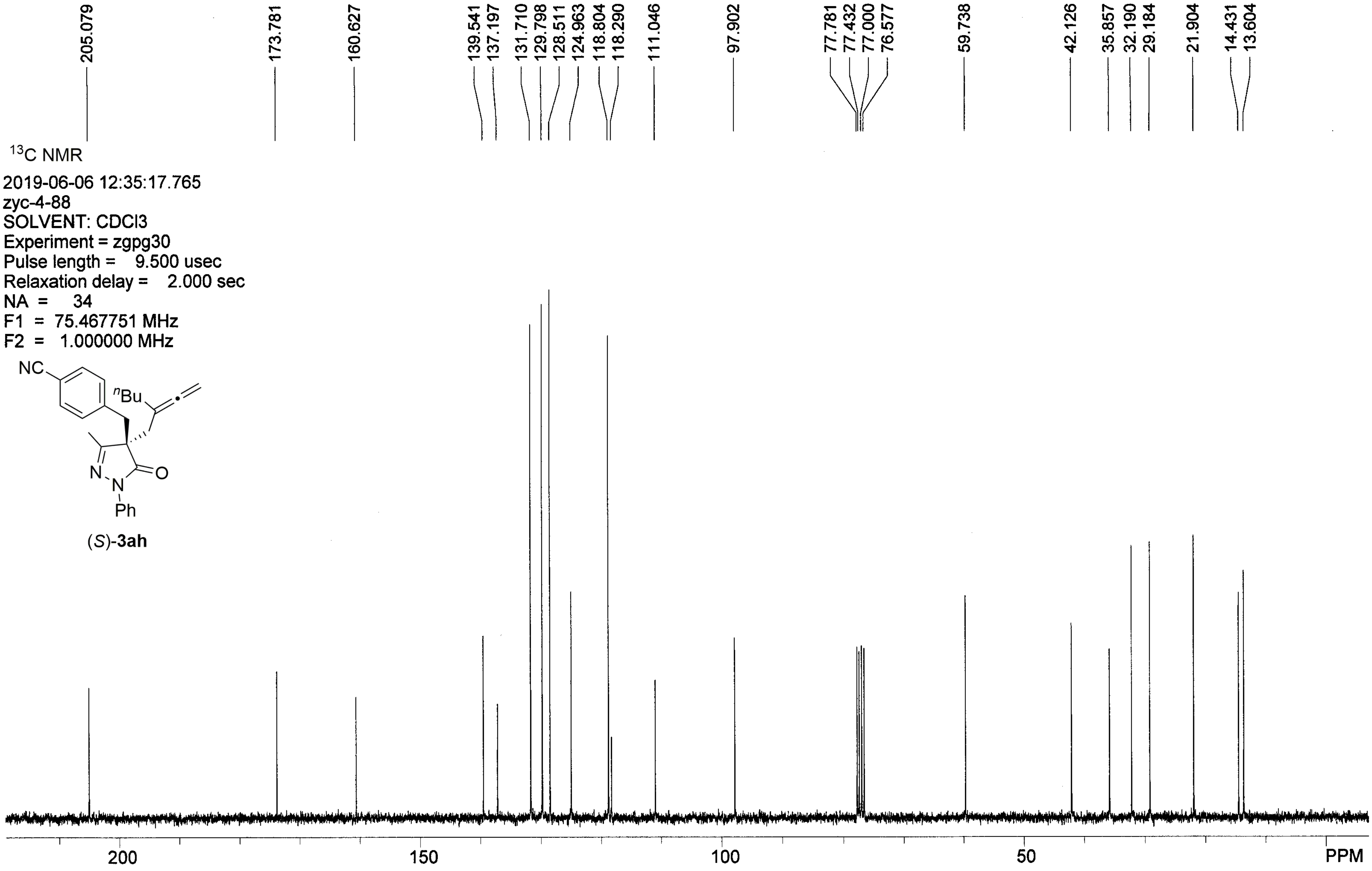
205.079
173.781
160.627
139.541
137.197
131.710
129.798
128.511
124.963
118.804
118.290
111.046
97.902
77.781
77.432
77.000
76.577
59.738
42.126
35.857
32.190
29.184
21.904
14.431
13.604
^{13}C NMR
2019-06-06 12:35:17.765
zyc-4-88
SOLVENT: CDCl3
Experiment = zgpg30
Pulse length = 9.500 usec
Relaxation delay = 2.000 sec
NA = 34
F1 = 75.467751 MHz
F2 = 1.000000 MHz
200
150
100
50
PPM
NC
^{n}Bu
N
N
O
Ph

(*S*)-**3ah**

zyc-4-88

实验时间：2019-06-06,16:17:40
谱图文件:D:\浙大智达\N2000\样品\S20190606161740.org
方法文件:D:\浙大智达\N2000\djx.mtd

实验者：zyc
报告时间：2019-06-06,18:02:55
积分方法:面积归一法

实验内容简介：
ia,n-hexane/i-PrOH = 90/10,1.0,254

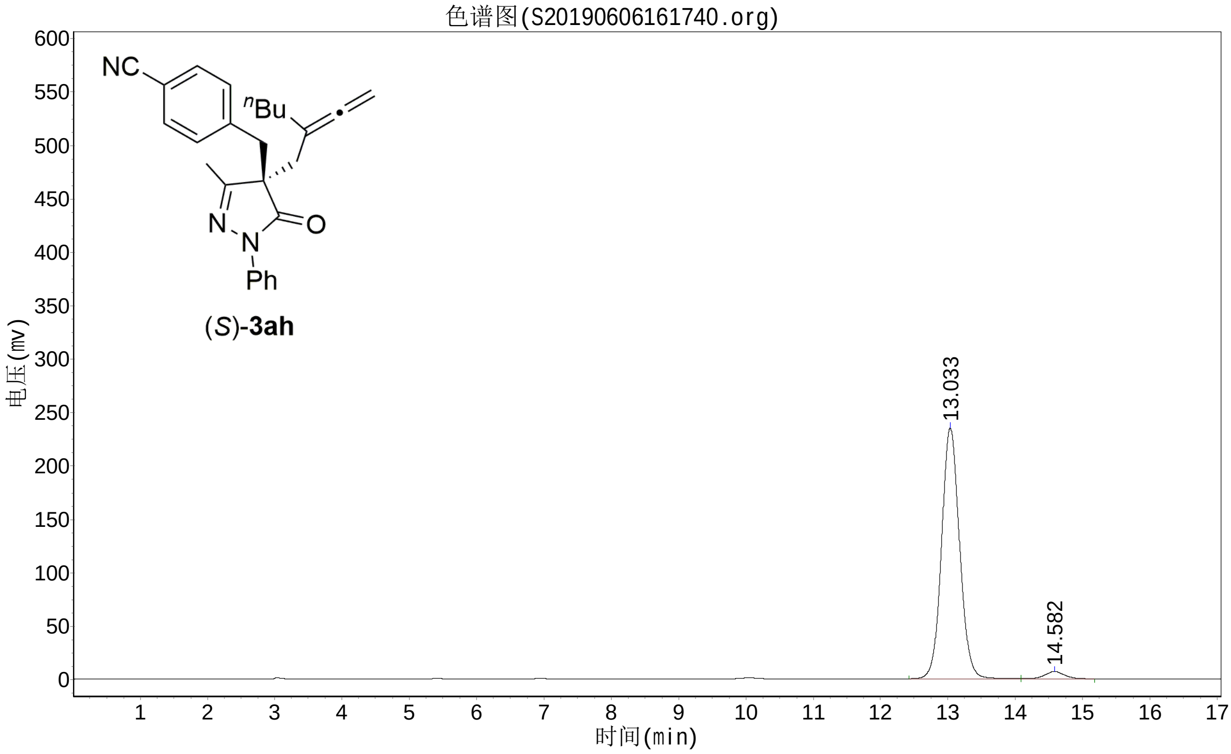

分析结果表

峰号	峰名	保留时间	峰高	峰面积	含量
1		13.033	234868.797	4374834.000	96.9086
2		14.582	6735.401	139557.734	3.0914
总计			241604.198	4514391.734	100.0000

zyc-4-88mix

实验时间：2019-06-06,15:54:18
谱图文件:D:\浙大智达\N2000\样品\S20190606155418.org
方法文件:D:\浙大智达\N2000\djx.mtd

实验者：zyc
报告时间：2019-06-06,16:16:36
积分方法:面积归一法

实验内容简介：
ia,n-hexane/i-PrOH = 90/10,1.0,254

色谱图(S20190606155418.org)

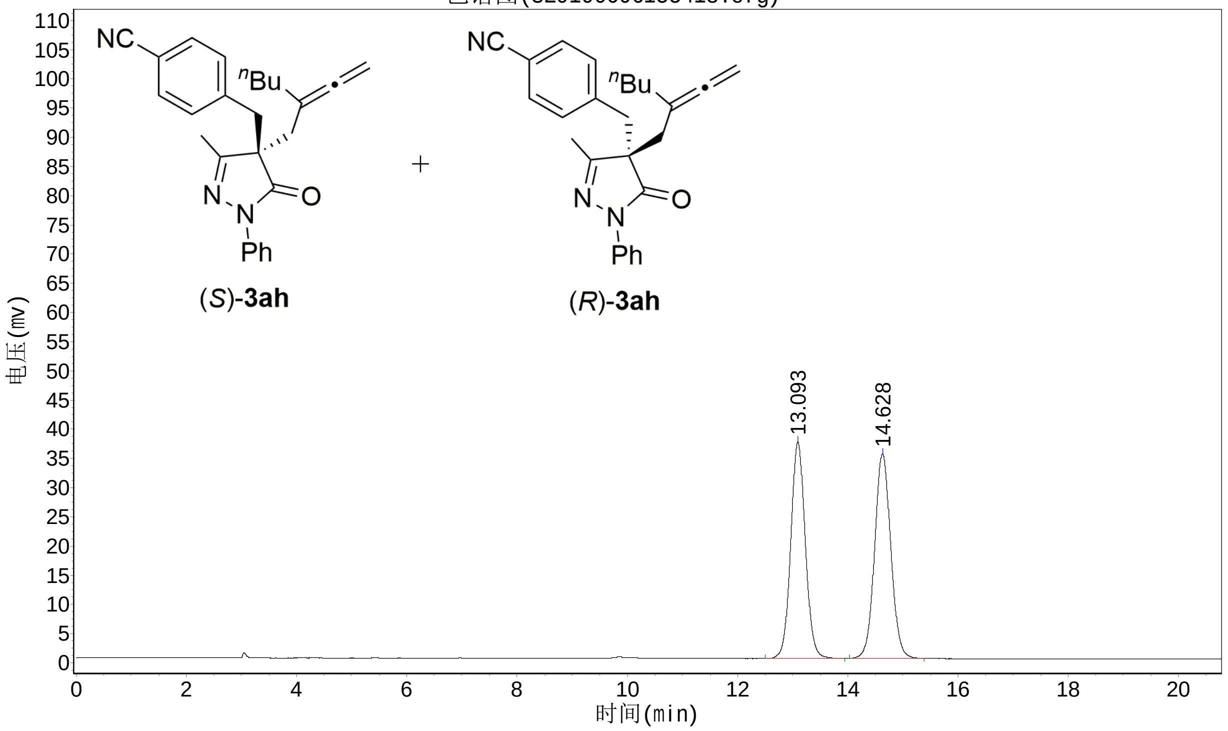

分析结果表

峰号	峰名	保留时间	峰高	峰面积	含量
1		13.093	37058.094	679914.625	48.7912
2		14.628	35058.352	713604.625	51.2088
总计			72116.445	1393519.250	100.0000

^{1}H NMR
2019-07-12 21:46:31.203
zyc-4-134
SOLVENT: CDCl3
Experiment = zg30
Pulse length =   14.000 usec
Relaxation delay =   1.000 sec
NA =   8
F1 = 300.130005 MHz
F2 = 1.000000 MHz

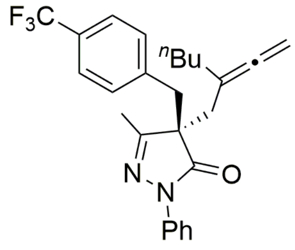

(*S*)-**3ai**

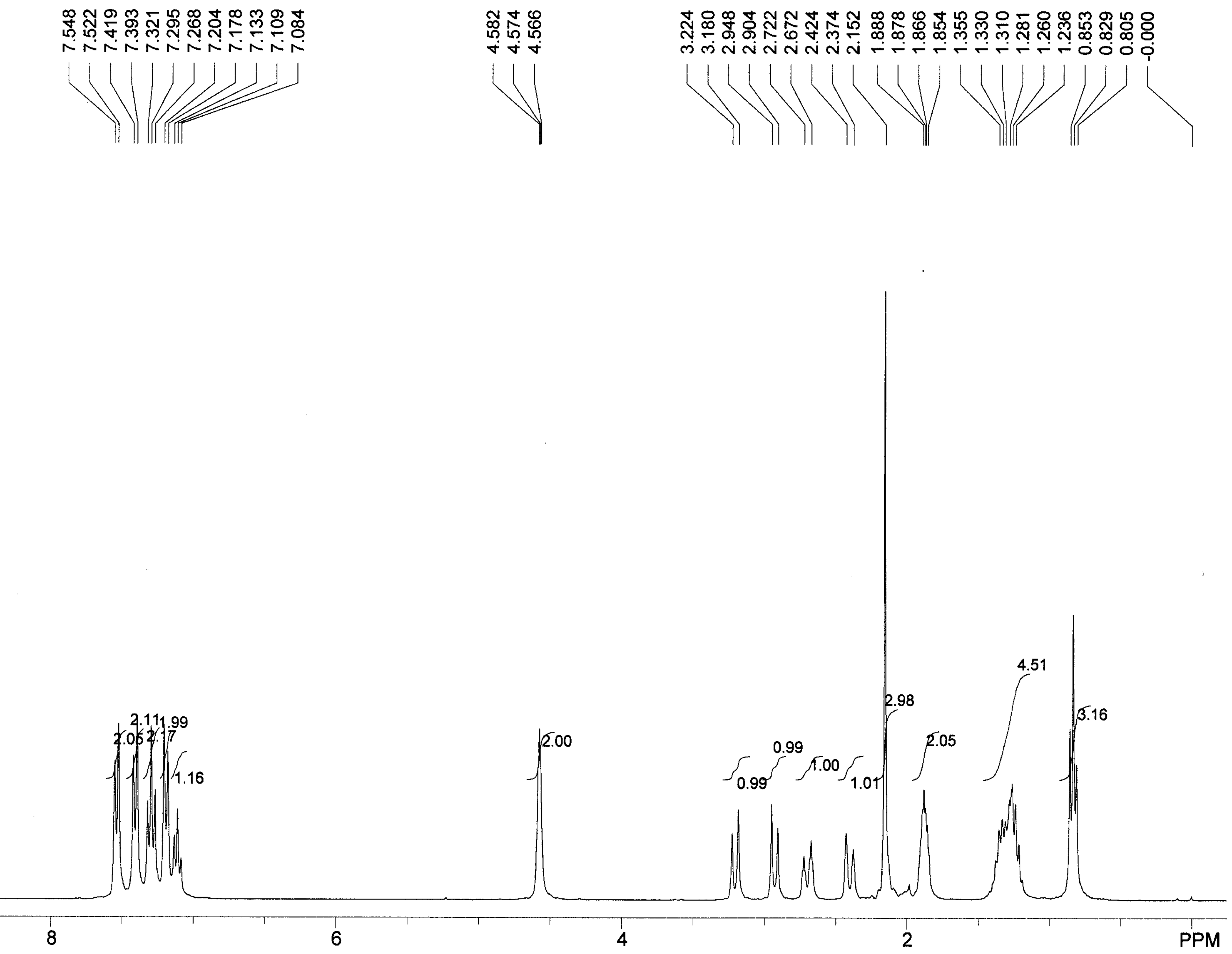

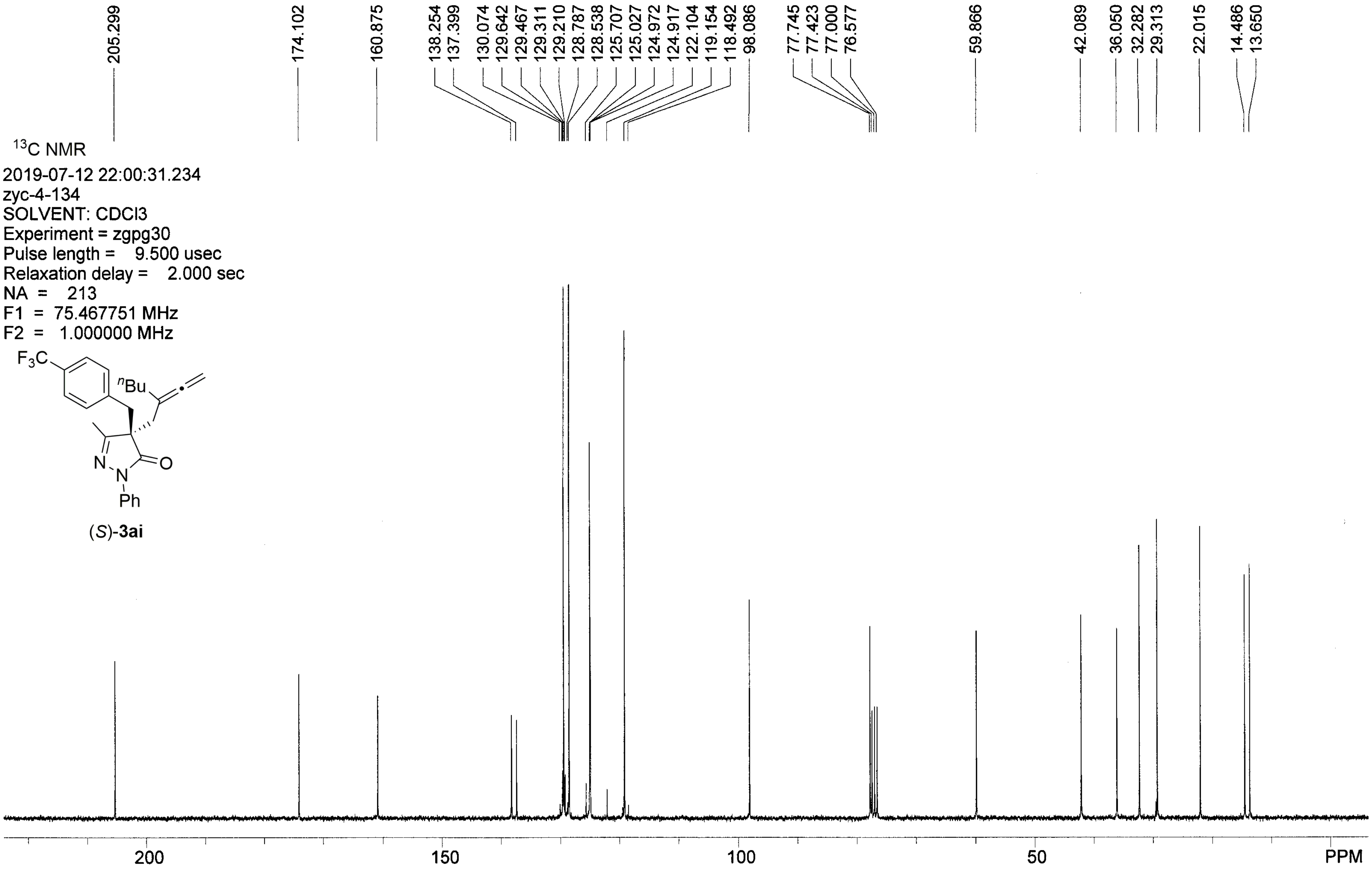

^{13}C NMR

2019-07-12 22:00:31.234
zyc-4-134
SOLVENT: CDCl3
Experiment = zgpg30
Pulse length = 9.500 usec
Relaxation delay = 2.000 sec
NA = 213
F1 = 75.467751 MHz
F2 = 1.000000 MHz

(*S*)-**3ai**

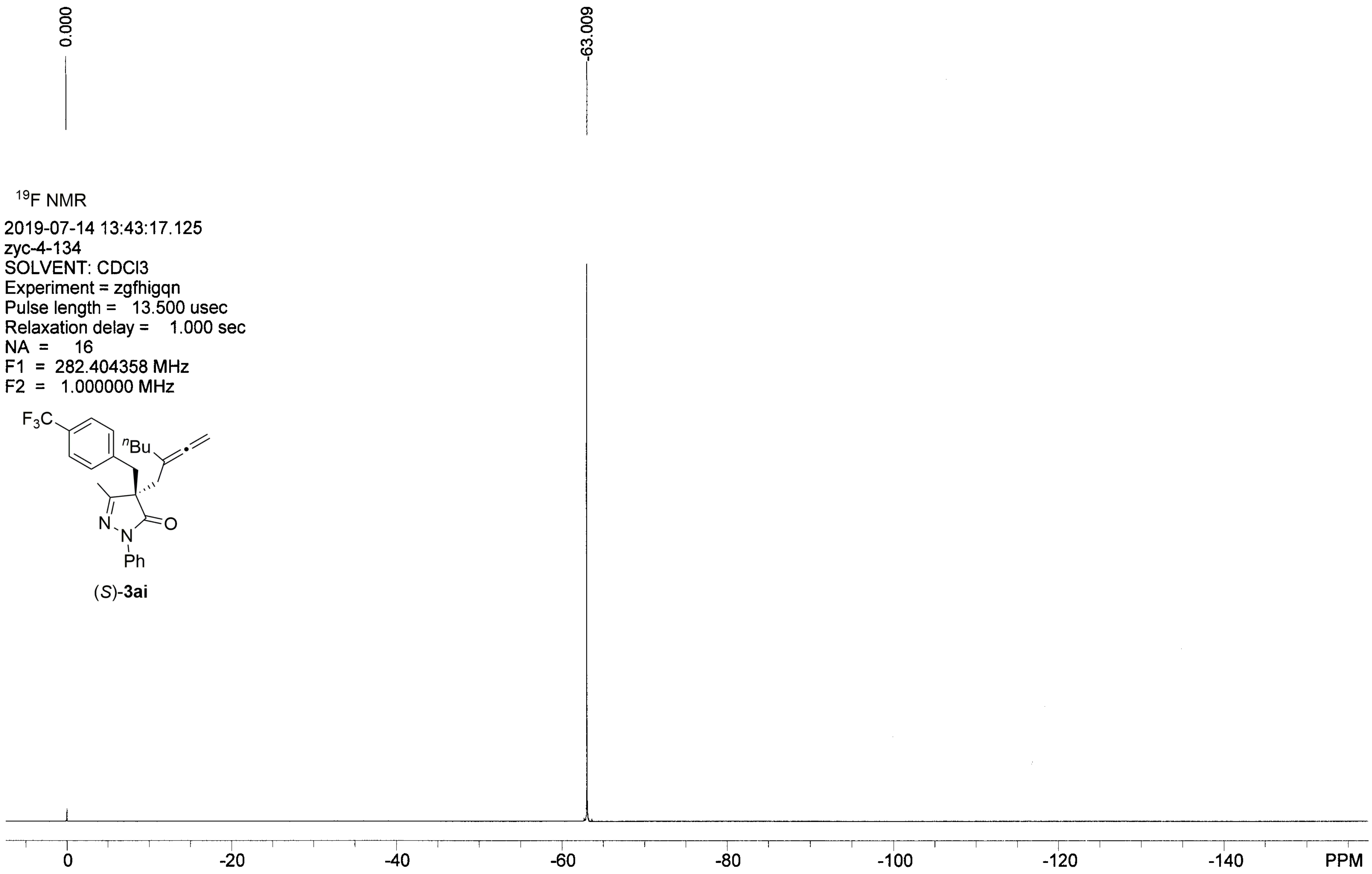

^{19}F NMR

2019-07-14 13:43:17.125
zyc-4-134
SOLVENT: CDCl3
Experiment = zgfhigqn
Pulse length =   13.500 usec
Relaxation delay =   1.000 sec
NA =   16
F1 = 282.404358 MHz
F2 =   1.000000 MHz

(*S*)-**3ai**

zyc-4-134

实验时间：2019-07-12,17:59:23
谱图文件:D:\浙大智达\N2000\样品\S20190712175923.org
方法文件:D:\浙大智达\N2000\djx.mtd

实验者：zyc
报告时间：2019-07-12,18:11:42
积分方法:面积归一法

实验内容简介：
ia,n-hexane/i-PrOH = 90/10,1.0,254

色谱图(S20190712175923.org)

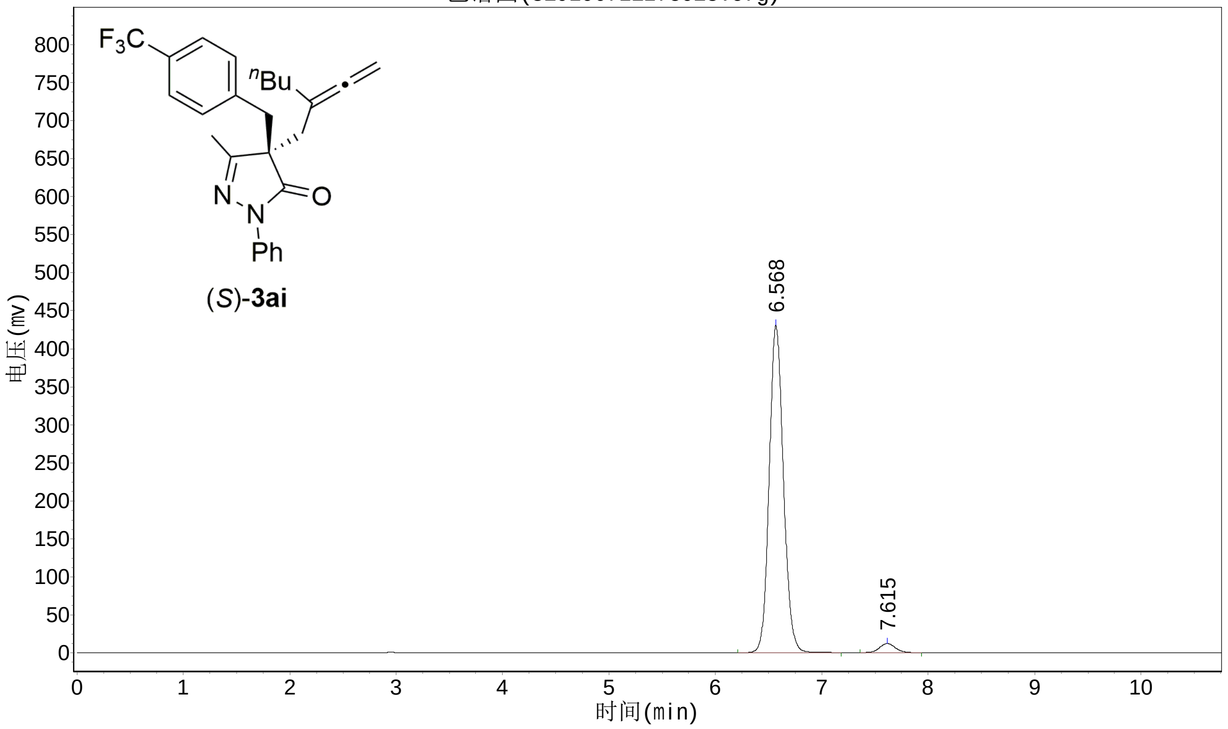

分析结果表

峰号	峰名	保留时间	峰高	峰面积	含量
1		6.568	430721.750	4000545.750	97.0170
2		7.615	11850.139	123007.094	2.9830
总计			442571.889	4123552.844	100.0000

zyc-4-134mix

实验时间：2019-07-12,18:28:13
谱图文件:D:\浙大智达\N2000\样品\S20190712182813.org
方法文件:D:\浙大智达\N2000\djx.mtd

实验者：zyc
报告时间：2019-07-12,18:42:06
积分方法:面积归一法

实验内容简介：
ia,n-hexane/i-PrOH = 90/10,1.0,254

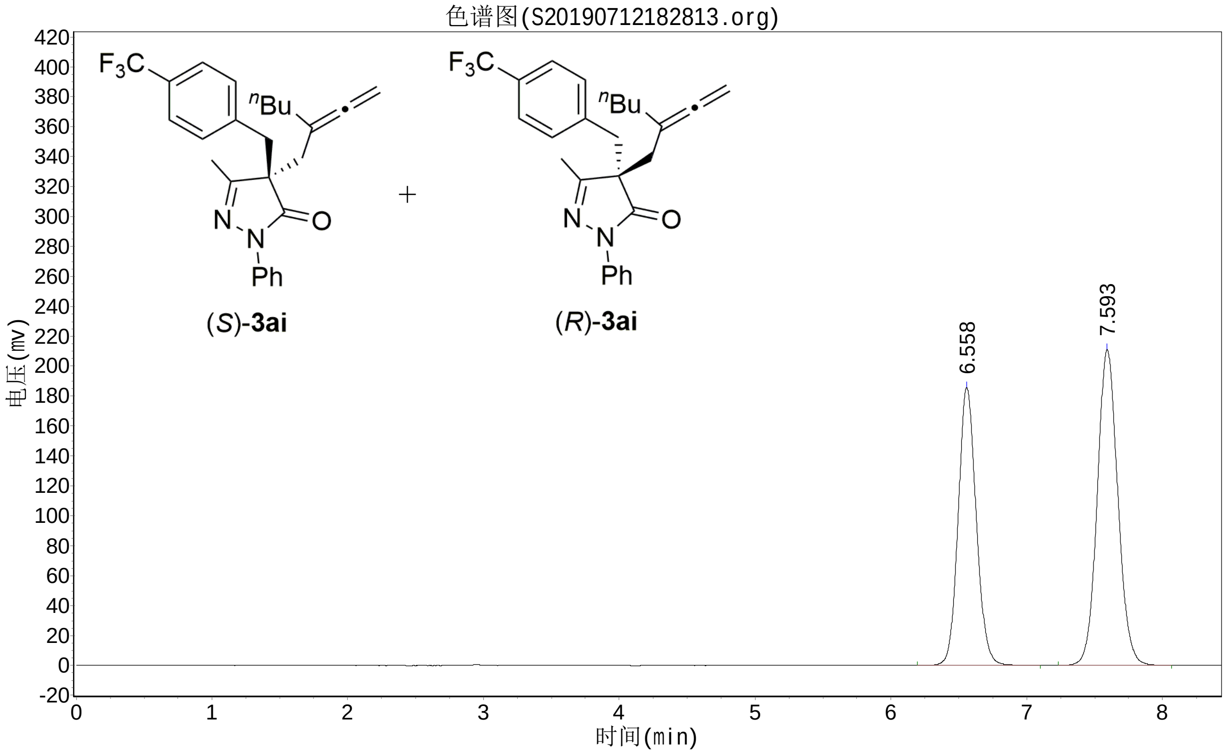

分析结果表

峰号	峰名	保留时间	峰高	峰面积	含量
1		6.558	185700.609	1711524.875	43.5248
2		7.593	211012.313	2220775.250	56.4752
总计			396712.922	3932300.125	100.0000

^{1}H NMR
2019-06-16 14:04:20.343
zyc-4-101
SOLVENT: CDCl3
Experiment = zg30
Pulse length = 14.000 usec
Relaxation delay = 1.000 sec
NA = 8
F1 = 300.130005 MHz
F2 = 1.000000 MHz

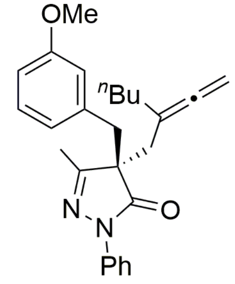

(*S*)-**3aj**

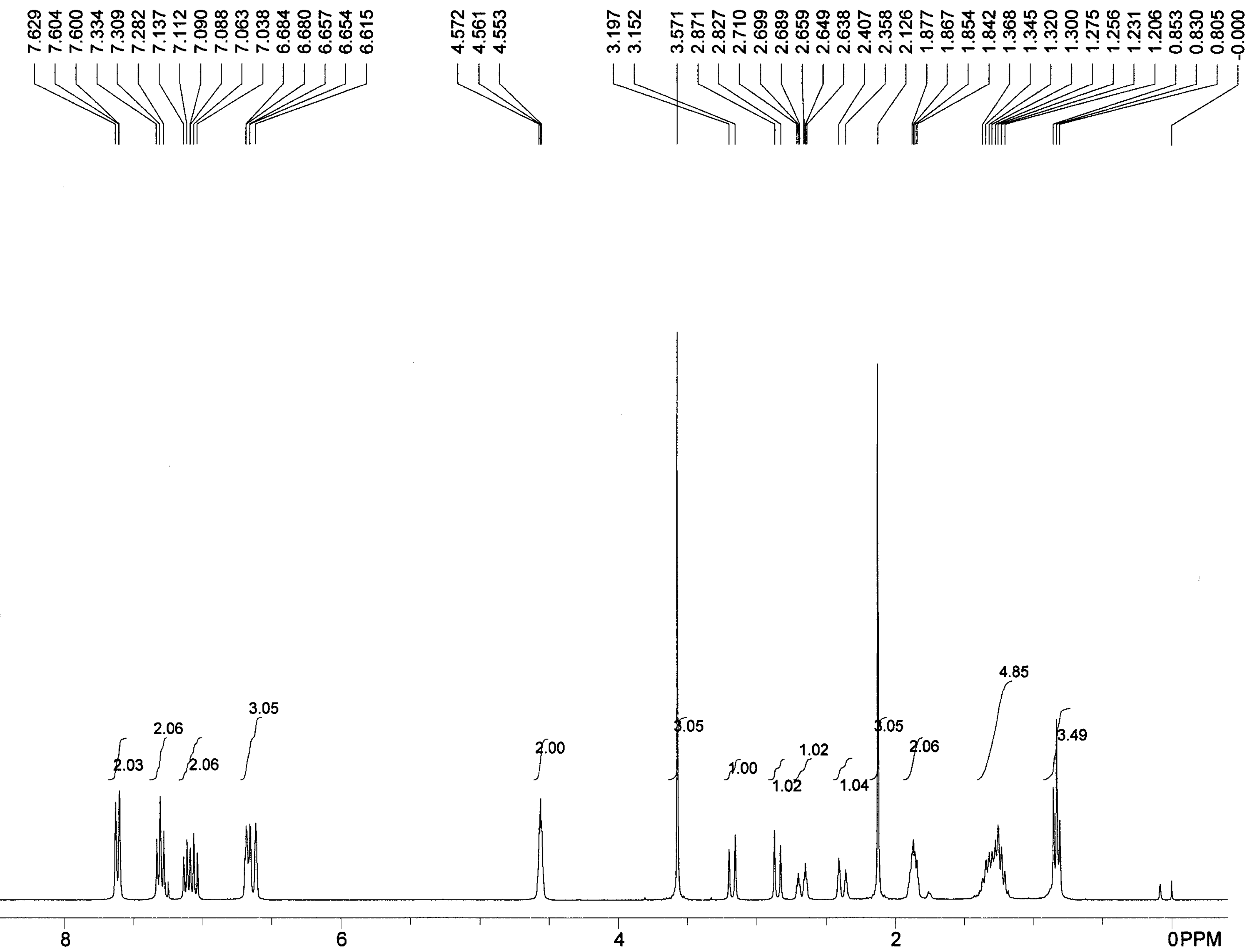

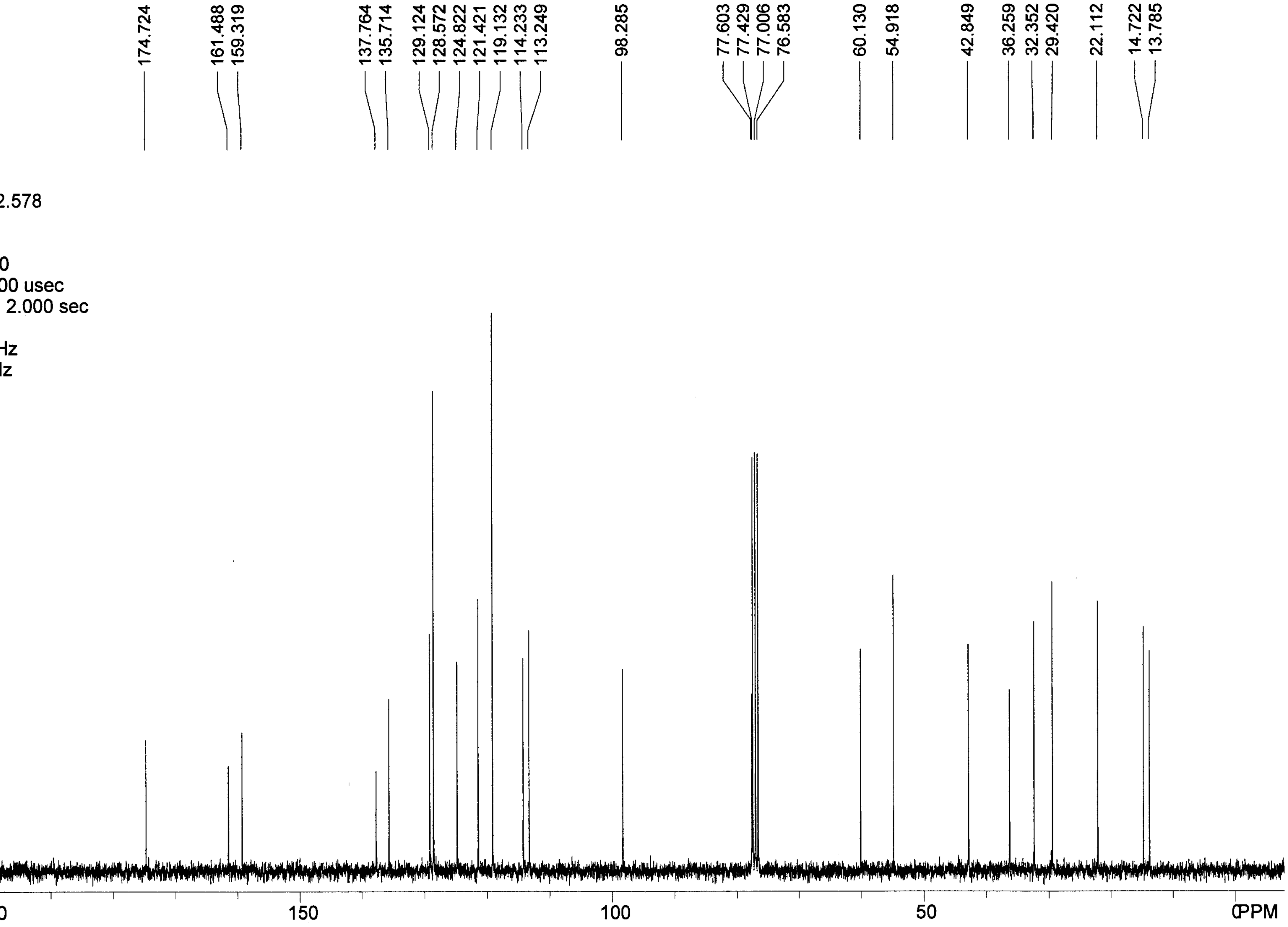

^{13}C NMR
2019-06-16 14:12:12.578
zyc-4-101
SOLVENT: CDCl3
Experiment = zgpg30
Pulse length = 9.500 usec
Relaxation delay = 2.000 sec
NA = 101
F1 = 75.467751 MHz
F2 = 1.000000 MHz

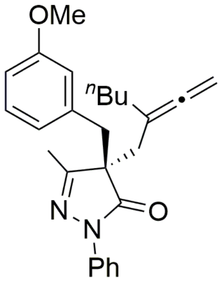

(*S*)-**3aj**

zyc-4-101

实验时间：2019-06-16,16:46:15
谱图文件:D:\浙大智达\N2000\样品\S20190616164615.org
方法文件:D:\浙大智达\N2000\djx.mtd

实验者：zyc
报告时间：2019-06-16,16:59:54
积分方法:面积归一法

实验内容简介：
ia,n-hexane/i-PrOH = 90/10,1.0,254

色谱图(S20190616164615.org)

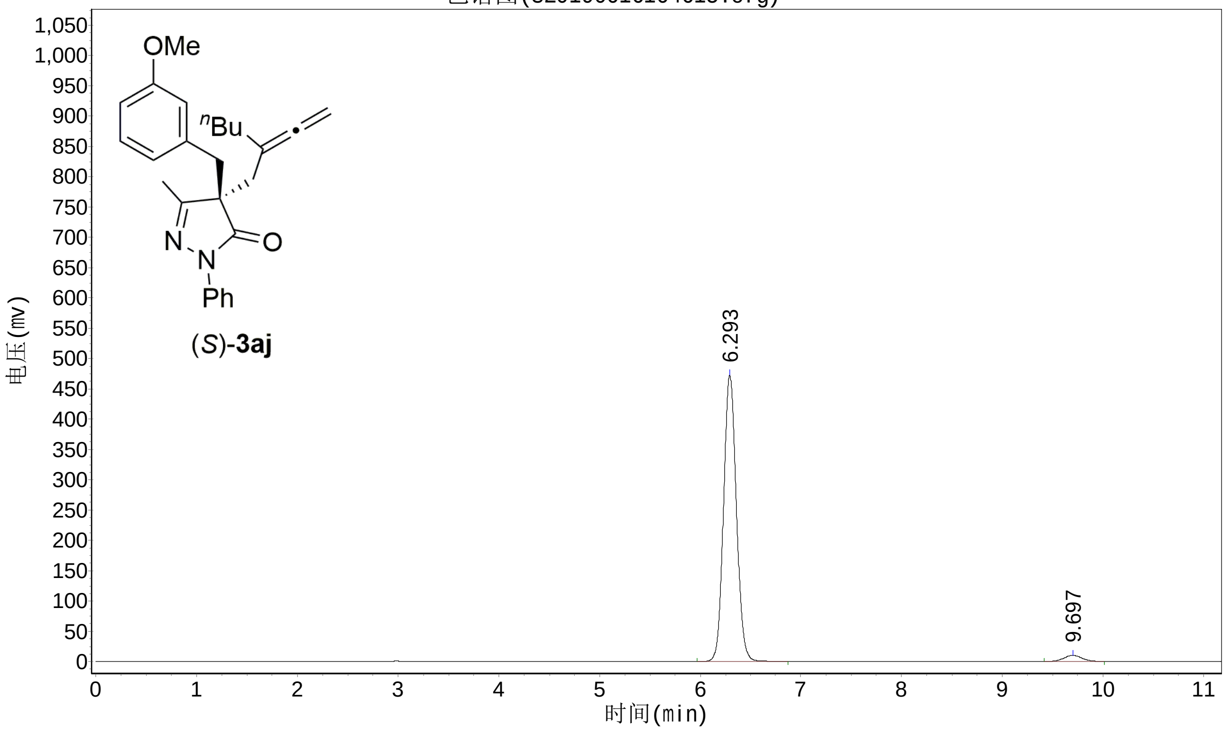

分析结果表

峰号	峰名	保留时间	峰高	峰面积	含量
1		6.293	472555.031	4176963.250	96.9930
2		9.697	9902.858	129495.008	3.0070
总计			482457.890	4306458.258	100.0000

zyc-4-101mix

实验时间：2019-06-16,16:32:37
谱图文件:D:\浙大智达\N2000\样品\S20190616163237.org
方法文件:D:\浙大智达\N2000\djx.mtd

实验者：zyc
报告时间：2019-06-16,16:51:54
积分方法:面积归一法

实验内容简介：
ia,n-hexane/i-PrOH = 90/10,1.0,254

色谱图(S20190616163237.org)

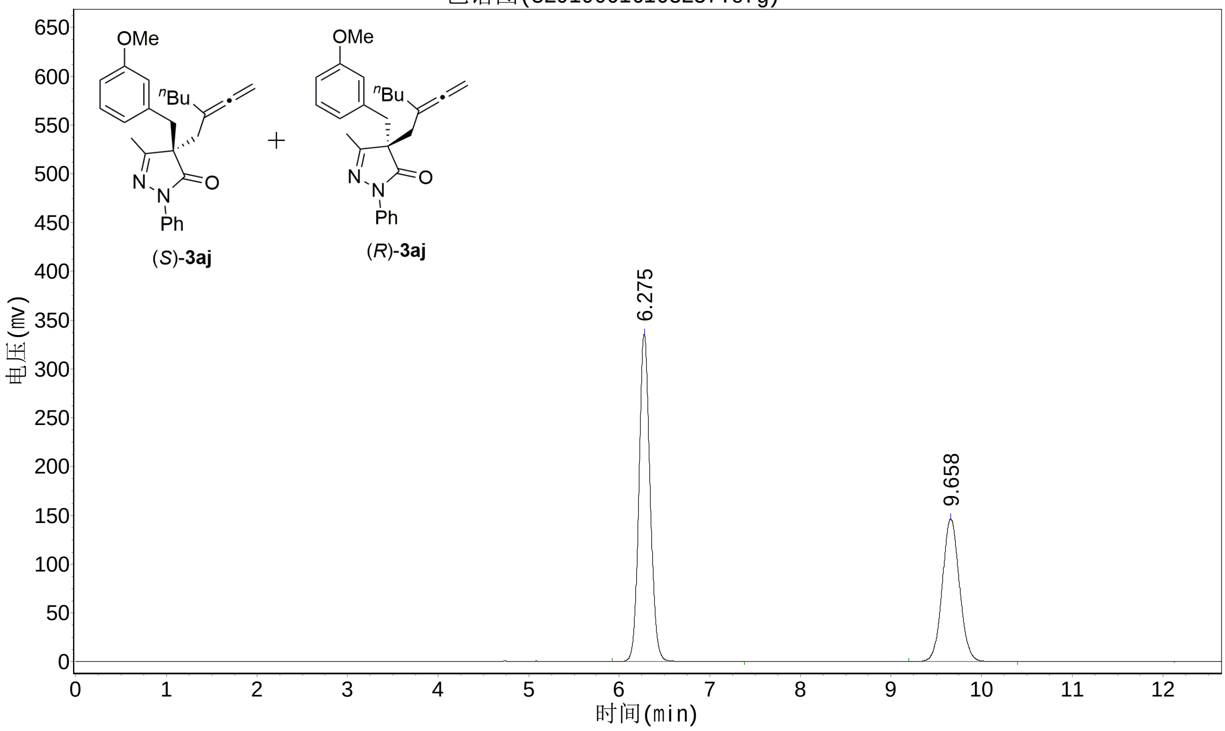

分析结果表

峰号	峰名	保留时间	峰高	峰面积	含量
1		6.275	335769.969	2880302.500	59.8318
2		9.658	146577.000	1933696.250	40.1682
总计			482346.969	4813998.750	100.0000

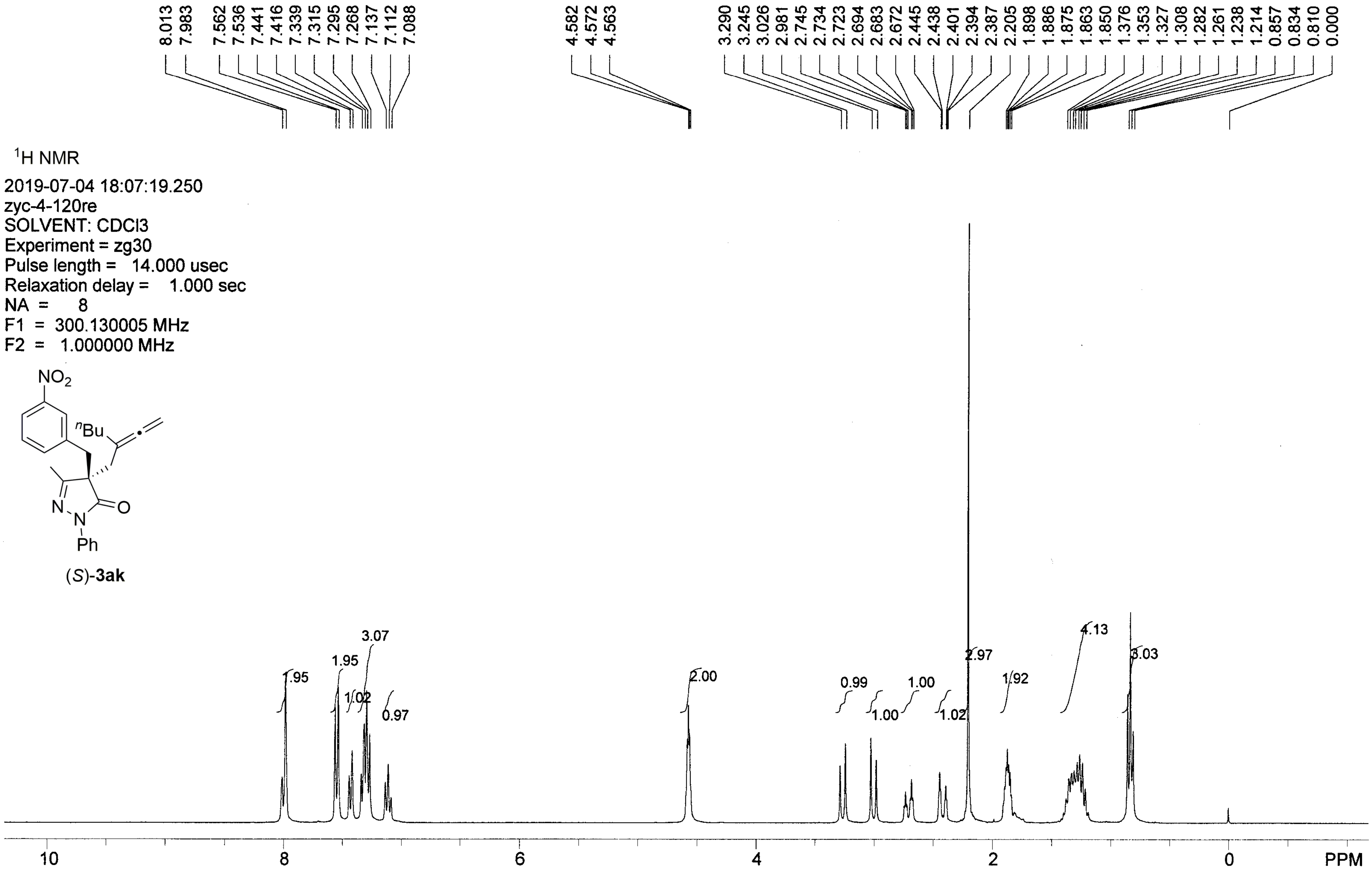
8.013
7.983
7.562
7.536
7.441
7.416
7.339
7.315
7.295
7.268
7.137
7.112
7.088
4.582
4.572
4.563
3.290
3.245
3.026
2.981
2.745
2.734
2.723
2.694
2.683
2.672
2.445
2.438
2.401
2.394
2.387
2.205
1.898
1.886
1.875
1.863
1.850
1.376
1.353
1.327
1.308
1.282
1.261
1.238
1.214
0.857
0.834
0.810
0.000
^{1}H NMR
2019-07-04 18:07:19.250
zyc-4-120re
SOLVENT: CDCl3
Experiment = zg30
Pulse length = 14.000 usec
Relaxation delay = 1.000 sec
NA = 8
F1 = 300.130005 MHz
F2 = 1.000000 MHz
NO_2
^{n}Bu
N
N
O
Ph
1.95
1.95
3.07
1.02
0.97
2.00
0.99
1.00
1.00
1.02
2.97
1.92
4.13
3.03
10
8
6
4
2
0
PPM

(*S*)-**3ak**

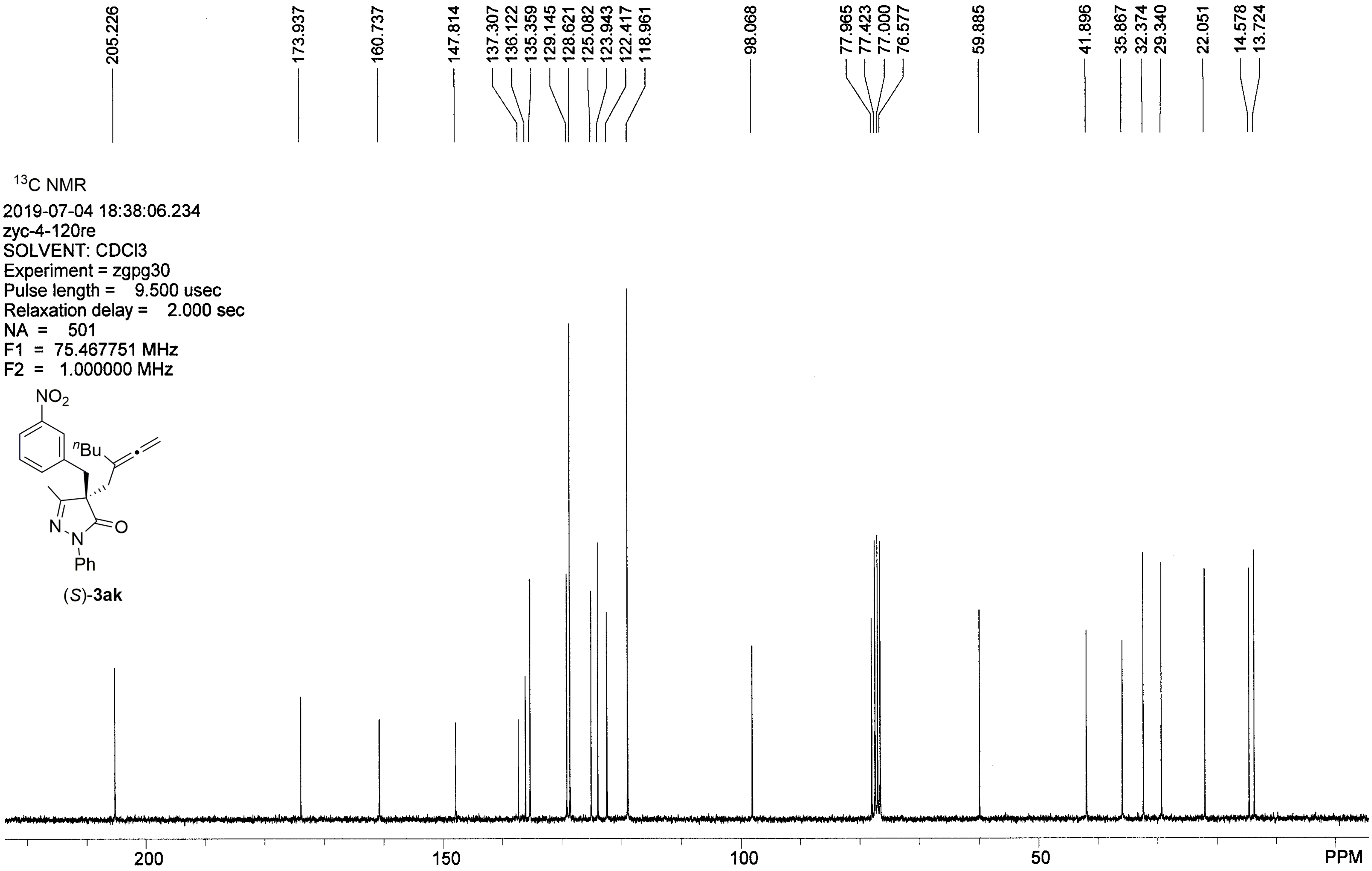

^{13}C NMR
2019-07-04 18:38:06.234
zyc-4-120re
SOLVENT: CDCl3
Experiment = zgpg30
Pulse length = 9.500 usec
Relaxation delay = 2.000 sec
NA = 501
F1 = 75.467751 MHz
F2 = 1.000000 MHz

(*S*)-**3ak**

zyc-4-120re

实验时间：2019-07-04,21:42:13
谱图文件:D:\浙大智达\N2000\样品\S20190704214213.org
方法文件:D:\浙大智达\N2000\djx.mtd

实验者：zyc
报告时间：2019-07-04,22:02:27
积分方法:面积归一法

实验内容简介：
ia,n-hexane/i-PrOH = 90/10,1.0,254

色谱图(S20190704214213.org)

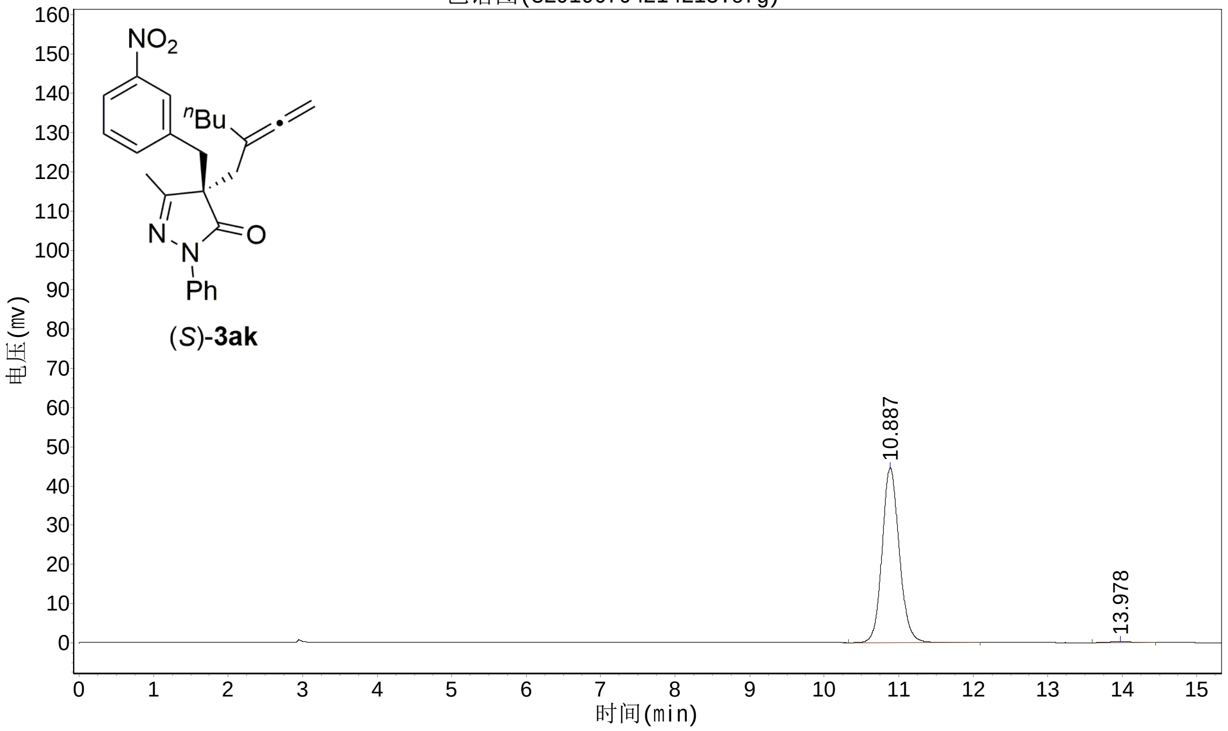

分析结果表

峰号	峰名	保留时间	峰高	峰面积	含量
1		10.887	44500.336	709666.875	99.2372
2		13.978	262.047	5455.301	0.7628
总计			44762.383	715122.176	100.0000

zyc-5-120remix

实验时间：2020-04-27,13:25:19
谱图文件:D:\浙大智达\N2000\样品\S20200427132519.org
方法文件:D:\浙大智达\N2000\djx.mtd

实验者：zyc
报告时间：2020-04-27,13:49:38
积分方法:面积归一法

实验内容简介：
ia,n-hexane/i-PrOH = 90/10,1.0,254

色谱图(S20200427132519.org)

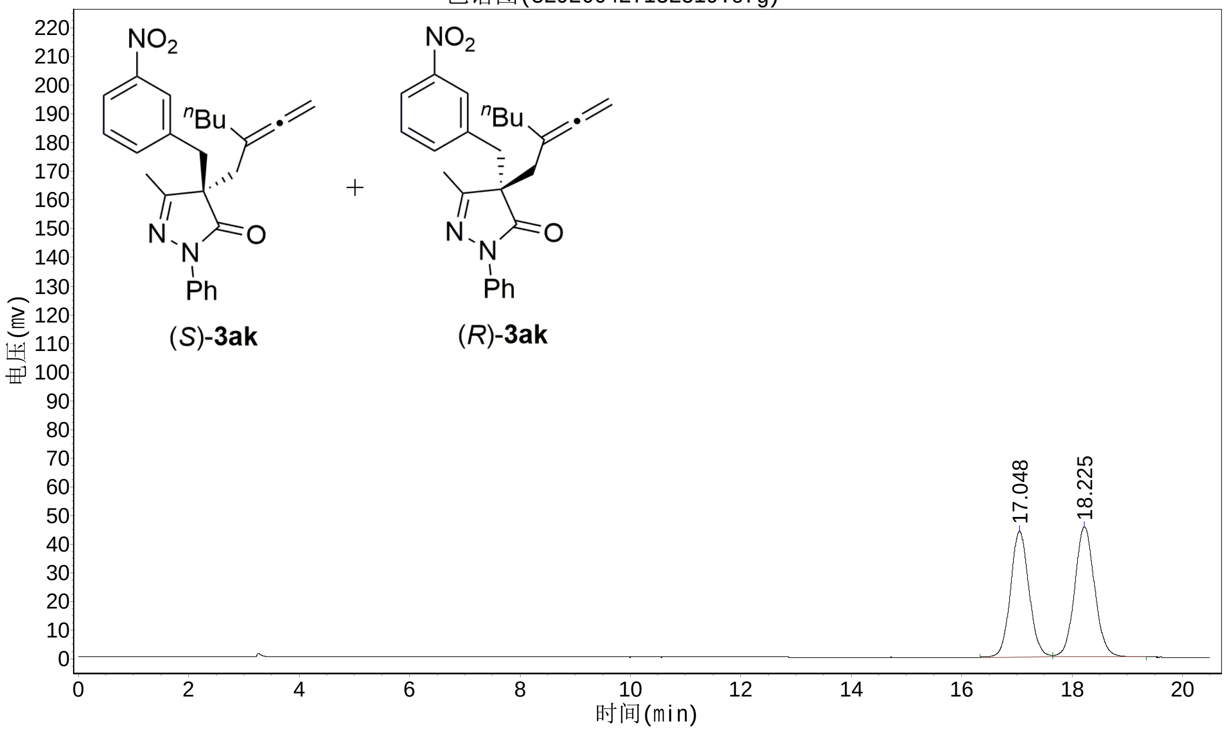

分析结果表

峰号	峰名	保留时间	峰高	峰面积	含量
1		17.048	43929.574	1022159.563	47.2614
2		18.225	45472.398	1140620.000	52.7386
总计			89401.973	2162779.563	100.0000

^{1}H NMR
2019-05-24 09:40:18.890
zyc-4-64
SOLVENT: CDCl3
Experiment = zg30
Pulse length = 14.000 usec
Relaxation delay = 1.000 sec
NA = 8
F1 = 300.130005 MHz
F2 = 1.000000 MHz

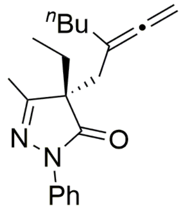

(*S*)-**3al**

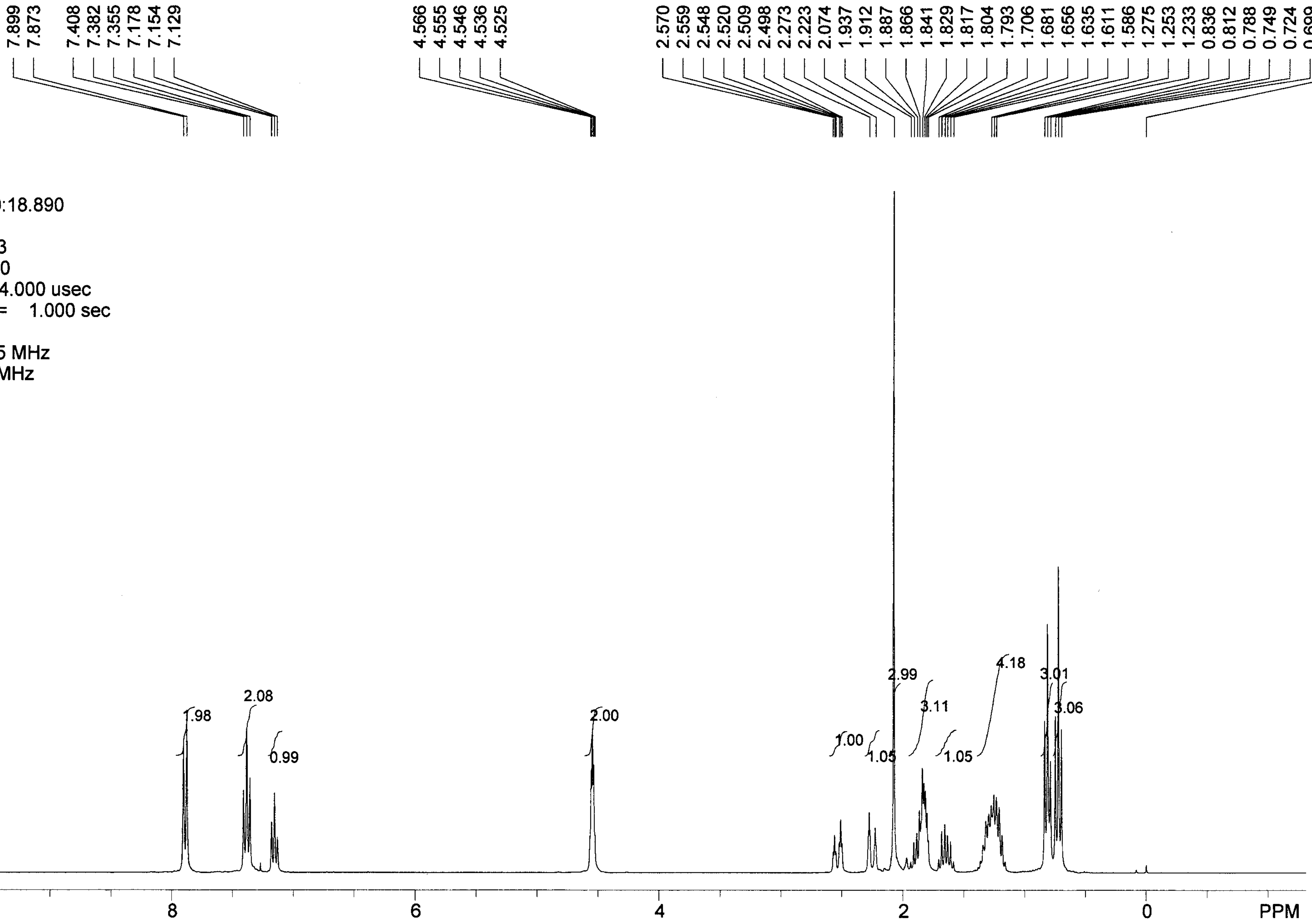

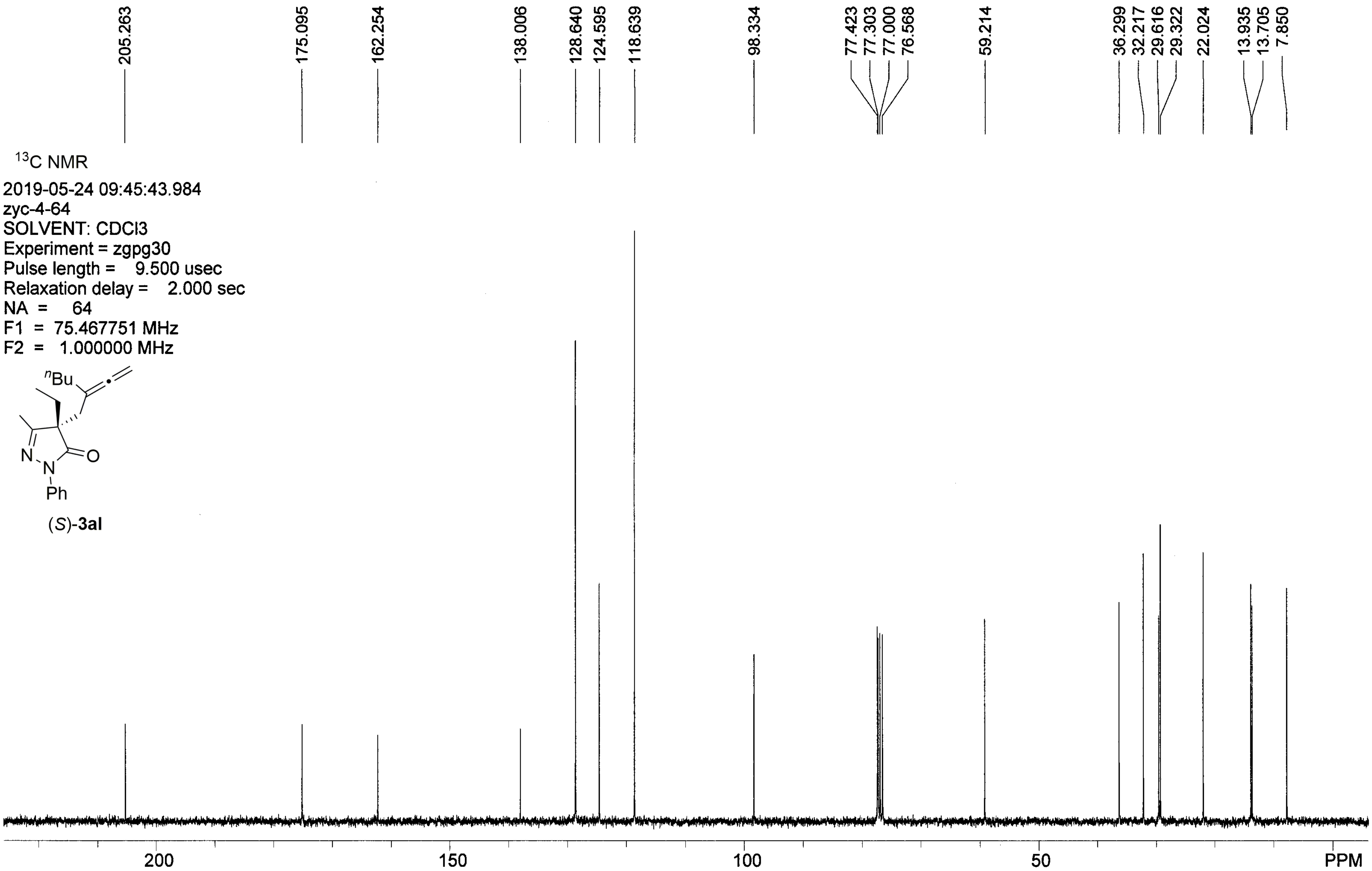

^{13}C NMR

2019-05-24 09:45:43.984
zyc-4-64
SOLVENT: CDCl3
Experiment = zgpg30
Pulse length = 9.500 usec
Relaxation delay = 2.000 sec
NA = 64
F1 = 75.467751 MHz
F2 = 1.000000 MHz

(*S*)-**3al**

zyc-4-64

实验时间：2019-05-22,19:28:31
谱图文件:D:\浙大智达\N2000\样品\S20190522192831.org
方法文件:D:\浙大智达\N2000\djx.mtd

实验者：zyc
报告时间：2019-05-22,19:39:15
积分方法:面积归一法

实验内容简介：
ia,n-hexane/i-PrOH = 95/5,1.0,254

色谱图(S20190522192831.org)

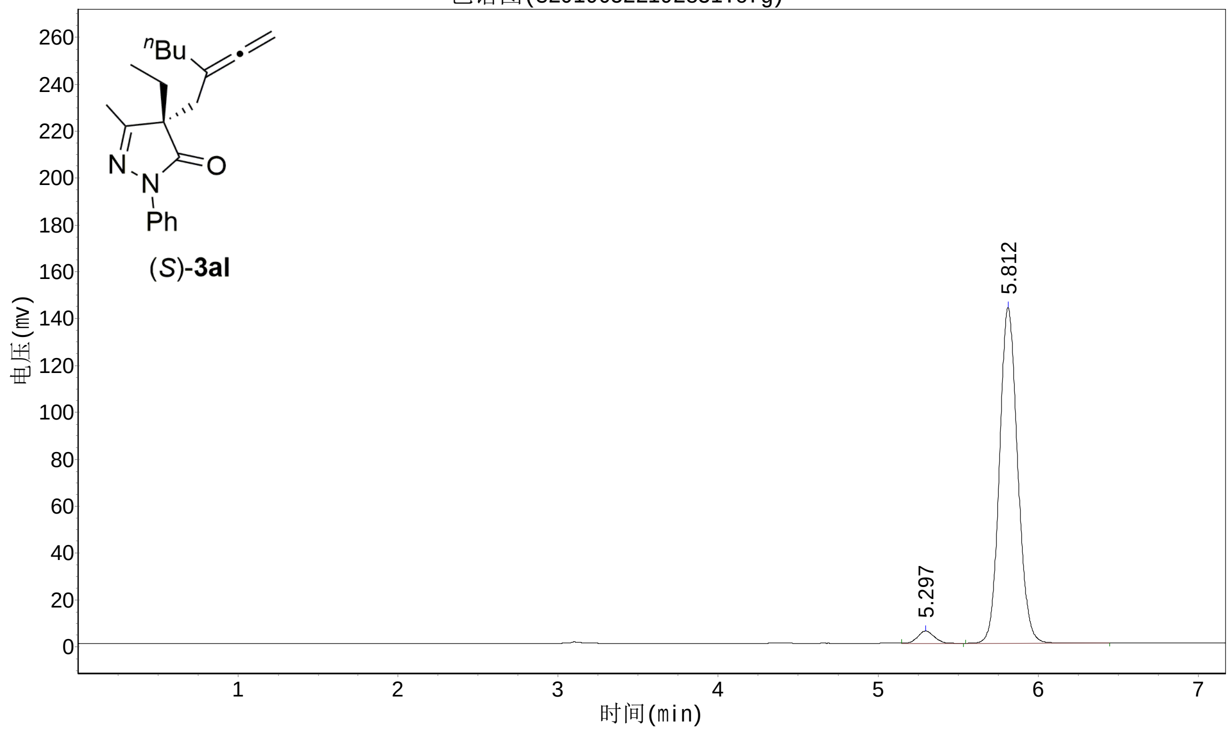

分析结果表

峰号	峰名	保留时间	峰高	峰面积	含量
1		5.297	5166.639	36413.531	3.1253
2		5.812	143335.531	1128692.500	96.8747
总计			148502.170	1165106.031	100.0000

zyc-4-64mix

实验时间：2019-05-22,19:40:45
谱图文件:D:\浙大智达\N2000\样品\S20190522194045.org
方法文件:D:\浙大智达\N2000\djx.mtd

实验者：zyc
报告时间：2019-05-22,19:51:10
积分方法:面积归一法

实验内容简介：
ia,n-hexane/i-PrOH = 95/5,1.0,254

色谱图(S20190522194045.org)

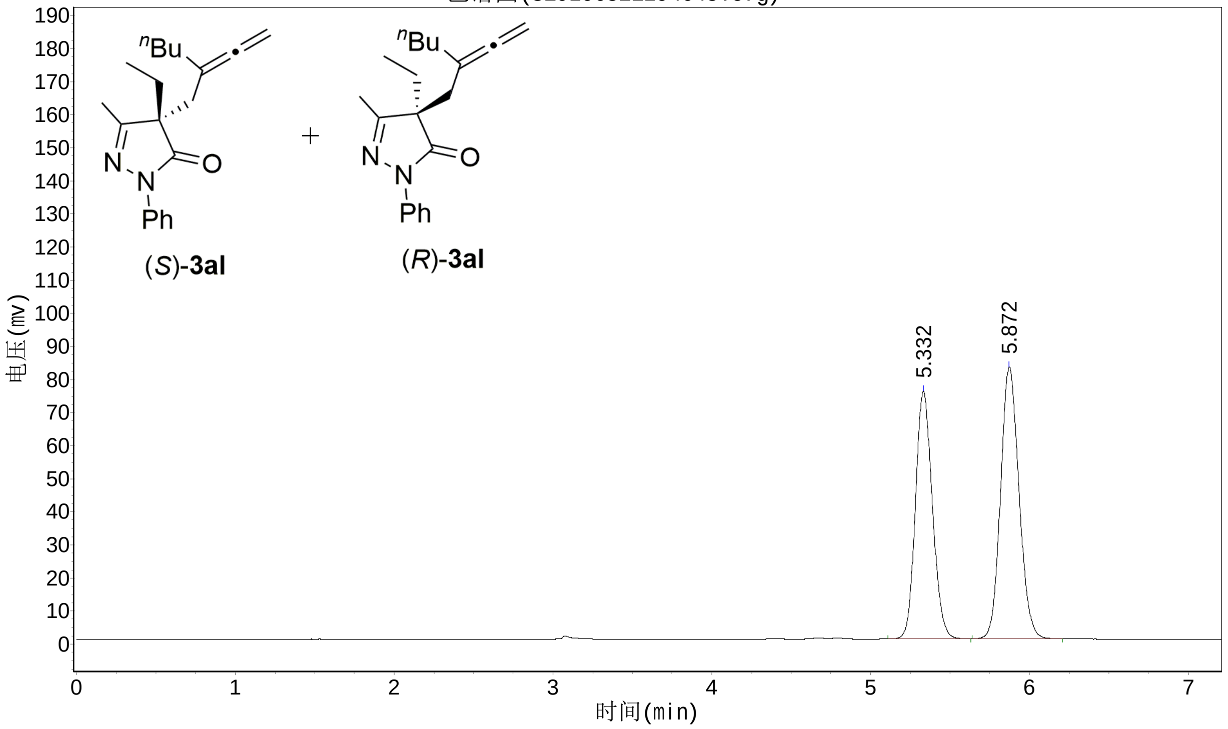

分析结果表

峰号	峰名	保留时间	峰高	峰面积	含量
1		5.332	74872.156	555788.750	45.5583
2		5.872	82200.742	664163.063	54.4417
总计			157072.898	1219951.813	100.0000

^{1}H NMR

2019-06-06 12:22:59.546
zyc-4-87
SOLVENT: CDCl3
Experiment = zg30
Pulse length = 14.000 usec
Relaxation delay = 1.000 sec
NA = 8
F1 = 300.130005 MHz
F2 = 1.000000 MHz

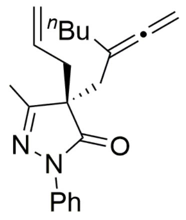

(S)-**3am**

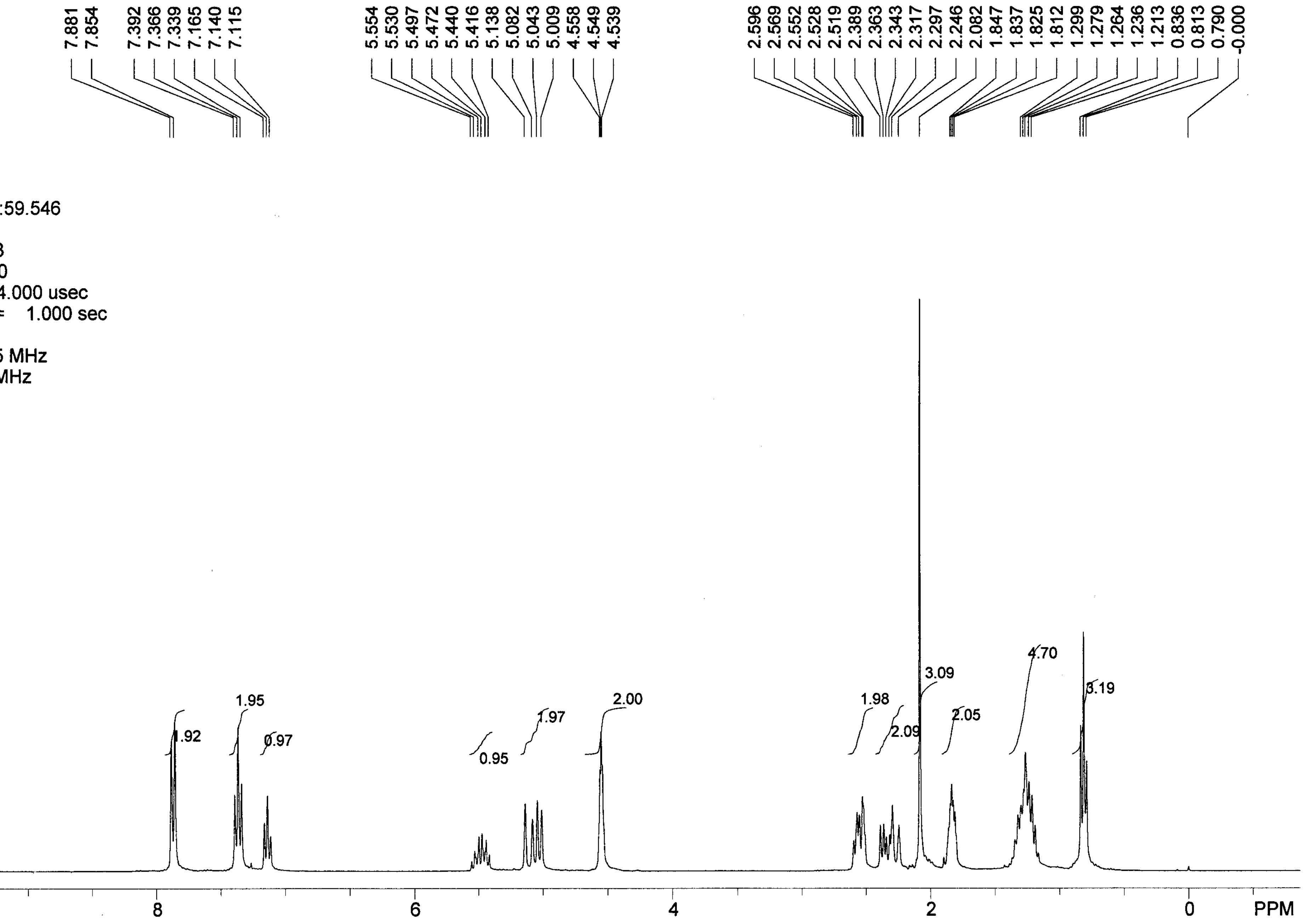

^{13}C NMR

2019-06-06 10:16:22.687
zyc-4-87
SOLVENT: CDCl3
Experiment = zgpg30
Pulse length = 9.500 usec
Relaxation delay = 2.000 sec
NA = 21
F1 = 75.467751 MHz
F2 = 1.000000 MHz

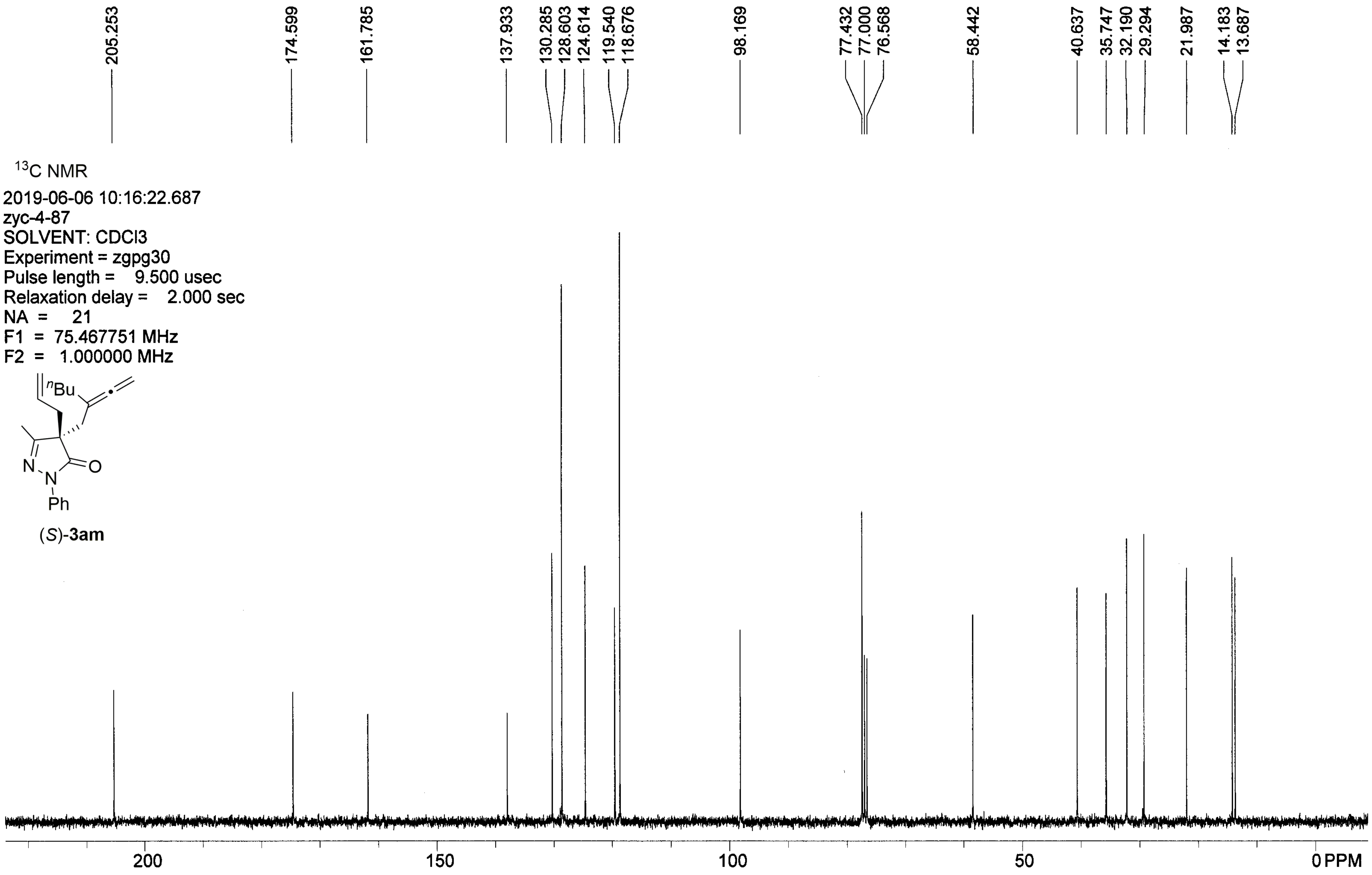

(S)-**3am**

zyc-4-87

实验时间：2019-06-06,20:55:59
谱图文件:D:\浙大智达\N2000\样品\S20190606205559.org
方法文件:D:\浙大智达\N2000\djx.mtd

实验者：zyc
报告时间：2019-06-06,21:11:16
积分方法:面积归一法

实验内容简介：
od,n-hexane/i-PrOH = 98/2,1.0,254

色谱图(S20190606205559.org)

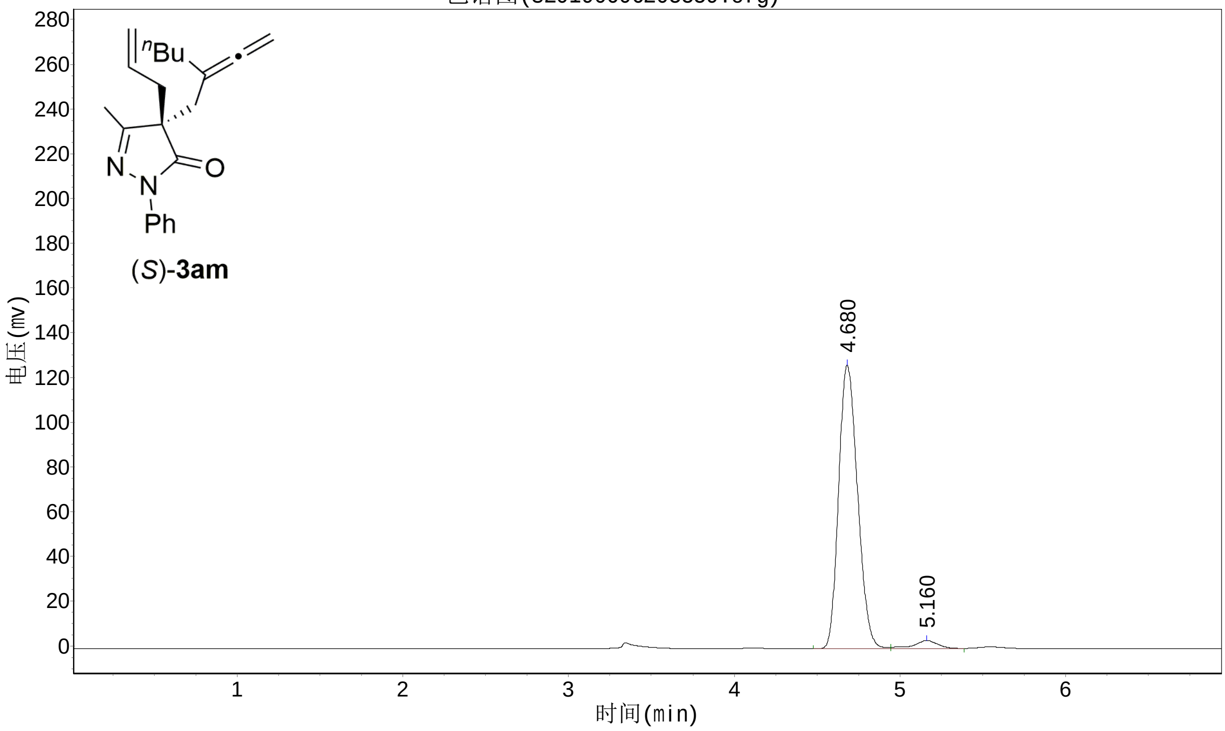

分析结果表

峰号	峰名	保留时间	峰高	峰面积	含量
1		4.680	126890.555	1008419.500	96.3601
2		5.160	3628.614	38091.730	3.6399
总计			130519.169	1046511.230	100.0000

zyc-4-87mix

实验时间：2019-06-06,20:28:28
谱图文件:D:\浙大智达\N2000\样品\S20190606202828.org
方法文件:D:\浙大智达\N2000\djx.mtd

实验者：zyc
报告时间：2019-06-06,20:39:55
积分方法:面积归一法

实验内容简介：
od,n-hexane/i-PrOH = 98/2,1.0,254

色谱图(S20190606202828.org)

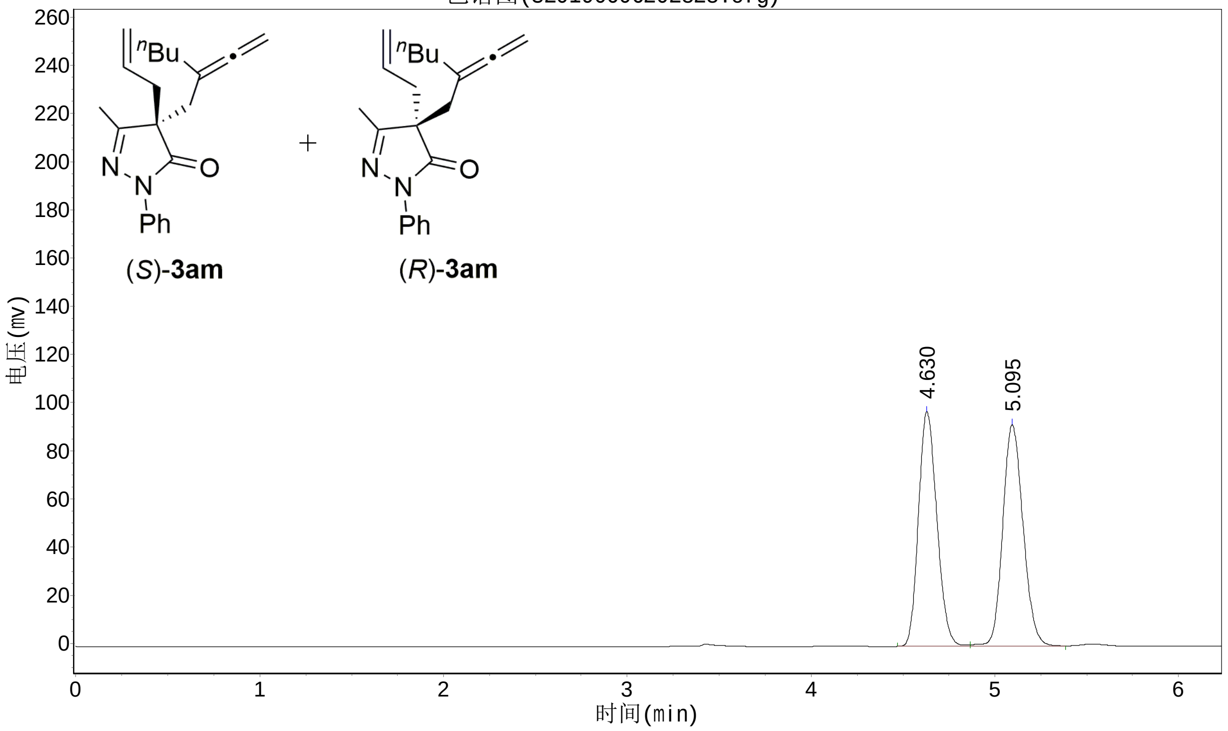

分析结果表

峰号	峰名	保留时间	峰高	峰面积	含量
1		4.630	97395.195	678758.938	48.8714
2		5.095	91996.266	710108.875	51.1286
总计			189391.461	1388867.813	100.0000

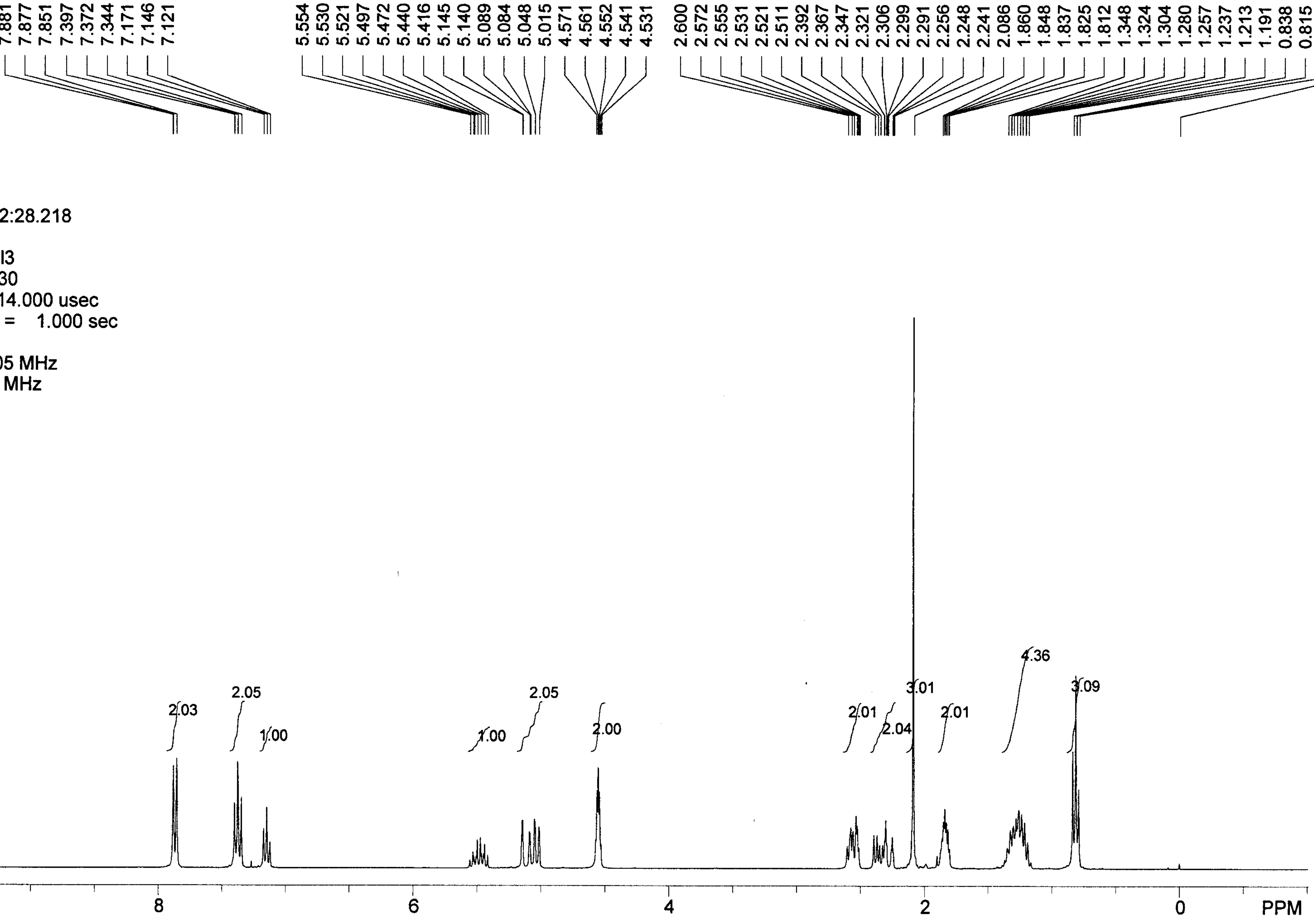
7.881 7.877 7.851 7.397 7.372 7.344 7.171 7.146 7.121
5.554 5.530 5.521 5.497 5.472 5.440 5.416 5.145 5.140 5.089 5.084 5.048 5.015 4.571 4.561 4.552 4.541 4.531
2.600 2.572 2.555 2.531 2.521 2.511 2.392 2.367 2.347 2.321 2.306 2.299 2.291 2.256 2.248 2.241 2.086 1.860 1.848 1.837 1.825 1.812 1.348 1.324 1.304 1.280 1.257 1.237 1.213 1.191 0.838 0.815 0.791 0.000
^{1}H NMR
2019-06-05 10:32:28.218
zyc-4-86
SOLVENT: CDCl3
Experiment = zg30
Pulse length = 14.000 usec
Relaxation delay = 1.000 sec
NA = 8
F1 = 300.130005 MHz
F2 = 1.000000 MHz
2.03
2.05
1.00
1.00
2.05
2.00
2.01
2.04
3.01
2.01
4.36
3.09
10
8
6
4
2
0
PPM

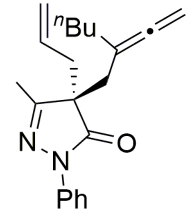
nBu
N
N
O
Ph

(*R*)-**3am**

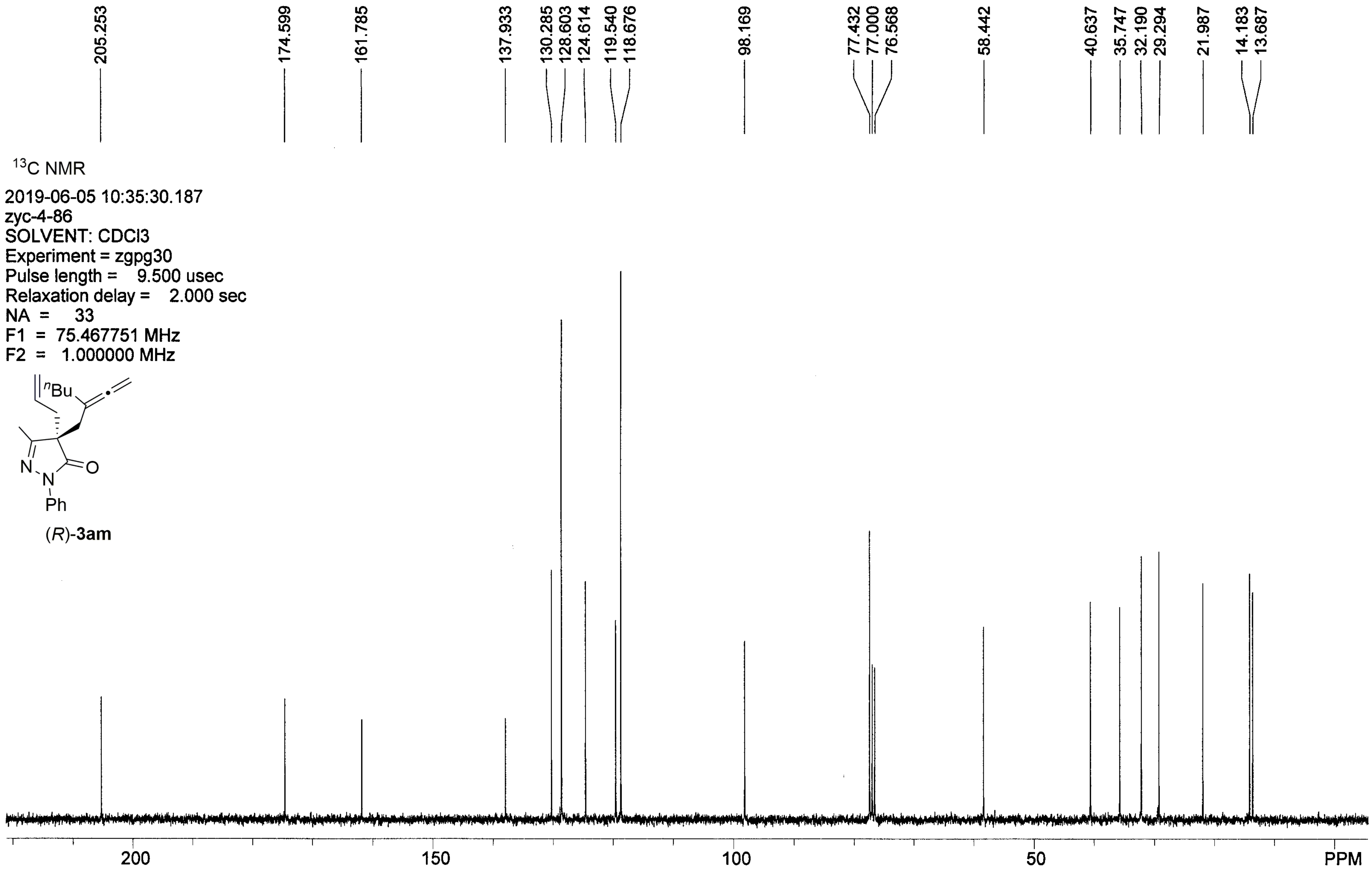
205.253
174.599
161.785
137.933
130.285
128.603
124.614
119.540
118.676
98.169
77.432
77.000
76.568
58.442
40.637
35.747
32.190
29.294
21.987
14.183
13.687
^{13}C NMR
2019-06-05 10:35:30.187
zyc-4-86
SOLVENT: CDCl3
Experiment = zgpg30
Pulse length = 9.500 usec
Relaxation delay = 2.000 sec
NA = 33
F1 = 75.467751 MHz
F2 = 1.000000 MHz
ⁿBu
N
N
O
Ph
(R)-3am
200
150
100
50
PPM

zyc-4-86

实验时间：2019-06-06,20:43:27
谱图文件:D:\浙大智达\N2000\样品\S20190606204327.org
方法文件:D:\浙大智达\N2000\djx.mtd

实验者：zyc
报告时间：2019-06-06,20:52:44
积分方法:面积归一法

实验内容简介：
od,n-hexane/i-PrOH = 98/2,1.0,254

色谱图(S20190606204327.org)

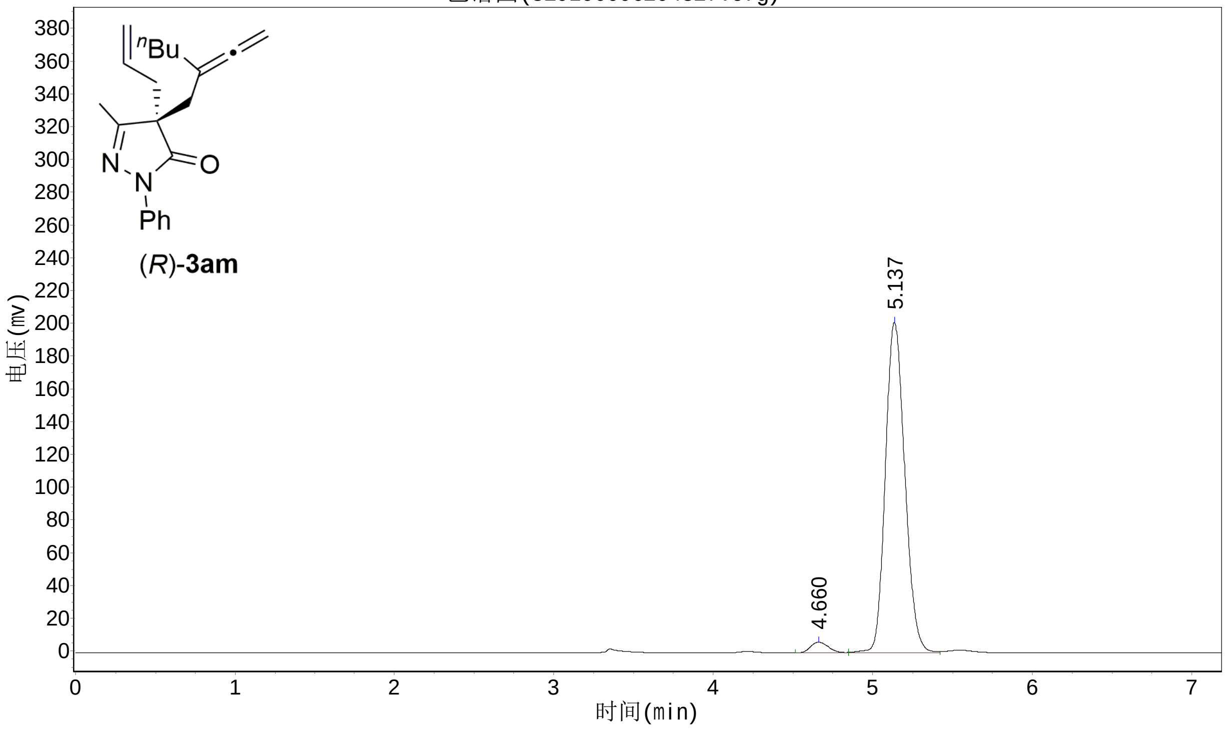

分析结果表

峰号	峰名	保留时间	峰高	峰面积	含量
1		4.660	6597.457	51965.223	2.9410
2		5.137	201756.391	1714949.375	97.0590
总计			208353.847	1766914.598	100.0000

zyc-4-87mix

实验时间：2019-06-06,20:28:28
谱图文件:D:\浙大智达\N2000\样品\S20190606202828.org
方法文件:D:\浙大智达\N2000\djx.mtd

实验者：zyc
报告时间：2019-06-06,20:39:55
积分方法:面积归一法

实验内容简介：
od,n-hexane/i-PrOH = 98/2,1.0,254

色谱图(S20190606202828.org)

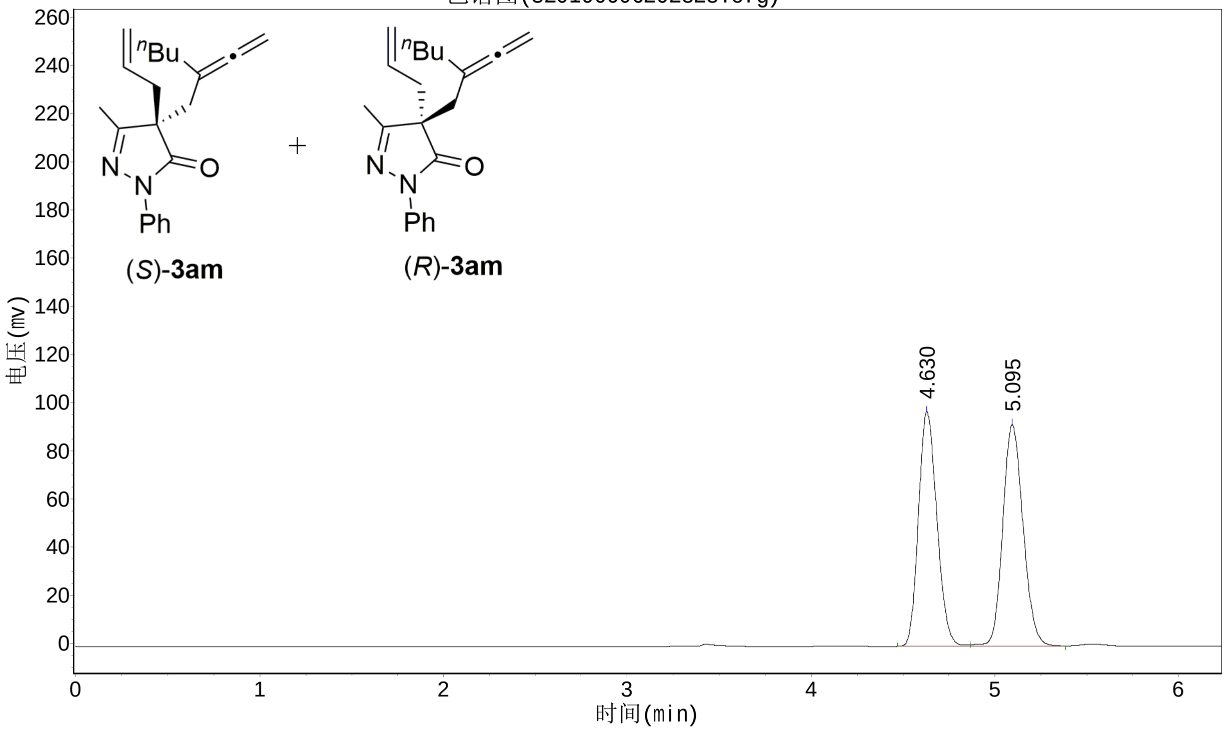

分析结果表

峰号	峰名	保留时间	峰高	峰面积	含量
1		4.630	97395.195	678758.938	48.8714
2		5.095	91996.266	710108.875	51.1286
总计			189391.461	1388867.813	100.0000

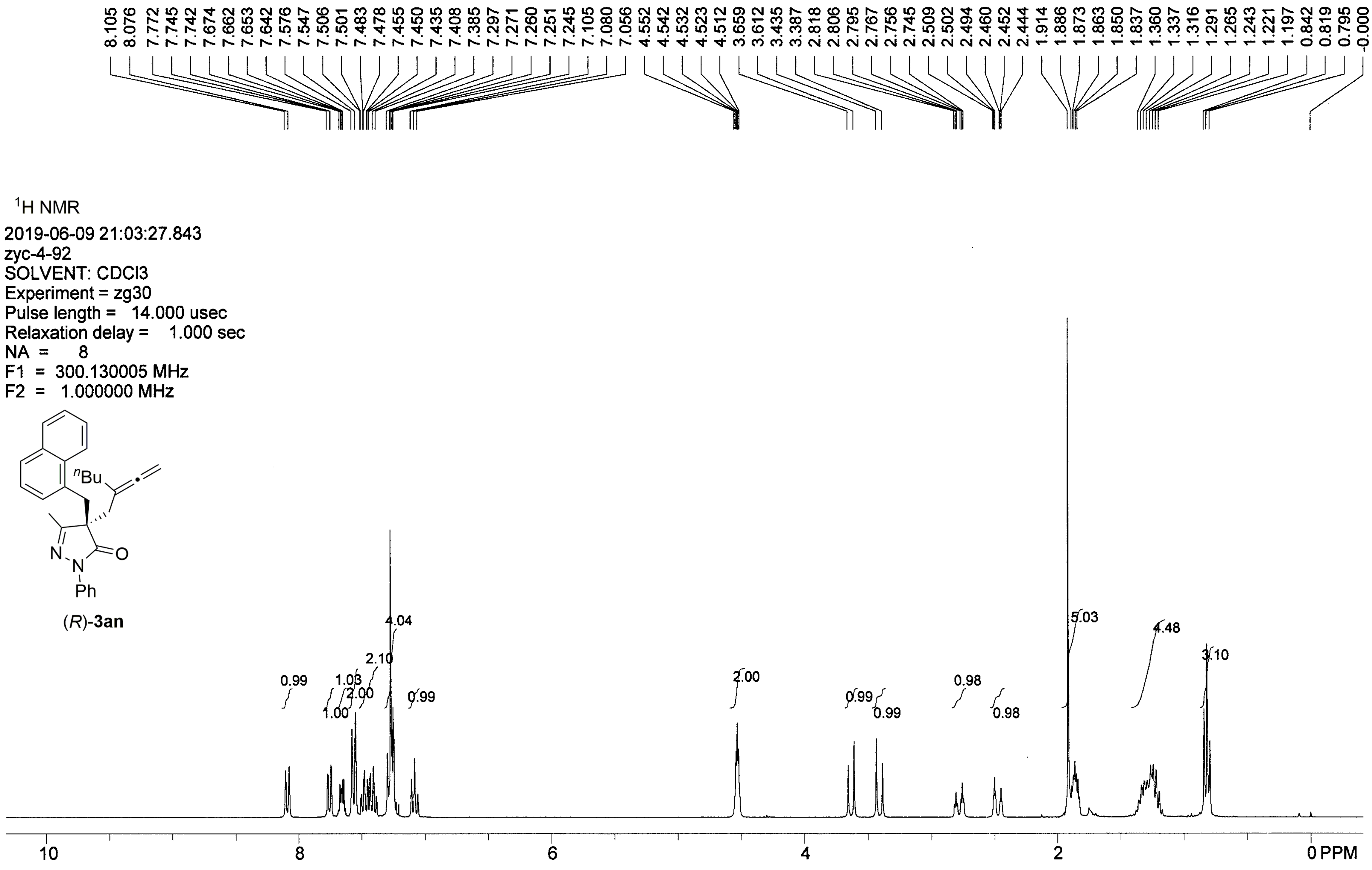
8.105 8.076 7.772 7.745 7.742 7.674 7.662 7.653 7.642 7.576 7.547 7.506 7.501 7.483 7.478 7.455 7.450 7.435 7.408 7.385 7.297 7.271 7.260 7.251 7.245 7.105 7.080 7.056 4.552 4.542 4.532 4.523 4.512 3.659 3.612 3.435 3.387 2.818 2.806 2.795 2.767 2.756 2.745 2.509 2.502 2.494 2.460 2.452 2.444 1.914 1.886 1.873 1.863 1.850 1.837 1.360 1.337 1.316 1.291 1.265 1.243 1.221 1.197 0.842 0.819 0.795 -0.000
¹H NMR
2019-06-09 21:03:27.843
zyc-4-92
SOLVENT: CDCl3
Experiment = zg30
Pulse length = 14.000 usec
Relaxation delay = 1.000 sec
NA = 8
F1 = 300.130005 MHz
F2 = 1.000000 MHz
nBu
N
N
O
Ph
(R)-3an
0.99
1.00
1.03
2.00
2.10
4.04
0.99
2.00
0.99
0.99
0.98
0.98
5.03
4.48
3.10
10
8
6
4
2
0 PPM

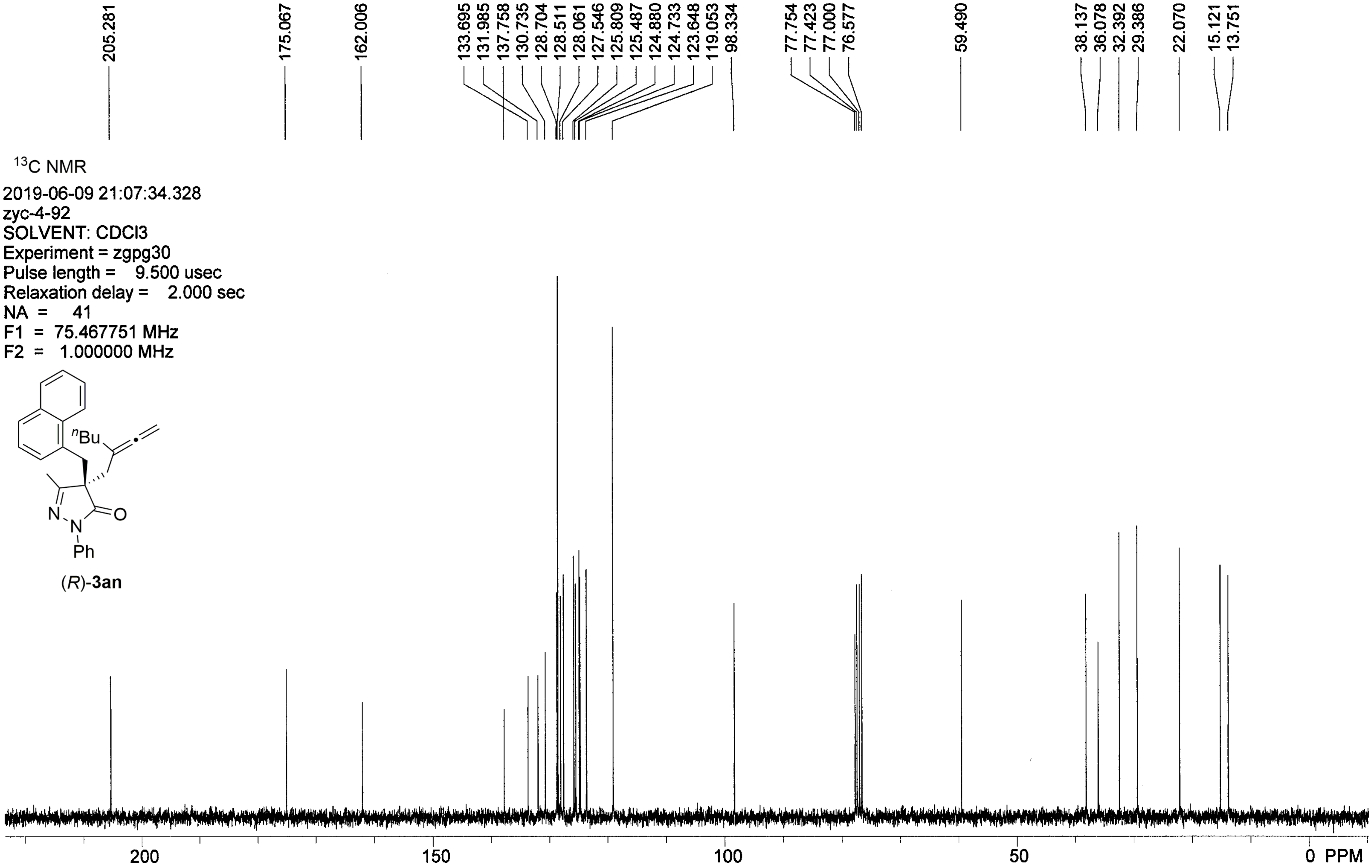

^{13}C NMR

2019-06-09 21:07:34.328
zyc-4-92
SOLVENT: CDCl3
Experiment = zgpg30
Pulse length = 9.500 usec
Relaxation delay = 2.000 sec
NA = 41
F1 = 75.467751 MHz
F2 = 1.000000 MHz

(*R*)-**3an**

zyc-4-92

实验时间：2019-06-10,22:05:21
谱图文件:D:\浙大智达\N2000\样品\S20190610220521.org
方法文件:D:\浙大智达\N2000\djx.mtd

实验者：zyc
报告时间：2019-06-10,22:19:22
积分方法:面积归一法

实验内容简介：
ia,n-hexane/i-PrOH = 90/10,1.0,254

色谱图(S20190610220521.org)

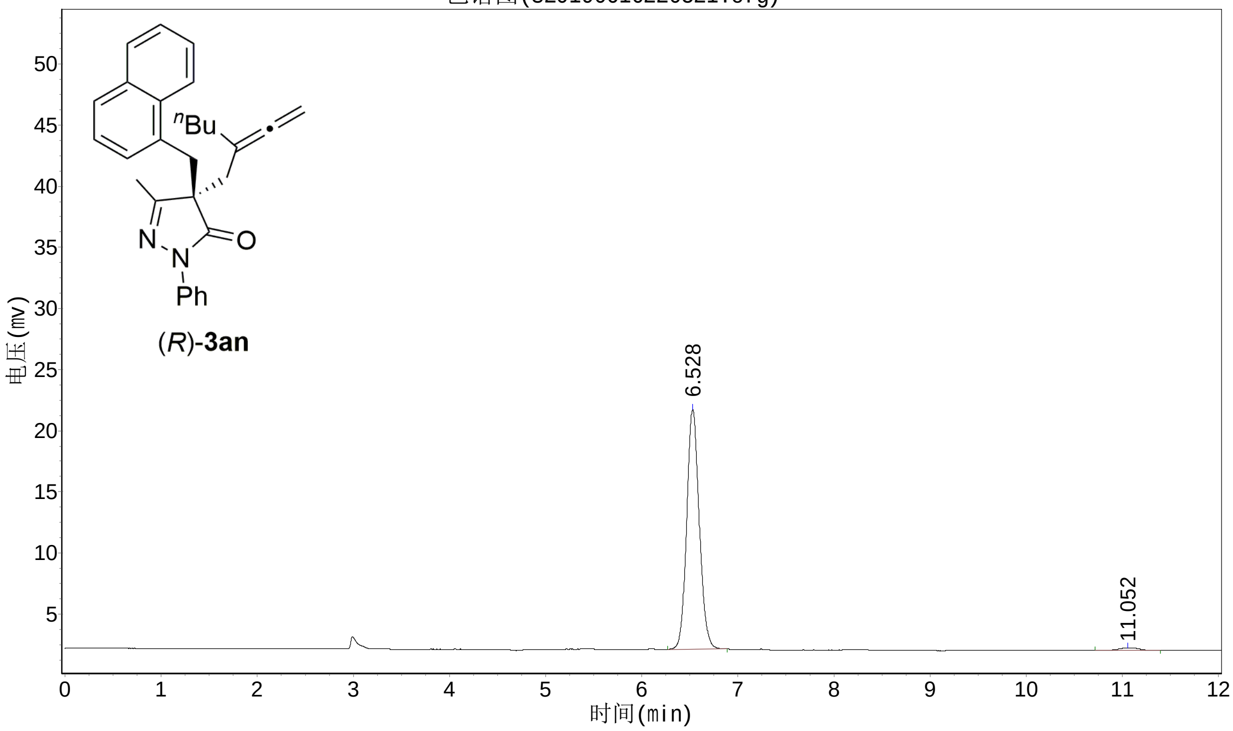

分析结果表

峰号	峰名	保留时间	峰高	峰面积	含量
1		6.528	19622.469	178493.000	98.3511
2		11.052	206.517	2992.599	1.6489
总计			19828.986	181485.599	100.0000

zyc-4-92mix

实验时间：2019-06-10,21:33:59
谱图文件:D:\浙大智达\N2000\样品\S20190610213359.org
方法文件:D:\浙大智达\N2000\djx.mtd

实验者：zyc
报告时间：2019-06-10,21:56:54
积分方法:面积归一法

实验内容简介：
ia,n-hexane/i-PrOH = 90/10,1.0,254

色谱图(S20190610213359.org)

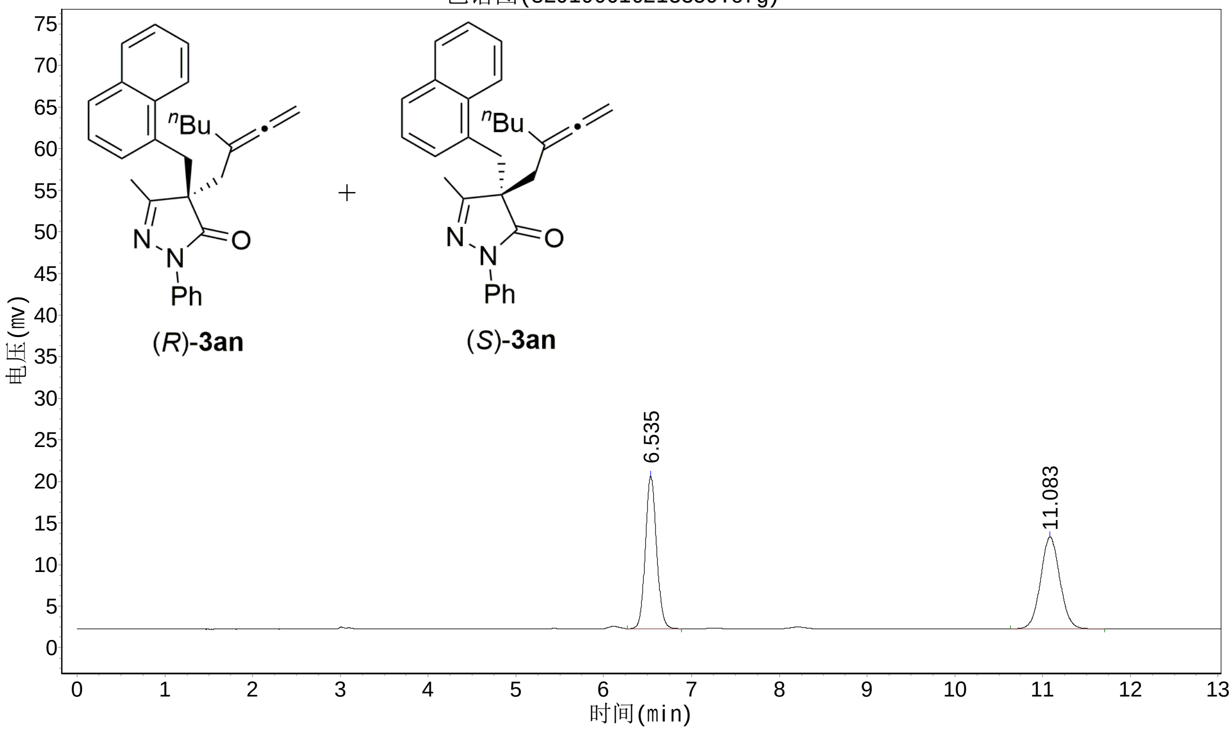

分析结果表

峰号	峰名	保留时间	峰高	峰面积	含量
1		6.535	18318.156	162300.406	49.0179
2		11.083	11079.329	168803.906	50.9821
总计			29397.485	331104.313	100.0000

^{1}H NMR
2019-07-09 12:51:58.562
zyc-4-126
SOLVENT: CDCl3
Experiment = zg30
Pulse length = 14.000 usec
Relaxation delay = 1.000 sec
NA = 8
F1 = 300.130005 MHz
F2 = 1.000000 MHz

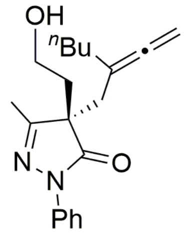

(*R*)-**3ao**

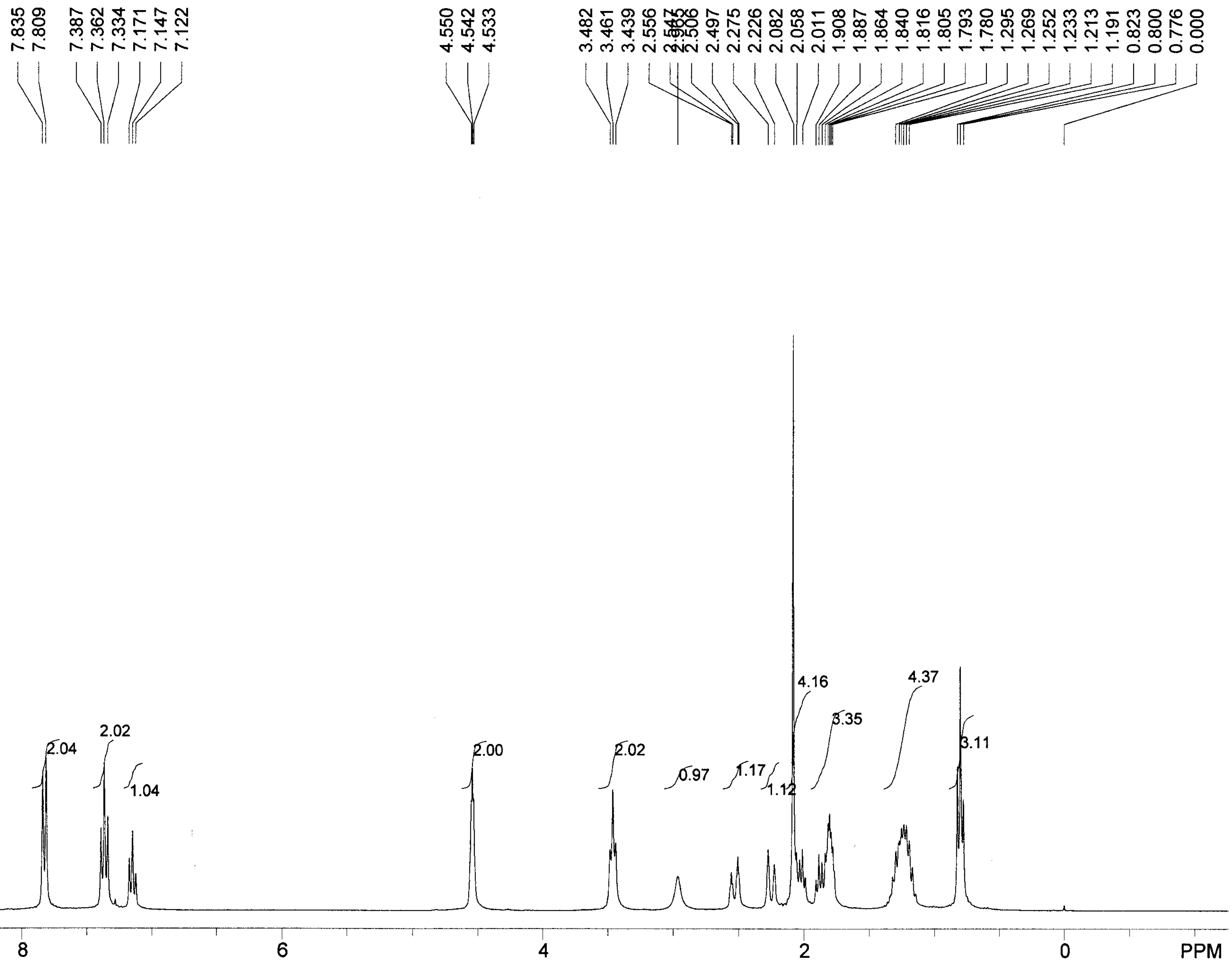

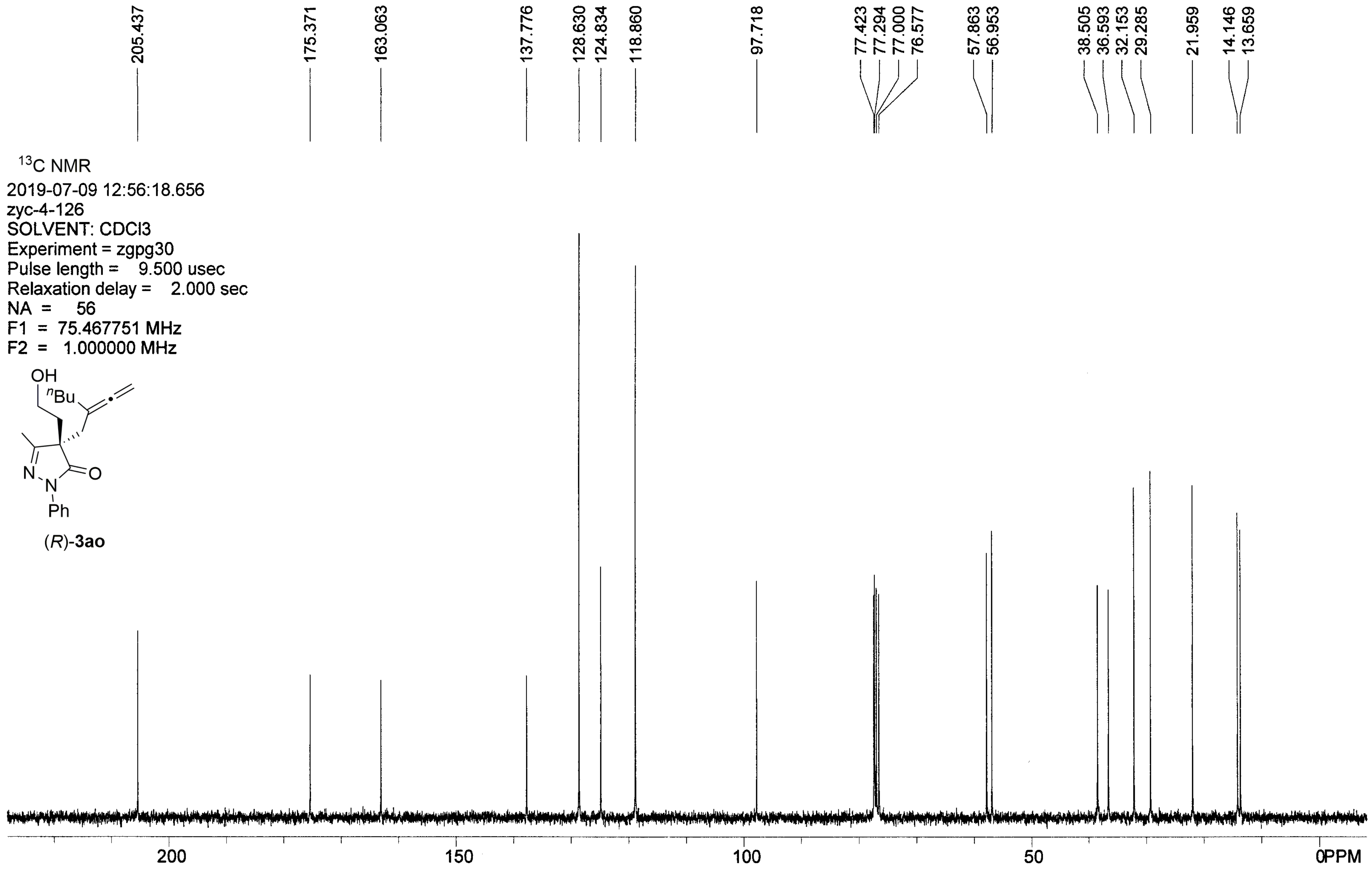
205.437
175.371
163.063
137.776
128.630
124.834
118.860
97.718
77.423
77.294
77.000
76.577
57.863
56.953
38.505
36.593
32.153
29.285
21.959
14.146
13.659
^{13}C NMR
2019-07-09 12:56:18.656
zyc-4-126
SOLVENT: CDCl3
Experiment = zgpg30
Pulse length = 9.500 usec
Relaxation delay = 2.000 sec
NA = 56
F1 = 75.467751 MHz
F2 = 1.000000 MHz
OH
^{n}Bu
N
N
O
Ph
(*R*)-**3ao**
200
150
100
50
0PPM

zyc-4-126

实验时间：2019-07-09,21:42:51
谱图文件:D:\浙大智达\N2000\样品\S20190709214251.org
方法文件:D:\浙大智达\N2000\djx.mtd

实验者：zyc
报告时间：2019-07-09,21:57:42
积分方法:面积归一法

实验内容简介：
od,n-hexane/i-PrOH = 90/10,1.0,254

色谱图(S20190709214251.org)

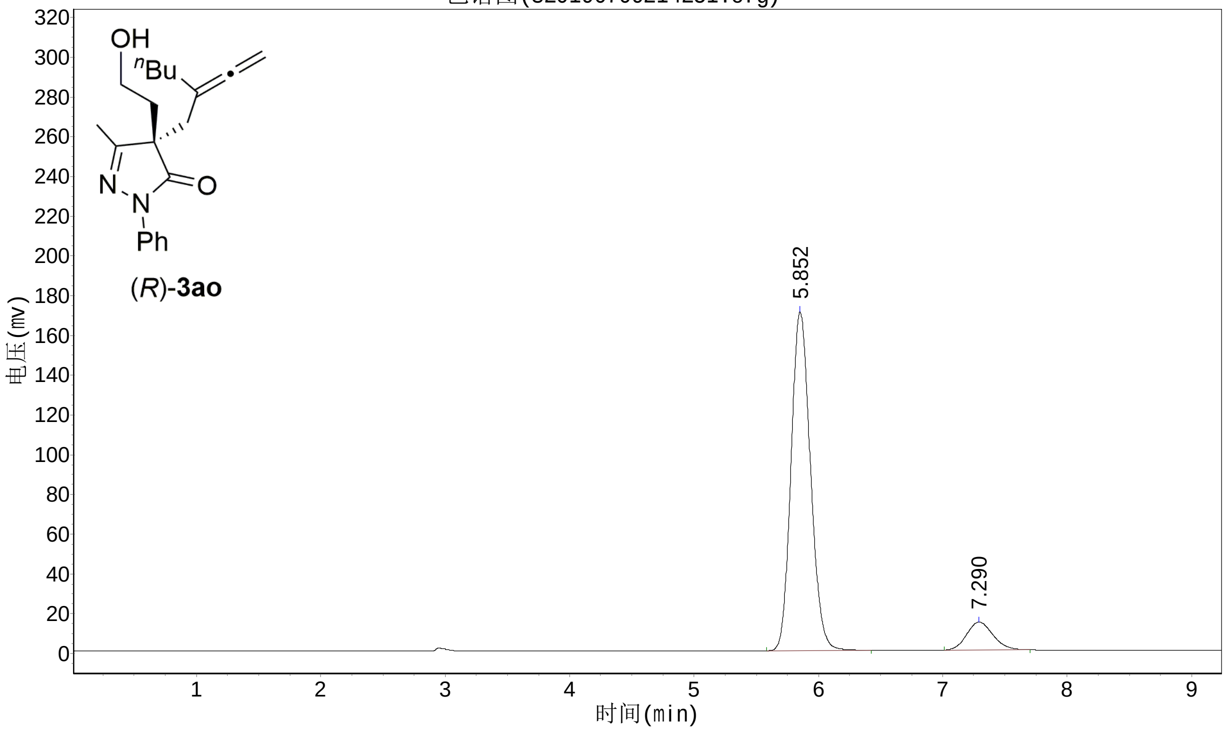

分析结果表

峰号	峰名	保留时间	峰高	峰面积	含量
1		5.852	170293.203	1839755.250	90.0188
2		7.290	13948.662	203991.203	9.9812
总计			184241.865	2043746.453	100.0000

zyc-4-126mix

实验时间：2019-07-09,21:56:32
谱图文件:D:\浙大智达\N2000\样品\S20190709215632.org
方法文件:D:\浙大智达\N2000\djx.mtd

实验者：zyc
报告时间：2019-07-09,22:09:22
积分方法:面积归一法

实验内容简介：
od,n-hexane/i-PrOH = 90/10,1.0,254

色谱图(S20190709215632.org)

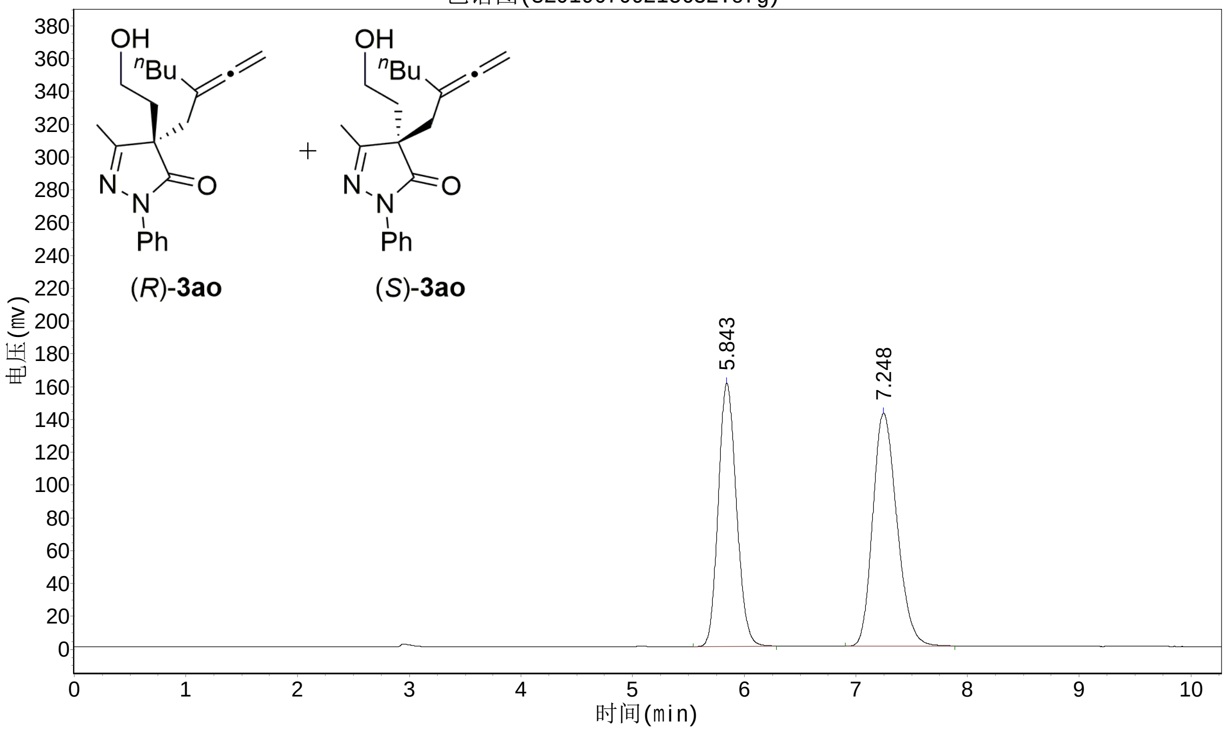

分析结果表

峰号	峰名	保留时间	峰高	峰面积	含量
1		5.843	160429.359	1806947.625	45.7591
2		7.248	142194.328	2141882.000	54.2409
总计			302623.688	3948829.625	100.0000

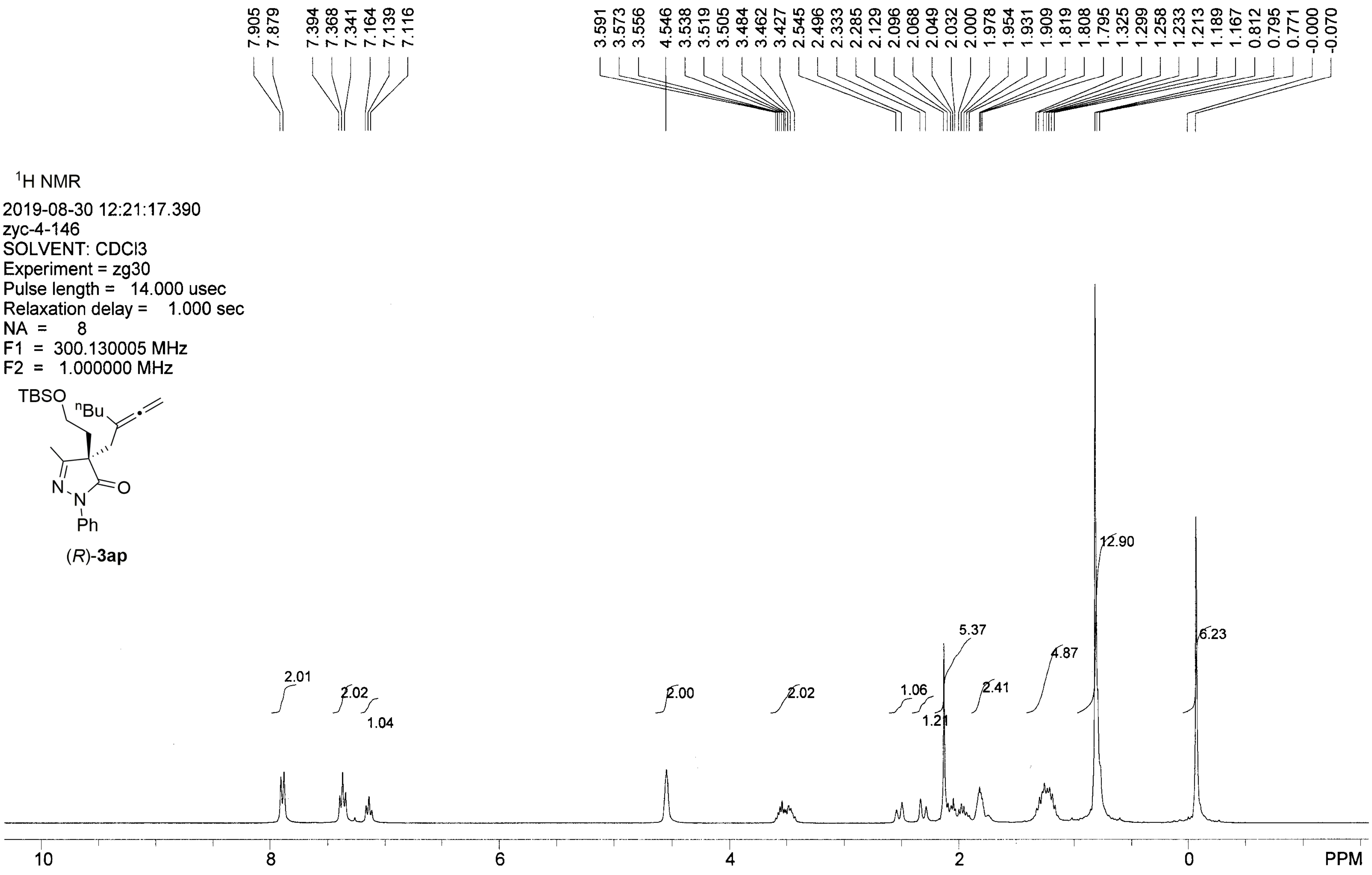

^{1}H NMR

2019-08-30 12:21:17.390
zyc-4-146
SOLVENT: CDCl3
Experiment = zg30
Pulse length = 14.000 usec
Relaxation delay = 1.000 sec
NA = 8
F1 = 300.130005 MHz
F2 = 1.000000 MHz

(*R*)-**3ap**

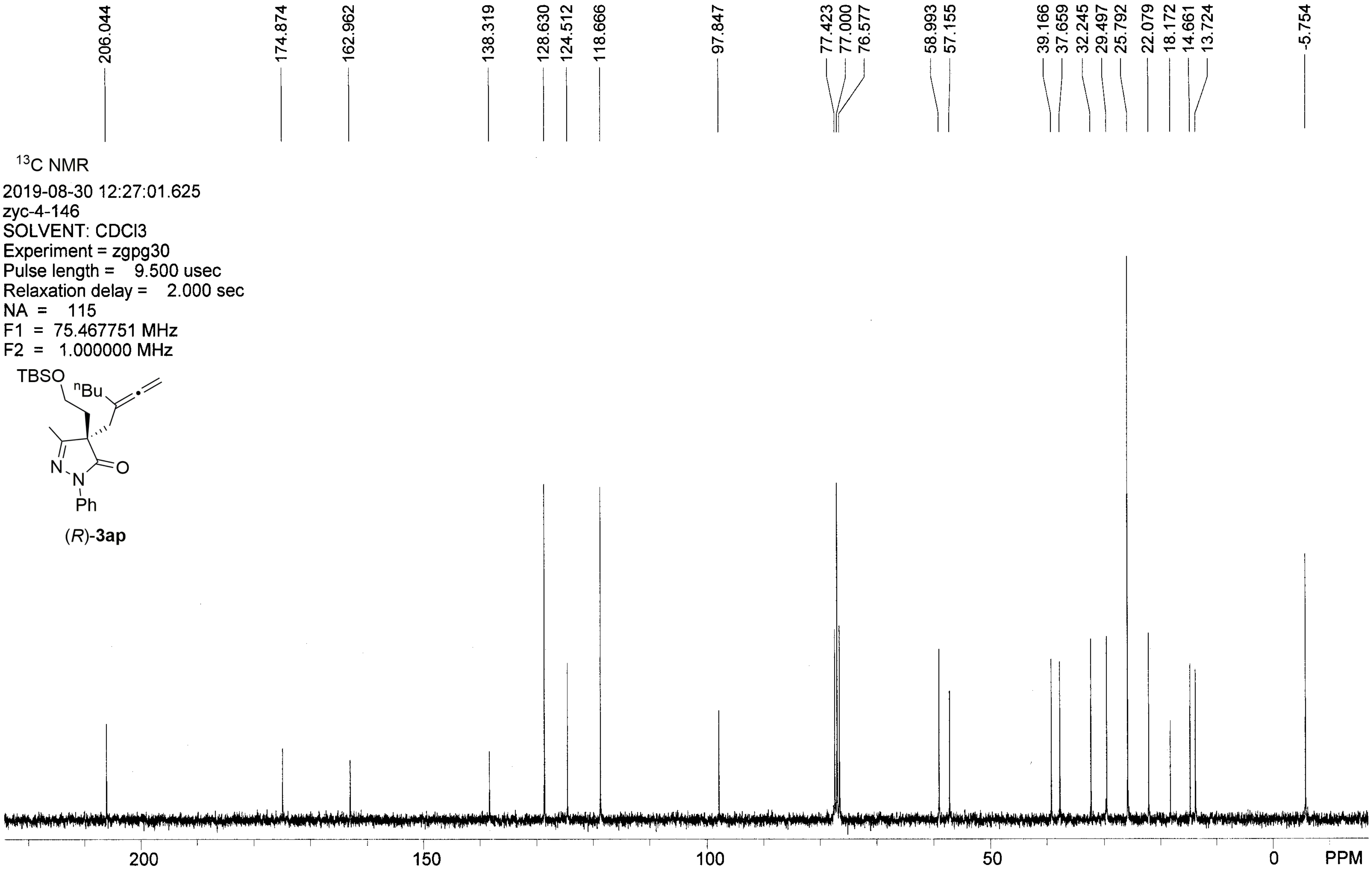

^{13}C NMR
2019-08-30 12:27:01.625
zyc-4-146
SOLVENT: CDCl3
Experiment = zgpg30
Pulse length = 9.500 usec
Relaxation delay = 2.000 sec
NA = 115
F1 = 75.467751 MHz
F2 = 1.000000 MHz

(*R*)-**3ap**

zyc-4-146

实验时间：2019-08-30,10:32:10
谱图文件:D:\浙大智达\N2000\样品\S20190830103210.org
方法文件:D:\浙大智达\N2000\djx.mtd

实验者：zyc
报告时间：2019-08-30,10:45:13
积分方法:面积归一法

实验内容简介：
ia,n-hexane/i-PrOH = 95/5,1.0,254

色谱图(S20190830103210.org)

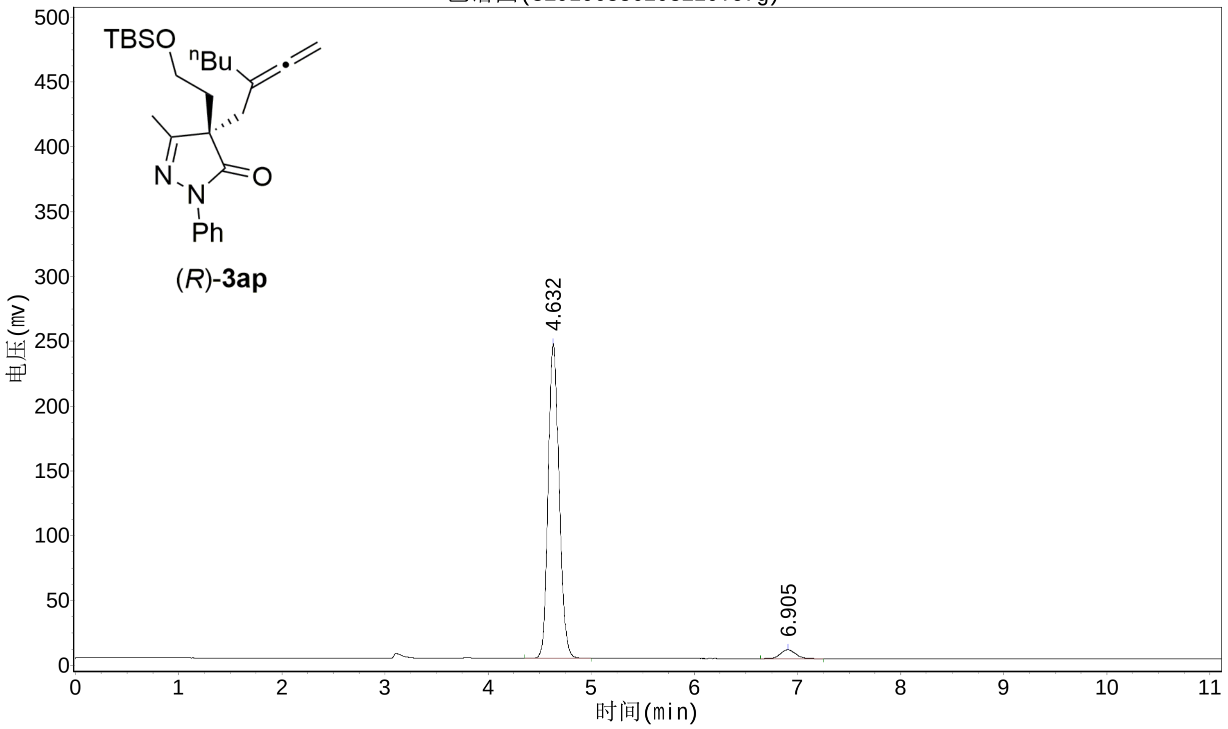

分析结果表

峰号	峰名	保留时间	峰高	峰面积	含量
1		4.632	242558.375	1792585.375	96.3371
2		6.905	6806.700	68157.250	3.6629
总计			249365.075	1860742.625	100.0000

zyc-4-146mix

实验时间：2019-08-30,11:11:42
谱图文件:D:\浙大智达\N2000\样品\S20190830111142.org
方法文件:D:\浙大智达\N2000\djx.mtd

实验者：zyc
报告时间：2019-08-30,11:31:45
积分方法:面积归一法

实验内容简介：
ia,n-hexane/i-PrOH = 95/5,1.0,254

色谱图(S20190830111142.org)

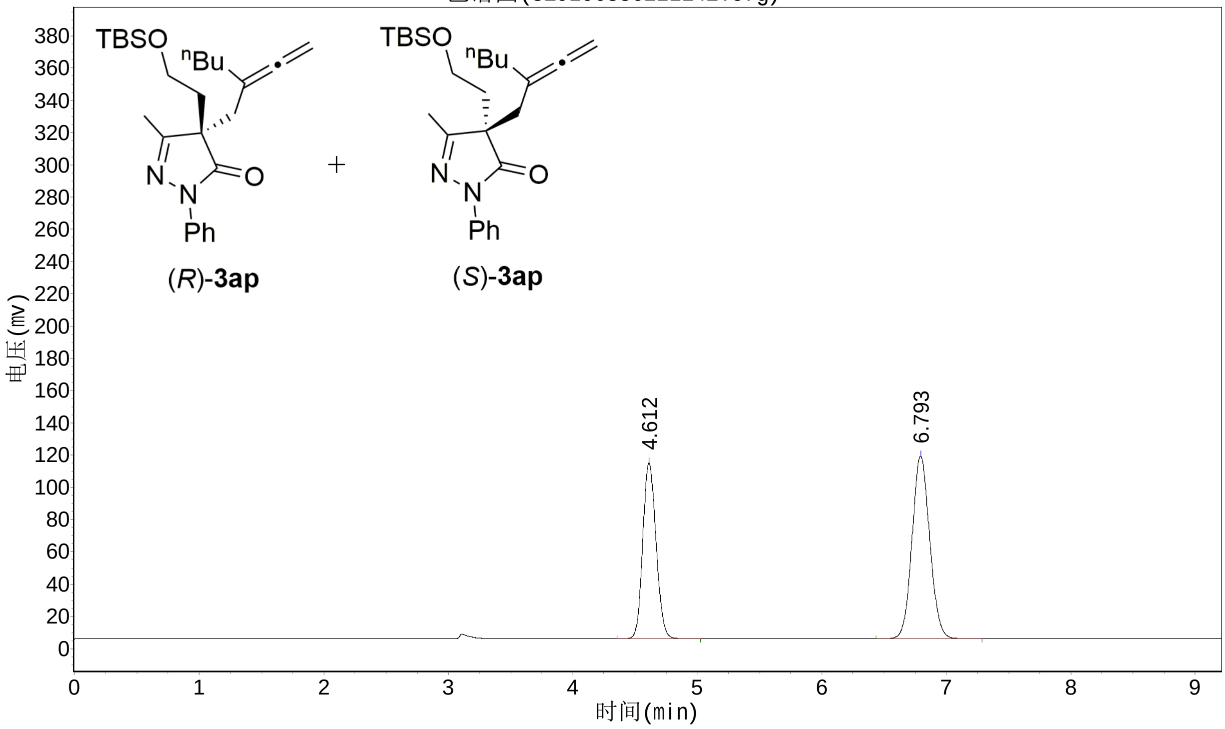

分析结果表

峰号	峰名	保留时间	峰高	峰面积	含量
1		4.612	109236.977	812670.625	41.9686
2		6.793	113315.180	1123706.500	58.0314
总计			222552.156	1936377.125	100.0000

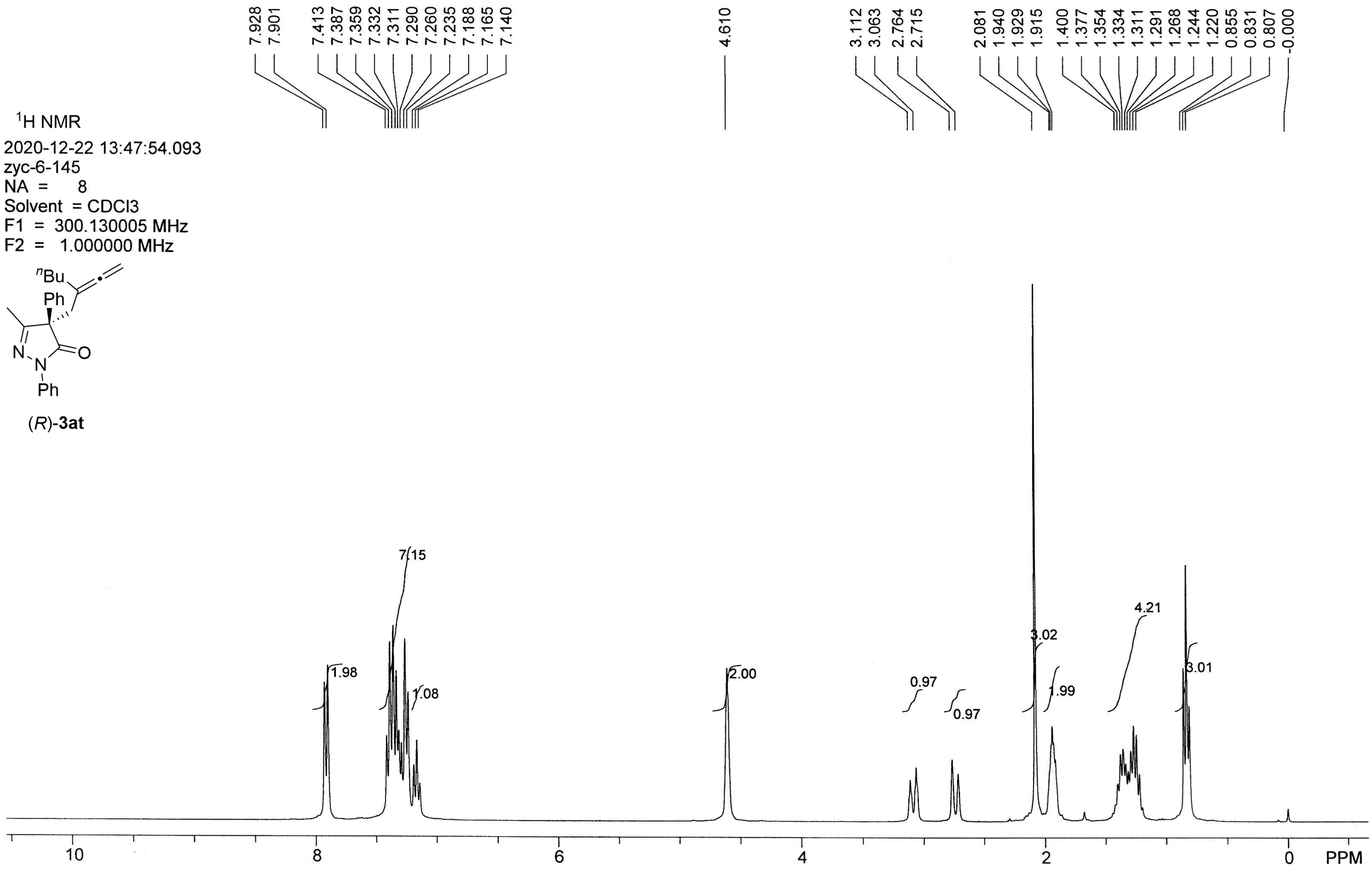

^{1}H NMR
2020-12-22 13:47:54.093
zyc-6-145
NA = 8
Solvent = CDCl3
F1 = 300.130005 MHz
F2 = 1.000000 MHz
^{n}Bu
Ph
N
N
O
Ph
7.928
7.901
7.413
7.387
7.359
7.332
7.311
7.290
7.260
7.235
7.188
7.165
7.140
4.610
3.112
3.063
2.764
2.715
2.081
1.940
1.929
1.915
1.400
1.377
1.354
1.334
1.311
1.291
1.268
1.244
1.220
0.855
0.831
0.807
-0.000
1.98
7.15
1.08
2.00
0.97
0.97
3.02
1.99
4.21
3.01
10
8
6
4
2
0
PPM

(*R*)-**3at**

^{13}C NMR
2020-12-22 09:14:45.250
zyc-6-145
NA = 137
Solvent = CDCl3
F1 = 75.467751 MHz
F2 = 1.000000 MHz

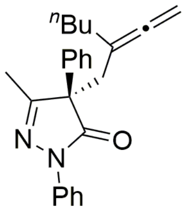

(*R*)-**3at**

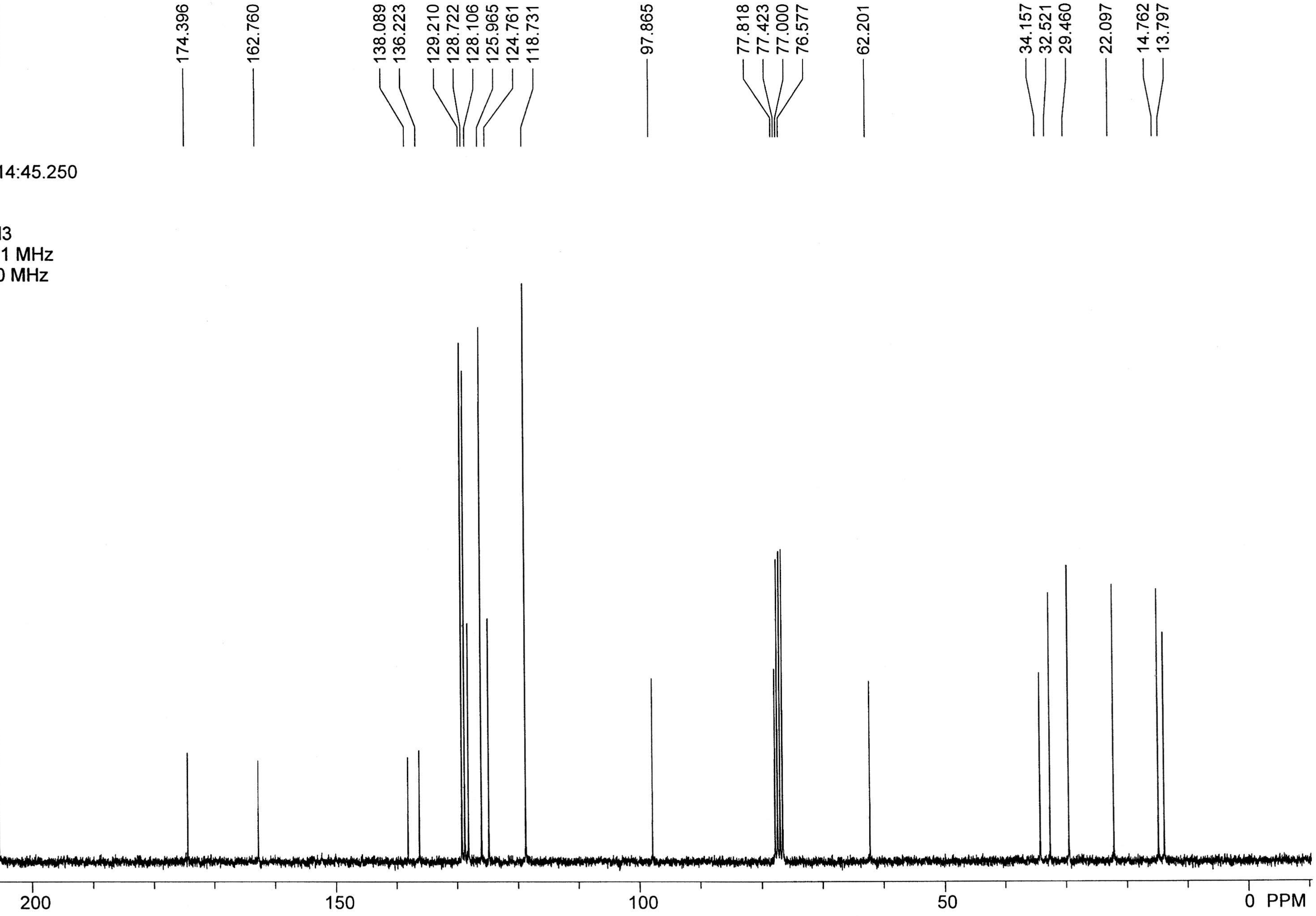

zyc-6-145

实验时间：2020-12-22,16:43:33
谱图文件:D:\浙大智达\N2000\样品\S20201222164333.org
方法文件:D:\浙大智达\N2000\djx.mtd

实验者：zyc
报告时间：2020-12-22,17:51:41
积分方法:面积归一法

实验内容简介：
ia,n-hexane/i-PrOH = 90/10,1.0,254

色谱图(S20201222164333.org)

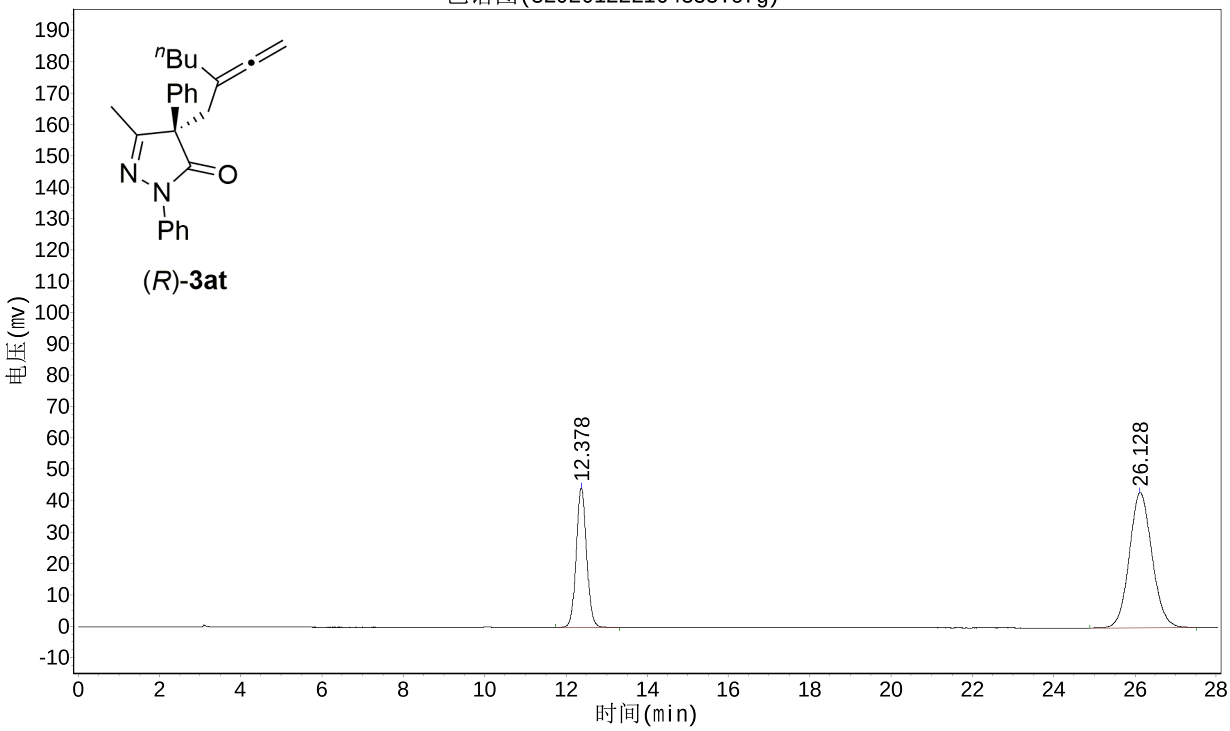

分析结果表

峰号	峰名	保留时间	峰高	峰面积	含量
1		12.378	44343.410	786986.750	32.0023
2		26.128	43023.926	1672170.375	67.9977
总计			87367.336	2459157.125	100.0000

zyc-6-145mix

实验时间：2020-12-22,17:12:13
谱图文件:D:\浙大智达\N2000\样品\S20201222171213.org
方法文件:D:\浙大智达\N2000\djx.mtd

实验者：zyc
报告时间：2020-12-22,17:52:35
积分方法:面积归一法

实验内容简介：
ia,n-hexane/i-PrOH = 90/10,1.0,254

色谱图(S20201222171213.org)

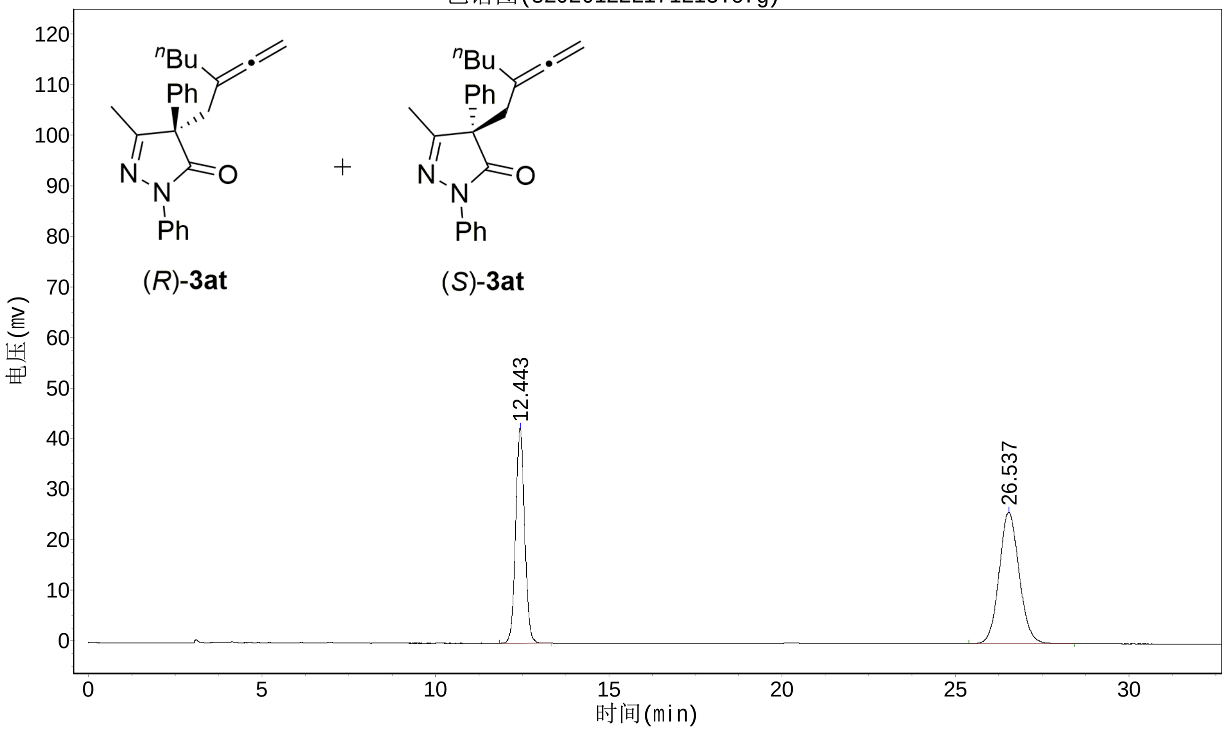

分析结果表

峰号	峰名	保留时间	峰高	峰面积	含量
1		12.443	42509.164	765909.625	42.3294
2		26.537	25965.709	1043495.000	57.6706
总计			68474.873	1809404.625	100.0000

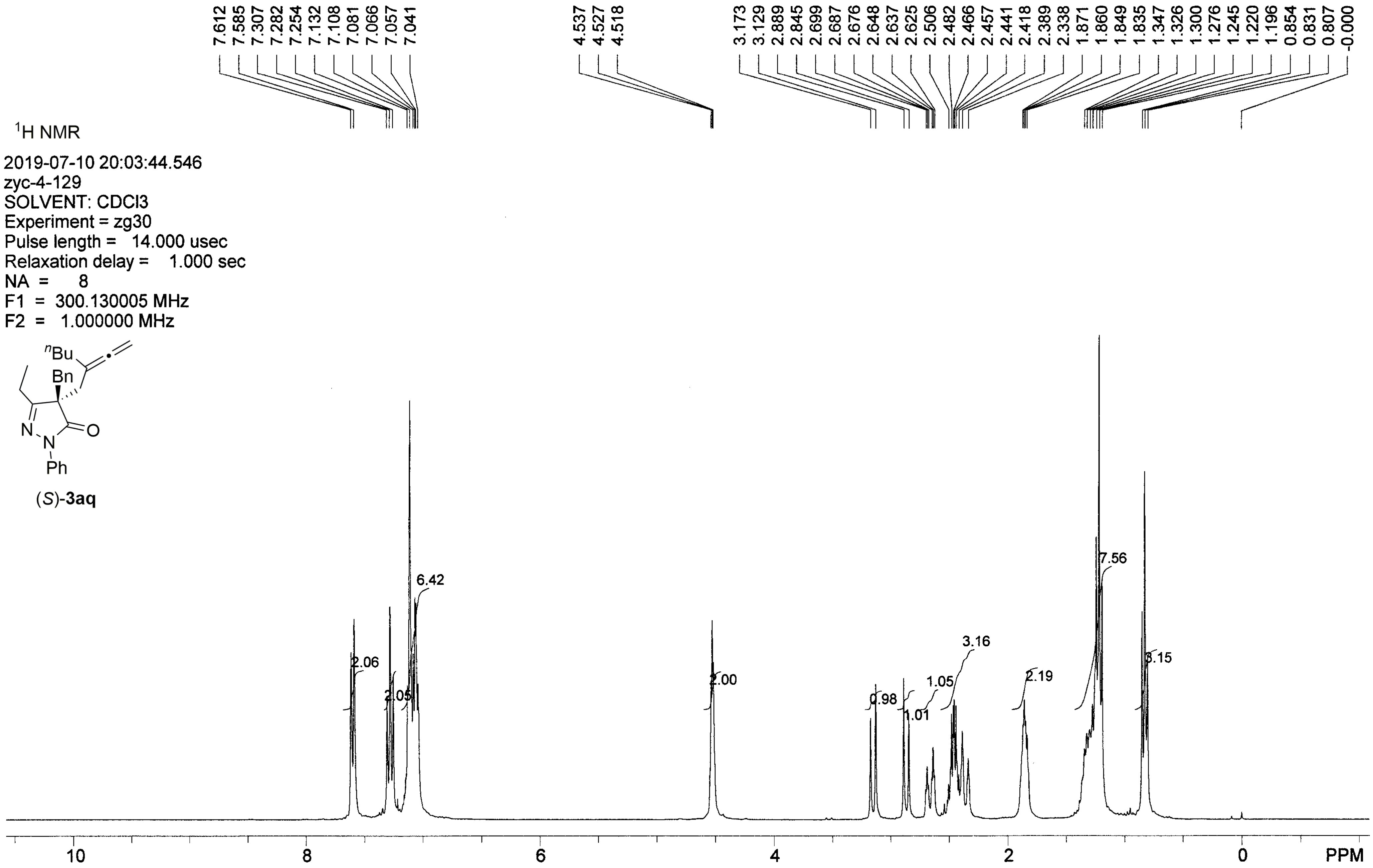
1H NMR
2019-07-10 20:03:44.546
zyc-4-129
SOLVENT: CDCl3
Experiment = zg30
Pulse length = 14.000 usec
Relaxation delay = 1.000 sec
NA = 8
F1 = 300.130005 MHz
F2 = 1.000000 MHz
nBu
Bn
N
N
O
Ph
(S)-3aq
7.612
7.585
7.307
7.282
7.254
7.132
7.108
7.081
7.066
7.057
7.041
4.537
4.527
4.518
3.173
3.129
2.889
2.845
2.699
2.687
2.676
2.648
2.637
2.625
2.506
2.482
2.466
2.457
2.441
2.418
2.389
2.338
1.871
1.860
1.849
1.835
1.347
1.326
1.300
1.276
1.245
1.220
1.196
0.854
0.831
0.807
-0.000
2.06
2.05
6.42
2.00
0.98
1.01
1.05
3.16
2.19
7.56
3.15
10
8
6
4
2
0
PPM

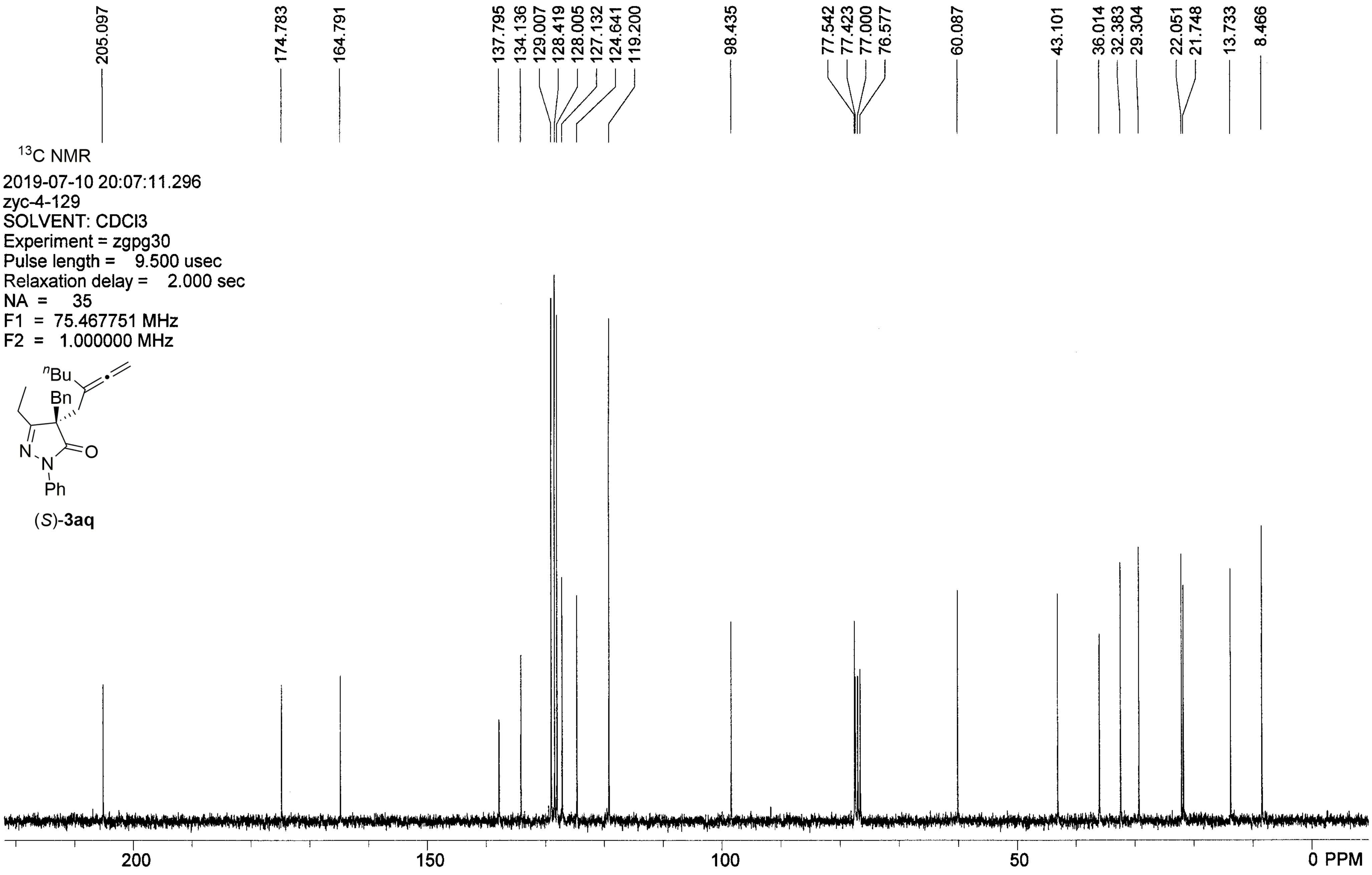
205.097
174.783
164.791
137.795
134.136
129.007
128.419
128.005
127.132
124.641
119.200
98.435
77.542
77.423
77.000
76.577
60.087
43.101
36.014
32.383
29.304
22.051
21.748
13.733
8.466
13C NMR
2019-07-10 20:07:11.296
zyc-4-129
SOLVENT: CDCl3
Experiment = zgpg30
Pulse length = 9.500 usec
Relaxation delay = 2.000 sec
NA = 35
F1 = 75.467751 MHz
F2 = 1.000000 MHz
nBu
Bn
N
N
O
Ph
200
150
100
50
0 PPM
(S)-**3aq**

zyc-4-129

实验时间：2019-07-10,9:35:25
谱图文件:D:\浙大智达\N2000\样品\S20190710093525.org
方法文件:D:\浙大智达\N2000\djx.mtd

实验者：zyc
报告时间：2019-07-10,10:02:59
积分方法:面积归一法

实验内容简介：
ia,n-hexane/i-PrOH = 90/10,1.0,254

色谱图(S20190710093525.org)

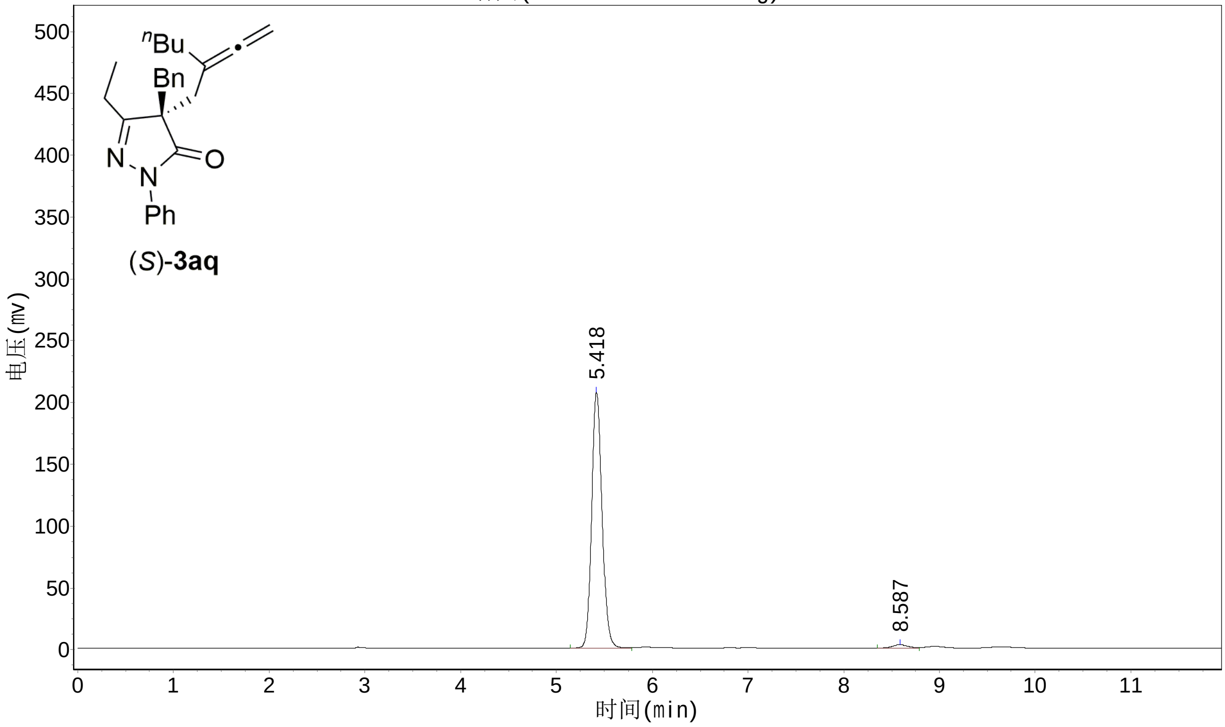

分析结果表

峰号	峰名	保留时间	峰高	峰面积	含量
1		5.418	206521.313	1556628.875	98.1114
2		8.587	2605.543	29964.865	1.8886
总计			209126.855	1586593.740	100.0000

zyc-4-129mix

实验时间：2019-07-10,10:05:16
谱图文件:D:\浙大智达\N2000\样品\S20190710100516.org
方法文件:D:\浙大智达\N2000\djx.mtd

实验者：zyc
报告时间：2019-07-10,10:18:33
积分方法:面积归一法

实验内容简介：
ia,n-hexane/i-PrOH = 90/10,1.0,254

色谱图(S20190710100516.org)

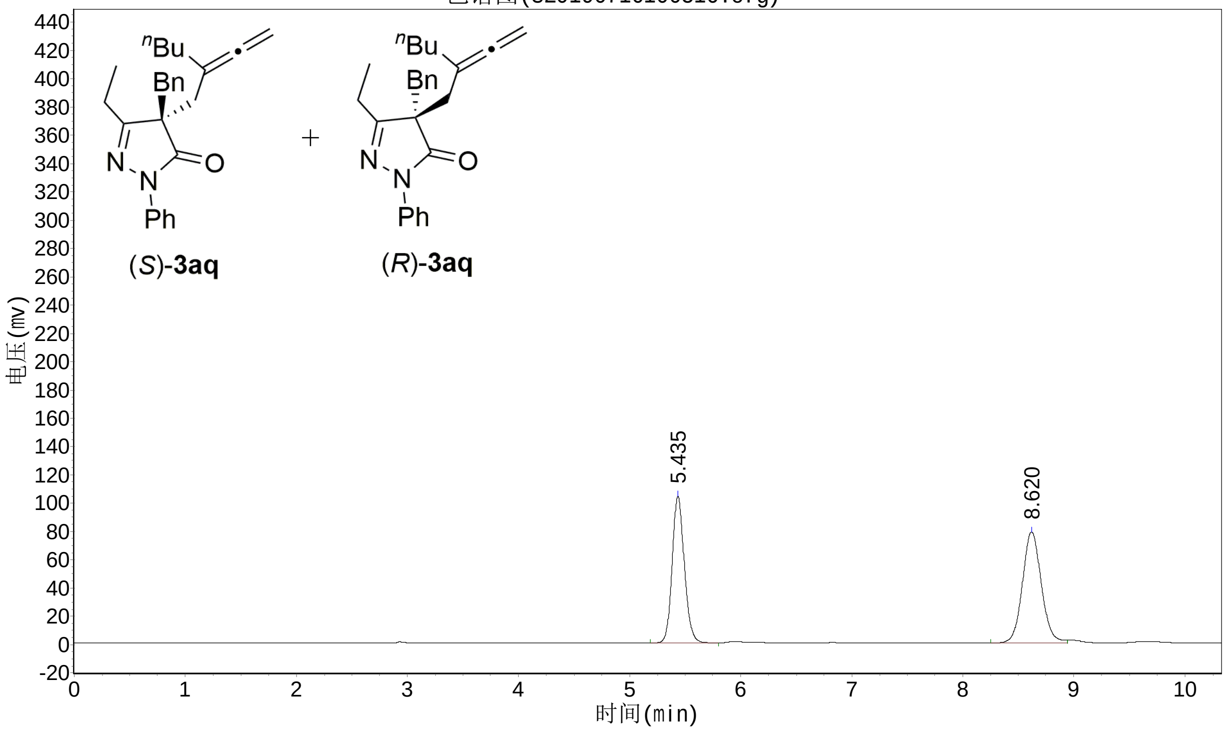

分析结果表

峰号	峰名	保留时间	峰高	峰面积	含量
1		5.435	103663.898	789314.375	46.0052
2		8.620	78365.828	926394.000	53.9948
总计			182029.727	1715708.375	100.0000

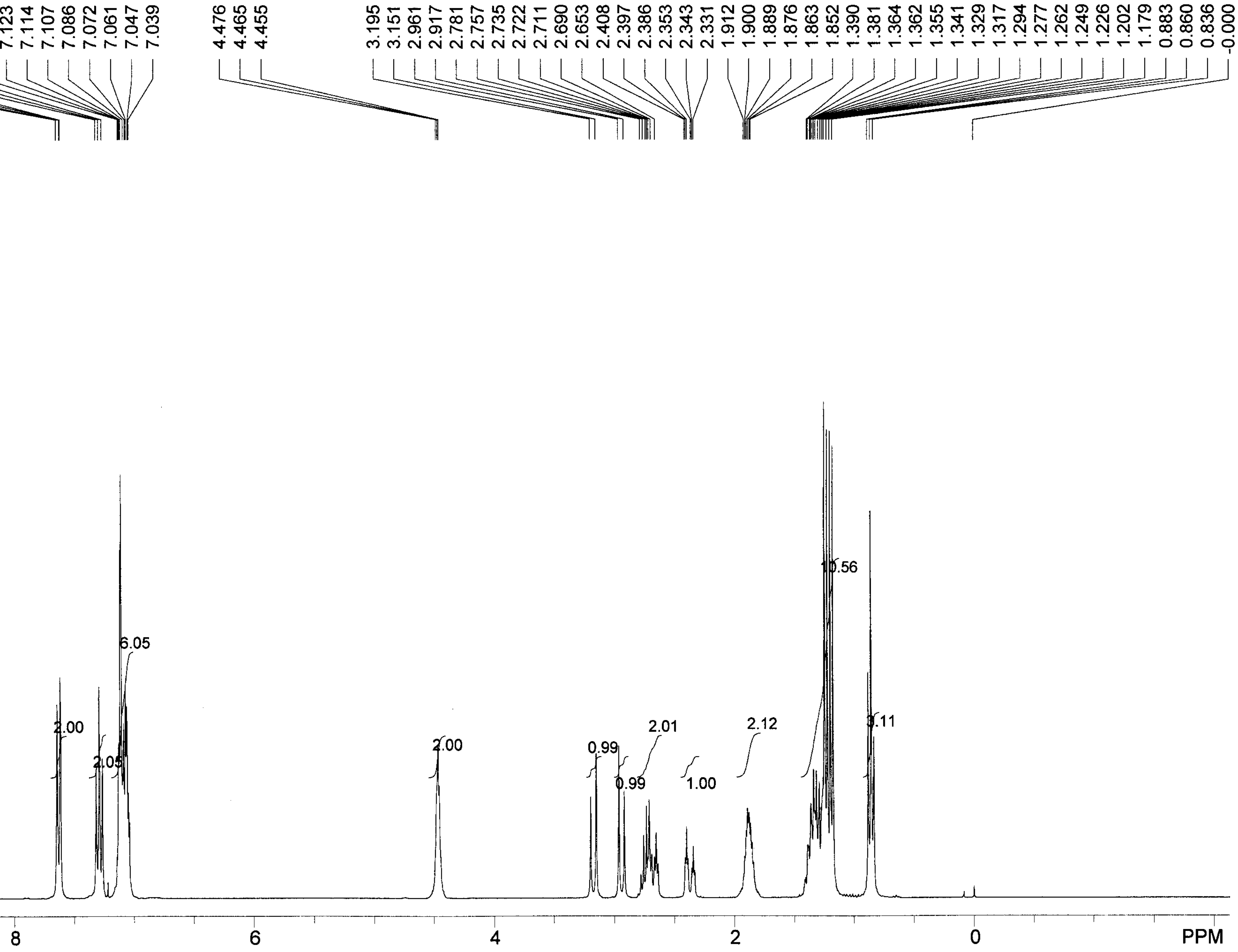

^{1}H NMR

2019-07-18 10:04:16.812
zyc-4-141
SOLVENT: CDCl3
Experiment = zg30
Pulse length = 14.000 usec
Relaxation delay = 1.000 sec
NA = 8
F1 = 300.130005 MHz
F2 = 1.000000 MHz

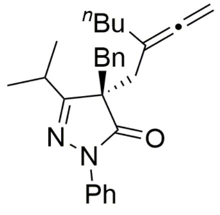

(*S*)-**3ar**

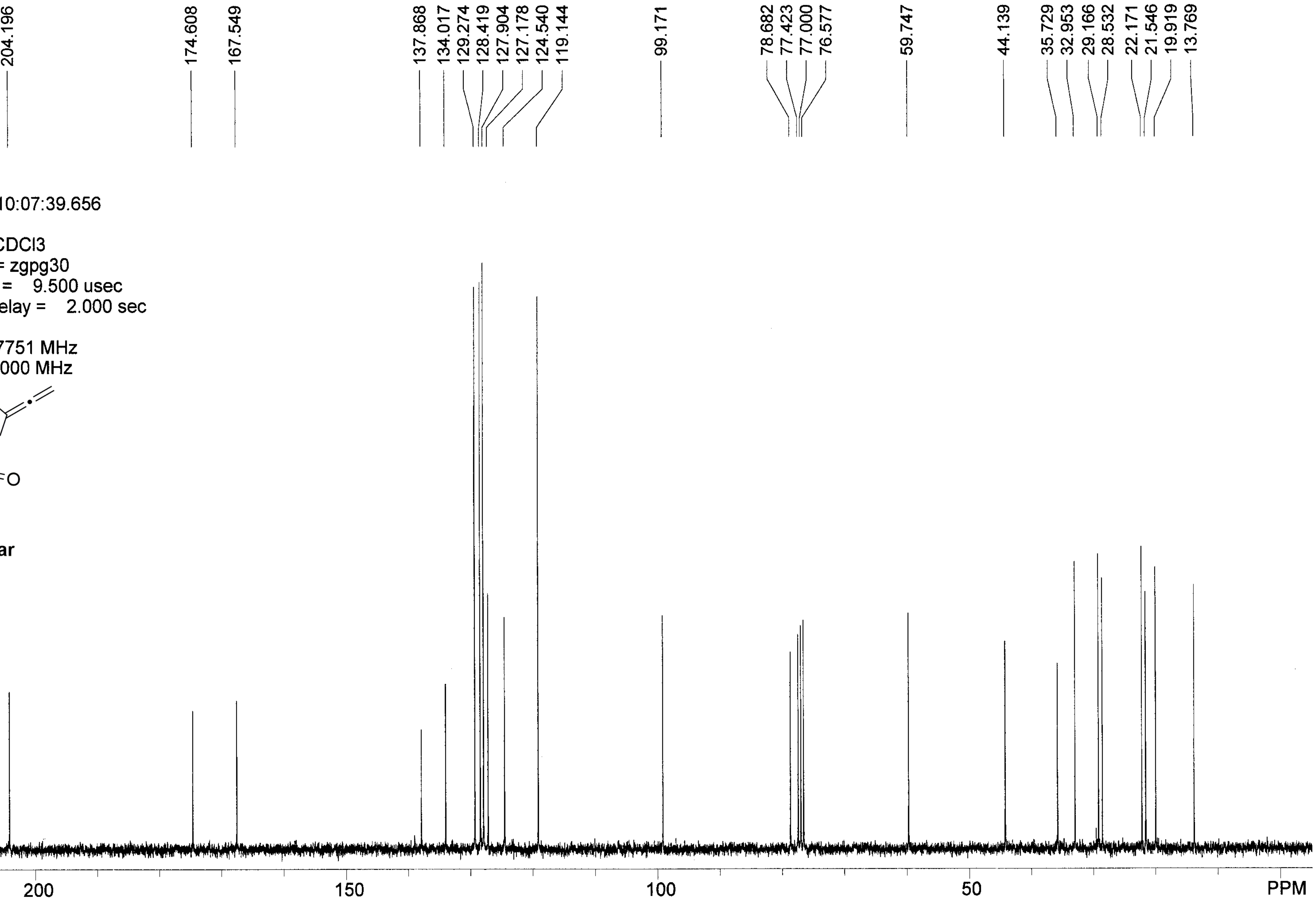

^{13}C NMR

2019-07-18 10:07:39.656
zyc-4-141
SOLVENT: CDCl3
Experiment = zgpg30
Pulse length = 9.500 usec
Relaxation delay = 2.000 sec
NA = 38
F1 = 75.467751 MHz
F2 = 1.000000 MHz

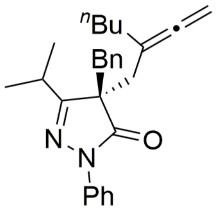

(*S*)-**3ar**

zyc-4-141

实验时间：2019-07-17,21:43:57
谱图文件:D:\浙大智达\N2000\样品\S20190717214357.org
方法文件:D:\浙大智达\N2000\djx.mtd

实验者：zyc
报告时间：2019-07-17,22:07:00
积分方法:面积归一法

实验内容简介：
ia,n-hexane/i-PrOH = 90/10,1.0,254

色谱图(S20190717214357.org)

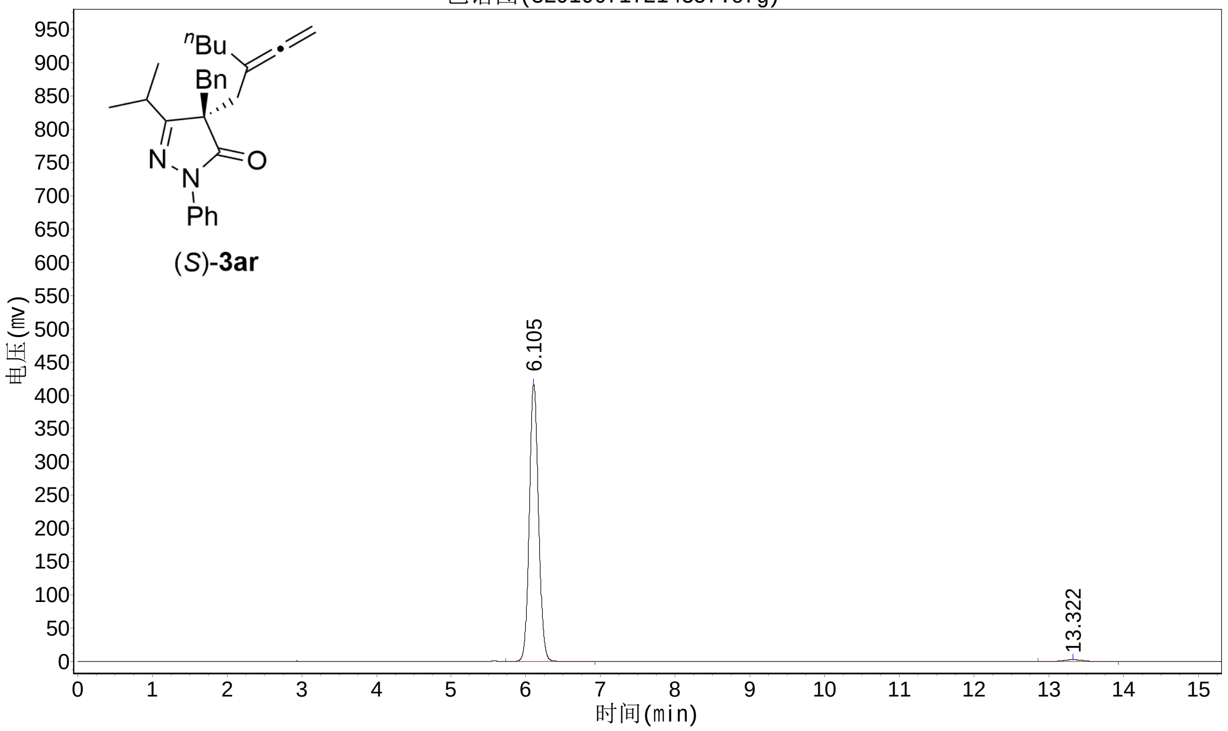

分析结果表

峰号	峰名	保留时间	峰高	峰面积	含量
1		6.105	416669.969	3572972.000	98.4148
2		13.322	3050.423	57552.598	1.5852
总计			419720.392	3630524.598	100.0000

zyc-4-141mix

实验时间：2019-07-17,22:00:14　　实验者：zyc
谱图文件:D:\浙大智达\N2000\样品\S20190718085028.org　　报告时间：2019-07-18,9:16:57
方法文件:D:\浙大智达\N2000\djx.mtd　　积分方法:面积归一法

实验内容简介：
ia,n-hexane/i-PrOH = 90/10,1.0,254

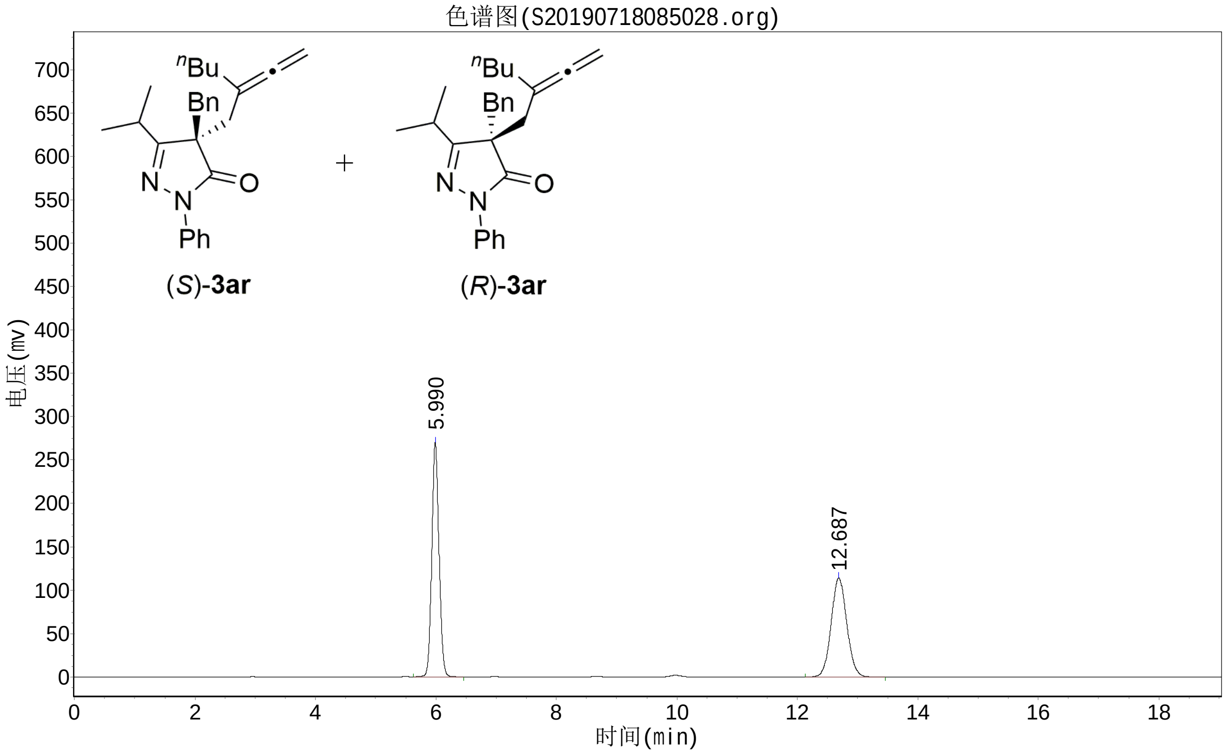

分析结果表

峰号	峰名	保留时间	峰高	峰面积	含量
1		5.990	270197.375	2260187.250	52.2578
2		12.687	114249.672	2064885.375	47.7422
总计			384447.047	4325072.625	100.0000

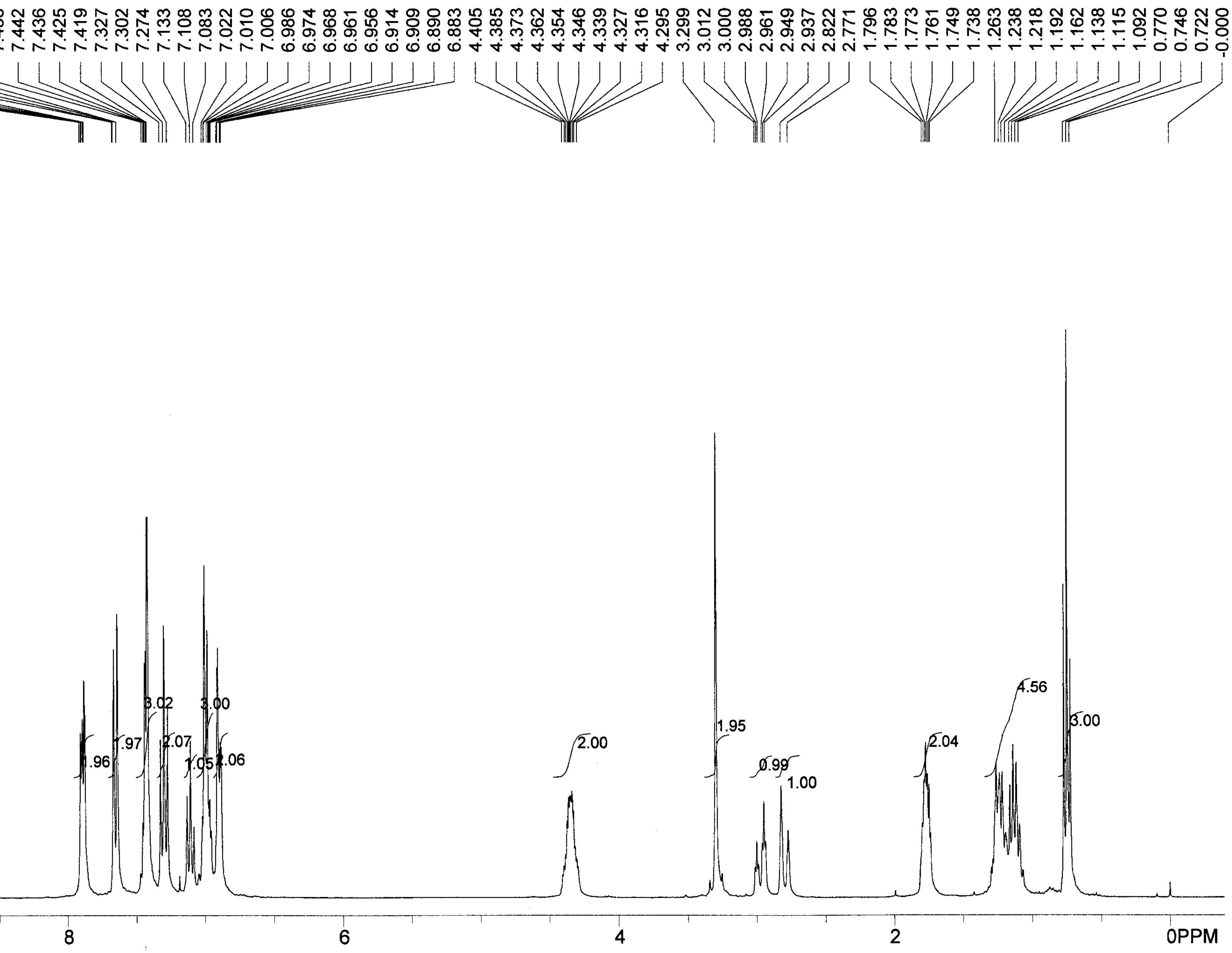

^{1}H NMR

2019-07-18 20:02:44.093
zyc-4-142
SOLVENT: CDCl3
Experiment = zg30
Pulse length = 14.000 usec
Relaxation delay = 1.000 sec
NA = 8
F1 = 300.130005 MHz
F2 = 1.000000 MHz

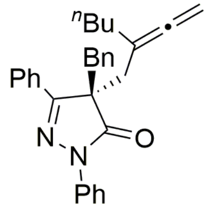

(*S*)-**3as**

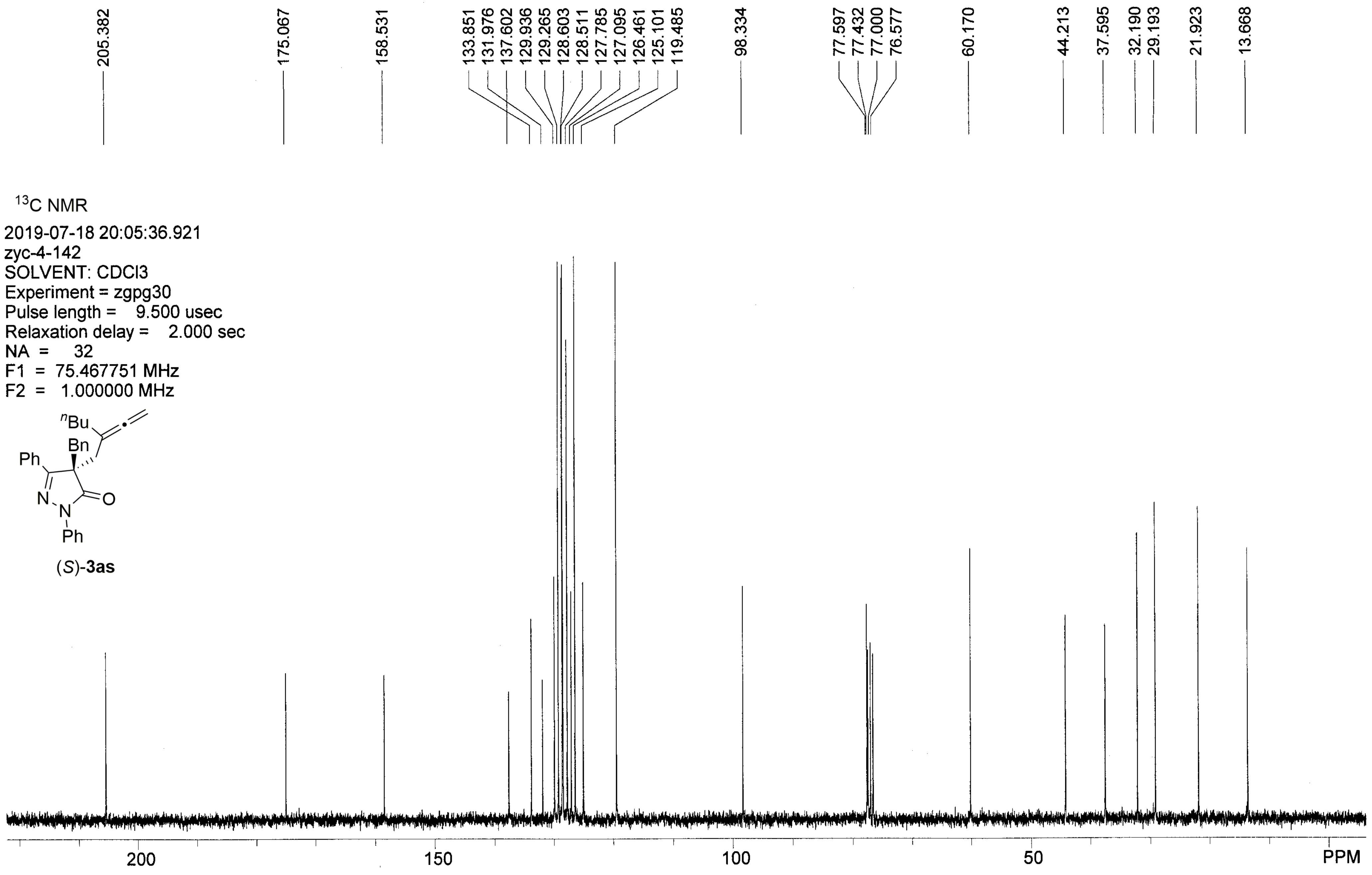
205.382
175.067
158.531
133.851
131.976
137.602
129.936
129.265
128.603
128.511
127.785
127.095
126.461
125.101
119.485
98.334
77.597
77.432
77.000
76.577
60.170
44.213
37.595
32.190
29.193
21.923
13.668
^{13}C NMR
2019-07-18 20:05:36.921
zyc-4-142
SOLVENT: CDCl3
Experiment = zgpg30
Pulse length = 9.500 usec
Relaxation delay = 2.000 sec
NA = 32
F1 = 75.467751 MHz
F2 = 1.000000 MHz
^{n}Bu
Bn
Ph
N
N
O
Ph
(S)-3as
200
150
100
50
PPM

zyc-4-142

实验时间：2019-07-18,20:56:25
谱图文件:D:\浙大智达\N2000\样品\S20190718205625.org
方法文件:D:\浙大智达\N2000\djx.mtd

实验者：zyc
报告时间：2019-07-18,21:10:53
积分方法:面积归一法

实验内容简介：
ia,n-hexane/i-PrOH = 90/10,1.0,254

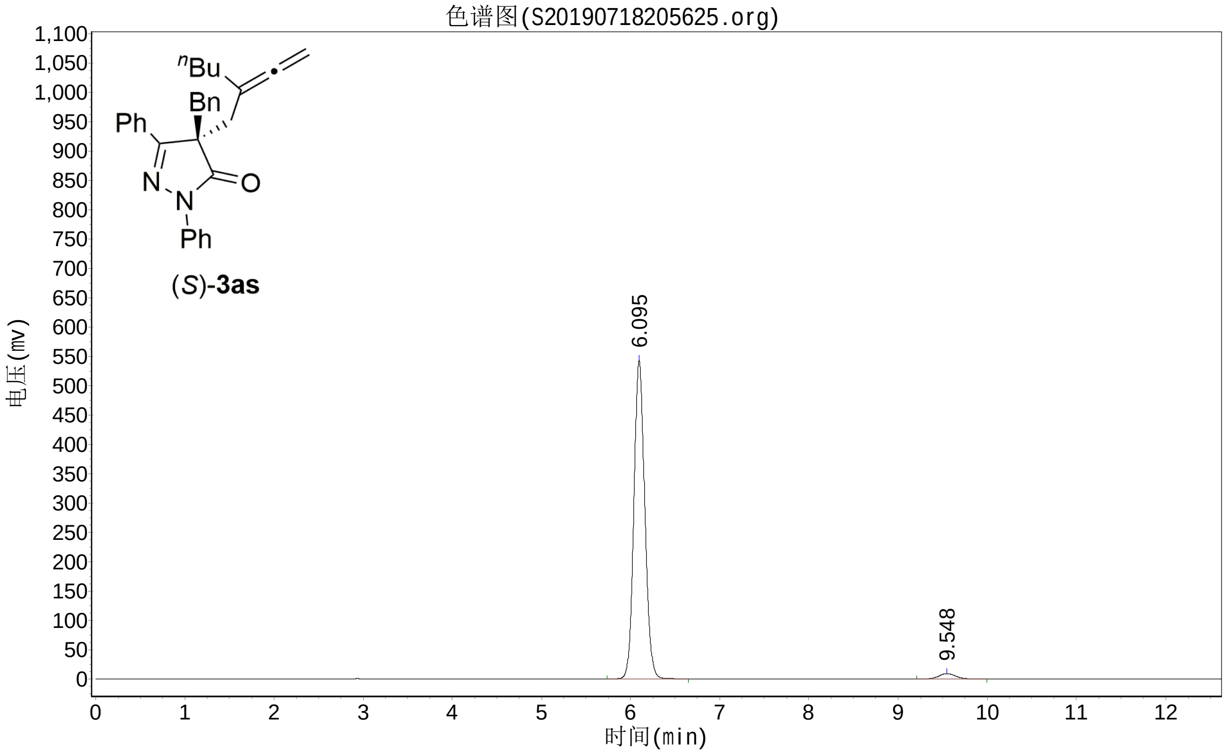

分析结果表

峰号	峰名	保留时间	峰高	峰面积	含量
1		6.095	542785.125	4632142.000	97.3335
2		9.548	9567.741	126899.102	2.6665
总计			552352.866	4759041.102	100.0000

zyc-4-142mix

实验时间：2019-07-18,21:39:07
谱图文件:D:\浙大智达\N2000\样品\S20190718213907.org
方法文件:D:\浙大智达\N2000\djx.mtd

实验者：zyc
报告时间：2019-07-18,21:56:21
积分方法:面积归一法

实验内容简介：
ia,n-hexane/i-PrOH = 90/10,1.0,254

色谱图(S20190718213907.org)

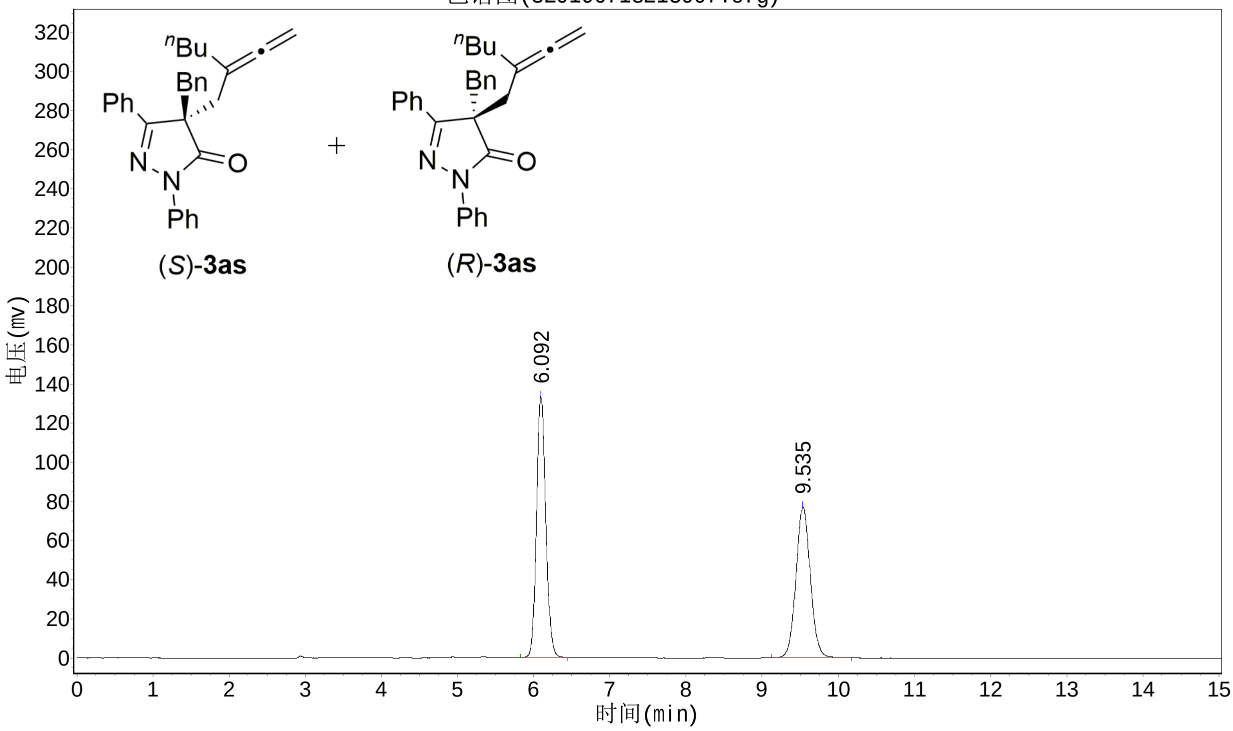

分析结果表

峰号	峰名	保留时间	峰高	峰面积	含量
1		6.092	133501.953	1130080.375	52.7279
2		9.535	77054.789	1013150.063	47.2721
总计			210556.742	2143230.438	100.0000

^{1}H NMR
2020-12-18 15:13:39.734
zyc-6-140
NA = 8
Solvent = CDCl3
F1 = 300.130005 MHz
F2 = 1.000000 MHz

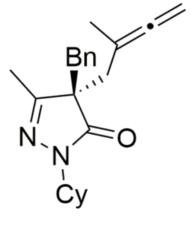

(*S*)-**3bu**

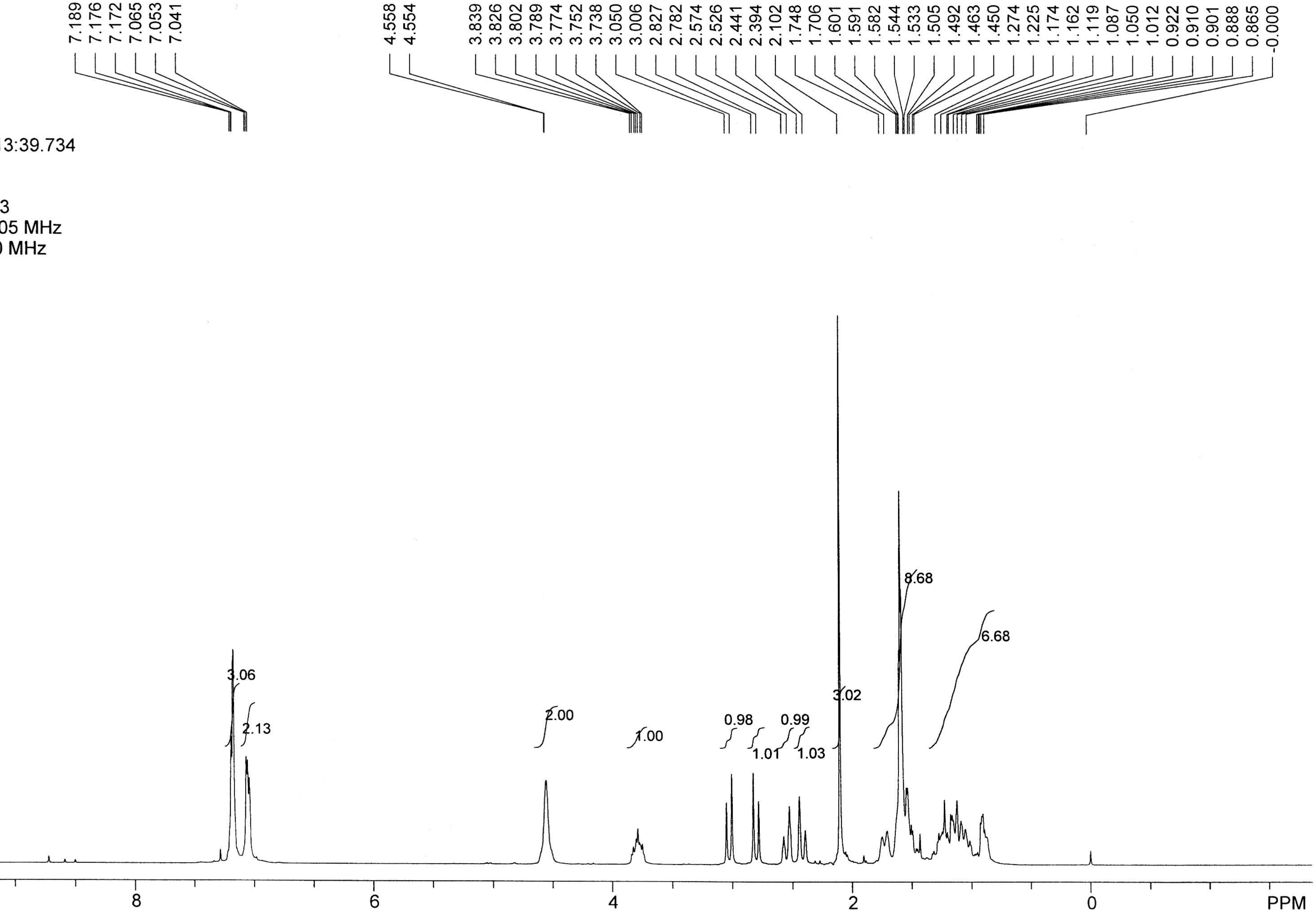

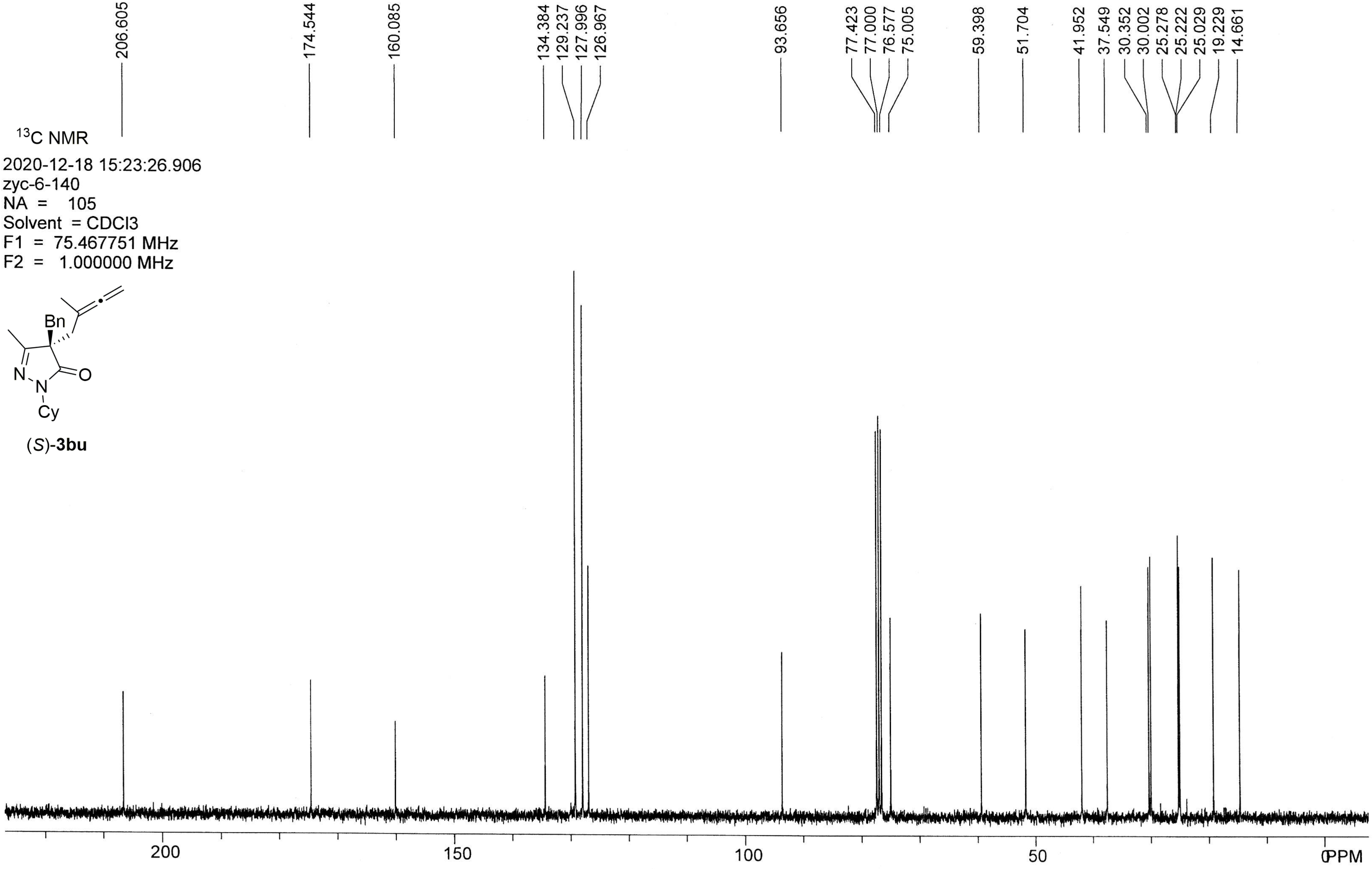

206.605
174.544
160.085
134.384
129.237
127.996
126.967
93.656
77.423
77.000
76.577
75.005
59.398
51.704
41.952
37.549
30.352
30.002
25.278
25.222
25.029
19.229
14.661
^{13}C NMR
2020-12-18 15:23:26.906
zyc-6-140
NA = 105
Solvent = CDCl3
F1 = 75.467751 MHz
F2 = 1.000000 MHz
Bn
N
N
O
Cy
200
150
100
50
0PPM

(*S*)-**3bu**

2020-12-18 21:02:27.093
zyc-6-140purity
NA = 8
Solvent = CDCl3
F1 = 300.130005 MHz
F2 = 1.000000 MHz

29.5mg sample was added 4.0uL CH_2Br_2

$$\text{purity} = \frac{\frac{148.26}{2\times100} \times \frac{4}{35} \times 336.48}{29.5} \times 100\%$$

= 97%

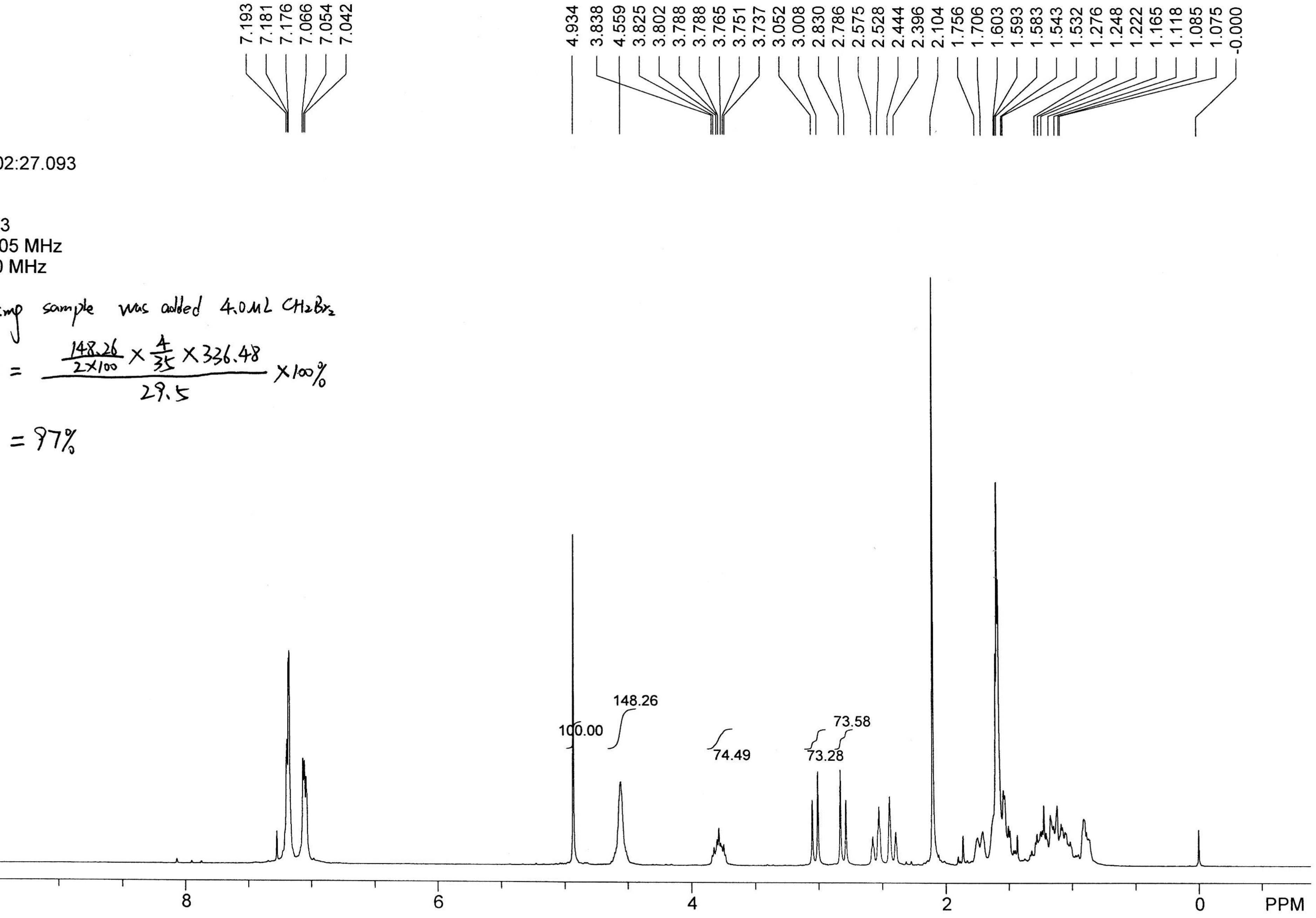

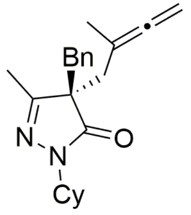

(*S*)-**3bu**

zyc-6-140

实验时间：2020-12-17,21:35:31
谱图文件:D:\浙大智达\N2000\样品\S20201217213531.org
方法文件:D:\浙大智达\N2000\djx.mtd

实验者：zyc
报告时间：2020-12-17,21:58:25
积分方法:面积归一法

实验内容简介：
ia,n-hexane/i-PrOH = 90/10,1.0,254

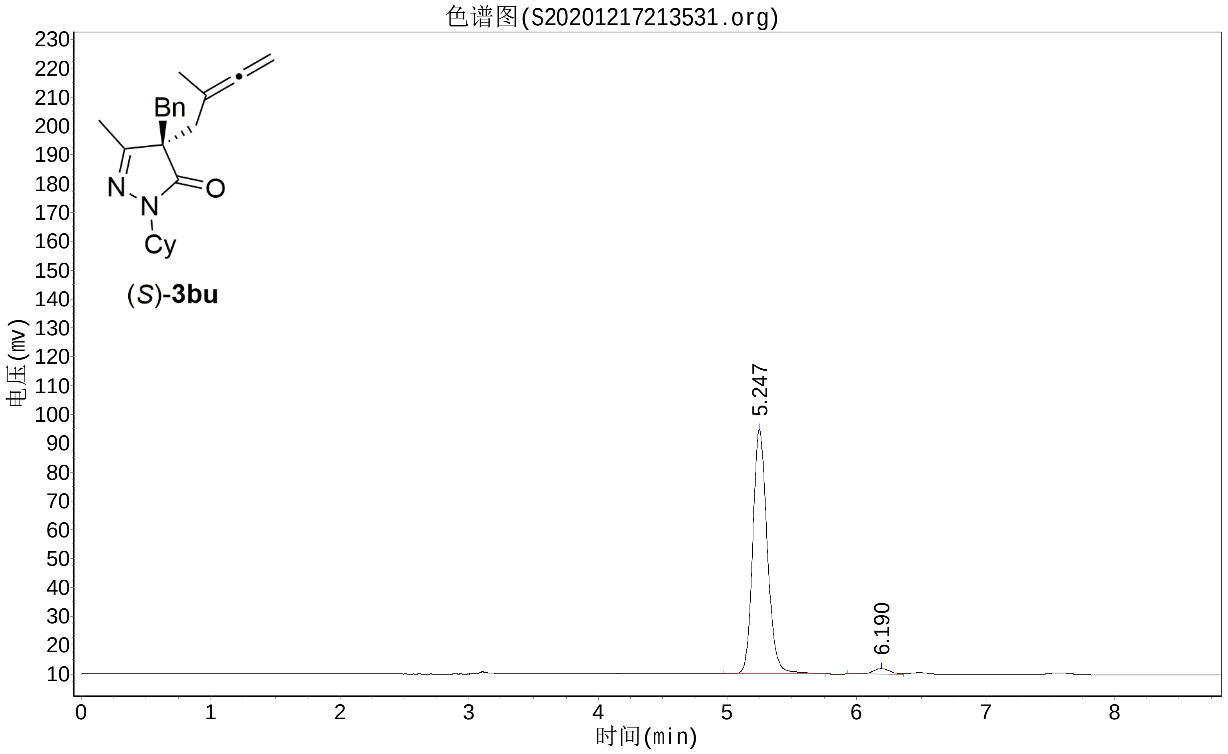

分析结果表

峰号	峰名	保留时间	峰高	峰面积	含量
1		5.247	84927.836	661223.813	97.5003
2		6.190	1888.196	16952.416	2.4997
总计			86816.032	678176.229	100.0000

zyc-6-140mix

实验时间：2020-12-17,22:08:02
谱图文件:D:\浙大智达\N2000\样品\S20201217220802.org
方法文件:D:\浙大智达\N2000\djx.mtd

实验者：zyc
报告时间：2020-12-17,22:18:45
积分方法:面积归一法

实验内容简介：
ia,n-hexane/i-PrOH = 90/10,1.0,254

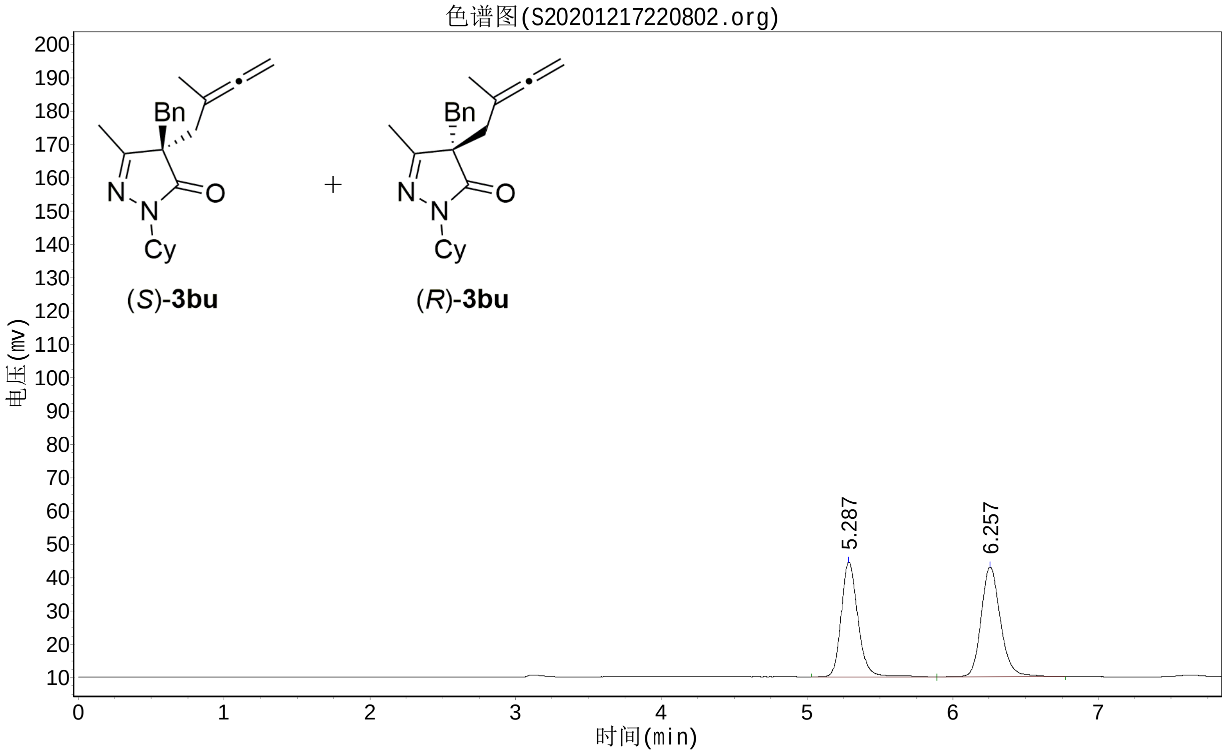

分析结果表

峰号	峰名	保留时间	峰高	峰面积	含量
1		5.287	34358.934	273516.469	47.3397
2		6.257	32990.156	304258.031	52.6603
总计			67349.090	577774.500	100.0000

^{1}H NMR
2020-12-18 15:28:55.484
zyc-6-142
NA = 8
Solvent = CDCl3
F1 = 300.130005 MHz
F2 = 1.000000 MHz

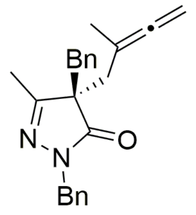

(*S*)-**3bv**

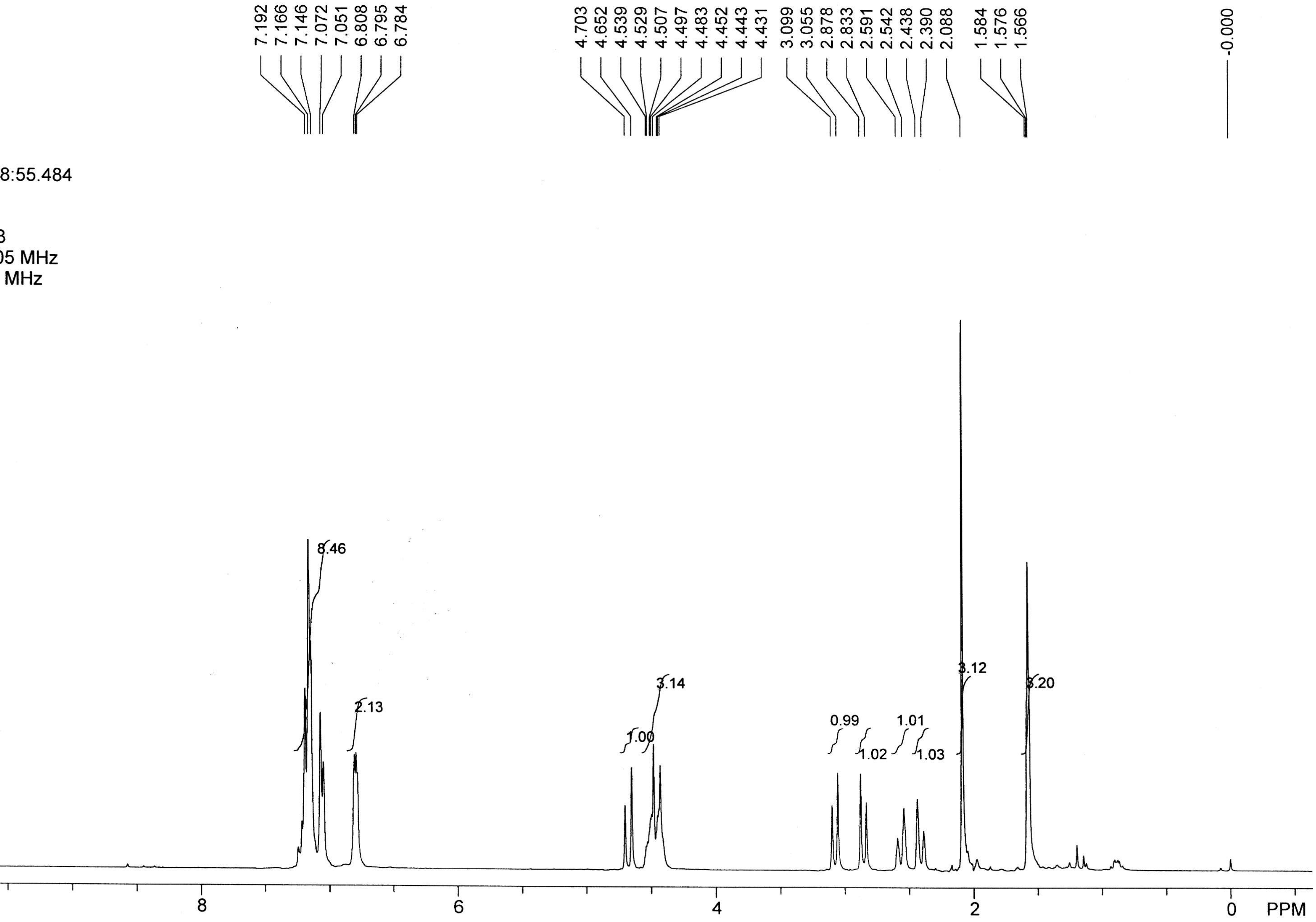

^{13}C NMR

2020-12-18 15:23:26.906
zyc-6-140
NA = 105
Solvent = CDCl3
F1 = 75.467751 MHz
F2 = 1.000000 MHz

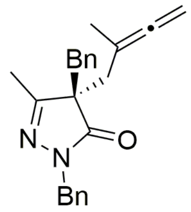

(*S*)-**3bv**

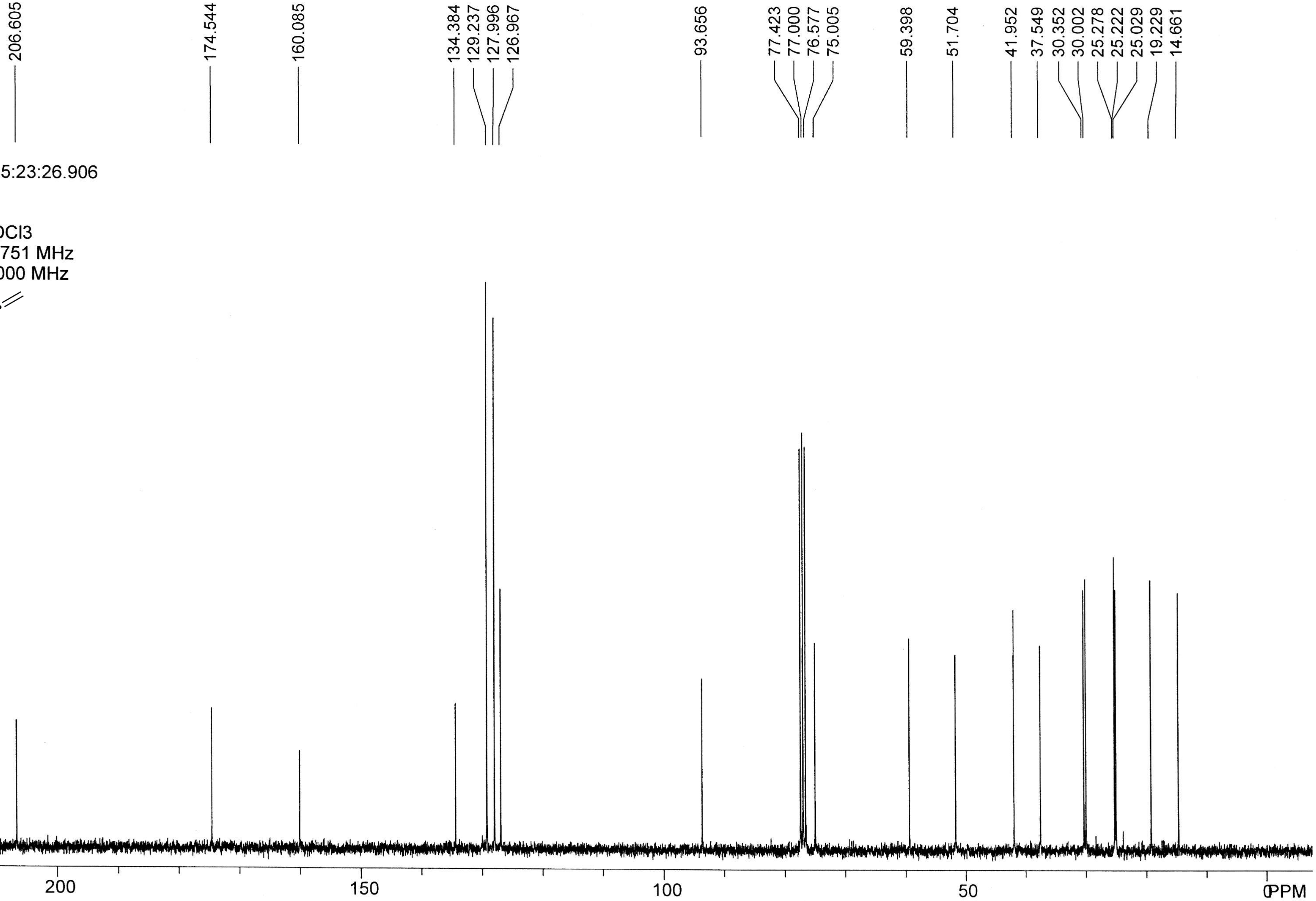

2020-12-18 21:10:34.296
zyc-6-142purity
NA = 8
Solvent = CDCl3
F1 = 300.130005 MHz
F2 = 1.000000 MHz

23.8 mg sample was added 3 μL CH_2Br_2

$$\text{purity} = \frac{\frac{78.89}{100} \times \frac{3}{35} \times 344.46}{23.8} \times 100\%$$

$= 98\%$

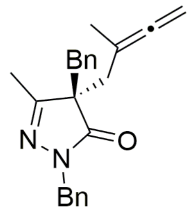

(S)-**3bv**

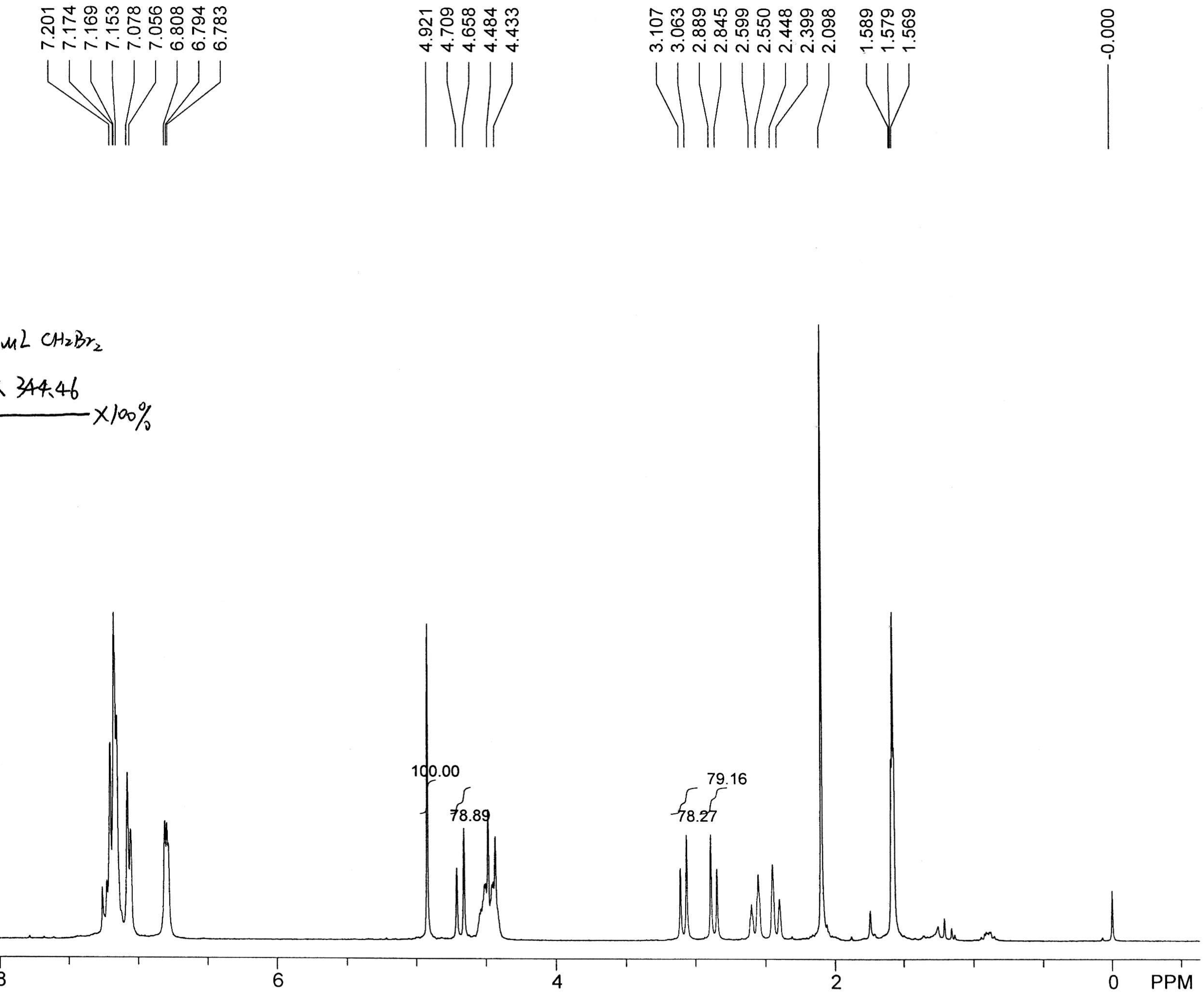

zyc-6-142

实验时间：2020-12-18,16:53:03
谱图文件:D:\浙大智达\N2000\样品\S20201218165303.org
方法文件:D:\浙大智达\N2000\djx.mtd

实验者：zyc
报告时间：2020-12-18,17:09:12
积分方法:面积归一法

实验内容简介：
ia,n-hexane/i-PrOH = 90/10,1.0,254

色谱图(S20201218165303.org)

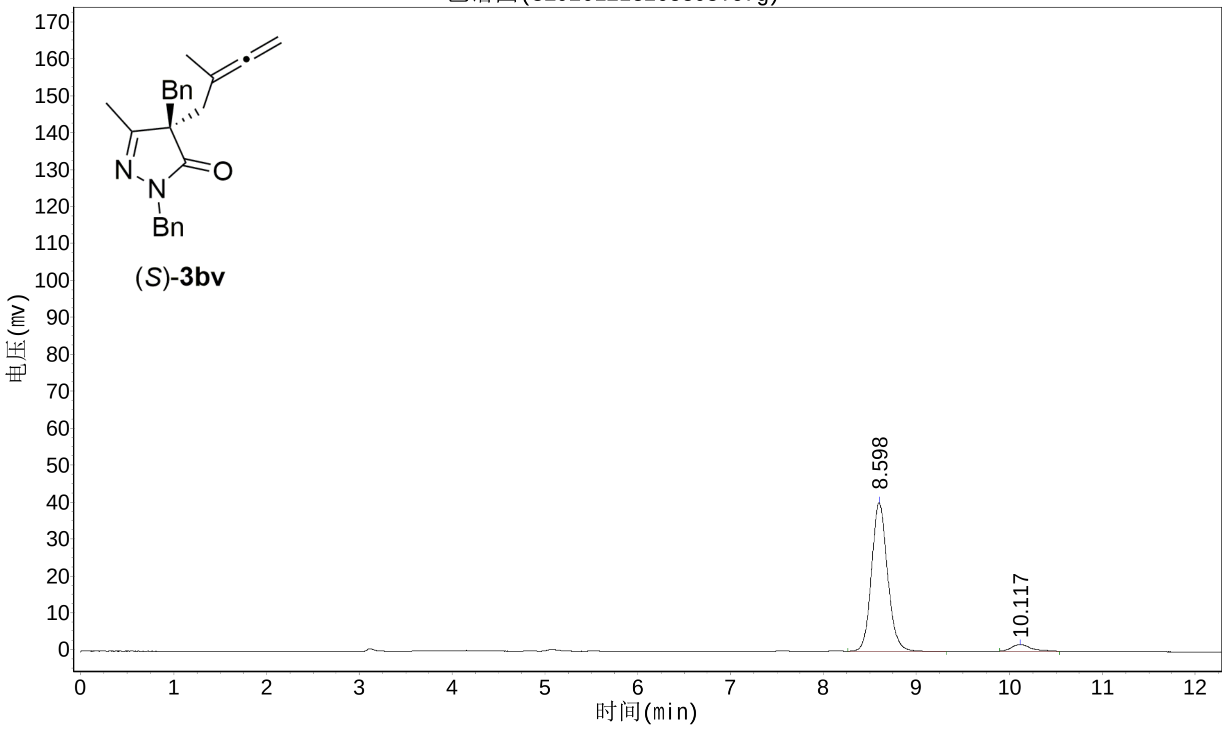

分析结果表

峰号	峰名	保留时间	峰高	峰面积	含量
1		8.598	40263.625	482625.313	94.8137
2		10.117	1791.573	26399.500	5.1863
总计			42055.198	509024.813	100.0000

zyc-6-142mix

实验时间：2020-12-18,17:55:31
谱图文件:D:\浙大智达\N2000\样品\S20201218175531.org
方法文件:D:\浙大智达\N2000\djx.mtd

实验者：zyc
报告时间：2020-12-18,18:23:31
积分方法:面积归一法

实验内容简介：
ia,n-hexane/i-PrOH = 90/10,1.0,254

色谱图(S20201218175531.org)

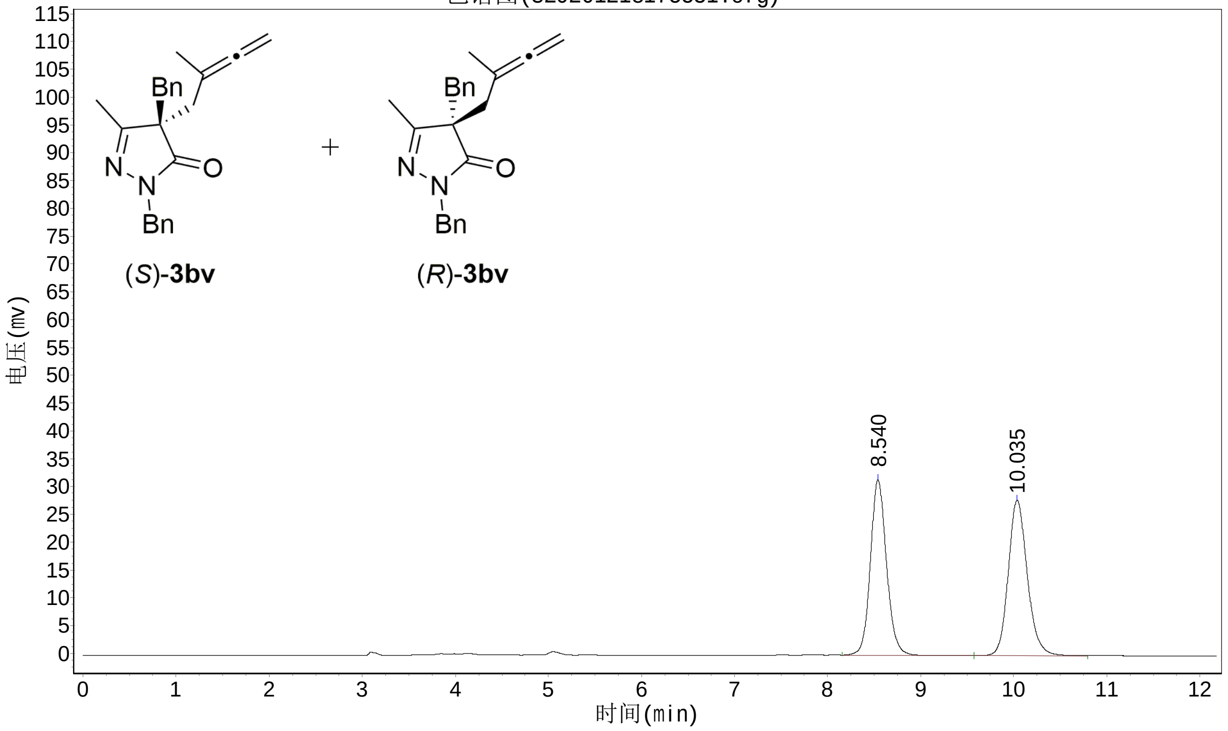

分析结果表

峰号	峰名	保留时间	峰高	峰面积	含量
1		8.540	31643.889	384463.375	49.0643
2		10.035	27979.572	399128.250	50.9357
总计			59623.461	783591.625	100.0000

^1H NMR

2019-09-05 11:48:38.109
zyc-4-151
SOLVENT: CDCl3
Experiment = zg30
Pulse length = 14.000 usec
Relaxation delay = 1.000 sec
NA = 8
F1 = 300.130005 MHz
F2 = 1.000000 MHz

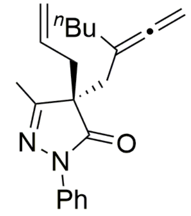

(*S*)-**3am**

Gram-scale

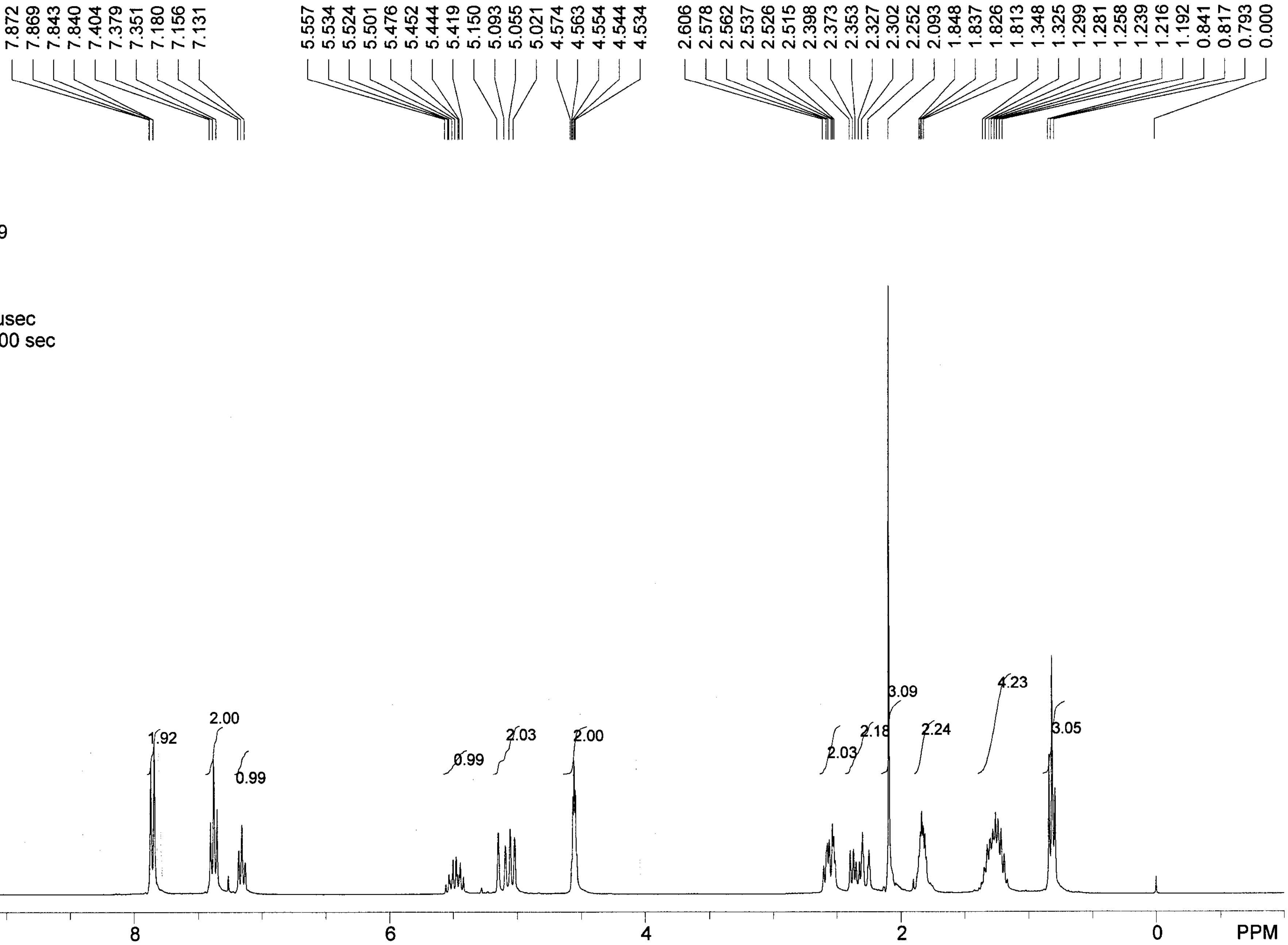

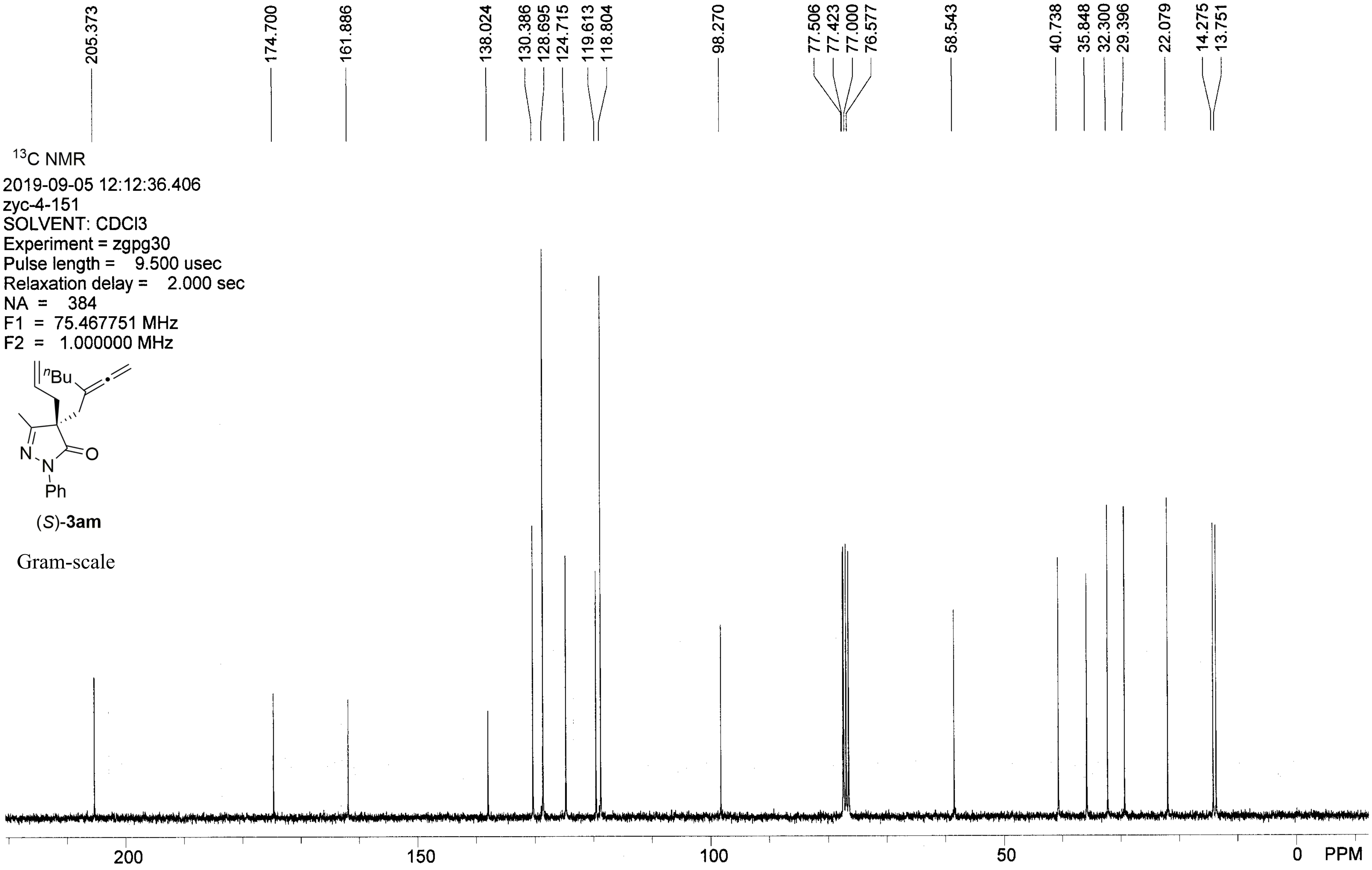

^{13}C NMR
2019-09-05 12:12:36.406
zyc-4-151
SOLVENT: CDCl3
Experiment = zgpg30
Pulse length = 9.500 usec
Relaxation delay = 2.000 sec
NA = 384
F1 = 75.467751 MHz
F2 = 1.000000 MHz

(*S*)-**3am**

Gram-scale

zyc-4-151

实验时间：2019-09-05,12:39:13
谱图文件:D:\浙大智达\N2000\样品\S20190905123913.org
方法文件:D:\浙大智达\N2000\djx.mtd

实验者：zyc
报告时间：2019-09-05,12:48:58
积分方法:面积归一法

实验内容简介：
od,n-hexane/i-PrOH = 98/2,1.0,254

色谱图(S20190905123913.org)

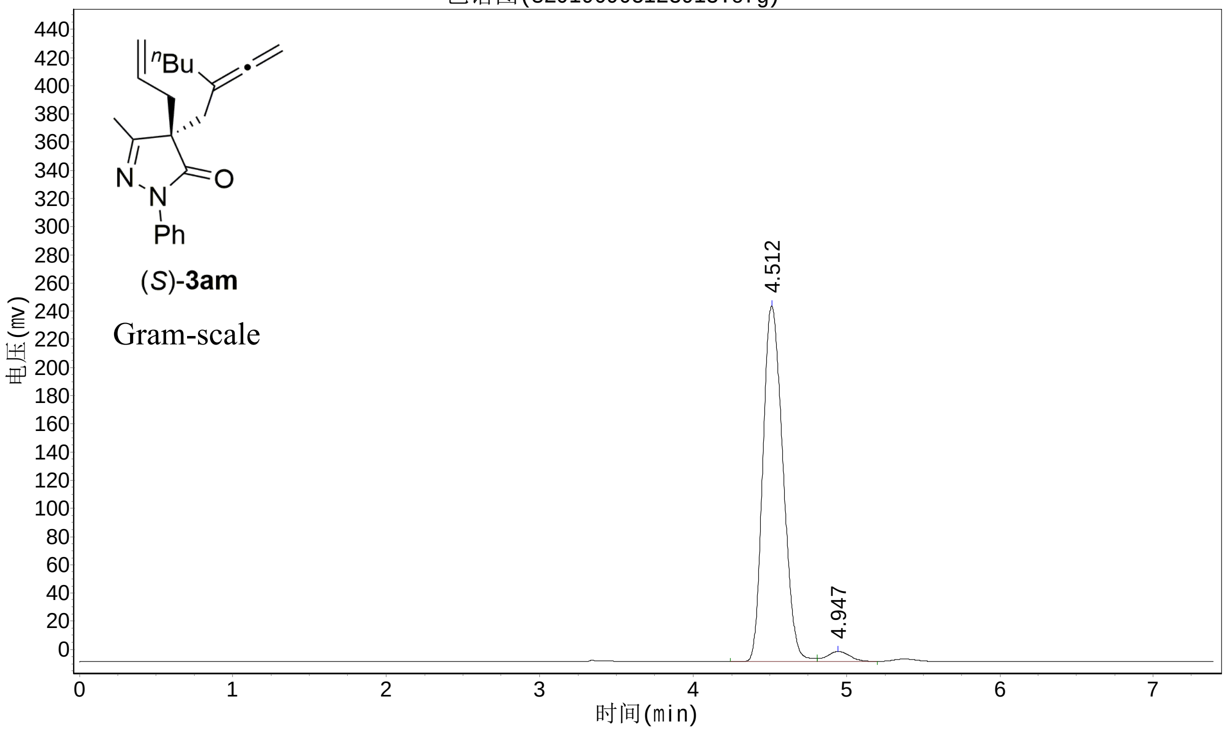

分析结果表

峰号	峰名	保留时间	峰高	峰面积	含量
1		4.512	252460.625	2290747.500	96.7465
2		4.947	7322.021	77036.789	3.2535
总计			259782.646	2367784.289	100.0000

zyc-4-151mix

实验时间：2019-09-05,12:50:19
谱图文件:D:\浙大智达\N2000\样品\S20190905125019.org
方法文件:D:\浙大智达\N2000\djx.mtd

实验者：zyc
报告时间：2019-09-05,12:58:41
积分方法:面积归一法

实验内容简介：
od,n-hexane/i-PrOH = 98/2,1.0,254

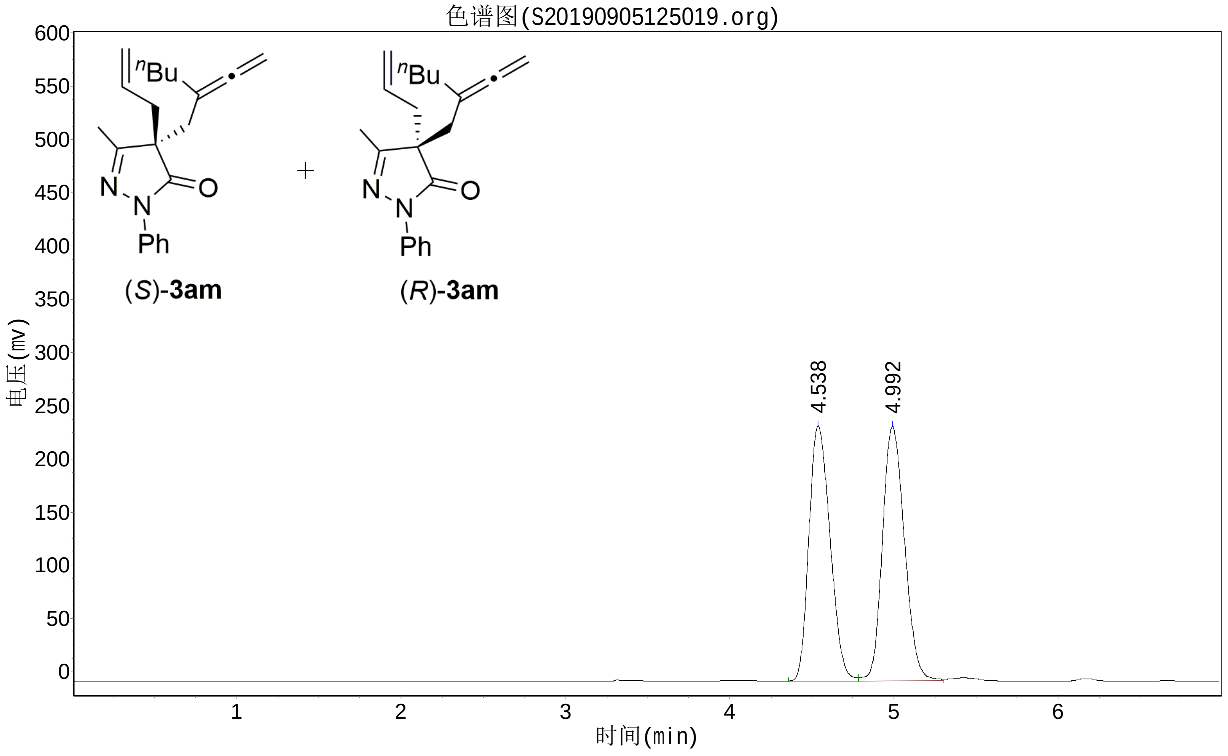

分析结果表

峰号	峰名	保留时间	峰高	峰面积	含量
1		4.538	239187.219	2145647.250	49.0460
2		4.992	239015.656	2229121.500	50.9540
总计			478202.875	4374768.750	100.0000

^{1}H NMR

2019-10-18 18:06:05.906
zyc-4-193
SOLVENT: CDCl3
Experiment = zg30
Pulse length = 14.000 usec
Relaxation delay = 1.000 sec
NA = 8
F1 = 300.130005 MHz
F2 = 1.000000 MHz

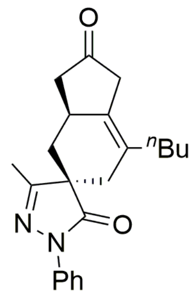

(3a*S*,5*S*)-**5am**

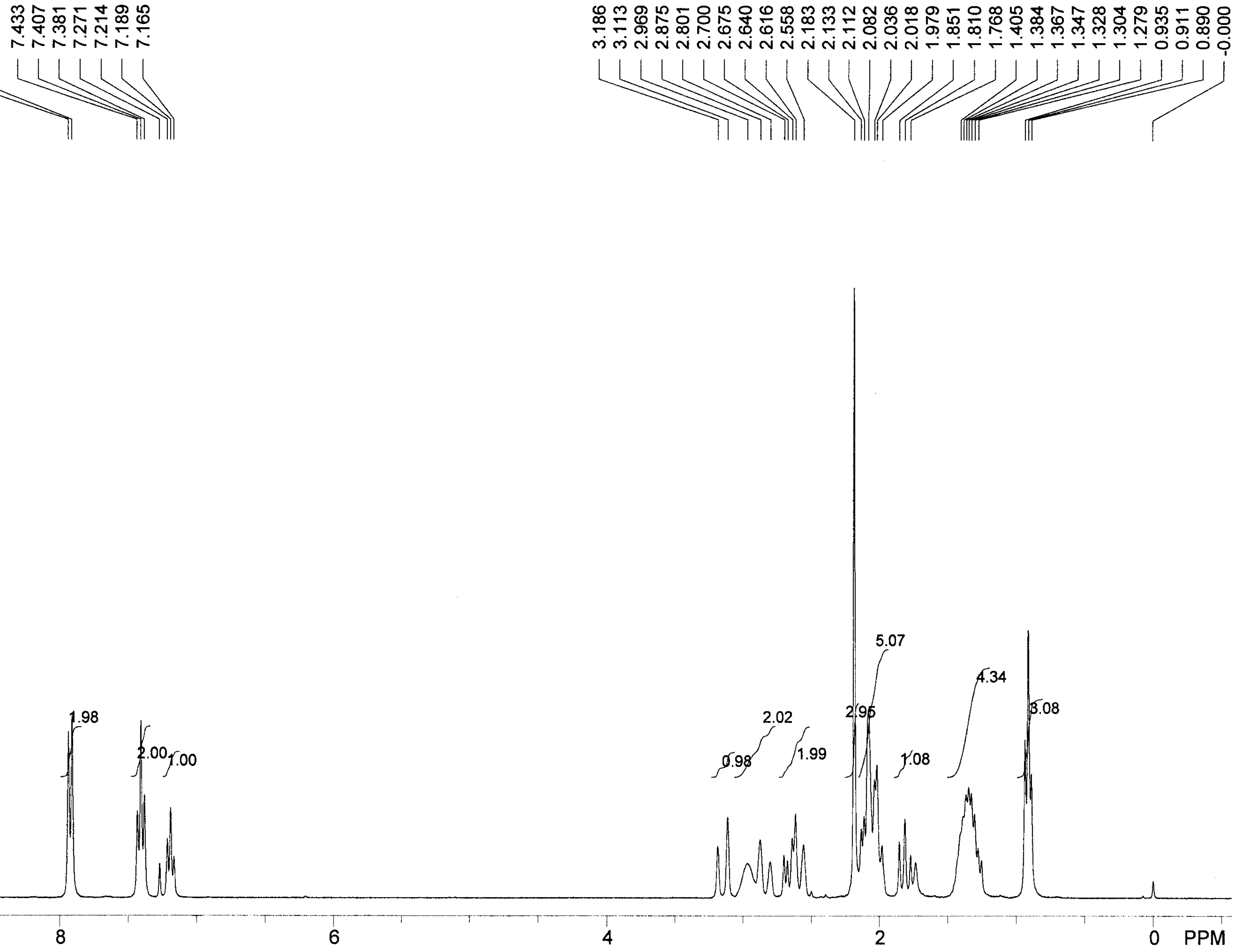

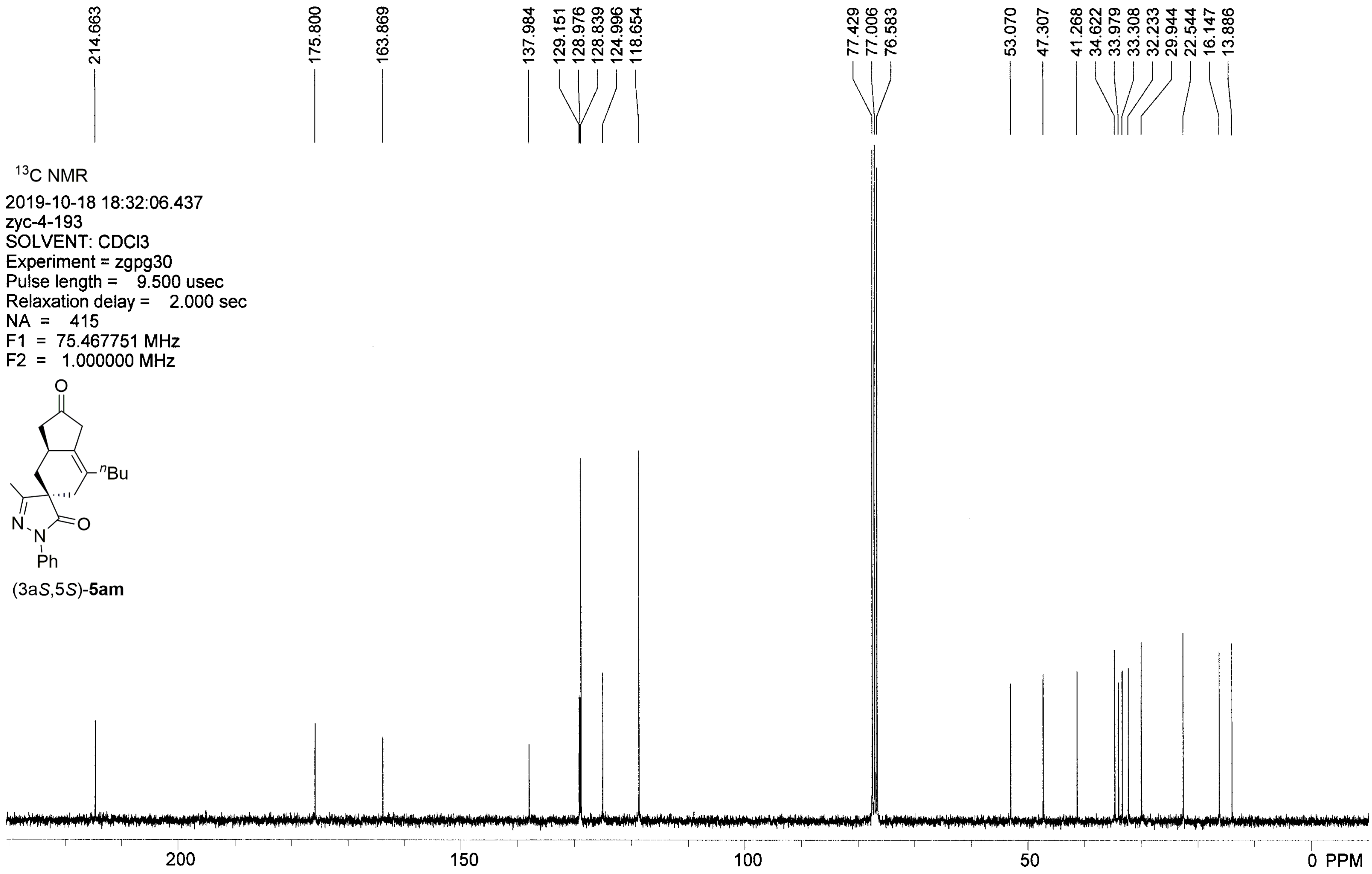

214.663
175.800
163.869
137.984
129.151
128.976
128.839
124.996
118.654
77.429
77.006
76.583
53.070
47.307
41.268
34.622
33.979
33.308
32.233
29.944
22.544
16.147
13.886
^{13}C NMR
2019-10-18 18:32:06.437
zyc-4-193
SOLVENT: CDCl3
Experiment = zgpg30
Pulse length = 9.500 usec
Relaxation delay = 2.000 sec
NA = 415
F1 = 75.467751 MHz
F2 = 1.000000 MHz
O
^{n}Bu
N
N
O
Ph
(3a*S*,5*S*)-**5am**
200
150
100
50
0 PPM

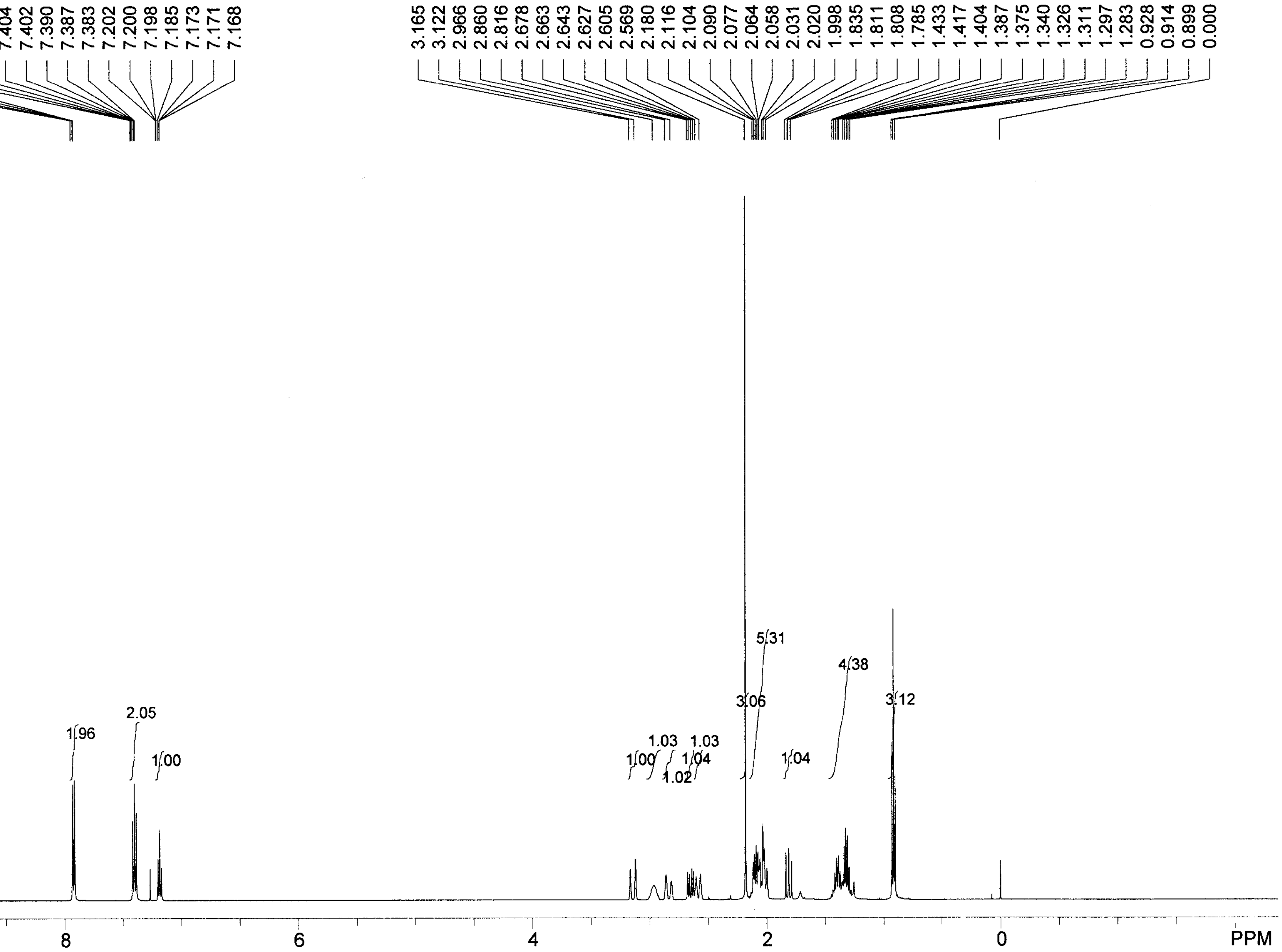

^{1}H NMR
2020-04-02 12:24:16.590
zyc-5-102
NA = 16
Solvent = CDCl3
F1 = 500.130005 MHz
F2 = 1.000000 MHz

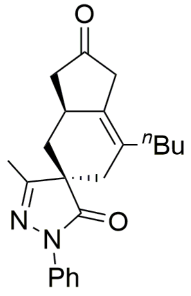

(3a*S*,5*S*)-**5am**

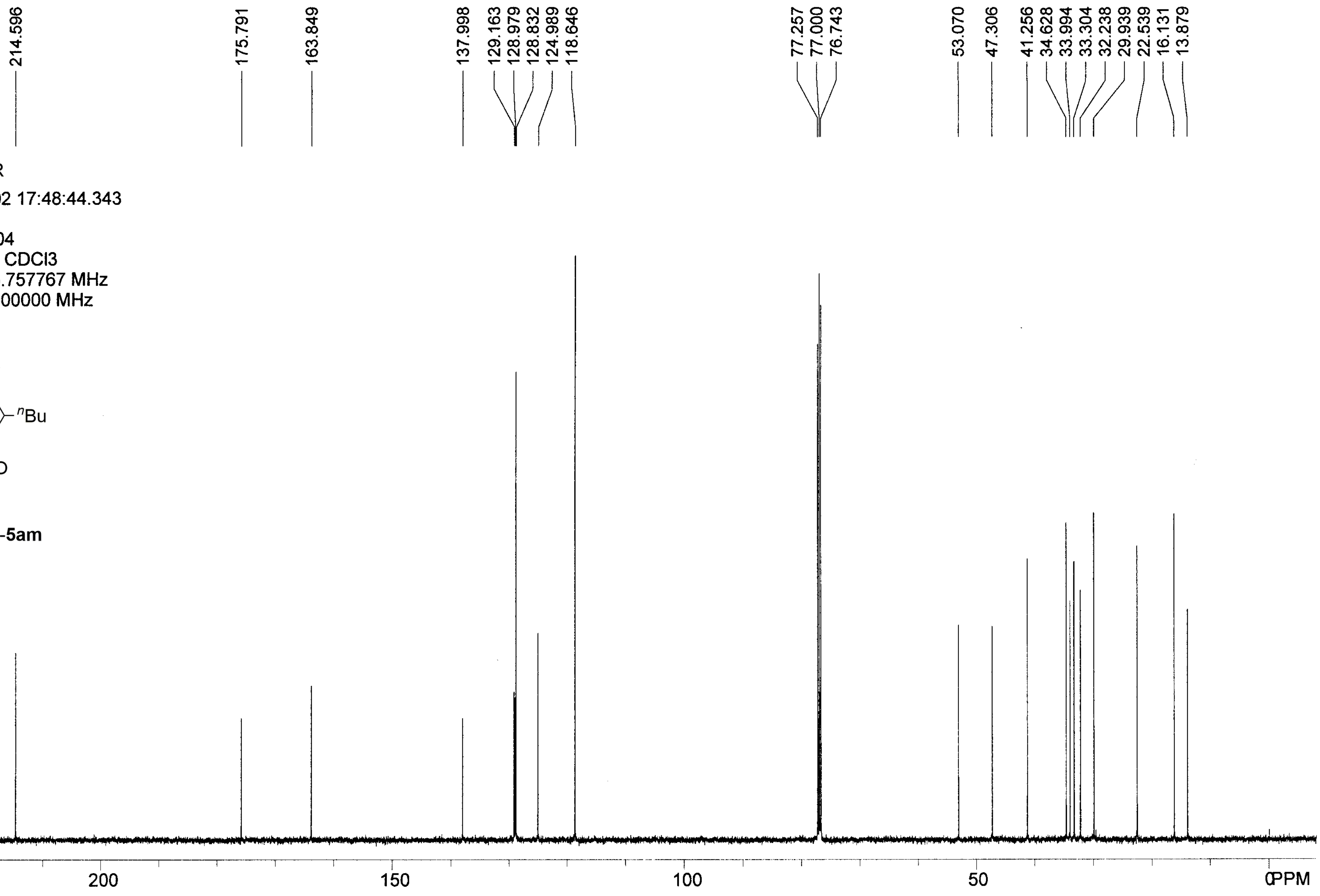

^{13}C NMR

2020-04-02 17:48:44.343
zyc-5-102
NA = 304
Solvent = CDCl3
F1 = 125.757767 MHz
F2 = 1.000000 MHz

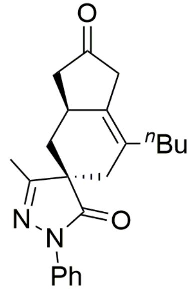

(3a*S*,5*S*)-**5am**

2020-04-02
zyc-5-102
DEFT90
solvent = CDCl3
F1 = 500.130005 MHz
F2 = 1.000000 MHz

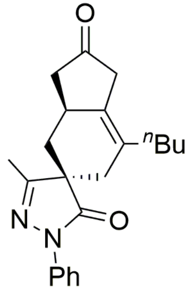

(3a*S*,5*S*)-**5am**

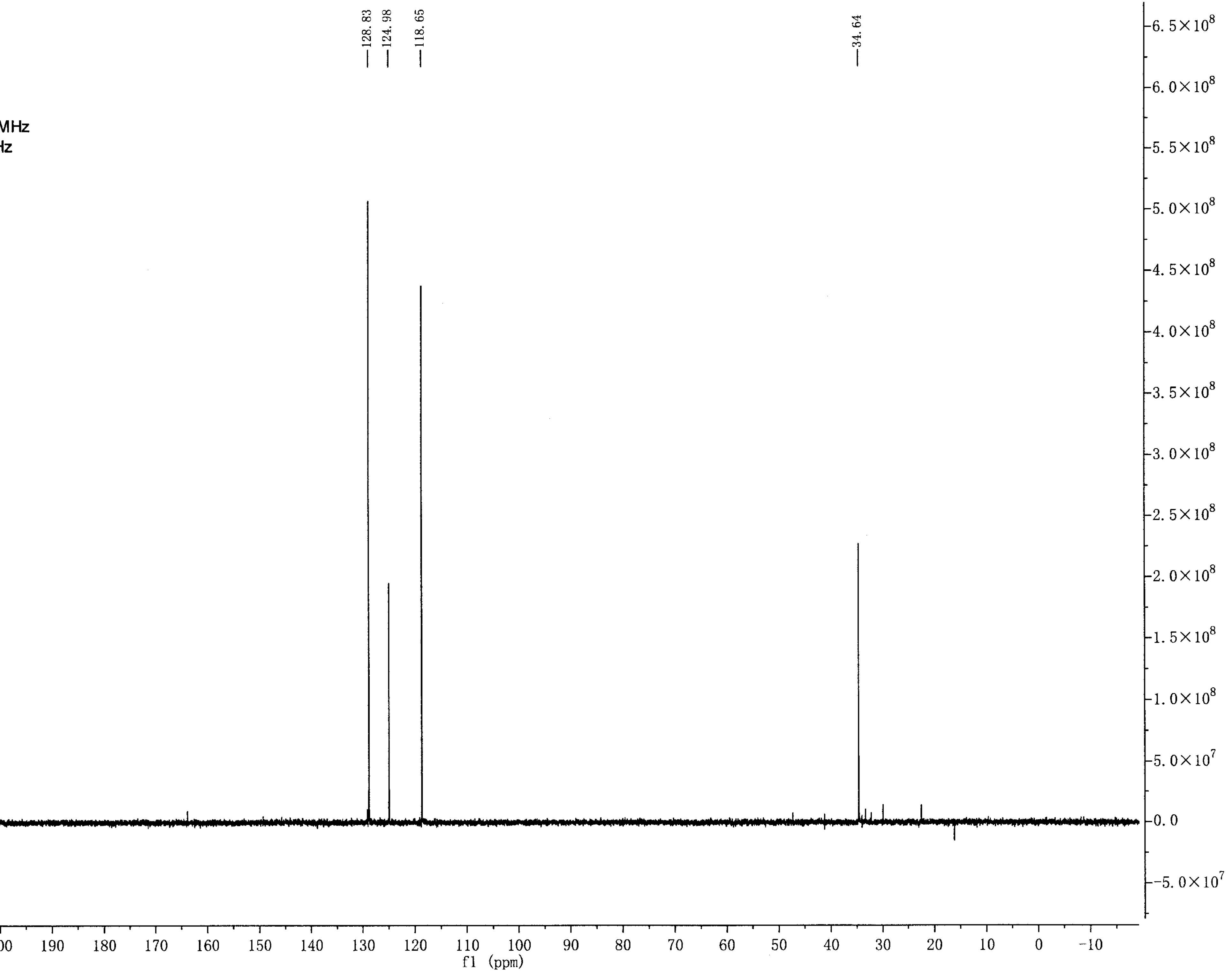

536
2020-04-02
zyc-5-102
DEPT135
solvent = CDCl3
F1 = 500.130005 MHz
F2 = 1.000000 MHz

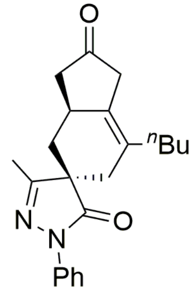

(3a*S*,5*S*)-**5am**

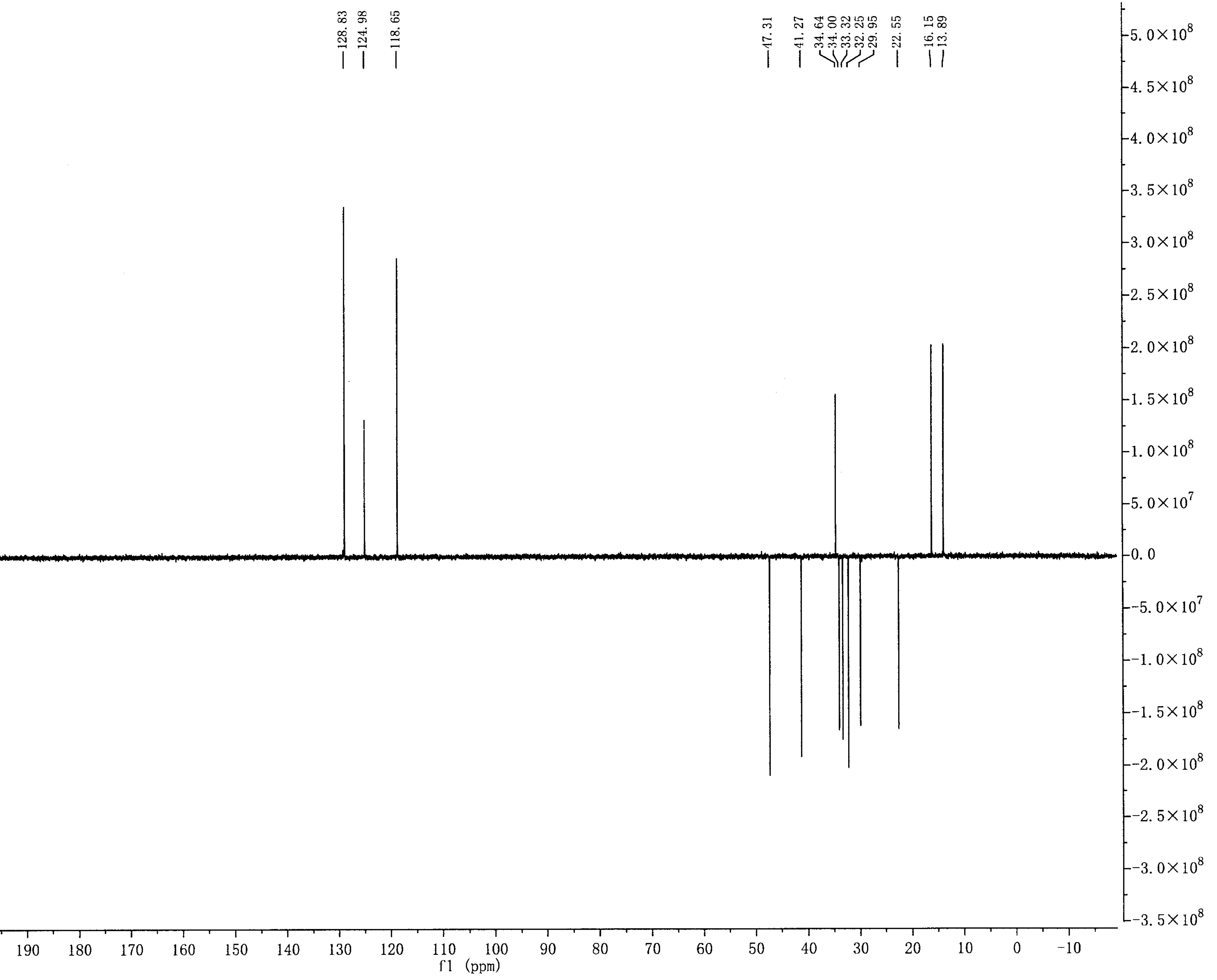

2020-04-02
zyc-5-102noe
solvent = CDCl3
F1 = 500.130005 MHz
F2 = 1.000000 MHz

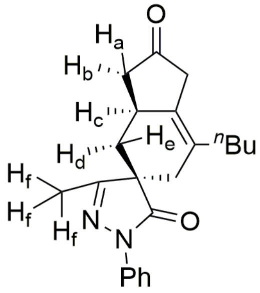

(3a*S*,5*S*)-**5am**

3a*S* was determined by NOE effect between H_c and H_b, H_d, H_f.

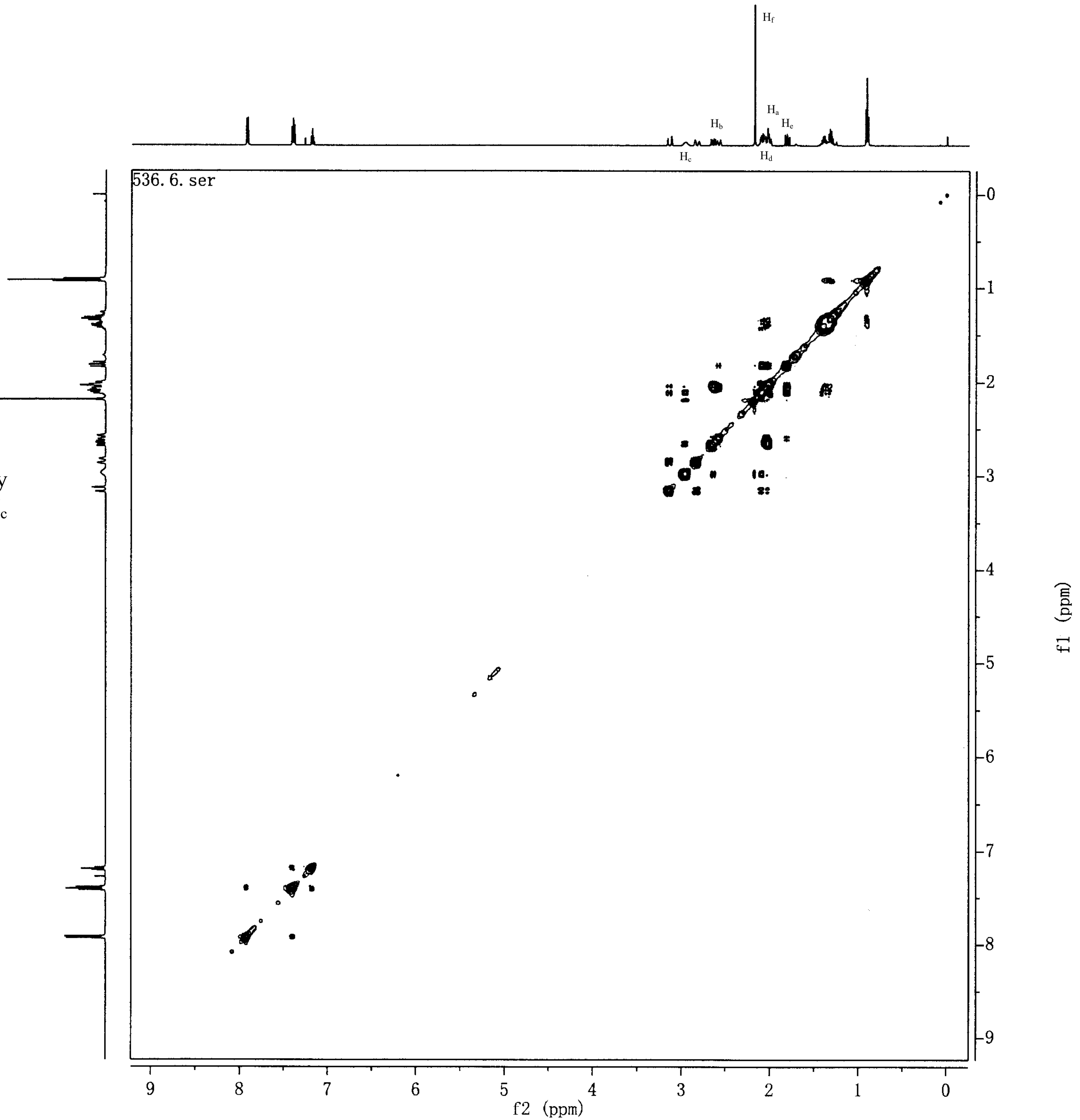

2020-04-02
zyc-5-102noe
solvent = CDCl3
F1 = 500.130005 MHz
F2 = 1.000000 MHz

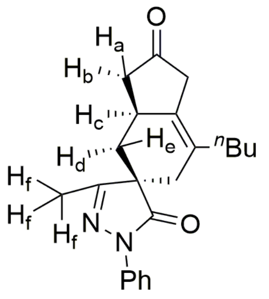

(3a*S*,5*S*)-**5am**

3a*S* was determined by NOE effect between H_c and H_b, H_d, H_f.

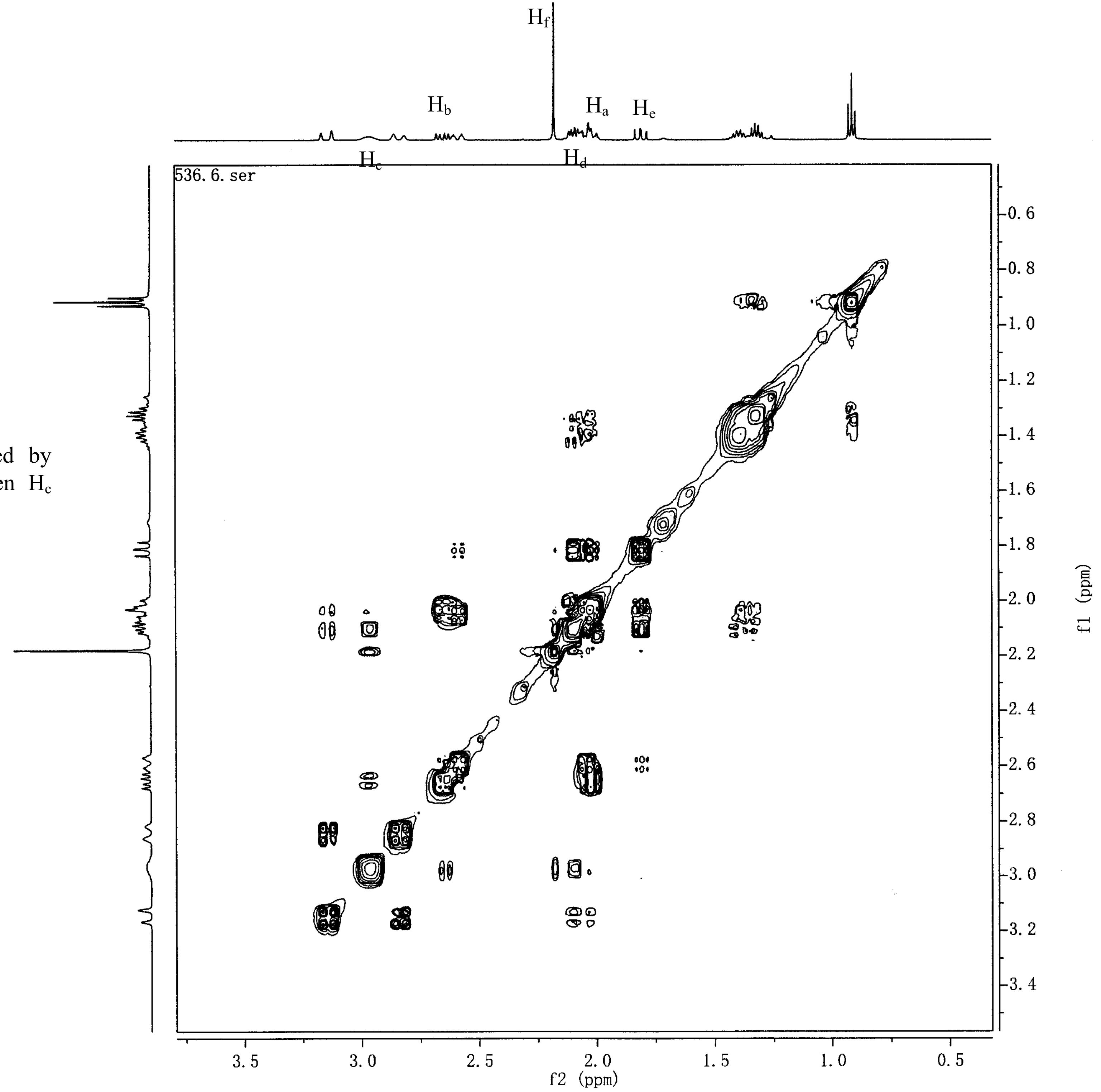

2020-04-02
zyc-5-102noe
solvent = CDCl3
F1 = 500.130005 MHz
F2 = 1.000000 MHz

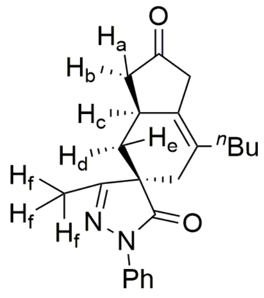

(3a*S*,5*S*)-**5am**

3a*S* was determined by NOE effect between H_c and H_b, H_d, H_f.

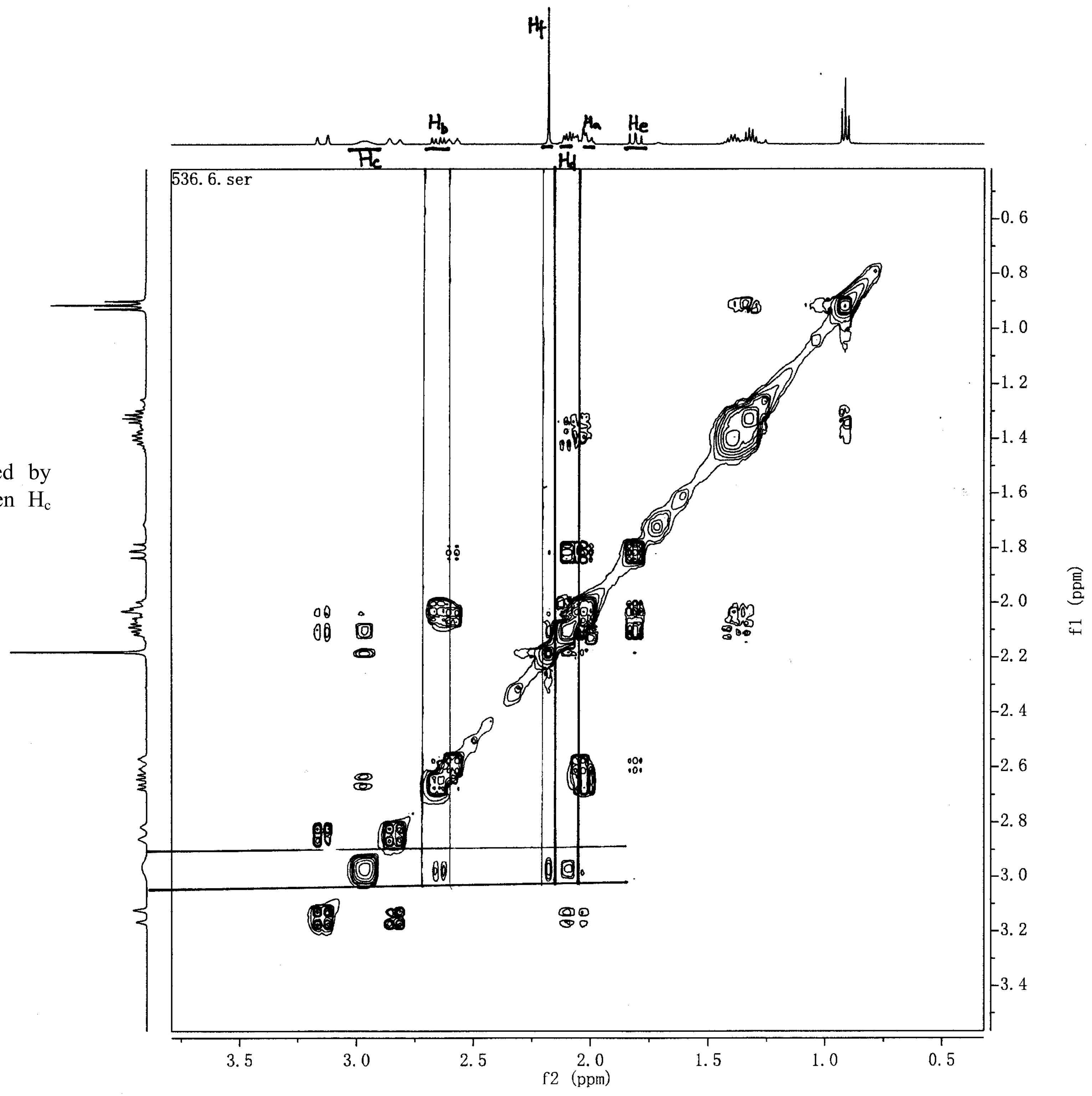

2020-04-02
zyc-5-102cosy
solvent = CDCl3
F1 = 500.130005 MHz
F2 =1.000000 MHz

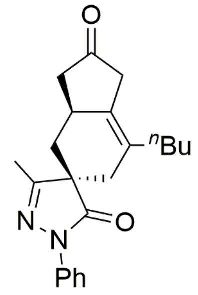

(3a*S*,5*S*)-**5am**

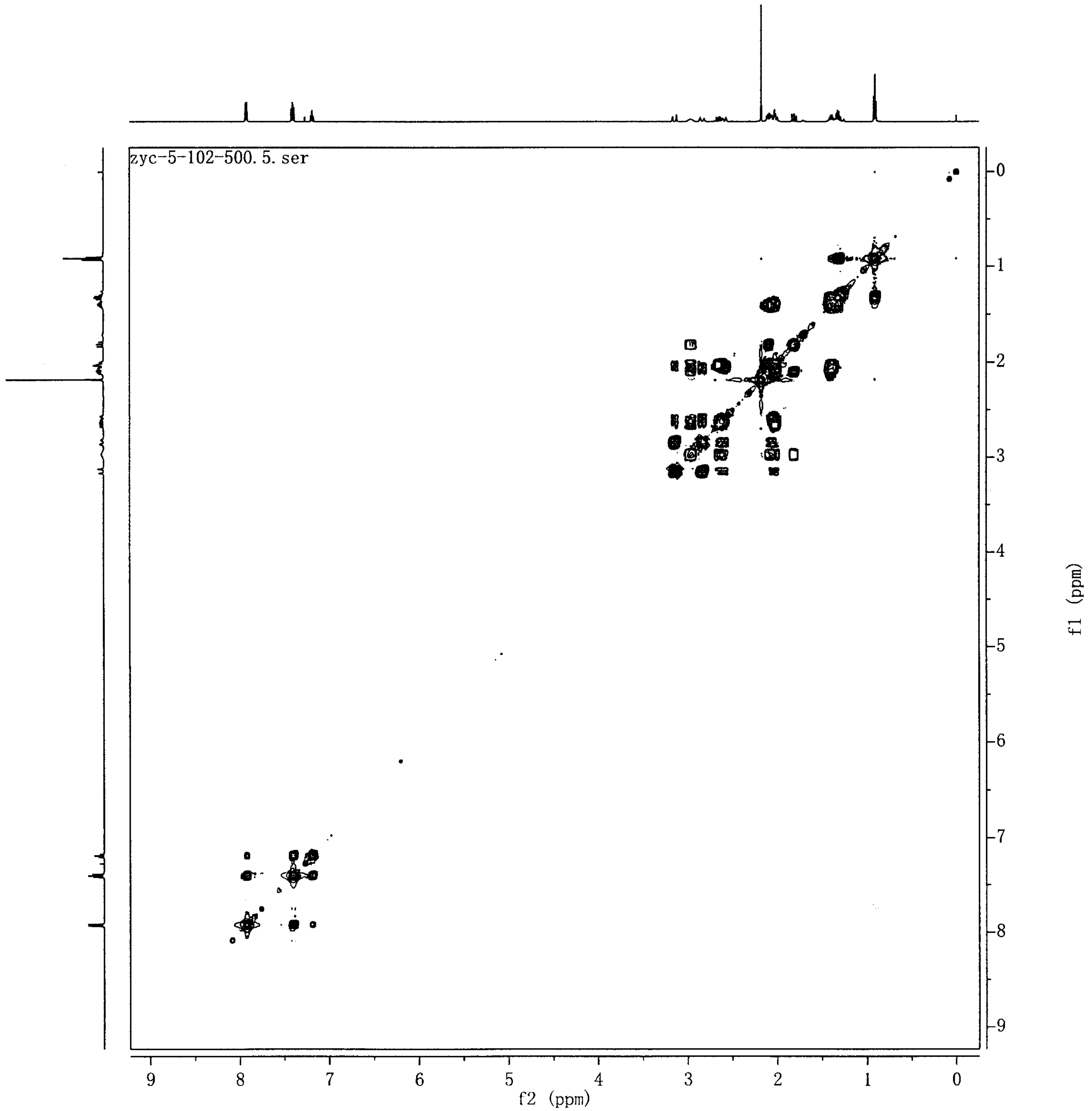

2020-04-02
zyc-5-102cosy
solvent = CDCl3
F1 = 500.130005 MHz
F2 =1.000000 MHz

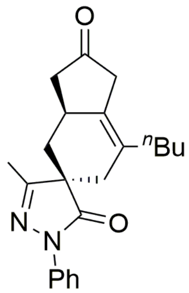

(3a*S*,5*S*)-**5am**

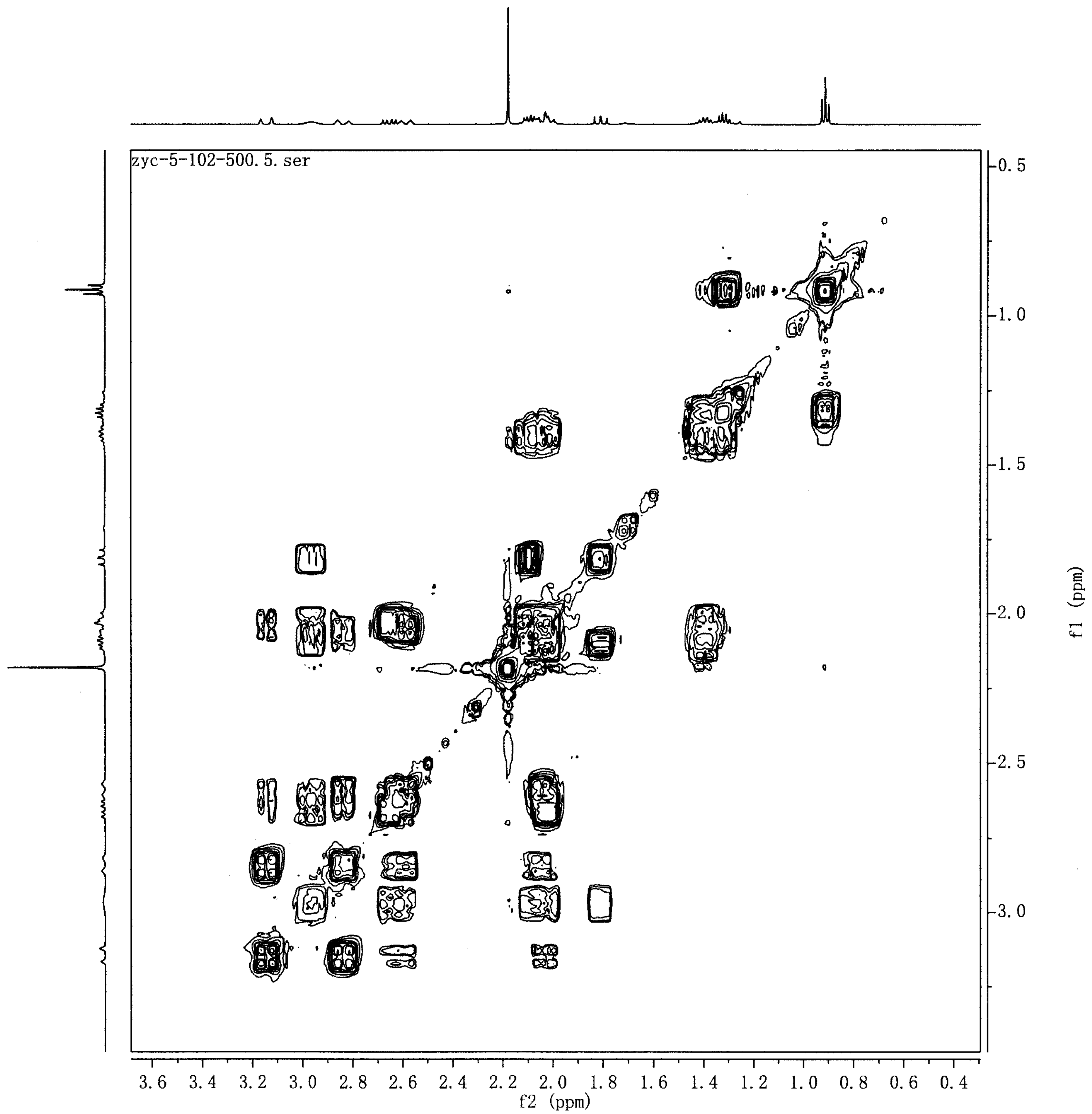

2020-04-02
zyc-5-102HSQC
solvent = CDCl3
F1 = 500.130005 MHz
F2 = 1.000000 MHz

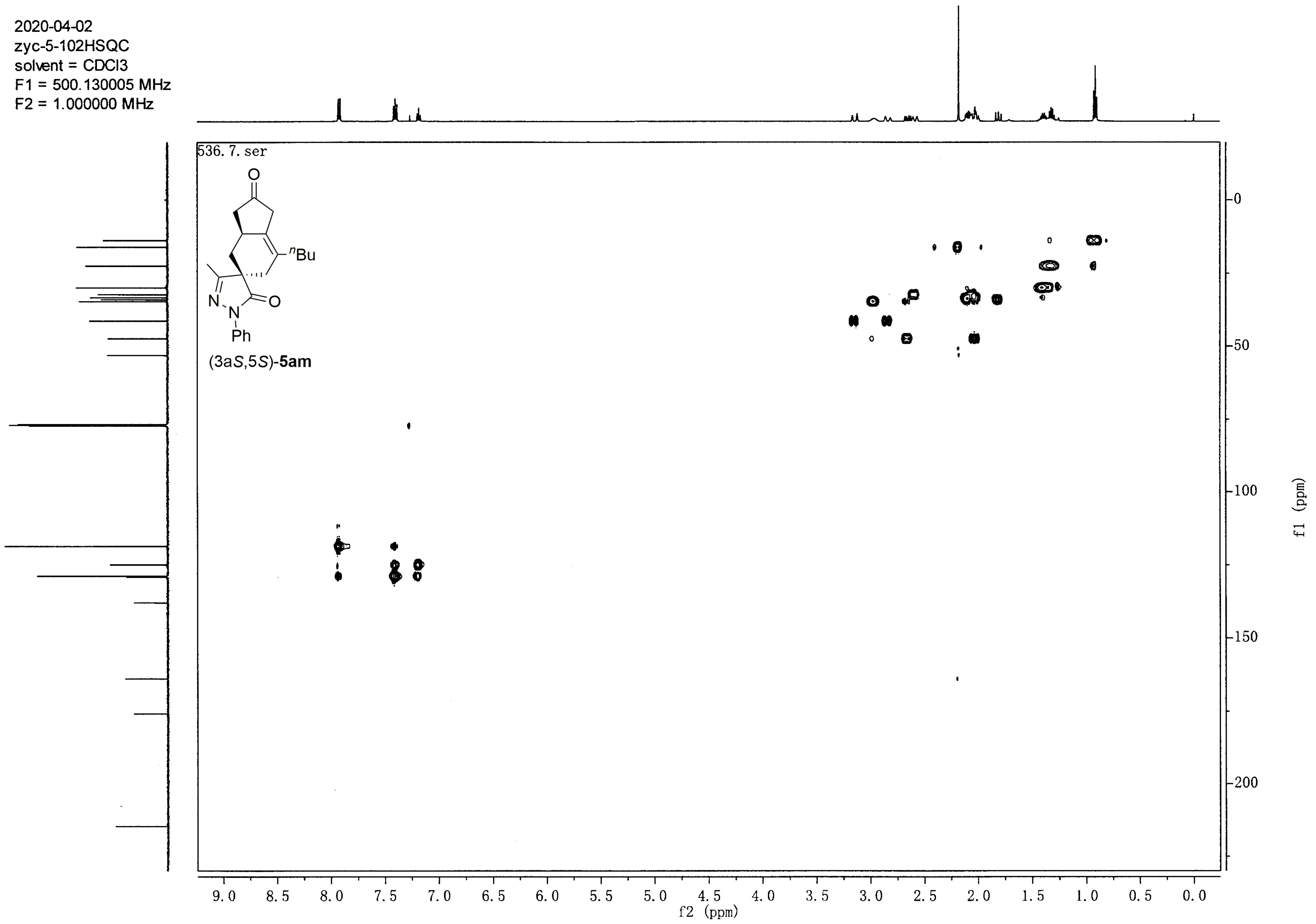

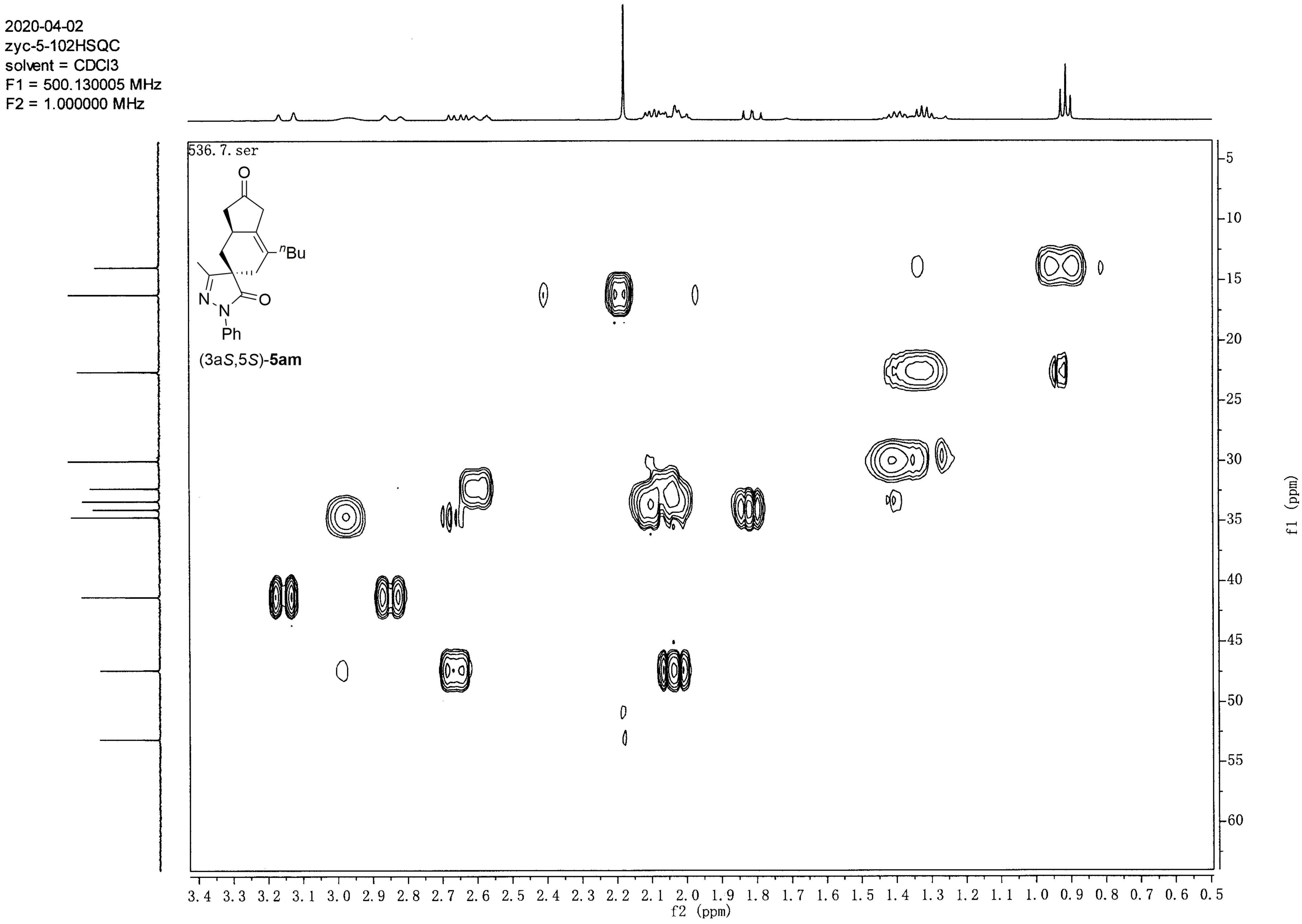
2020-04-02
zyc-5-102HSQC
solvent = CDCl3
F1 = 500.130005 MHz
F2 = 1.000000 MHz
536.7.ser
(3aS,5S)-5am
f2 (ppm)
f1 (ppm)

zyc-4-193

实验时间：2019-10-29,9:27:36
谱图文件:D:\浙大智达\N2000\样品\S20191029092736.org
方法文件:D:\浙大智达\N2000\djx.mtd

实验者：zyc
报告时间：2019-10-29,10:48:57
积分方法:面积归一法

实验内容简介：
ia,n-hexane/i-PrOH = 90/10,1.0,254

色谱图(S20191029092736.org)

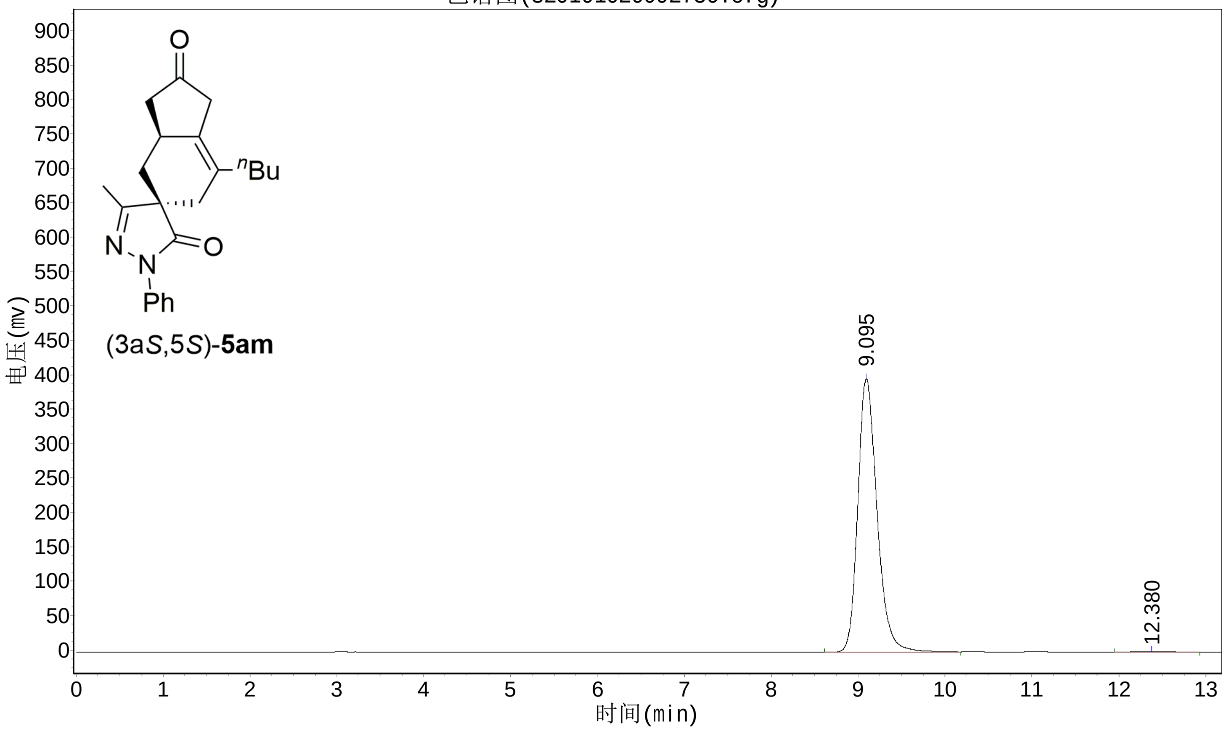

分析结果表

峰号	峰名	保留时间	峰高	峰面积	含量
1		9.095	397750.719	6168396.000	99.4130
2		12.380	1293.532	36423.801	0.5870
总计			399044.251	6204819.801	100.0000

zyc-4-193mix

实验时间：2019-10-29,11:00:21
谱图文件:D:\浙大智达\N2000\样品\S20191029110021.org
方法文件:D:\浙大智达\N2000\djx.mtd

实验者：zyc
报告时间：2019-10-29,11:15:42
积分方法:面积归一法

实验内容简介：
ia,n-hexane/i-PrOH = 90/10,1.0,254

色谱图(S20191029110021.org)

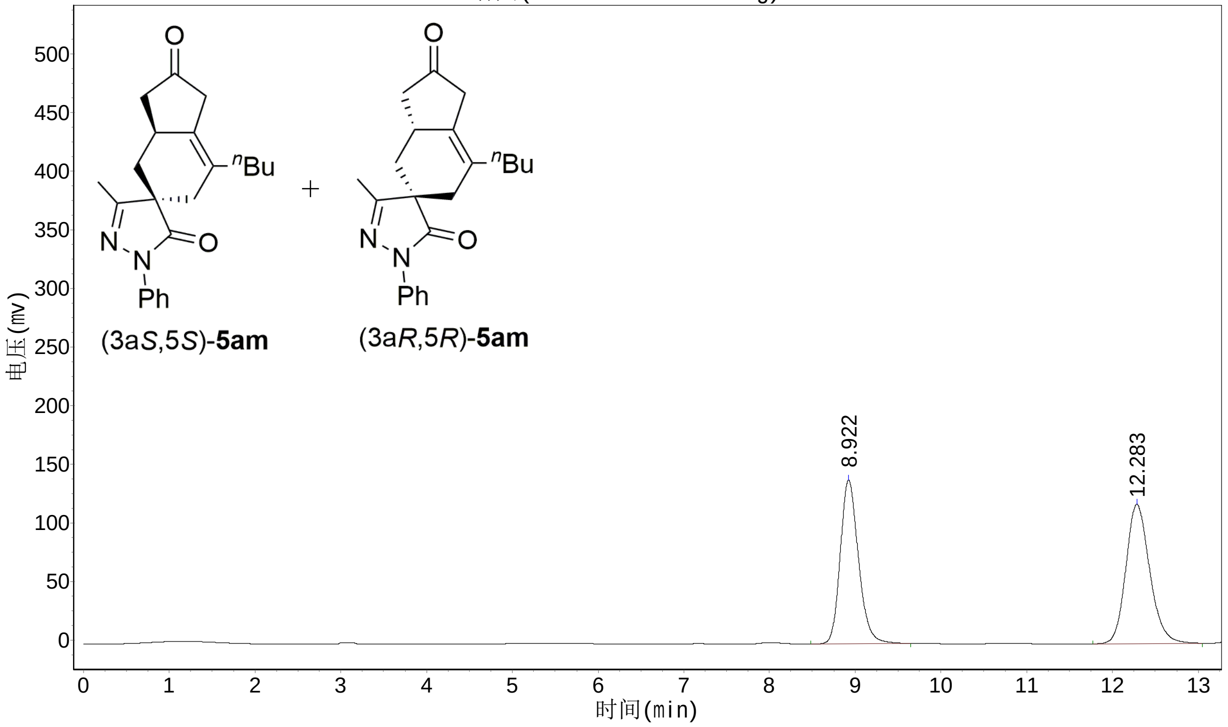

分析结果表

峰号	峰名	保留时间	峰高	峰面积	含量
1		8.922	139504.203	2066846.375	47.2562
2		12.283	118925.766	2306856.250	52.7438
总计			258429.969	4373702.625	100.0000

^{1}H NMR

2020-07-21 17:58:06.984
zyc-6-31
NA = 8
Solvent = CDCl3
F1 = 300.130005 MHz
F2 = 1.000000 MHz

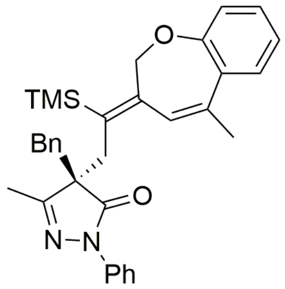

(*S*,*Z*)-**6da**

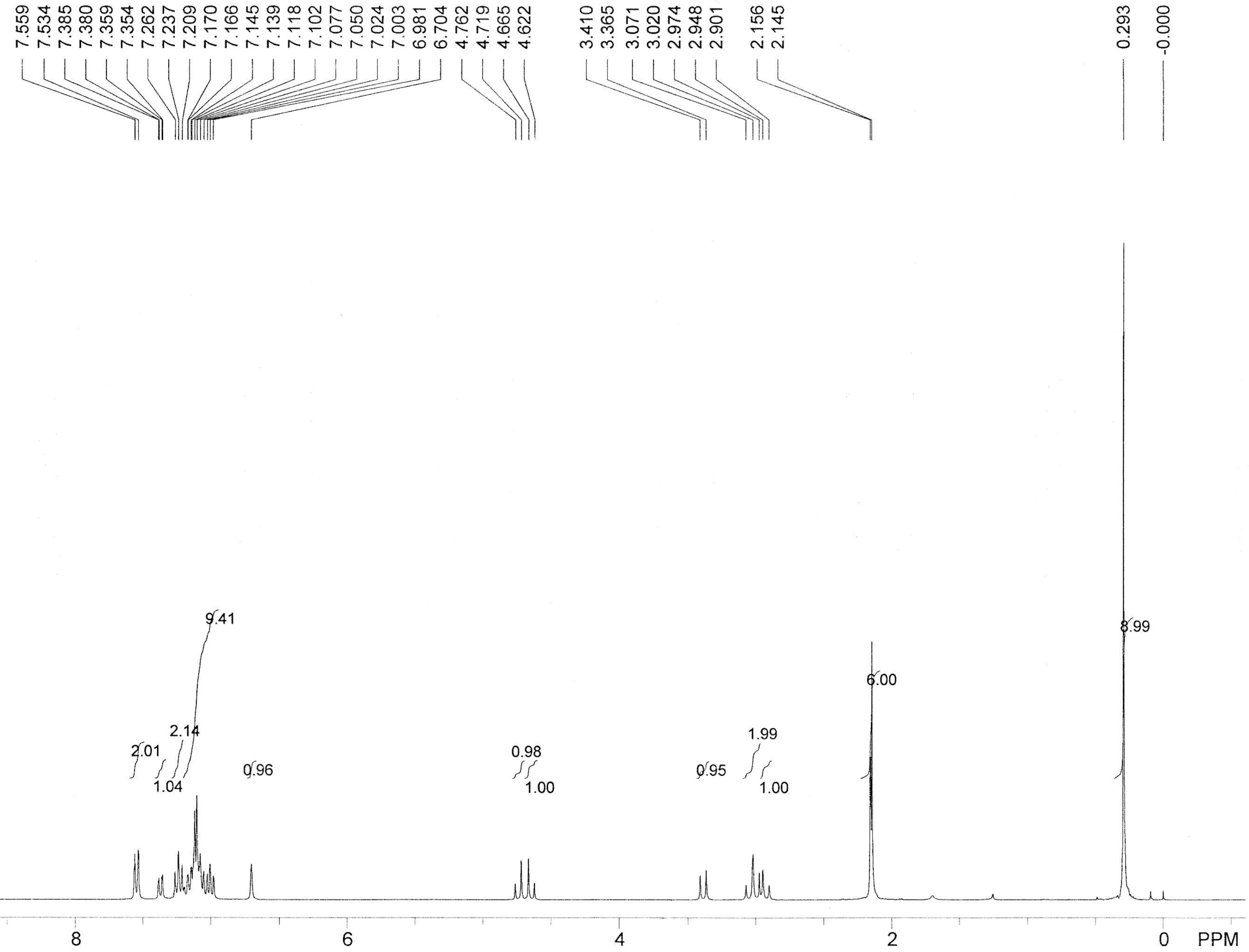

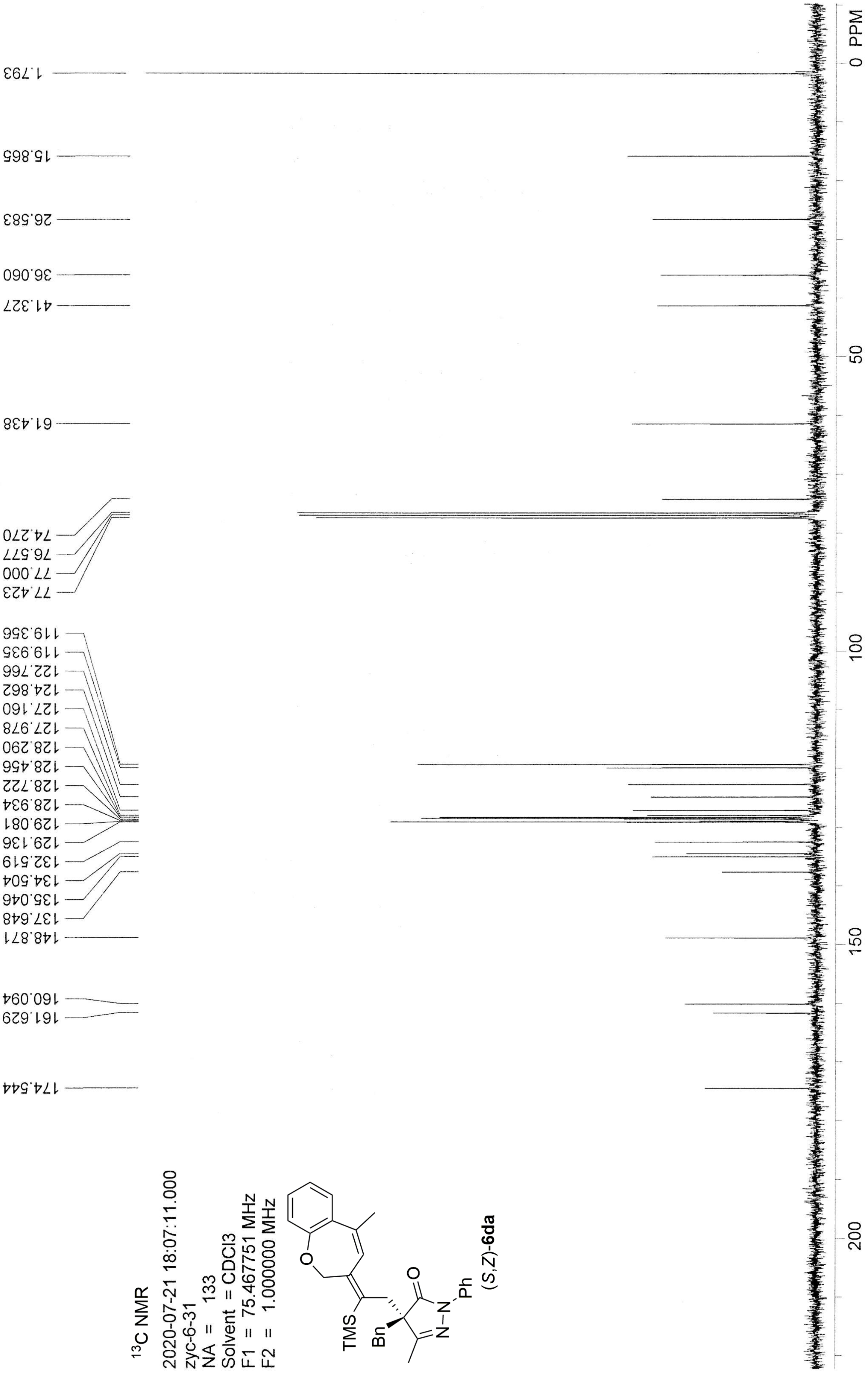

^{13}C NMR
2020-07-21 18:07:11.000
zyc-6-31
NA = 133
Solvent = CDCl3
F1 = 75.467751 MHz
F2 = 1.000000 MHz
TMS
Bn
O
N
N
Ph
(S,Z)-6da
1.793
15.865
26.583
36.060
41.327
61.438
74.270
76.577
77.000
77.423
119.356
119.935
122.766
124.862
127.160
127.978
128.290
128.456
128.722
128.934
129.081
129.136
132.519
134.504
135.046
137.648
148.871
160.094
161.629
174.544
0 PPM
50
100
150
200

zyc-6-31

实验时间：2020-07-23,18:42:58
谱图文件:D:\浙大智达\N2000\样品\S20200723184258.org
方法文件:D:\浙大智达\N2000\djx.mtd

实验者：zyc
报告时间：2020-07-23,18:53:12
积分方法:面积归一法

实验内容简介：
ia,n-hexane/i-PrOH = 90/10,1.0,254

色谱图(S20200723184258.org)

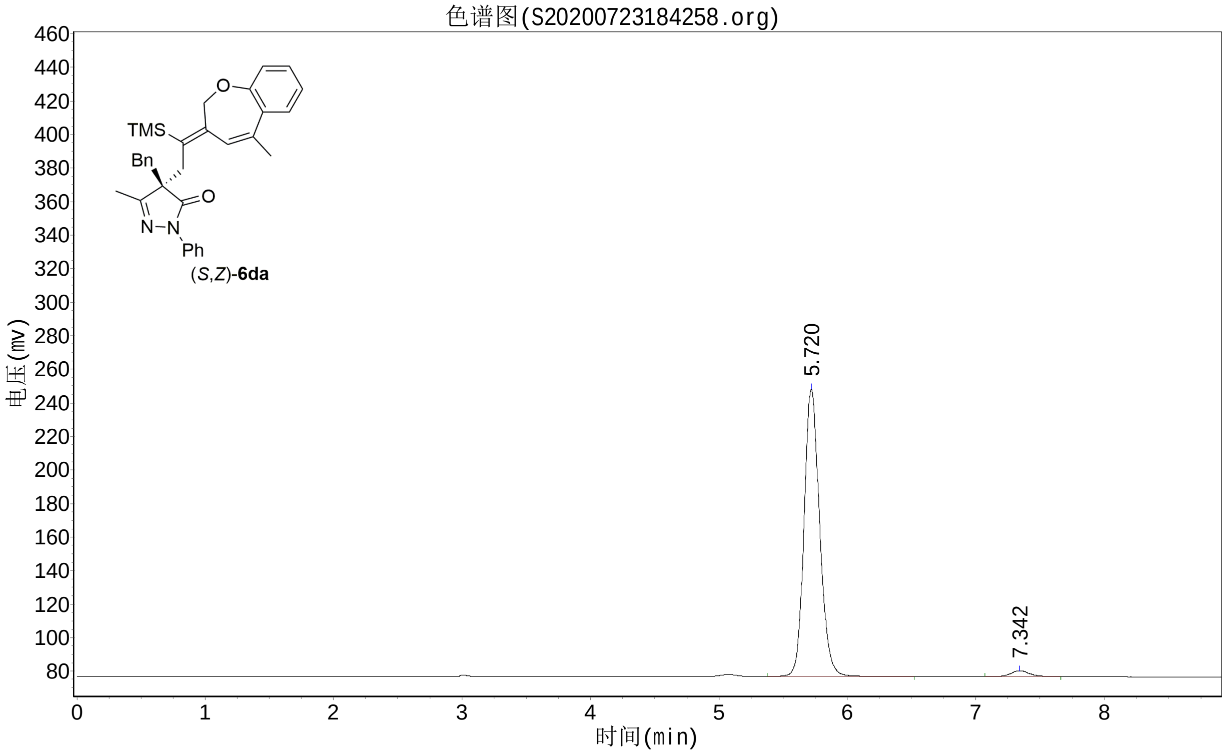

分析结果表

峰号	峰名	保留时间	峰高	峰面积	含量
1		5.720	171376.938	1454788.625	97.5143
2		7.342	3465.814	37083.910	2.4857
总计			174842.752	1491872.535	100.0000

zyc-6-31mix

实验时间：2020-07-23,18:55:19
谱图文件:D:\浙大智达\N2000\样品\S20200723185519.org
方法文件:D:\浙大智达\N2000\djx.mtd

实验者：zyc
报告时间：2020-07-23,19:06:02
积分方法:面积归一法

实验内容简介：
ia,n-hexane/i-PrOH = 90/10,1.0,254

色谱图(S20200723185519.org)

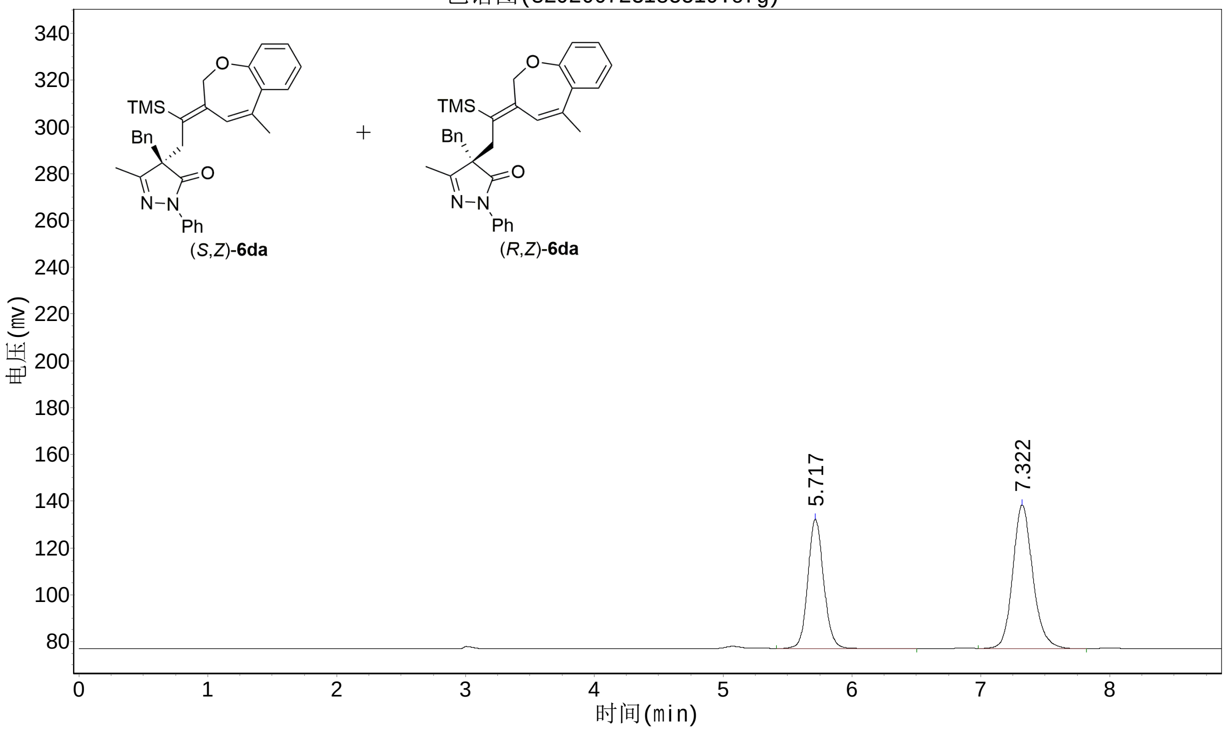

分析结果表

峰号	峰名	保留时间	峰高	峰面积	含量
1		5.717	55618.082	479877.375	41.6889
2		7.322	61551.066	671212.813	58.3111
总计			117169.148	1151090.188	100.0000

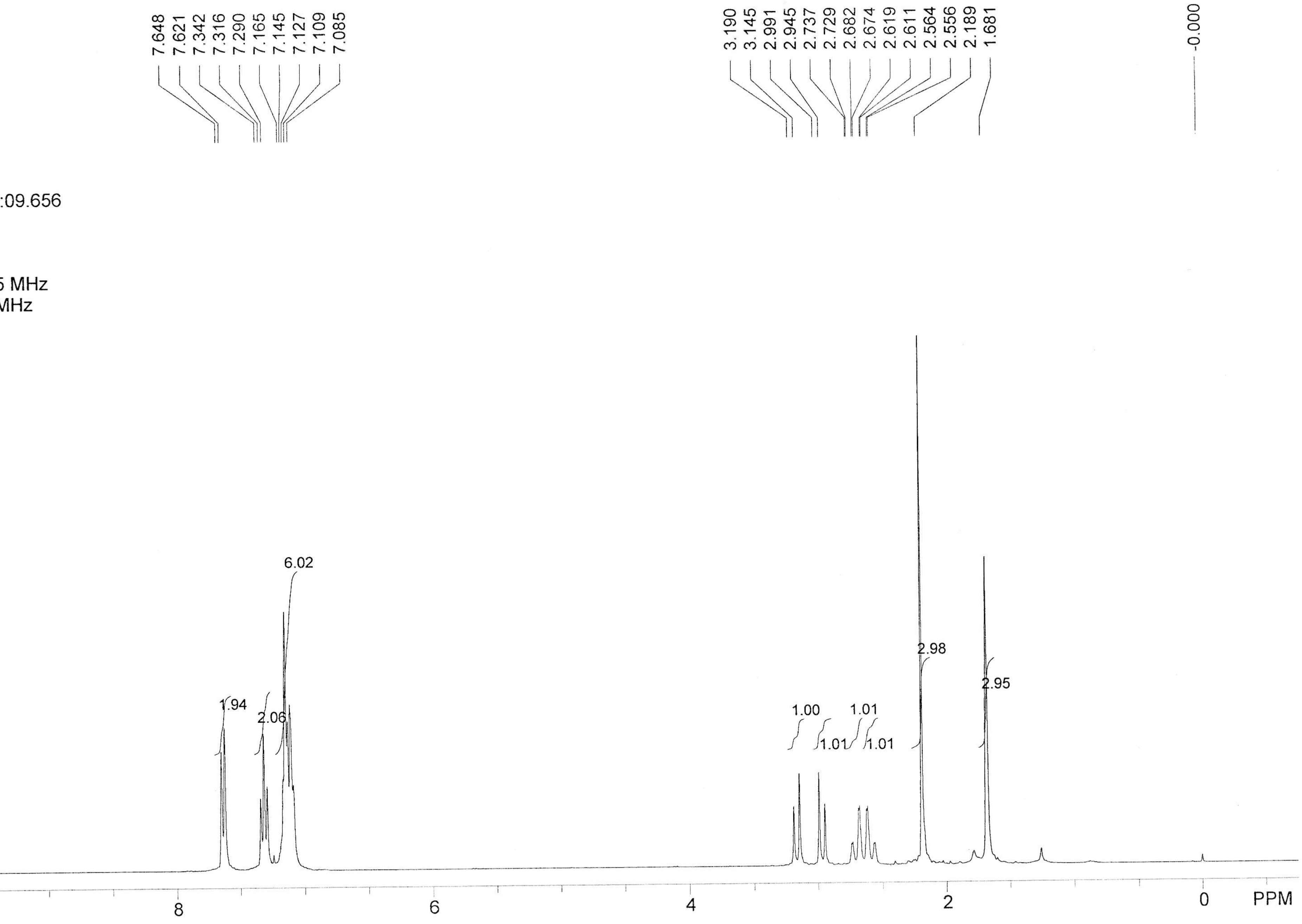

^{1}H NMR
2020-07-24 10:57:09.656
zyc-6-40
NA = 8
Solvent = CDCl3
F1 = 300.130005 MHz
F2 = 1.000000 MHz

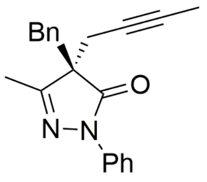

(*S*)-**7da**

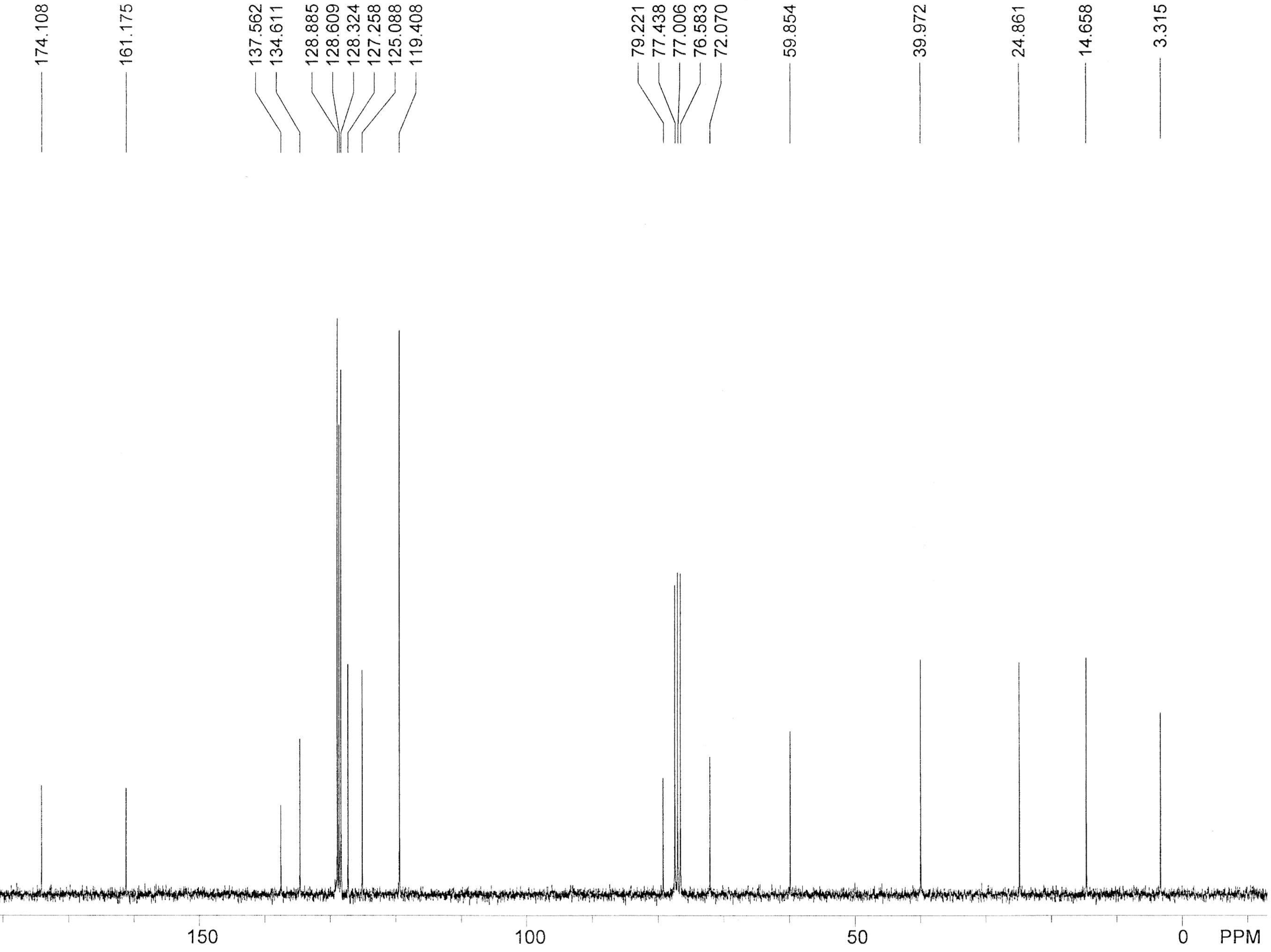

^{13}C NMR

2020-07-24 11:04:50.781
zyc-6-40
NA = 115
Solvent = CDCl3
F1 = 75.467751 MHz
F2 = 1.000000 MHz

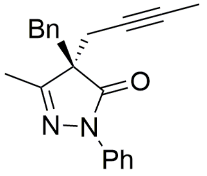

(*S*)-**7da**

zyc-6-40

实验时间：2020-07-24,9:51:39
谱图文件:D:\浙大智达\N2000\样品\S20200724095139.org
方法文件:D:\浙大智达\N2000\djx.mtd

实验者：zyc
报告时间：2020-07-24,10:29:08
积分方法:面积归一法

实验内容简介：
ia,n-hexane/i-PrOH = 90/10,1.0,254

色谱图(S20200724095139.org)

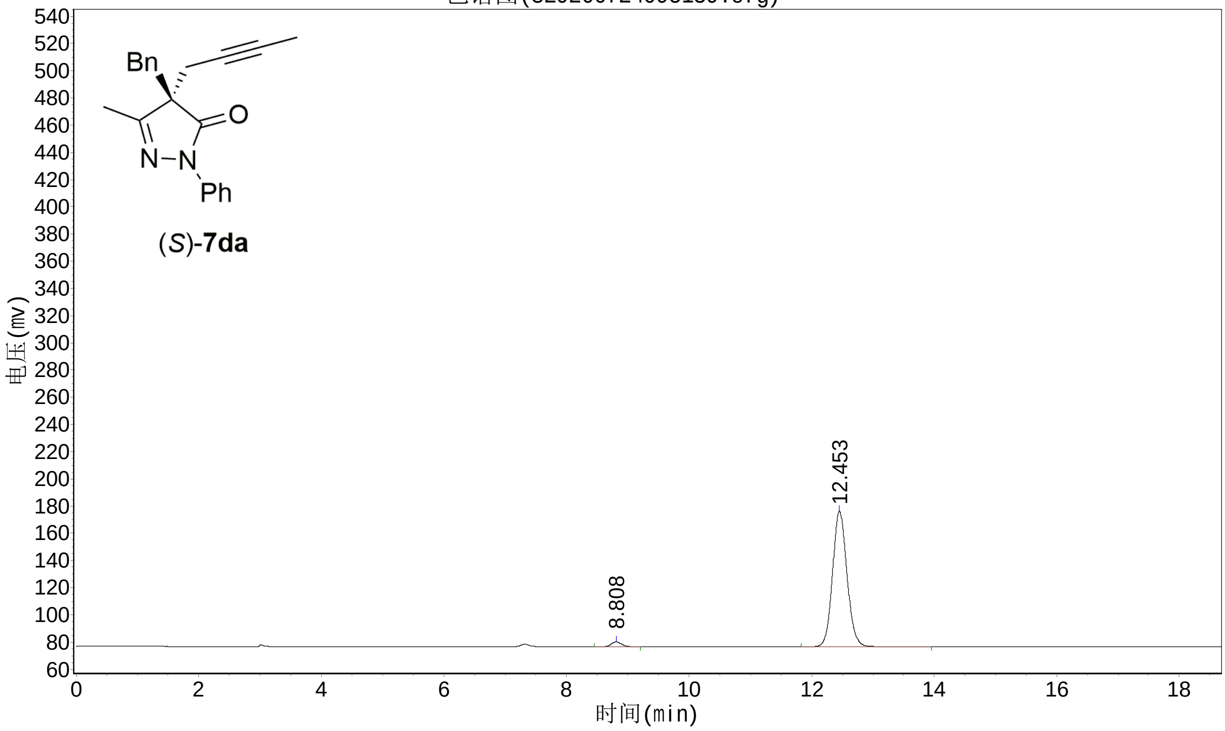

分析结果表

峰号	峰名	保留时间	峰高	峰面积	含量
1		8.808	3683.839	42586.055	2.5279
2		12.453	99910.820	1642033.750	97.4721
总计			103594.659	1684619.805	100.0000

zyc-6-40mix

实验时间：2020-07-24,10:38:50
谱图文件:D:\浙大智达\N2000\样品\S20200724103850.org
方法文件:D:\浙大智达\N2000\djx.mtd

实验者：zyc
报告时间：2020-07-24,11:25:46
积分方法:面积归一法

实验内容简介：
ia,n-hexane/i-PrOH = 90/10,1.0,254

色谱图(S20200724103850.org)

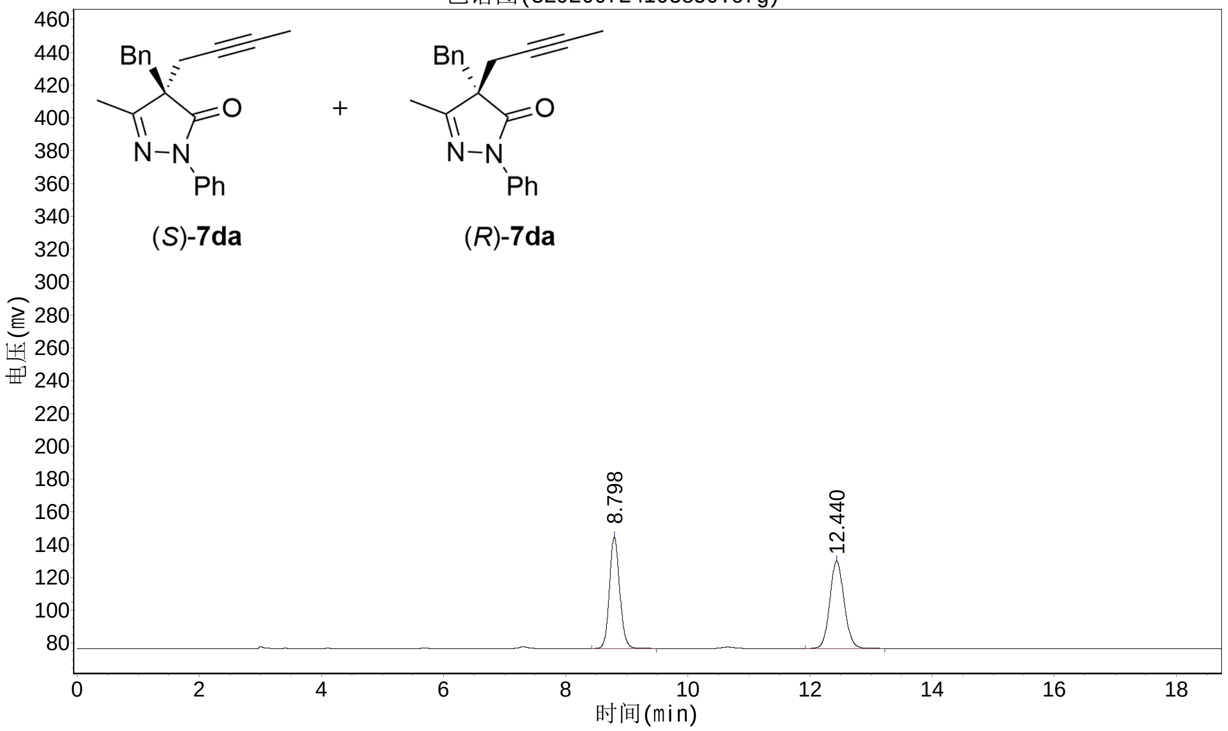

分析结果表

峰号	峰名	保留时间	峰高	峰面积	含量
1		8.798	67912.063	794214.188	47.5845
2		12.440	53559.273	874846.250	52.4155
总计			121471.336	1669060.438	100.0000

^{1}H NMR
2020-06-30 14:41:05.265
zyc-4-198re
NA = 8
Solvent = CDCl3
F1 = 300.130005 MHz
F2 = 1.000000 MHz

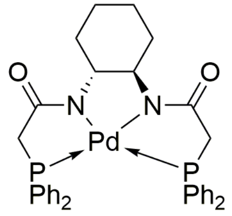

Pd(II)-**DACH-ZYC-Phos-C1**

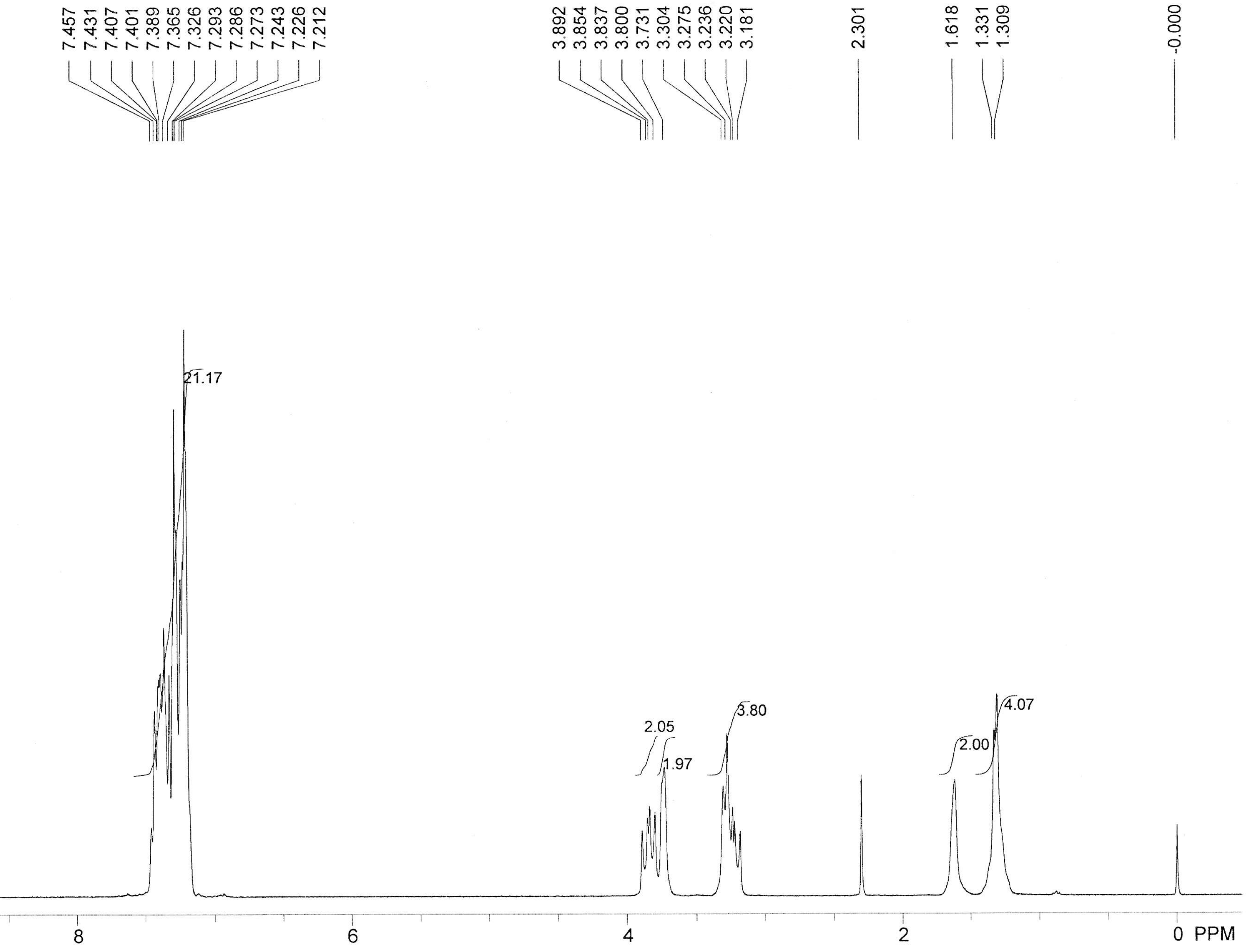

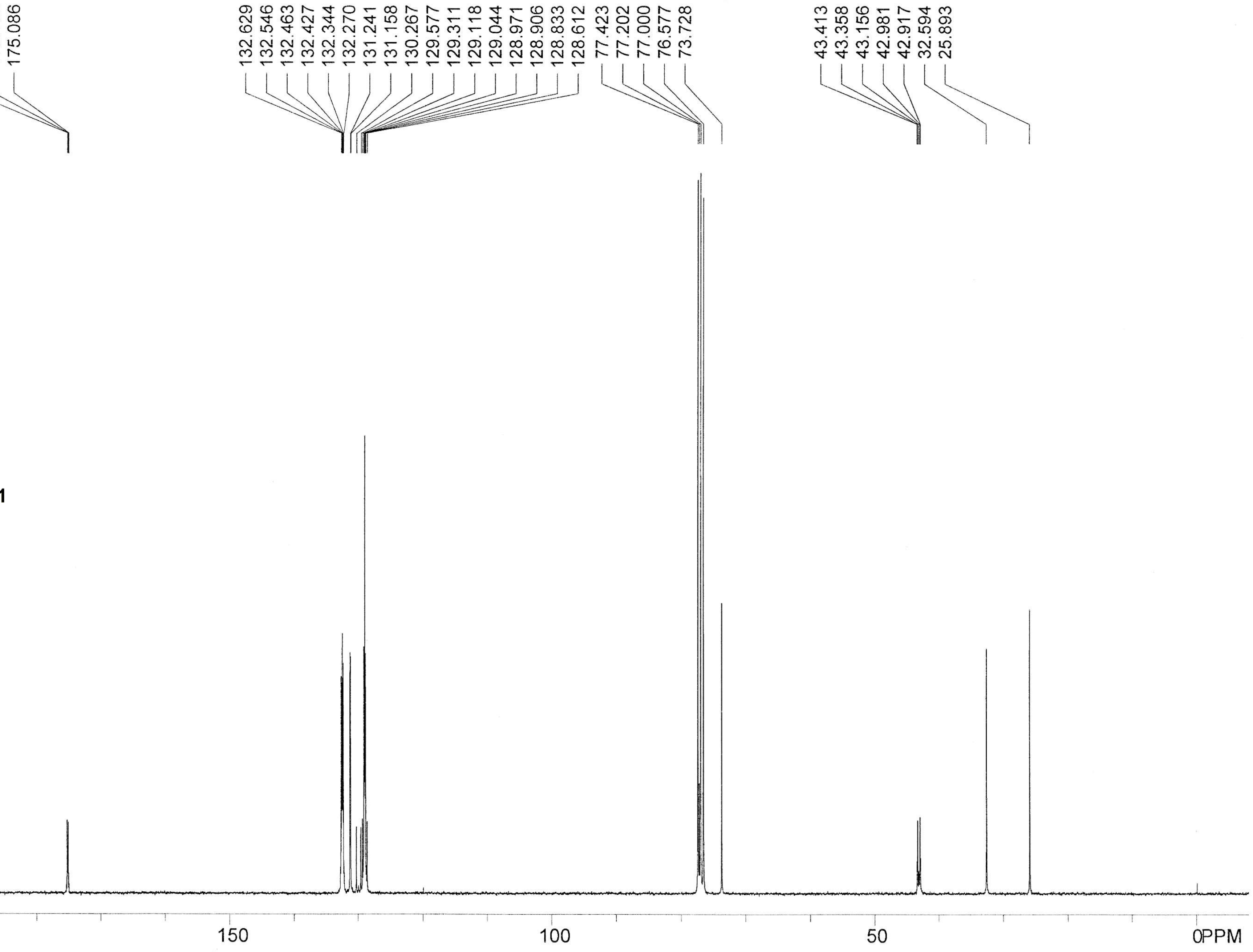

^{13}C NMR

2020-07-02 08:39:04.375
zyc-4-198re
NA = 10707
Solvent = CDCl3
F1 = 75.467751 MHz
F2 = 1.000000 MHz

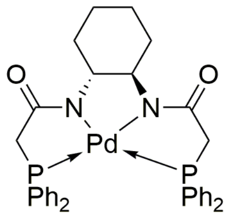

Pd(II)-**DACH-ZYC-Phos-C1**

^{13}C NMR

2020-07-02 08:39:04.375
zyc-4-198re
NA = 10707
Solvent = CDCl3
F1 = 75.467751 MHz
F2 = 1.000000 MHz

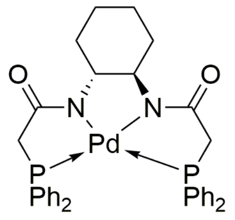

Pd(II)-**DACH-ZYC-Phos-C1**

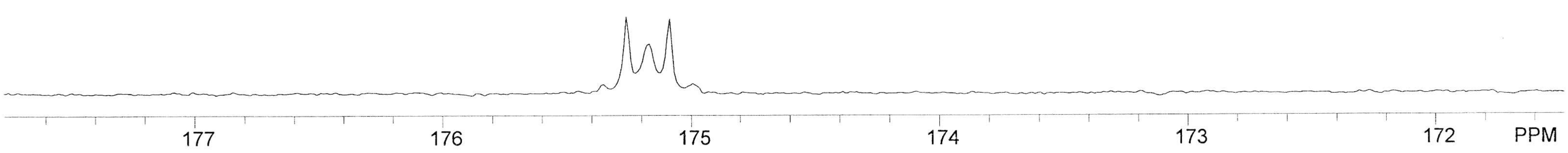

^{13}C NMR

2020-07-02 08:39:04.375
zyc-4-198re
NA = 10707
Solvent = CDCl3
F1 = 75.467751 MHz
F2 = 1.000000 MHz

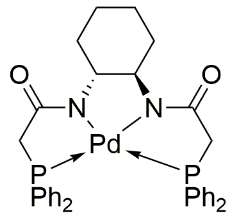

Pd(II)-**DACH-ZYC-Phos-C1**

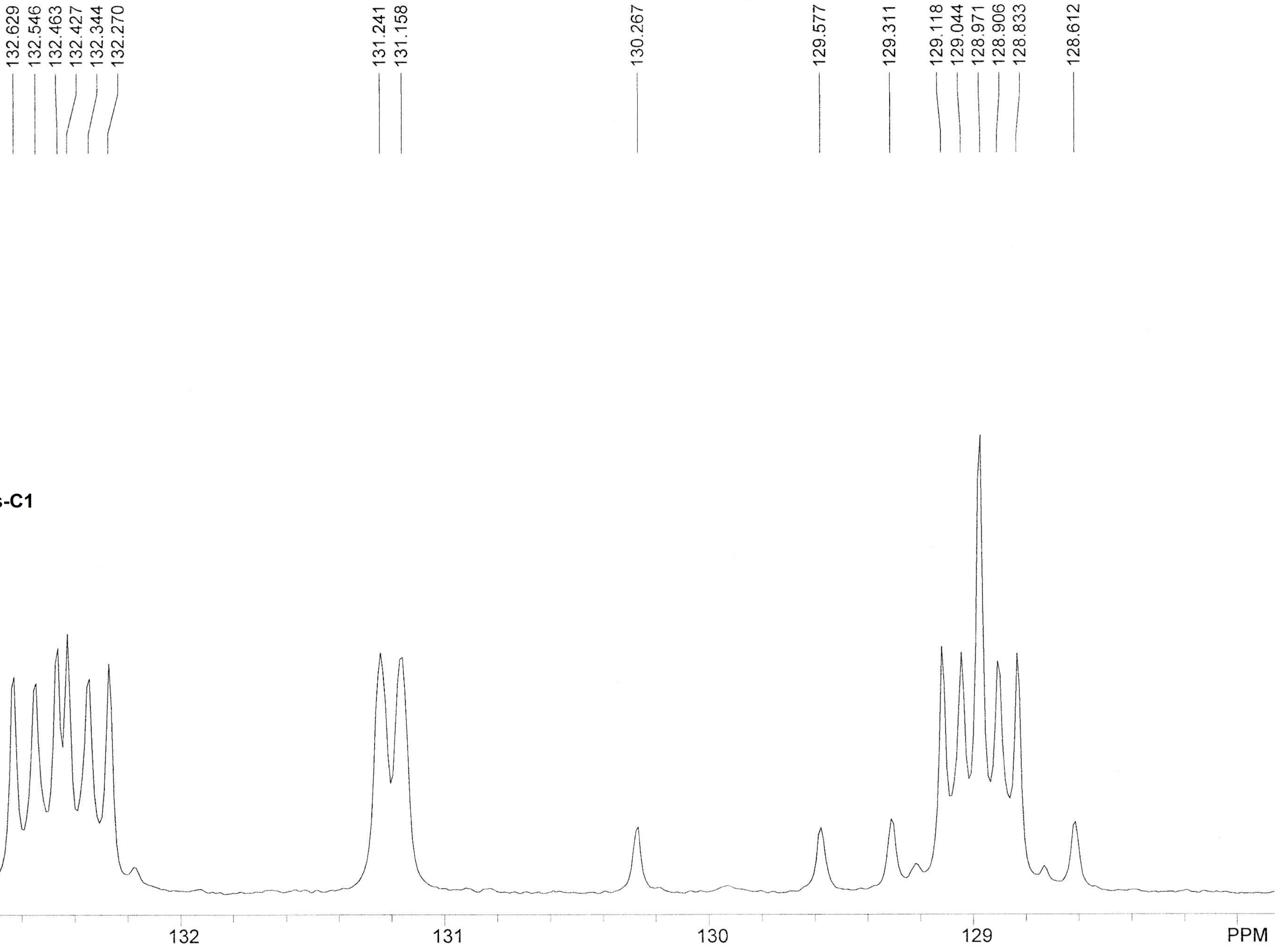

^{13}C NMR

2020-07-02 08:39:04.375
zyc-4-198re
NA = 10707
Solvent = CDCl3
F1 = 75.467751 MHz
F2 = 1.000000 MHz

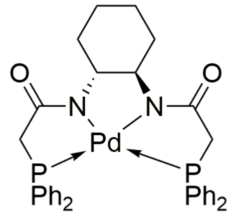

Pd(II)-**DACH-ZYC-Phos-C1**

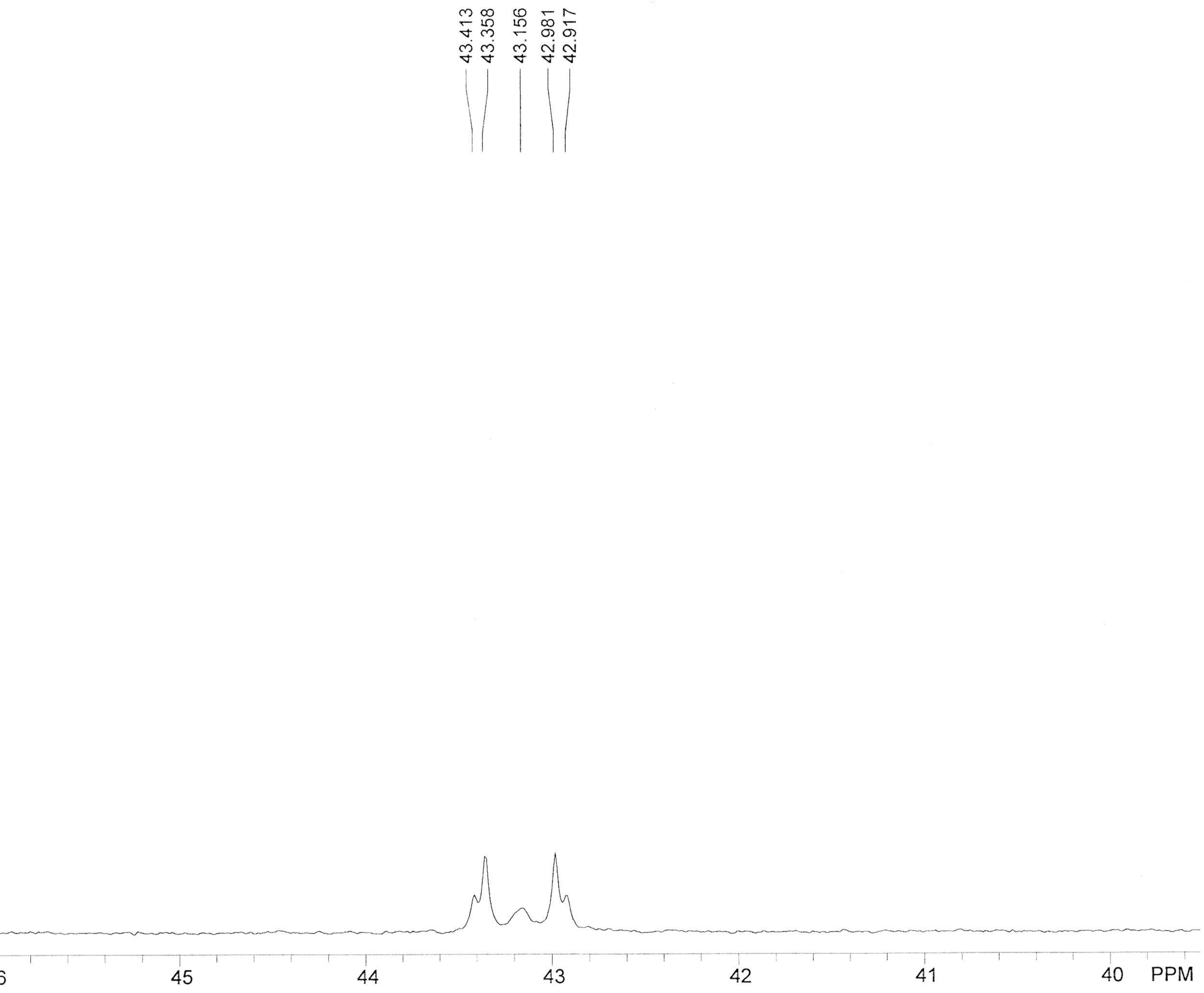

^{31}P NMR

2020-06-30 14:44:36.203
zyc-4-198re
NA = 16
Solvent = CDCl3
F1 = 121.494850 MHz
F2 = 1.000000 MHz

Pd(II)-**DACH-ZYC-Phos-C1**

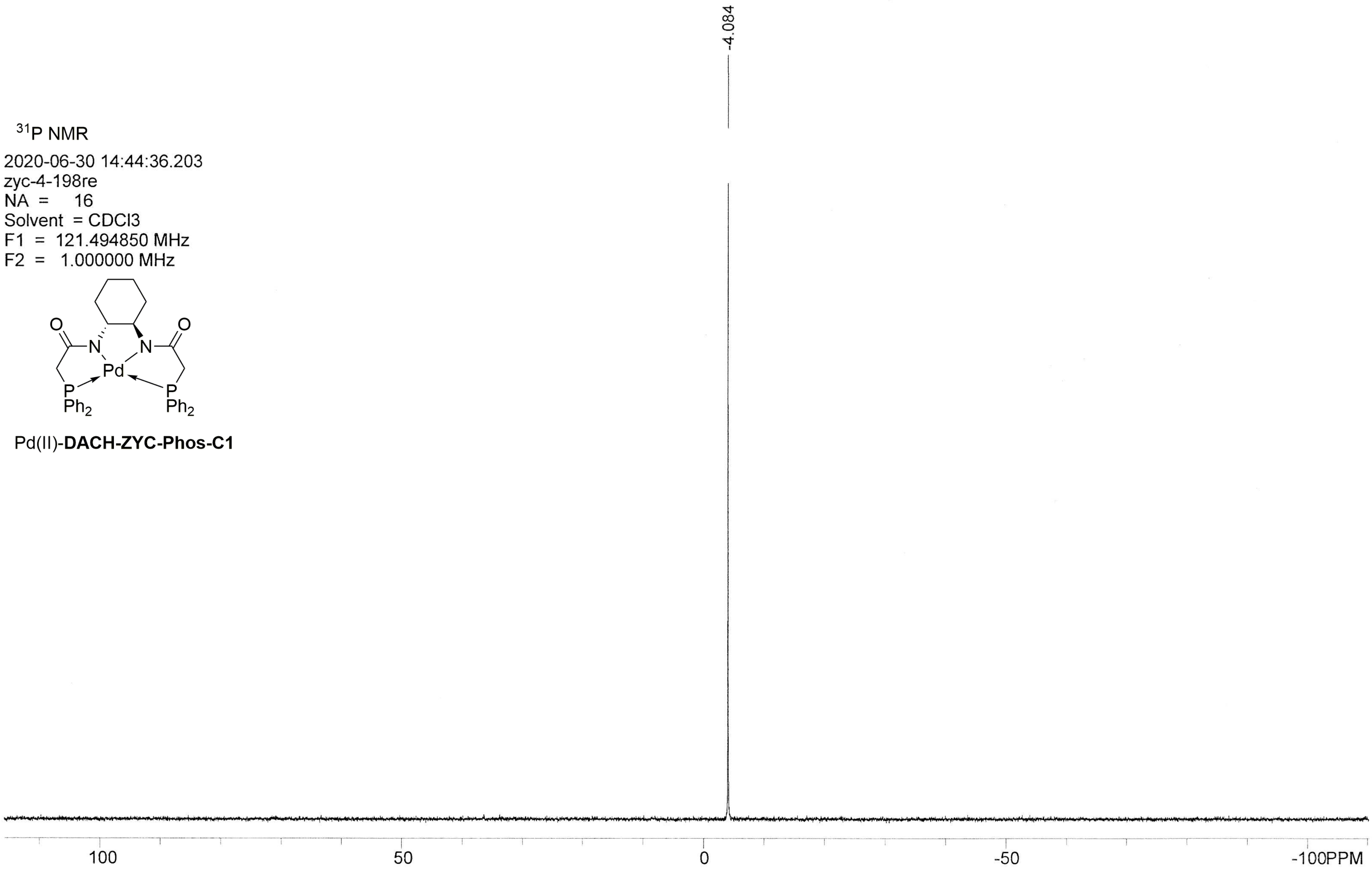

Supplementary References

1 Zhu, T. & Ma, S. 3,4-Alkadienyl ketones via the palladium-catalyzed decarboxylative allenylation of 3-oxocarboxylic acids. *Chem. Commun.* **53**, 6037-6040 (2017).

2 Rioz-Martínez, A., Cuetos, A., Rodriguéz, C., Gonzalo, G., Lavandera, I., Fraaije, M. W. & Gotor, W. Dynamic Kinetic Resolution of α-Substituted β-Ketoesters Catalyzed by Baeyer–Villiger Monooxygenases: Access to Enantiopure α-Hydroxy Esters. *Angew. Chem. Int. Ed.* **50**, 8387-8390 (2011).

3 Nakagawa, H., Ohyama, R., Kimata, A., Suzuki, T. & Miyata, N. Hydroxyl radical scavenging by edaravone derivatives: Efficient scavenging by 3-methyl-1-(pyridin-2-yl)-5-pyrazolone with an intramolecular base. *Bioorg. Med. Chem. Lett.* **16**, 5939-5942 (2006).

4 Tao, Z.-L., Zhang, W.-Q., Chen, D.-F., Adele, A. & Gong, L.-Z. Pd-Catalyzed Asymmetric Allylic Alkylation of Pyrazol-5-ones with Allylic Alcohols: The Role of the Chiral Phosphoric Acid in C−O Bond Cleavage and Stereocontrol. *J. Am. Chem. Soc.* **135**, 9255-9258 (2013).

5 Zhou, H., Wei, Z., Zhang, J., Yang, H., Xia, C. & Jiang, G. From Palladium to Brønsted Acid Catalysis: Highly Enantioselective Regiodivergent Addition of Alkoxyallenes to Pyrazolones. *Angew. Chem. Int. Ed.* **56**, 1077-1081 (2017).

6 Wang, Z., Chen, Z., Bai, S., Li, W., Liu, X., Lin, L. & Feng, X. Highly Z-Selective Asymmetric Conjugate Addition of Alkynones with Pyrazol-5-ones Promoted by N,N'-Dioxide–Metal Complexes. *Angew. Chem. Int. Ed.* **51**, 2776-2779 (2012).

7 Kimata, A., Nakagawa, H., Ohyama, R., Fukuuchi, T., Ohta, S., Suzuki, T. & Miyata,

N. New Series of Antiprion Compounds: Pyrazolone Derivatives Have the Potent Activity of Inhibiting Protease-Resistant Prion Protein Accumulation. *J. Med. Chem.* **50**, 5053-5056 (2007).

8 Tsvetkov, E. N., Bondarenko, N. A., Malakhova, I. G. & Kabachnik, M. I. A Simple Synthesis and Some Synthetic Application of Substituted Phosphide and Phosphinite Anions. *Synthesis* 198-208 (1986).

9 Guisado-Barrios, G., Muñoz, B. K., Kamer, P. C. J., Lastdrager, B., Marel, G., Overhand, M., Vega-Vázquez, M. & Martin-Pastor, M. Cyclic decapeptide gramicidin S derivatives containing phosphines: novel ligands for asymmetric catalysis. *Dalton Trans.* **42**, 1973-1978 (2013).

10 Khalil, A., Hassan, M., Mohamed, M. & El-Sayed, A. Phase-Transfer Catalyzed Alkylation and Cycloalkylation of 3-Substituted-1H-pyrazol-2-in-5-ones in the Absence or Presence of Carbon Disulphide. *Phosphorus, Sulfur, and Silicon*, **180**, 479-496 (2005).

11 Frisch, M. J., Trucks, G. W., Schlegel, H. B., Scuseria, G. E., Robb, M. A., Cheeseman, J. R., Scalmani, G., Barone, V., Mennucci, B., Petersson, G. A., Nakatsuji, H., Caricato, M., Li, X., Hratchian, H. P., Izmaylov, A. F., Bloino, J., Zheng, G., Sonnenberg, J. L., Hada, M., Ehara, M., Toyota, K., Fukuda, R., Hasegawa, J., Ishida, M., Nakajima, T., Honda, Y., Kitao, O., Nakai, H., Vreven, T., Montgomery, J. A., Jr., J. E, P., Ogliaro, F., Bearpark, M., Heyd, J. J., Brothers, E., Kudin, K. N., Staroverov, V. N., Keith, T., Kobayashi, R., Normand, J., Raghavachari, K., Rendell, A., Burant, J. C., Iyengar, S. S., Tomasi, J., Cossi, M., Rega, N., Millam, J. M., Klene, M., Knox, J.

E., Cross, J. B., Bakken, V., Adamo, C., Jaramillo, J., Gomperts, R., Stratmann, R. E., Yazyev, O., Austin, A. J., Cammi, R., Pomelli, C., Ochterski, J. W., Martin, R. L., Morokuma, K., Zakrzewski, V. G., Voth, G. A., Salvador, P., Dannenberg, J. J., Dapprich, S., Daniels, A. D., Farkas, O., Foresman, J. B., Ortiz, J. V., Cioslowski, J. & Fox, D. J. Gaussian 09, Revision D.01, Gaussian, Inc., Wallingford, CT, (2009).

12 Lee, C., Yang, W. & Parr, R. G. Development of the Colle-Salvetti correlation-energy formula into a functional of the electron density. *Phys. Rev. B: Condens. Matter Mater. Phys.* **37**, 785-789 (1988).

13 Becke, A. D. Density-functional thermochemistry. III. The role of exact exchange. *J. Chem. Phys.* **98**, 5648-5652 (1993).

14 Johnson, E. R. & Becke, A. D. A post-Hartree–Fock model of intermolecular interactions. *J. Chem. Phys.* **123**, 024101 (2005).

15 Grimme, S., Antony, J., Ehrlich, S. & Krieg, H. A consistent and accurate ab initio parametrization of density functional dispersion correction (DFT-D) for the 94 elements H-Pu. *J. Chem. Phys.* **132**, 154104 (2010).

16 Grimme, S., Ehrlich, S. & Goerigk, L. Effect of the Damping Function in Dispersion Corrected Density Functional Theory. *J. Comput. Chem.* **32**, 1456-1465 (2011).

17 Hay, P. J. & Wadt, W. R. Ab initio effective core potentials for molecular calculations. Potentials for the transition metal atoms Sc to Hg. *J. Chem. Phys.* **82**, 270-283 (1985).

18 Wadt, W. R. & Hay, P. J. Ab initio effective core potentials for molecular

calculations. Potentials for main group elements Na to Bi. *J. Chem. Phys.* **82**, 284-298 (1985).

19 Hay, P. J. & Wadt, W. R. Ab initio effective core potentials for molecular calculations. Potentials for K to Au including the outermost core orbitals. *J. Chem. Phys.* **82**, 299-310 (1985).

20 Hariharan, P. C. & Pople, J. A. The Influence of Polarization Functions on Molecular Orbital Hydrogenation Energies. *Theor. Chim. Acta* **28**, 213-222 (1973).

21 Hehre, W. J., Ditchfield, R. & Pople, J. A. Self—Consistent Molecular Orbital Methods. XII. Further Extensions of Gaussian—Type Basis Sets for Use in Molecular Orbital Studies of Organic Molecules. *J. Chem. Phys.* **56**, 2257-2261 (1972).

22 Fukui, K. A Formulation of the Reaction Coordinate. *J. Phys. Chem.* **74**, 4161-4163 (1970).

23 Gonzalez, C. & Schlegel, H. B. An improved algorithm for reaction path following. *J. Chem. Phys.* **90**, 2154-2161 (1989).

24 Gonzalez, C. & Schlegel, H. B. Reaction Path Following In Mass-Weighted Internal Coordinates. *J. Phys. Chem.* **94**, 5523-5527 (1990).

25 Zhao, Y. & Truhlar, D. G. Density Functionals with Broad Applicability in Chemistry. *Acc. Chem. Res.* **41**, 157-167 (2008).

26 Zhao Y. & Truhlar, D. G. The M06 suite of density functionals for main group thermochemistry, thermochemical kinetics, noncovalent interactions, excited states, and transition elements: two new functionals and systematic testing of four M06-class functionals and 12 other functionals. *Theor. Chem. Acc.* **120**, 215-241 (2008).

27 Andrae, D., Häußermann, U., Dolg, M., Stoll H. & Preuß, H. Energy-adjusted ab initio pseudopotentials for the second and third row transition elements. *Theor. Chim. Acta* **77**, 123-141 (1990).

28 Scalmani G. & Frisch, M. J. Continuous surface charge polarizable continuum models of solvation. I. General formalism. *J. Chem. Phys.* **132,** 114110 (2010).

29 Legault, C. Y. *CYLview*, 1.0b, Universitéde Sherbrooke, Montreal, (2009), http://www.Cylview.org.

30 Lu, T. & Chen, F. Multiwfn: A Multifunctional Wavefunction Analyzer. *J. Comput. Chem.* **33***,* 580-592 (2012).

31 Lu, T. *Multiwfn*, version 3.5.

32 Humphrey, W., Dalke, A. & Schulten, K. VMD: Visual Molecular Dynamics. *J. Mol. Graph.* **14**, 33-38 (1996).
